# Non-linear associations between meteorological factors, ambient air pollutants and major mosquito-borne diseases in Thailand

**DOI:** 10.1371/journal.pntd.0011763

**Published:** 2023-12-27

**Authors:** Pranav Tewari, Pei Ma, Gregory Gan, A. Janhavi, Esther Li Wen Choo, Joel Ruihan Koo, Borame Lee Dickens, Jue Tao Lim

**Affiliations:** 1 Lee Kong Chian School of Medicine, Nanyang Technological University, Singapore, Singapore; 2 Saw Swee Hock School of Public Health, National University of Singapore, Singapore, Singapore; 3 Department of Biological Sciences, National University of Singapore, Singapore, Singapore; Columbia University, UNITED STATES

## Abstract

**Background:**

Transmission intensity for mosquito-borne diseases are highly heterogenous and multi-factorial. Understanding risk factors associated to disease transmission allow the optimization of vector control. This study sets out to understand and compare the combined anthropogenic and environmental risk factors of four major mosquito-borne diseases, dengue, malaria, chikungunya and Japanese encephalitis in Thailand.

**Methods:**

An integrated analysis of mosquito-borne diseases, meteorological and ambient air pollutants of 76 provinces of Thailand was conducted over 2003–2021. We explored the use of generalized linear models and generalized additive models to consider both linear and non-linear associations between meteorological factors, ambient air pollutants and mosquito-borne disease incidence. Different assumptions on spatio-temporal dependence and nonlinearity were considered through province-specific and panel models, as well as different spline functions. Disease-specific model evidence was assessed to select best-fit models for epidemiological inference downstream.

**Results:**

Analyses indicated several findings which can be generally applied to all diseases explored: **(1)** higher AH above mean values was positively associated with disease case counts **(2)** higher total precipitation above mean values was positively associated with disease case counts **(3)** extremely high temperatures were negatively associated with disease case counts **(4)** higher SO_2_ and PM_2.5_ surface concentrations were negatively associated with disease case counts. However, the relationships between disease and RH, non-extreme temperatures and CO surface concentration were more mixed, with directions of associations changing across the different diseases considered.

**Conclusions:**

This study found protective and enhancing effects of meteorological and ambient air pollutant factors on mosquito-borne diseases burdens in Thailand. Further studies should employ these factors to understand and predict risk factors associated with mosquito-borne disease transmission.

## Author summary

In this study, we examined the associations between meteorological variables, ambient air pollutants and four major mosquito-borne diseases, dengue, malaria, chikungunya and Japanese encephalitis in Thailand. For the optimization of vector control, it is pertinent to understand risk factors associated to disease transmission. Given the complex transmission patterns of mosquito-borne diseases, risk factors are known to be multi-factorial in nature. Although associations between climate variables and mosquito-borne diseases have been studied extensively, the role of ambient air pollutants in mosquito-borne disease dynamics remains relatively unexplored. Recognizing that urban areas experience unique challenges related to air quality and that pollutants can have a significant impact on ecological systems, it is imperative to understand the interplay between climate variables and pollutants comprehensively. Therefore, we aim to begin addressing this gap by determining the risk factors driving multiple major mosquito-borne diseases in Thailand from 2003 to 2021. By doing so, we aim to provide valuable insights into the complex dynamics of disease transmission in urban settings, ultimately contributing to more effective public health interventions and strategies.

## Introduction

Many mosquito-borne diseases are increasing in incidence and geographical distribution, reinvading into areas once previously eradicated and emerging in new regions [1]. Of particular concern is the current evidence which shows that the global incidence of *Aedes*-borne dengue virus (DENV) infections has increased by ~30 times over the past 50 years with ~95 million cases occurring per year at present, and that case numbers of Anopheles-borne malaria are also high at 105–200 million cases per year. General drivers of growing incidence and disease expansion include urbanisation, agricultural expansion and land use change such as deforestation [2]. Examples include urban expansion driving the increase in chikungunya virus (CHIKV) infections at ~700,000 cases per year due through *Ae*. *Aegypti* transmission, and agricultural-related dam creation which can increase the risk of Culex-borne Japanese encephalitis virus (JEV) transmission, currently standing at ~70,000 cases per year [3].

Several countries furthermore have burdens of multiple mosquito-borne diseases, causing extensive public health burdens, breaches in healthcare capacity, and substantial negative financial and societal effects across communities facing shortages in medical resources [1]. Thailand, as one of the largest nations in Southeast Asia, continually records DENV, malaria and JEV cases monthly across all provinces from 2000 to 2022, and sporadic chikungunya cases. Of note is the fact that humans serve as the primary reservoir facilitating pathogen transmission for DENV, malaria and CHIKV, for which transmission occurs within urban settings primarily due to the man-made creation of breeding habitats [4]. For JEV however, as pigs act as the primary host, urban pig farming is a key contributor with humans being infected by living in close proximity.

Treatment of these four diseases is additionally challenging. As of 2022, while pharmaceuticals are available to alleviate transmission and symptom presentation for malaria, drug resistance is a resurging problem for malaria in southeast Asia [5]. Whereas for DENV, the only available vaccine, Dengvaxia, has a complicated safety profile [6], although other promising antivirals and vaccines in development [6]. While a safe and effective vaccine is available for JEV [7], no other therapeutics are available for either chikungunya [8] and JEV [7] for susceptible individuals. For all four of these diseases, control primarily focuses on the rapid identification of patients to halt transmission, the elimination of mosquito larvae breeding areas by removing stagnant water or pouring abate sands into stored water and fogging to kill adult mosquitoes. The two latter vector control methods remain the primary preventative method in reducing mosquito-borne disease burdens [6].

For the optimization of vector control, it is pertinent to understand risk factors associated to disease transmission. Given the complex transmission pattern of mosquito-borne diseases, risk factors are known to be multi-factorial in nature. Meteorological parameters such as temperature, humidity and rainfall [9–11] influence the transmission of mosquito-borne diseases, as mosquito vectors can be affected by environmental changes, which can alter their survival and growth rates, thus modifying the vector’s ability to survive and breed [12]. Anthropogenic modification of the atmosphere through industrial, commercial and residential ambient air pollution is also known to add significant stress onto biological mechanisms regulating insect population sizes [13,14]. This is further compounded by increasing urbanization, greater host population sizes and continuing technological developments, which have resulted in rising levels of ambient air pollutants. Previous work has also shown strong negative correlations between PM_2.5_ and mosquito blood feeding activity levels [15]. It is assumed that PM_2.5_ particles on the antennae and abdominal body parts diminish olfactory capacity and host-seeking ability. Another study found that O_3_ decreased antennal detection of volatile organic compounds (VOCs) [16,17], which may affect blood feeding as female mosquitoes use the olfactory receptors on their maxillary palpi and antennae to find hosts [18]. Other socio-demographic and environmental risk factors related to both mosquito-borne disease burden and severity include landuse, mobility, age-structure of population, socio-economic status, access to healthcare, population density [19–22].

To date however, there has been very limited research on the impacts of both meteorological and environmental pollution exposures on multiple mosquito-borne disease burdens via long time series data. While the impact of meteorological variables on disease incidence rates has been extensively studied, the role of ambient air pollutants in shaping disease patterns remains a crucial yet relatively underexplored aspect. Recognizing that urban areas experience unique challenges related to air quality and that pollutants can have a significant impact on ecological systems, it is imperative to understand the interplay between meteorological variables and pollutants comprehensively. Therefore, we aim to begin addressing this gap by determining the risk factors driving multiple major mosquito-borne diseases in Thailand from 2003 to 2021. By doing so, we aim to provide valuable insights into the complex dynamics of disease transmission in urban settings, ultimately contributing to more effective public health interventions and strategies.

## Methods

### Mosquito-borne disease case data

Disease surveillance data was obtained from Thailand’s Ministry of Public Health disease surveillance system [23], which records reported disease case counts at the province level. Bueng Khan was split from Nong Khai in 2011 to form a separate province therefore we have merged disease case counts from Bueng Khan back to Nong Khai from 2011 onwards for consistency over the timeframe of the dataset. We considered major mosquito-borne diseases in circulation in Thailand, which include confirmed DENV, CHIKV, JEV and malaria infections between 2003 and 2021.

### Meteorological data

Climate data was obtained from ERA5, published by the European Centre for Medium-Range Weather Forecasts [24]. Each data point covers a 30km grid, which we spatially averaged across each province. Mean, median and maximum of total precipitation, vegetation index, air temperature at 2m and dew point temperature at 2m was collected. Relative humidity (RH) and average humidity (AH) were calculated using standard formula [25].

### Ambient air quality data

Ambient air pollutant data was obtained from NASA’s Goddard Earth Sciences (GES) Data and Information Services Center, GES DISC. The 1-Hourly CO Surface Concentration was obtained from GES [26], 1-Hourly Aerosol diagnostics were obtained from GES and Buchard et al. [27–30] to derive PM_2.5_ levels. The surface model layer of the 3D 3-Hourly Aerosol Mixing Ratio was similarly obtained to derive PM_2.5_ and PM_10_ levels. These datasets are a part of Modern-Era Retrospective Analysis for Research and Applications, version 2 (MERRA 2), which is the latest atmospheric reanalysis of the modern satellite era data produced by NASA’s Global Modelling and Assimilation Office (GMAO) [31].

### Demographic data

Data on annual population size for each province from 2003 to 2021 was obtained from the Official Statistics Registration Systems of Thailand [32,33]. Similarly, as Bueng Khan was split from Nong Khai in 2011 to form a separate province, we have merged the population numbers from Bueng Khan back to Nong Khai from 2011 onwards, to allow consistent analysis over the timeframe of the dataset.

### Assessing linear and non-linear associations between pollutants and dengue

Generalized linear models (GLMs) were first used to estimate linear associations between ambient air pollutants, meteorological variables and reported disease case counts. In each analysis, the dependent variable was taken as the mosquito-borne disease of interest separately. Negative binomial models were used as case counts for each disease of interest were found to be zero-inflated (refer to S1 Fig). Explanatory variables included ambient air pollutants and meteorological variables for the previous two months, as described in the data section above. We also utilised each disease’s lagged monthly case count data, up to a two-month lag, in order to account for the temporal dependence of disease case counts. This two-month lag can account for both the extrinsic and intrinsic incubation periods of the respective pathogens [34]. The log of the population was added as an offset term to account for the differences in at-risk population within each province.

To determine the presence of non-linear exposure-responses between environmental variables and disease case counts, we employed generalized additive models (GAMs). Starting from generalized linear models, each set of covariates was smoothed in succession to observe which combination of linear and smoothed terms would give us the most statistically significant improvement of fit. We separately considered models with linear meteorological terms and estimated smooth functions for each respective ambient air pollutant (Eq 1), models with smooth functions for each respective ambient air pollutant and meteorological variable (Eq 2) and models with penalized smooth functions for each respective ambient air pollutant and meteorological variable (Eq 3). Similar to GLMs, negative binomial additive models were utilized, and the log of the population was added in each model as an offset term. The proposed GAMs can be expressed as follows. Let Y be the response variable (e.g. case counts for the disease of interest), X_i_ = (X_1_,…,X_j_)’ and Z_i_ = (Z_1_,…,Z_j_) be the meteorological and ambient air pollutant covariates respectively. The GAMs assume that:

$$log(E[Y|X])=\beta_{0}+\beta_{1}Y_{t-1}+{\beta_{2}Y}_{t-2}+\sum_{n=1}^{2}\sum_{i=1}^{j}\beta_{i}X_{i,t-n}+\sum_{n=1}^{2}\sum_{i=1}^{j}f_{i}(Z_{i,t-n})+log(Population)$$ (1)

$$log(E[Y|X])=\beta_{0}+\beta_{1}Y_{t-1}+{\beta_{2}Y}_{t-2}+\sum_{n=1}^{2}\sum_{i=1}^{j}f_{i}X_{i,t-n}+\sum_{n=1}^{2}\sum_{i=1}^{j}f_{i}(Z_{i,t-n})+log(Population)$$ (2)

$$log(E[Y|X])=\beta_{0}+\beta_{1}Y_{t-1}+{\beta_{2}Y}_{t-2}+\sum_{n=1}^{2}\sum_{i=1}^{j}f_{i(penalized)}X_{i,t-n}+\sum_{n=1}^{2}\sum_{i=1}^{j}f_{i(penalized)}(Z_{i,t-n})+log(Population)$$ (3)

where β_0_ is a constant, Y_t-1_ is a one-month lag of case counts, Y_t-2_ is a two-month lag of cases counts, β_i_ is a vector of coefficients for linear terms, f_i_, i = 1,…,j are smooth functions for the meteorological and ambient air pollutant covariates. In (1) and (2), we considered the thin-plate spline as the smooth function of choice as they provide the smallest mean squared errors over alternatives [35]. As the estimation strategy of generalized cross-validation tends to under-smooth the exposure-response curves versus the true values, we utilized restricted maximum likelihood (REML) to estimate the splines in specifications (1)–(3).

As disease case counts were resolved by province and time, we separately estimated province-specific models for linear and non-linear analyses as described above, as well as the models incorporating all data longitudinally to estimate joint environmental exposure-response curves. For the latter, we considered first, a pooled GAM model with all provinces and timepoints incorporated into the same regression model with a common intercept term. We then ran fixed-effects GAM models with each province having an indicator variable as an offset. The fixed-effects models control for province specific differences in disease burden irrespective of the factors which are considered in the regression. Approximate hypothesis testing on the smoothed terms in the GAM was conducted using the Wald test in both province-specific and panel models [35].

Autocorrelation and partial autocorrelation function plots of the penultimate models used for epidemiological inference were used to assess autocorrelation. To assess model evidence, we employed the Akaike Information Criterion (AIC), which compared province-specific models and panel models separately. The AIC was used as it enables comparison between non-nested alternatives and penalizes model complexity.

### Deriving incidence rate ratios

We derived and visualized the impacts of the meteorological and ambient air pollution exposures on disease case counts by computing the Incidence Rate ratio (IRR), which provides the ratio difference in contemporaneous disease case counts given past values of the covariate of interest. Here, we first predicted incidence rates by successively varying the observed value for the environmental covariate of interest from its observed range, while keeping all other exposures constant at their mean values. The IRR estimates of the predicted disease incidence rates were then derived by taking the ratio of the predicted incidence rates at varying exposure levels (numerator) to the predicted disease incidence rates at the mean exposure level using Eq 4:

$$IRR_{i,k}=\frac{E[\hat{Y}|(X_{i}=X_{i,k},X_{j}=\bar{X_{j}})]}{E[\hat{Y}|(X_{i}=\bar{X_{i}},X_{j}=\bar{X_{j}})]},$$ (4)

where $\hat{Y}$ is the estimated disease incidence rate, *X_i_* is the exposure of interest, *X_i,k_* is the *k*^th^ quantile of the exposure *i*, and *X_j_* is the set of remaining exposures. Thus, the IRR can be expressed as a factor increase or decrease in disease case counts given a value of an exposure of interest, as compared to the disease incidence rate at the mean value of the exposure of interest, while holding all other exposures at their mean values. IRR estimates and their corresponding CIs were generated to produce the exposure response curves of the relationship between the environmental covariates and disease risk for these analyses.

## Results

### Descriptive results

Over the observational period of 2003–2021, 86 381, 292 550, 10 568, 759 220 cases were reported for CHIKV, malaria, JEV and DENV respectively, with stark heterogeneity in terms of their overall burdens as well as burdens divided by the total human population size (Table 1). Overall, the burden per 100 000 individuals is larger for malaria and DENV versus CHIKV and JEV with more substantial spread observed in the number of malaria cases (Table 1) versus the other three diseases. Ambient air temperatures (Range: 17.64–34.5°C) and relative humidity (Range: 0.46–0.92) did not vary considerably from their mean values but large variations were observed for total precipitation (Range: 0–1.24mm) across time and the region (Table 1) Similarly, large variations were observed for all ambient air pollutant measurements as well with large deviations from mean values across the study period (Table 1).

**Fig 1:**
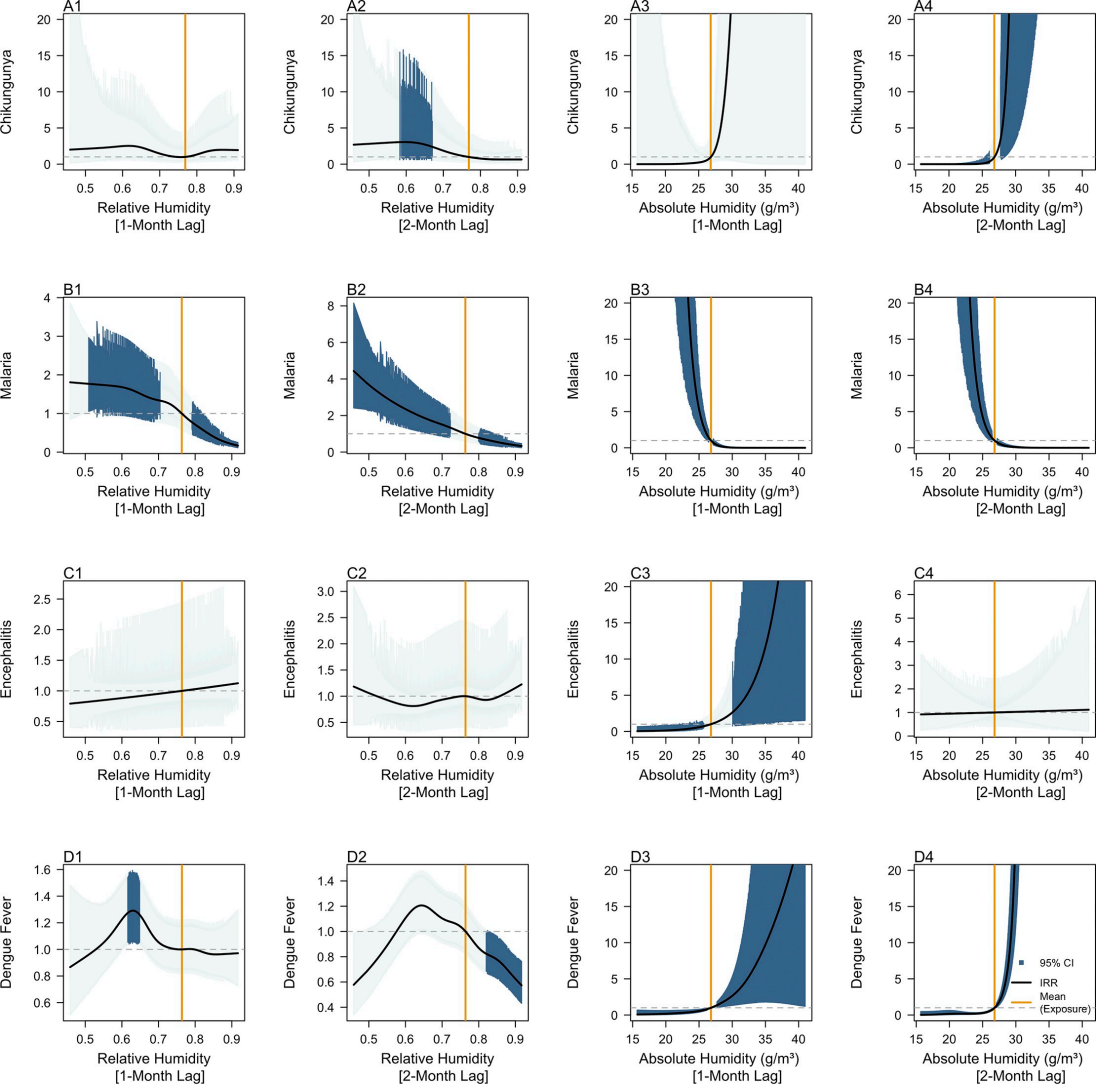
Incidence Rate Ratios of past 1-month and 2-month measurements of relative humidity (%) and absolute humidity for contemporaneous chikungunya (A1-4), malaria (B1-4), Japanese encephalitis (C1-4) and dengue fever (D1-D4) case counts per 100 000 person-months. Dark blue shaded areas represent exposure response curves with 95% confidence intervals which do not cross 1 and orange lines represent the mean recorded measurement of the respective exposure across all provinces from 2003–2021 as a reference value. The black lines represent IRR estimates, indicating the factor change in disease incidence rates across the observed range of the exposure of interest relative to the mean value of that exposure.

**Table 1:** Monthly measurements of dependent variables, and meteorological, ambient air pollutant exposures in Thailand from 2003–2021.

Variable (Monthly)	Mean	Range	SD
Chikungunya case counts per 100 000	0.96	(0, 410.23)	10.28
Malaria case counts per 100 000	3.71	(0, 492.29)	16.97
Encephalitis case counts per 100 000	0.07	(0, 2.35)	0.15
Dengue case counts per 100 000	5.12	(0, 271.61)	8.54
Absolute Humidity (g/m^3^)	26.79	(15.7, 40.98)	3.07
Relative Humidity	0.76	(0.46, 0.92)	0.08
Total Precipitation (mm)	0.18	(0, 1.24)	0.16
Temperature (°C)	26.94	(17.64, 34.5)	2.14
SO_2_ Surface Concentration (mg/m^3^)	43.06	(2.72, 241.0)	36.70
PM_2.5_ Surface Concentration (μg/m^3^)	18.21	(3.06, 110.0)	9.36
CO Surface Concentration (ppb)	152.99	(51.68, 853.72)	63.17

### Associations between past meteorological variables and major mosquito-borne diseases in Thailand

Model assessment demonstrated that the fixed-effects GAM models with unpenalized smooth functions provided the best fit to the data (see S1 Table) over other alternatives across most disease outcomes of interest. We refer to results estimated using this specification unless stated otherwise.

Across the study period, higher AH in the preceding 2 months were mainly associated with increases in CHIKV, JEV and DENV incidence rates, while higher RH values were found to be negatively associated with malaria and DENV incidence rates, when taking the mean for the observed AH and RH values as a reference. For CHIKV, RH values in the preceding 2 months (Fig 1A2) between 59% (IRR: 3.05, 95% CI: 1.04–8.98) and 68% (IRR: 2.24, 95% CI: 1.01–4.98) were estimated to be significantly positively associated with disease incidence rates. For malaria, RH values in the preceding month (Fig 1B1) between 50% (IRR:1.77, 95% CI:1.02–3.10) and 73% (IRR: 1.01–1.59), and RH values in the preceding 2 months (Fig 1B2) between 46% (IRR: 4.34, 95% CI: 2.41–8.16) and 72% (IRR: 1.03–1.77) were estimated to be positively associated with disease incidence rates. However, RH values in the preceding month (Fig 1B1) between 79% (IRR: 0.80, 95% CI: 0.64–0.99) and 92% (IRR: 0.17, 95% CI: 0.11–0.25), and RH values in the preceding 2 months (Fig 1B2) between 80% (IRR: 0.77, 95% CI: 0.62–0.96) and 92% (IRR: 0.33, 95% CI: 0.23–0.47) were estimated to be negatively associated with disease incidence rates. No significant associations were estimated between RH and JEV incidence rates, as the confidence intervals contained 1 across all observed values of RH at both 1-month and 2-month lags (Fig 1C1 and 1C2). For DENV, RH values in the preceding month (Fig 1D1) between 60% (IRR: 1.21, 95% CI: 1.01–1.48) and 66% (IRR: 1.19, 95% CI: 1.01–1.41) were predicted to be positively associated with dengue incidence rates, while RH values in the preceding 2-months (Fig 1D2) between 81% (IRR: 0.85, 95% CI: 0.72–0.99) and 92% (IRR: 0.57, 95% CI: 0.43–0.76) were predicted to be negatively associated with dengue incidence rates.

The impact of past AH on contemporaneous disease case counts was overall more pronounced. AH above 26.79g/m^3^ two months prior was estimated to be associated with increased CHIKV (Fig 1A4) and DENV (Fig 1D4) incidence rates, while AH above 26.79g/m^3^ in the preceding month were estimated to be positively associated with JEV (Fig 1C3) and DENV (Fig 1D3) incidence rates. For CHIKV, AH values in the preceding 2-months (Fig 1A4) between 15.78 g/m^3^ (IRR: 1.24 × 10−07–0.01) and 25.68g/m^3^ (IRR: 0.21, 95% CI: 0.06–0.73) were estimated to negatively associated with disease incidence rates. For JEV, AH values in the preceding month (Fig 1C3) between 15.70 g/m^3^ (IRR: 0.04, 95% CI: 1.94 × 10^−03^–0.66) and 26.02 g/m^3^ (IRR: 0.79, 95% CI: 0.63–0.99) were estimated to be negatively associated with disease incidence rates. For DENV, AH values in the preceding month (Fig 1D3) between 15.70 g/m^3^ (IRR: 0.07, 95% CI: 0.01–0.70) and 26.29 g/m^3^ (IRR: 0.84, 95% CI: 0.70–0.99), and AH values in the preceding 2-months (Fig 1D4) between 15.70 g/m^3^ (IRR: 0.02, 95% CI: 9.34 × 10^−04^–0.49) and 26.60 g/m^3^ (IRR: 0.84, 95% CI: 0.72–0.98) were found to be negatively associated with disease incidence rates. However, for malaria, AH values below 26.79 g/m^3^ were found to be positively associated with disease incidence rates. Moreover, AH values in the preceding month (Fig 1B3) between 27.06 g/m^3^ (IRR:0.77, 95% CI: 0.60–0.99) and 40.98g/m^3^ (IRR: 1.29 × 10^−08^, 95% CI: 4.07 × 10^−11^–4.09 × 10^−06^), and AH values in the preceding 2-months (Fig 1B4) between 27.14g/m^3^ (IRR: 0.77, 95% CI: 0.59–0.99) and 40.98g/m^3^ (IRR: 1.94 × 10^−05^, 95% CI: 3.29 × 10−07–1.15 × 10^−03^) were estimated to be negatively associated with disease incidence rates.

**Fig 2:**
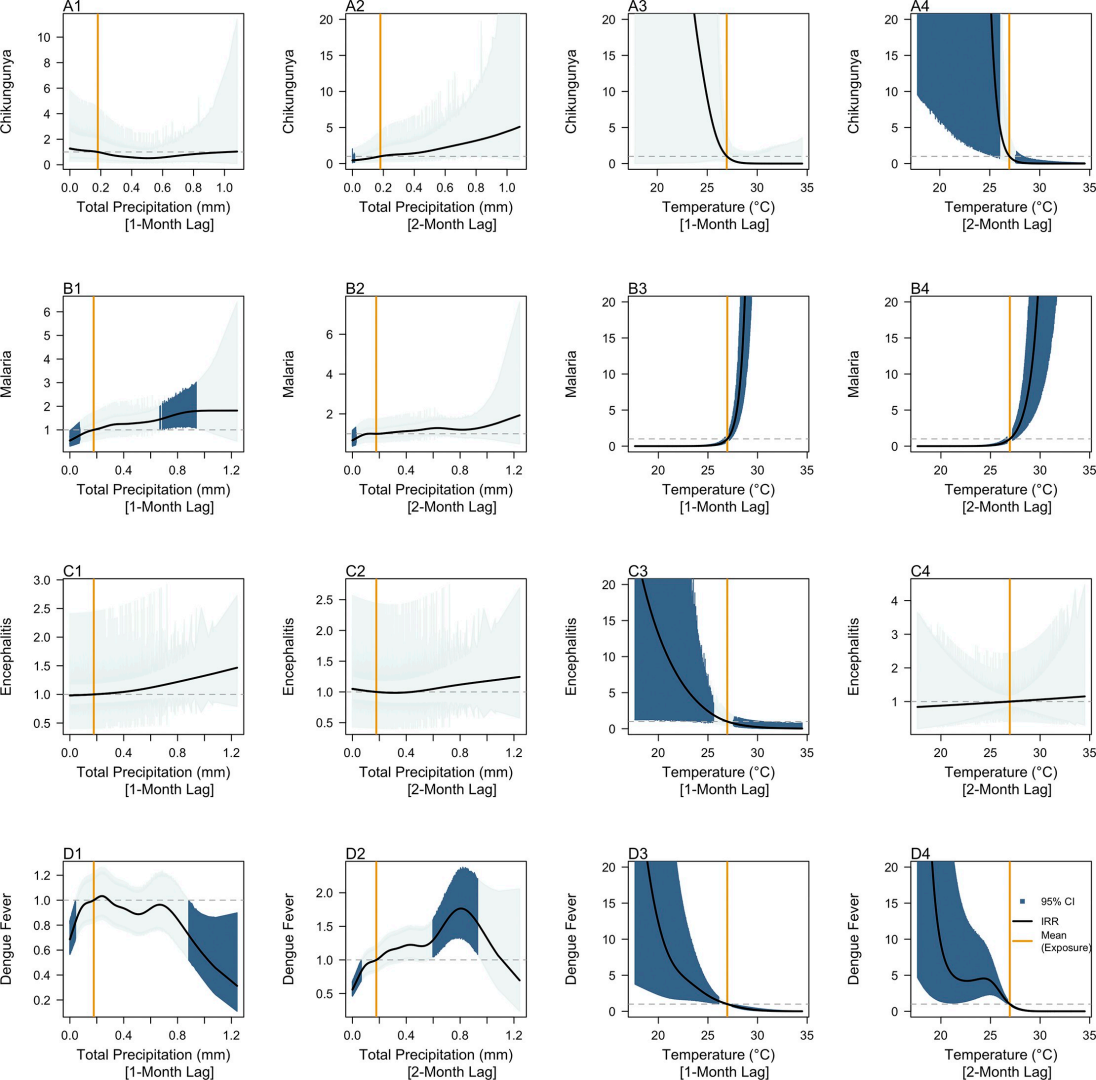
Incidence Rate Ratios of past 1-month and 2-month measurements of total precipitation (mm) and temperature (°C) on contemporaneous chikungunya (A1-4), malaria (B1-4), Japanese encephalitis (C1-4) and dengue fever (D1-4) case counts per 100 000 person-months. Dark blue shaded areas represent exposure response curves with 95% confidence intervals which do not cross 0 and orange lines represent the mean recorded measurement of the respective exposure across all provinces from 2003–2021 as a reference value. The black lines represent IRR estimates, indicating the factor change in disease incidence rates across the observed range of the exposure of interest relative to the mean value of that exposure.

In general, increased precipitation was predicted to be associated with increased disease incidence rates, while higher temperatures were predicted to be associated with decreased disease incidence rates. For CHIKV, total precipitation values in the preceding 2-months (Fig 2A2) between 0.00mm (IRR: 0.44, 95% CI: 0.19–0.99) and 0.02mm (IRR: 0.48, 95% CI: 0.23–0.99) were found to be negatively associated with disease incidence rates. For malaria, total precipitation values in the preceding month (Fig 2B1) between 0.00mm (IRR: 0.55, 95% CI: 0.38–0.80) and 0.08mm (IRR: 0.80, 95% CI: 0.65–0.99) were negatively associated with disease incidence rates, while values between 0.63mm (IRR: 1.40, 95% CI: 1.03–1.88) and 0.98mm (IRR:1.81, 95% CI: 1.02–3.21) were estimated to be positively associated with disease incidence rates. When total precipitation values in the preceding 2-months (Fig 2B2) ranged between 0.00mm (IRR: 0.67, 95% CI: 0.46–0.98) and 0.03mm (IRR: 0.77, 95% CI: 0.61–0.97), the association between total precipitation and malaria incidence rates was estimated to be negative. No significant associations were predicted between total precipitation and JEV incidence rates throughout the entire observed range of total precipitation values in this study (Fig 2C1 and 2C2). For DENV, total precipitation values in the preceding month (Fig 2D1) between 0.00mm (IRR: 0.67, 95% CI: 0.57–0.82) and 0.05mm (IRR: 0.85, 95% CI: 0.73–0.99), and values between 0.88mm (IRR: 0.73, 95% CI: 0.53–0.99) and 1.24mm (IRR: 0.31, 95% CI: 0.11–0.90) were estimated to be negatively associated with dengue incidence rates. Total precipitation values in the preceding 2-months (Fig 2D2) between 0.00mm (IRR: 0.56, 95% CI: 0.47–0.67) and 0.07mm (IRR: 0.84, 95% CI: 0.71–0.99) were estimated to be negatively associated with disease incidence rates, while values between 0.59mm (IRR: 1.29, 95% CI: 1.05–1.57) and 0.94mm (IRR: 1.52, 95% CI: 1.05–2.19) were estimated to be positively associated with dengue incidence rates.

Temperatures between 17.64°C and 26.75°C (Fig 2A4) 2-months prior were positively associated with CHIKV incidence rates, while temperatures between 27.11°C (IRR: 0.82, 95% CI: 0.69–0.98) and 34.50°C (IRR: 2.75e—09, 95% CI: 1.23 × 10^−11^–6.10 × 10^−07^) were estimated to be negatively associated with CHIKV incidence rates. Similarly, JEV incidence rates were estimated to be positively associated with temperatures 1-month prior (Fig 2C3) between 17.64°C (IRR: 25.4, 95% CI: 1.23–5.30 × 10^02^) and 26.00°C (IRR:1.56, 95% CI: 1.01–2.42). However, temperatures between 27.42°C (IRR: 0.80, 95% CI: 0.64–0.99) and 34.50°C (IRR: 0.03, 95% CI: 8.98 × 10^−04^–0.81) were estimated to be negatively associated with JEV incidence rates. Similarly, for DENV, temperatures 1-month prior (Fig 2D3) between 17.64°C (IRR: 37.04, 95% CI: 3.77–364.10) and 26.26°C (IRR: 1.33, 95% CI: 1.02–1.74) were estimated to be positively associated with disease incidence rates, while temperatures between 27.41°C (IRR: 0.81, 95% CI: 0.67–0.98) and 34.50°C (IRR: 2.78 × 10^−03^, 95% CI: 1.51 × 10^−04^–0.05) were estimated to be negatively associated with disease incidence rates. Temperatures 2-months prior (Fig 2D4) between 17.64°C (IRR:1.07 × 10^02^, 95% CI: 4.68–2.45 × 10^03^) and 26.77°C (IRR: 1.22, 95% CI: 1.02–1.47) were predicted to be positively associated with disease incidence rates, while temperatures 2-months prior between 27.09°C (IRR: 0.84, 95% CI: 0.72–0.99) and 34.50°C (IRR: 2.75 × 10^−09^, 95% CI: 1.24 ×10^−11^–6.10 × 10^−07^) were predicted to be negatively associated with disease incidence rates. In contrast, for malaria, temperatures in the preceding month (Fig 2B3) between 17.64°C (IRR: 5.66 × 10^−05^, 95% CI: 1.23 × 10^−06^–2.61 × 10^−03^) and 26.73°C (IRR: 0.71, 95% CI: 0.53–0.95) were estimated to negatively associated with disease incidence rates, while temperatures between 27.12°C (IRR: 1.31, 95% CI: 1.04–1.66) and 30.38°C (IRR: 7.03 × 10^02^, 95% CI: 1.13 × 1002–4.37 × 10^03^) were estimated to be positively associated with disease incidence rates. At a 2-month lag, temperatures (Fig 2B4) between 17.64°C (IRR: 1.90 × 10^−04^, 95% CI: 7.77 × 10^−06^–4.66 × 10^−03^) and 26.72°C (IRR:0.76, 95% CI: 0.59–0.99) were estimated to be negatively associated with disease incidence rates, while temperatures between 27.18°C (IRR: 1.30, 95% CI: 1.03–1.65) and 30.05°C (IRR: 28.35, 95% CI: 6.66–1.21 × 10^02^) were estimated to be positively associated with disease incidence rates.

### Associations between past ambient air pollutant variables and major mosquito-borne diseases in Thailand

**Fig 3:**
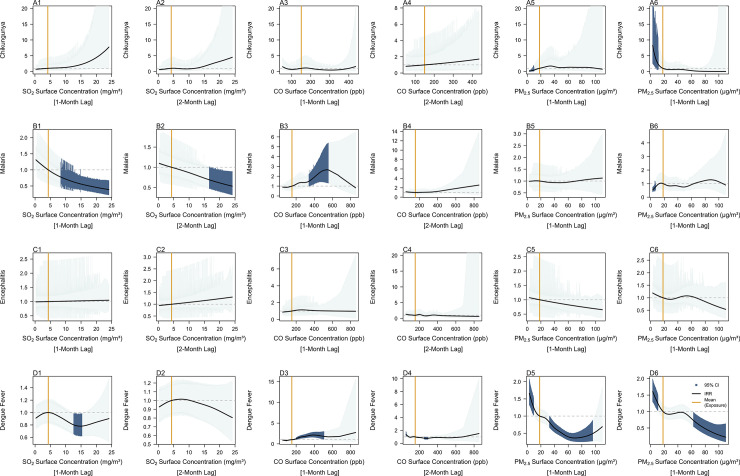
Incidence Rate Ratios of past 2 months measurements of SO_2_ surface concentration (mg/m^3^), CO surface concentration (ppb) and PM_2.5_ surface concentration (μg/m^3^) on contemporaneous chikungunya (A1-6), malaria (B1-6), Japanese encephalitis (C1-6) and dengue fever (D1-6) case counts per 100 000 person-months. Dark blue shaded areas represent exposure response curves with 95% confidence intervals which do not cross 1 and orange lines represent the mean recorded measurement of the respective exposure across all provinces from 2003–2021 as a reference value. The black lines represent IRR estimates, indicating the factor change in disease incidence rates across the observed range of the exposure of interest relative to the mean value of that exposure.

Past SO_2_ surface concentrations above 10mg/m^3^ were negatively associated with contemporaneous malaria (Fig 3B1 and 3B2) and DENV incidence rates (Fig 3D1). SO_2_ surface concentrations in the preceding month between (Fig 3B1) 9.15mg/m^3^ (IRR: 0.75, 95% CI: 0.59–0.95) and 24.1mg/m^3^ (IRR: 0.39, 95% CI: 0.22–0.67), and SO_2_ surface concentrations in the preceding 2 months between 15.3mg/m^3^ (IRR: 0.72, 95% CI: 0.53–0.99) and 24.1mg/m^3^ (IRR: 0.53, 95% CI: 0.32–0.90) were both estimated to be negatively associated with malaria incidence rates. Similarly, for DENV, SO_2_ surface concentrations in the preceding month between 11.6mg/m^3^ (IRR: 0.83, 95% CI: 0.69–0.99) and 16.9mg/m^3^ (IRR: 0.80, 95% CI: 0.63–0.99) were estimated to be negatively associated with dengue incidence rates. No significant associations were estimated between past SO_2_ surface concentrations and CHIKV or JEV incidence rates.

Increased past CO surface concentrations were predicted to be positively associated with malaria and DENV incidence rates, while associations between past CO surface concentrations and CHIKV and JEV incidence rates were predicted to be insignificant. For malaria, past CO surface concentrations in the preceding month (Fig 3B3) between 327ppb (IRR: 1.43, 95% CI: 1.03–2.00) and 554ppb (IRR: 2.65, 95% CI: 1.31–5.38) were estimated to be positively associated with incidence rates. For DENV, past CO surface concentrations in the preceding month (Fig 1D3) between 77ppb (IRR:0.83, 95% CI: 0.69–0.99) and 96ppb (IRR: 0.83, 95% CI: 0.69–0.98) were estimated to be negatively associated with disease incidence rates, while values between 198ppb (IRR:1.22, 95% CI: 1.03–1.44) and 506ppb (IRR: 1.81, 95% CI: 1.10–2.98) were estimated to be positively associated with disease incidence rates. CO surface concentrations 2-months prior (Fig 3D4) between 52ppb (IRR: 1.40, 95% CI: 1.08–1.81) and 61ppb (IRR: 1.26, 95% CI: 1.01–1.58) were positively associated with dengue incidence rates, while values between 247ppb (IRR: 0.81, 95% CI: 0.67–0.99) and 304ppb (IRR: 0.80, 95% CI: 0.65–0.99) were negatively associated with dengue incidence rates.

In general, an inverse relationship was estimated between past PM_2.5_ surface concentrations, CHIKV and DENV incidence rates. For CHIKV, past PM_2.5_ surface concentrations in the 2-months prior (Fig 3A6) between 3.06 μg/m^3^ (IRR: 8.27, 95% CI: 2.50–27.29) and 10.7μg/m^3^ (IRR:2.51, 95% CI: 1.08–5.84) were predicted to be positively associated with disease incidence rates. For DENV, at a 1-month lag, past PM_2.5_ surface concentrations between 3.06μg/m^3^ (IRR: 1.66, 95% CI:1.32–2.08) and 10.6μg/m^3^ (IRR: 1.20, 95% CI: 1.01–1.41) were positively associated with disease incidence rates, while PM_2.5_ surface concentrations between 31.7μg/m^3^ (IRR: 0.84, 95% CI: 0.71–0.99) and 98.7μg/m^3^ (IRR: 0.52, 0.28–0.99) were estimated to be negatively associated with disease incidence rates. Similarly, at a 2-month lag, past PM_2.5_ surface concentrations between 3.06μg/m^3^ (IRR: 1.61, 95% CI: 1.30–1.99) and 11.9μg/m^3^ (IRR: 1.20, 95% CI: 1.01–1.42) were estimated to be positively associated with disease incidence rates, while surface concentrations between 61.4μg/m^3^ (IRR:0.76, 95% CI: 0.57–0.99) and 110μg/m^3^ (IRR: 0.22, 95% CI: 0.08–0.63) were estimated to be negatively associated with disease incidence rates.

### Comparison to province-specific models

While the pooled generalized additive model allowed us to exploit the panel data structure to estimate exposure-response curves, unobserved spatial confounding may hamper the validity of previously estimated associations. Therefore, we ran province-specific models with relatively smaller sample sizes (*N*≈160) to examine whether the province-specific exposure-responses reflected those of the pooled model. Model assessment was also conducted here to validate whether the ideal linear or non-linear model for panel data is reflected in the province-specific model (See S1 Supplementary Information). Province specific estimates of IRR followed closely to that of the pooled model. In particular, past AH values above the mean measurements were mainly associated to decreased contemporaneous disease case counts (Table 2) but the relationships of RH and temperature were more mixed (Table 2). The complex associations between ambient air pollutants and disease case counts in the pooled model (Fig 3) were reflected in the mixed associations between ambient air pollutants and disease case counts at the province-specific level (Table 2) Past total precipitation above mean measurements was also mainly associated to increases in contemporaneous disease case counts in the province specific models.

**Table 2:** Percentage of provinces with explored meteorological and ambient air pollutant variables estimated to have Incidence Rate Ratios below or above 1 when values were above their respective mean measurements at 1-month and 2-month lags. IRRs greater than 1 indicate a positive associations, while IRRs less than 1 indicate negative associations.

Variable	CHIKV	Malaria	JEV	DENV	Variable	CHIKV	Malaria	JEV	DENV
Absolute Humidity(1-Month Lag)					CO (1-Month Lag)				
% IRR < 1	0.00	86.84	75.00	15.79	% IRR < 1	0.00	6.58	0.00	64.47
% IRR = 1	100.00	11.63	10.53	3.95	% IRR = 1	98.68	35.53	100.00	6.58
% IRR > 1	0.00	10.53	14.47	80.26	% IRR > 1	1.32	57.89	0.00	28.95
Absolute Humidity (2-Month Lag)					CO (2-Month Lag)				
% IRR < 1	17.11	72.37	0.00	21.05	% IRR < 1	0.00	0.00	5.27	22.37
% IRR = 1	7.89	5.26	100.00	1.32	% IRR = 1	100.00	10.00	86.84	56.58
% IRR > 1	75.00	22.37	0.00	77.63	% IRR > 1	0.00	0.00	7.89	21.05
Relative Humidity (1-Month Lag)					PM_2.5_ (1-Month Lag)				
% IRR < 1	0	98.68	0.00	0.00	% IRR < 1	84.21	0.00	0.00	3.95
% IRR = 1	93.42	1.32	100.00	18.42	% IRR = 1	15.79	10.00	100.00	0.00
% IRR > 1	6.58	0.00	0.00	81.58	% IRR > 1	0.00	0.00	0.00	96.05
Relative Humidity (2-Month Lag)					PM_2.5_ (2-Month Lag)				
% IRR < 1	0.00	94.74	0.00	76.32	% IRR < 1	0.00	0.00	0.00	0.00
% IRR = 1	59.21	0.00	100.00	0.00	% IRR = 1	6.58	56.58	100.00	0.00
% IRR > 1	40.79	5.26	0.000	23.68	% IRR > 1	93.42	43.42	0.00	100.00
Temperature (1-Month Lag)					SO_2_ (1-Month Lag)				
% IRR < 1	0.00	2.63	80.26	100.00	% IRR < 1	0.00	43.42	0.00	6.58
% IRR = 1	100.00	0.00	7.90	0.00	% IRR = 1	100.00	36.84	100.00	90.79
% IRR > 1	0.00	97.37	11.84	0.00	% IRR > 1	0.00	19.74	0.00	2.63
Temperature (2-Month Lag)					SO_2_ (2-Month Lag)				
% IRR < 1	96.05	7.89	0.00	90.79	% IRR < 1	0.00	9.21	0.00	0.00
% IRR = 1	3.95	1.32	100.00	1.32	% IRR = 1	92.11	90.79	100.00	100.00
% IRR > 1	0.00	90.79	0.00	7.89	% IRR > 1	7.89	0.00	0.00	0.00
Total Precipitation (1-Month Lag)					Total Precipitation (2-Month Lag)				
% IRR < 1	0.00	97.37	0.00	100.00	% IRR < 1	31.59	0.00	0.00	100.00
% IRR = 1	100.00	2.63	100.00	0.00	% IRR = 1	57.89	5.26	100.00	0.00
% IRR > 1	0.00	0.00	0.00	0.00	% IRR > 1	10.52	94.74	0.00	0.00

## Discussion

Our study set out to determine the drivers over two decades of CHIKV, DENV, malaria and JEV cases in Thailand. The prevalence of these four major mosquito-borne diseases were substantially different across Thailand (Table 1). Both lagged meteorological and ambient air pollutants were found to be significantly associated to contemporaneous disease case counts across all diseases.

Our analyses indicated several findings which can be generally applied to all diseases explored: **(1)** higher AH above mean values was positively associated with disease case counts **(2)** higher total precipitation above mean values was positively associated with disease case counts **(3)** extremely high temperatures were negatively associated with disease case counts **(4)** higher SO_2_ and PM_2.5_ surface concentrations were negatively associated with disease case counts. However, the relationships between disease and RH, non-extreme temperatures and CO surface concentration were more mixed, with directions of associations changing across the different diseases considered.

Consistent with previous findings, AH and precipitation above mean values were found to be positively associated with CHIKV [36], JEV [37] and DENV [38] incidence rates. Higher humidity results in increased adult abundance and extended survival beyond the post-extrinsic incubation period (EIP) of the *Aedes aegypti* vector [39], which is responsible for both CHIKV and DENV transmission. While increased AH was positively associated with disease incidence rates, our study found that RH above 79% (Fig 1B1 and 1B2) and 81% (Fig 1D2) was negatively associated with malaria and dengue incidence rates respectively. These findings corroborate with the work of da Cruz Ferreira et al. (2017), where RH had an approximately linear negative effect on the *Aedes aegypti* vector population when RH was above 79% [40]. A possible explanation is that high RH saturates the air with water vapour, potentially reducing mosquito host-seeking activity by diluting the chemical attractants released by hosts. Precipitation provides more vector breeding habitats and potential for vector populations to increase. However, excessive precipitation may also disrupt the vector reproductive cycle by flushing out aquatic stages from breeding sites [41], leading to a lower risk of dengue outbreaks in subsequent months, which aligns strongly with the negative associations found between dengue incidence rates and precipitation (Fig 2D1) in our study.

While there is also compelling evidence supporting the hypotheses that mosquito oviposition, development from mosquito larva to adult, biting rate and virus replication rate in mosquitoes are strongly enhanced at raised ambient temperatures, we found that temperatures above 27°C were negatively associated with CHIKV, JEV and DENV incidence rates (Fig 2). This effect may be linked to the temperature-sensitive duration of EIP in the vector, which is critical for transmission [42], and the negative effects of high temperatures on adult survival, larval development, and vector competence [43–45]. These findings are consistent with the concept of an optimal temperature window which allows for maximal disease transmission, while temperatures outside this window may inhibit disease transmission [46]. However, our findings suggest that CHIKV, JEV and DENV transmission is optimal at lower temperatures (17°C—25°C), which deviate from the optimal temperature window proposed by Mordecai et al. (2017). Nevertheless, Mordecai’s work ascertains that disease transmission of CHIKV and DENV can occur between 18°C and 34°C, especially in tropical as well as sub-tropical regions, and the differences in the findings can be potentially attributed to the incorporation of lagged-temperature effects in our study. Malaria was also estimated to have contrasting associations with temperature as compared to CHIKV, JEV and DENV. Malaria incidence rates were estimated to negatively associated with temperatures between 17°C and 26.7°C, and positively associated with temperatures above 27°C instead. A possible explanation is that the *Anopheles* mosquito, which is responsible for malaria transmission, is less sensitive to higher temperatures as compared to the *Aedes* and *Culex* species that are responsible for CHIKV, DENV and JEV transmission, resulting in differing optimal temperature windows across the vectors.

Aside from meteorological variables, our study found that past increases in ambient air pollutants surface concentrations were associated with negative disease incidence rates, which is consistent with previous work [47]. SO_2_ surface concentrations above approximately 10mg/m^3^ were negatively associated to contemporaneous malaria and DENV case counts. One possibility is that SO_2_ is known to cause acid deposition, potentially causing higher mortality for egg, larval and pupal states. Additionally, SO_2_ is known to be produced from the combustion of sulphur-bearing fossil fuels [48] and may be a proxy for urbanicity, which is a known driver of dengue transmission. Similarly, associations between PM_2.5_ and DENV incidence rates were also negative. These findings are validated by the work of Phanitchat et al. (2021), where strong negative correlations were found PM_2.5_ concentrations and *Aedes aegypti* blood feeding activity levels, which was attributed to the stress induced by PM_2.5_ on the vector’s olfactory system. In general, increases in CO in the preceding month above 198ppb and 327ppb were associated with increased DENV and malaria incidence rates respectively. Further work is required to understand the underlying atmospheric and biological mechanisms behind how PM_2.5_, SO_2_, and CO affect mosquitoes and disease transmission.

Our study has several key advantages. We utilized a large, routinely collected set of longitudinal surveillance data in a region where mosquito-borne diseases were endemic. This spanned across a large geographical scale which allowed us to exploit spatio-temporal variations and delineate the associations between meteorological and ambient air pollutant risk factors for disease transmission. By harmonizing analyses and datasets for multiple major mosquito-borne diseases, we enabled cross-comparisons between factors which increase or reduce the risk of mosquito-borne disease transmission. Furthermore, our analysis explored both location-specific and panel models which incorporated non-linearity, departing from restrictive linear models commonly used in epidemiological analysis and enabling us to estimate ecological exposure-response curves. This allows us to understand specific ranges at which exposures were associated to increases or decreases in disease incidence rates, as well as potential non-linearities between exposures and disease incidence rates (Figs 1–3).

While our research has yielded valuable insights, it is essential to acknowledge and address several limitations that should be considered when interpreting the results. The under-reporting of disease cases could potentially occur across time which could lead to interactive effects not being fully captured. We took the province-level average of air pollutants within this study whereas the occurrence of acute or prolonged point pollutant events could cause differing effects spatially across each province. Other spatial biases include heterogeneous vegetation levels interacting with air quality impacting mosquito survival and breeding behaviour, or temperature differences between highly built up and relatively rural areas due to urban heat island effects. Moreover, meteorological variables such as humidity and temperature are typically correlated, and the attribution of changes in disease incidence rates can be restricted by this collinearity. Future work should therefore account for spatial heterogeneity as well as collinearity in ambient air pollutants, environmental confounders and disease transmission. We were further constrained by the resolution of our data. Although we utilized the finest spatial and temporal scale available, disease case counts were only available on a monthly basis, preventing us from accounting for effects on a weekly time-scale. Furthermore, while we included the majority of common ambient air pollutants, it is possible that the secondary products of these or minor pollutants which were not included could introduce new interactive effects or drive disease counts. Other major ambient air pollutants which were not considered were NO_2_ and O_3_ due to limited data availability. Lastly, spatial confounders were aggregated as fixed effect terms in the panel analyses but should ideally be incorporated as separate terms in the regression specification to delineate their contribution on disease transmission. However, there was insufficient data on the same time scale as disease surveillance data to allow imputation for downstream analysis.

## Supporting information

S1 Table Akaike Information Criterion for models incorporating all longitudinal measurements in Thailand for chikungunya, malaria, Japanese encephalitis and dengue fever. Bold values represent the best fitting model in terms of AIC, which balances model fit and parsimony.
(PDF)

S1 Fig Kernel density estimates of Chikungunya (A1), Malaria (A2), Japanese Encephalitis (B1), and Dengue Fever (B2) case counts. Concentrated densities near zero indicate that case counts are zero-inflated.
(PDF)

S1 Supplementary Information Incidence Rate Ratio plots of province-specific models for each province considered in this study. Dark blue shaded areas represent exposure response curves with 95% confidence intervals which do not cross 1 and orange lines represent the mean recorded measurement of the respective exposure across for the respective province from 2003–2021 as a reference value. The black lines represent IRR estimates, indicating the factor change in disease incidence rates across the observed range of the exposure of interest relative to the mean value of that exposure.
(PDF)
