# Supplementary material for: Non-linear associations between meteorological factors, ambient air pollutants and major mosquito-borne diseases in Thailand
Source: PLoS Negl Trop Dis. 2023 Dec 27;17(12):e0011763. doi: 10.1371/journal.pntd.0011763 (PMC10752508; doi:10.1371/journal.pntd.0011763)
Supplement: S1 Table — Bold values represent the best fitting model in terms of AIC, which balances model fit and parsimony. (PDF) [file pntd.0011763.s001.pdf]

Model assessment

	Akaike Information Criterion	
	Pooled	Fixed Effects
	Chikungunya	
Linear	23057.06	21515.14
GAM Pollutants	22596.67	21454.44
GAM All	22449.46	**21405.47**
GAM All Penalized	22484.84	21427.97
	Malaria	
Linear	812374.44*	78570.06
GAM Pollutants	87029.89	78447.91
GAM All	86723.64	78006.17
GAM All Penalized	86719.96	**78004.80**
	Japanese Encephalitis	
Linear	30587.51	28858.24
GAM Pollutants	30500.03	28826.64
GAM All	30484.07	**28809.94**
GAM All Penalized	30482.19	28813.33
	Dengue Fever	
Linear	151272.97	148250.24
GAM Pollutants	150651.18	147925.62
GAM All	150303.13	**147229.62**
GAM All Penalized	150310.67	147237.66

**Regular poisson model was used in this instance as the negative binomial algorithm fails to converge, resulting in an exceptionally high AIC value*
