# Supplementary figures and images for: Non-linear associations between meteorological factors, ambient air pollutants and major mosquito-borne diseases in Thailand
Source: PLoS Negl Trop Dis. 2023 Dec 27;17(12):e0011763. doi: 10.1371/journal.pntd.0011763 (PMC10752508; doi:10.1371/journal.pntd.0011763)

# Distribution of Disease Case Counts

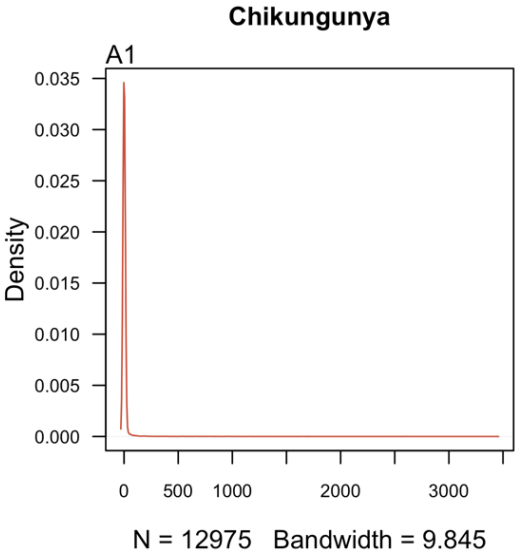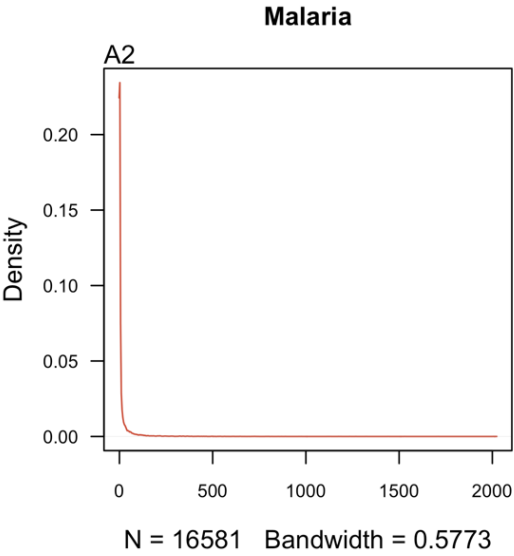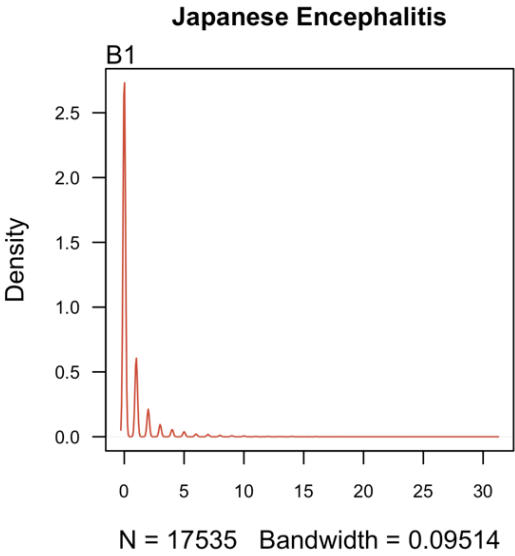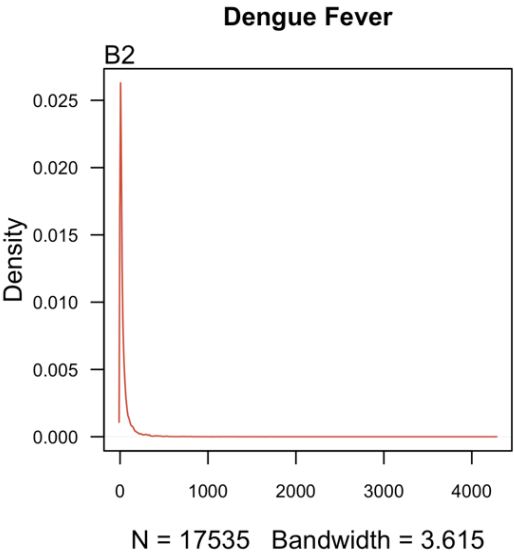

Supplement: S1 Fig — Concentrated densities near zero indicate that case counts are zero-inflated. (PDF) [file pntd.0011763.s002.pdf]
