# Supplementary material for: Non-linear associations between meteorological factors, ambient air pollutants and major mosquito-borne diseases in Thailand
Source: PLoS Negl Trop Dis. 2023 Dec 27;17(12):e0011763. doi: 10.1371/journal.pntd.0011763 (PMC10752508; doi:10.1371/journal.pntd.0011763)
Supplement: S1 Supplementary Information — Dark blue shaded areas represent exposure response curves with 95% confidence intervals which do not cross 1 and orange lines represent the mean recorded measurement of the respective exposure across for the respective province from 2003–2021 as a reference value. The black lines represent IRR estimates, indicating the factor change in disease incidence rates across the observed range of the exposure of interest relative to the mean value of that exposure. (PDF) [file pntd.0011763.s003.pdf]

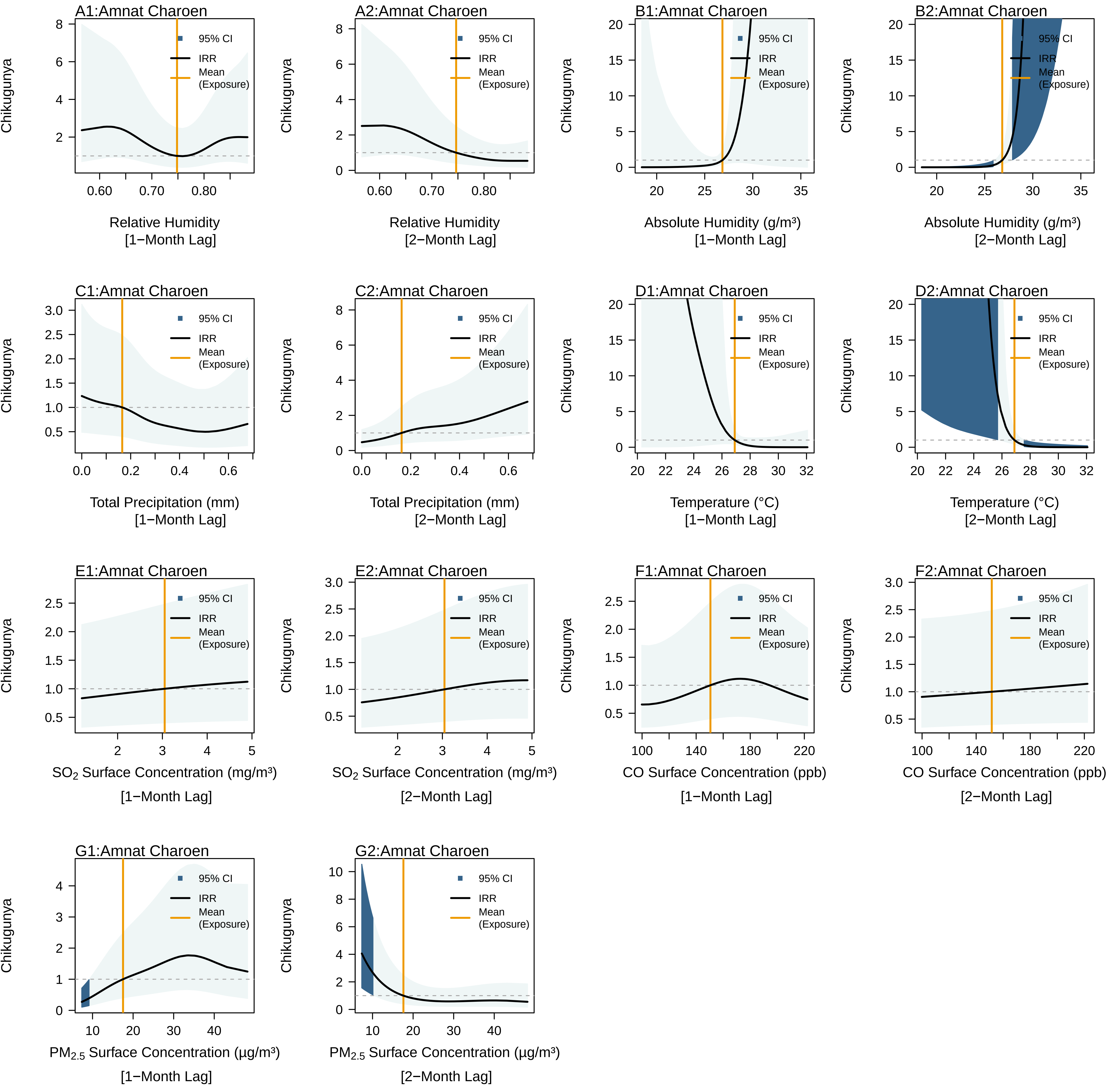
A1:Amnat Charoen
Chikugunya
95% CI
IRR
Mean (Exposure)
Relative Humidity [1−Month Lag]
A2:Amnat Charoen
Chikugunya
95% CI
IRR
Mean (Exposure)
Relative Humidity [2−Month Lag]
B1:Amnat Charoen
Chikugunya
95% CI
IRR
Mean (Exposure)
Absolute Humidity (g/m³) [1−Month Lag]
B2:Amnat Charoen
Chikugunya
95% CI
IRR
Mean (Exposure)
Absolute Humidity (g/m³) [2−Month Lag]
C1:Amnat Charoen
Chikugunya
95% CI
IRR
Mean (Exposure)
Total Precipitation (mm) [1−Month Lag]
C2:Amnat Charoen
Chikugunya
95% CI
IRR
Mean (Exposure)
Total Precipitation (mm) [2−Month Lag]
D1:Amnat Charoen
Chikugunya
95% CI
IRR
Mean (Exposure)
Temperature (°C) [1−Month Lag]
D2:Amnat Charoen
Chikugunya
95% CI
IRR
Mean (Exposure)
Temperature (°C) [2−Month Lag]
E1:Amnat Charoen
Chikugunya
95% CI
IRR
Mean (Exposure)
SO2 Surface Concentration (mg/m³) [1−Month Lag]
E2:Amnat Charoen
Chikugunya
95% CI
IRR
Mean (Exposure)
SO2 Surface Concentration (mg/m³) [2−Month Lag]
F1:Amnat Charoen
Chikugunya
95% CI
IRR
Mean (Exposure)
CO Surface Concentration (ppb) [1−Month Lag]
F2:Amnat Charoen
Chikugunya
95% CI
IRR
Mean (Exposure)
CO Surface Concentration (ppb) [2−Month Lag]
G1:Amnat Charoen
Chikugunya
95% CI
IRR
Mean (Exposure)
PM2.5 Surface Concentration (μg/m³) [1−Month Lag]
G2:Amnat Charoen
Chikugunya
95% CI
IRR
Mean (Exposure)
PM2.5 Surface Concentration (μg/m³) [2−Month Lag]

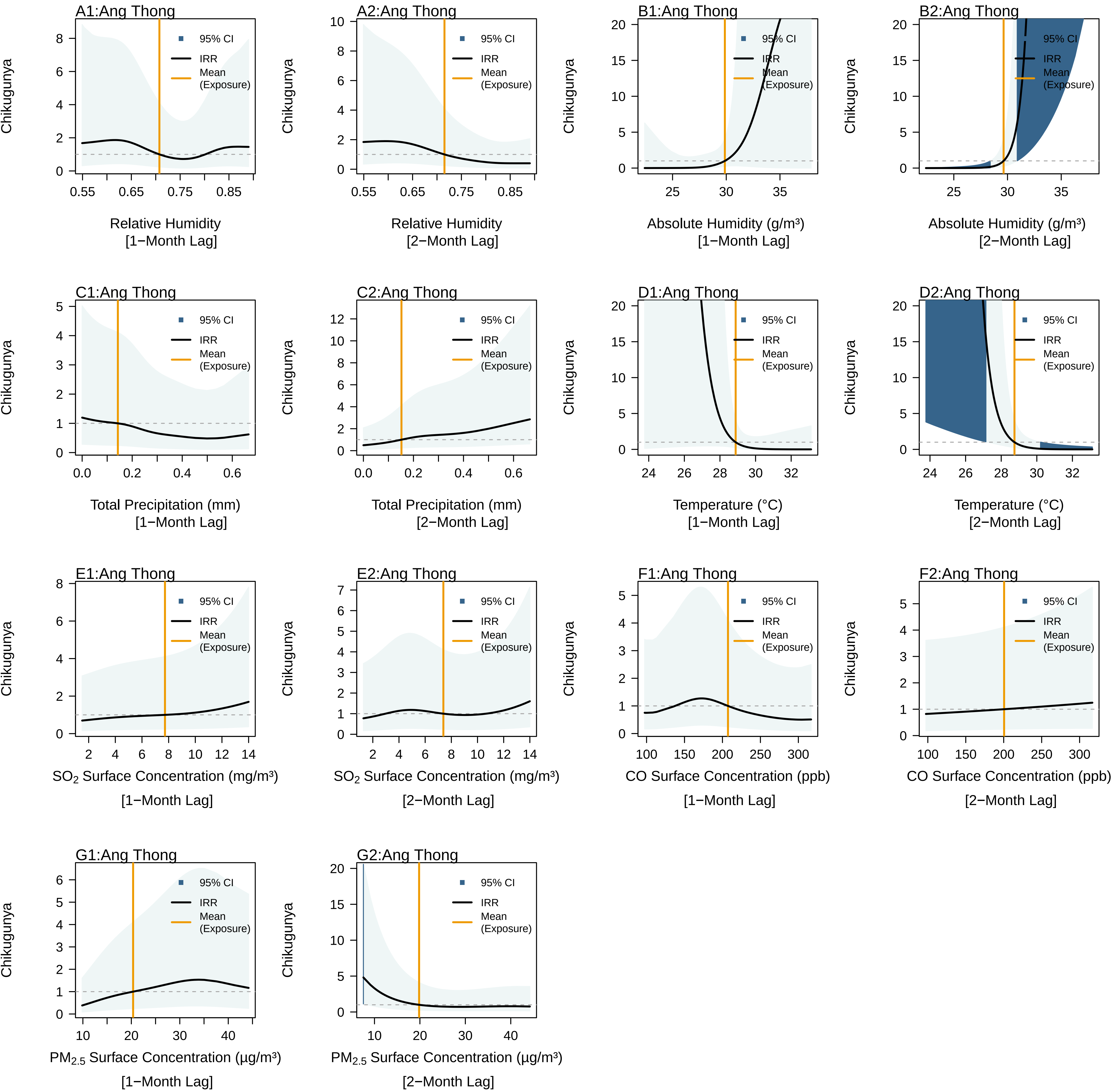
A1:Ang Thong
Chikugunya
95% CI
IRR
Mean (Exposure)
Relative Humidity [1−Month Lag]
A2:Ang Thong
Chikugunya
95% CI
IRR
Mean (Exposure)
Relative Humidity [2−Month Lag]
B1:Ang Thong
Chikugunya
95% CI
IRR
Mean (Exposure)
Absolute Humidity (g/m³) [1−Month Lag]
B2:Ang Thong
Chikugunya
95% CI
IRR
Mean (Exposure)
Absolute Humidity (g/m³) [2−Month Lag]
C1:Ang Thong
Chikugunya
95% CI
IRR
Mean (Exposure)
Total Precipitation (mm) [1−Month Lag]
C2:Ang Thong
Chikugunya
95% CI
IRR
Mean (Exposure)
Total Precipitation (mm) [2−Month Lag]
D1:Ang Thong
Chikugunya
95% CI
IRR
Mean (Exposure)
Temperature (°C) [1−Month Lag]
D2:Ang Thong
Chikugunya
95% CI
IRR
Mean (Exposure)
Temperature (°C) [2−Month Lag]
E1:Ang Thong
Chikugunya
95% CI
IRR
Mean (Exposure)
SO2 Surface Concentration (mg/m³) [1−Month Lag]
E2:Ang Thong
Chikugunya
95% CI
IRR
Mean (Exposure)
SO2 Surface Concentration (mg/m³) [2−Month Lag]
F1:Ang Thong
Chikugunya
95% CI
IRR
Mean (Exposure)
CO Surface Concentration (ppb) [1−Month Lag]
F2:Ang Thong
Chikugunya
95% CI
IRR
Mean (Exposure)
CO Surface Concentration (ppb) [2−Month Lag]
G1:Ang Thong
Chikugunya
95% CI
IRR
Mean (Exposure)
PM2.5 Surface Concentration (µg/m³) [1−Month Lag]
G2:Ang Thong
Chikugunya
95% CI
IRR
Mean (Exposure)
PM2.5 Surface Concentration (µg/m³) [2−Month Lag]

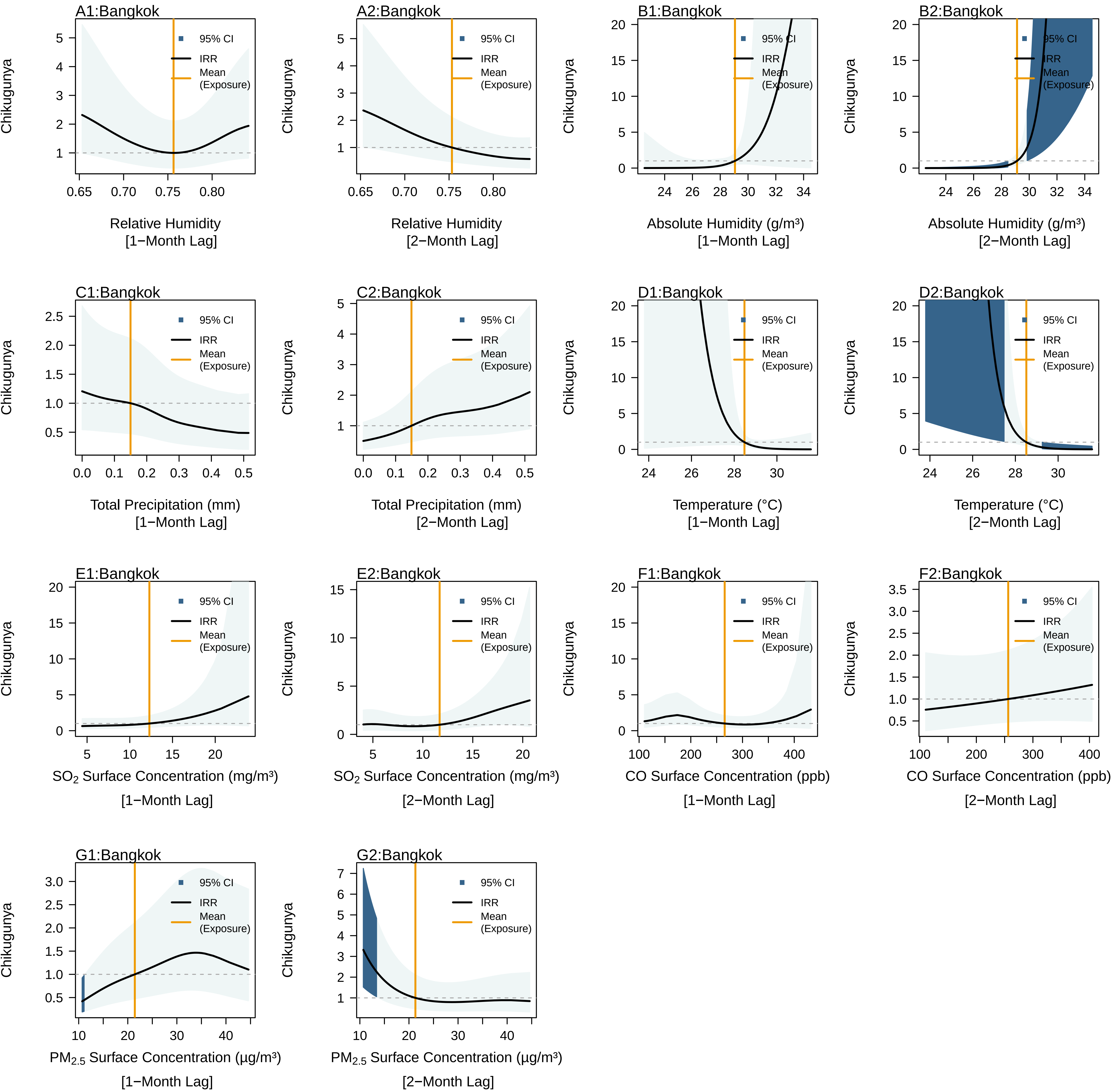
A1:Bangkok
Chikugunya
95% CI
IRR
Mean
(Exposure)
Relative Humidity
[1−Month Lag]
A2:Bangkok
Chikugunya
95% CI
IRR
Mean
(Exposure)
Relative Humidity
[2−Month Lag]
B1:Bangkok
Chikugunya
95% CI
IRR
Mean
(Exposure)
Absolute Humidity (g/m³)
[1−Month Lag]
B2:Bangkok
Chikugunya
95% CI
IRR
Mean
(Exposure)
Absolute Humidity (g/m³)
[2−Month Lag]
C1:Bangkok
Chikugunya
95% CI
IRR
Mean
(Exposure)
Total Precipitation (mm)
[1−Month Lag]
C2:Bangkok
Chikugunya
95% CI
IRR
Mean
(Exposure)
Total Precipitation (mm)
[2−Month Lag]
D1:Bangkok
Chikugunya
95% CI
IRR
Mean
(Exposure)
Temperature (°C)
[1−Month Lag]
D2:Bangkok
Chikugunya
95% CI
IRR
Mean
(Exposure)
Temperature (°C)
[2−Month Lag]
E1:Bangkok
Chikugunya
95% CI
IRR
Mean
(Exposure)
SO_2 Surface Concentration (mg/m³)
[1−Month Lag]
E2:Bangkok
Chikugunya
95% CI
IRR
Mean
(Exposure)
SO_2 Surface Concentration (mg/m³)
[2−Month Lag]
F1:Bangkok
Chikugunya
95% CI
IRR
Mean
(Exposure)
CO Surface Concentration (ppb)
[1−Month Lag]
F2:Bangkok
Chikugunya
95% CI
IRR
Mean
(Exposure)
CO Surface Concentration (ppb)
[2−Month Lag]
G1:Bangkok
Chikugunya
95% CI
IRR
Mean
(Exposure)
$PM_{2.5}$ Surface Concentration (μg/m³)
[1−Month Lag]
G2:Bangkok
Chikugunya
95% CI
IRR
Mean
(Exposure)
$PM_{2.5}$ Surface Concentration (μg/m³)
[2−Month Lag]

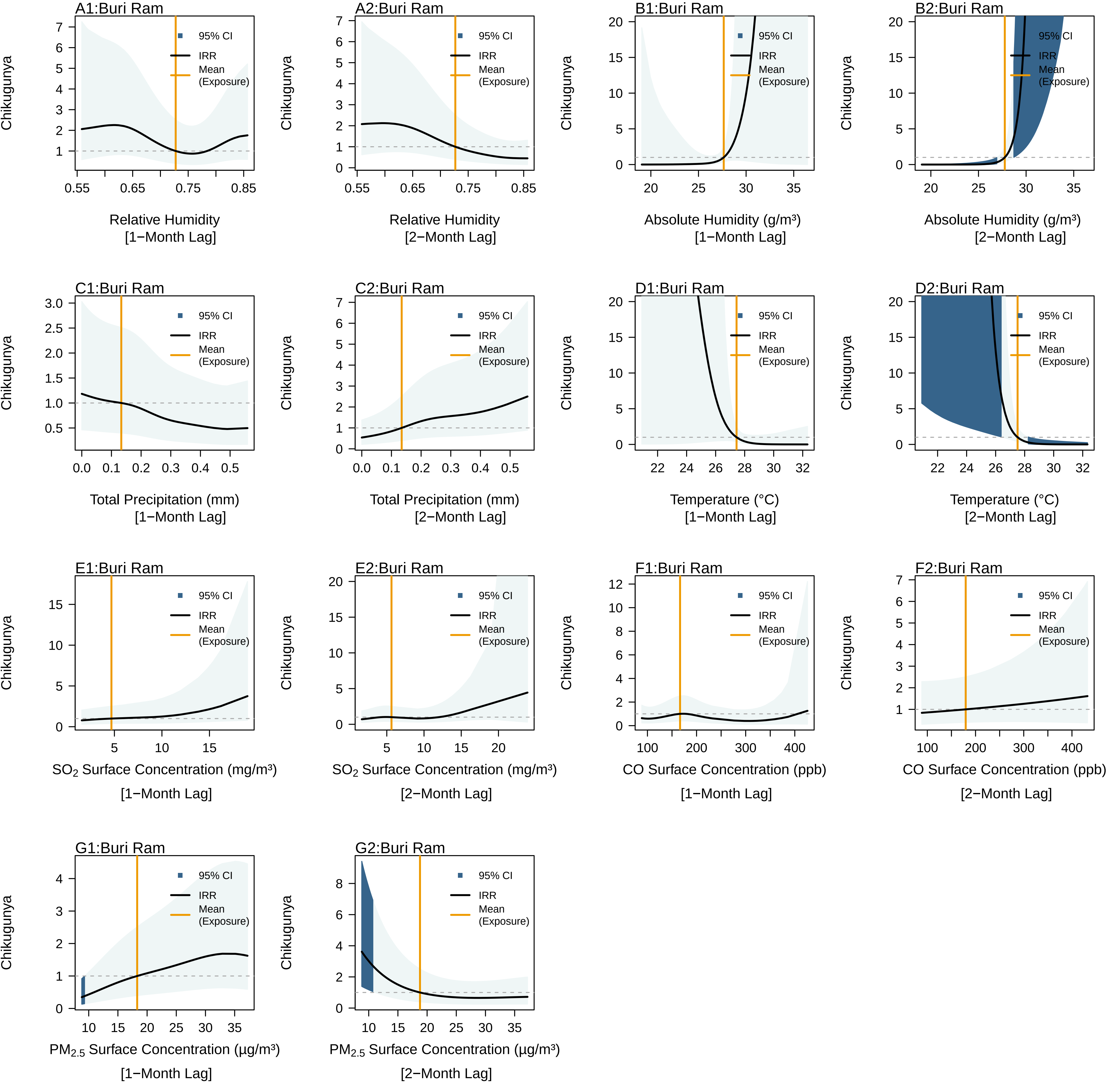

A1:Buri Ram
Chikugunya
95% CI
IRR
Mean (Exposure)
Relative Humidity [1−Month Lag]
A2:Buri Ram
Relative Humidity [2−Month Lag]
B1:Buri Ram
Absolute Humidity (g/m³) [1−Month Lag]
B2:Buri Ram
Absolute Humidity (g/m³) [2−Month Lag]
C1:Buri Ram
Total Precipitation (mm) [1−Month Lag]
C2:Buri Ram
Total Precipitation (mm) [2−Month Lag]
D1:Buri Ram
Temperature (°C) [1−Month Lag]
D2:Buri Ram
Temperature (°C) [2−Month Lag]
E1:Buri Ram
SO2 Surface Concentration (mg/m³) [1−Month Lag]
E2:Buri Ram
SO2 Surface Concentration (mg/m³) [2−Month Lag]
F1:Buri Ram
CO Surface Concentration (ppb) [1−Month Lag]
F2:Buri Ram
CO Surface Concentration (ppb) [2−Month Lag]
G1:Buri Ram
PM2.5 Surface Concentration (μg/m³) [1−Month Lag]
G2:Buri Ram
PM2.5 Surface Concentration (μg/m³) [2−Month Lag]

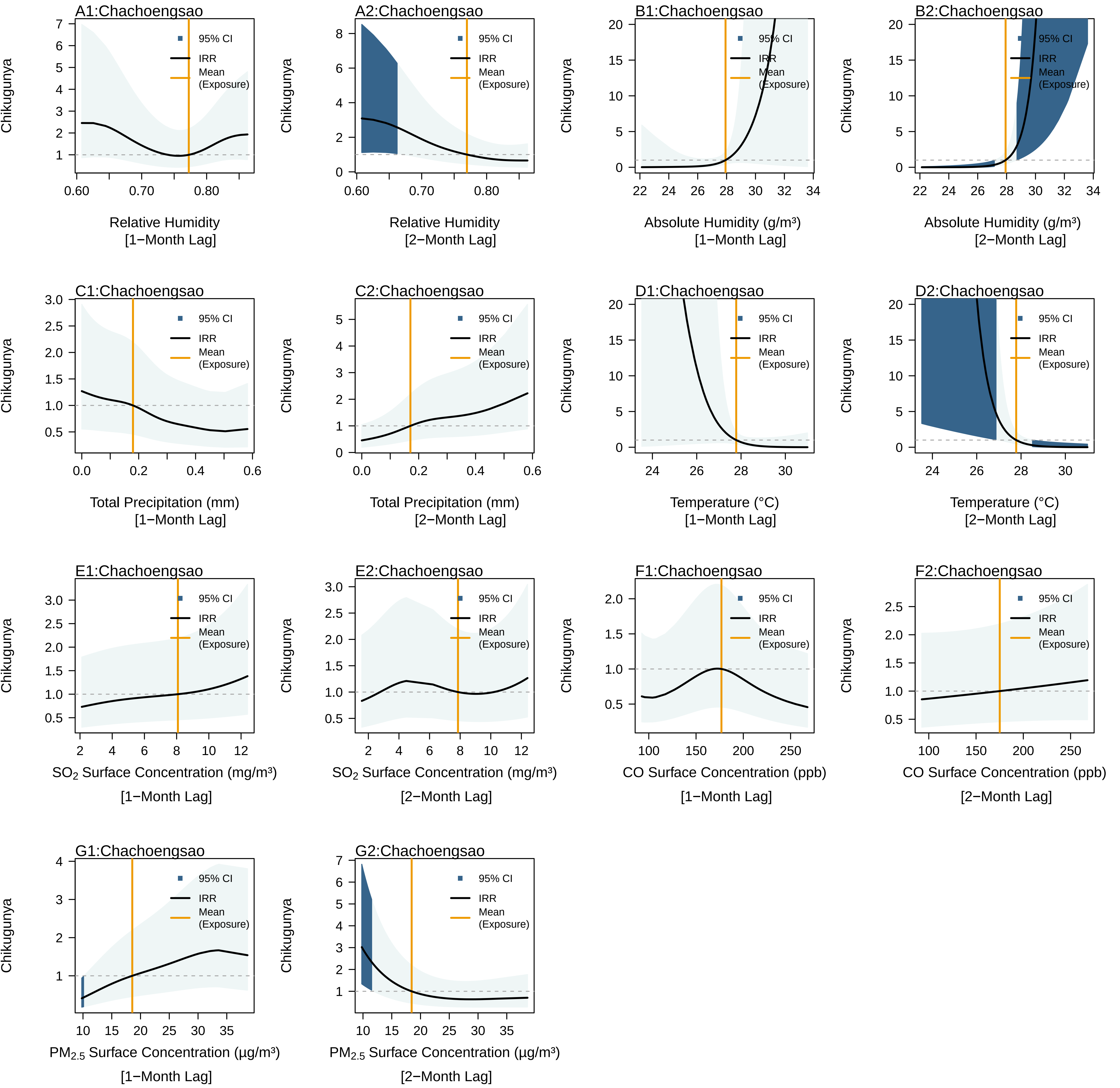
A1:Chachoengsao
Chikugunya
95% CI
IRR
Mean (Exposure)
Relative Humidity [1−Month Lag]
A2:Chachoengsao
Chikugunya
95% CI
IRR
Mean (Exposure)
Relative Humidity [2−Month Lag]
B1:Chachoengsao
Chikugunya
95% CI
IRR
Mean (Exposure)
Absolute Humidity (g/m³) [1−Month Lag]
B2:Chachoengsao
Chikugunya
95% CI
IRR
Mean (Exposure)
Absolute Humidity (g/m³) [2−Month Lag]
C1:Chachoengsao
Chikugunya
95% CI
IRR
Mean (Exposure)
Total Precipitation (mm) [1−Month Lag]
C2:Chachoengsao
Chikugunya
95% CI
IRR
Mean (Exposure)
Total Precipitation (mm) [2−Month Lag]
D1:Chachoengsao
Chikugunya
95% CI
IRR
Mean (Exposure)
Temperature (°C) [1−Month Lag]
D2:Chachoengsao
Chikugunya
95% CI
IRR
Mean (Exposure)
Temperature (°C) [2−Month Lag]
E1:Chachoengsao
Chikugunya
95% CI
IRR
Mean (Exposure)
SO2 Surface Concentration (mg/m³) [1−Month Lag]
E2:Chachoengsao
Chikugunya
95% CI
IRR
Mean (Exposure)
SO2 Surface Concentration (mg/m³) [2−Month Lag]
F1:Chachoengsao
Chikugunya
95% CI
IRR
Mean (Exposure)
CO Surface Concentration (ppb) [1−Month Lag]
F2:Chachoengsao
Chikugunya
95% CI
IRR
Mean (Exposure)
CO Surface Concentration (ppb) [2−Month Lag]
G1:Chachoengsao
Chikugunya
95% CI
IRR
Mean (Exposure)
PM2.5 Surface Concentration (µg/m³) [1−Month Lag]
G2:Chachoengsao
Chikugunya
95% CI
IRR
Mean (Exposure)
PM2.5 Surface Concentration (µg/m³) [2−Month Lag]

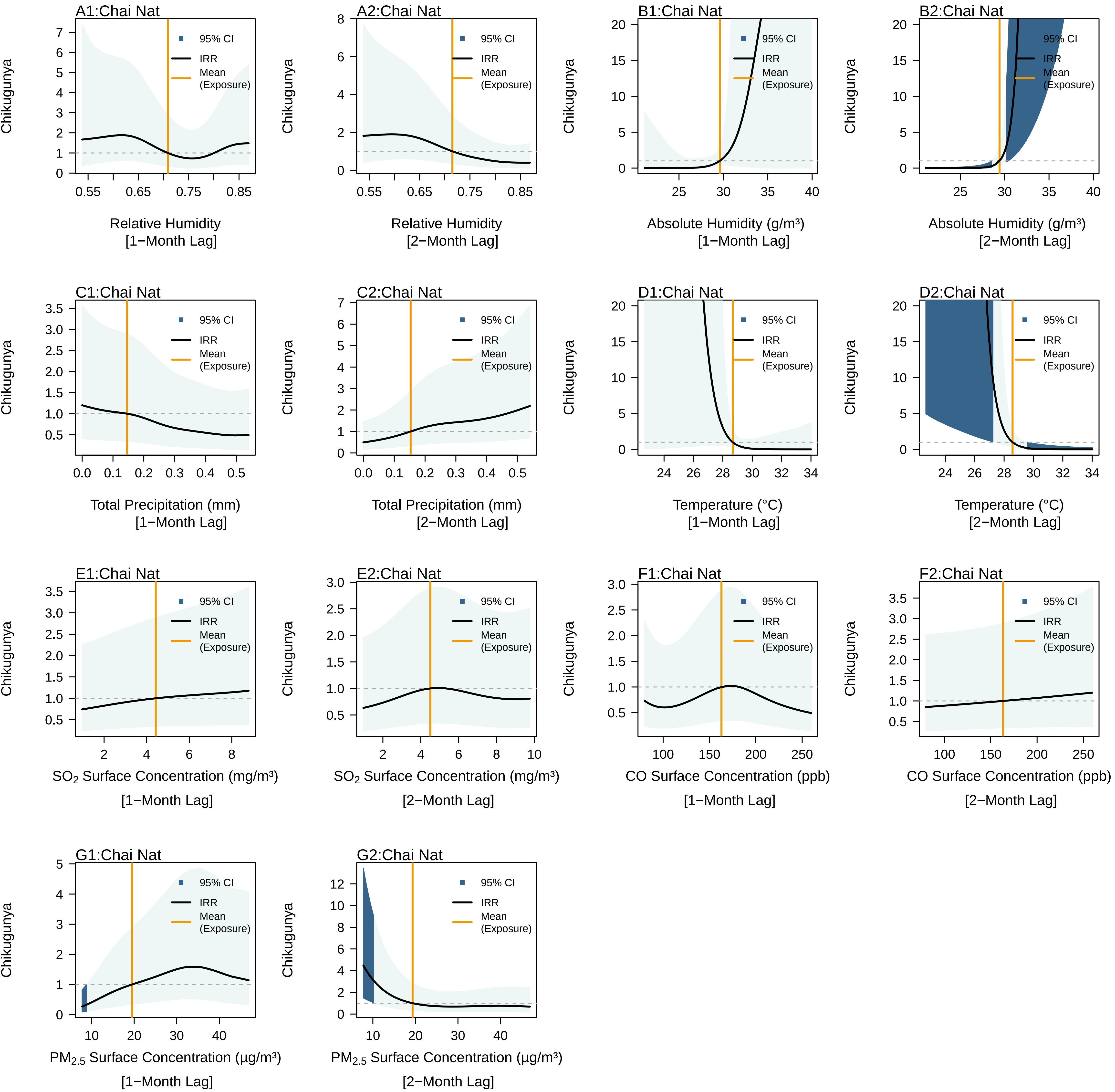
A1:Chai Nat
A2:Chai Nat
B1:Chai Nat
B2:Chai Nat
C1:Chai Nat
C2:Chai Nat
D1:Chai Nat
D2:Chai Nat
E1:Chai Nat
E2:Chai Nat
F1:Chai Nat
F2:Chai Nat
G1:Chai Nat
G2:Chai Nat
Chikugunya
95% CI
IRR
Mean (Exposure)
Relative Humidity [1−Month Lag]
Relative Humidity [2−Month Lag]
Absolute Humidity (g/m³) [1−Month Lag]
Absolute Humidity (g/m³) [2−Month Lag]
Total Precipitation (mm) [1−Month Lag]
Total Precipitation (mm) [2−Month Lag]
Temperature (°C) [1−Month Lag]
Temperature (°C) [2−Month Lag]
SO_2 Surface Concentration (mg/m³) [1−Month Lag]
SO_2 Surface Concentration (mg/m³) [2−Month Lag]
CO Surface Concentration (ppb) [1−Month Lag]
CO Surface Concentration (ppb) [2−Month Lag]
$PM_{2.5}$ Surface Concentration (µg/m³) [1−Month Lag]
$PM_{2.5}$ Surface Concentration (µg/m³) [2−Month Lag]

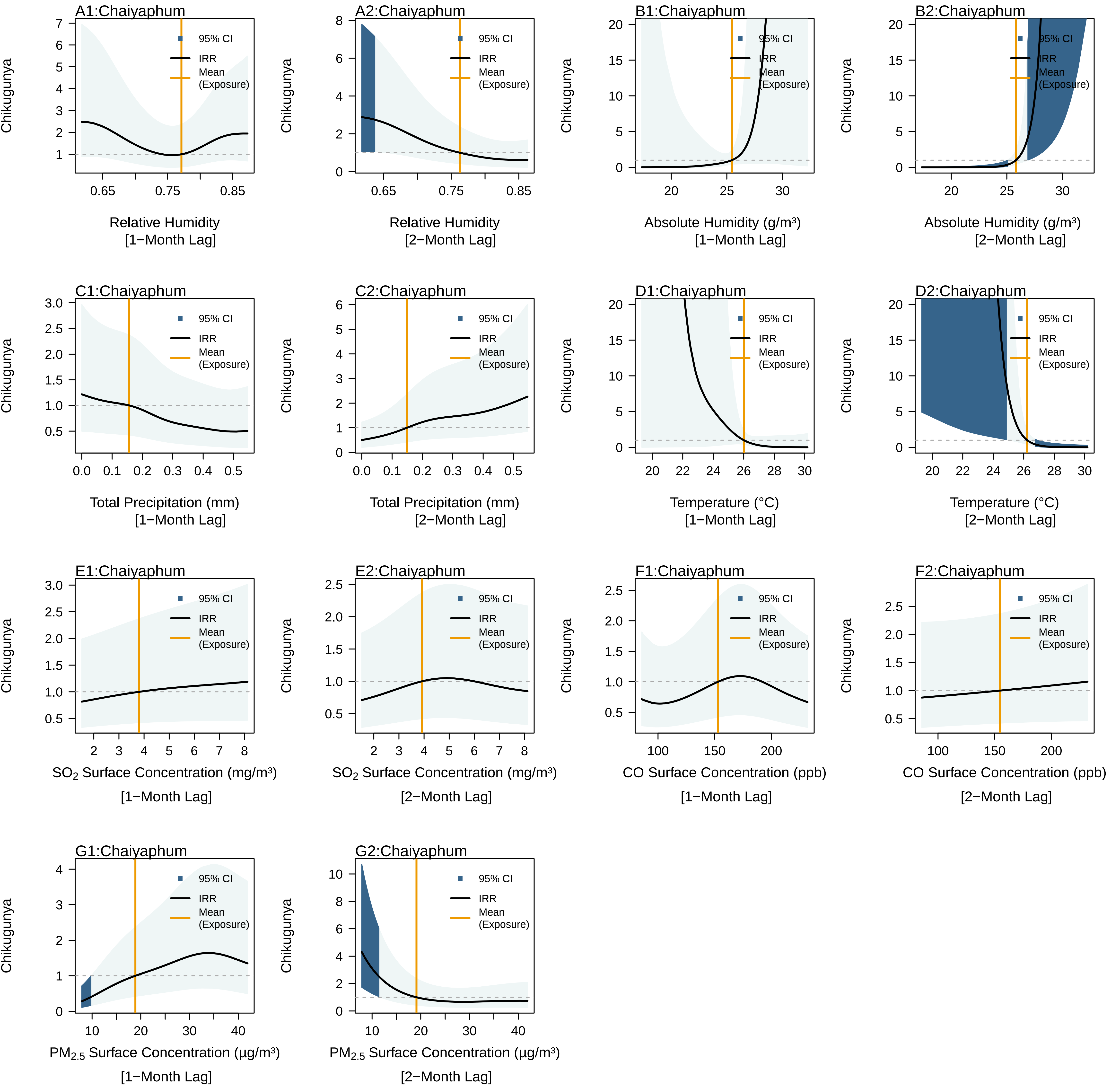
A1:Chaiyaphum
Chikungunya
95% CI
IRR
Mean
(Exposure)
Relative Humidity
[1−Month Lag]
A2:Chaiyaphum
Chikungunya
Relative Humidity
[2−Month Lag]
B1:Chaiyaphum
Chikungunya
Absolute Humidity (g/m³)
[1−Month Lag]
B2:Chaiyaphum
Chikungunya
Absolute Humidity (g/m³)
[2−Month Lag]
C1:Chaiyaphum
Chikungunya
Total Precipitation (mm)
[1−Month Lag]
C2:Chaiyaphum
Chikungunya
Total Precipitation (mm)
[2−Month Lag]
D1:Chaiyaphum
Chikungunya
Temperature (°C)
[1−Month Lag]
D2:Chaiyaphum
Chikungunya
Temperature (°C)
[2−Month Lag]
E1:Chaiyaphum
Chikungunya
SO_2 Surface Concentration (mg/m³)
[1−Month Lag]
E2:Chaiyaphum
Chikungunya
SO_2 Surface Concentration (mg/m³)
[2−Month Lag]
F1:Chaiyaphum
Chikungunya
CO Surface Concentration (ppb)
[1−Month Lag]
F2:Chaiyaphum
Chikungunya
CO Surface Concentration (ppb)
[2−Month Lag]
G1:Chaiyaphum
Chikungunya
$PM_{2.5}$ Surface Concentration (µg/m³)
[1−Month Lag]
G2:Chaiyaphum
Chikungunya
$PM_{2.5}$ Surface Concentration (µg/m³)
[2−Month Lag]

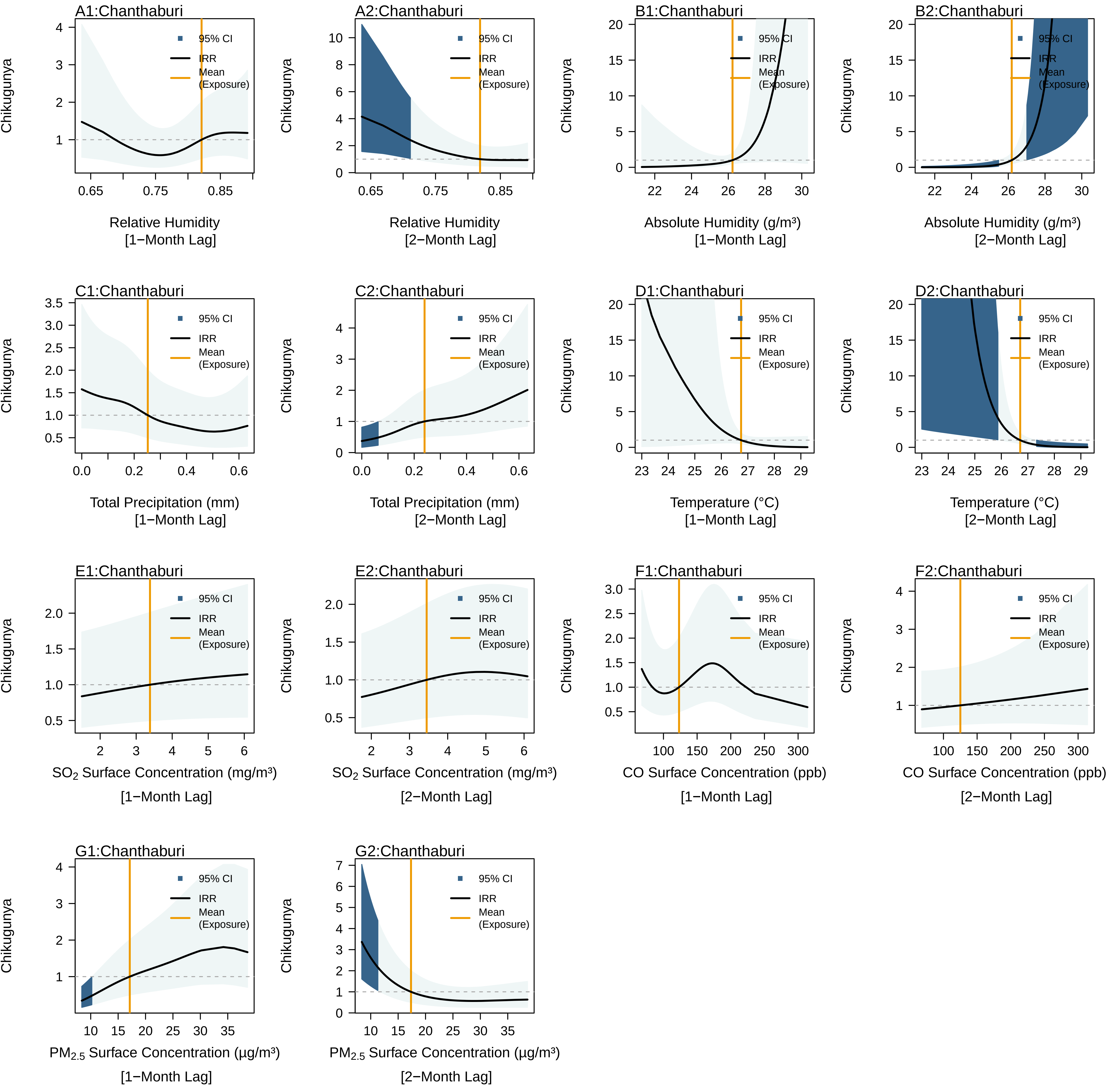
A1:Chanthaburi
Chikugunya
95% CI
IRR
Mean (Exposure)
Relative Humidity [1−Month Lag]
A2:Chanthaburi
Chikugunya
95% CI
IRR
Mean (Exposure)
Relative Humidity [2−Month Lag]
B1:Chanthaburi
Chikugunya
95% CI
IRR
Mean (Exposure)
Absolute Humidity (g/m³) [1−Month Lag]
B2:Chanthaburi
Chikugunya
95% CI
IRR
Mean (Exposure)
Absolute Humidity (g/m³) [2−Month Lag]
C1:Chanthaburi
Chikugunya
95% CI
IRR
Mean (Exposure)
Total Precipitation (mm) [1−Month Lag]
C2:Chanthaburi
Chikugunya
95% CI
IRR
Mean (Exposure)
Total Precipitation (mm) [2−Month Lag]
D1:Chanthaburi
Chikugunya
95% CI
IRR
Mean (Exposure)
Temperature (°C) [1−Month Lag]
D2:Chanthaburi
Chikugunya
95% CI
IRR
Mean (Exposure)
Temperature (°C) [2−Month Lag]
E1:Chanthaburi
Chikugunya
95% CI
IRR
Mean (Exposure)
SO2 Surface Concentration (mg/m³) [1−Month Lag]
E2:Chanthaburi
Chikugunya
95% CI
IRR
Mean (Exposure)
SO2 Surface Concentration (mg/m³) [2−Month Lag]
F1:Chanthaburi
Chikugunya
95% CI
IRR
Mean (Exposure)
CO Surface Concentration (ppb) [1−Month Lag]
F2:Chanthaburi
Chikugunya
95% CI
IRR
Mean (Exposure)
CO Surface Concentration (ppb) [2−Month Lag]
G1:Chanthaburi
Chikugunya
95% CI
IRR
Mean (Exposure)
PM2.5 Surface Concentration (µg/m³) [1−Month Lag]
G2:Chanthaburi
Chikugunya
95% CI
IRR
Mean (Exposure)
PM2.5 Surface Concentration (µg/m³) [2−Month Lag]

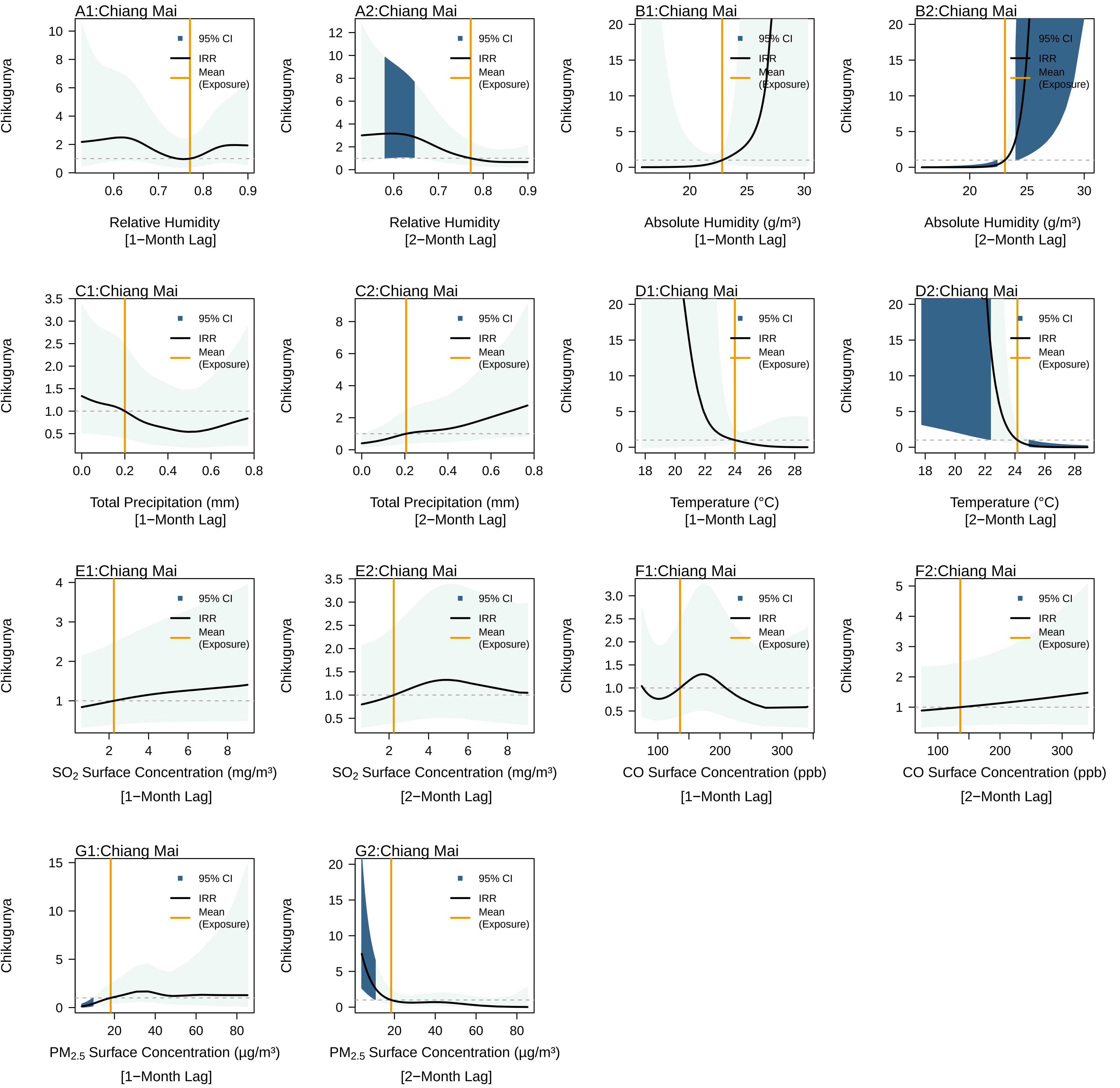
A1:Chiang Mai
95% CI
IRR
Mean (Exposure)
Chikugunya
Relative Humidity [1−Month Lag]
A2:Chiang Mai
Relative Humidity [2−Month Lag]
B1:Chiang Mai
Absolute Humidity (g/m³) [1−Month Lag]
B2:Chiang Mai
Absolute Humidity (g/m³) [2−Month Lag]
C1:Chiang Mai
Total Precipitation (mm) [1−Month Lag]
C2:Chiang Mai
Total Precipitation (mm) [2−Month Lag]
D1:Chiang Mai
Temperature (°C) [1−Month Lag]
D2:Chiang Mai
Temperature (°C) [2−Month Lag]
E1:Chiang Mai
SO_2 Surface Concentration (mg/m³) [1−Month Lag]
E2:Chiang Mai
SO_2 Surface Concentration (mg/m³) [2−Month Lag]
F1:Chiang Mai
CO Surface Concentration (ppb) [1−Month Lag]
F2:Chiang Mai
CO Surface Concentration (ppb) [2−Month Lag]
G1:Chiang Mai
$PM_{2.5}$ Surface Concentration (µg/m³) [1−Month Lag]
G2:Chiang Mai
$PM_{2.5}$ Surface Concentration (µg/m³) [2−Month Lag]

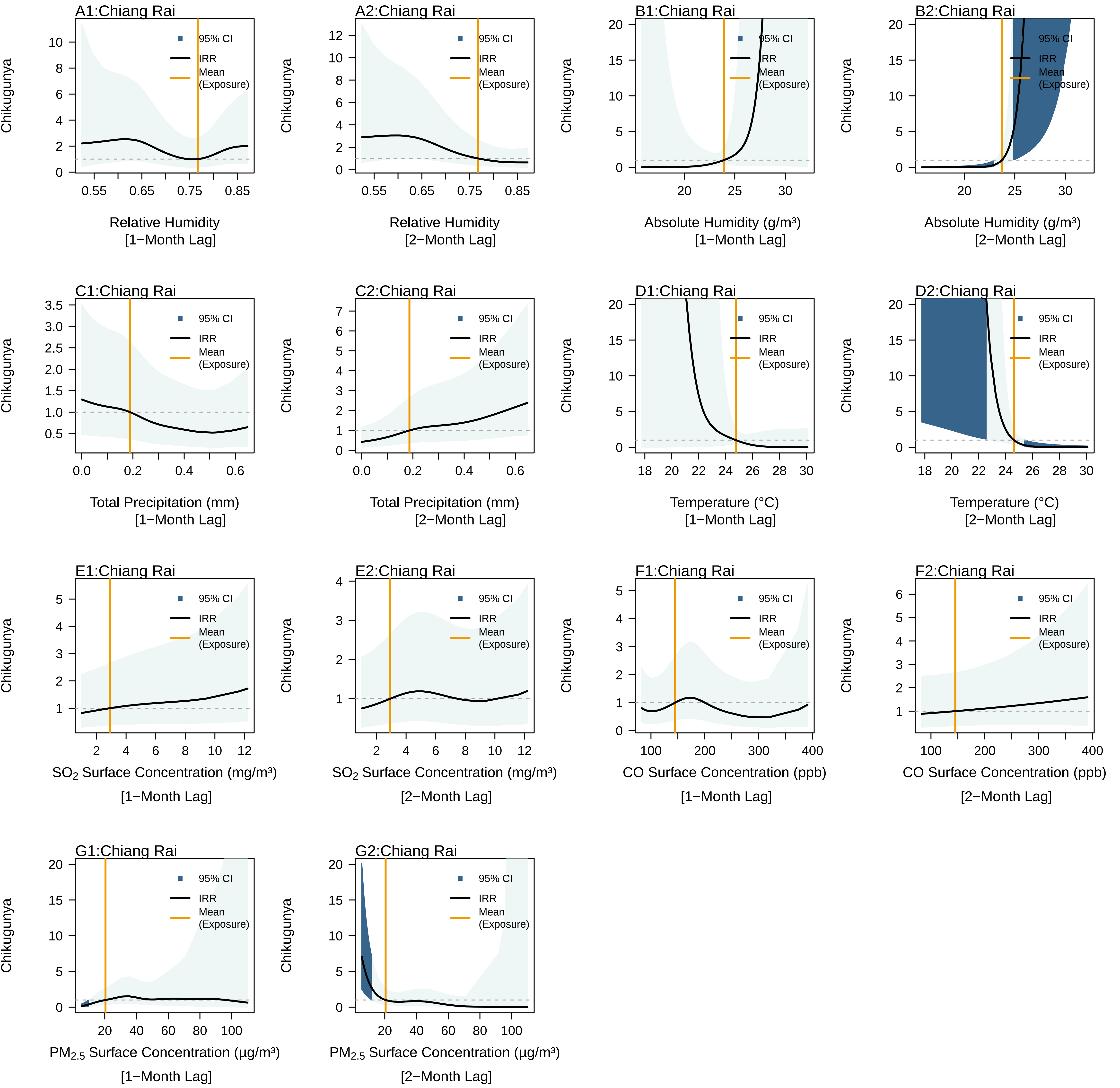

A1:Chiang Rai
Chikungunya
95% CI
IRR
Mean (Exposure)
Relative Humidity [1−Month Lag]
A2:Chiang Rai
Chikungunya
95% CI
IRR
Mean (Exposure)
Relative Humidity [2−Month Lag]
B1:Chiang Rai
Chikungunya
95% CI
IRR
Mean (Exposure)
Absolute Humidity (g/m³) [1−Month Lag]
B2:Chiang Rai
Chikungunya
95% CI
IRR
Mean (Exposure)
Absolute Humidity (g/m³) [2−Month Lag]
C1:Chiang Rai
Chikungunya
95% CI
IRR
Mean (Exposure)
Total Precipitation (mm) [1−Month Lag]
C2:Chiang Rai
Chikungunya
95% CI
IRR
Mean (Exposure)
Total Precipitation (mm) [2−Month Lag]
D1:Chiang Rai
Chikungunya
95% CI
IRR
Mean (Exposure)
Temperature (°C) [1−Month Lag]
D2:Chiang Rai
Chikungunya
95% CI
IRR
Mean (Exposure)
Temperature (°C) [2−Month Lag]
E1:Chiang Rai
Chikungunya
95% CI
IRR
Mean (Exposure)
SO2 Surface Concentration (mg/m³) [1−Month Lag]
E2:Chiang Rai
Chikungunya
95% CI
IRR
Mean (Exposure)
SO2 Surface Concentration (mg/m³) [2−Month Lag]
F1:Chiang Rai
Chikungunya
95% CI
IRR
Mean (Exposure)
CO Surface Concentration (ppb) [1−Month Lag]
F2:Chiang Rai
Chikungunya
95% CI
IRR
Mean (Exposure)
CO Surface Concentration (ppb) [2−Month Lag]
G1:Chiang Rai
Chikungunya
95% CI
IRR
Mean (Exposure)
PM2.5 Surface Concentration (µg/m³) [1−Month Lag]
G2:Chiang Rai
Chikungunya
95% CI
IRR
Mean (Exposure)
PM2.5 Surface Concentration (µg/m³) [2−Month Lag]

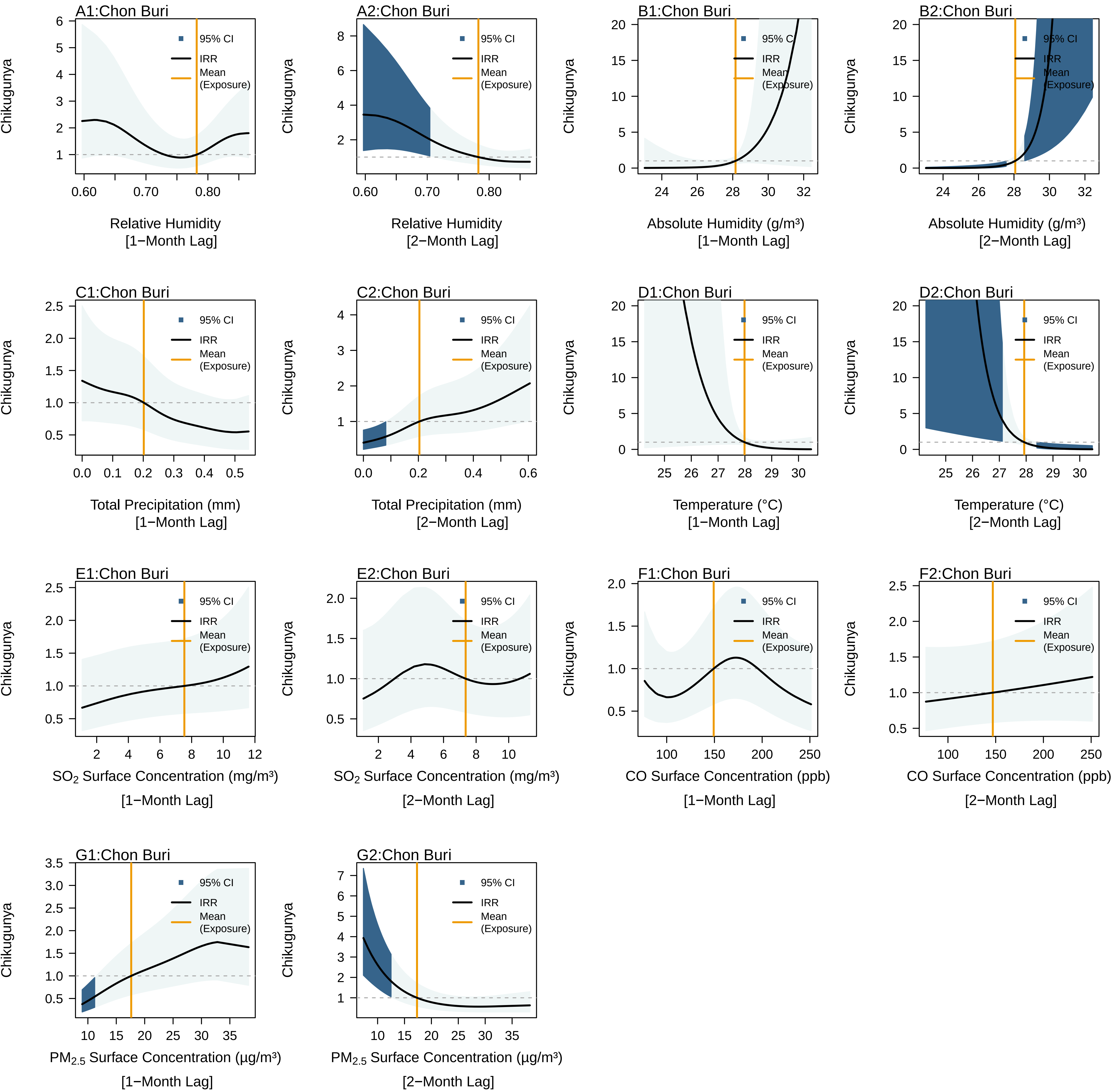
A1:Chon Buri
A2:Chon Buri
B1:Chon Buri
B2:Chon Buri
C1:Chon Buri
C2:Chon Buri
D1:Chon Buri
D2:Chon Buri
E1:Chon Buri
E2:Chon Buri
F1:Chon Buri
F2:Chon Buri
G1:Chon Buri
G2:Chon Buri
Chikugunya
95% CI
IRR
Mean (Exposure)
Relative Humidity [1−Month Lag]
Relative Humidity [2−Month Lag]
Absolute Humidity (g/m³) [1−Month Lag]
Absolute Humidity (g/m³) [2−Month Lag]
Total Precipitation (mm) [1−Month Lag]
Total Precipitation (mm) [2−Month Lag]
Temperature (°C) [1−Month Lag]
Temperature (°C) [2−Month Lag]
SO2 Surface Concentration (mg/m³) [1−Month Lag]
SO2 Surface Concentration (mg/m³) [2−Month Lag]
CO Surface Concentration (ppb) [1−Month Lag]
CO Surface Concentration (ppb) [2−Month Lag]
PM2.5 Surface Concentration (μg/m³) [1−Month Lag]
PM2.5 Surface Concentration (μg/m³) [2−Month Lag]

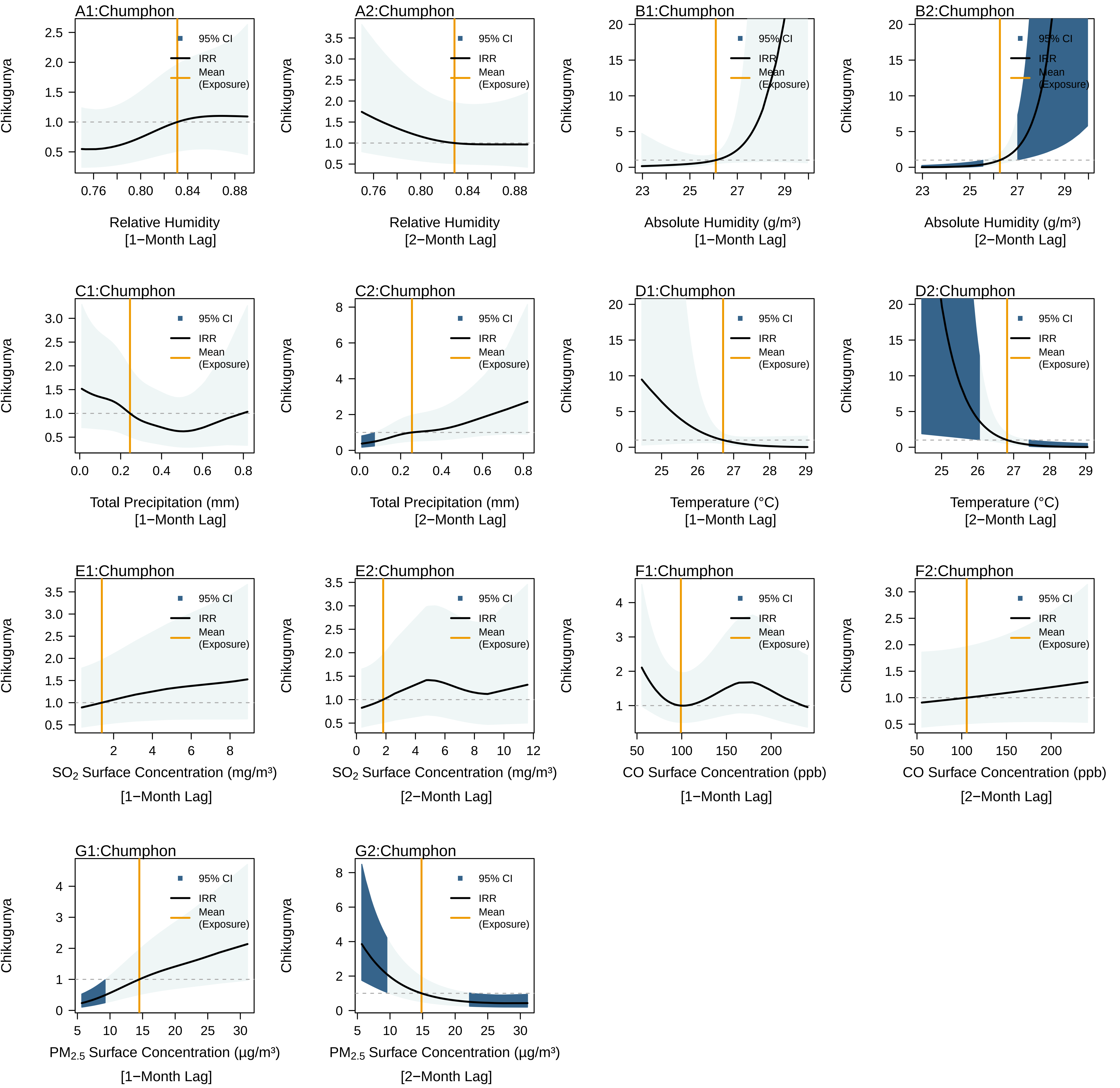

A1:Chumphon
95% CI
IRR
Mean (Exposure)
Chikugunya
Relative Humidity [1−Month Lag]
A2:Chumphon
Relative Humidity [2−Month Lag]
B1:Chumphon
Absolute Humidity (g/m³) [1−Month Lag]
B2:Chumphon
Absolute Humidity (g/m³) [2−Month Lag]
C1:Chumphon
Total Precipitation (mm) [1−Month Lag]
C2:Chumphon
Total Precipitation (mm) [2−Month Lag]
D1:Chumphon
Temperature (°C) [1−Month Lag]
D2:Chumphon
Temperature (°C) [2−Month Lag]
E1:Chumphon
SO2 Surface Concentration (mg/m³) [1−Month Lag]
E2:Chumphon
SO2 Surface Concentration (mg/m³) [2−Month Lag]
F1:Chumphon
CO Surface Concentration (ppb) [1−Month Lag]
F2:Chumphon
CO Surface Concentration (ppb) [2−Month Lag]
G1:Chumphon
PM2.5 Surface Concentration (µg/m³) [1−Month Lag]
G2:Chumphon
PM2.5 Surface Concentration (µg/m³) [2−Month Lag]

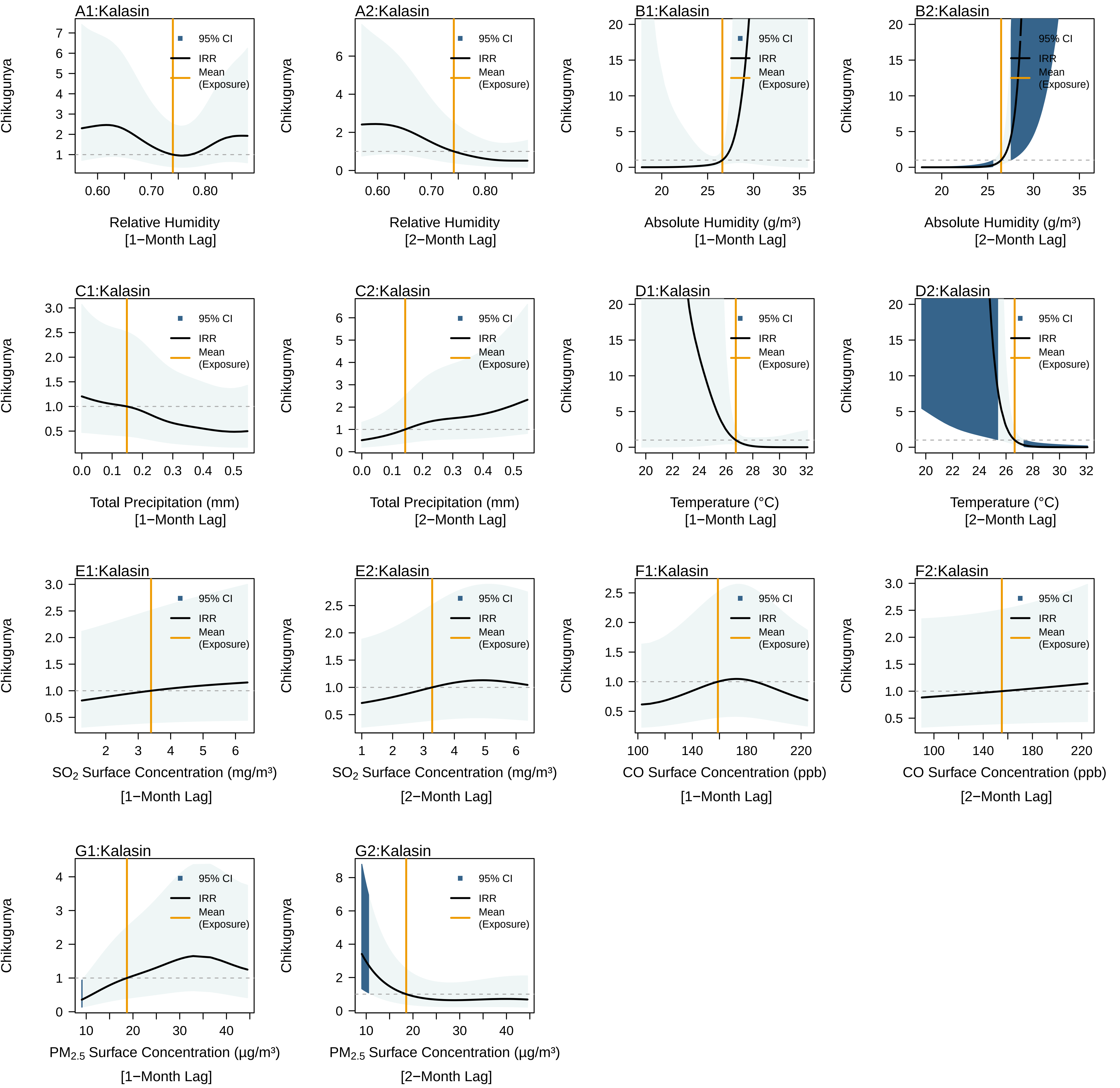

A1:Kalasin
Chikungunya
95% CI
IRR
Mean (Exposure)
Relative Humidity [1−Month Lag]
A2:Kalasin
Relative Humidity [2−Month Lag]
B1:Kalasin
Absolute Humidity (g/m³) [1−Month Lag]
B2:Kalasin
Absolute Humidity (g/m³) [2−Month Lag]
C1:Kalasin
Total Precipitation (mm) [1−Month Lag]
C2:Kalasin
Total Precipitation (mm) [2−Month Lag]
D1:Kalasin
Temperature (°C) [1−Month Lag]
D2:Kalasin
Temperature (°C) [2−Month Lag]
E1:Kalasin
SO_2 Surface Concentration (mg/m³) [1−Month Lag]
E2:Kalasin
SO_2 Surface Concentration (mg/m³) [2−Month Lag]
F1:Kalasin
CO Surface Concentration (ppb) [1−Month Lag]
F2:Kalasin
CO Surface Concentration (ppb) [2−Month Lag]
G1:Kalasin
$PM_{2.5}$ Surface Concentration (µg/m³) [1−Month Lag]
G2:Kalasin
$PM_{2.5}$ Surface Concentration (µg/m³) [2−Month Lag]

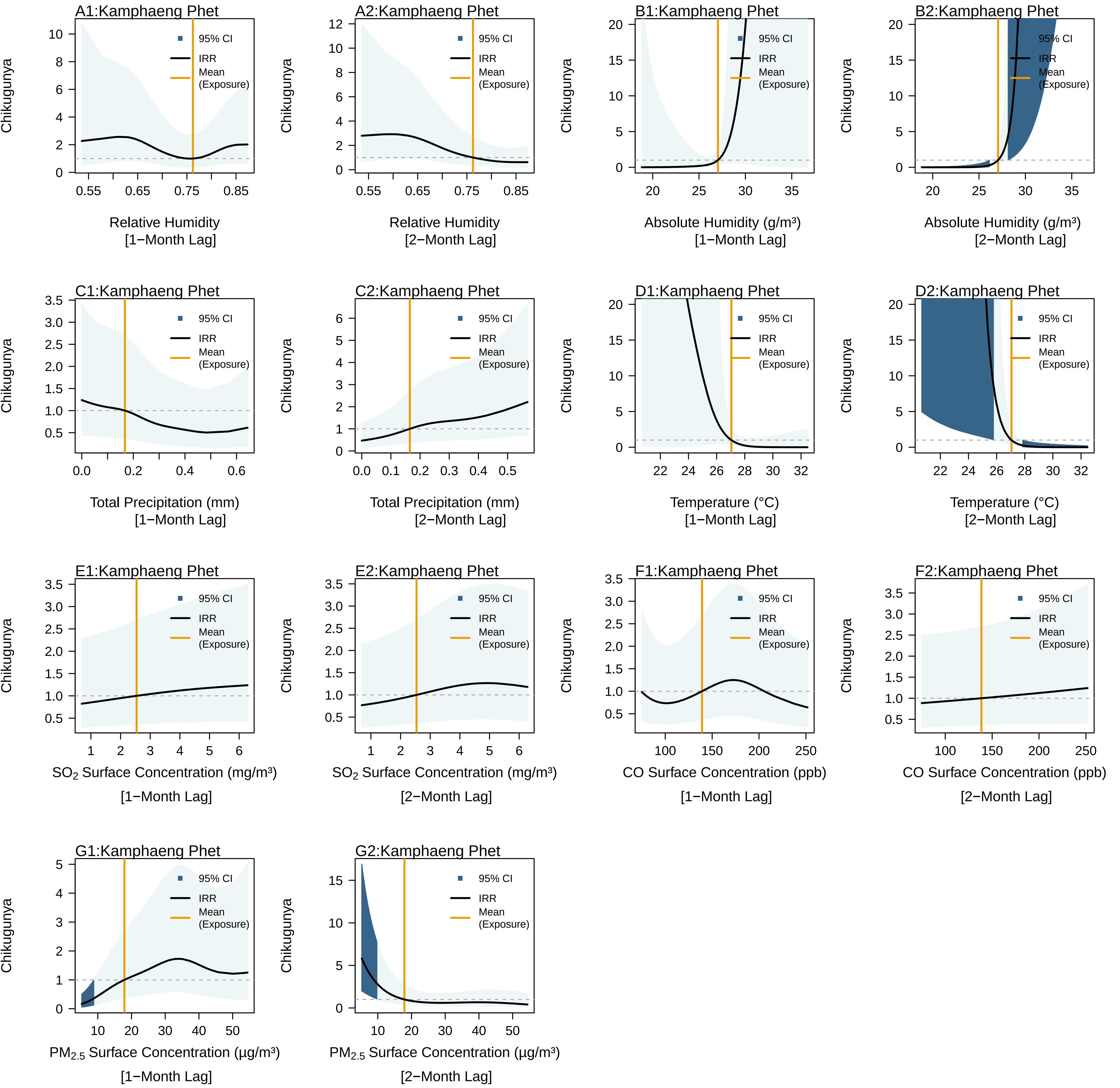
A1:Kamphaeng Phet
Chikugunya
95% CI
IRR
Mean (Exposure)
Relative Humidity [1−Month Lag]
A2:Kamphaeng Phet
Chikugunya
95% CI
IRR
Mean (Exposure)
Relative Humidity [2−Month Lag]
B1:Kamphaeng Phet
Chikugunya
95% CI
IRR
Mean (Exposure)
Absolute Humidity (g/m³) [1−Month Lag]
B2:Kamphaeng Phet
Chikugunya
95% CI
IRR
Mean (Exposure)
Absolute Humidity (g/m³) [2−Month Lag]
C1:Kamphaeng Phet
Chikugunya
95% CI
IRR
Mean (Exposure)
Total Precipitation (mm) [1−Month Lag]
C2:Kamphaeng Phet
Chikugunya
95% CI
IRR
Mean (Exposure)
Total Precipitation (mm) [2−Month Lag]
D1:Kamphaeng Phet
Chikugunya
95% CI
IRR
Mean (Exposure)
Temperature (°C) [1−Month Lag]
D2:Kamphaeng Phet
Chikugunya
95% CI
IRR
Mean (Exposure)
Temperature (°C) [2−Month Lag]
E1:Kamphaeng Phet
Chikugunya
95% CI
IRR
Mean (Exposure)
SO_2 Surface Concentration (mg/m³) [1−Month Lag]
E2:Kamphaeng Phet
Chikugunya
95% CI
IRR
Mean (Exposure)
SO_2 Surface Concentration (mg/m³) [2−Month Lag]
F1:Kamphaeng Phet
Chikugunya
95% CI
IRR
Mean (Exposure)
CO Surface Concentration (ppb) [1−Month Lag]
F2:Kamphaeng Phet
Chikugunya
95% CI
IRR
Mean (Exposure)
CO Surface Concentration (ppb) [2−Month Lag]
G1:Kamphaeng Phet
Chikugunya
95% CI
IRR
Mean (Exposure)
$PM_{2.5}$ Surface Concentration (µg/m³) [1−Month Lag]
G2:Kamphaeng Phet
Chikugunya
95% CI
IRR
Mean (Exposure)
$PM_{2.5}$ Surface Concentration (µg/m³) [2−Month Lag]

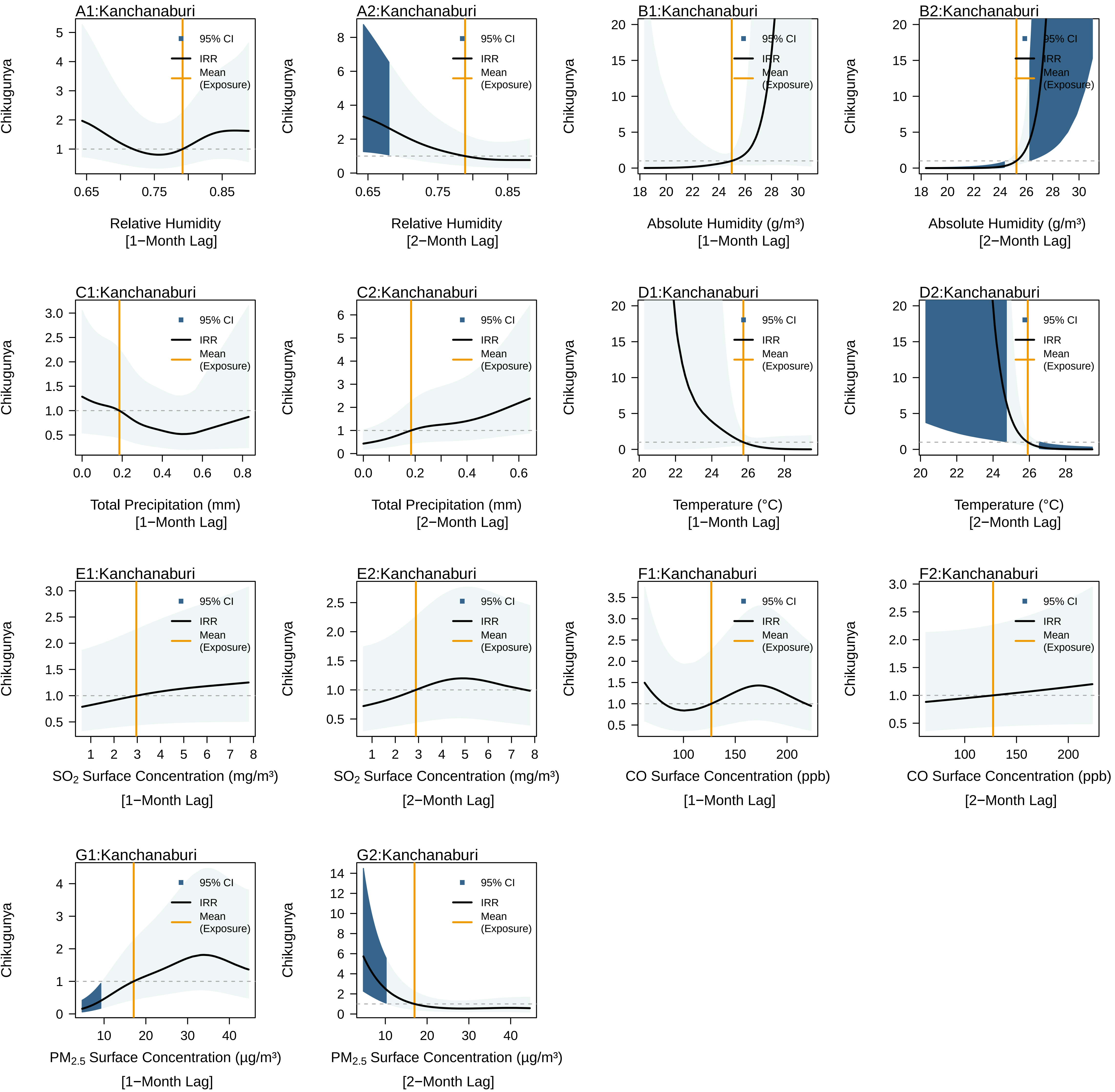
A1:Kanchanaburi
A2:Kanchanaburi
B1:Kanchanaburi
B2:Kanchanaburi
C1:Kanchanaburi
C2:Kanchanaburi
D1:Kanchanaburi
D2:Kanchanaburi
E1:Kanchanaburi
E2:Kanchanaburi
F1:Kanchanaburi
F2:Kanchanaburi
G1:Kanchanaburi
G2:Kanchanaburi
Chikugunya
95% CI
IRR
Mean (Exposure)
Relative Humidity [1−Month Lag]
Relative Humidity [2−Month Lag]
Absolute Humidity (g/m³) [1−Month Lag]
Absolute Humidity (g/m³) [2−Month Lag]
Total Precipitation (mm) [1−Month Lag]
Total Precipitation (mm) [2−Month Lag]
Temperature (°C) [1−Month Lag]
Temperature (°C) [2−Month Lag]
SO2 Surface Concentration (mg/m³) [1−Month Lag]
SO2 Surface Concentration (mg/m³) [2−Month Lag]
CO Surface Concentration (ppb) [1−Month Lag]
CO Surface Concentration (ppb) [2−Month Lag]
PM2.5 Surface Concentration (µg/m³) [1−Month Lag]
PM2.5 Surface Concentration (µg/m³) [2−Month Lag]

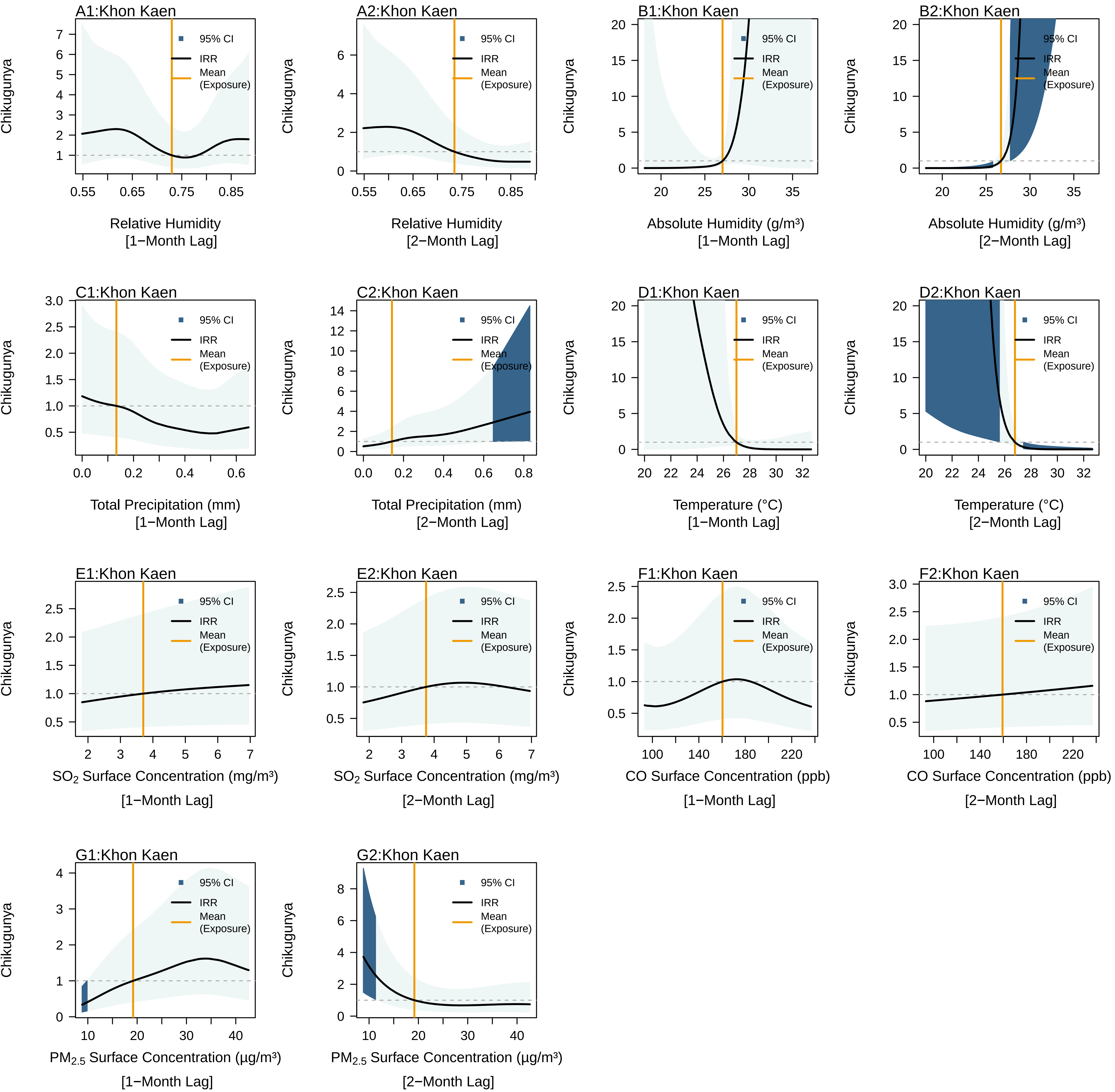
A1:Khon Kaen
A2:Khon Kaen
B1:Khon Kaen
B2:Khon Kaen
C1:Khon Kaen
C2:Khon Kaen
D1:Khon Kaen
D2:Khon Kaen
E1:Khon Kaen
E2:Khon Kaen
F1:Khon Kaen
F2:Khon Kaen
G1:Khon Kaen
G2:Khon Kaen
Chikugunya
95% CI
IRR
Mean (Exposure)
Relative Humidity [1−Month Lag]
Relative Humidity [2−Month Lag]
Absolute Humidity (g/m³) [1−Month Lag]
Absolute Humidity (g/m³) [2−Month Lag]
Total Precipitation (mm) [1−Month Lag]
Total Precipitation (mm) [2−Month Lag]
Temperature (°C) [1−Month Lag]
Temperature (°C) [2−Month Lag]
SO_2 Surface Concentration (mg/m³) [1−Month Lag]
SO_2 Surface Concentration (mg/m³) [2−Month Lag]
CO Surface Concentration (ppb) [1−Month Lag]
CO Surface Concentration (ppb) [2−Month Lag]
$PM_{2.5}$ Surface Concentration (µg/m³) [1−Month Lag]
$PM_{2.5}$ Surface Concentration (µg/m³) [2−Month Lag]

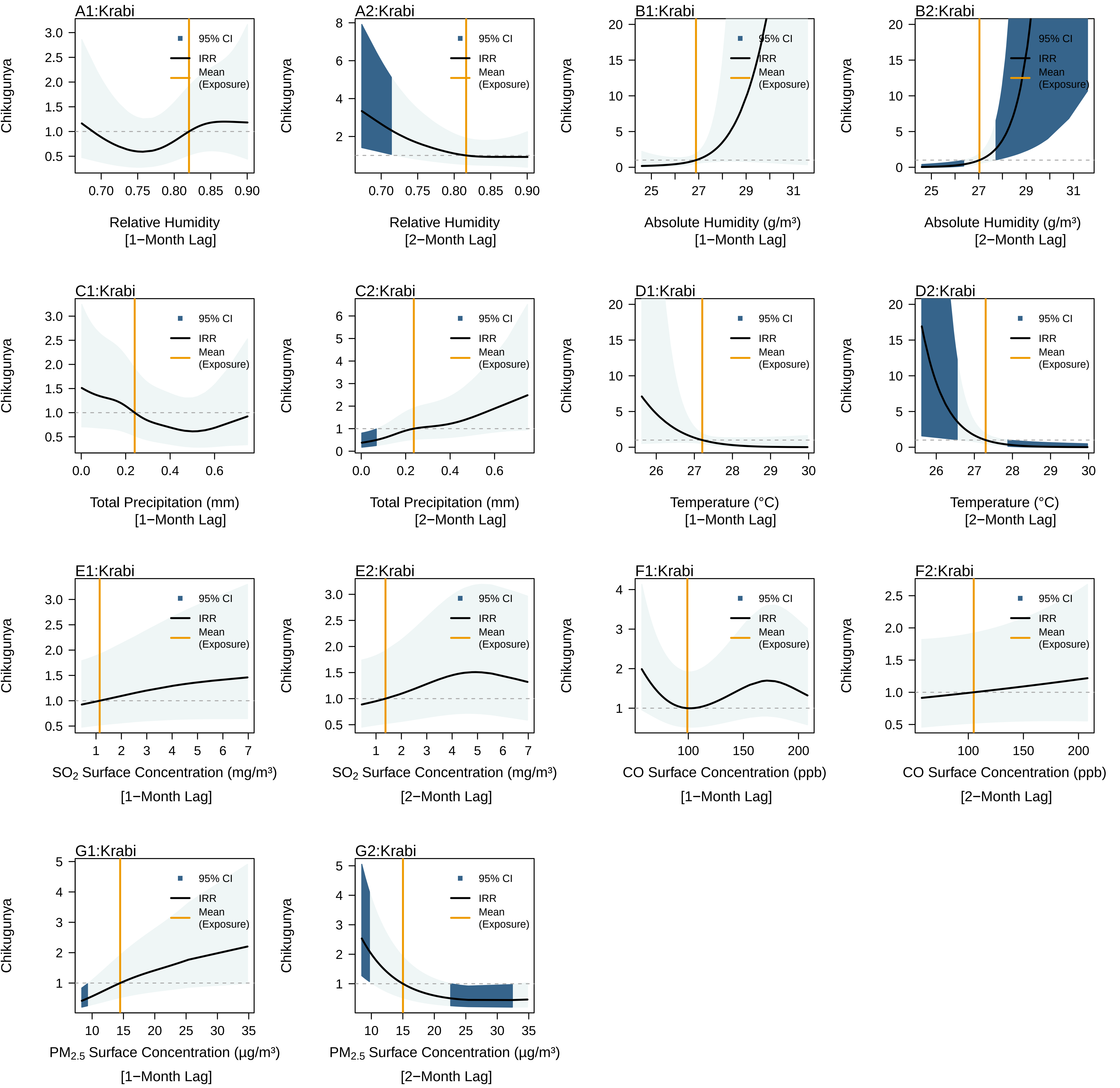
A1:Krabi
Chikugunya
95% CI
IRR
Mean (Exposure)
Relative Humidity [1−Month Lag]
A2:Krabi
Relative Humidity [2−Month Lag]
B1:Krabi
Absolute Humidity (g/m³) [1−Month Lag]
B2:Krabi
Absolute Humidity (g/m³) [2−Month Lag]
C1:Krabi
Total Precipitation (mm) [1−Month Lag]
C2:Krabi
Total Precipitation (mm) [2−Month Lag]
D1:Krabi
Temperature (°C) [1−Month Lag]
D2:Krabi
Temperature (°C) [2−Month Lag]
E1:Krabi
SO_2 Surface Concentration (mg/m³) [1−Month Lag]
E2:Krabi
SO_2 Surface Concentration (mg/m³) [2−Month Lag]
F1:Krabi
CO Surface Concentration (ppb) [1−Month Lag]
F2:Krabi
CO Surface Concentration (ppb) [2−Month Lag]
G1:Krabi
$PM_{2.5}$ Surface Concentration (µg/m³) [1−Month Lag]
G2:Krabi
$PM_{2.5}$ Surface Concentration (µg/m³) [2−Month Lag]

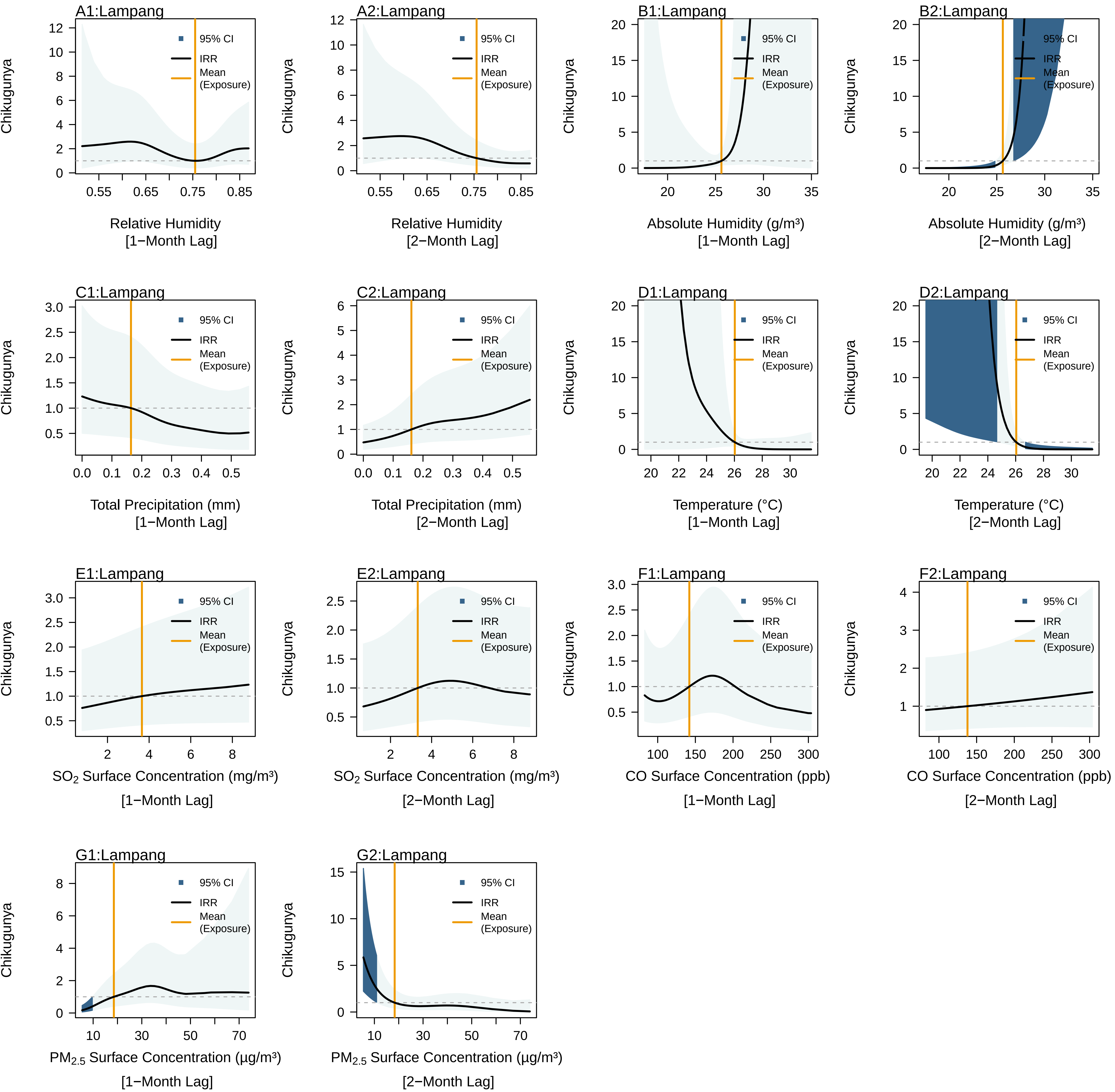
A1:Lampang
A2:Lampang
B1:Lampang
B2:Lampang
C1:Lampang
C2:Lampang
D1:Lampang
D2:Lampang
E1:Lampang
E2:Lampang
F1:Lampang
F2:Lampang
G1:Lampang
G2:Lampang
Chikugunya
95% CI
IRR
Mean (Exposure)
Relative Humidity [1−Month Lag]
Relative Humidity [2−Month Lag]
Absolute Humidity (g/m³) [1−Month Lag]
Absolute Humidity (g/m³) [2−Month Lag]
Total Precipitation (mm) [1−Month Lag]
Total Precipitation (mm) [2−Month Lag]
Temperature (°C) [1−Month Lag]
Temperature (°C) [2−Month Lag]
SO_2 Surface Concentration (mg/m³) [1−Month Lag]
SO_2 Surface Concentration (mg/m³) [2−Month Lag]
CO Surface Concentration (ppb) [1−Month Lag]
CO Surface Concentration (ppb) [2−Month Lag]
$PM_{2.5}$ Surface Concentration (µg/m³) [1−Month Lag]
$PM_{2.5}$ Surface Concentration (µg/m³) [2−Month Lag]

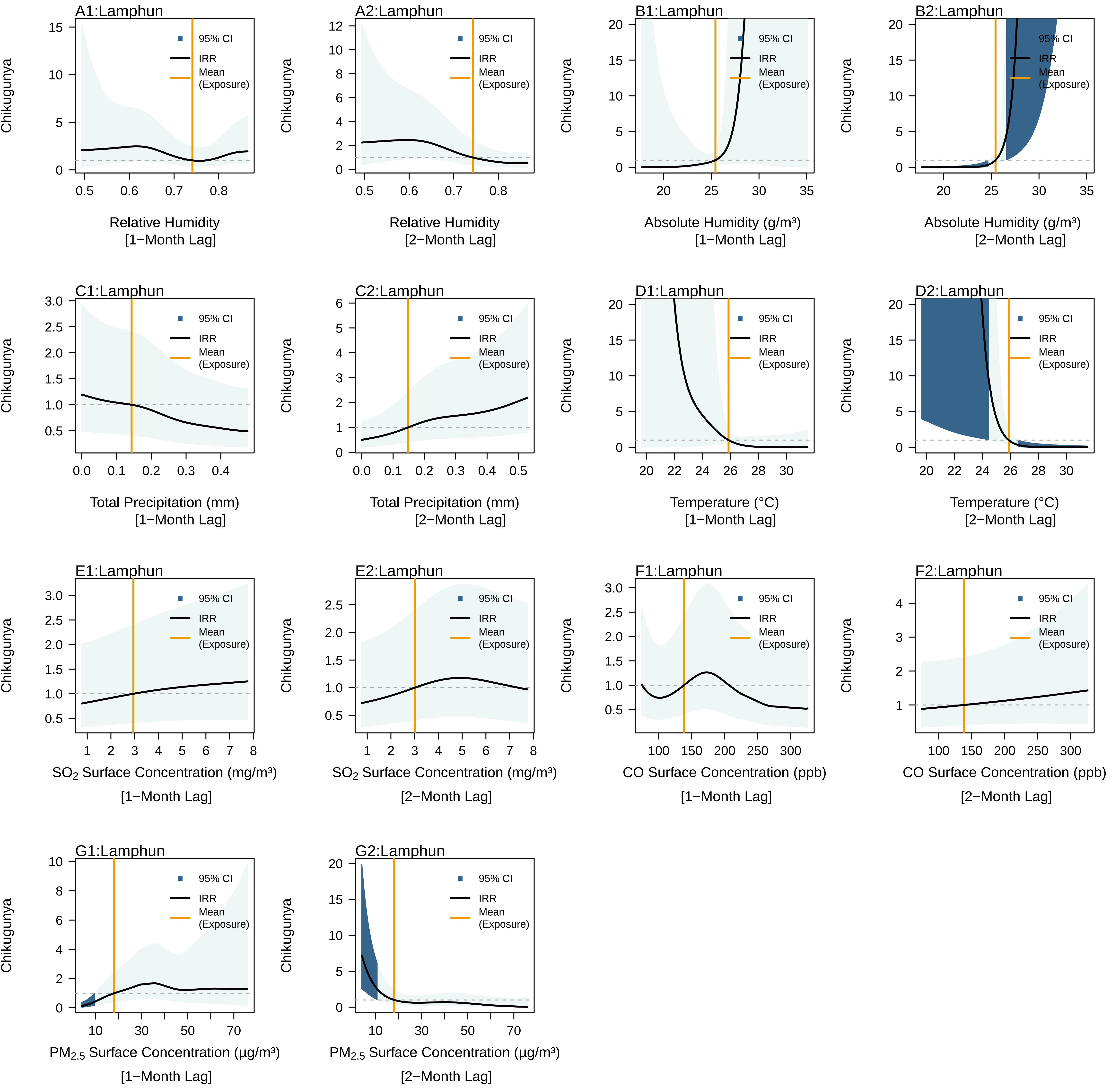
A1:Lamphun
95% CI
IRR
Mean
(Exposure)
Chikugunya
Relative Humidity
[1−Month Lag]
A2:Lamphun
Relative Humidity
[2−Month Lag]
B1:Lamphun
Absolute Humidity (g/m³)
[1−Month Lag]
B2:Lamphun
Absolute Humidity (g/m³)
[2−Month Lag]
C1:Lamphun
Total Precipitation (mm)
[1−Month Lag]
C2:Lamphun
Total Precipitation (mm)
[2−Month Lag]
D1:Lamphun
Temperature (°C)
[1−Month Lag]
D2:Lamphun
Temperature (°C)
[2−Month Lag]
E1:Lamphun
SO_2 Surface Concentration (mg/m³)
[1−Month Lag]
E2:Lamphun
SO_2 Surface Concentration (mg/m³)
[2−Month Lag]
F1:Lamphun
CO Surface Concentration (ppb)
[1−Month Lag]
F2:Lamphun
CO Surface Concentration (ppb)
[2−Month Lag]
G1:Lamphun
$PM_{2.5}$ Surface Concentration (µg/m³)
[1−Month Lag]
G2:Lamphun
$PM_{2.5}$ Surface Concentration (µg/m³)
[2−Month Lag]

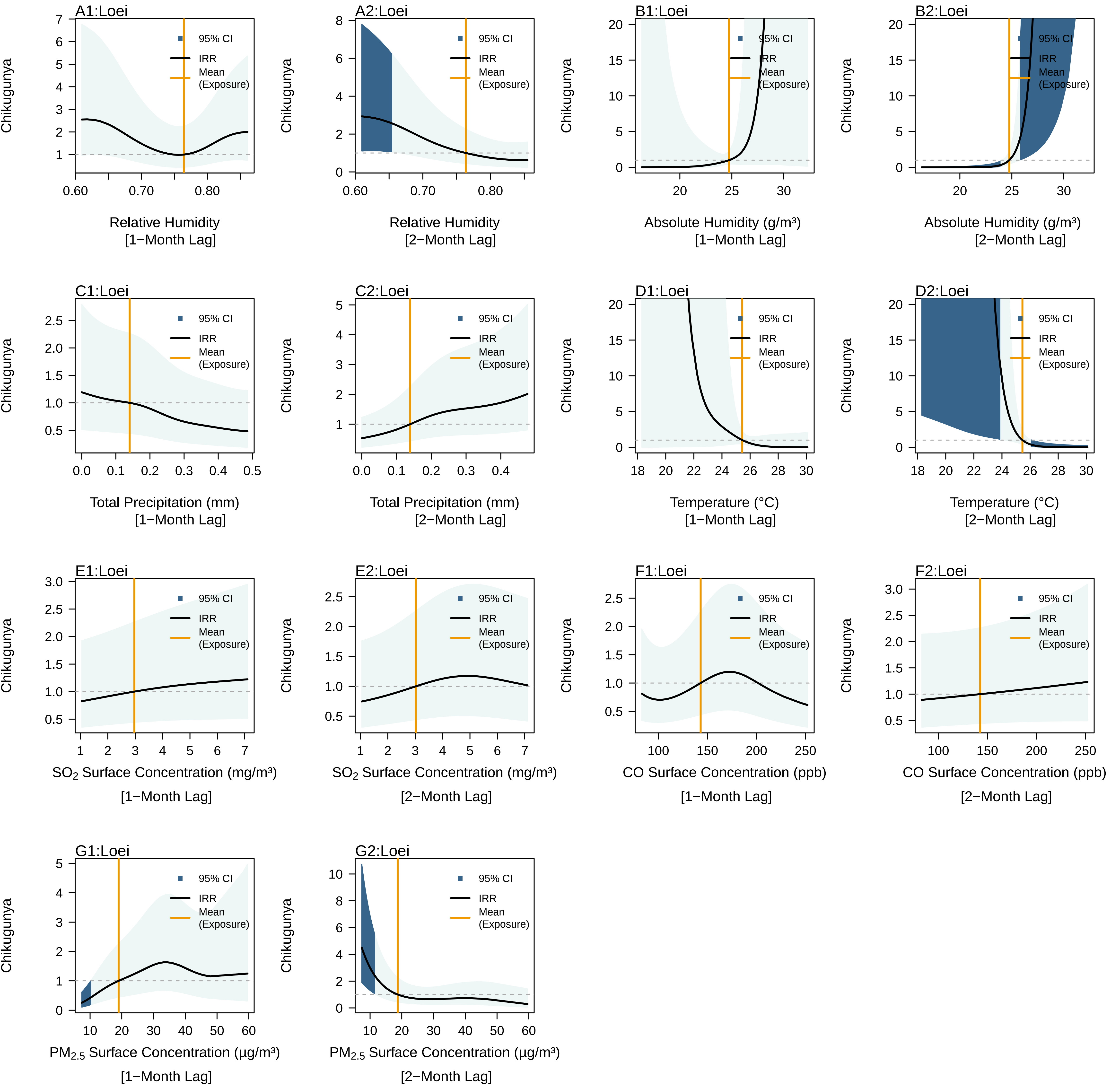
A1:Loei
A2:Loei
B1:Loei
B2:Loei
C1:Loei
C2:Loei
D1:Loei
D2:Loei
E1:Loei
E2:Loei
F1:Loei
F2:Loei
G1:Loei
G2:Loei
Chikugunya
95% CI
IRR
Mean
(Exposure)
Relative Humidity
[1−Month Lag]
Relative Humidity
[2−Month Lag]
Absolute Humidity (g/m³)
[1−Month Lag]
Absolute Humidity (g/m³)
[2−Month Lag]
Total Precipitation (mm)
[1−Month Lag]
Total Precipitation (mm)
[2−Month Lag]
Temperature (°C)
[1−Month Lag]
Temperature (°C)
[2−Month Lag]
SO_2 Surface Concentration (mg/m³)
[1−Month Lag]
SO_2 Surface Concentration (mg/m³)
[2−Month Lag]
CO Surface Concentration (ppb)
[1−Month Lag]
CO Surface Concentration (ppb)
[2−Month Lag]
$PM_{2.5}$ Surface Concentration (µg/m³)
[1−Month Lag]
$PM_{2.5}$ Surface Concentration (µg/m³)
[2−Month Lag]

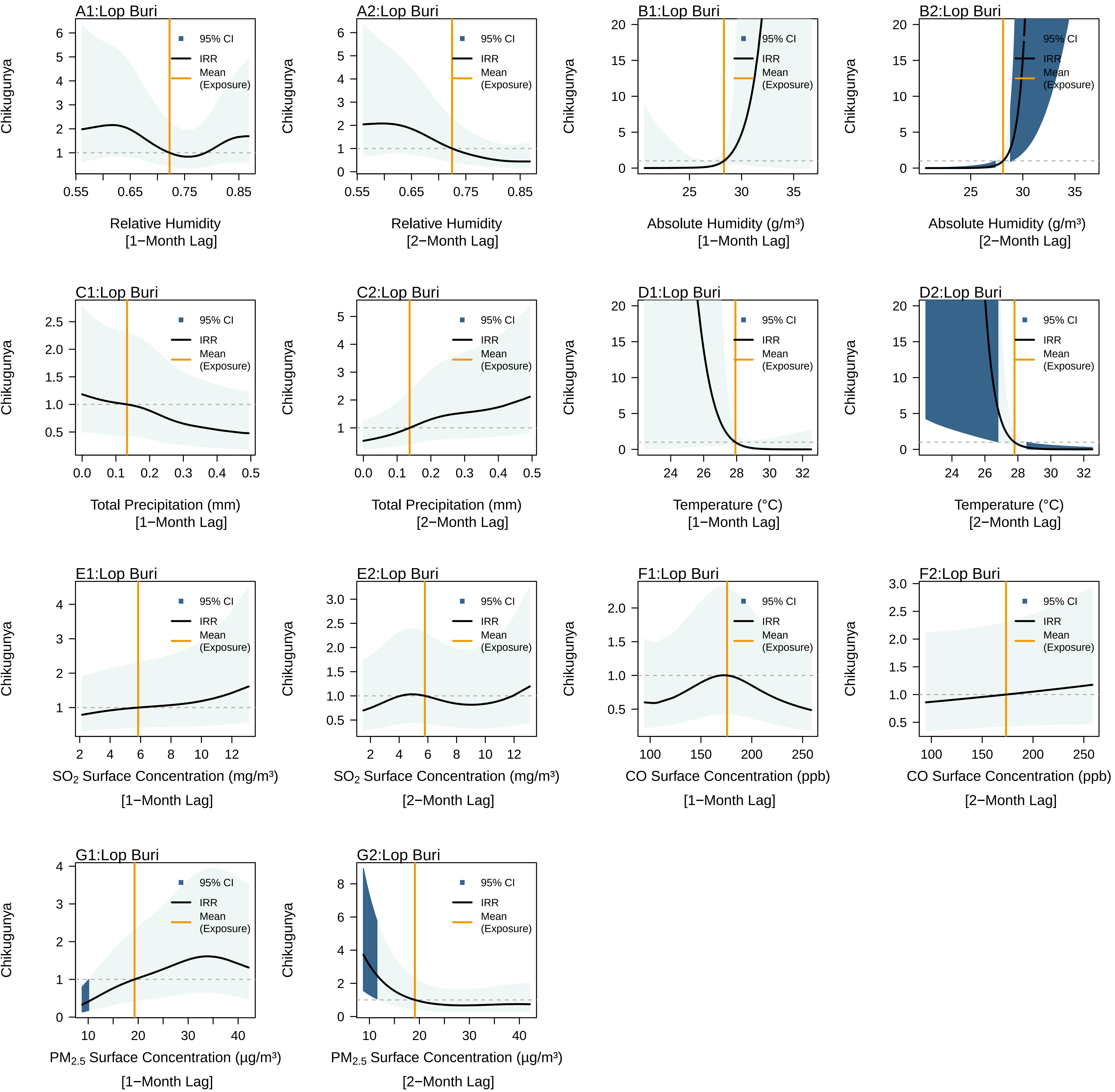
A1:Lop Buri
Chikugunya
95% CI
IRR
Mean (Exposure)
Relative Humidity [1−Month Lag]
A2:Lop Buri
Chikugunya
95% CI
IRR
Mean (Exposure)
Relative Humidity [2−Month Lag]
B1:Lop Buri
Chikugunya
95% CI
IRR
Mean (Exposure)
Absolute Humidity (g/m³) [1−Month Lag]
B2:Lop Buri
Chikugunya
95% CI
IRR
Mean (Exposure)
Absolute Humidity (g/m³) [2−Month Lag]
C1:Lop Buri
Chikugunya
95% CI
IRR
Mean (Exposure)
Total Precipitation (mm) [1−Month Lag]
C2:Lop Buri
Chikugunya
95% CI
IRR
Mean (Exposure)
Total Precipitation (mm) [2−Month Lag]
D1:Lop Buri
Chikugunya
95% CI
IRR
Mean (Exposure)
Temperature (°C) [1−Month Lag]
D2:Lop Buri
Chikugunya
95% CI
IRR
Mean (Exposure)
Temperature (°C) [2−Month Lag]
E1:Lop Buri
Chikugunya
95% CI
IRR
Mean (Exposure)
SO2 Surface Concentration (mg/m³) [1−Month Lag]
E2:Lop Buri
Chikugunya
95% CI
IRR
Mean (Exposure)
SO2 Surface Concentration (mg/m³) [2−Month Lag]
F1:Lop Buri
Chikugunya
95% CI
IRR
Mean (Exposure)
CO Surface Concentration (ppb) [1−Month Lag]
F2:Lop Buri
Chikugunya
95% CI
IRR
Mean (Exposure)
CO Surface Concentration (ppb) [2−Month Lag]
G1:Lop Buri
Chikugunya
95% CI
IRR
Mean (Exposure)
PM2.5 Surface Concentration (µg/m³) [1−Month Lag]
G2:Lop Buri
Chikugunya
95% CI
IRR
Mean (Exposure)
PM2.5 Surface Concentration (µg/m³) [2−Month Lag]

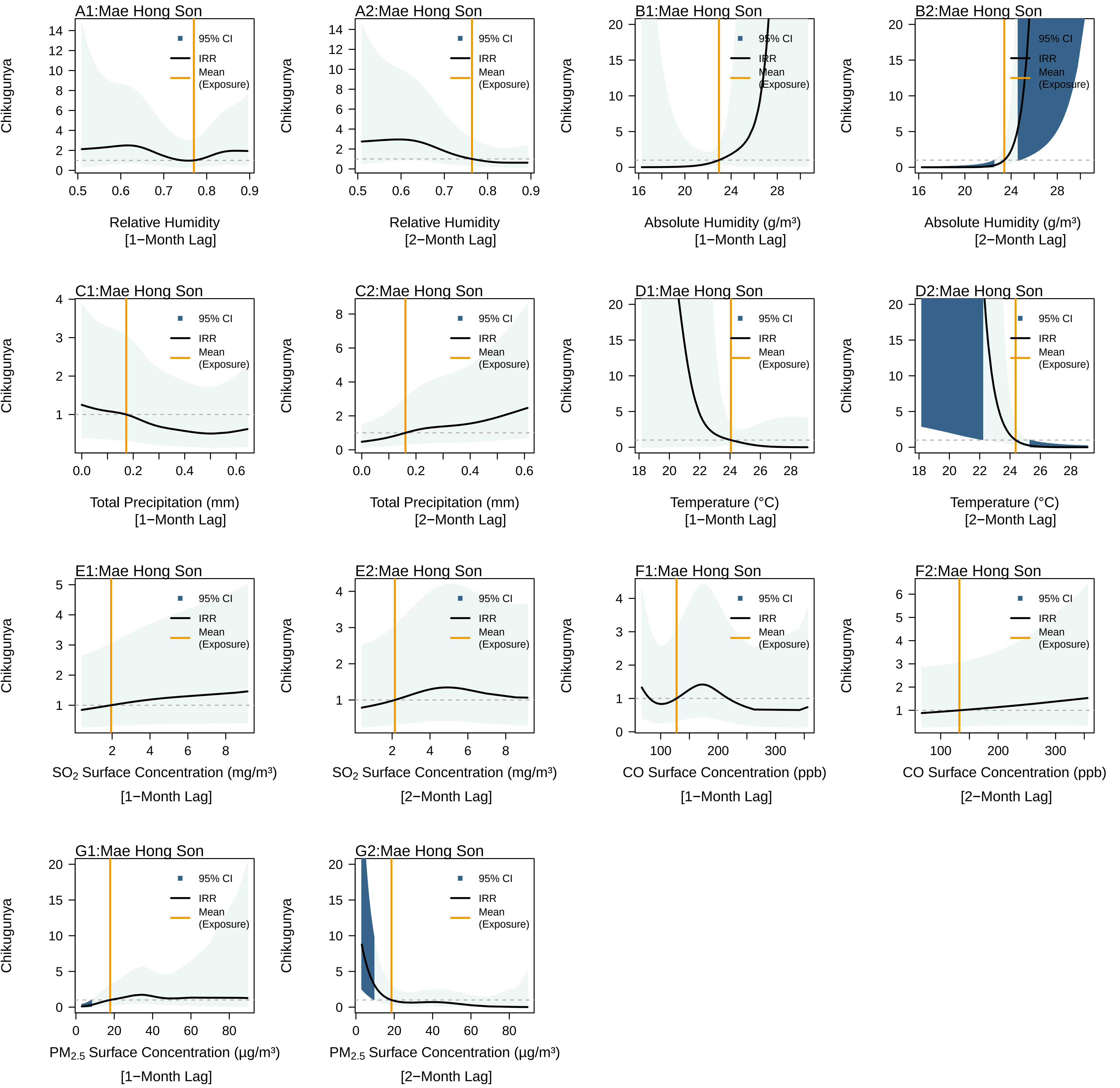
A1:Mae Hong Son
Chikugunya
95% CI
IRR
Mean (Exposure)
Relative Humidity [1−Month Lag]
A2:Mae Hong Son
Chikugunya
95% CI
IRR
Mean (Exposure)
Relative Humidity [2−Month Lag]
B1:Mae Hong Son
Chikugunya
95% CI
IRR
Mean (Exposure)
Absolute Humidity (g/m³) [1−Month Lag]
B2:Mae Hong Son
Chikugunya
95% CI
IRR
Mean (Exposure)
Absolute Humidity (g/m³) [2−Month Lag]
C1:Mae Hong Son
Chikugunya
95% CI
IRR
Mean (Exposure)
Total Precipitation (mm) [1−Month Lag]
C2:Mae Hong Son
Chikugunya
95% CI
IRR
Mean (Exposure)
Total Precipitation (mm) [2−Month Lag]
D1:Mae Hong Son
Chikugunya
95% CI
IRR
Mean (Exposure)
Temperature (°C) [1−Month Lag]
D2:Mae Hong Son
Chikugunya
95% CI
IRR
Mean (Exposure)
Temperature (°C) [2−Month Lag]
E1:Mae Hong Son
Chikugunya
95% CI
IRR
Mean (Exposure)
SO_2 Surface Concentration (mg/m³) [1−Month Lag]
E2:Mae Hong Son
Chikugunya
95% CI
IRR
Mean (Exposure)
SO_2 Surface Concentration (mg/m³) [2−Month Lag]
F1:Mae Hong Son
Chikugunya
95% CI
IRR
Mean (Exposure)
CO Surface Concentration (ppb) [1−Month Lag]
F2:Mae Hong Son
Chikugunya
95% CI
IRR
Mean (Exposure)
CO Surface Concentration (ppb) [2−Month Lag]
G1:Mae Hong Son
Chikugunya
95% CI
IRR
Mean (Exposure)
$PM_{2.5}$ Surface Concentration (μg/m³) [1−Month Lag]
G2:Mae Hong Son
Chikugunya
95% CI
IRR
Mean (Exposure)
$PM_{2.5}$ Surface Concentration (μg/m³) [2−Month Lag]

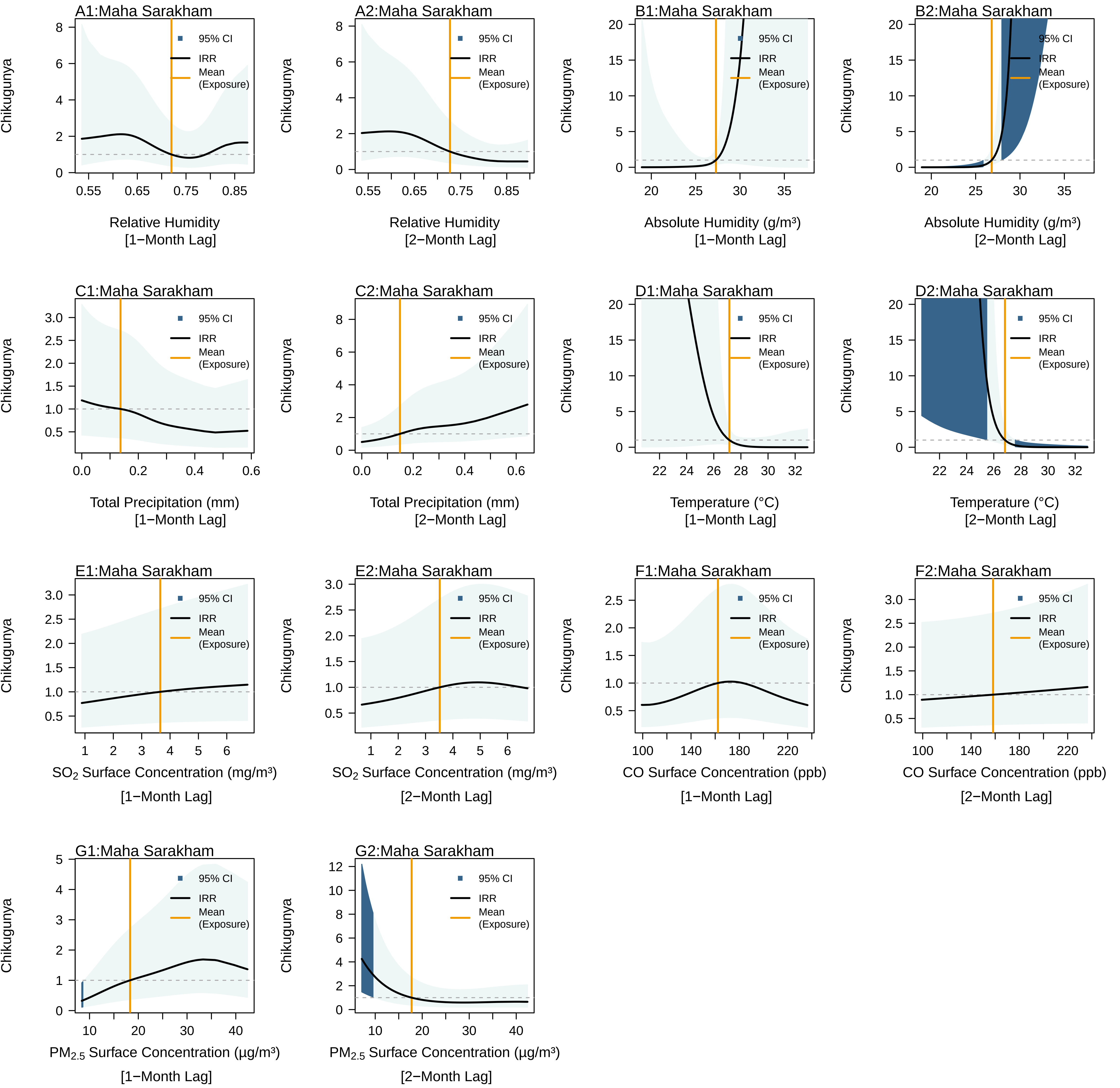
A1:Maha Sarakham
Chikugunya
95% CI
IRR
Mean (Exposure)
Relative Humidity [1−Month Lag]
A2:Maha Sarakham
Relative Humidity [2−Month Lag]
B1:Maha Sarakham
Absolute Humidity (g/m³) [1−Month Lag]
B2:Maha Sarakham
Absolute Humidity (g/m³) [2−Month Lag]
C1:Maha Sarakham
Total Precipitation (mm) [1−Month Lag]
C2:Maha Sarakham
Total Precipitation (mm) [2−Month Lag]
D1:Maha Sarakham
Temperature (°C) [1−Month Lag]
D2:Maha Sarakham
Temperature (°C) [2−Month Lag]
E1:Maha Sarakham
SO_2 Surface Concentration (mg/m³) [1−Month Lag]
E2:Maha Sarakham
SO_2 Surface Concentration (mg/m³) [2−Month Lag]
F1:Maha Sarakham
CO Surface Concentration (ppb) [1−Month Lag]
F2:Maha Sarakham
CO Surface Concentration (ppb) [2−Month Lag]
G1:Maha Sarakham
$PM_{2.5}$ Surface Concentration (μg/m³) [1−Month Lag]
G2:Maha Sarakham
$PM_{2.5}$ Surface Concentration (μg/m³) [2−Month Lag]

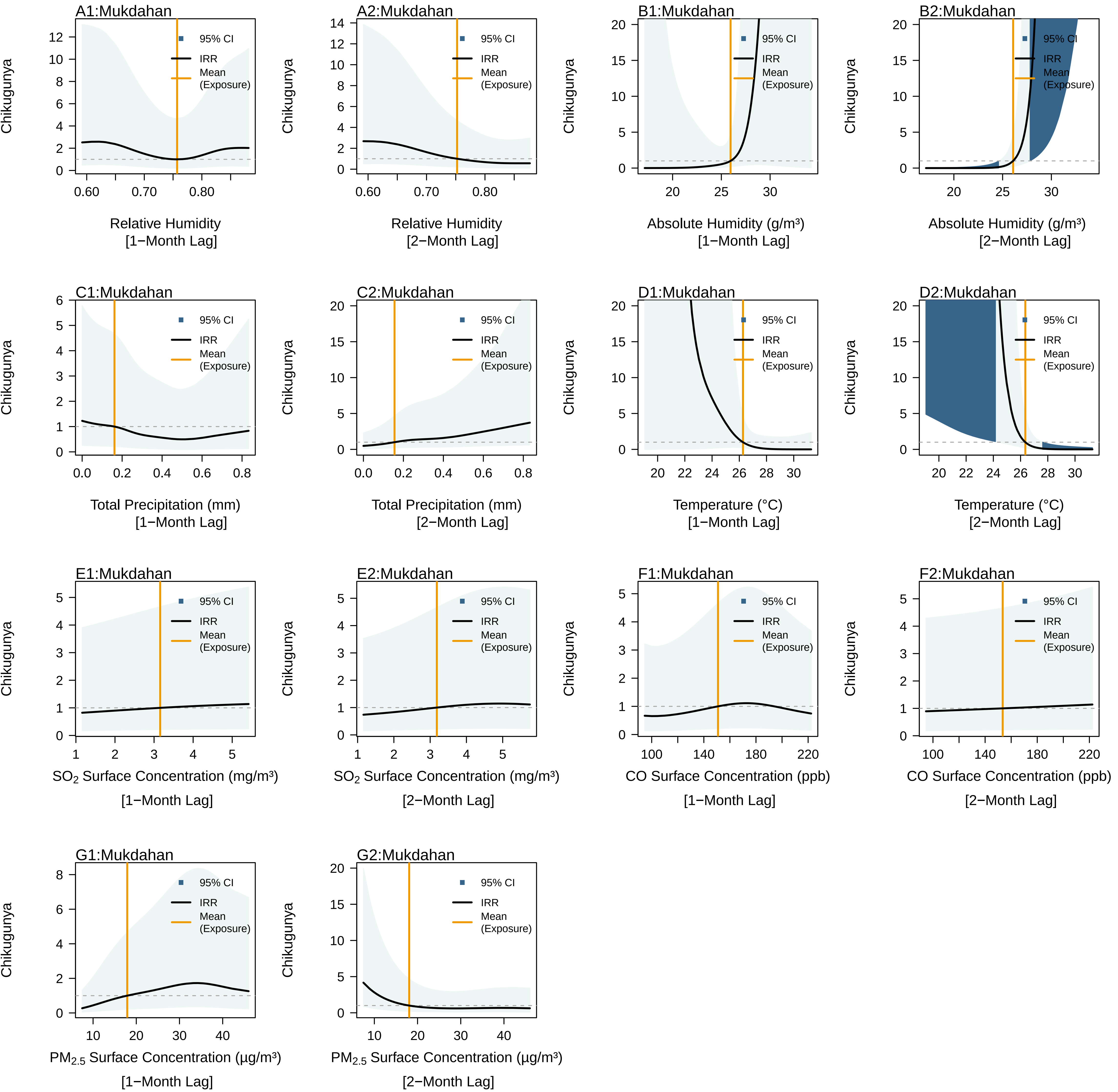

A1:Mukdahan
Chikugunya
95% CI
IRR
Mean (Exposure)
Relative Humidity [1−Month Lag]
A2:Mukdahan
Chikugunya
95% CI
IRR
Mean (Exposure)
Relative Humidity [2−Month Lag]
B1:Mukdahan
Chikugunya
95% CI
IRR
Mean (Exposure)
Absolute Humidity (g/m³) [1−Month Lag]
B2:Mukdahan
Chikugunya
95% CI
IRR
Mean (Exposure)
Absolute Humidity (g/m³) [2−Month Lag]
C1:Mukdahan
Chikugunya
95% CI
IRR
Mean (Exposure)
Total Precipitation (mm) [1−Month Lag]
C2:Mukdahan
Chikugunya
95% CI
IRR
Mean (Exposure)
Total Precipitation (mm) [2−Month Lag]
D1:Mukdahan
Chikugunya
95% CI
IRR
Mean (Exposure)
Temperature (°C) [1−Month Lag]
D2:Mukdahan
Chikugunya
95% CI
IRR
Mean (Exposure)
Temperature (°C) [2−Month Lag]
E1:Mukdahan
Chikugunya
95% CI
IRR
Mean (Exposure)
SO2 Surface Concentration (mg/m³) [1−Month Lag]
E2:Mukdahan
Chikugunya
95% CI
IRR
Mean (Exposure)
SO2 Surface Concentration (mg/m³) [2−Month Lag]
F1:Mukdahan
Chikugunya
95% CI
IRR
Mean (Exposure)
CO Surface Concentration (ppb) [1−Month Lag]
F2:Mukdahan
Chikugunya
95% CI
IRR
Mean (Exposure)
CO Surface Concentration (ppb) [2−Month Lag]
G1:Mukdahan
Chikugunya
95% CI
IRR
Mean (Exposure)
PM2.5 Surface Concentration (µg/m³) [1−Month Lag]
G2:Mukdahan
Chikugunya
95% CI
IRR
Mean (Exposure)
PM2.5 Surface Concentration (µg/m³) [2−Month Lag]

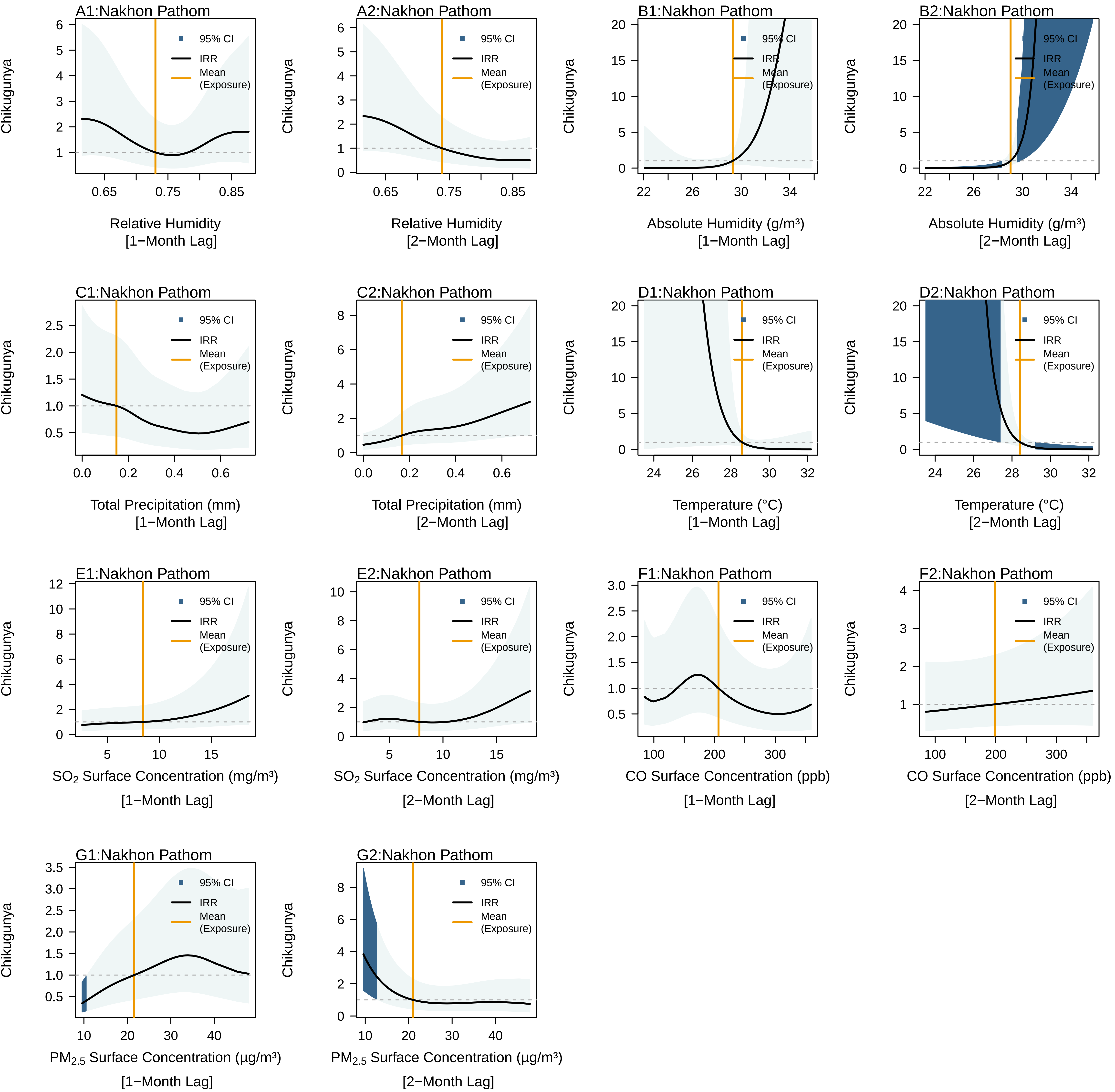
A1:Nakhon Pathom
A2:Nakhon Pathom
B1:Nakhon Pathom
B2:Nakhon Pathom
C1:Nakhon Pathom
C2:Nakhon Pathom
D1:Nakhon Pathom
D2:Nakhon Pathom
E1:Nakhon Pathom
E2:Nakhon Pathom
F1:Nakhon Pathom
F2:Nakhon Pathom
G1:Nakhon Pathom
G2:Nakhon Pathom
Chikugunya
95% CI
IRR
Mean (Exposure)
Relative Humidity [1−Month Lag]
Relative Humidity [2−Month Lag]
Absolute Humidity (g/m³) [1−Month Lag]
Absolute Humidity (g/m³) [2−Month Lag]
Total Precipitation (mm) [1−Month Lag]
Total Precipitation (mm) [2−Month Lag]
Temperature (°C) [1−Month Lag]
Temperature (°C) [2−Month Lag]
SO_2 Surface Concentration (mg/m³) [1−Month Lag]
SO_2 Surface Concentration (mg/m³) [2−Month Lag]
CO Surface Concentration (ppb) [1−Month Lag]
CO Surface Concentration (ppb) [2−Month Lag]
$PM_{2.5}$ Surface Concentration (µg/m³) [1−Month Lag]
$PM_{2.5}$ Surface Concentration (µg/m³) [2−Month Lag]

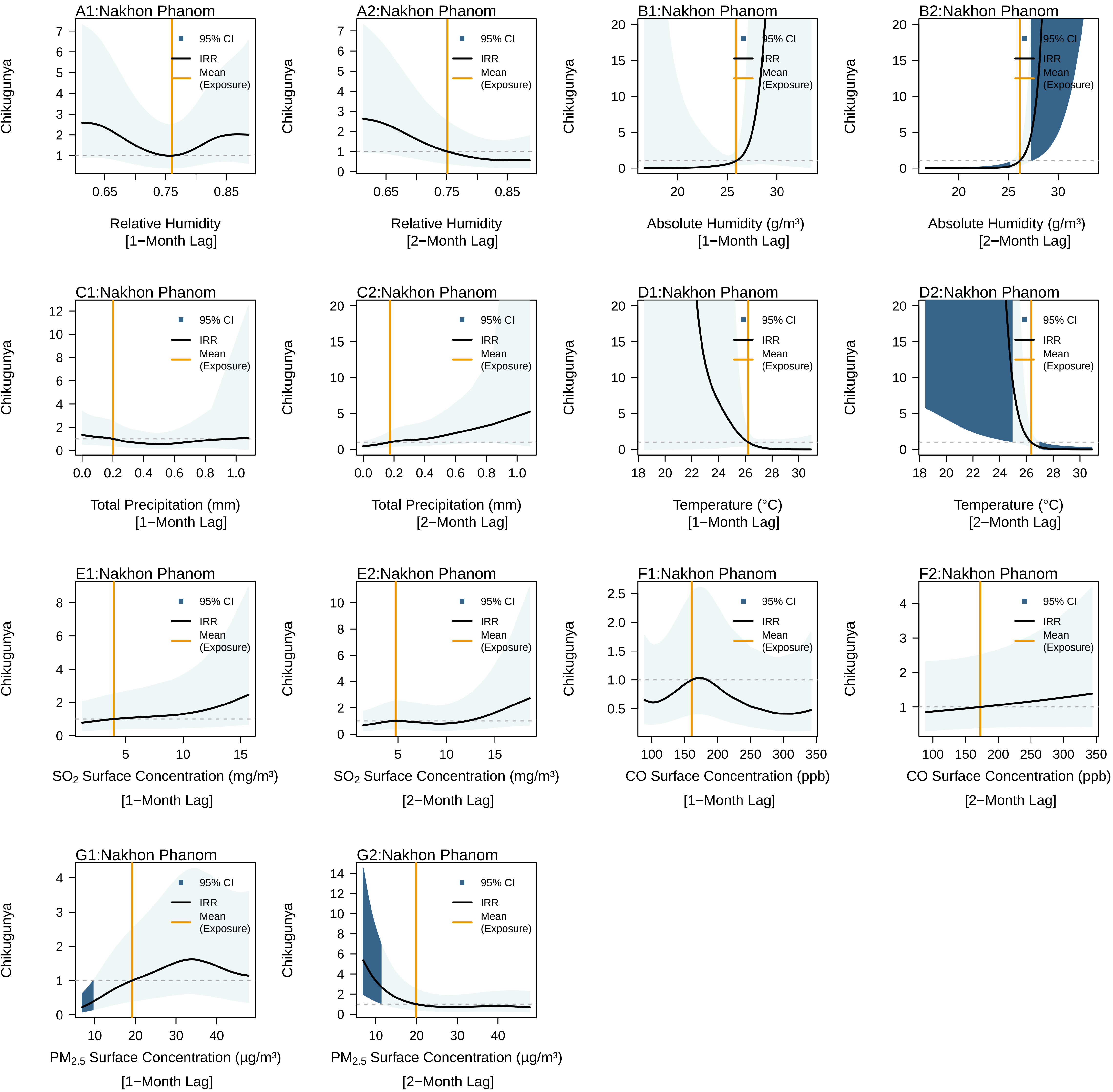
A1:Nakhon Phanom
A2:Nakhon Phanom
B1:Nakhon Phanom
B2:Nakhon Phanom
C1:Nakhon Phanom
C2:Nakhon Phanom
D1:Nakhon Phanom
D2:Nakhon Phanom
E1:Nakhon Phanom
E2:Nakhon Phanom
F1:Nakhon Phanom
F2:Nakhon Phanom
G1:Nakhon Phanom
G2:Nakhon Phanom
Chikugunya
95% CI
IRR
Mean (Exposure)
Relative Humidity [1−Month Lag]
Relative Humidity [2−Month Lag]
Absolute Humidity (g/m³) [1−Month Lag]
Absolute Humidity (g/m³) [2−Month Lag]
Total Precipitation (mm) [1−Month Lag]
Total Precipitation (mm) [2−Month Lag]
Temperature (°C) [1−Month Lag]
Temperature (°C) [2−Month Lag]
SO_2 Surface Concentration (mg/m³) [1−Month Lag]
SO_2 Surface Concentration (mg/m³) [2−Month Lag]
CO Surface Concentration (ppb) [1−Month Lag]
CO Surface Concentration (ppb) [2−Month Lag]
$PM_{2.5}$ Surface Concentration (µg/m³) [1−Month Lag]
$PM_{2.5}$ Surface Concentration (µg/m³) [2−Month Lag]

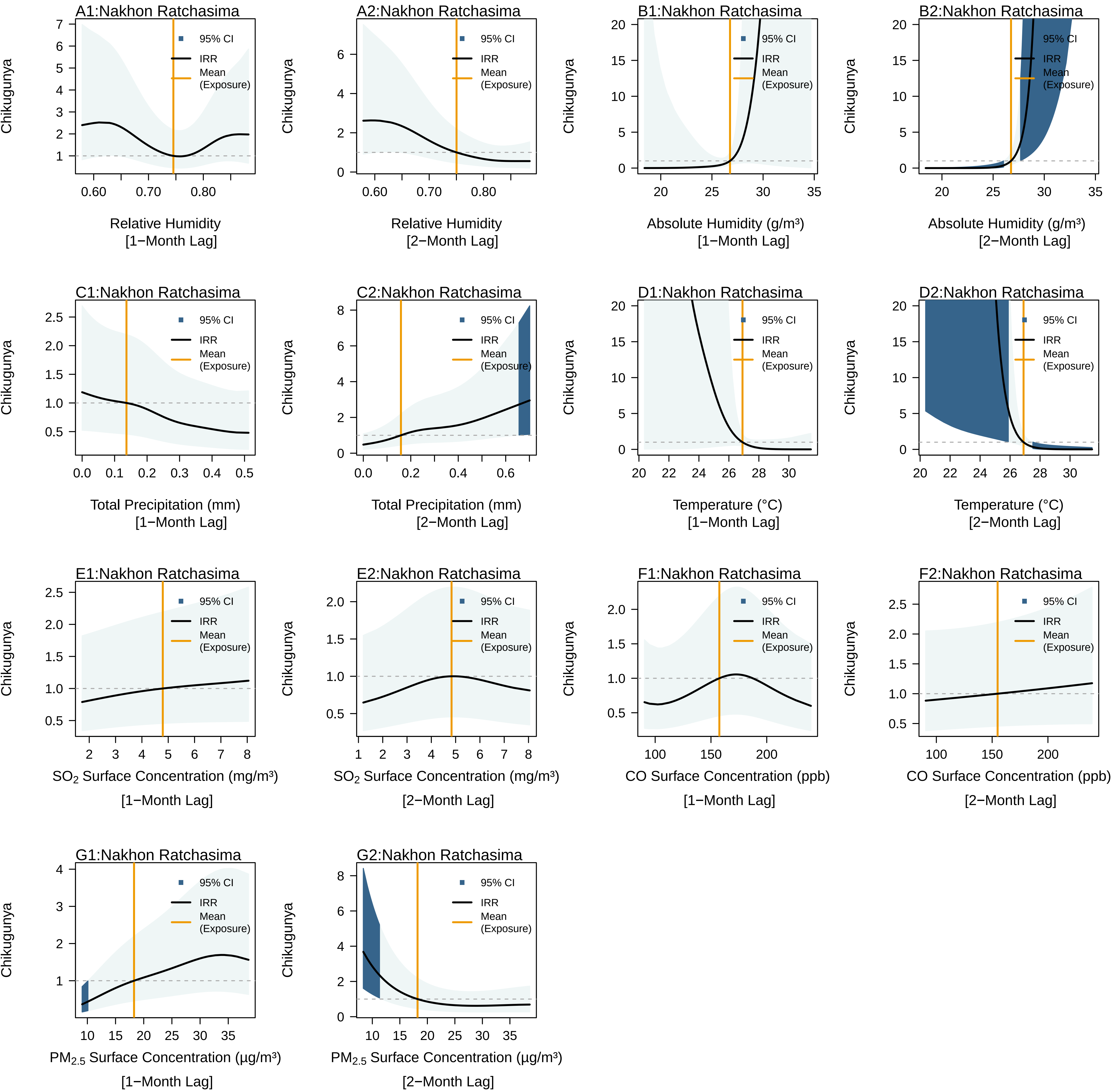
A1:Nakhon Ratchasima
A2:Nakhon Ratchasima
B1:Nakhon Ratchasima
B2:Nakhon Ratchasima
95% CI
IRR
Mean
(Exposure)
Chikugunya
Relative Humidity
[1−Month Lag]
Relative Humidity
[2−Month Lag]
Absolute Humidity (g/m³)
[1−Month Lag]
Absolute Humidity (g/m³)
[2−Month Lag]
C1:Nakhon Ratchasima
C2:Nakhon Ratchasima
D1:Nakhon Ratchasima
D2:Nakhon Ratchasima
Total Precipitation (mm)
[1−Month Lag]
Total Precipitation (mm)
[2−Month Lag]
Temperature (°C)
[1−Month Lag]
Temperature (°C)
[2−Month Lag]
E1:Nakhon Ratchasima
E2:Nakhon Ratchasima
F1:Nakhon Ratchasima
F2:Nakhon Ratchasima
SO_2 Surface Concentration (mg/m³)
[1−Month Lag]
SO_2 Surface Concentration (mg/m³)
[2−Month Lag]
CO Surface Concentration (ppb)
[1−Month Lag]
CO Surface Concentration (ppb)
[2−Month Lag]
G1:Nakhon Ratchasima
G2:Nakhon Ratchasima
$PM_{2.5}$ Surface Concentration (μg/m³)
[1−Month Lag]
$PM_{2.5}$ Surface Concentration (μg/m³)
[2−Month Lag]

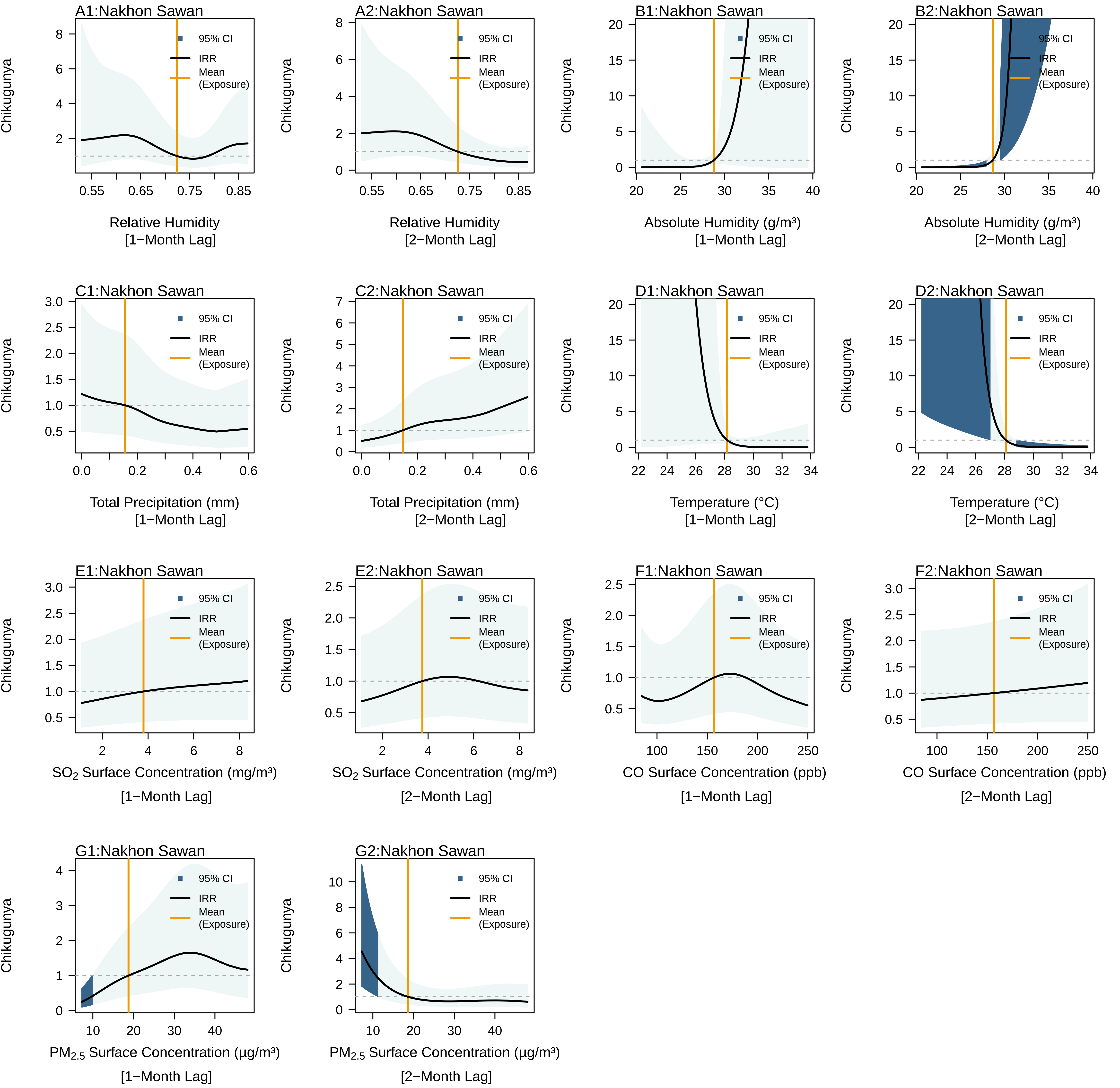

A1:Nakhon Sawan
A2:Nakhon Sawan
B1:Nakhon Sawan
B2:Nakhon Sawan
Chikugunya
95% CI
IRR
Mean (Exposure)
Relative Humidity [1−Month Lag]
Relative Humidity [2−Month Lag]
Absolute Humidity (g/m³) [1−Month Lag]
Absolute Humidity (g/m³) [2−Month Lag]
C1:Nakhon Sawan
C2:Nakhon Sawan
D1:Nakhon Sawan
D2:Nakhon Sawan
Total Precipitation (mm) [1−Month Lag]
Total Precipitation (mm) [2−Month Lag]
Temperature (°C) [1−Month Lag]
Temperature (°C) [2−Month Lag]
E1:Nakhon Sawan
E2:Nakhon Sawan
F1:Nakhon Sawan
F2:Nakhon Sawan
SO2 Surface Concentration (mg/m³) [1−Month Lag]
SO2 Surface Concentration (mg/m³) [2−Month Lag]
CO Surface Concentration (ppb) [1−Month Lag]
CO Surface Concentration (ppb) [2−Month Lag]
G1:Nakhon Sawan
G2:Nakhon Sawan
PM2.5 Surface Concentration (µg/m³) [1−Month Lag]
PM2.5 Surface Concentration (µg/m³) [2−Month Lag]

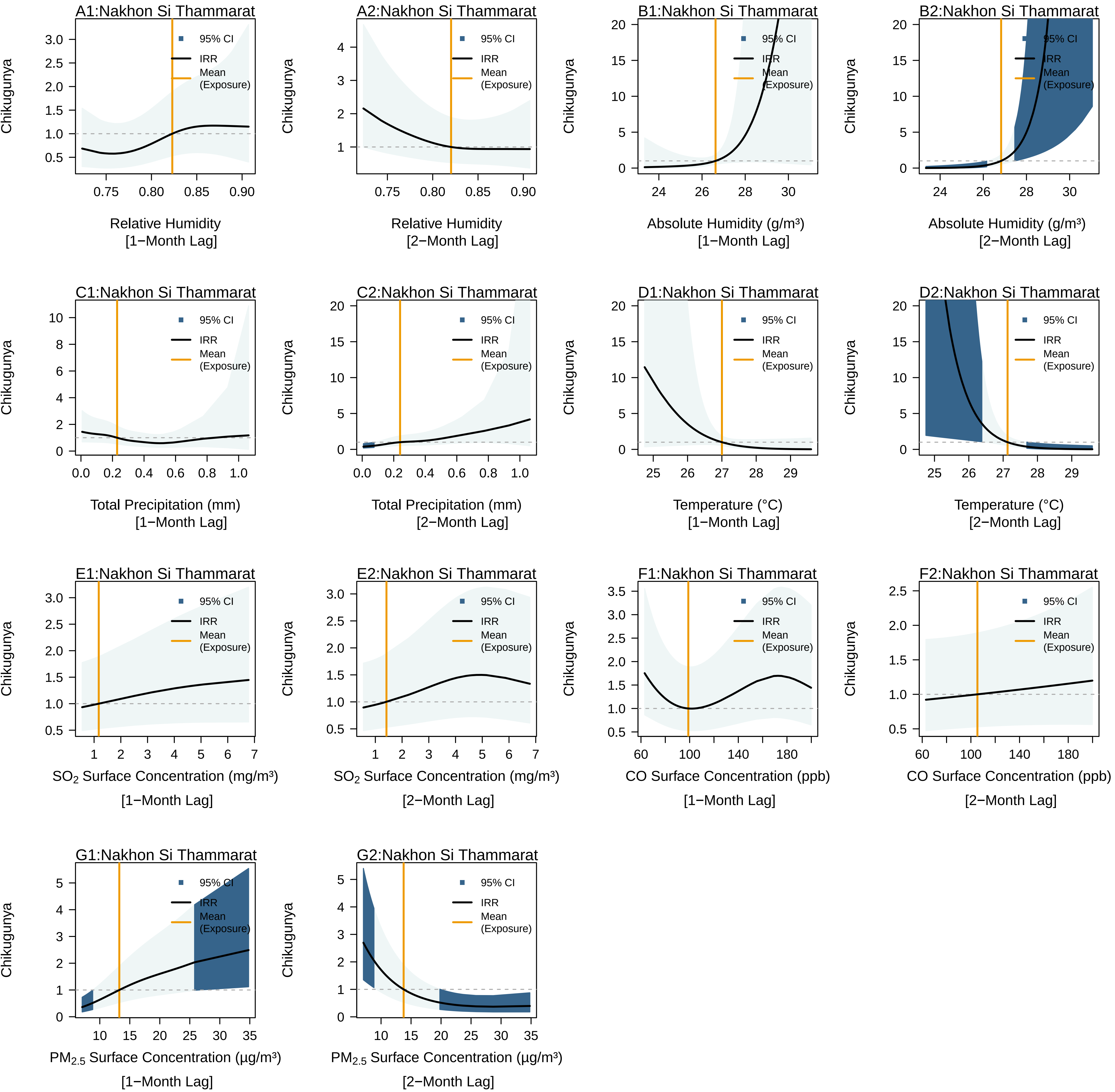
A1:Nakhon Si Thammarat
Chikugunya
95% CI
IRR
Mean (Exposure)
Relative Humidity [1−Month Lag]
A2:Nakhon Si Thammarat
Chikugunya
95% CI
IRR
Mean (Exposure)
Relative Humidity [2−Month Lag]
B1:Nakhon Si Thammarat
Chikugunya
95% CI
IRR
Mean (Exposure)
Absolute Humidity (g/m³) [1−Month Lag]
B2:Nakhon Si Thammarat
Chikugunya
95% CI
IRR
Mean (Exposure)
Absolute Humidity (g/m³) [2−Month Lag]
C1:Nakhon Si Thammarat
Chikugunya
95% CI
IRR
Mean (Exposure)
Total Precipitation (mm) [1−Month Lag]
C2:Nakhon Si Thammarat
Chikugunya
95% CI
IRR
Mean (Exposure)
Total Precipitation (mm) [2−Month Lag]
D1:Nakhon Si Thammarat
Chikugunya
95% CI
IRR
Mean (Exposure)
Temperature (°C) [1−Month Lag]
D2:Nakhon Si Thammarat
Chikugunya
95% CI
IRR
Mean (Exposure)
Temperature (°C) [2−Month Lag]
E1:Nakhon Si Thammarat
Chikugunya
95% CI
IRR
Mean (Exposure)
SO_2 Surface Concentration (mg/m³) [1−Month Lag]
E2:Nakhon Si Thammarat
Chikugunya
95% CI
IRR
Mean (Exposure)
SO_2 Surface Concentration (mg/m³) [2−Month Lag]
F1:Nakhon Si Thammarat
Chikugunya
95% CI
IRR
Mean (Exposure)
CO Surface Concentration (ppb) [1−Month Lag]
F2:Nakhon Si Thammarat
Chikugunya
95% CI
IRR
Mean (Exposure)
CO Surface Concentration (ppb) [2−Month Lag]
G1:Nakhon Si Thammarat
Chikugunya
95% CI
IRR
Mean (Exposure)
$PM_{2.5}$ Surface Concentration (μg/m³) [1−Month Lag]
G2:Nakhon Si Thammarat
Chikugunya
95% CI
IRR
Mean (Exposure)
$PM_{2.5}$ Surface Concentration (μg/m³) [2−Month Lag]

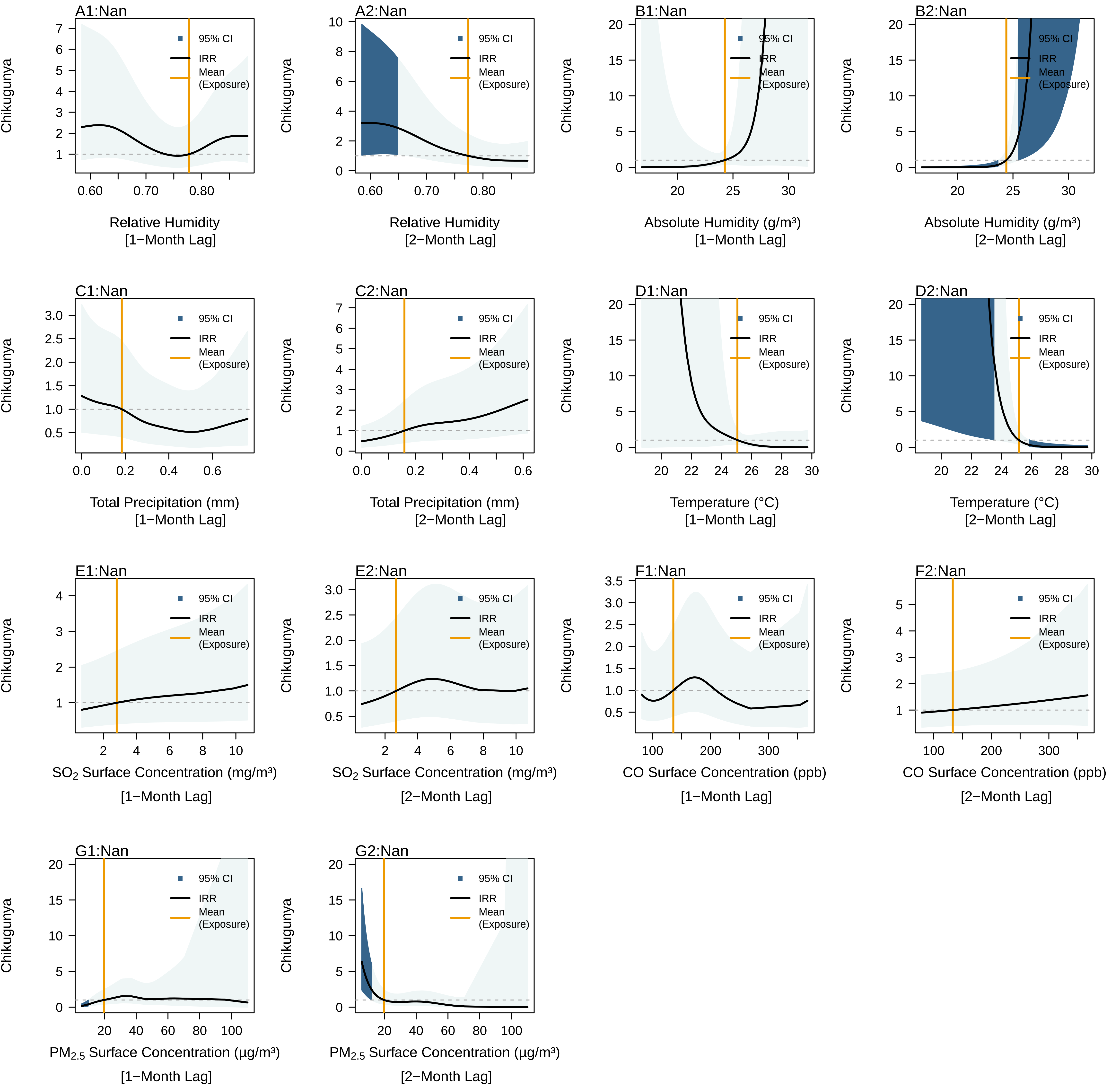
A1:Nan
Chikugunya
95% CI
IRR
Mean (Exposure)
Relative Humidity [1−Month Lag]
A2:Nan
Relative Humidity [2−Month Lag]
B1:Nan
Absolute Humidity (g/m³) [1−Month Lag]
B2:Nan
Absolute Humidity (g/m³) [2−Month Lag]
C1:Nan
Total Precipitation (mm) [1−Month Lag]
C2:Nan
Total Precipitation (mm) [2−Month Lag]
D1:Nan
Temperature (°C) [1−Month Lag]
D2:Nan
Temperature (°C) [2−Month Lag]
E1:Nan
SO2 Surface Concentration (mg/m³) [1−Month Lag]
E2:Nan
SO2 Surface Concentration (mg/m³) [2−Month Lag]
F1:Nan
CO Surface Concentration (ppb) [1−Month Lag]
F2:Nan
CO Surface Concentration (ppb) [2−Month Lag]
G1:Nan
PM2.5 Surface Concentration (μg/m³) [1−Month Lag]
G2:Nan
PM2.5 Surface Concentration (μg/m³) [2−Month Lag]

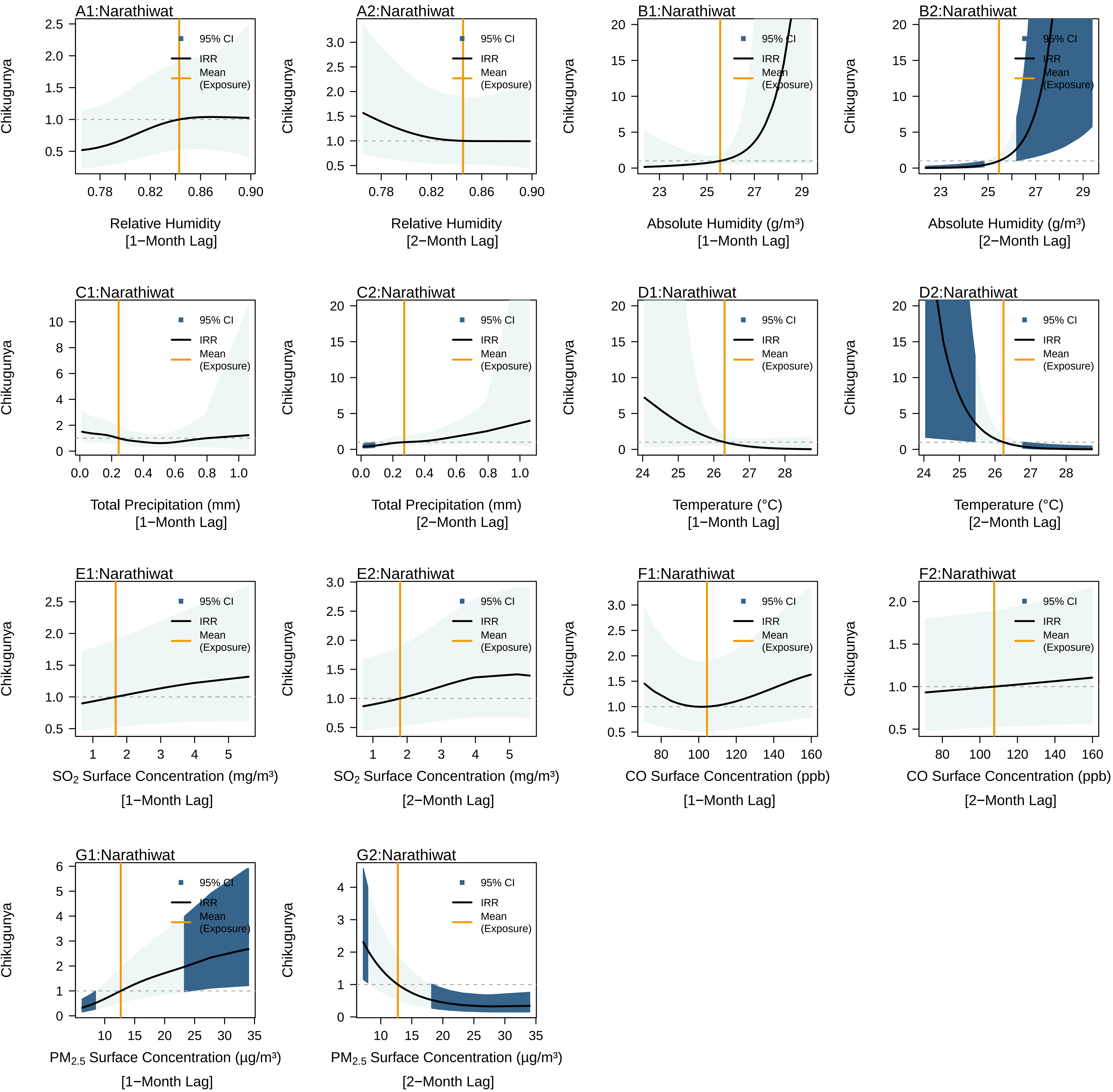
A1:Narathiwat
Chikugunya
95% CI
IRR
Mean (Exposure)
Relative Humidity [1−Month Lag]
A2:Narathiwat
Chikugunya
95% CI
IRR
Mean (Exposure)
Relative Humidity [2−Month Lag]
B1:Narathiwat
Chikugunya
95% CI
IRR
Mean (Exposure)
Absolute Humidity (g/m³) [1−Month Lag]
B2:Narathiwat
Chikugunya
95% CI
IRR
Mean (Exposure)
Absolute Humidity (g/m³) [2−Month Lag]
C1:Narathiwat
Chikugunya
95% CI
IRR
Mean (Exposure)
Total Precipitation (mm) [1−Month Lag]
C2:Narathiwat
Chikugunya
95% CI
IRR
Mean (Exposure)
Total Precipitation (mm) [2−Month Lag]
D1:Narathiwat
Chikugunya
95% CI
IRR
Mean (Exposure)
Temperature (°C) [1−Month Lag]
D2:Narathiwat
Chikugunya
95% CI
IRR
Mean (Exposure)
Temperature (°C) [2−Month Lag]
E1:Narathiwat
Chikugunya
95% CI
IRR
Mean (Exposure)
SO2 Surface Concentration (mg/m³) [1−Month Lag]
E2:Narathiwat
Chikugunya
95% CI
IRR
Mean (Exposure)
SO2 Surface Concentration (mg/m³) [2−Month Lag]
F1:Narathiwat
Chikugunya
95% CI
IRR
Mean (Exposure)
CO Surface Concentration (ppb) [1−Month Lag]
F2:Narathiwat
Chikugunya
95% CI
IRR
Mean (Exposure)
CO Surface Concentration (ppb) [2−Month Lag]
G1:Narathiwat
Chikugunya
95% CI
IRR
Mean (Exposure)
PM2.5 Surface Concentration (µg/m³) [1−Month Lag]
G2:Narathiwat
Chikugunya
95% CI
IRR
Mean (Exposure)
PM2.5 Surface Concentration (µg/m³) [2−Month Lag]

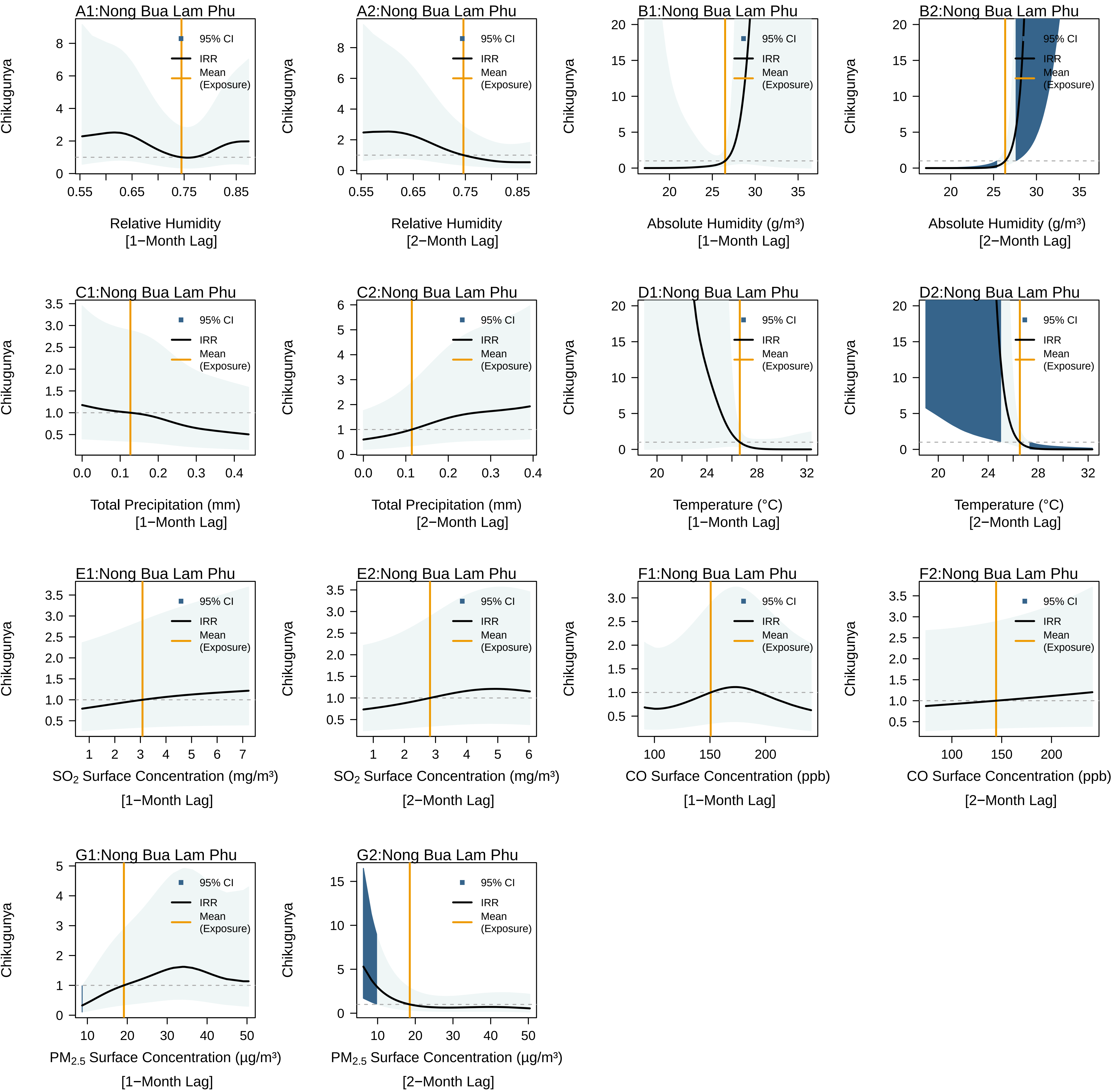
A1:Nong Bua Lam Phu
A2:Nong Bua Lam Phu
B1:Nong Bua Lam Phu
B2:Nong Bua Lam Phu
C1:Nong Bua Lam Phu
C2:Nong Bua Lam Phu
D1:Nong Bua Lam Phu
D2:Nong Bua Lam Phu
E1:Nong Bua Lam Phu
E2:Nong Bua Lam Phu
F1:Nong Bua Lam Phu
F2:Nong Bua Lam Phu
G1:Nong Bua Lam Phu
G2:Nong Bua Lam Phu
Chikugunya
95% CI
IRR
Mean (Exposure)
Relative Humidity [1−Month Lag]
Relative Humidity [2−Month Lag]
Absolute Humidity (g/m³) [1−Month Lag]
Absolute Humidity (g/m³) [2−Month Lag]
Total Precipitation (mm) [1−Month Lag]
Total Precipitation (mm) [2−Month Lag]
Temperature (°C) [1−Month Lag]
Temperature (°C) [2−Month Lag]
SO2 Surface Concentration (mg/m³) [1−Month Lag]
SO2 Surface Concentration (mg/m³) [2−Month Lag]
CO Surface Concentration (ppb) [1−Month Lag]
CO Surface Concentration (ppb) [2−Month Lag]
PM2.5 Surface Concentration (µg/m³) [1−Month Lag]
PM2.5 Surface Concentration (µg/m³) [2−Month Lag]

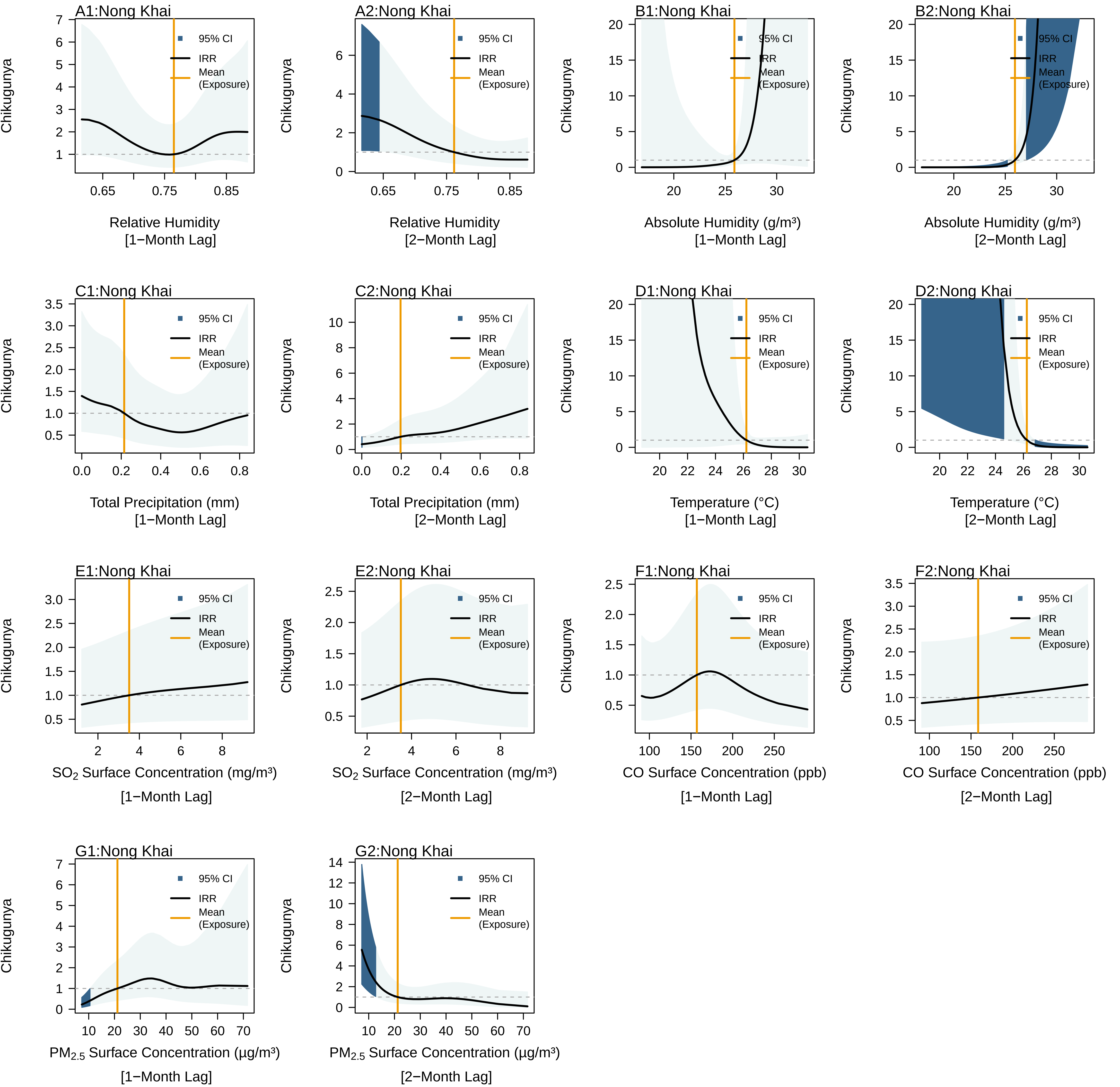

A1:Nong Khai
Chikugunya
95% CI
IRR
Mean (Exposure)
Relative Humidity [1−Month Lag]
A2:Nong Khai
Chikugunya
95% CI
IRR
Mean (Exposure)
Relative Humidity [2−Month Lag]
B1:Nong Khai
Chikugunya
95% CI
IRR
Mean (Exposure)
Absolute Humidity (g/m³) [1−Month Lag]
B2:Nong Khai
Chikugunya
95% CI
IRR
Mean (Exposure)
Absolute Humidity (g/m³) [2−Month Lag]
C1:Nong Khai
Chikugunya
95% CI
IRR
Mean (Exposure)
Total Precipitation (mm) [1−Month Lag]
C2:Nong Khai
Chikugunya
95% CI
IRR
Mean (Exposure)
Total Precipitation (mm) [2−Month Lag]
D1:Nong Khai
Chikugunya
95% CI
IRR
Mean (Exposure)
Temperature (°C) [1−Month Lag]
D2:Nong Khai
Chikugunya
95% CI
IRR
Mean (Exposure)
Temperature (°C) [2−Month Lag]
E1:Nong Khai
Chikugunya
95% CI
IRR
Mean (Exposure)
SO_2 Surface Concentration (mg/m³) [1−Month Lag]
E2:Nong Khai
Chikugunya
95% CI
IRR
Mean (Exposure)
SO_2 Surface Concentration (mg/m³) [2−Month Lag]
F1:Nong Khai
Chikugunya
95% CI
IRR
Mean (Exposure)
CO Surface Concentration (ppb) [1−Month Lag]
F2:Nong Khai
Chikugunya
95% CI
IRR
Mean (Exposure)
CO Surface Concentration (ppb) [2−Month Lag]
G1:Nong Khai
Chikugunya
95% CI
IRR
Mean (Exposure)
$PM_{2.5}$ Surface Concentration (µg/m³) [1−Month Lag]
G2:Nong Khai
Chikugunya
95% CI
IRR
Mean (Exposure)
$PM_{2.5}$ Surface Concentration (µg/m³) [2−Month Lag]

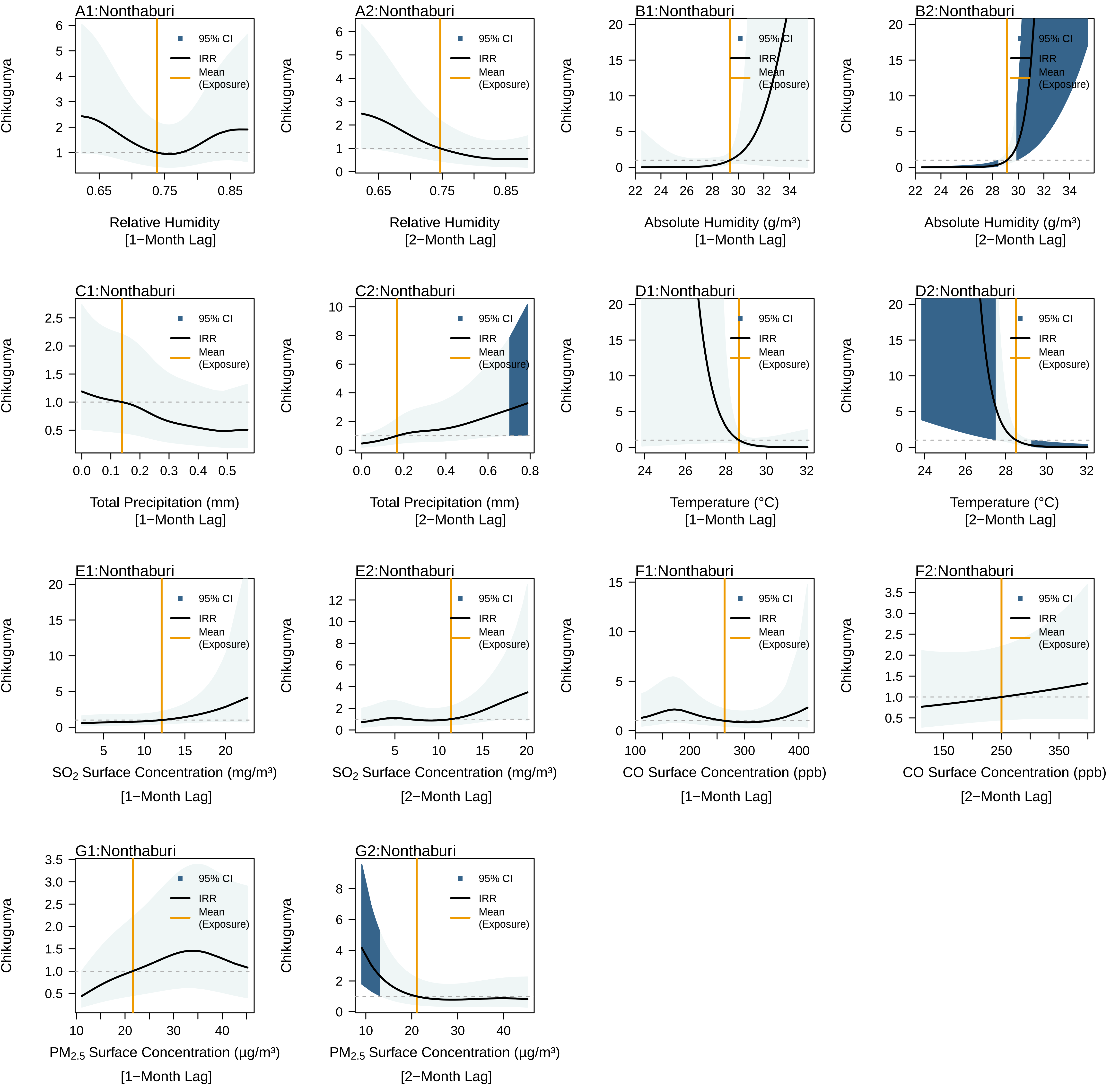
A1:Nonthaburi
Chikugunya
95% CI
IRR
Mean (Exposure)
Relative Humidity [1−Month Lag]
A2:Nonthaburi
Chikugunya
Relative Humidity [2−Month Lag]
B1:Nonthaburi
Absolute Humidity (g/m³) [1−Month Lag]
B2:Nonthaburi
Absolute Humidity (g/m³) [2−Month Lag]
C1:Nonthaburi
Total Precipitation (mm) [1−Month Lag]
C2:Nonthaburi
Total Precipitation (mm) [2−Month Lag]
D1:Nonthaburi
Temperature (°C) [1−Month Lag]
D2:Nonthaburi
Temperature (°C) [2−Month Lag]
E1:Nonthaburi
SO2 Surface Concentration (mg/m³) [1−Month Lag]
E2:Nonthaburi
SO2 Surface Concentration (mg/m³) [2−Month Lag]
F1:Nonthaburi
CO Surface Concentration (ppb) [1−Month Lag]
F2:Nonthaburi
CO Surface Concentration (ppb) [2−Month Lag]
G1:Nonthaburi
PM2.5 Surface Concentration (µg/m³) [1−Month Lag]
G2:Nonthaburi
PM2.5 Surface Concentration (µg/m³) [2−Month Lag]

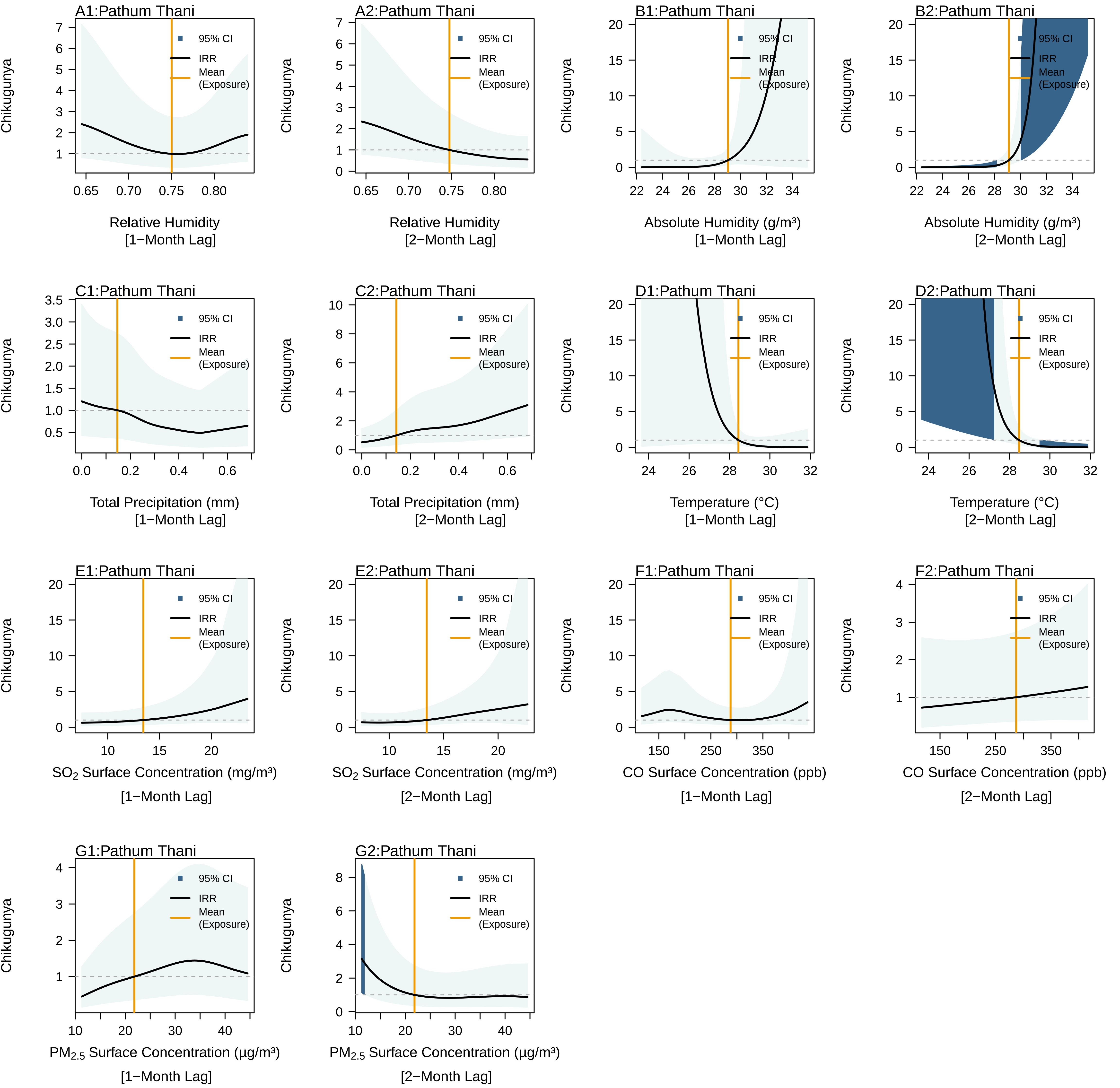

A1:Pathum Thani
Chikugunya
95% CI
IRR
Mean (Exposure)
Relative Humidity [1−Month Lag]
A2:Pathum Thani
Chikugunya
95% CI
IRR
Mean (Exposure)
Relative Humidity [2−Month Lag]
B1:Pathum Thani
Chikugunya
95% CI
IRR
Mean (Exposure)
Absolute Humidity (g/m³) [1−Month Lag]
B2:Pathum Thani
Chikugunya
95% CI
IRR
Mean (Exposure)
Absolute Humidity (g/m³) [2−Month Lag]
C1:Pathum Thani
Chikugunya
95% CI
IRR
Mean (Exposure)
Total Precipitation (mm) [1−Month Lag]
C2:Pathum Thani
Chikugunya
95% CI
IRR
Mean (Exposure)
Total Precipitation (mm) [2−Month Lag]
D1:Pathum Thani
Chikugunya
95% CI
IRR
Mean (Exposure)
Temperature (°C) [1−Month Lag]
D2:Pathum Thani
Chikugunya
95% CI
IRR
Mean (Exposure)
Temperature (°C) [2−Month Lag]
E1:Pathum Thani
Chikugunya
95% CI
IRR
Mean (Exposure)
SO_2 Surface Concentration (mg/m³) [1−Month Lag]
E2:Pathum Thani
Chikugunya
95% CI
IRR
Mean (Exposure)
SO_2 Surface Concentration (mg/m³) [2−Month Lag]
F1:Pathum Thani
Chikugunya
95% CI
IRR
Mean (Exposure)
CO Surface Concentration (ppb) [1−Month Lag]
F2:Pathum Thani
Chikugunya
95% CI
IRR
Mean (Exposure)
CO Surface Concentration (ppb) [2−Month Lag]
G1:Pathum Thani
Chikugunya
95% CI
IRR
Mean (Exposure)
$PM_{2.5}$ Surface Concentration (µg/m³) [1−Month Lag]
G2:Pathum Thani
Chikugunya
95% CI
IRR
Mean (Exposure)
$PM_{2.5}$ Surface Concentration (µg/m³) [2−Month Lag]

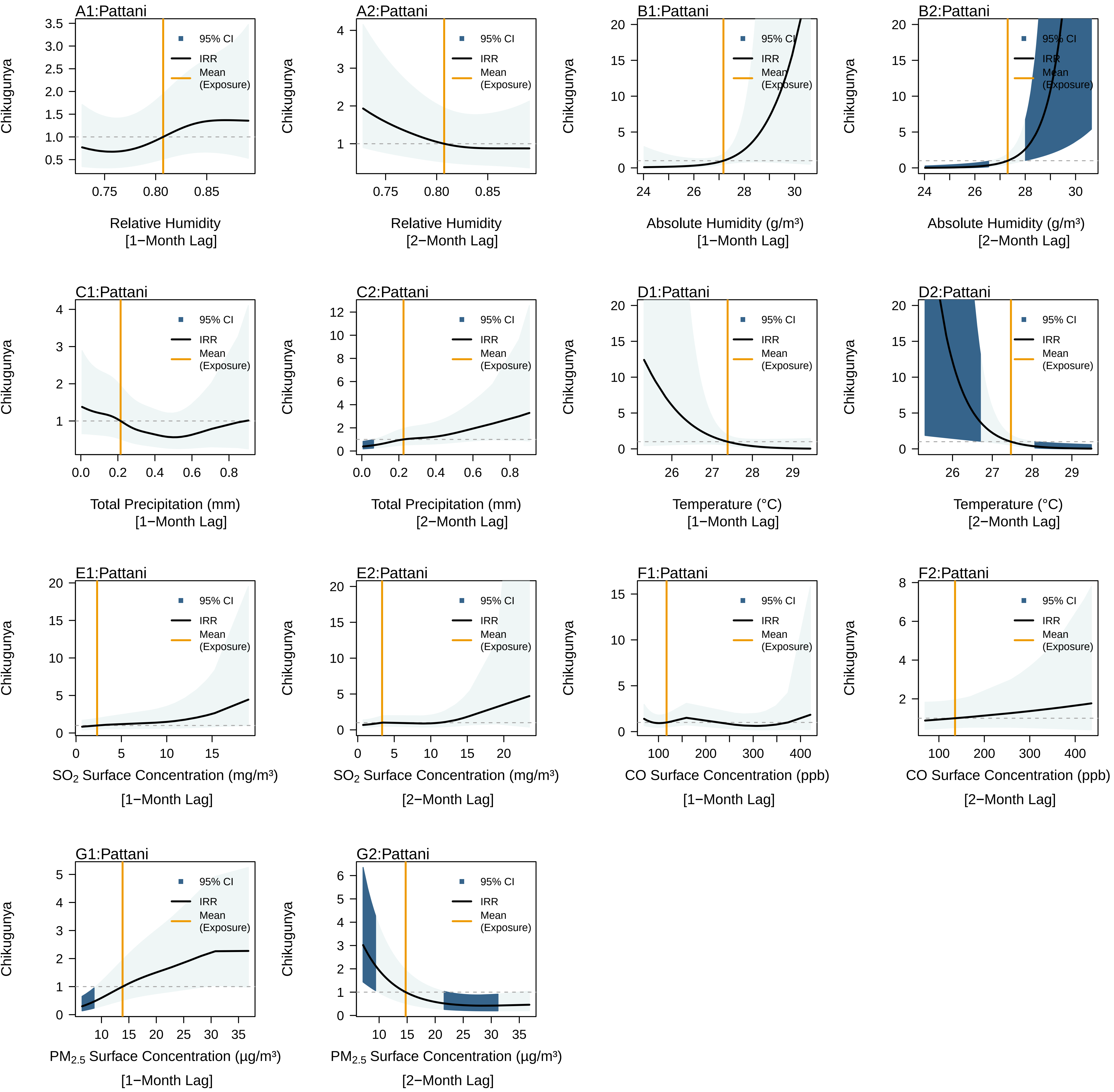

A1:Pattani
A2:Pattani
B1:Pattani
B2:Pattani
Chikugunya
95% CI
IRR
Mean (Exposure)
Relative Humidity [1−Month Lag]
Relative Humidity [2−Month Lag]
Absolute Humidity (g/m³) [1−Month Lag]
Absolute Humidity (g/m³) [2−Month Lag]
C1:Pattani
C2:Pattani
D1:Pattani
D2:Pattani
Total Precipitation (mm) [1−Month Lag]
Total Precipitation (mm) [2−Month Lag]
Temperature (°C) [1−Month Lag]
Temperature (°C) [2−Month Lag]
E1:Pattani
E2:Pattani
F1:Pattani
F2:Pattani
SO_2 Surface Concentration (mg/m³) [1−Month Lag]
SO_2 Surface Concentration (mg/m³) [2−Month Lag]
CO Surface Concentration (ppb) [1−Month Lag]
CO Surface Concentration (ppb) [2−Month Lag]
G1:Pattani
G2:Pattani
$PM_{2.5}$ Surface Concentration (µg/m³) [1−Month Lag]
$PM_{2.5}$ Surface Concentration (µg/m³) [2−Month Lag]

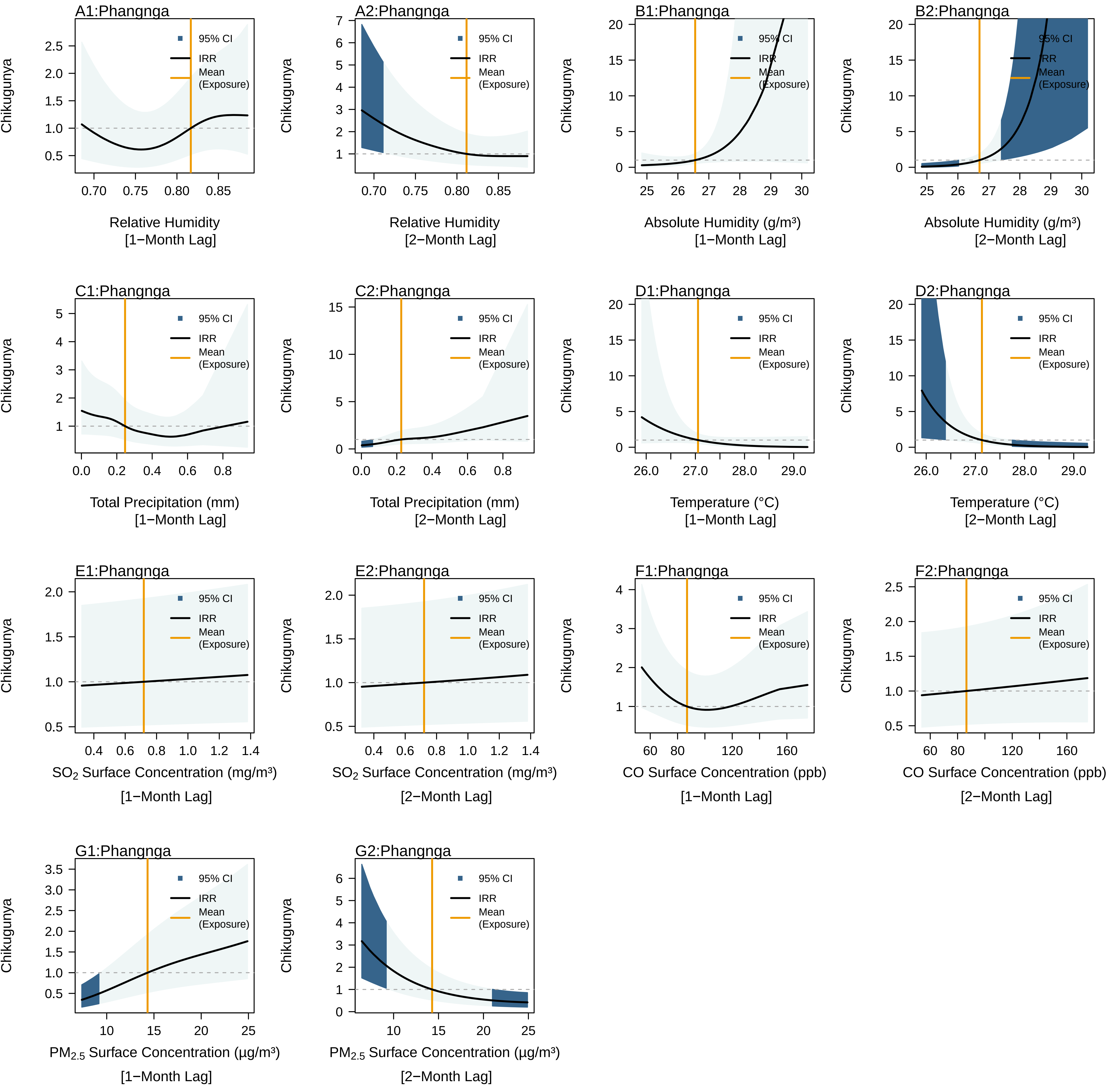
A1:Phangnga
Chikugunya
95% CI
IRR
Mean
(Exposure)
Relative Humidity
[1−Month Lag]
A2:Phangnga
Chikugunya
Relative Humidity
[2−Month Lag]
B1:Phangnga
Chikugunya
Absolute Humidity (g/m³)
[1−Month Lag]
B2:Phangnga
Chikugunya
Absolute Humidity (g/m³)
[2−Month Lag]
C1:Phangnga
Chikugunya
Total Precipitation (mm)
[1−Month Lag]
C2:Phangnga
Chikugunya
Total Precipitation (mm)
[2−Month Lag]
D1:Phangnga
Chikugunya
Temperature (°C)
[1−Month Lag]
D2:Phangnga
Chikugunya
Temperature (°C)
[2−Month Lag]
E1:Phangnga
Chikugunya
SO2 Surface Concentration (mg/m³)
[1−Month Lag]
E2:Phangnga
Chikugunya
SO2 Surface Concentration (mg/m³)
[2−Month Lag]
F1:Phangnga
Chikugunya
CO Surface Concentration (ppb)
[1−Month Lag]
F2:Phangnga
Chikugunya
CO Surface Concentration (ppb)
[2−Month Lag]
G1:Phangnga
Chikugunya
PM2.5 Surface Concentration (µg/m³)
[1−Month Lag]
G2:Phangnga
Chikugunya
PM2.5 Surface Concentration (µg/m³)
[2−Month Lag]

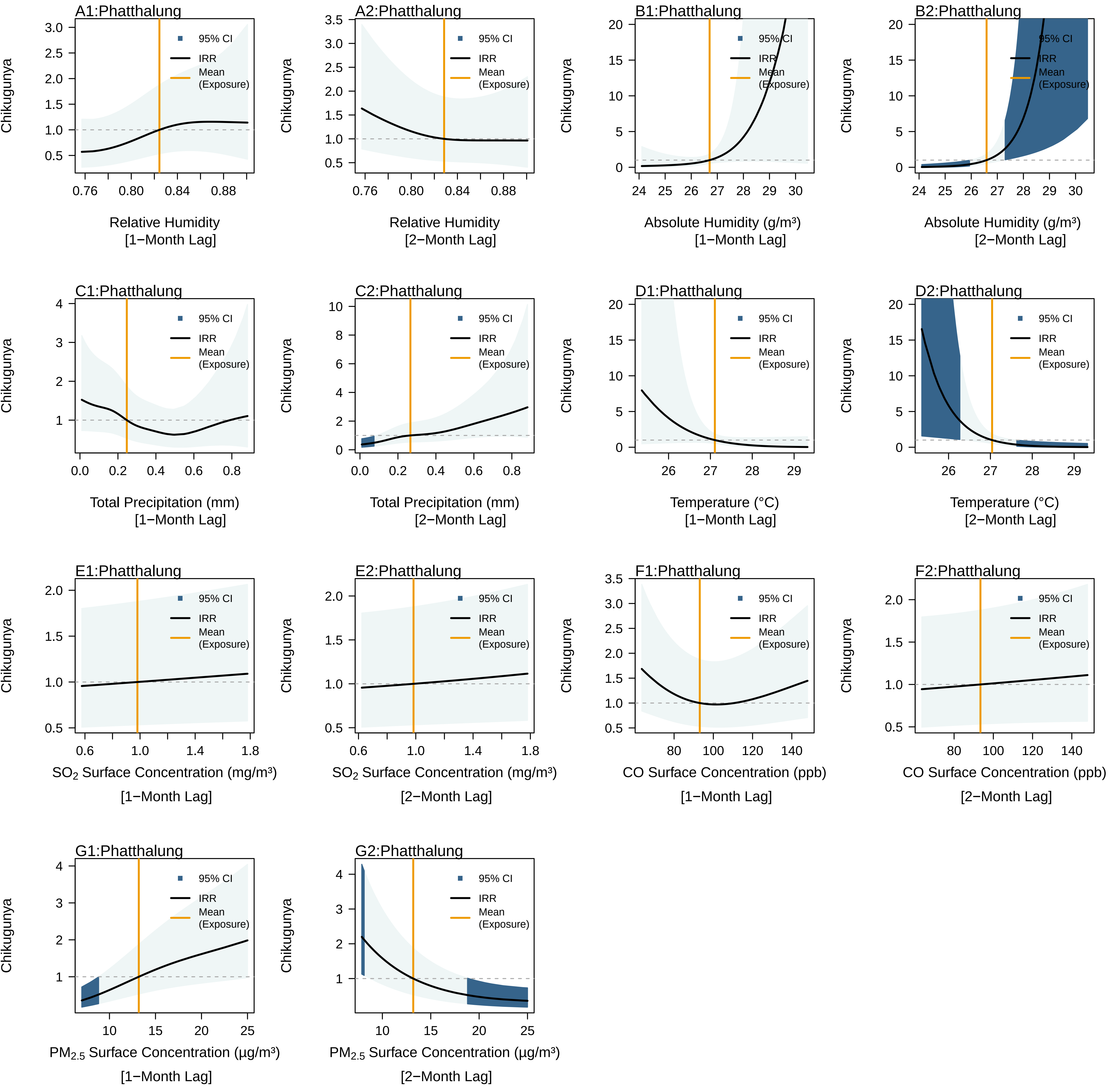

A1:Phatthalung
Chikugunya
Relative Humidity
[1−Month Lag]
95% CI
IRR
Mean
(Exposure)
A2:Phatthalung
Chikugunya
Relative Humidity
[2−Month Lag]
B1:Phatthalung
Chikugunya
Absolute Humidity (g/m³)
[1−Month Lag]
B2:Phatthalung
Chikugunya
Absolute Humidity (g/m³)
[2−Month Lag]
C1:Phatthalung
Chikugunya
Total Precipitation (mm)
[1−Month Lag]
C2:Phatthalung
Chikugunya
Total Precipitation (mm)
[2−Month Lag]
D1:Phatthalung
Chikugunya
Temperature (°C)
[1−Month Lag]
D2:Phatthalung
Chikugunya
Temperature (°C)
[2−Month Lag]
E1:Phatthalung
Chikugunya
SO_2 Surface Concentration (mg/m³)
[1−Month Lag]
E2:Phatthalung
Chikugunya
SO_2 Surface Concentration (mg/m³)
[2−Month Lag]
F1:Phatthalung
Chikugunya
CO Surface Concentration (ppb)
[1−Month Lag]
F2:Phatthalung
Chikugunya
CO Surface Concentration (ppb)
[2−Month Lag]
G1:Phatthalung
Chikugunya
$PM_{2.5}$ Surface Concentration (µg/m³)
[1−Month Lag]
G2:Phatthalung
Chikugunya
$PM_{2.5}$ Surface Concentration (µg/m³)
[2−Month Lag]

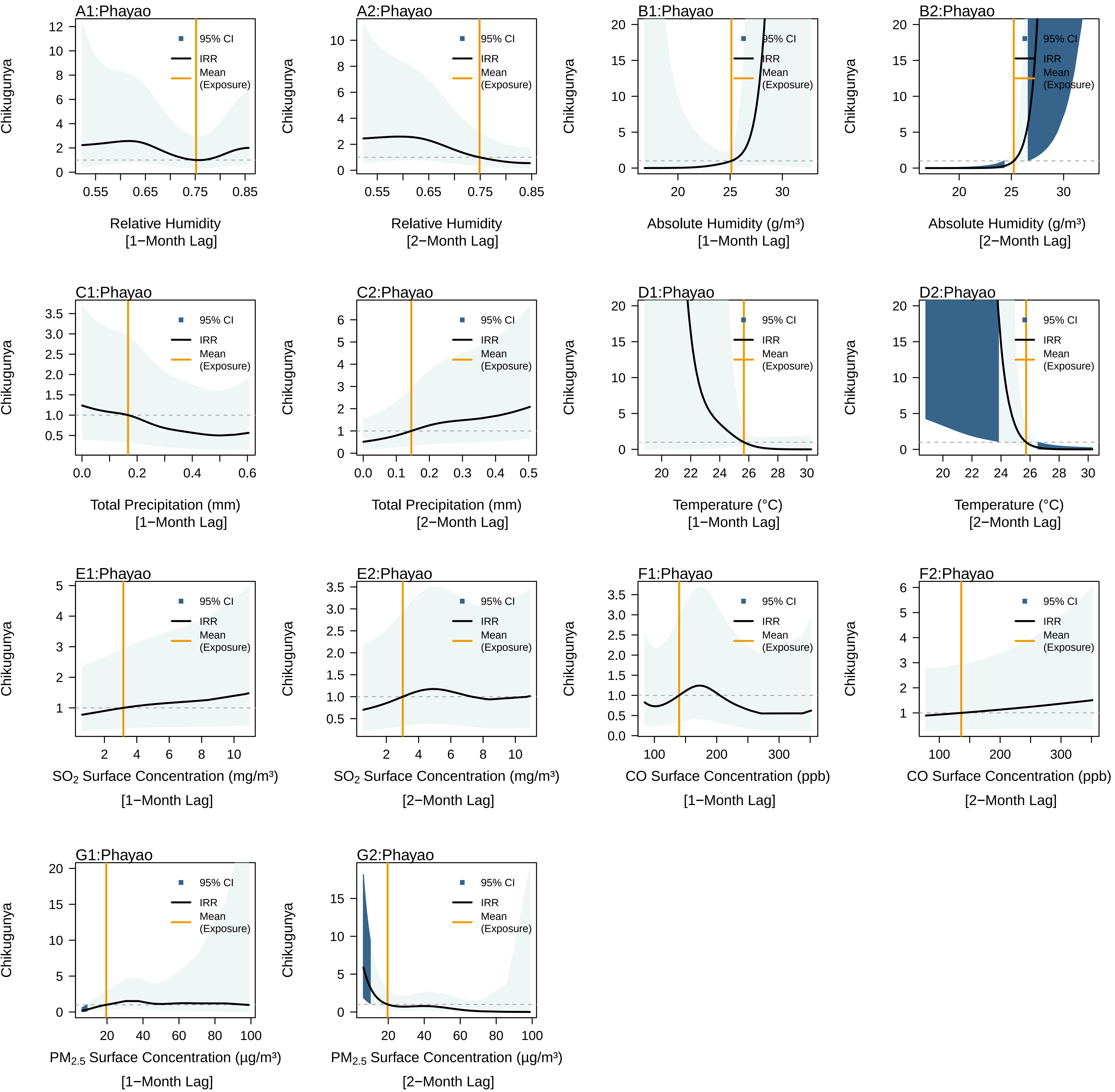

A1:Phayao
A2:Phayao
B1:Phayao
B2:Phayao
C1:Phayao
C2:Phayao
D1:Phayao
D2:Phayao
E1:Phayao
E2:Phayao
F1:Phayao
F2:Phayao
G1:Phayao
G2:Phayao
95% CI
IRR
Mean
(Exposure)
Chikugunya
Relative Humidity
[1−Month Lag]
Relative Humidity
[2−Month Lag]
Absolute Humidity (g/m³)
[1−Month Lag]
Absolute Humidity (g/m³)
[2−Month Lag]
Total Precipitation (mm)
[1−Month Lag]
Total Precipitation (mm)
[2−Month Lag]
Temperature (°C)
[1−Month Lag]
Temperature (°C)
[2−Month Lag]
SO_2 Surface Concentration (mg/m³)
[1−Month Lag]
SO_2 Surface Concentration (mg/m³)
[2−Month Lag]
CO Surface Concentration (ppb)
[1−Month Lag]
CO Surface Concentration (ppb)
[2−Month Lag]
$PM_{2.5}$ Surface Concentration (μg/m³)
[1−Month Lag]
$PM_{2.5}$ Surface Concentration (μg/m³)
[2−Month Lag]

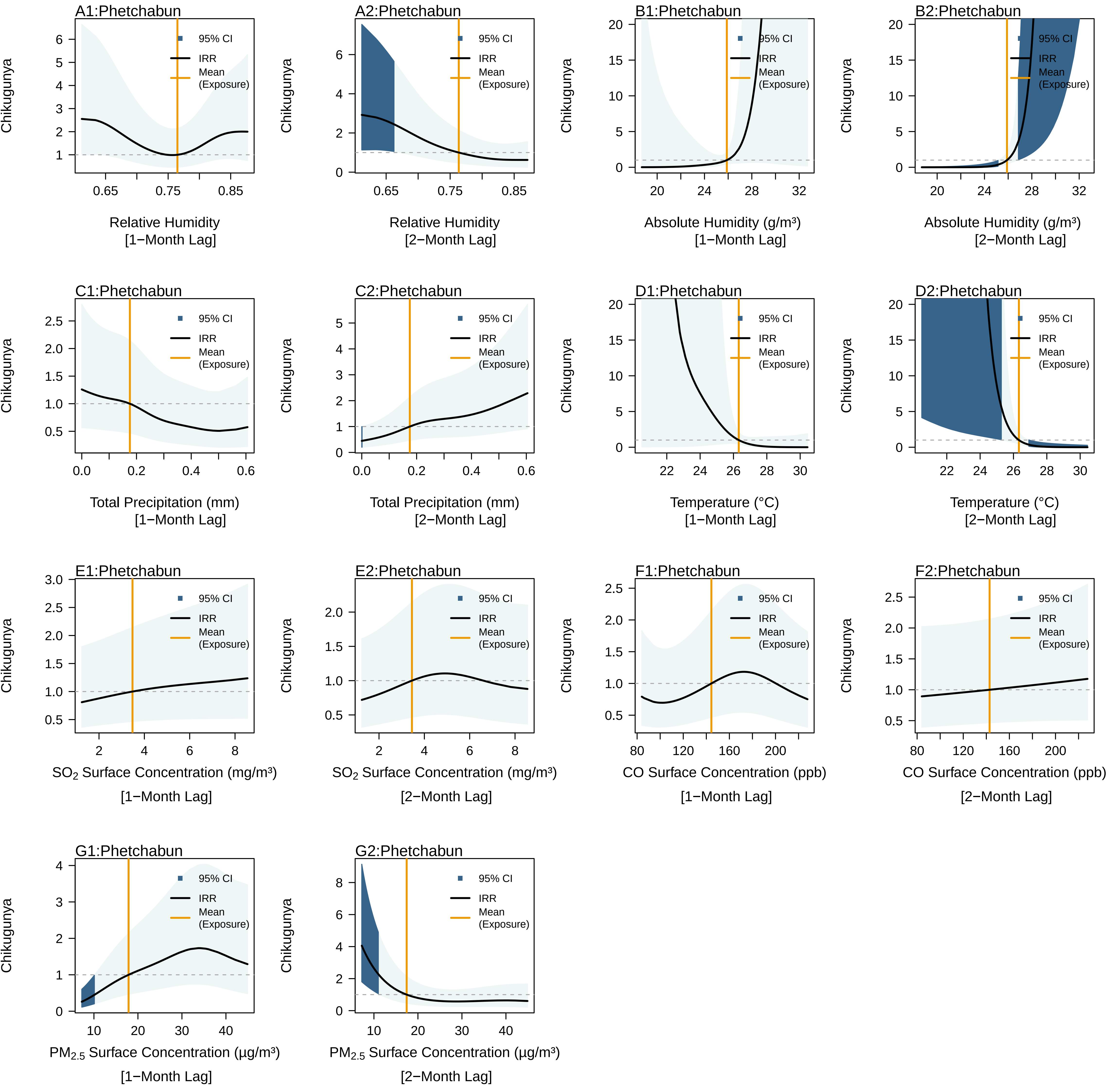
A1:Phetchabun
Chikugunya
95% CI
IRR
Mean (Exposure)
Relative Humidity [1−Month Lag]
A2:Phetchabun
Relative Humidity [2−Month Lag]
B1:Phetchabun
Absolute Humidity (g/m³) [1−Month Lag]
B2:Phetchabun
Absolute Humidity (g/m³) [2−Month Lag]
C1:Phetchabun
Total Precipitation (mm) [1−Month Lag]
C2:Phetchabun
Total Precipitation (mm) [2−Month Lag]
D1:Phetchabun
Temperature (°C) [1−Month Lag]
D2:Phetchabun
Temperature (°C) [2−Month Lag]
E1:Phetchabun
SO_2 Surface Concentration (mg/m³) [1−Month Lag]
E2:Phetchabun
SO_2 Surface Concentration (mg/m³) [2−Month Lag]
F1:Phetchabun
CO Surface Concentration (ppb) [1−Month Lag]
F2:Phetchabun
CO Surface Concentration (ppb) [2−Month Lag]
G1:Phetchabun
$PM_{2.5}$ Surface Concentration (µg/m³) [1−Month Lag]
G2:Phetchabun
$PM_{2.5}$ Surface Concentration (µg/m³) [2−Month Lag]

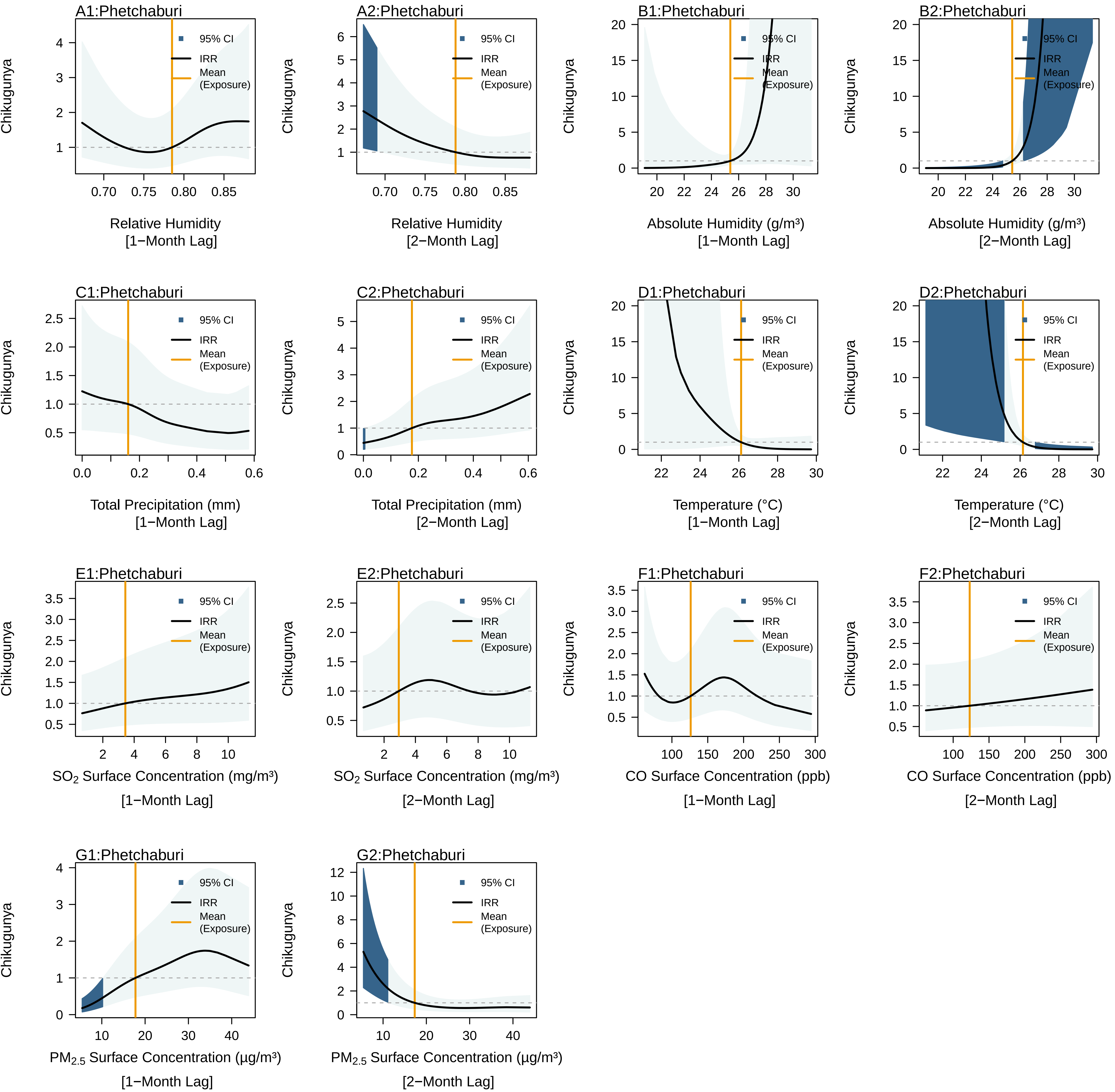
A1:Phetchaburi
A2:Phetchaburi
B1:Phetchaburi
B2:Phetchaburi
C1:Phetchaburi
C2:Phetchaburi
D1:Phetchaburi
D2:Phetchaburi
E1:Phetchaburi
E2:Phetchaburi
F1:Phetchaburi
F2:Phetchaburi
G1:Phetchaburi
G2:Phetchaburi
Chikugunya
95% CI
IRR
Mean
(Exposure)
Relative Humidity
[1−Month Lag]
[2−Month Lag]
Absolute Humidity (g/m³)
Total Precipitation (mm)
Temperature (°C)
SO_2 Surface Concentration (mg/m³)
CO Surface Concentration (ppb)
$PM_{2.5}$ Surface Concentration (µg/m³)

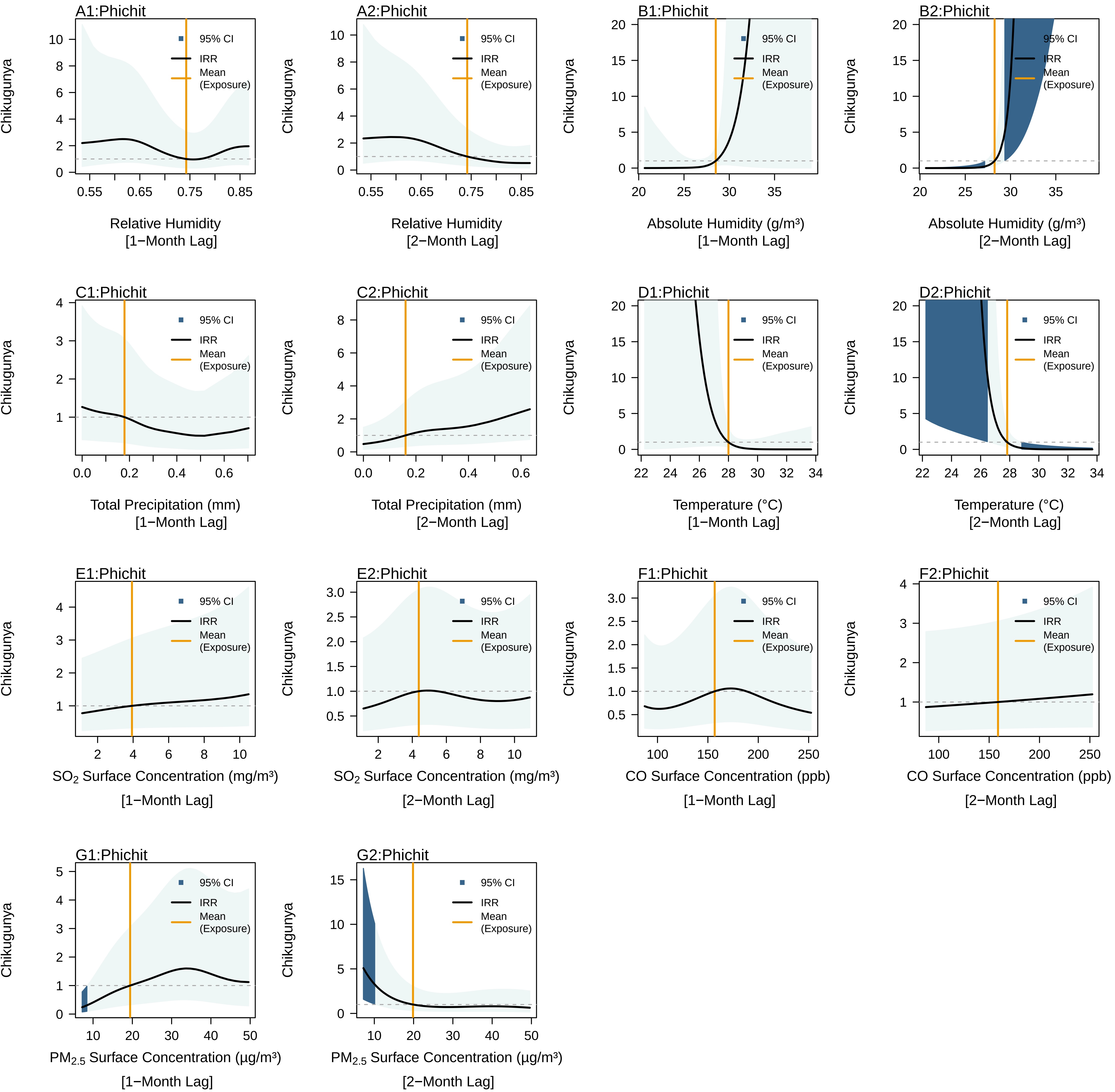
A1:Phichit
Chikungunya
95% CI
IRR
Mean (Exposure)
Relative Humidity [1−Month Lag]
A2:Phichit
Chikungunya
95% CI
IRR
Mean (Exposure)
Relative Humidity [2−Month Lag]
B1:Phichit
Chikungunya
95% CI
IRR
Mean (Exposure)
Absolute Humidity (g/m³) [1−Month Lag]
B2:Phichit
Chikungunya
95% CI
IRR
Mean (Exposure)
Absolute Humidity (g/m³) [2−Month Lag]
C1:Phichit
Chikungunya
95% CI
IRR
Mean (Exposure)
Total Precipitation (mm) [1−Month Lag]
C2:Phichit
Chikungunya
95% CI
IRR
Mean (Exposure)
Total Precipitation (mm) [2−Month Lag]
D1:Phichit
Chikungunya
95% CI
IRR
Mean (Exposure)
Temperature (°C) [1−Month Lag]
D2:Phichit
Chikungunya
95% CI
IRR
Mean (Exposure)
Temperature (°C) [2−Month Lag]
E1:Phichit
Chikungunya
95% CI
IRR
Mean (Exposure)
SO2 Surface Concentration (mg/m³) [1−Month Lag]
E2:Phichit
Chikungunya
95% CI
IRR
Mean (Exposure)
SO2 Surface Concentration (mg/m³) [2−Month Lag]
F1:Phichit
Chikungunya
95% CI
IRR
Mean (Exposure)
CO Surface Concentration (ppb) [1−Month Lag]
F2:Phichit
Chikungunya
95% CI
IRR
Mean (Exposure)
CO Surface Concentration (ppb) [2−Month Lag]
G1:Phichit
Chikungunya
95% CI
IRR
Mean (Exposure)
PM2.5 Surface Concentration (µg/m³) [1−Month Lag]
G2:Phichit
Chikungunya
95% CI
IRR
Mean (Exposure)
PM2.5 Surface Concentration (µg/m³) [2−Month Lag]

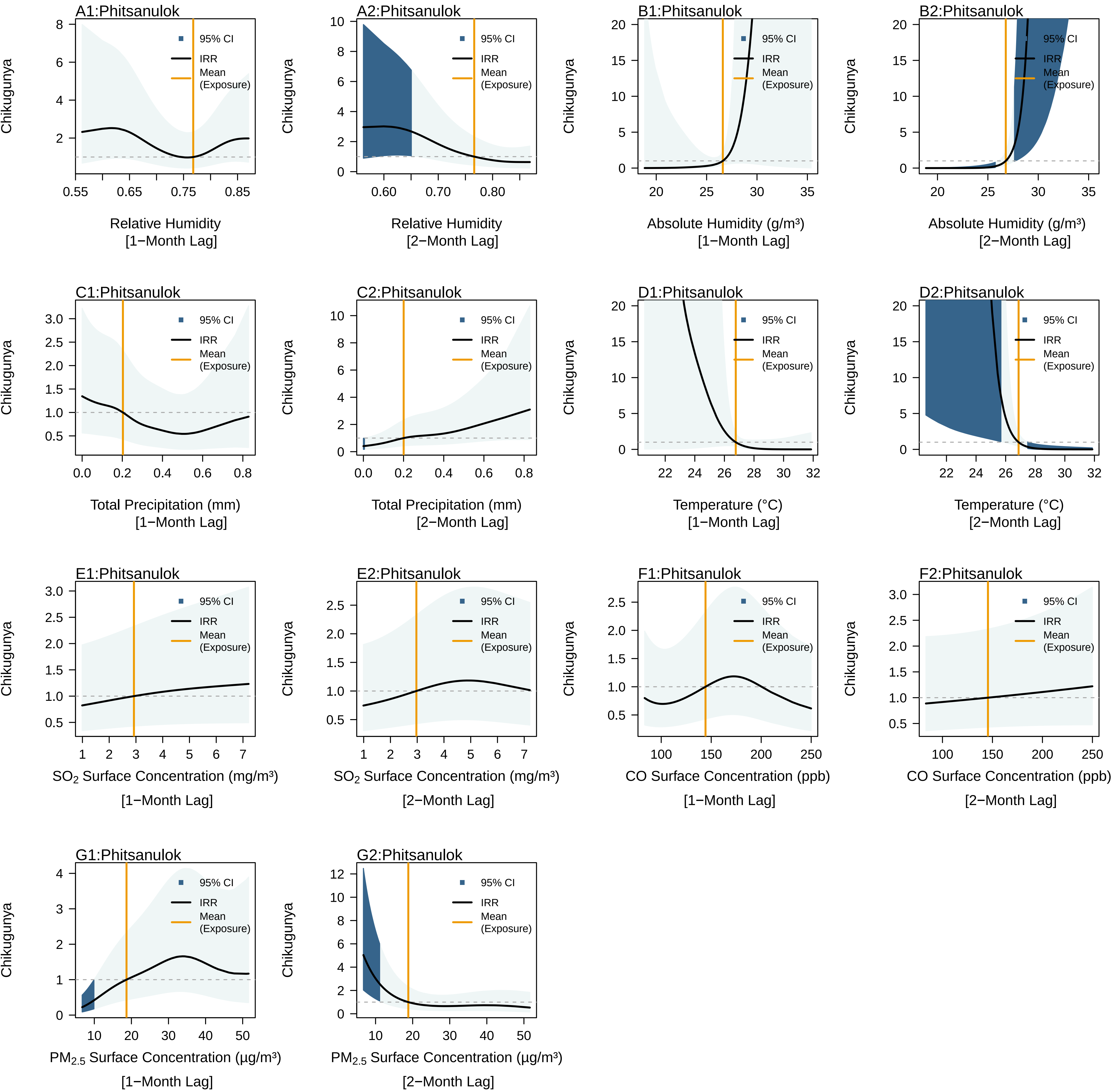
A1:Phitsanulok
A2:Phitsanulok
B1:Phitsanulok
B2:Phitsanulok
C1:Phitsanulok
C2:Phitsanulok
D1:Phitsanulok
D2:Phitsanulok
E1:Phitsanulok
E2:Phitsanulok
F1:Phitsanulok
F2:Phitsanulok
G1:Phitsanulok
G2:Phitsanulok
Chikugunya
95% CI
IRR
Mean
(Exposure)
Relative Humidity
[1−Month Lag]
Relative Humidity
[2−Month Lag]
Absolute Humidity (g/m³)
[1−Month Lag]
Absolute Humidity (g/m³)
[2−Month Lag]
Total Precipitation (mm)
[1−Month Lag]
Total Precipitation (mm)
[2−Month Lag]
Temperature (°C)
[1−Month Lag]
Temperature (°C)
[2−Month Lag]
SO2 Surface Concentration (mg/m³)
[1−Month Lag]
SO2 Surface Concentration (mg/m³)
[2−Month Lag]
CO Surface Concentration (ppb)
[1−Month Lag]
CO Surface Concentration (ppb)
[2−Month Lag]
PM2.5 Surface Concentration (µg/m³)
[1−Month Lag]
PM2.5 Surface Concentration (µg/m³)
[2−Month Lag]

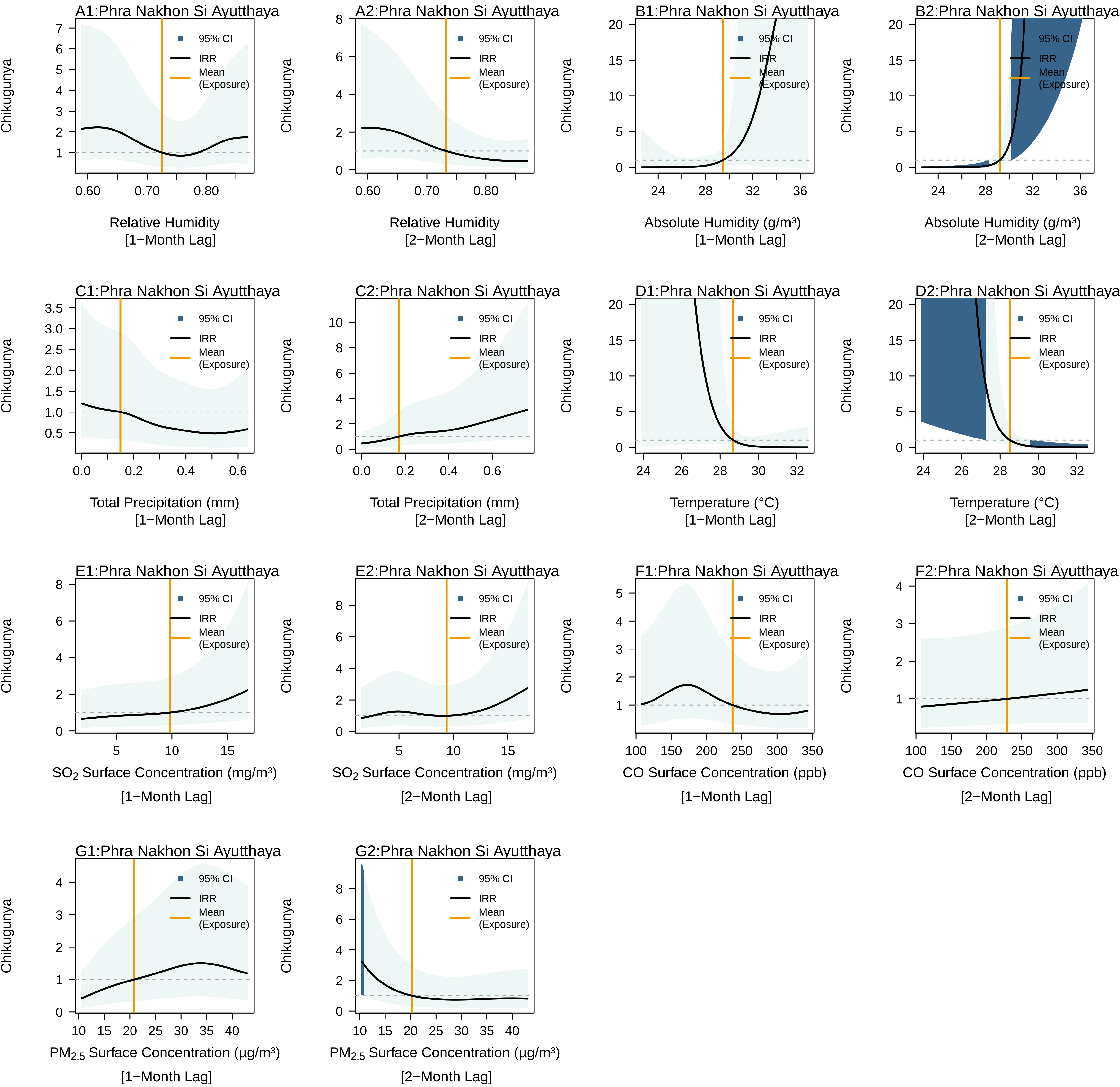
A1:Phra Nakhon Si Ayutthaya
Chikugunya
95% CI
IRR
Mean (Exposure)
Relative Humidity [1−Month Lag]
A2:Phra Nakhon Si Ayutthaya
Chikugunya
95% CI
IRR
Mean (Exposure)
Relative Humidity [2−Month Lag]
B1:Phra Nakhon Si Ayutthaya
Chikugunya
95% CI
IRR
Mean (Exposure)
Absolute Humidity (g/m³) [1−Month Lag]
B2:Phra Nakhon Si Ayutthaya
Chikugunya
95% CI
IRR
Mean (Exposure)
Absolute Humidity (g/m³) [2−Month Lag]
C1:Phra Nakhon Si Ayutthaya
Chikugunya
95% CI
IRR
Mean (Exposure)
Total Precipitation (mm) [1−Month Lag]
C2:Phra Nakhon Si Ayutthaya
Chikugunya
95% CI
IRR
Mean (Exposure)
Total Precipitation (mm) [2−Month Lag]
D1:Phra Nakhon Si Ayutthaya
Chikugunya
95% CI
IRR
Mean (Exposure)
Temperature (°C) [1−Month Lag]
D2:Phra Nakhon Si Ayutthaya
Chikugunya
95% CI
IRR
Mean (Exposure)
Temperature (°C) [2−Month Lag]
E1:Phra Nakhon Si Ayutthaya
Chikugunya
95% CI
IRR
Mean (Exposure)
SO2 Surface Concentration (mg/m³) [1−Month Lag]
E2:Phra Nakhon Si Ayutthaya
Chikugunya
95% CI
IRR
Mean (Exposure)
SO2 Surface Concentration (mg/m³) [2−Month Lag]
F1:Phra Nakhon Si Ayutthaya
Chikugunya
95% CI
IRR
Mean (Exposure)
CO Surface Concentration (ppb) [1−Month Lag]
F2:Phra Nakhon Si Ayutthaya
Chikugunya
95% CI
IRR
Mean (Exposure)
CO Surface Concentration (ppb) [2−Month Lag]
G1:Phra Nakhon Si Ayutthaya
Chikugunya
95% CI
IRR
Mean (Exposure)
PM2.5 Surface Concentration (μg/m³) [1−Month Lag]
G2:Phra Nakhon Si Ayutthaya
Chikugunya
95% CI
IRR
Mean (Exposure)
PM2.5 Surface Concentration (μg/m³) [2−Month Lag]

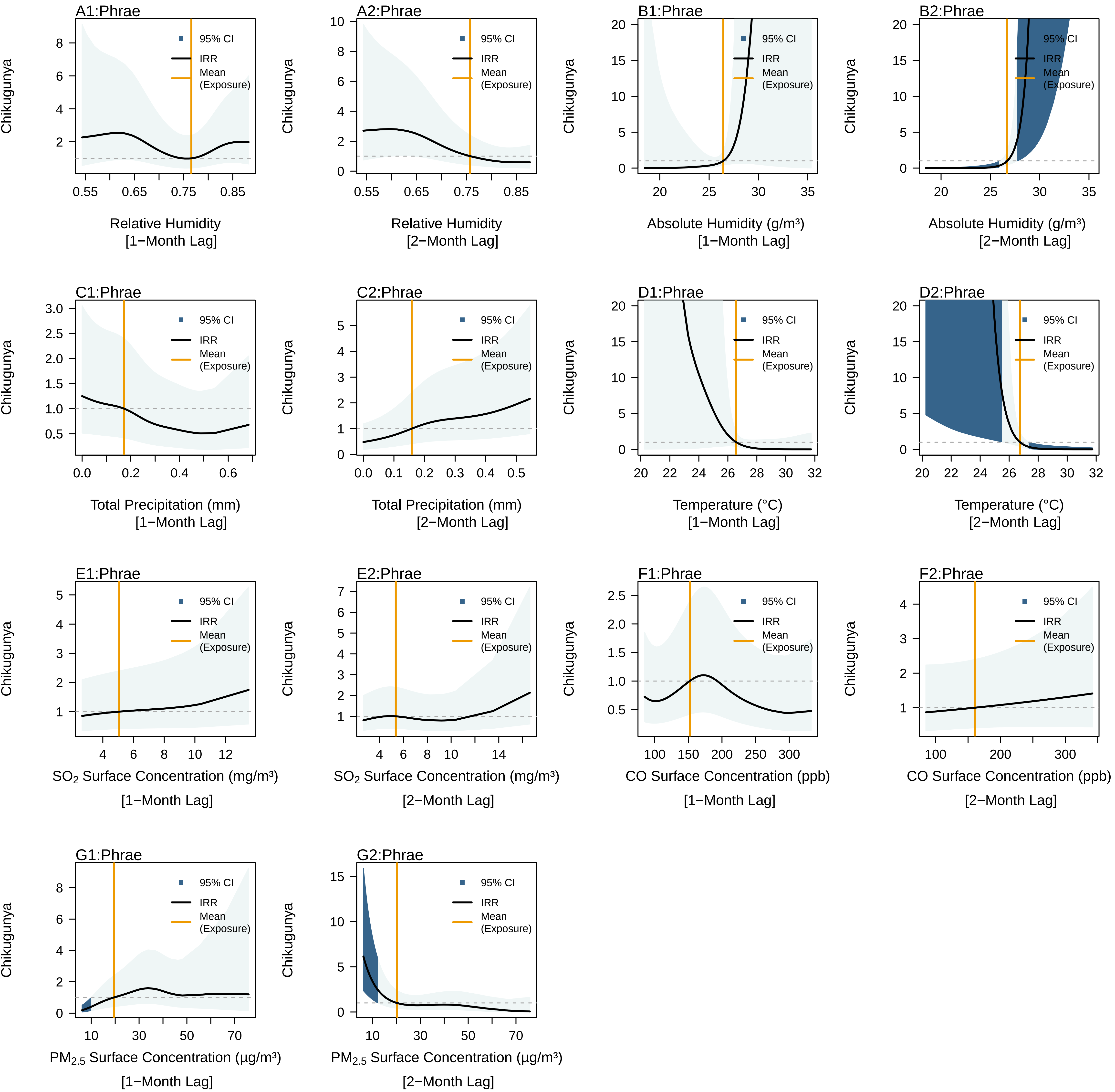
A1:Phrae
A2:Phrae
B1:Phrae
B2:Phrae
C1:Phrae
C2:Phrae
D1:Phrae
D2:Phrae
E1:Phrae
E2:Phrae
F1:Phrae
F2:Phrae
G1:Phrae
G2:Phrae
Chikugunya
95% CI
IRR
Mean (Exposure)
Relative Humidity [1−Month Lag]
Relative Humidity [2−Month Lag]
Absolute Humidity (g/m³) [1−Month Lag]
Absolute Humidity (g/m³) [2−Month Lag]
Total Precipitation (mm) [1−Month Lag]
Total Precipitation (mm) [2−Month Lag]
Temperature (°C) [1−Month Lag]
Temperature (°C) [2−Month Lag]
SO_2 Surface Concentration (mg/m³) [1−Month Lag]
SO_2 Surface Concentration (mg/m³) [2−Month Lag]
CO Surface Concentration (ppb) [1−Month Lag]
CO Surface Concentration (ppb) [2−Month Lag]
$PM_{2.5}$ Surface Concentration (μg/m³) [1−Month Lag]
$PM_{2.5}$ Surface Concentration (μg/m³) [2−Month Lag]

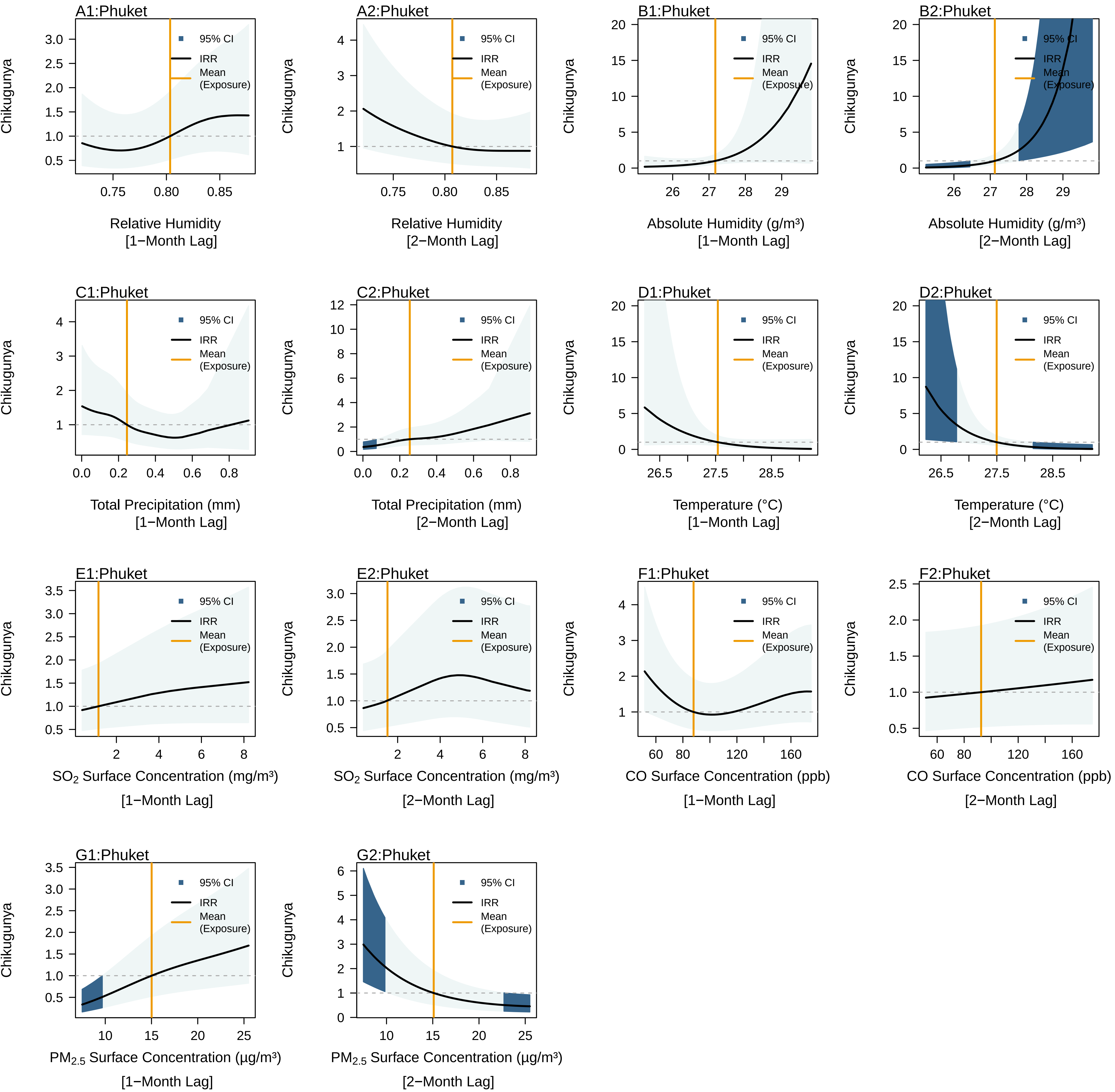
A1:Phuket
A2:Phuket
B1:Phuket
B2:Phuket
C1:Phuket
C2:Phuket
D1:Phuket
D2:Phuket
E1:Phuket
E2:Phuket
F1:Phuket
F2:Phuket
G1:Phuket
G2:Phuket
Chikugunya
95% CI
IRR
Mean
(Exposure)
Relative Humidity
[1−Month Lag]
[2−Month Lag]
Absolute Humidity (g/m³)
Total Precipitation (mm)
Temperature (°C)
SO_2 Surface Concentration (mg/m³)
CO Surface Concentration (ppb)
$PM_{2.5}$ Surface Concentration (μg/m³)

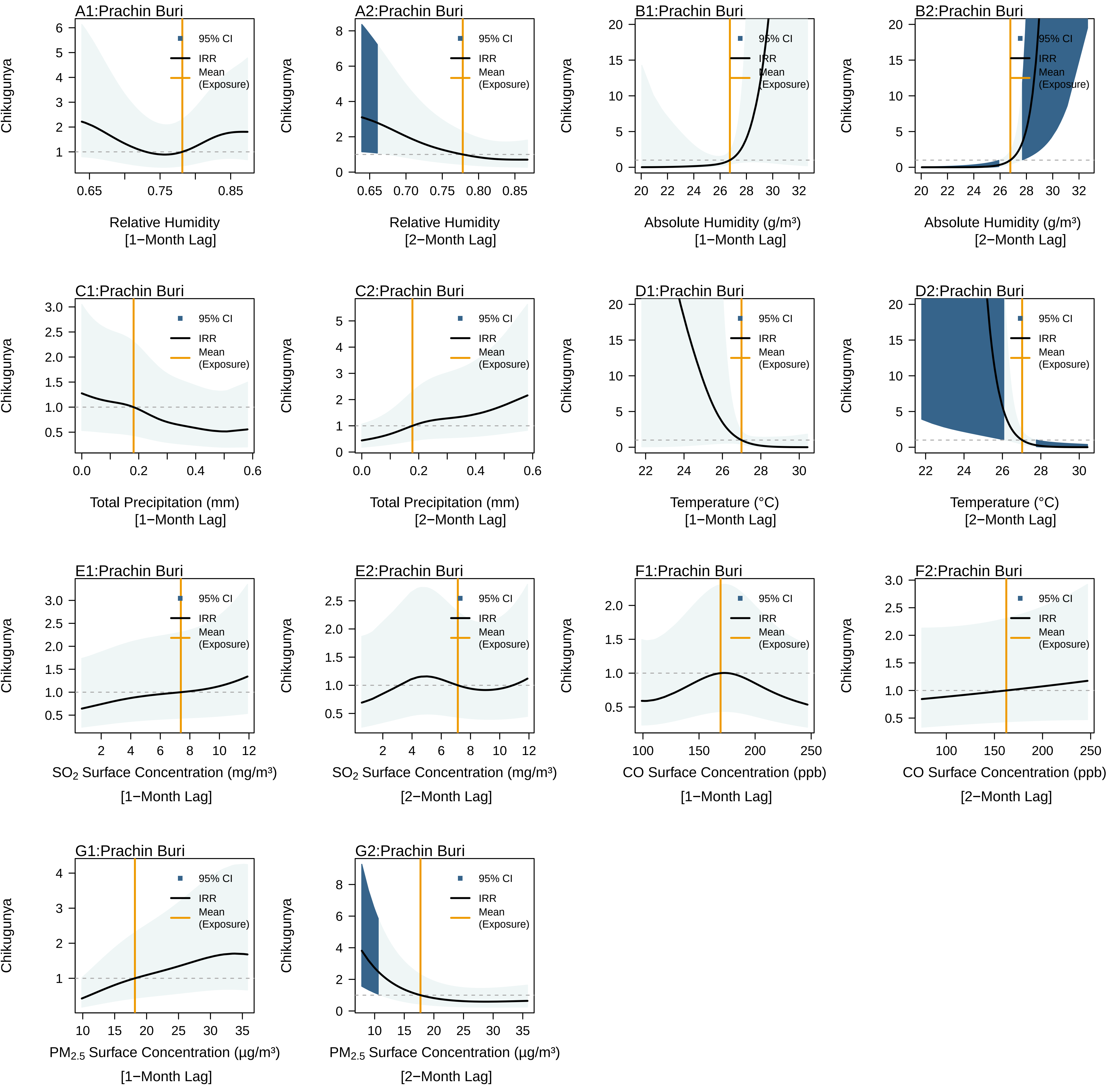
A1:Prachin Buri
Chikugunya
95% CI
IRR
Mean
(Exposure)
Relative Humidity
[1−Month Lag]
A2:Prachin Buri
Chikugunya
95% CI
IRR
Mean
(Exposure)
Relative Humidity
[2−Month Lag]
B1:Prachin Buri
Chikugunya
95% CI
IRR
Mean
(Exposure)
Absolute Humidity (g/m³)
[1−Month Lag]
B2:Prachin Buri
Chikugunya
95% CI
IRR
Mean
(Exposure)
Absolute Humidity (g/m³)
[2−Month Lag]
C1:Prachin Buri
Chikugunya
95% CI
IRR
Mean
(Exposure)
Total Precipitation (mm)
[1−Month Lag]
C2:Prachin Buri
Chikugunya
95% CI
IRR
Mean
(Exposure)
Total Precipitation (mm)
[2−Month Lag]
D1:Prachin Buri
Chikugunya
95% CI
IRR
Mean
(Exposure)
Temperature (°C)
[1−Month Lag]
D2:Prachin Buri
Chikugunya
95% CI
IRR
Mean
(Exposure)
Temperature (°C)
[2−Month Lag]
E1:Prachin Buri
Chikugunya
95% CI
IRR
Mean
(Exposure)
SO_2 Surface Concentration (mg/m³)
[1−Month Lag]
E2:Prachin Buri
Chikugunya
95% CI
IRR
Mean
(Exposure)
SO_2 Surface Concentration (mg/m³)
[2−Month Lag]
F1:Prachin Buri
Chikugunya
95% CI
IRR
Mean
(Exposure)
CO Surface Concentration (ppb)
[1−Month Lag]
F2:Prachin Buri
Chikugunya
95% CI
IRR
Mean
(Exposure)
CO Surface Concentration (ppb)
[2−Month Lag]
G1:Prachin Buri
Chikugunya
95% CI
IRR
Mean
(Exposure)
$PM_{2.5}$ Surface Concentration (µg/m³)
[1−Month Lag]
G2:Prachin Buri
Chikugunya
95% CI
IRR
Mean
(Exposure)
$PM_{2.5}$ Surface Concentration (µg/m³)
[2−Month Lag]

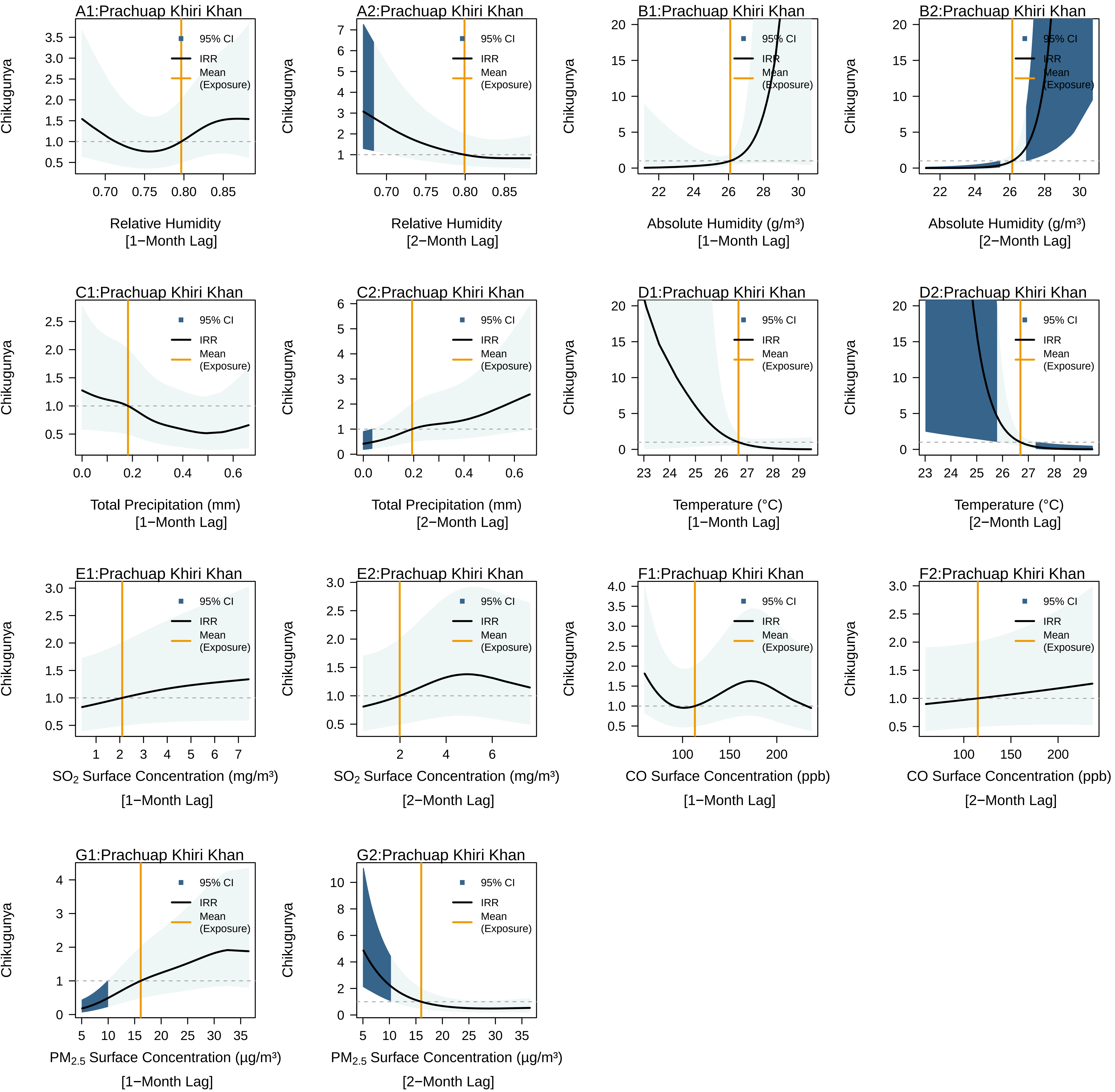
A1:Prachuap Khiri Khan
A2:Prachuap Khiri Khan
B1:Prachuap Khiri Khan
B2:Prachuap Khiri Khan
C1:Prachuap Khiri Khan
C2:Prachuap Khiri Khan
D1:Prachuap Khiri Khan
D2:Prachuap Khiri Khan
E1:Prachuap Khiri Khan
E2:Prachuap Khiri Khan
F1:Prachuap Khiri Khan
F2:Prachuap Khiri Khan
G1:Prachuap Khiri Khan
G2:Prachuap Khiri Khan
95% CI
IRR
Mean (Exposure)
Chikugunya
Relative Humidity [1−Month Lag]
Relative Humidity [2−Month Lag]
Absolute Humidity (g/m³) [1−Month Lag]
Absolute Humidity (g/m³) [2−Month Lag]
Total Precipitation (mm) [1−Month Lag]
Total Precipitation (mm) [2−Month Lag]
Temperature (°C) [1−Month Lag]
Temperature (°C) [2−Month Lag]
SO_2 Surface Concentration (mg/m³) [1−Month Lag]
SO_2 Surface Concentration (mg/m³) [2−Month Lag]
CO Surface Concentration (ppb) [1−Month Lag]
CO Surface Concentration (ppb) [2−Month Lag]
$PM_{2.5}$ Surface Concentration (µg/m³) [1−Month Lag]
$PM_{2.5}$ Surface Concentration (µg/m³) [2−Month Lag]

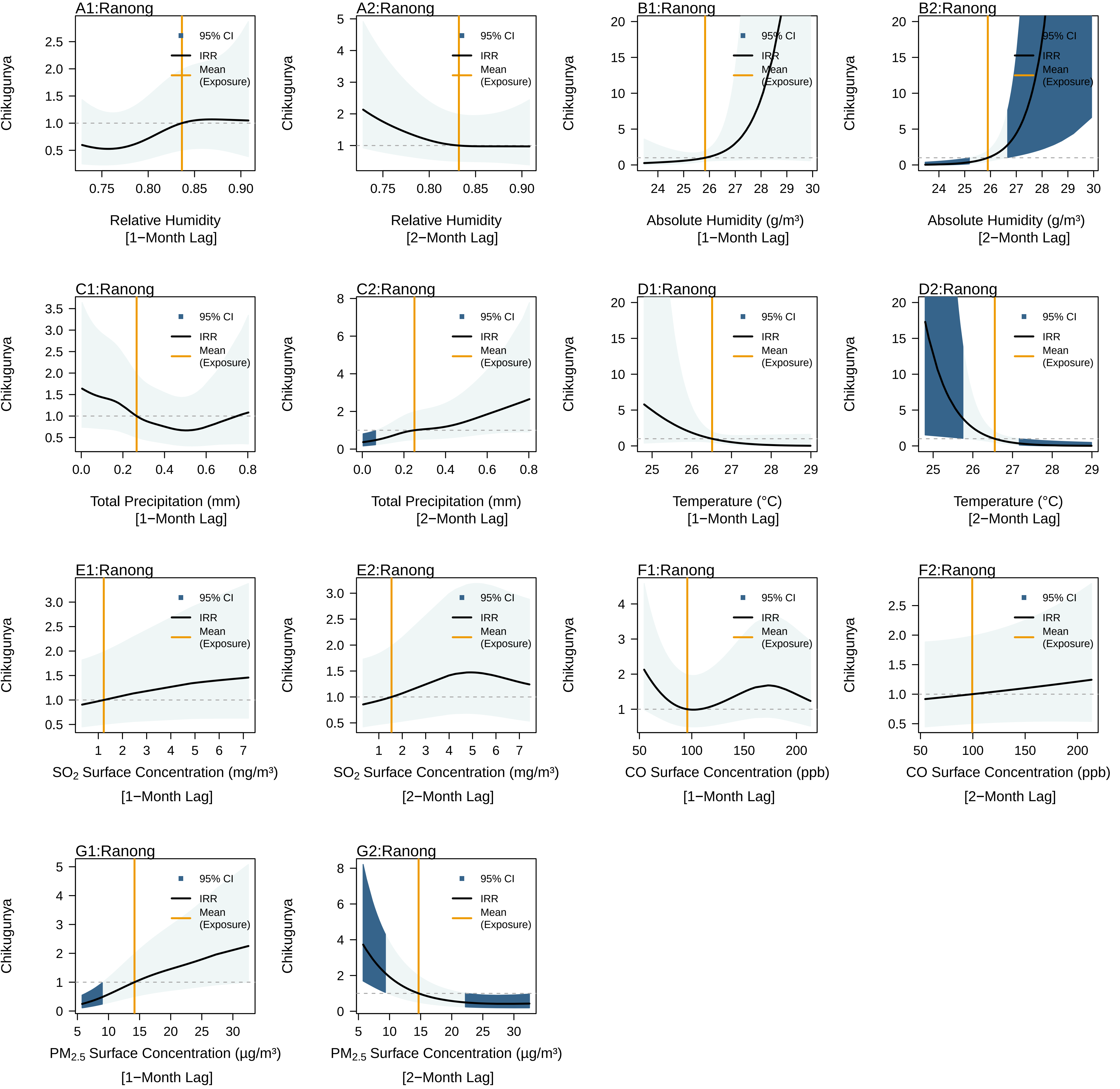
A1:Ranong
A2:Ranong
B1:Ranong
B2:Ranong
C1:Ranong
C2:Ranong
D1:Ranong
D2:Ranong
E1:Ranong
E2:Ranong
F1:Ranong
F2:Ranong
G1:Ranong
G2:Ranong
Chikugunya
95% CI
IRR
Mean (Exposure)
Relative Humidity [1−Month Lag]
Relative Humidity [2−Month Lag]
Absolute Humidity (g/m³) [1−Month Lag]
Absolute Humidity (g/m³) [2−Month Lag]
Total Precipitation (mm) [1−Month Lag]
Total Precipitation (mm) [2−Month Lag]
Temperature (°C) [1−Month Lag]
Temperature (°C) [2−Month Lag]
SO_2 Surface Concentration (mg/m³) [1−Month Lag]
SO_2 Surface Concentration (mg/m³) [2−Month Lag]
CO Surface Concentration (ppb) [1−Month Lag]
CO Surface Concentration (ppb) [2−Month Lag]
$PM_{2.5}$ Surface Concentration (µg/m³) [1−Month Lag]
$PM_{2.5}$ Surface Concentration (µg/m³) [2−Month Lag]

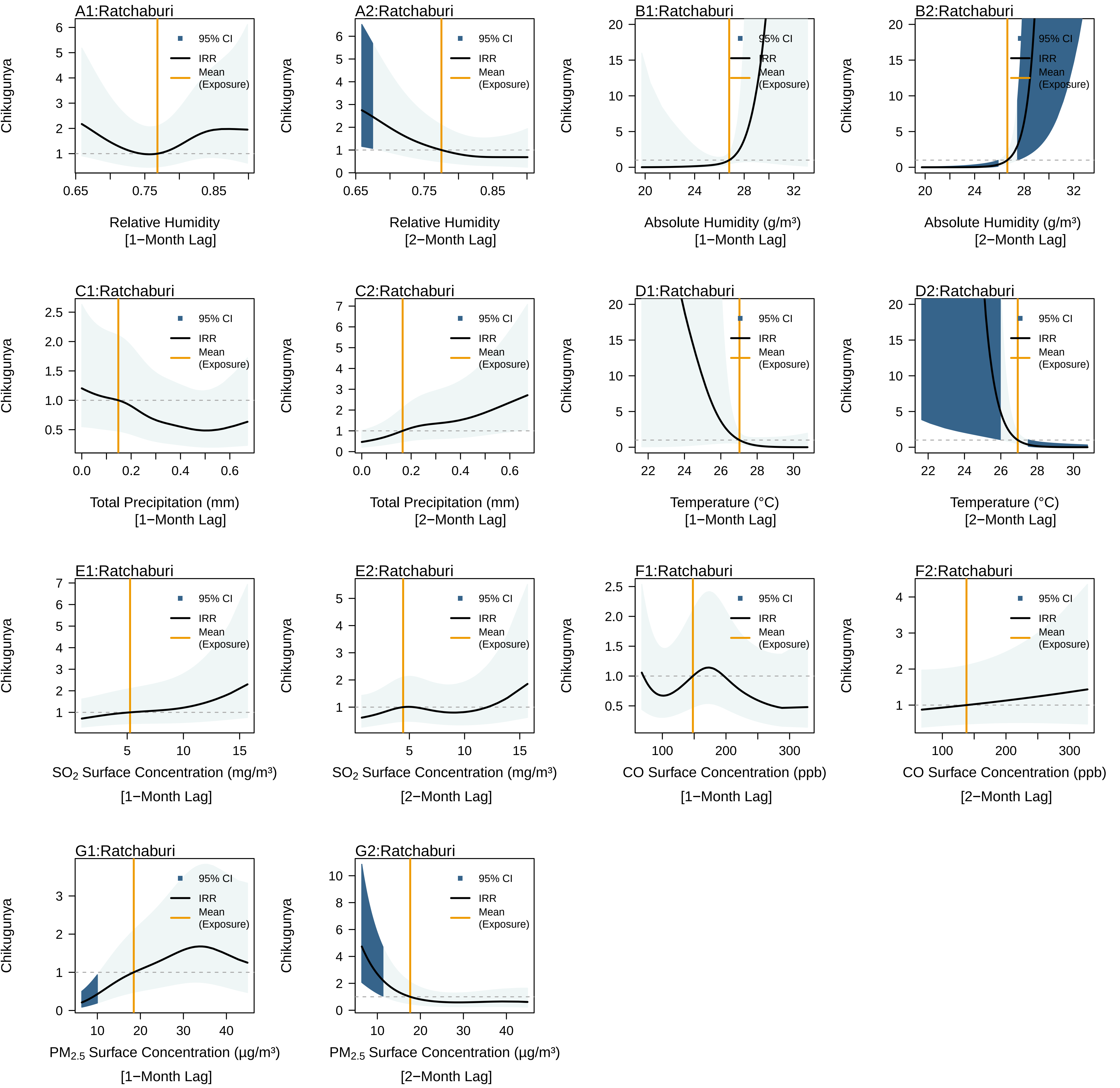
A1:Ratchaburi
95% CI
IRR
Mean (Exposure)
Chikugunya
Relative Humidity [1−Month Lag]
A2:Ratchaburi
Relative Humidity [2−Month Lag]
B1:Ratchaburi
Absolute Humidity (g/m³) [1−Month Lag]
B2:Ratchaburi
Absolute Humidity (g/m³) [2−Month Lag]
C1:Ratchaburi
Total Precipitation (mm) [1−Month Lag]
C2:Ratchaburi
Total Precipitation (mm) [2−Month Lag]
D1:Ratchaburi
Temperature (°C) [1−Month Lag]
D2:Ratchaburi
Temperature (°C) [2−Month Lag]
E1:Ratchaburi
SO2 Surface Concentration (mg/m³) [1−Month Lag]
E2:Ratchaburi
SO2 Surface Concentration (mg/m³) [2−Month Lag]
F1:Ratchaburi
CO Surface Concentration (ppb) [1−Month Lag]
F2:Ratchaburi
CO Surface Concentration (ppb) [2−Month Lag]
G1:Ratchaburi
PM2.5 Surface Concentration (µg/m³) [1−Month Lag]
G2:Ratchaburi
PM2.5 Surface Concentration (µg/m³) [2−Month Lag]

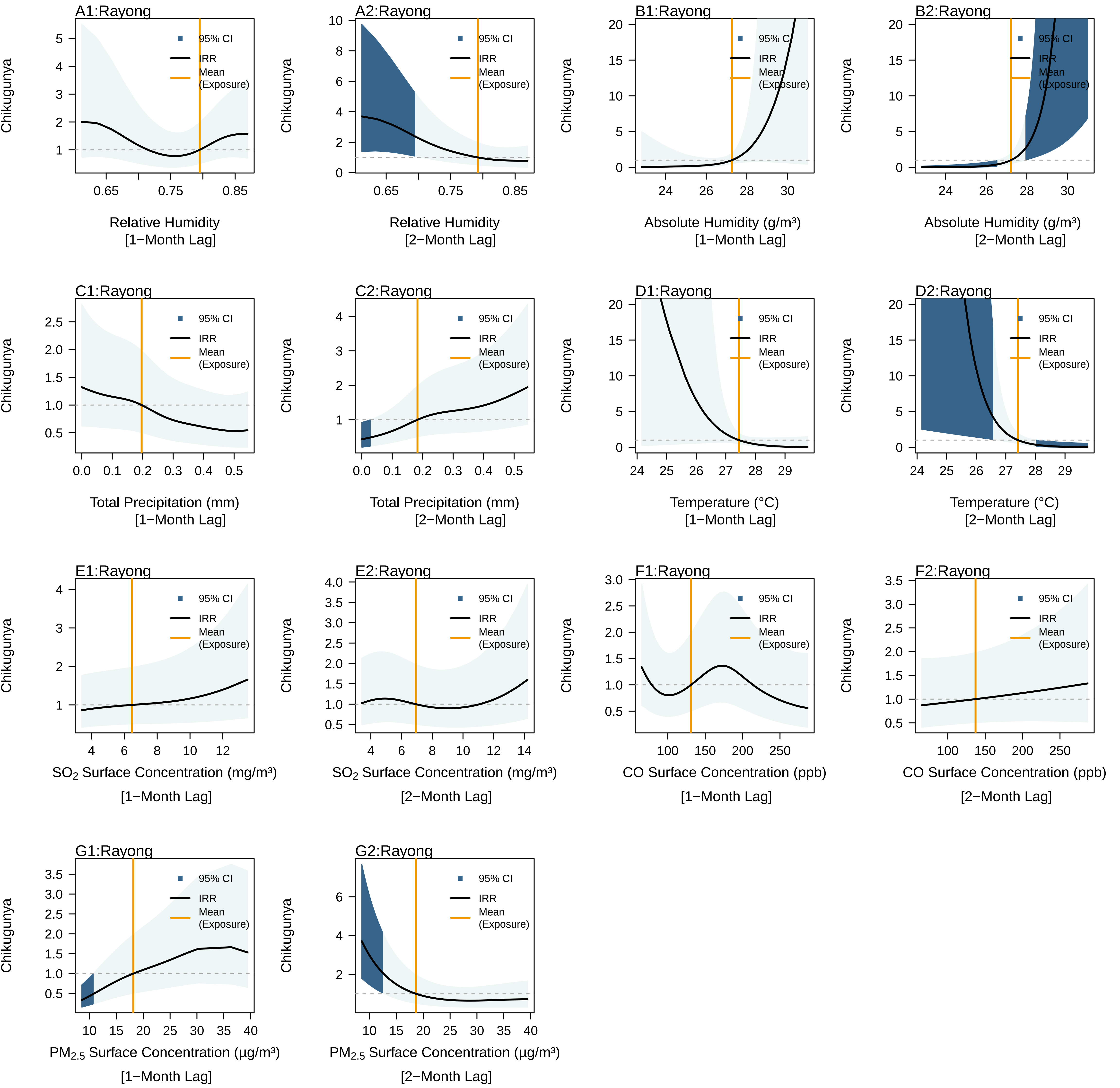
A1:Rayong
95% CI
IRR
Mean (Exposure)
Chikugunya
Relative Humidity [1−Month Lag]
A2:Rayong
Relative Humidity [2−Month Lag]
B1:Rayong
Absolute Humidity (g/m³) [1−Month Lag]
B2:Rayong
Absolute Humidity (g/m³) [2−Month Lag]
C1:Rayong
Total Precipitation (mm) [1−Month Lag]
C2:Rayong
Total Precipitation (mm) [2−Month Lag]
D1:Rayong
Temperature (°C) [1−Month Lag]
D2:Rayong
Temperature (°C) [2−Month Lag]
E1:Rayong
SO_2 Surface Concentration (mg/m³) [1−Month Lag]
E2:Rayong
SO_2 Surface Concentration (mg/m³) [2−Month Lag]
F1:Rayong
CO Surface Concentration (ppb) [1−Month Lag]
F2:Rayong
CO Surface Concentration (ppb) [2−Month Lag]
G1:Rayong
$PM_{2.5}$ Surface Concentration (µg/m³) [1−Month Lag]
G2:Rayong
$PM_{2.5}$ Surface Concentration (µg/m³) [2−Month Lag]

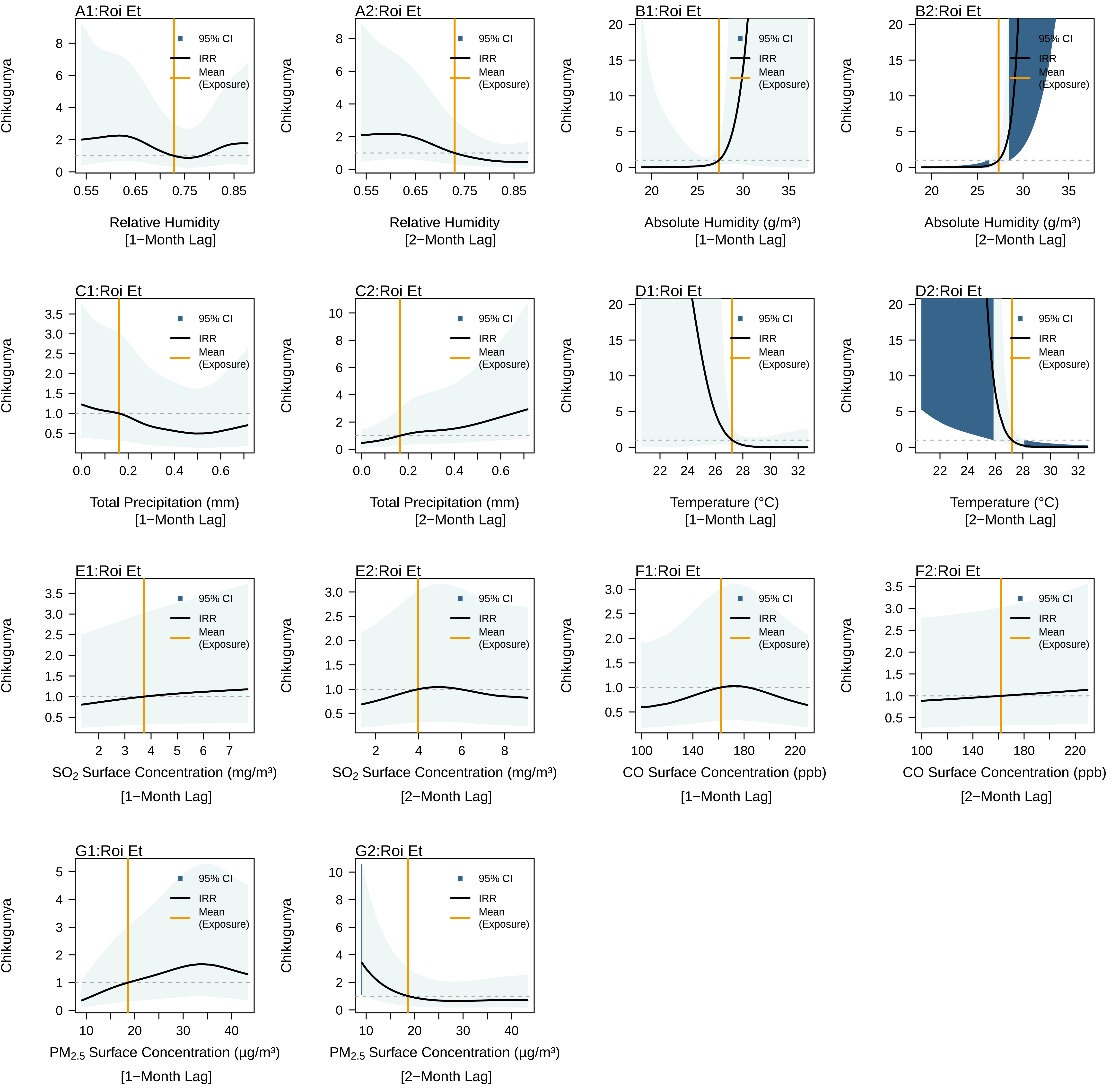
A1:Roi Et
A2:Roi Et
B1:Roi Et
B2:Roi Et
Chikugunya
95% CI
IRR
Mean
(Exposure)
Relative Humidity
[1−Month Lag]
Relative Humidity
[2−Month Lag]
Absolute Humidity (g/m³)
[1−Month Lag]
Absolute Humidity (g/m³)
[2−Month Lag]
C1:Roi Et
C2:Roi Et
D1:Roi Et
D2:Roi Et
Total Precipitation (mm)
[1−Month Lag]
Total Precipitation (mm)
[2−Month Lag]
Temperature (°C)
[1−Month Lag]
Temperature (°C)
[2−Month Lag]
E1:Roi Et
E2:Roi Et
F1:Roi Et
F2:Roi Et
SO_2 Surface Concentration (mg/m³)
[1−Month Lag]
SO_2 Surface Concentration (mg/m³)
[2−Month Lag]
CO Surface Concentration (ppb)
[1−Month Lag]
CO Surface Concentration (ppb)
[2−Month Lag]
G1:Roi Et
G2:Roi Et
$PM_{2.5}$ Surface Concentration (µg/m³)
[1−Month Lag]
$PM_{2.5}$ Surface Concentration (µg/m³)
[2−Month Lag]

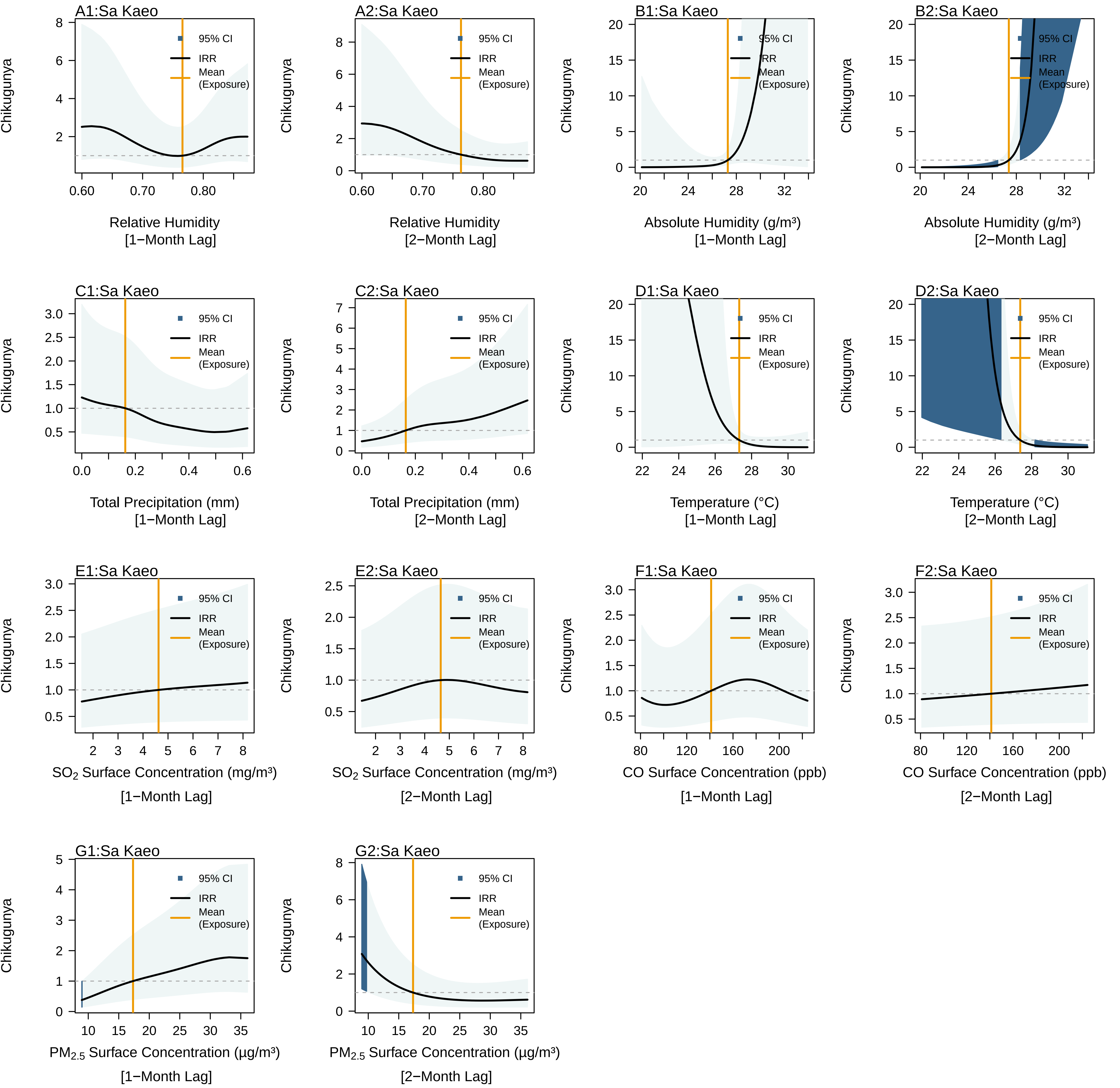
A1:Sa Kaeo
Chikugunya
95% CI
IRR
Mean
(Exposure)
Relative Humidity
[1−Month Lag]
A2:Sa Kaeo
Relative Humidity
[2−Month Lag]
B1:Sa Kaeo
Absolute Humidity (g/m³)
[1−Month Lag]
B2:Sa Kaeo
Absolute Humidity (g/m³)
[2−Month Lag]
C1:Sa Kaeo
Total Precipitation (mm)
[1−Month Lag]
C2:Sa Kaeo
Total Precipitation (mm)
[2−Month Lag]
D1:Sa Kaeo
Temperature (°C)
[1−Month Lag]
D2:Sa Kaeo
Temperature (°C)
[2−Month Lag]
E1:Sa Kaeo
SO2 Surface Concentration (mg/m³)
[1−Month Lag]
E2:Sa Kaeo
SO2 Surface Concentration (mg/m³)
[2−Month Lag]
F1:Sa Kaeo
CO Surface Concentration (ppb)
[1−Month Lag]
F2:Sa Kaeo
CO Surface Concentration (ppb)
[2−Month Lag]
G1:Sa Kaeo
PM2.5 Surface Concentration (μg/m³)
[1−Month Lag]
G2:Sa Kaeo
PM2.5 Surface Concentration (μg/m³)
[2−Month Lag]

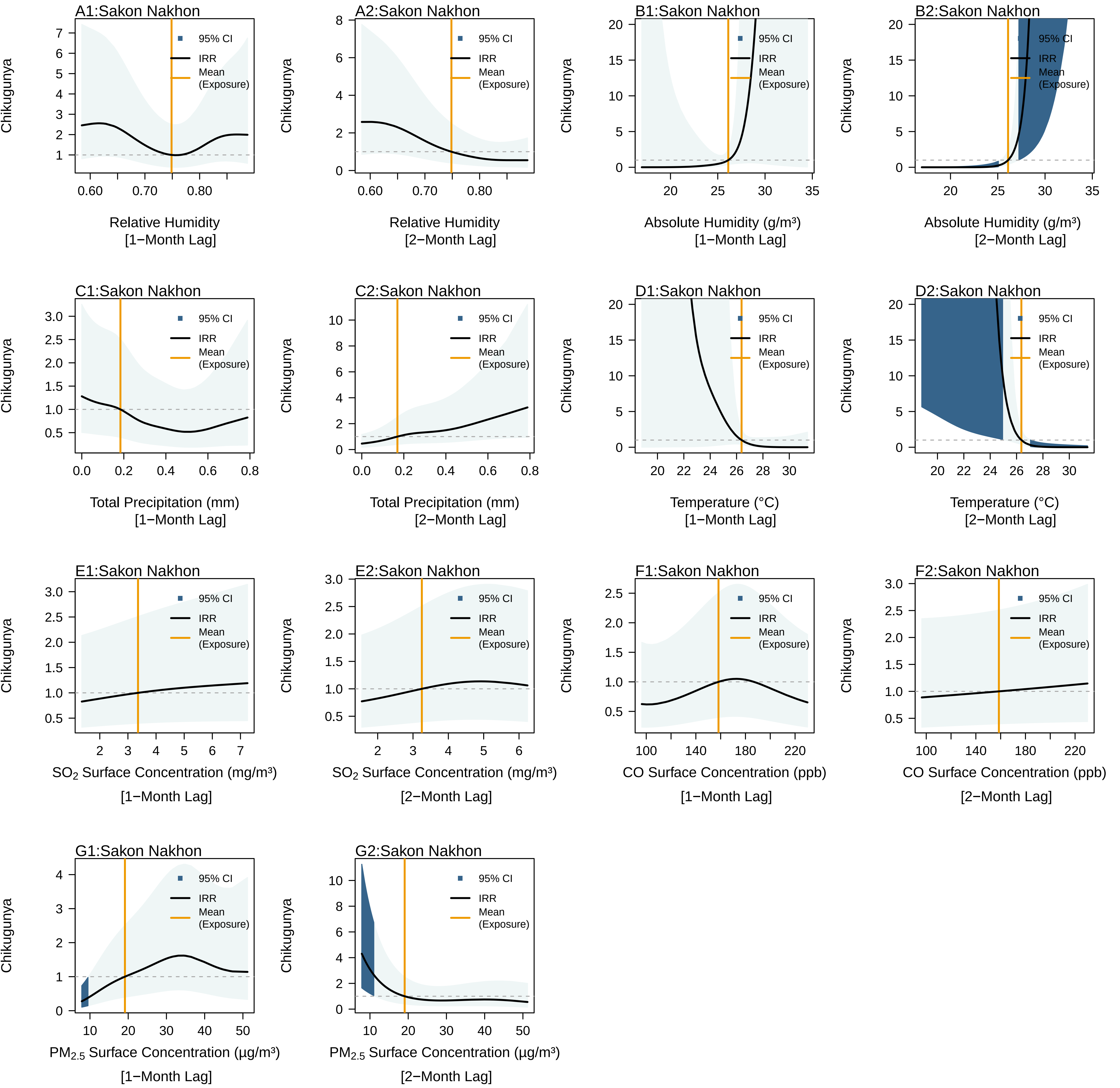
A1:Sakon Nakhon
Chikugunya
95% CI
IRR
Mean
(Exposure)
Relative Humidity
[1−Month Lag]
A2:Sakon Nakhon
Relative Humidity
[2−Month Lag]
B1:Sakon Nakhon
Absolute Humidity (g/m³)
[1−Month Lag]
B2:Sakon Nakhon
Absolute Humidity (g/m³)
[2−Month Lag]
C1:Sakon Nakhon
Total Precipitation (mm)
[1−Month Lag]
C2:Sakon Nakhon
Total Precipitation (mm)
[2−Month Lag]
D1:Sakon Nakhon
Temperature (°C)
[1−Month Lag]
D2:Sakon Nakhon
Temperature (°C)
[2−Month Lag]
E1:Sakon Nakhon
SO2 Surface Concentration (mg/m³)
[1−Month Lag]
E2:Sakon Nakhon
SO2 Surface Concentration (mg/m³)
[2−Month Lag]
F1:Sakon Nakhon
CO Surface Concentration (ppb)
[1−Month Lag]
F2:Sakon Nakhon
CO Surface Concentration (ppb)
[2−Month Lag]
G1:Sakon Nakhon
PM2.5 Surface Concentration (µg/m³)
[1−Month Lag]
G2:Sakon Nakhon
PM2.5 Surface Concentration (µg/m³)
[2−Month Lag]

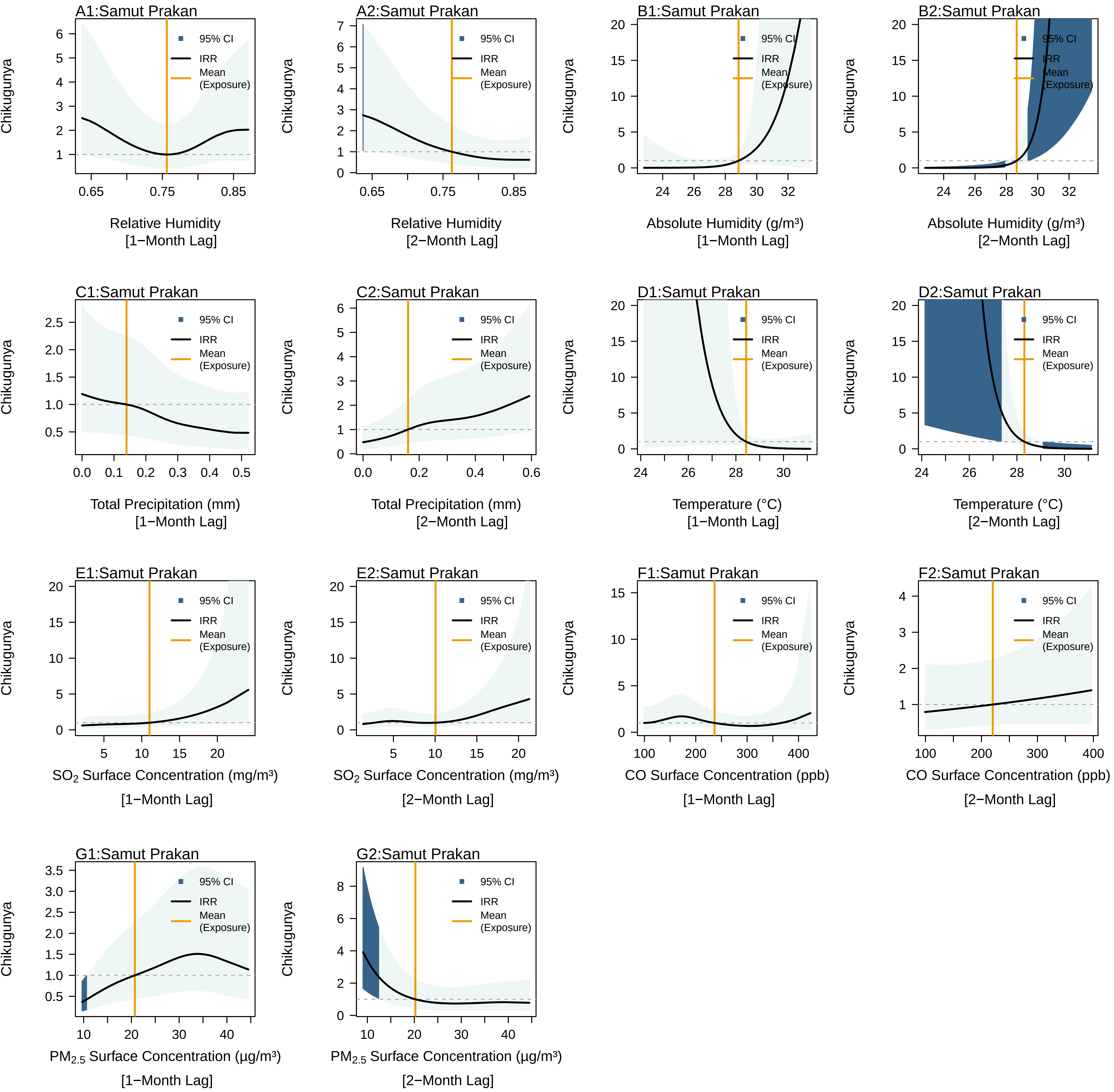
A1:Samut Prakan
A2:Samut Prakan
B1:Samut Prakan
B2:Samut Prakan
C1:Samut Prakan
C2:Samut Prakan
D1:Samut Prakan
D2:Samut Prakan
E1:Samut Prakan
E2:Samut Prakan
F1:Samut Prakan
F2:Samut Prakan
G1:Samut Prakan
G2:Samut Prakan
Chikugunya
95% CI
IRR
Mean (Exposure)
Relative Humidity [1−Month Lag]
Relative Humidity [2−Month Lag]
Absolute Humidity (g/m³) [1−Month Lag]
Absolute Humidity (g/m³) [2−Month Lag]
Total Precipitation (mm) [1−Month Lag]
Total Precipitation (mm) [2−Month Lag]
Temperature (°C) [1−Month Lag]
Temperature (°C) [2−Month Lag]
SO_2 Surface Concentration (mg/m³) [1−Month Lag]
SO_2 Surface Concentration (mg/m³) [2−Month Lag]
CO Surface Concentration (ppb) [1−Month Lag]
CO Surface Concentration (ppb) [2−Month Lag]
$PM_{2.5}$ Surface Concentration (µg/m³) [1−Month Lag]
$PM_{2.5}$ Surface Concentration (µg/m³) [2−Month Lag]

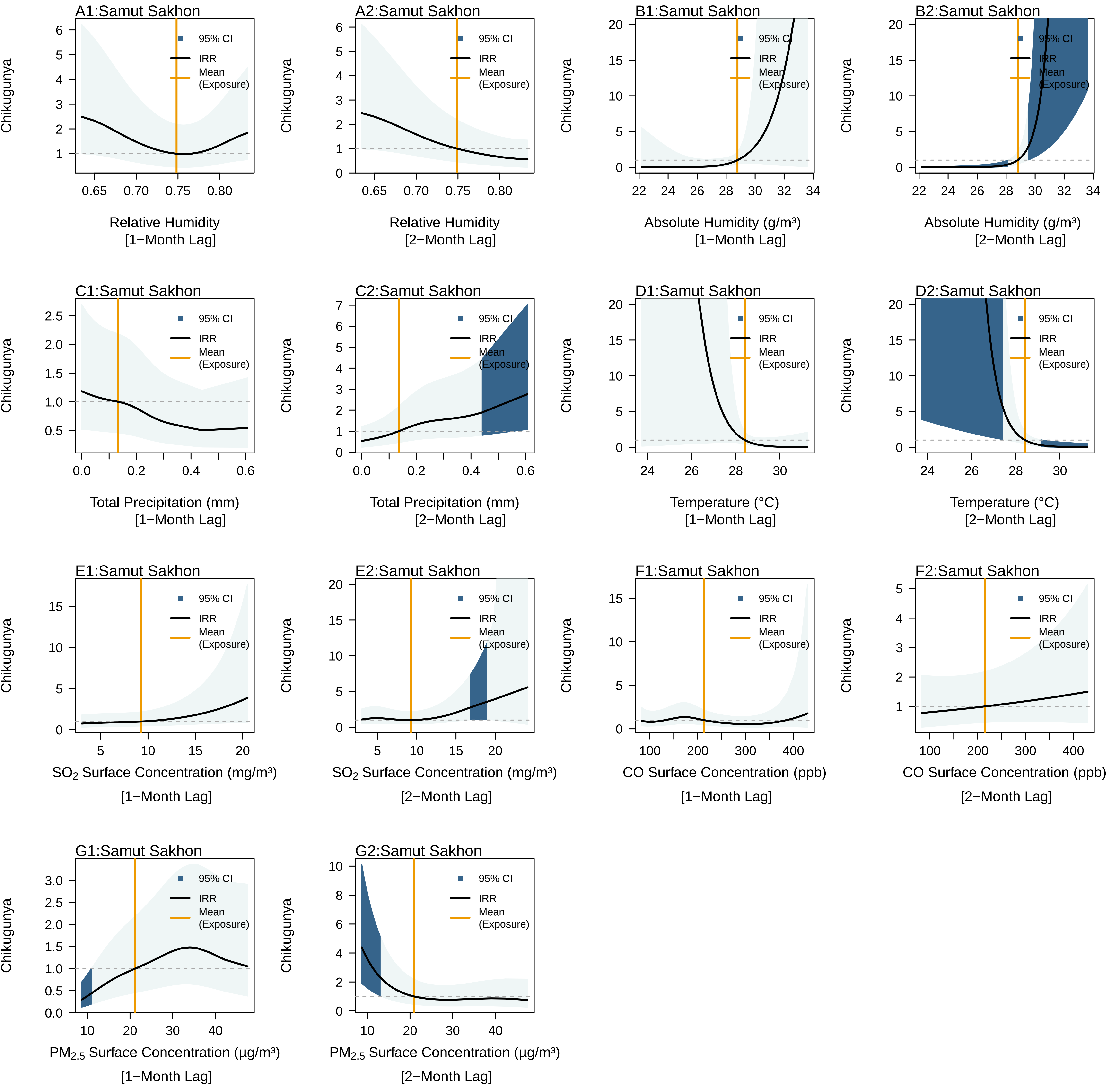

A1:Samut Sakhon
Chikugunya
95% CI
IRR
Mean (Exposure)
Relative Humidity [1−Month Lag]
A2:Samut Sakhon
Chikugunya
95% CI
IRR
Mean (Exposure)
Relative Humidity [2−Month Lag]
B1:Samut Sakhon
Chikugunya
95% CI
IRR
Mean (Exposure)
Absolute Humidity (g/m³) [1−Month Lag]
B2:Samut Sakhon
Chikugunya
95% CI
IRR
Mean (Exposure)
Absolute Humidity (g/m³) [2−Month Lag]
C1:Samut Sakhon
Chikugunya
95% CI
IRR
Mean (Exposure)
Total Precipitation (mm) [1−Month Lag]
C2:Samut Sakhon
Chikugunya
95% CI
IRR
Mean (Exposure)
Total Precipitation (mm) [2−Month Lag]
D1:Samut Sakhon
Chikugunya
95% CI
IRR
Mean (Exposure)
Temperature (°C) [1−Month Lag]
D2:Samut Sakhon
Chikugunya
95% CI
IRR
Mean (Exposure)
Temperature (°C) [2−Month Lag]
E1:Samut Sakhon
Chikugunya
95% CI
IRR
Mean (Exposure)
SO2 Surface Concentration (mg/m³) [1−Month Lag]
E2:Samut Sakhon
Chikugunya
95% CI
IRR
Mean (Exposure)
SO2 Surface Concentration (mg/m³) [2−Month Lag]
F1:Samut Sakhon
Chikugunya
95% CI
IRR
Mean (Exposure)
CO Surface Concentration (ppb) [1−Month Lag]
F2:Samut Sakhon
Chikugunya
95% CI
IRR
Mean (Exposure)
CO Surface Concentration (ppb) [2−Month Lag]
G1:Samut Sakhon
Chikugunya
95% CI
IRR
Mean (Exposure)
PM2.5 Surface Concentration (μg/m³) [1−Month Lag]
G2:Samut Sakhon
Chikugunya
95% CI
IRR
Mean (Exposure)
PM2.5 Surface Concentration (μg/m³) [2−Month Lag]

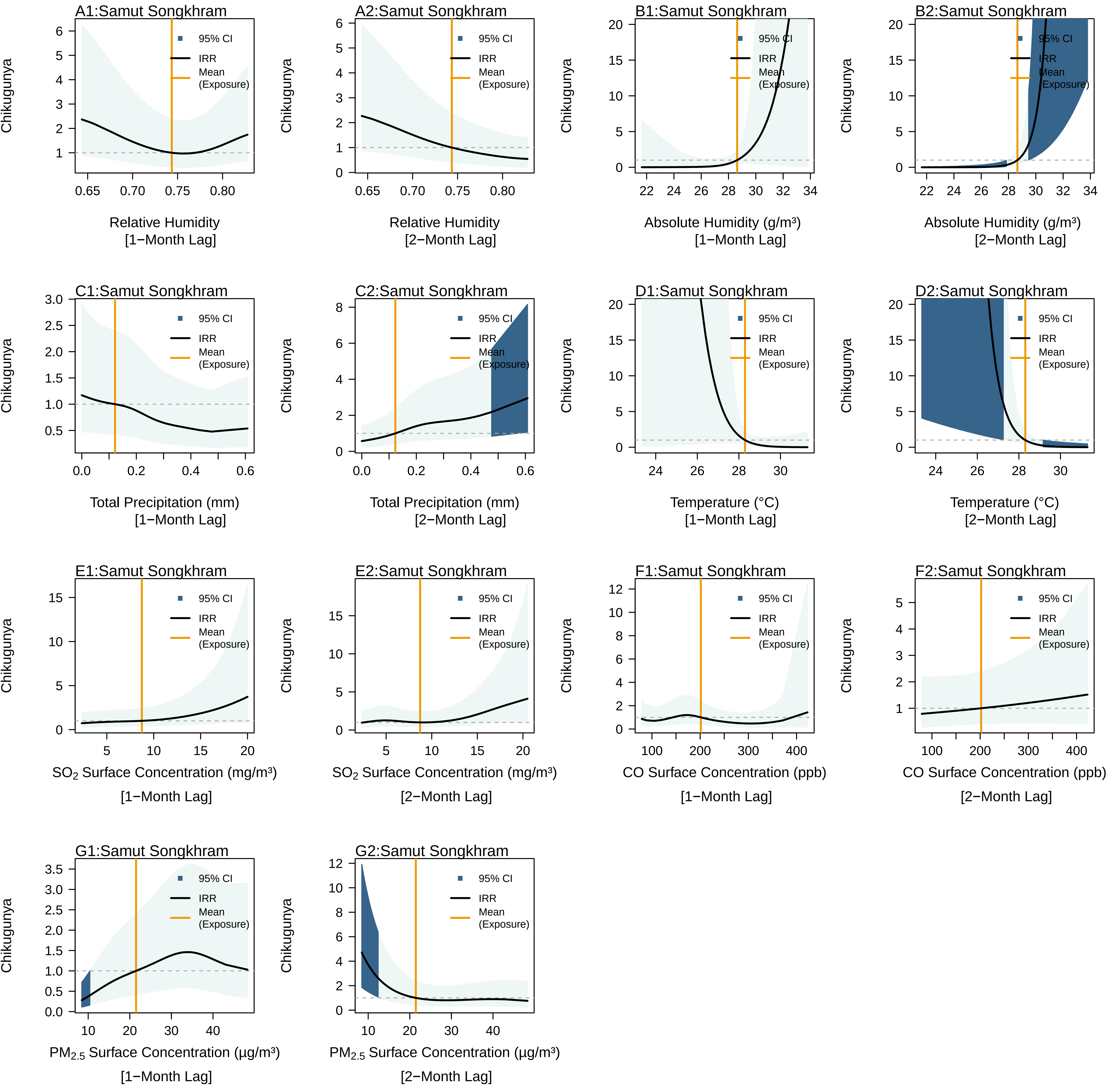

A1:Samut Songkhram
A2:Samut Songkhram
B1:Samut Songkhram
B2:Samut Songkhram
C1:Samut Songkhram
C2:Samut Songkhram
D1:Samut Songkhram
D2:Samut Songkhram
E1:Samut Songkhram
E2:Samut Songkhram
F1:Samut Songkhram
F2:Samut Songkhram
G1:Samut Songkhram
G2:Samut Songkhram
Chikugunya
95% CI
IRR
Mean (Exposure)
Relative Humidity [1−Month Lag]
Relative Humidity [2−Month Lag]
Absolute Humidity (g/m³) [1−Month Lag]
Absolute Humidity (g/m³) [2−Month Lag]
Total Precipitation (mm) [1−Month Lag]
Total Precipitation (mm) [2−Month Lag]
Temperature (°C) [1−Month Lag]
Temperature (°C) [2−Month Lag]
SO_2 Surface Concentration (mg/m³) [1−Month Lag]
SO_2 Surface Concentration (mg/m³) [2−Month Lag]
CO Surface Concentration (ppb) [1−Month Lag]
CO Surface Concentration (ppb) [2−Month Lag]
$PM_{2.5}$ Surface Concentration (µg/m³) [1−Month Lag]
$PM_{2.5}$ Surface Concentration (µg/m³) [2−Month Lag]

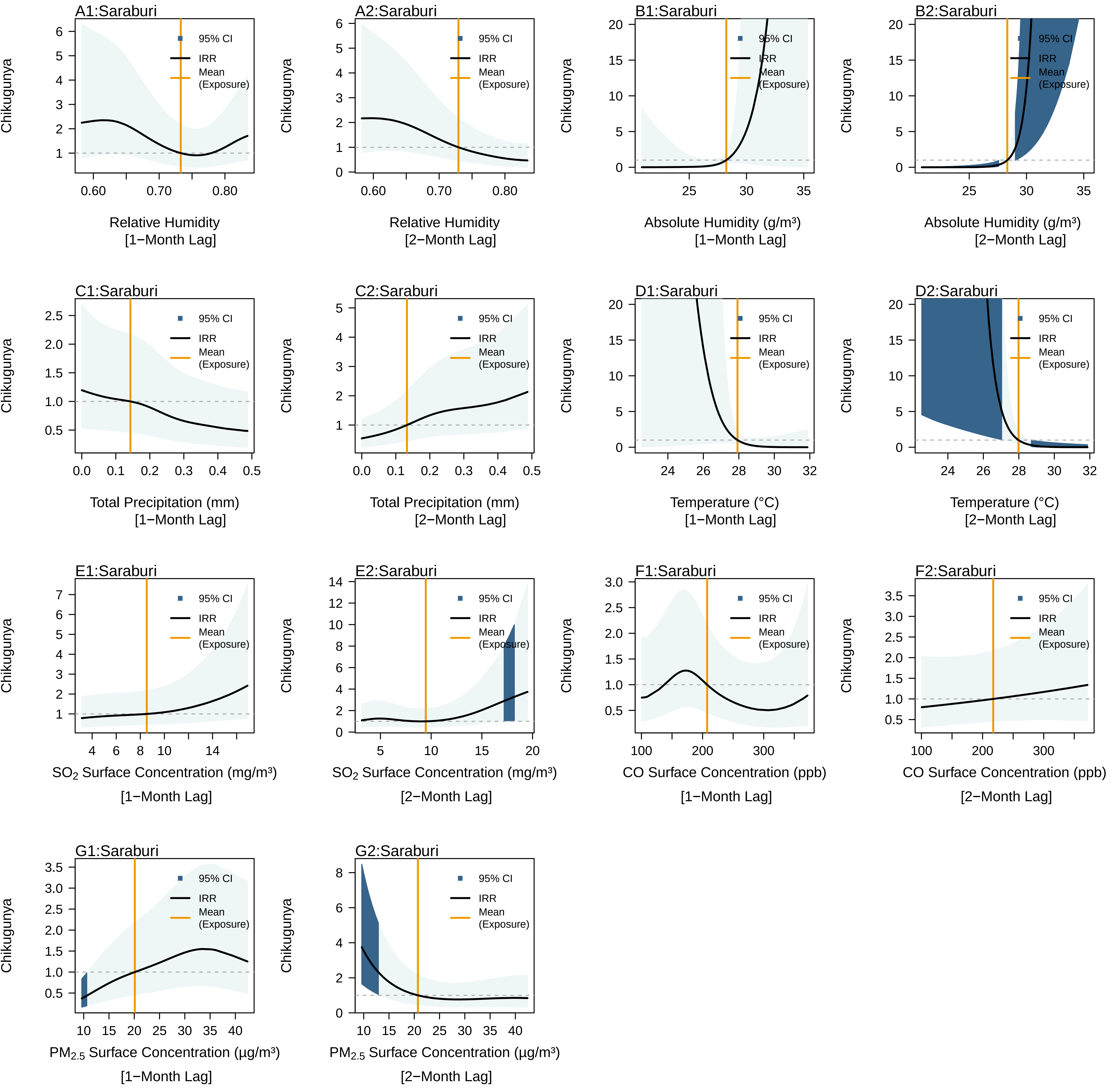
A1:Saraburi
A2:Saraburi
B1:Saraburi
B2:Saraburi
Chikugunya
95% CI
IRR
Mean
(Exposure)
Relative Humidity
[1−Month Lag]
Relative Humidity
[2−Month Lag]
Absolute Humidity (g/m³)
[1−Month Lag]
Absolute Humidity (g/m³)
[2−Month Lag]
C1:Saraburi
C2:Saraburi
D1:Saraburi
D2:Saraburi
Total Precipitation (mm)
[1−Month Lag]
Total Precipitation (mm)
[2−Month Lag]
Temperature (°C)
[1−Month Lag]
Temperature (°C)
[2−Month Lag]
E1:Saraburi
E2:Saraburi
F1:Saraburi
F2:Saraburi
SO2 Surface Concentration (mg/m³)
[1−Month Lag]
SO2 Surface Concentration (mg/m³)
[2−Month Lag]
CO Surface Concentration (ppb)
[1−Month Lag]
CO Surface Concentration (ppb)
[2−Month Lag]
G1:Saraburi
G2:Saraburi
PM2.5 Surface Concentration (µg/m³)
[1−Month Lag]
PM2.5 Surface Concentration (µg/m³)
[2−Month Lag]

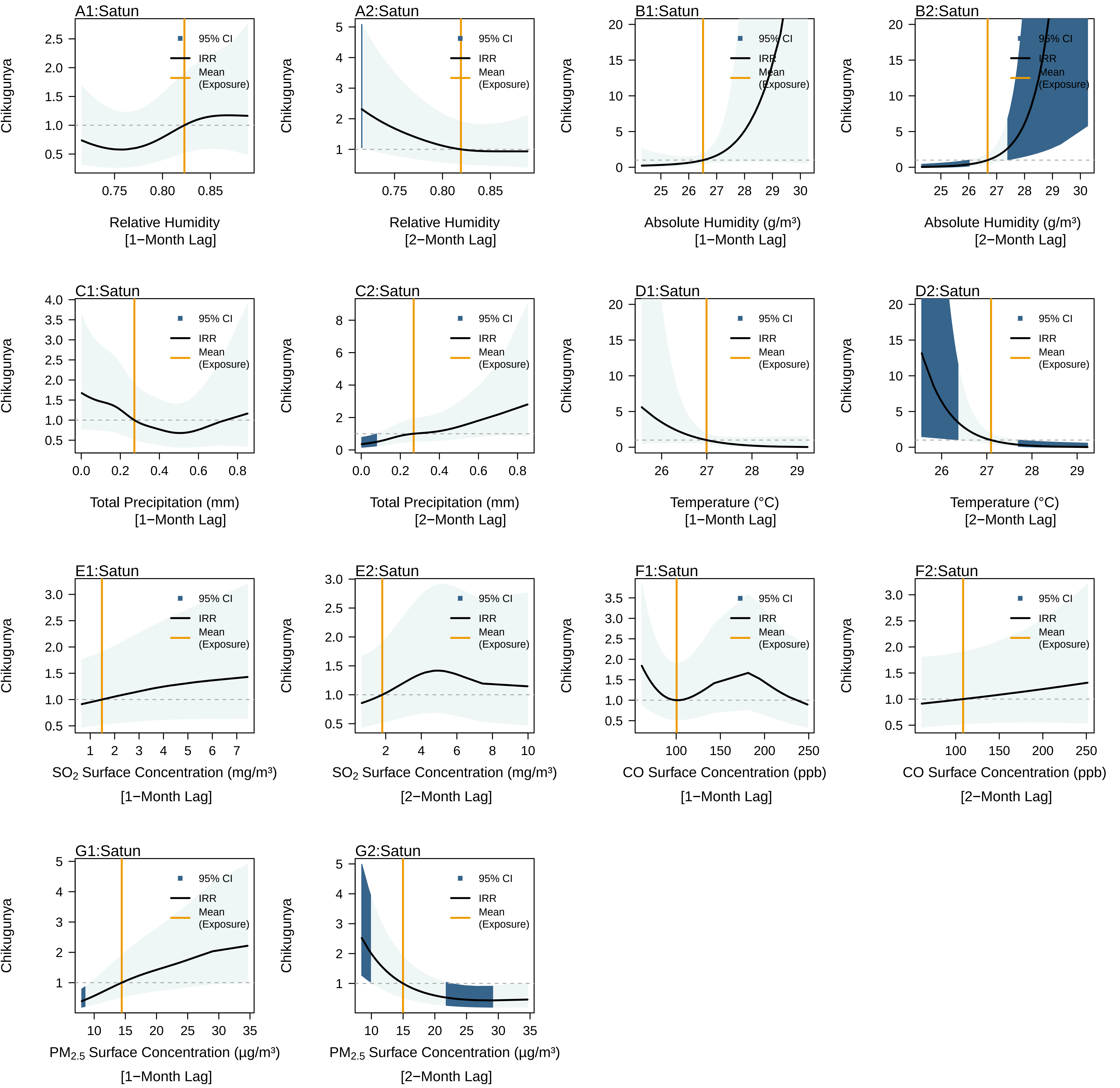
A1:Satun
A2:Satun
B1:Satun
B2:Satun
C1:Satun
C2:Satun
D1:Satun
D2:Satun
E1:Satun
E2:Satun
F1:Satun
F2:Satun
G1:Satun
G2:Satun
Chikugunya
95% CI
IRR
Mean (Exposure)
Relative Humidity [1−Month Lag]
Relative Humidity [2−Month Lag]
Absolute Humidity (g/m³) [1−Month Lag]
Absolute Humidity (g/m³) [2−Month Lag]
Total Precipitation (mm) [1−Month Lag]
Total Precipitation (mm) [2−Month Lag]
Temperature (°C) [1−Month Lag]
Temperature (°C) [2−Month Lag]
SO2 Surface Concentration (mg/m³) [1−Month Lag]
SO2 Surface Concentration (mg/m³) [2−Month Lag]
CO Surface Concentration (ppb) [1−Month Lag]
CO Surface Concentration (ppb) [2−Month Lag]
PM2.5 Surface Concentration (µg/m³) [1−Month Lag]
PM2.5 Surface Concentration (µg/m³) [2−Month Lag]

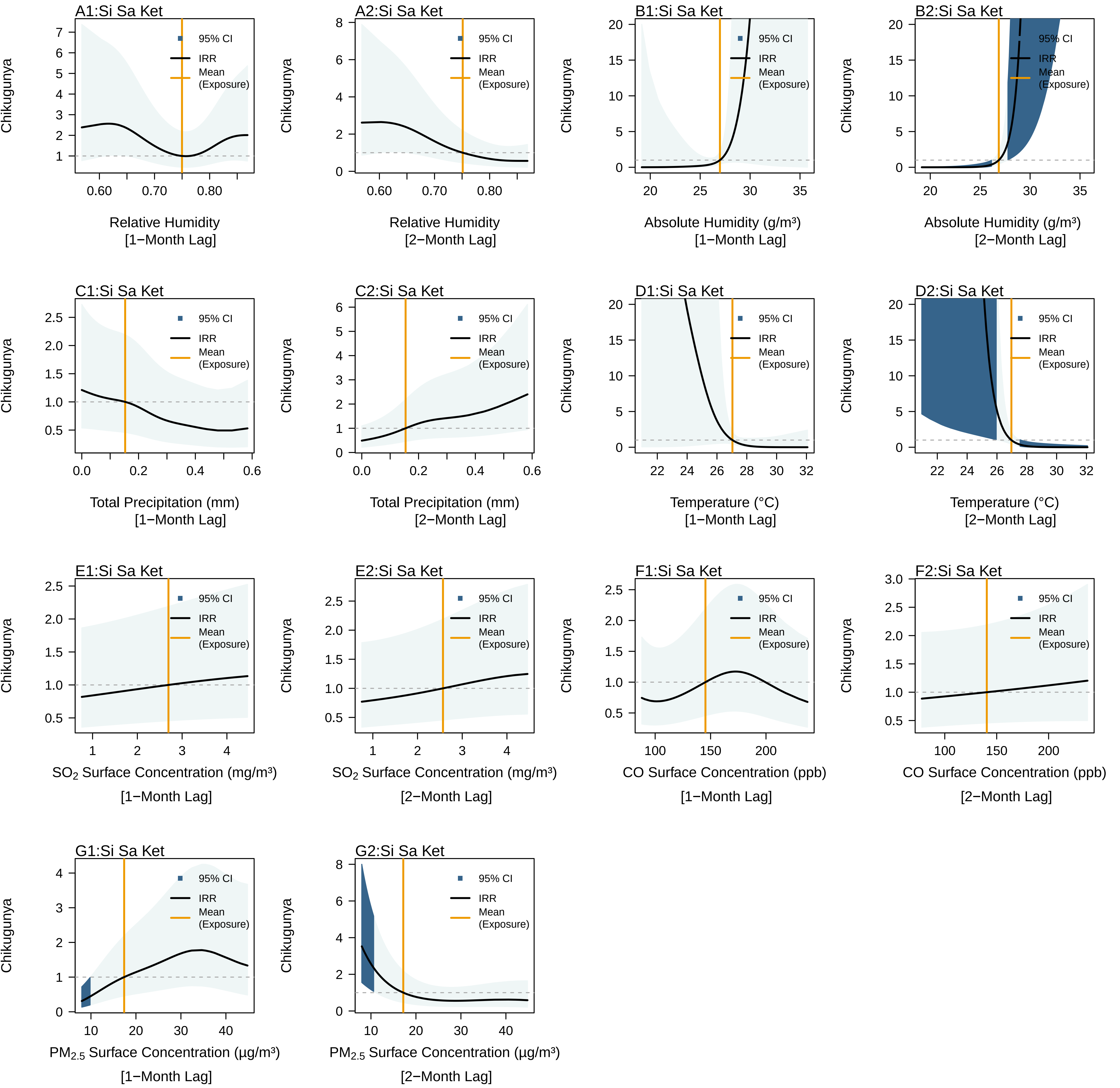
A1:Si Sa Ket
A2:Si Sa Ket
B1:Si Sa Ket
B2:Si Sa Ket
C1:Si Sa Ket
C2:Si Sa Ket
D1:Si Sa Ket
D2:Si Sa Ket
E1:Si Sa Ket
E2:Si Sa Ket
F1:Si Sa Ket
F2:Si Sa Ket
G1:Si Sa Ket
G2:Si Sa Ket
Chikugunya
95% CI
IRR
Mean
(Exposure)
Relative Humidity
[1−Month Lag]
Relative Humidity
[2−Month Lag]
Absolute Humidity (g/m³)
[1−Month Lag]
Absolute Humidity (g/m³)
[2−Month Lag]
Total Precipitation (mm)
[1−Month Lag]
Total Precipitation (mm)
[2−Month Lag]
Temperature (°C)
[1−Month Lag]
Temperature (°C)
[2−Month Lag]
SO_2 Surface Concentration (mg/m³)
[1−Month Lag]
SO_2 Surface Concentration (mg/m³)
[2−Month Lag]
CO Surface Concentration (ppb)
[1−Month Lag]
CO Surface Concentration (ppb)
[2−Month Lag]
$PM_{2.5}$ Surface Concentration (µg/m³)
[1−Month Lag]
$PM_{2.5}$ Surface Concentration (µg/m³)
[2−Month Lag]

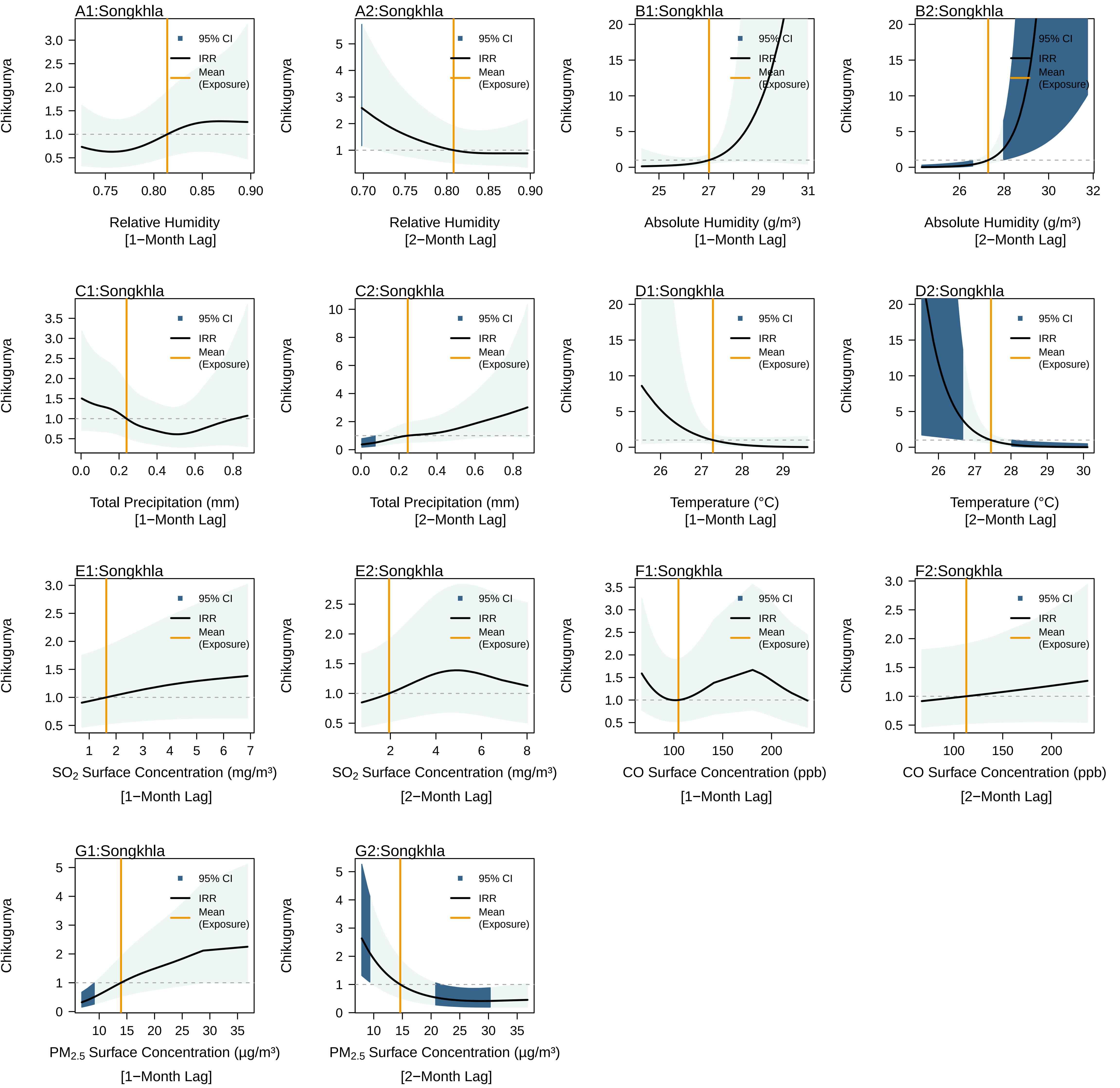
A1:Songkhla
Chikugunya
95% CI
IRR
Mean (Exposure)
Relative Humidity [1−Month Lag]
A2:Songkhla
Chikugunya
Relative Humidity [2−Month Lag]
B1:Songkhla
Chikugunya
Absolute Humidity (g/m³) [1−Month Lag]
B2:Songkhla
Chikugunya
Absolute Humidity (g/m³) [2−Month Lag]
C1:Songkhla
Chikugunya
Total Precipitation (mm) [1−Month Lag]
C2:Songkhla
Chikugunya
Total Precipitation (mm) [2−Month Lag]
D1:Songkhla
Chikugunya
Temperature (°C) [1−Month Lag]
D2:Songkhla
Chikugunya
Temperature (°C) [2−Month Lag]
E1:Songkhla
Chikugunya
SO_2 Surface Concentration (mg/m³) [1−Month Lag]
E2:Songkhla
Chikugunya
SO_2 Surface Concentration (mg/m³) [2−Month Lag]
F1:Songkhla
Chikugunya
CO Surface Concentration (ppb) [1−Month Lag]
F2:Songkhla
Chikugunya
CO Surface Concentration (ppb) [2−Month Lag]
G1:Songkhla
Chikugunya
$PM_{2.5}$ Surface Concentration (µg/m³) [1−Month Lag]
G2:Songkhla
Chikugunya
$PM_{2.5}$ Surface Concentration (µg/m³) [2−Month Lag]

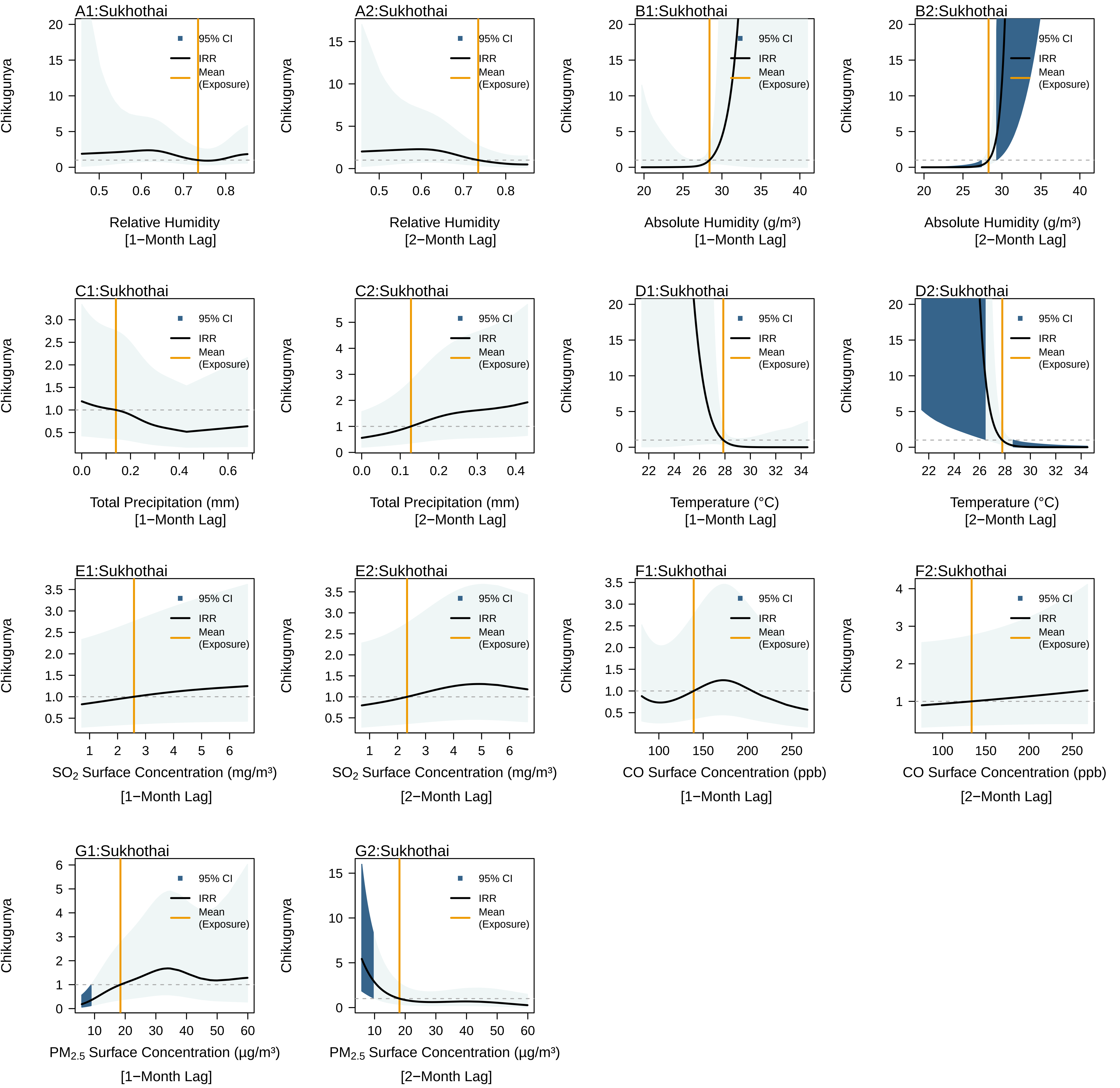

A1:Sukhothai
A2:Sukhothai
B1:Sukhothai
B2:Sukhothai
C1:Sukhothai
C2:Sukhothai
D1:Sukhothai
D2:Sukhothai
E1:Sukhothai
E2:Sukhothai
F1:Sukhothai
F2:Sukhothai
G1:Sukhothai
G2:Sukhothai
Chikugunya
95% CI
IRR
Mean (Exposure)
Relative Humidity [1−Month Lag]
Relative Humidity [2−Month Lag]
Absolute Humidity (g/m³) [1−Month Lag]
Absolute Humidity (g/m³) [2−Month Lag]
Total Precipitation (mm) [1−Month Lag]
Total Precipitation (mm) [2−Month Lag]
Temperature (°C) [1−Month Lag]
Temperature (°C) [2−Month Lag]
SO2 Surface Concentration (mg/m³) [1−Month Lag]
SO2 Surface Concentration (mg/m³) [2−Month Lag]
CO Surface Concentration (ppb) [1−Month Lag]
CO Surface Concentration (ppb) [2−Month Lag]
PM2.5 Surface Concentration (µg/m³) [1−Month Lag]
PM2.5 Surface Concentration (µg/m³) [2−Month Lag]

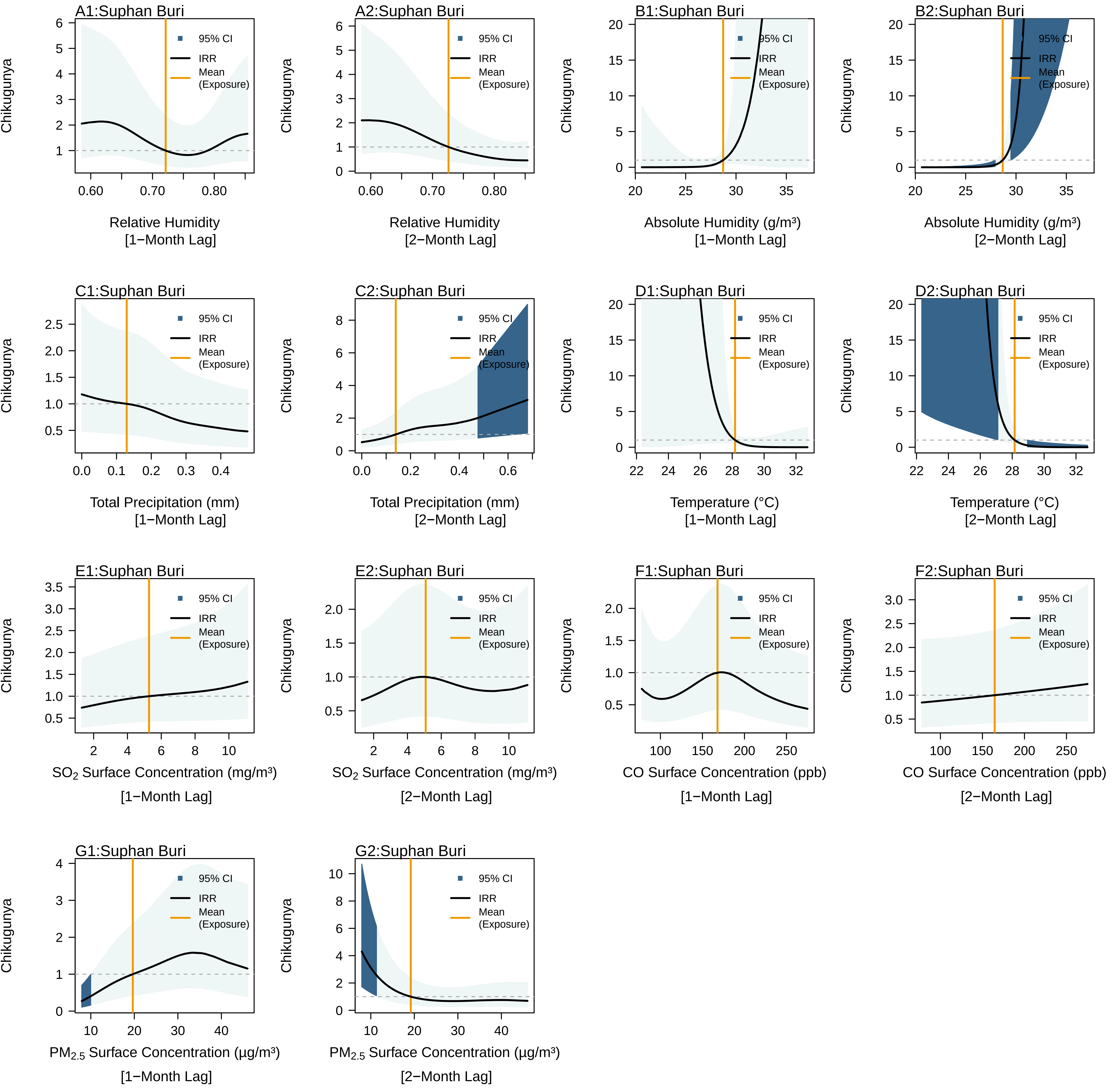
A1:Suphan Buri
Relative Humidity [1−Month Lag]
A2:Suphan Buri
Relative Humidity [2−Month Lag]
B1:Suphan Buri
Absolute Humidity (g/m³) [1−Month Lag]
B2:Suphan Buri
Absolute Humidity (g/m³) [2−Month Lag]
C1:Suphan Buri
Total Precipitation (mm) [1−Month Lag]
C2:Suphan Buri
Total Precipitation (mm) [2−Month Lag]
D1:Suphan Buri
Temperature (°C) [1−Month Lag]
D2:Suphan Buri
Temperature (°C) [2−Month Lag]
E1:Suphan Buri
SO2 Surface Concentration (mg/m³) [1−Month Lag]
E2:Suphan Buri
SO2 Surface Concentration (mg/m³) [2−Month Lag]
F1:Suphan Buri
CO Surface Concentration (ppb) [1−Month Lag]
F2:Suphan Buri
CO Surface Concentration (ppb) [2−Month Lag]
G1:Suphan Buri
PM2.5 Surface Concentration (µg/m³) [1−Month Lag]
G2:Suphan Buri
PM2.5 Surface Concentration (µg/m³) [2−Month Lag]
Chikugunya
95% CI
IRR
Mean (Exposure)

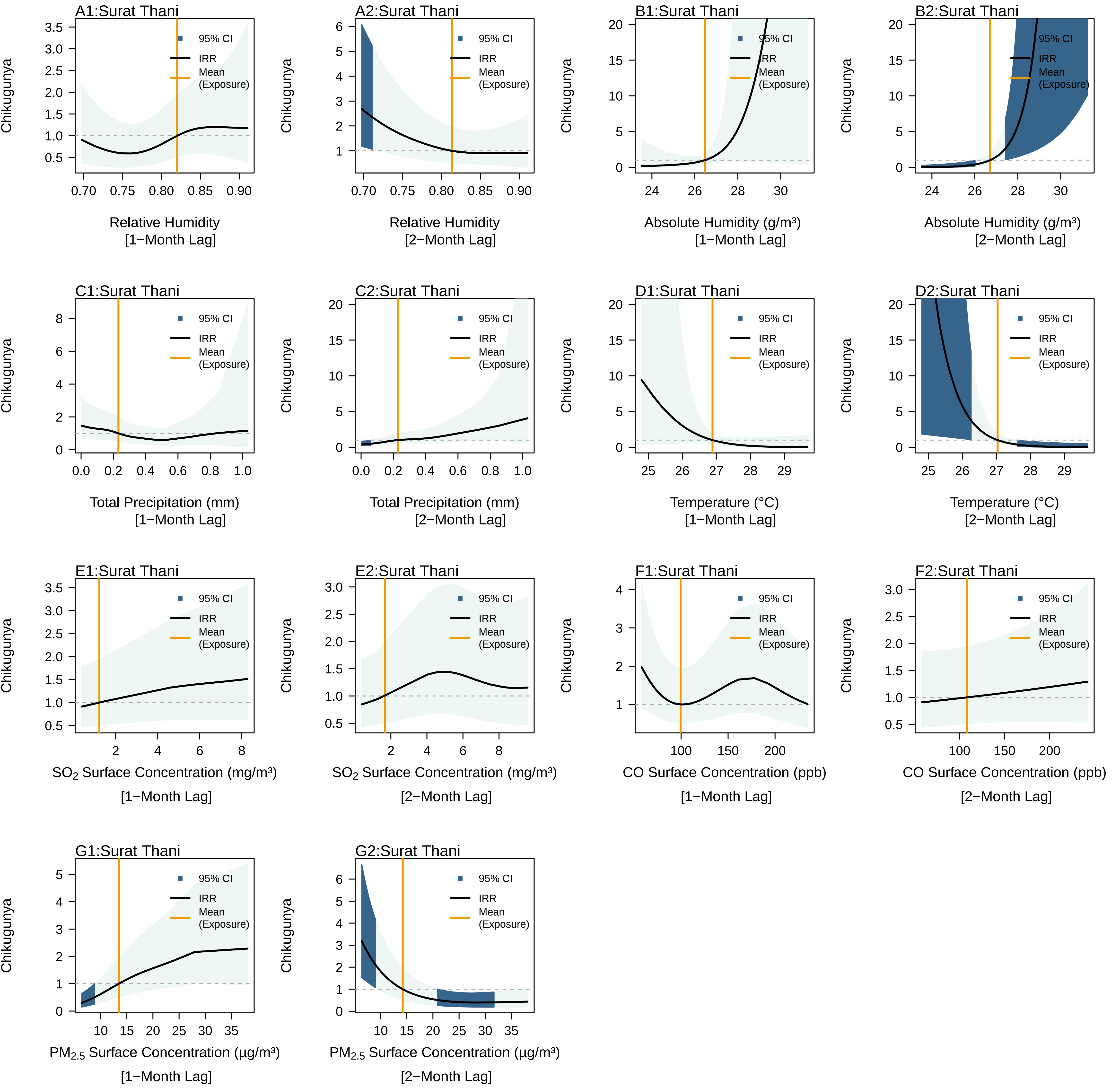
A1:Surat Thani
Chikugunya
95% CI
IRR
Mean (Exposure)
Relative Humidity [1−Month Lag]
A2:Surat Thani
Chikugunya
95% CI
IRR
Mean (Exposure)
Relative Humidity [2−Month Lag]
B1:Surat Thani
Chikugunya
95% CI
IRR
Mean (Exposure)
Absolute Humidity (g/m³) [1−Month Lag]
B2:Surat Thani
Chikugunya
95% CI
IRR
Mean (Exposure)
Absolute Humidity (g/m³) [2−Month Lag]
C1:Surat Thani
Chikugunya
95% CI
IRR
Mean (Exposure)
Total Precipitation (mm) [1−Month Lag]
C2:Surat Thani
Chikugunya
95% CI
IRR
Mean (Exposure)
Total Precipitation (mm) [2−Month Lag]
D1:Surat Thani
Chikugunya
95% CI
IRR
Mean (Exposure)
Temperature (°C) [1−Month Lag]
D2:Surat Thani
Chikugunya
95% CI
IRR
Mean (Exposure)
Temperature (°C) [2−Month Lag]
E1:Surat Thani
Chikugunya
95% CI
IRR
Mean (Exposure)
SO2 Surface Concentration (mg/m³) [1−Month Lag]
E2:Surat Thani
Chikugunya
95% CI
IRR
Mean (Exposure)
SO2 Surface Concentration (mg/m³) [2−Month Lag]
F1:Surat Thani
Chikugunya
95% CI
IRR
Mean (Exposure)
CO Surface Concentration (ppb) [1−Month Lag]
F2:Surat Thani
Chikugunya
95% CI
IRR
Mean (Exposure)
CO Surface Concentration (ppb) [2−Month Lag]
G1:Surat Thani
Chikugunya
95% CI
IRR
Mean (Exposure)
PM2.5 Surface Concentration (µg/m³) [1−Month Lag]
G2:Surat Thani
Chikugunya
95% CI
IRR
Mean (Exposure)
PM2.5 Surface Concentration (µg/m³) [2−Month Lag]

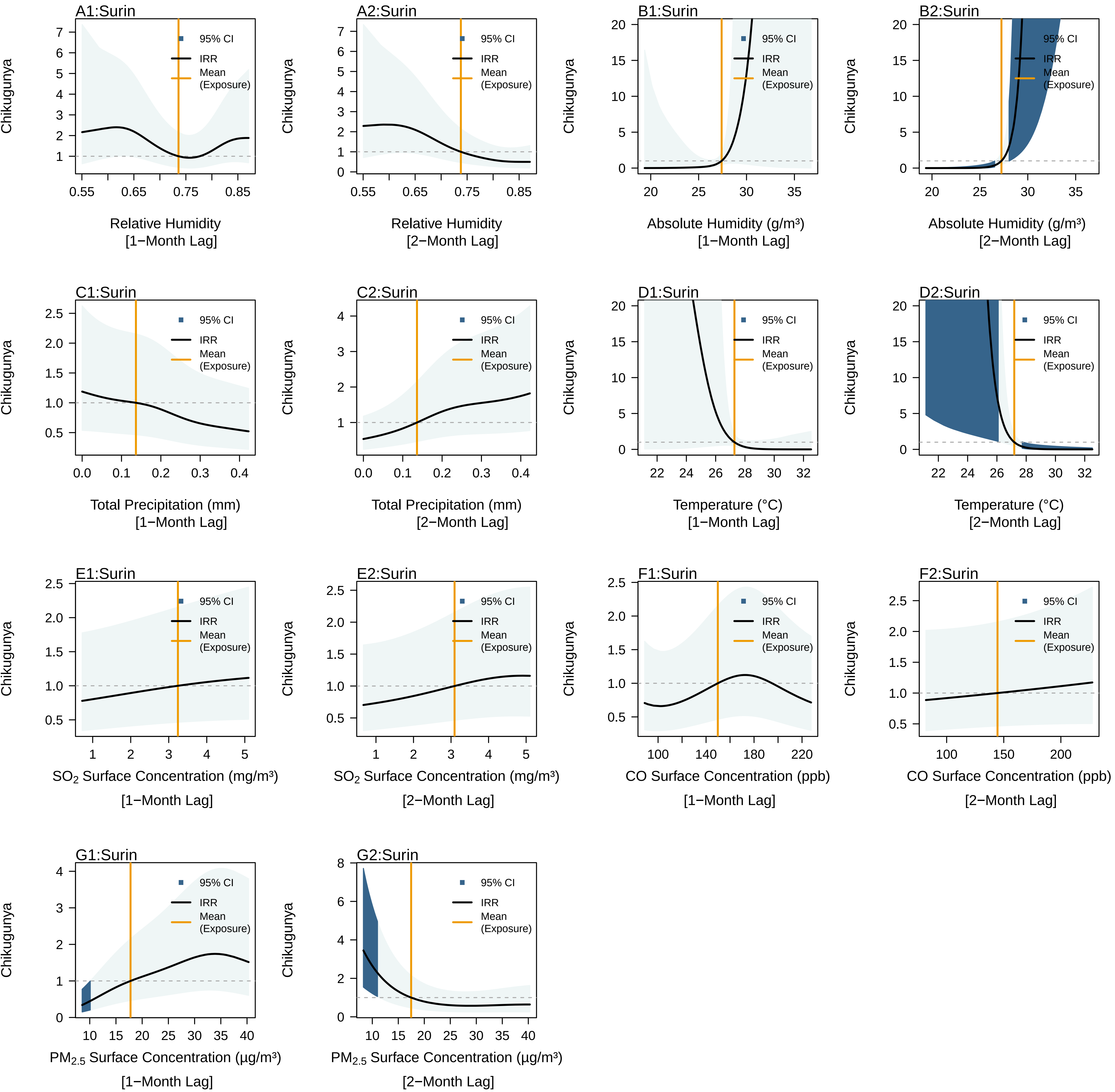

A1:Surin
A2:Surin
B1:Surin
B2:Surin
C1:Surin
C2:Surin
D1:Surin
D2:Surin
E1:Surin
E2:Surin
F1:Surin
F2:Surin
G1:Surin
G2:Surin
Chikugunya
95% CI
IRR
Mean (Exposure)
Relative Humidity [1−Month Lag]
Relative Humidity [2−Month Lag]
Absolute Humidity (g/m³) [1−Month Lag]
Absolute Humidity (g/m³) [2−Month Lag]
Total Precipitation (mm) [1−Month Lag]
Total Precipitation (mm) [2−Month Lag]
Temperature (°C) [1−Month Lag]
Temperature (°C) [2−Month Lag]
SO_2 Surface Concentration (mg/m³) [1−Month Lag]
SO_2 Surface Concentration (mg/m³) [2−Month Lag]
CO Surface Concentration (ppb) [1−Month Lag]
CO Surface Concentration (ppb) [2−Month Lag]
$PM_{2.5}$ Surface Concentration (µg/m³) [1−Month Lag]
$PM_{2.5}$ Surface Concentration (µg/m³) [2−Month Lag]

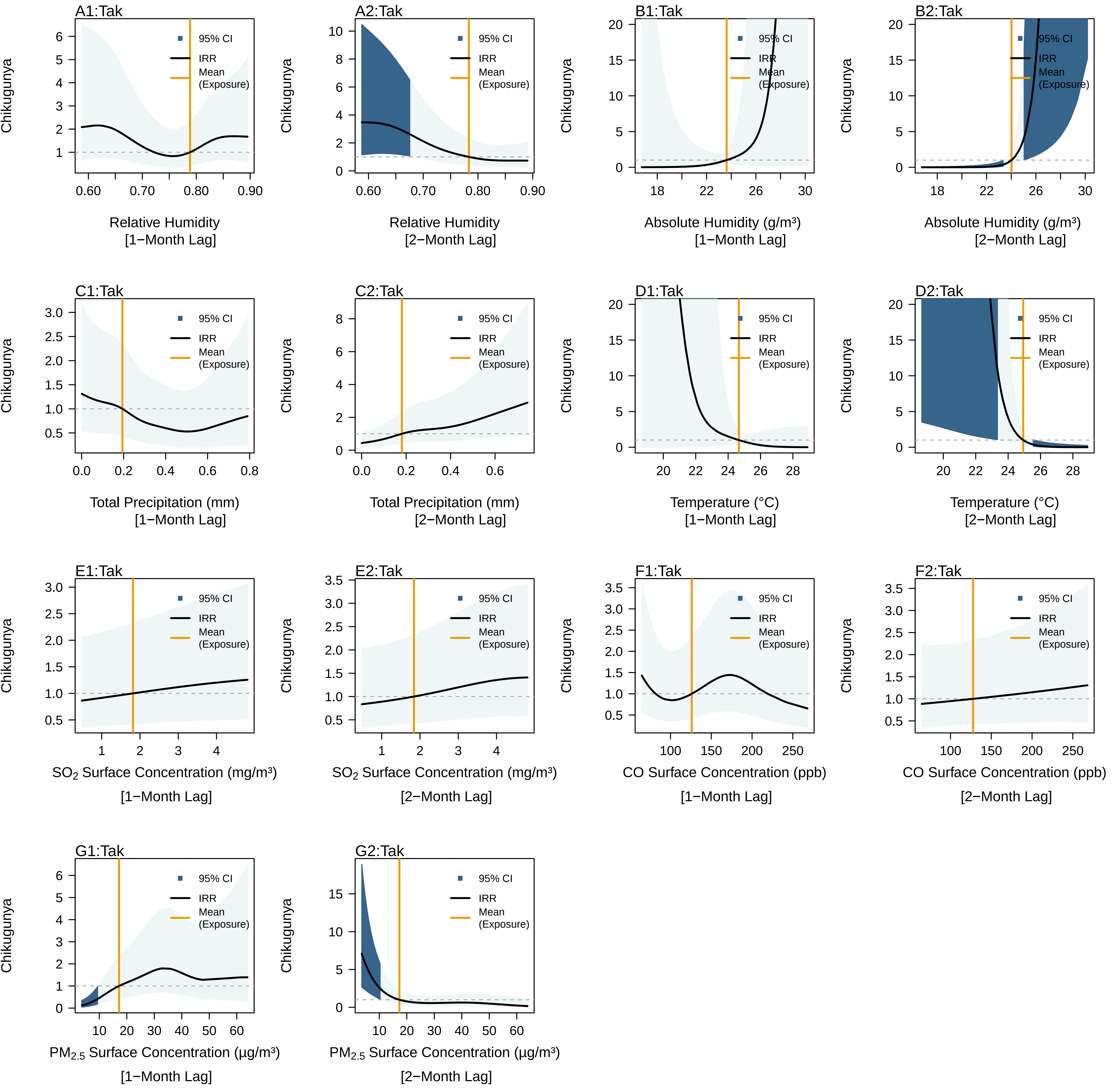
A1:Tak
A2:Tak
B1:Tak
B2:Tak
C1:Tak
C2:Tak
D1:Tak
D2:Tak
E1:Tak
E2:Tak
F1:Tak
F2:Tak
G1:Tak
G2:Tak
Chikugunya
95% CI
IRR
Mean (Exposure)
Relative Humidity [1−Month Lag]
Relative Humidity [2−Month Lag]
Absolute Humidity (g/m³) [1−Month Lag]
Absolute Humidity (g/m³) [2−Month Lag]
Total Precipitation (mm) [1−Month Lag]
Total Precipitation (mm) [2−Month Lag]
Temperature (°C) [1−Month Lag]
Temperature (°C) [2−Month Lag]
SO_2 Surface Concentration (mg/m³) [1−Month Lag]
SO_2 Surface Concentration (mg/m³) [2−Month Lag]
CO Surface Concentration (ppb) [1−Month Lag]
CO Surface Concentration (ppb) [2−Month Lag]
$PM_{2.5}$ Surface Concentration (µg/m³) [1−Month Lag]
$PM_{2.5}$ Surface Concentration (µg/m³) [2−Month Lag]

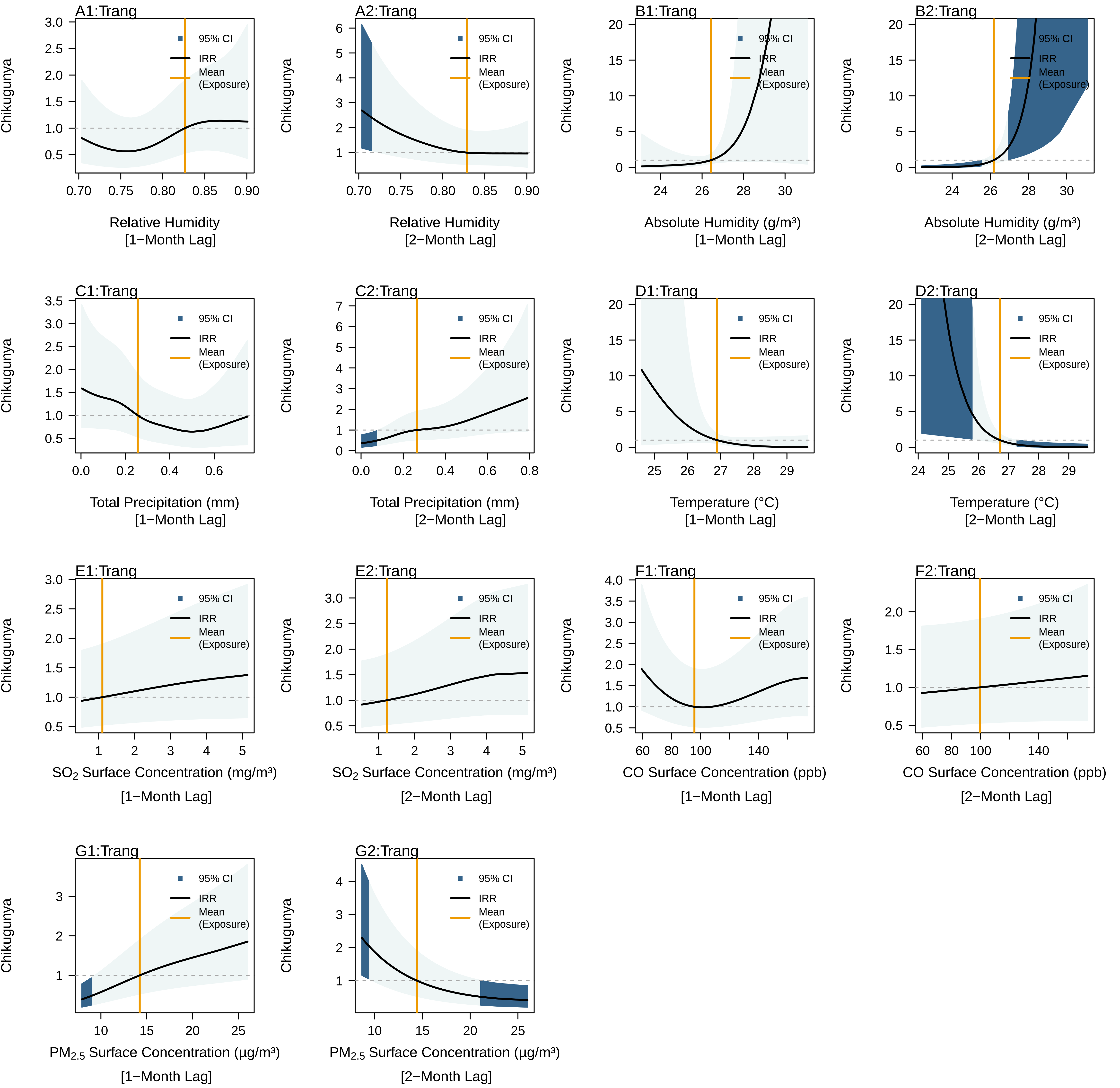

A1:Trang
A2:Trang
B1:Trang
B2:Trang
C1:Trang
C2:Trang
D1:Trang
D2:Trang
E1:Trang
E2:Trang
F1:Trang
F2:Trang
G1:Trang
G2:Trang
95% CI
IRR
Mean (Exposure)
Chikugunya
Relative Humidity [1−Month Lag]
Relative Humidity [2−Month Lag]
Absolute Humidity (g/m³) [1−Month Lag]
Absolute Humidity (g/m³) [2−Month Lag]
Total Precipitation (mm) [1−Month Lag]
Total Precipitation (mm) [2−Month Lag]
Temperature (°C) [1−Month Lag]
Temperature (°C) [2−Month Lag]
SO_2 Surface Concentration (mg/m³) [1−Month Lag]
SO_2 Surface Concentration (mg/m³) [2−Month Lag]
CO Surface Concentration (ppb) [1−Month Lag]
CO Surface Concentration (ppb) [2−Month Lag]
$PM_{2.5}$ Surface Concentration (μg/m³) [1−Month Lag]
$PM_{2.5}$ Surface Concentration (μg/m³) [2−Month Lag]

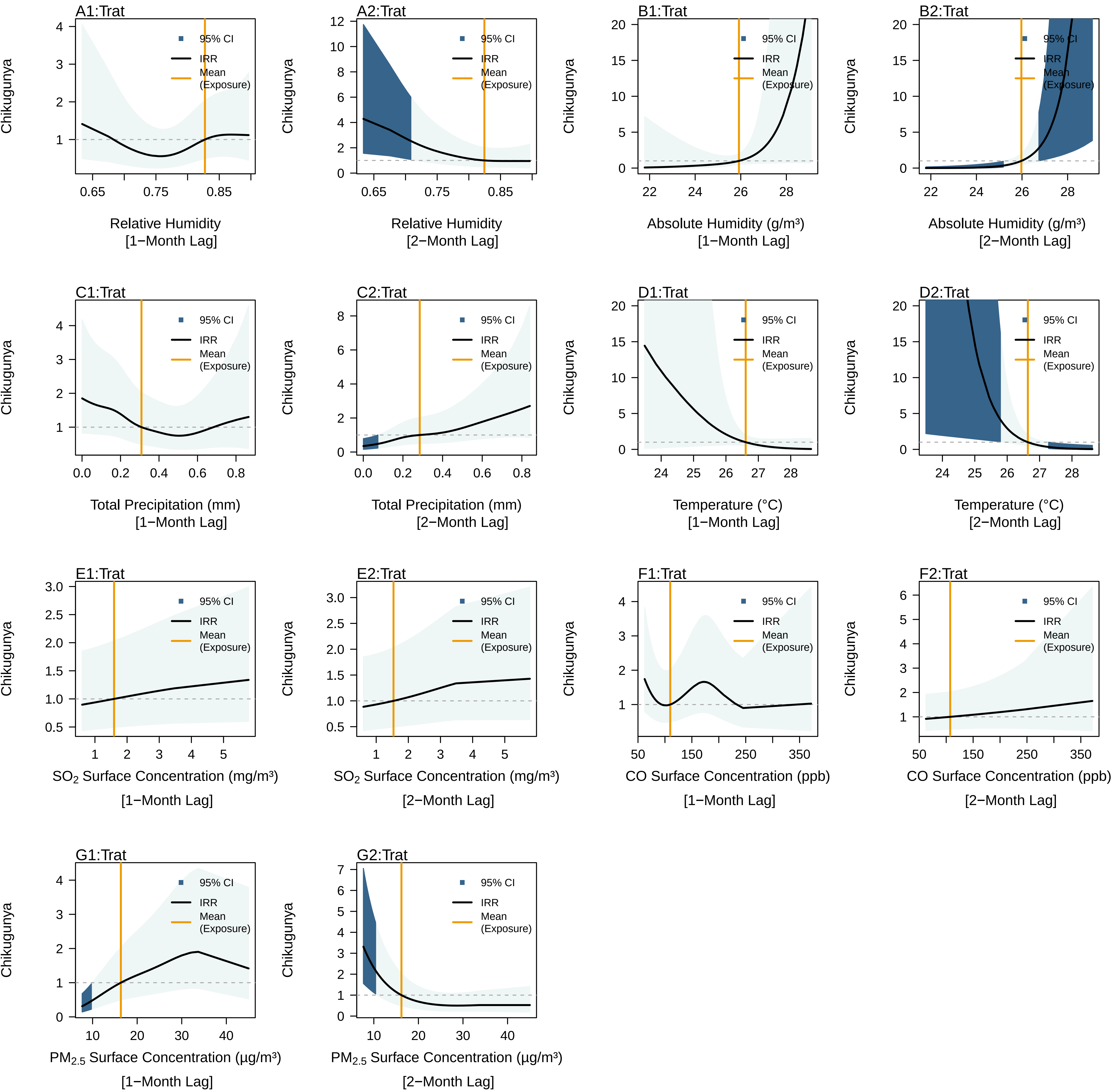

A1:Trat
A2:Trat
B1:Trat
B2:Trat
C1:Trat
C2:Trat
D1:Trat
D2:Trat
E1:Trat
E2:Trat
F1:Trat
F2:Trat
G1:Trat
G2:Trat
Chikugunya
95% CI
IRR
Mean (Exposure)
Relative Humidity [1−Month Lag]
Relative Humidity [2−Month Lag]
Absolute Humidity (g/m³) [1−Month Lag]
Absolute Humidity (g/m³) [2−Month Lag]
Total Precipitation (mm) [1−Month Lag]
Total Precipitation (mm) [2−Month Lag]
Temperature (°C) [1−Month Lag]
Temperature (°C) [2−Month Lag]
SO2 Surface Concentration (mg/m³) [1−Month Lag]
SO2 Surface Concentration (mg/m³) [2−Month Lag]
CO Surface Concentration (ppb) [1−Month Lag]
CO Surface Concentration (ppb) [2−Month Lag]
PM2.5 Surface Concentration (µg/m³) [1−Month Lag]
PM2.5 Surface Concentration (µg/m³) [2−Month Lag]

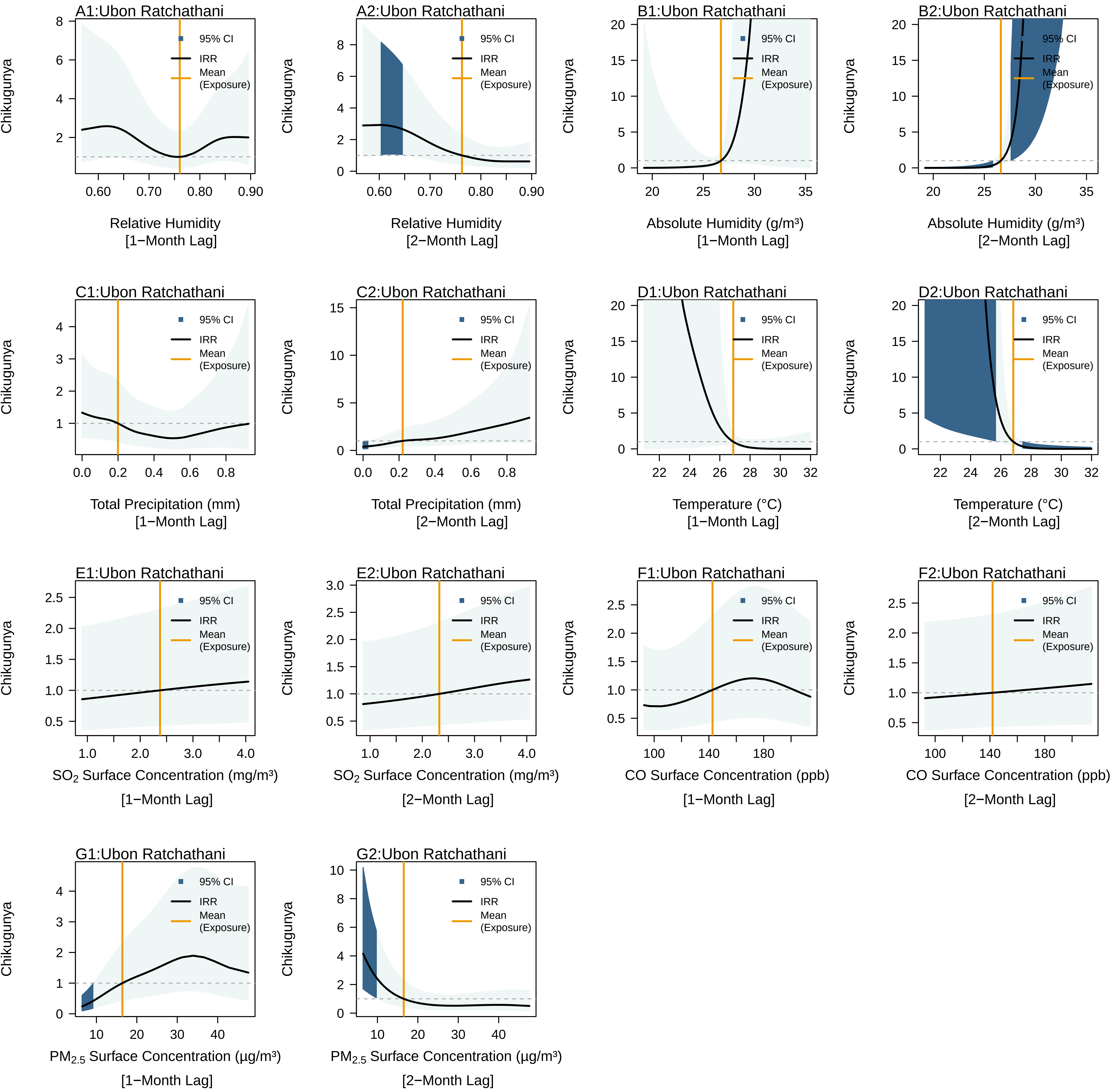
A1:Ubon Ratchathani
Chikugunya
95% CI
IRR
Mean
(Exposure)
Relative Humidity
[1−Month Lag]
A2:Ubon Ratchathani
Relative Humidity
[2−Month Lag]
B1:Ubon Ratchathani
Absolute Humidity (g/m³)
[1−Month Lag]
B2:Ubon Ratchathani
Absolute Humidity (g/m³)
[2−Month Lag]
C1:Ubon Ratchathani
Total Precipitation (mm)
[1−Month Lag]
C2:Ubon Ratchathani
Total Precipitation (mm)
[2−Month Lag]
D1:Ubon Ratchathani
Temperature (°C)
[1−Month Lag]
D2:Ubon Ratchathani
Temperature (°C)
[2−Month Lag]
E1:Ubon Ratchathani
SO2 Surface Concentration (mg/m³)
[1−Month Lag]
E2:Ubon Ratchathani
SO2 Surface Concentration (mg/m³)
[2−Month Lag]
F1:Ubon Ratchathani
CO Surface Concentration (ppb)
[1−Month Lag]
F2:Ubon Ratchathani
CO Surface Concentration (ppb)
[2−Month Lag]
G1:Ubon Ratchathani
PM2.5 Surface Concentration (µg/m³)
[1−Month Lag]
G2:Ubon Ratchathani
PM2.5 Surface Concentration (µg/m³)
[2−Month Lag]

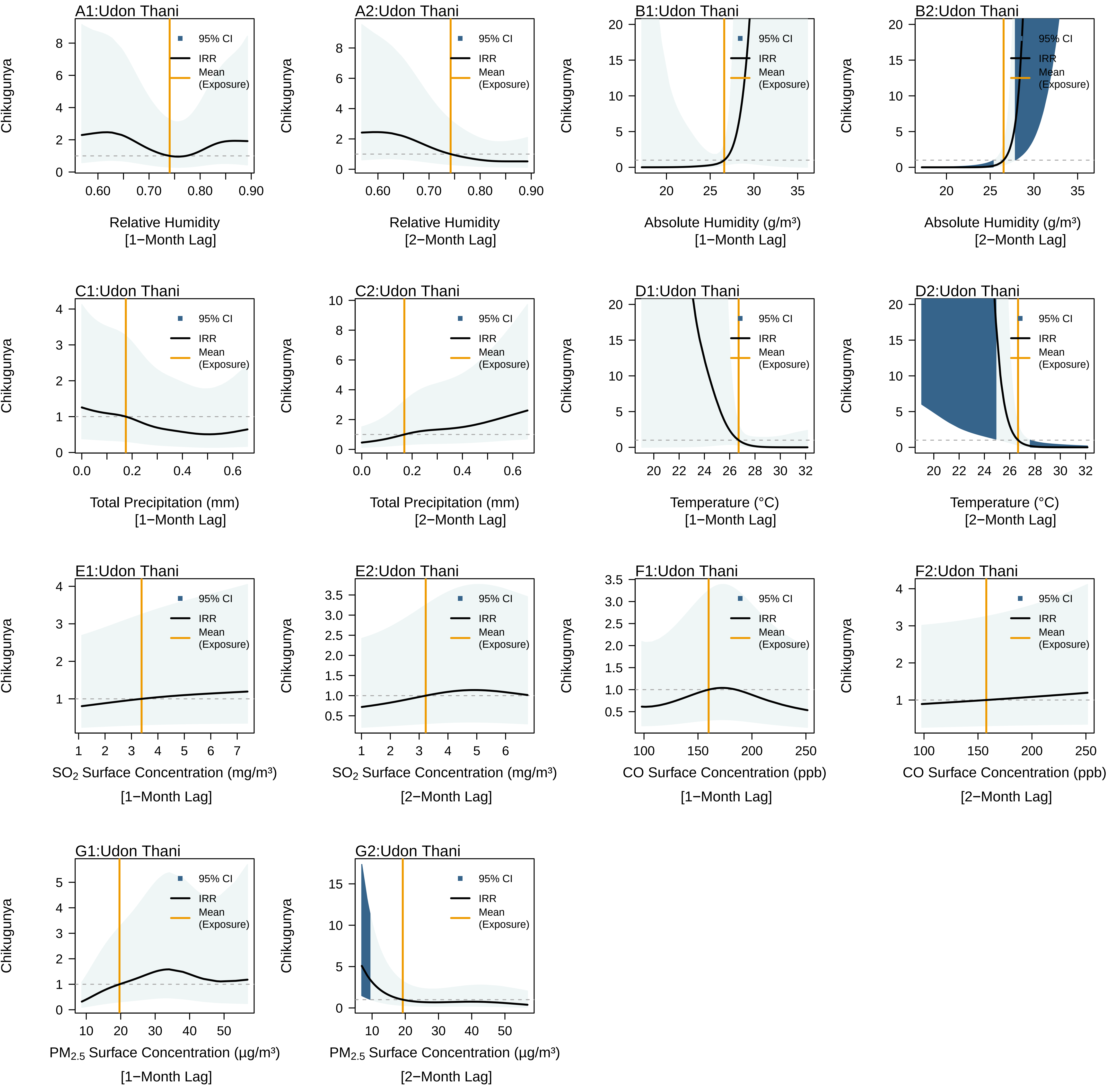
A1:Udon Thani
A2:Udon Thani
B1:Udon Thani
B2:Udon Thani
95% CI
IRR
Mean
(Exposure)
Chikugunya
Relative Humidity
[1−Month Lag]
Relative Humidity
[2−Month Lag]
Absolute Humidity (g/m³)
[1−Month Lag]
Absolute Humidity (g/m³)
[2−Month Lag]
C1:Udon Thani
C2:Udon Thani
D1:Udon Thani
D2:Udon Thani
Total Precipitation (mm)
[1−Month Lag]
Total Precipitation (mm)
[2−Month Lag]
Temperature (°C)
[1−Month Lag]
Temperature (°C)
[2−Month Lag]
E1:Udon Thani
E2:Udon Thani
F1:Udon Thani
F2:Udon Thani
SO_2 Surface Concentration (mg/m³)
[1−Month Lag]
SO_2 Surface Concentration (mg/m³)
[2−Month Lag]
CO Surface Concentration (ppb)
[1−Month Lag]
CO Surface Concentration (ppb)
[2−Month Lag]
G1:Udon Thani
G2:Udon Thani
$PM_{2.5}$ Surface Concentration (µg/m³)
[1−Month Lag]
$PM_{2.5}$ Surface Concentration (µg/m³)
[2−Month Lag]

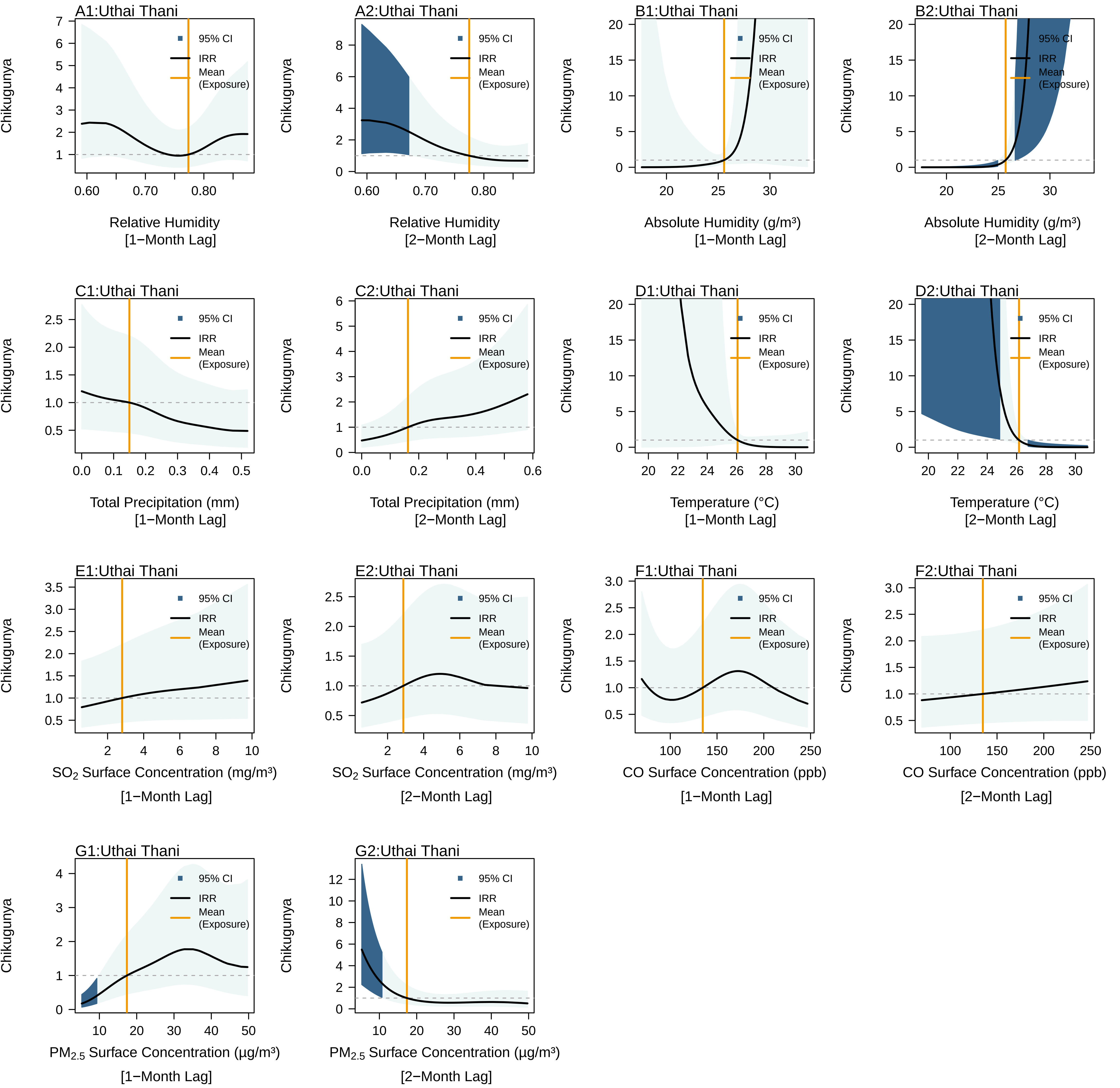

A1:Uthai Thani
Chikugunya
95% CI
IRR
Mean (Exposure)
Relative Humidity [1−Month Lag]
A2:Uthai Thani
Chikugunya
95% CI
IRR
Mean (Exposure)
Relative Humidity [2−Month Lag]
B1:Uthai Thani
Chikugunya
95% CI
IRR
Mean (Exposure)
Absolute Humidity (g/m³) [1−Month Lag]
B2:Uthai Thani
Chikugunya
95% CI
IRR
Mean (Exposure)
Absolute Humidity (g/m³) [2−Month Lag]
C1:Uthai Thani
Chikugunya
95% CI
IRR
Mean (Exposure)
Total Precipitation (mm) [1−Month Lag]
C2:Uthai Thani
Chikugunya
95% CI
IRR
Mean (Exposure)
Total Precipitation (mm) [2−Month Lag]
D1:Uthai Thani
Chikugunya
95% CI
IRR
Mean (Exposure)
Temperature (°C) [1−Month Lag]
D2:Uthai Thani
Chikugunya
95% CI
IRR
Mean (Exposure)
Temperature (°C) [2−Month Lag]
E1:Uthai Thani
Chikugunya
95% CI
IRR
Mean (Exposure)
SO2 Surface Concentration (mg/m³) [1−Month Lag]
E2:Uthai Thani
Chikugunya
95% CI
IRR
Mean (Exposure)
SO2 Surface Concentration (mg/m³) [2−Month Lag]
F1:Uthai Thani
Chikugunya
95% CI
IRR
Mean (Exposure)
CO Surface Concentration (ppb) [1−Month Lag]
F2:Uthai Thani
Chikugunya
95% CI
IRR
Mean (Exposure)
CO Surface Concentration (ppb) [2−Month Lag]
G1:Uthai Thani
Chikugunya
95% CI
IRR
Mean (Exposure)
PM2.5 Surface Concentration (µg/m³) [1−Month Lag]
G2:Uthai Thani
Chikugunya
95% CI
IRR
Mean (Exposure)
PM2.5 Surface Concentration (µg/m³) [2−Month Lag]

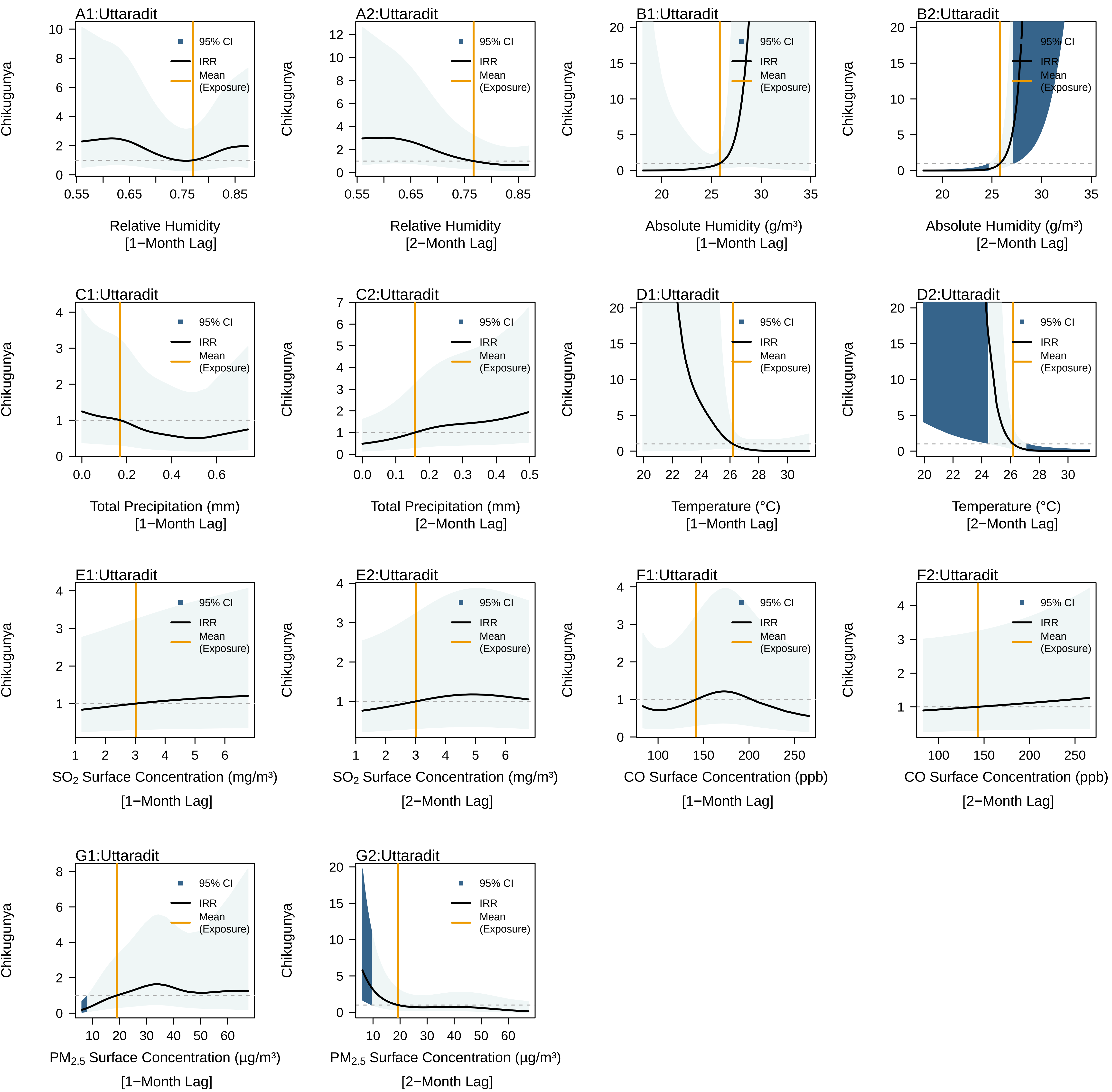
A1:Uttaradit
A2:Uttaradit
B1:Uttaradit
B2:Uttaradit
C1:Uttaradit
C2:Uttaradit
D1:Uttaradit
D2:Uttaradit
E1:Uttaradit
E2:Uttaradit
F1:Uttaradit
F2:Uttaradit
G1:Uttaradit
G2:Uttaradit
Chikugunya
95% CI
IRR
Mean
(Exposure)
Relative Humidity
[1−Month Lag]
Relative Humidity
[2−Month Lag]
Absolute Humidity (g/m³)
[1−Month Lag]
Absolute Humidity (g/m³)
[2−Month Lag]
Total Precipitation (mm)
[1−Month Lag]
Total Precipitation (mm)
[2−Month Lag]
Temperature (°C)
[1−Month Lag]
Temperature (°C)
[2−Month Lag]
SO_2 Surface Concentration (mg/m³)
[1−Month Lag]
SO_2 Surface Concentration (mg/m³)
[2−Month Lag]
CO Surface Concentration (ppb)
[1−Month Lag]
CO Surface Concentration (ppb)
[2−Month Lag]
$PM_{2.5}$ Surface Concentration (µg/m³)
[1−Month Lag]
$PM_{2.5}$ Surface Concentration (µg/m³)
[2−Month Lag]

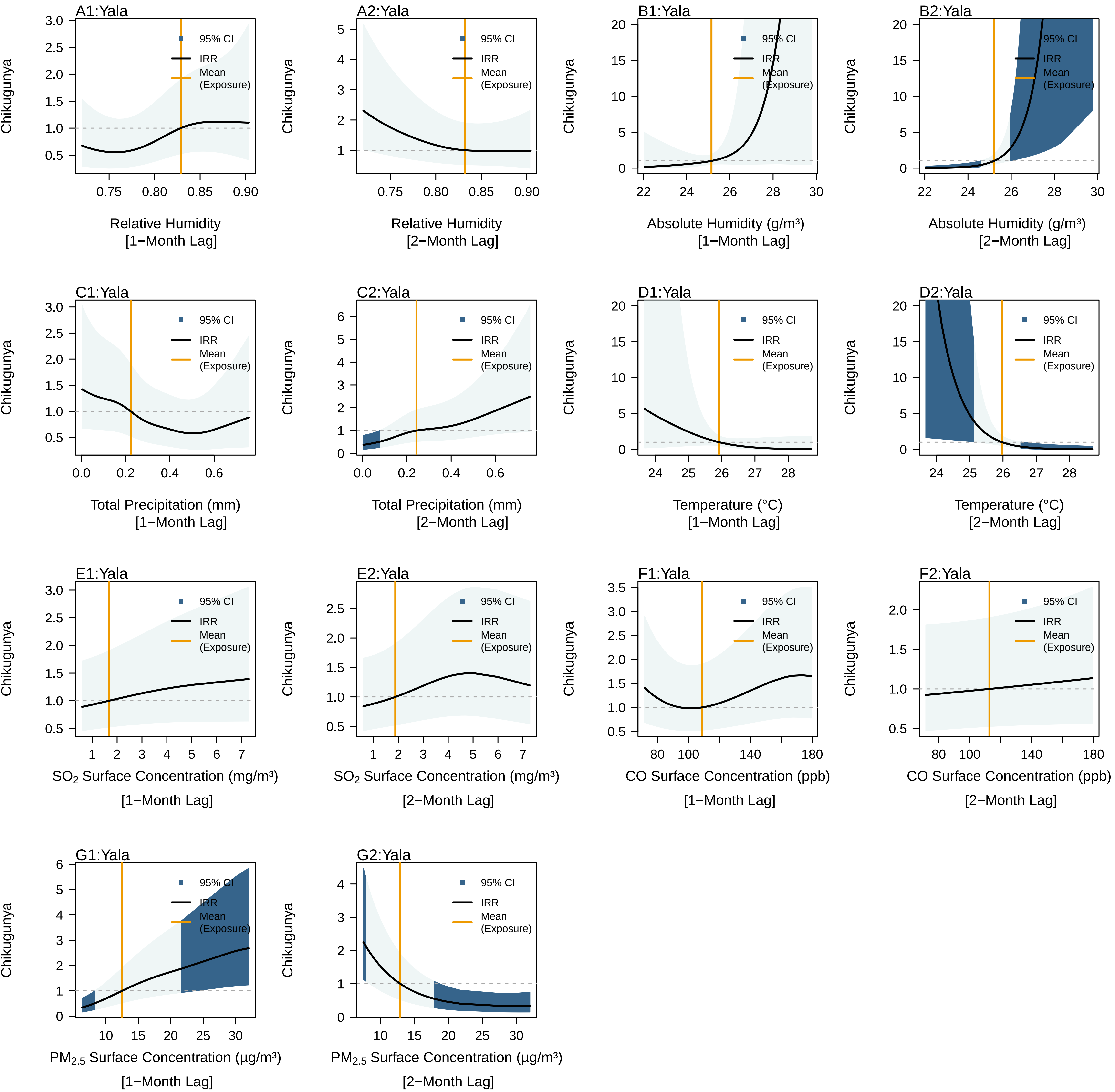
A1:Yala
Chikugunya
95% CI
IRR
Mean
(Exposure)
Relative Humidity
[1−Month Lag]
A2:Yala
Chikugunya
95% CI
IRR
Mean
(Exposure)
Relative Humidity
[2−Month Lag]
B1:Yala
Chikugunya
95% CI
IRR
Mean
(Exposure)
Absolute Humidity (g/m³)
[1−Month Lag]
B2:Yala
Chikugunya
95% CI
IRR
Mean
(Exposure)
Absolute Humidity (g/m³)
[2−Month Lag]
C1:Yala
Chikugunya
95% CI
IRR
Mean
(Exposure)
Total Precipitation (mm)
[1−Month Lag]
C2:Yala
Chikugunya
95% CI
IRR
Mean
(Exposure)
Total Precipitation (mm)
[2−Month Lag]
D1:Yala
Chikugunya
95% CI
IRR
Mean
(Exposure)
Temperature (°C)
[1−Month Lag]
D2:Yala
Chikugunya
95% CI
IRR
Mean
(Exposure)
Temperature (°C)
[2−Month Lag]
E1:Yala
Chikugunya
95% CI
IRR
Mean
(Exposure)
SO2 Surface Concentration (mg/m³)
[1−Month Lag]
E2:Yala
Chikugunya
95% CI
IRR
Mean
(Exposure)
SO2 Surface Concentration (mg/m³)
[2−Month Lag]
F1:Yala
Chikugunya
95% CI
IRR
Mean
(Exposure)
CO Surface Concentration (ppb)
[1−Month Lag]
F2:Yala
Chikugunya
95% CI
IRR
Mean
(Exposure)
CO Surface Concentration (ppb)
[2−Month Lag]
G1:Yala
Chikugunya
95% CI
IRR
Mean
(Exposure)
PM2.5 Surface Concentration (μg/m³)
[1−Month Lag]
G2:Yala
Chikugunya
95% CI
IRR
Mean
(Exposure)
PM2.5 Surface Concentration (μg/m³)
[2−Month Lag]

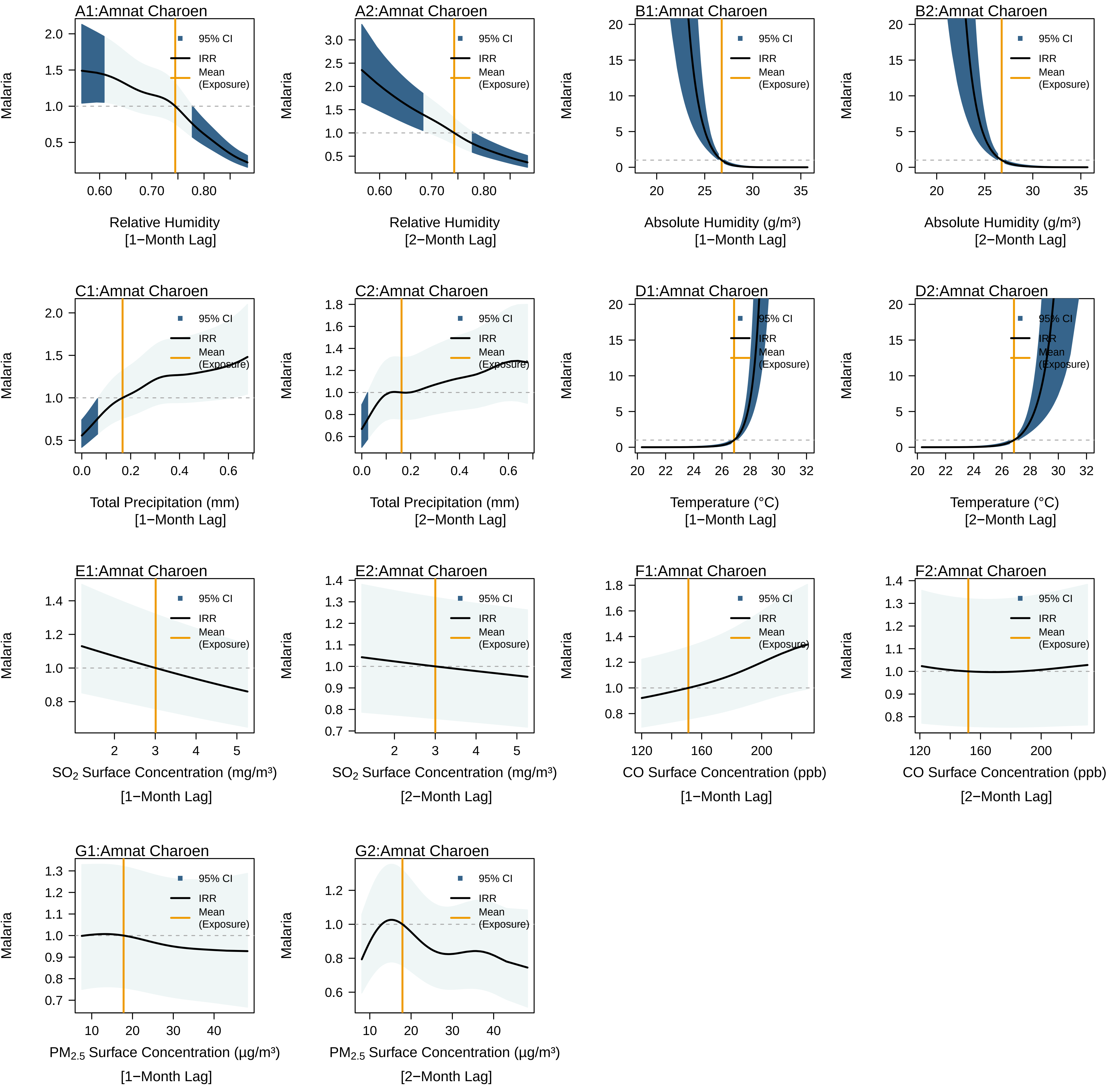
A1:Amnat Charoen
Malaria
95% CI
IRR
Mean (Exposure)
Relative Humidity [1−Month Lag]
A2:Amnat Charoen
Malaria
95% CI
IRR
Mean (Exposure)
Relative Humidity [2−Month Lag]
B1:Amnat Charoen
Malaria
95% CI
IRR
Mean (Exposure)
Absolute Humidity (g/m³) [1−Month Lag]
B2:Amnat Charoen
Malaria
95% CI
IRR
Mean (Exposure)
Absolute Humidity (g/m³) [2−Month Lag]
C1:Amnat Charoen
Malaria
95% CI
IRR
Mean (Exposure)
Total Precipitation (mm) [1−Month Lag]
C2:Amnat Charoen
Malaria
95% CI
IRR
Mean (Exposure)
Total Precipitation (mm) [2−Month Lag]
D1:Amnat Charoen
Malaria
95% CI
IRR
Mean (Exposure)
Temperature (°C) [1−Month Lag]
D2:Amnat Charoen
Malaria
95% CI
IRR
Mean (Exposure)
Temperature (°C) [2−Month Lag]
E1:Amnat Charoen
Malaria
95% CI
IRR
Mean (Exposure)
SO_2 Surface Concentration (mg/m³) [1−Month Lag]
E2:Amnat Charoen
Malaria
95% CI
IRR
Mean (Exposure)
SO_2 Surface Concentration (mg/m³) [2−Month Lag]
F1:Amnat Charoen
Malaria
95% CI
IRR
Mean (Exposure)
CO Surface Concentration (ppb) [1−Month Lag]
F2:Amnat Charoen
Malaria
95% CI
IRR
Mean (Exposure)
CO Surface Concentration (ppb) [2−Month Lag]
G1:Amnat Charoen
Malaria
95% CI
IRR
Mean (Exposure)
$PM_{2.5}$ Surface Concentration (µg/m³) [1−Month Lag]
G2:Amnat Charoen
Malaria
95% CI
IRR
Mean (Exposure)
$PM_{2.5}$ Surface Concentration (µg/m³) [2−Month Lag]

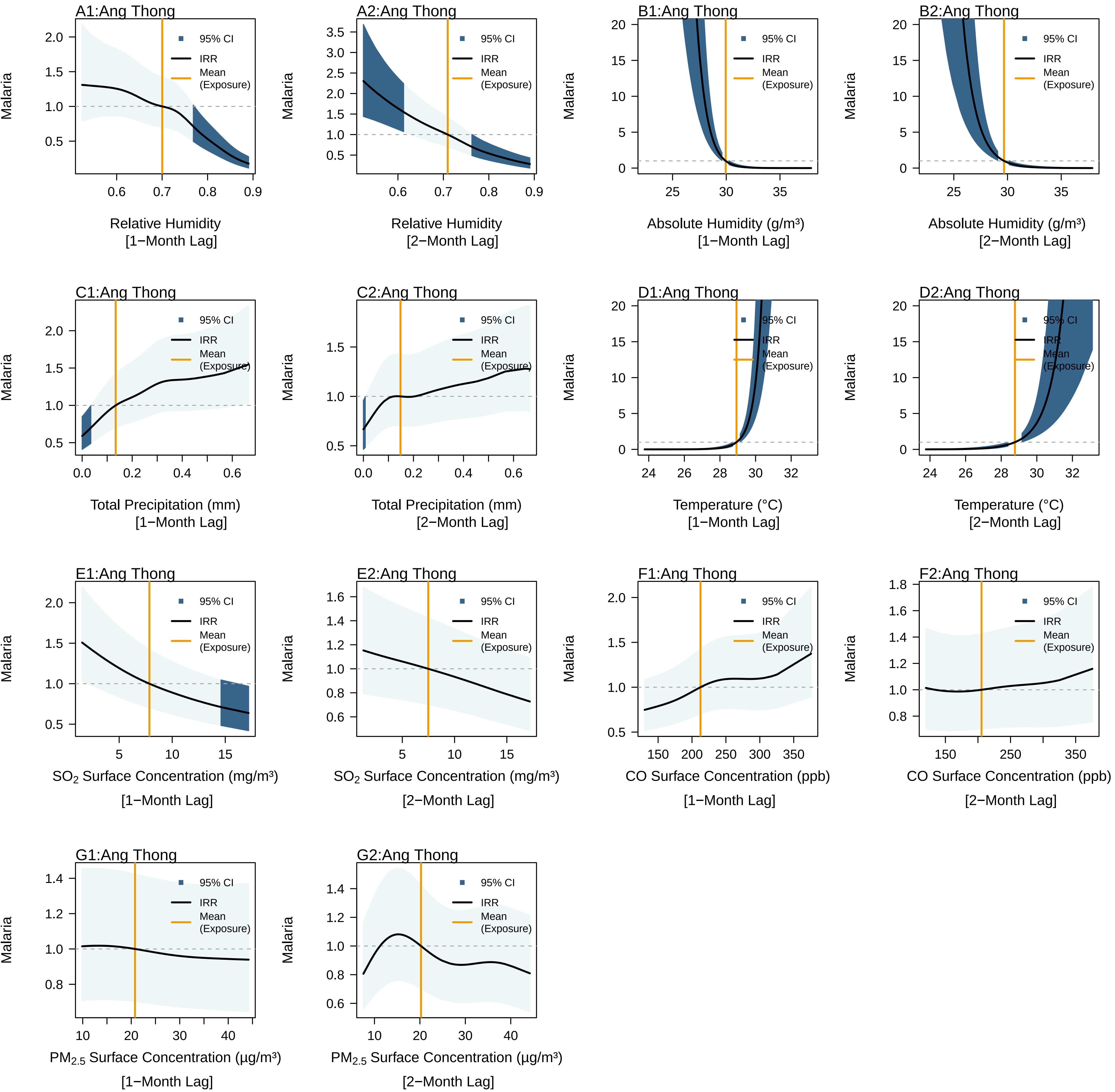

A1:Ang Thong
A2:Ang Thong
B1:Ang Thong
B2:Ang Thong
C1:Ang Thong
C2:Ang Thong
D1:Ang Thong
D2:Ang Thong
E1:Ang Thong
E2:Ang Thong
F1:Ang Thong
F2:Ang Thong
G1:Ang Thong
G2:Ang Thong
Malaria
95% CI
IRR
Mean (Exposure)
Relative Humidity [1−Month Lag]
Relative Humidity [2−Month Lag]
Absolute Humidity (g/m³) [1−Month Lag]
Absolute Humidity (g/m³) [2−Month Lag]
Total Precipitation (mm) [1−Month Lag]
Total Precipitation (mm) [2−Month Lag]
Temperature (°C) [1−Month Lag]
Temperature (°C) [2−Month Lag]
SO_2 Surface Concentration (mg/m³) [1−Month Lag]
SO_2 Surface Concentration (mg/m³) [2−Month Lag]
CO Surface Concentration (ppb) [1−Month Lag]
CO Surface Concentration (ppb) [2−Month Lag]
$PM_{2.5}$ Surface Concentration (µg/m³) [1−Month Lag]
$PM_{2.5}$ Surface Concentration (µg/m³) [2−Month Lag]

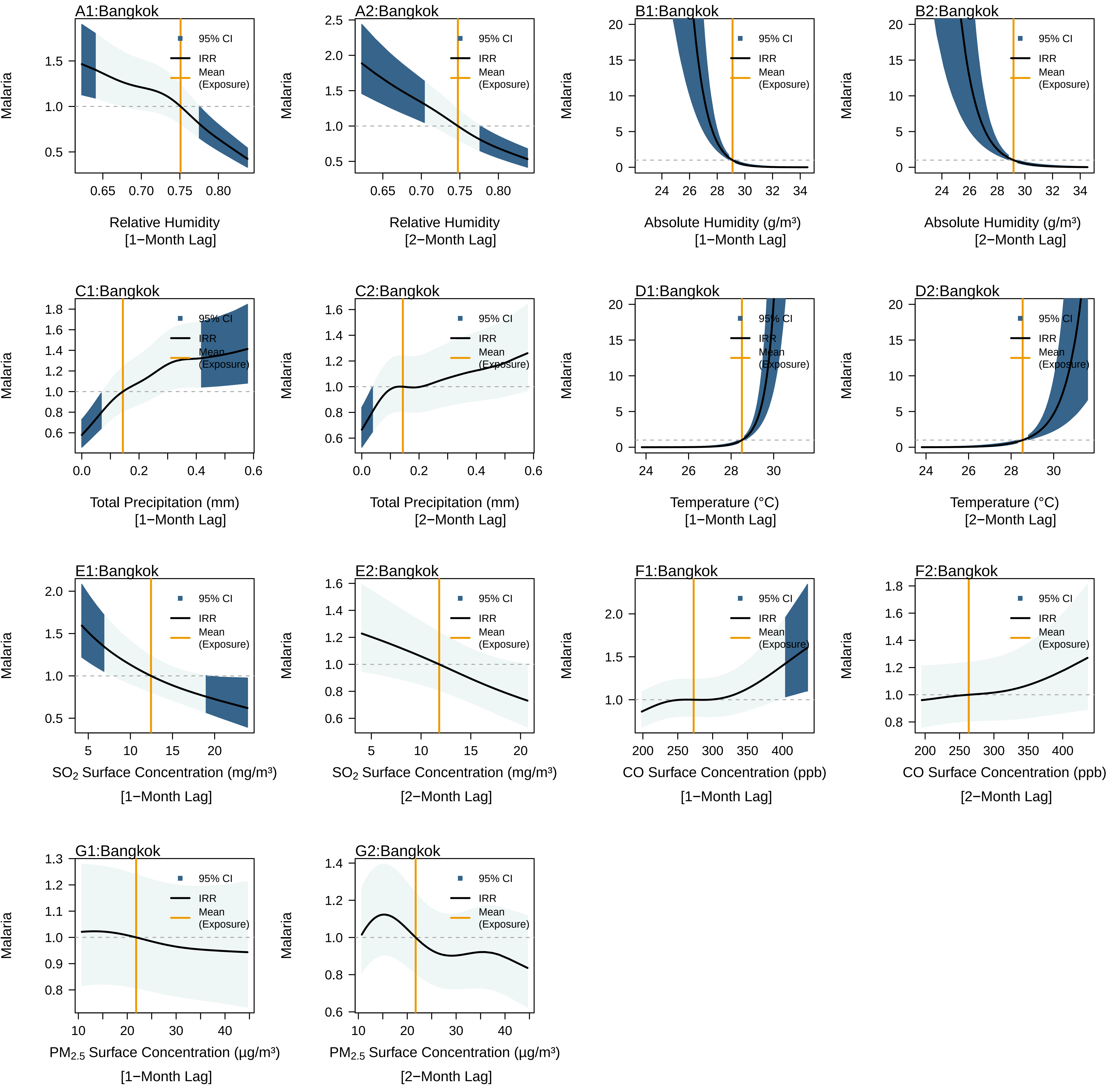
A1:Bangkok
A2:Bangkok
B1:Bangkok
B2:Bangkok
95% CI
IRR
Mean (Exposure)
Malaria
Relative Humidity [1−Month Lag]
Relative Humidity [2−Month Lag]
Absolute Humidity (g/m³) [1−Month Lag]
Absolute Humidity (g/m³) [2−Month Lag]
C1:Bangkok
C2:Bangkok
D1:Bangkok
D2:Bangkok
Total Precipitation (mm) [1−Month Lag]
Total Precipitation (mm) [2−Month Lag]
Temperature (°C) [1−Month Lag]
Temperature (°C) [2−Month Lag]
E1:Bangkok
E2:Bangkok
F1:Bangkok
F2:Bangkok
SO2 Surface Concentration (mg/m³) [1−Month Lag]
SO2 Surface Concentration (mg/m³) [2−Month Lag]
CO Surface Concentration (ppb) [1−Month Lag]
CO Surface Concentration (ppb) [2−Month Lag]
G1:Bangkok
G2:Bangkok
PM2.5 Surface Concentration (µg/m³) [1−Month Lag]
PM2.5 Surface Concentration (µg/m³) [2−Month Lag]

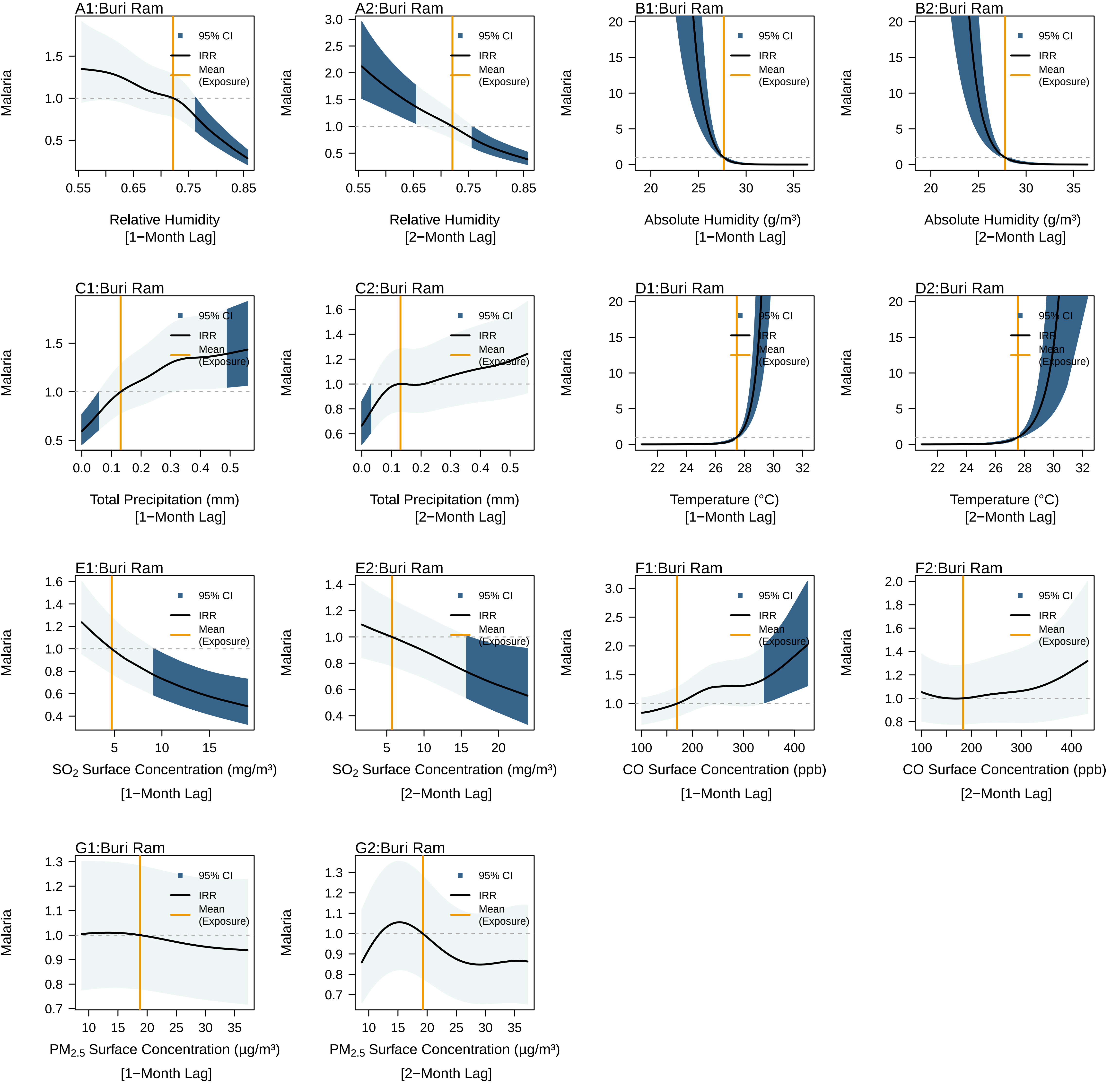
A1:Buri Ram
Malaria
95% CI
IRR
Mean (Exposure)
Relative Humidity [1−Month Lag]
A2:Buri Ram
Relative Humidity [2−Month Lag]
B1:Buri Ram
Absolute Humidity (g/m³) [1−Month Lag]
B2:Buri Ram
Absolute Humidity (g/m³) [2−Month Lag]
C1:Buri Ram
Total Precipitation (mm) [1−Month Lag]
C2:Buri Ram
Total Precipitation (mm) [2−Month Lag]
D1:Buri Ram
Temperature (°C) [1−Month Lag]
D2:Buri Ram
Temperature (°C) [2−Month Lag]
E1:Buri Ram
SO2 Surface Concentration (mg/m³) [1−Month Lag]
E2:Buri Ram
SO2 Surface Concentration (mg/m³) [2−Month Lag]
F1:Buri Ram
CO Surface Concentration (ppb) [1−Month Lag]
F2:Buri Ram
CO Surface Concentration (ppb) [2−Month Lag]
G1:Buri Ram
PM2.5 Surface Concentration (µg/m³) [1−Month Lag]
G2:Buri Ram
PM2.5 Surface Concentration (µg/m³) [2−Month Lag]

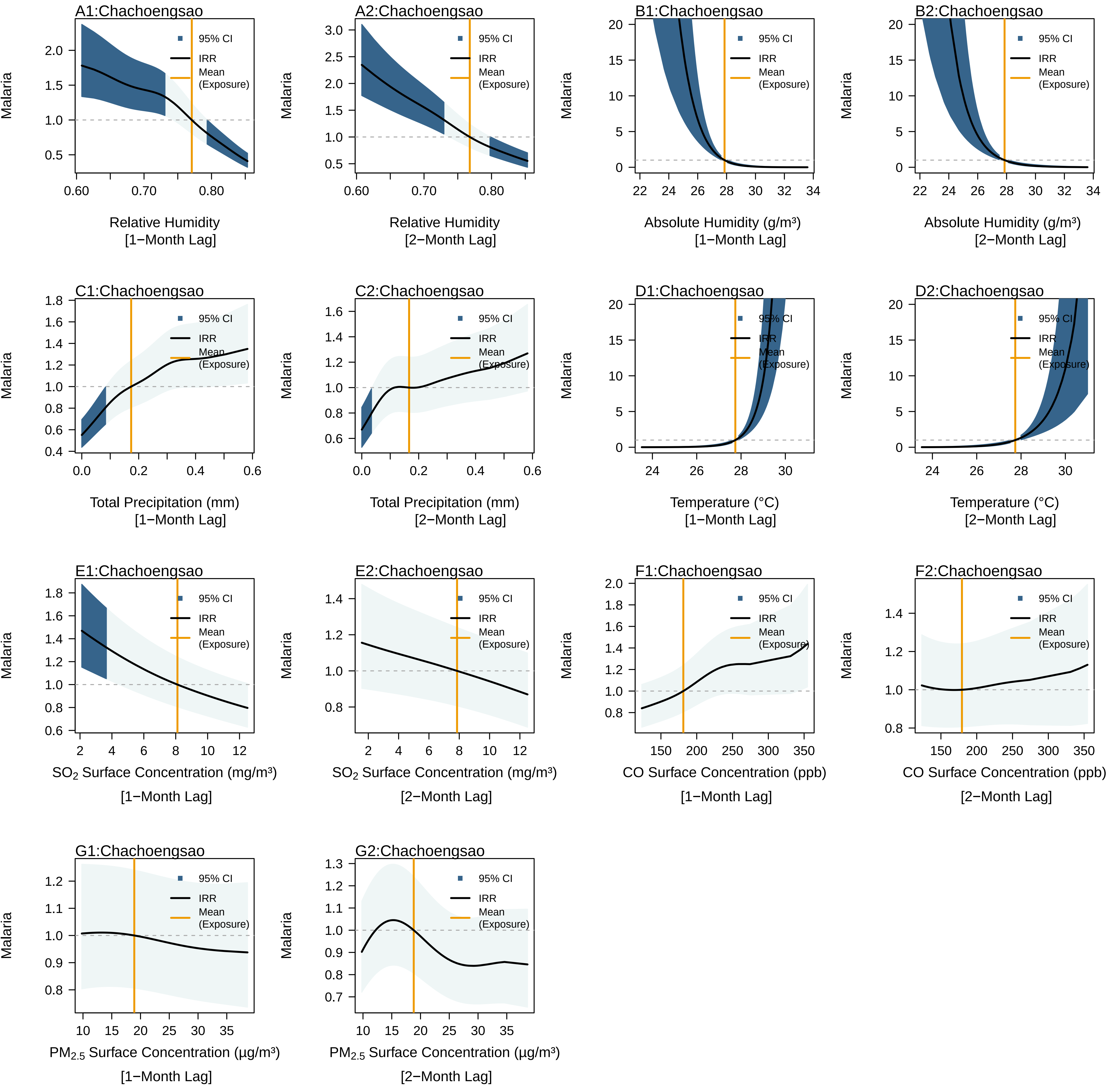
A1:Chachoengsao
A2:Chachoengsao
B1:Chachoengsao
B2:Chachoengsao
95% CI
IRR
Mean
(Exposure)
Malaria
Relative Humidity
[1−Month Lag]
Relative Humidity
[2−Month Lag]
Absolute Humidity (g/m³)
[1−Month Lag]
Absolute Humidity (g/m³)
[2−Month Lag]
C1:Chachoengsao
C2:Chachoengsao
D1:Chachoengsao
D2:Chachoengsao
Total Precipitation (mm)
[1−Month Lag]
Total Precipitation (mm)
[2−Month Lag]
Temperature (°C)
[1−Month Lag]
Temperature (°C)
[2−Month Lag]
E1:Chachoengsao
E2:Chachoengsao
F1:Chachoengsao
F2:Chachoengsao
SO_2 Surface Concentration (mg/m³)
[1−Month Lag]
SO_2 Surface Concentration (mg/m³)
[2−Month Lag]
CO Surface Concentration (ppb)
[1−Month Lag]
CO Surface Concentration (ppb)
[2−Month Lag]
G1:Chachoengsao
G2:Chachoengsao
$PM_{2.5}$ Surface Concentration (µg/m³)
[1−Month Lag]
$PM_{2.5}$ Surface Concentration (µg/m³)
[2−Month Lag]

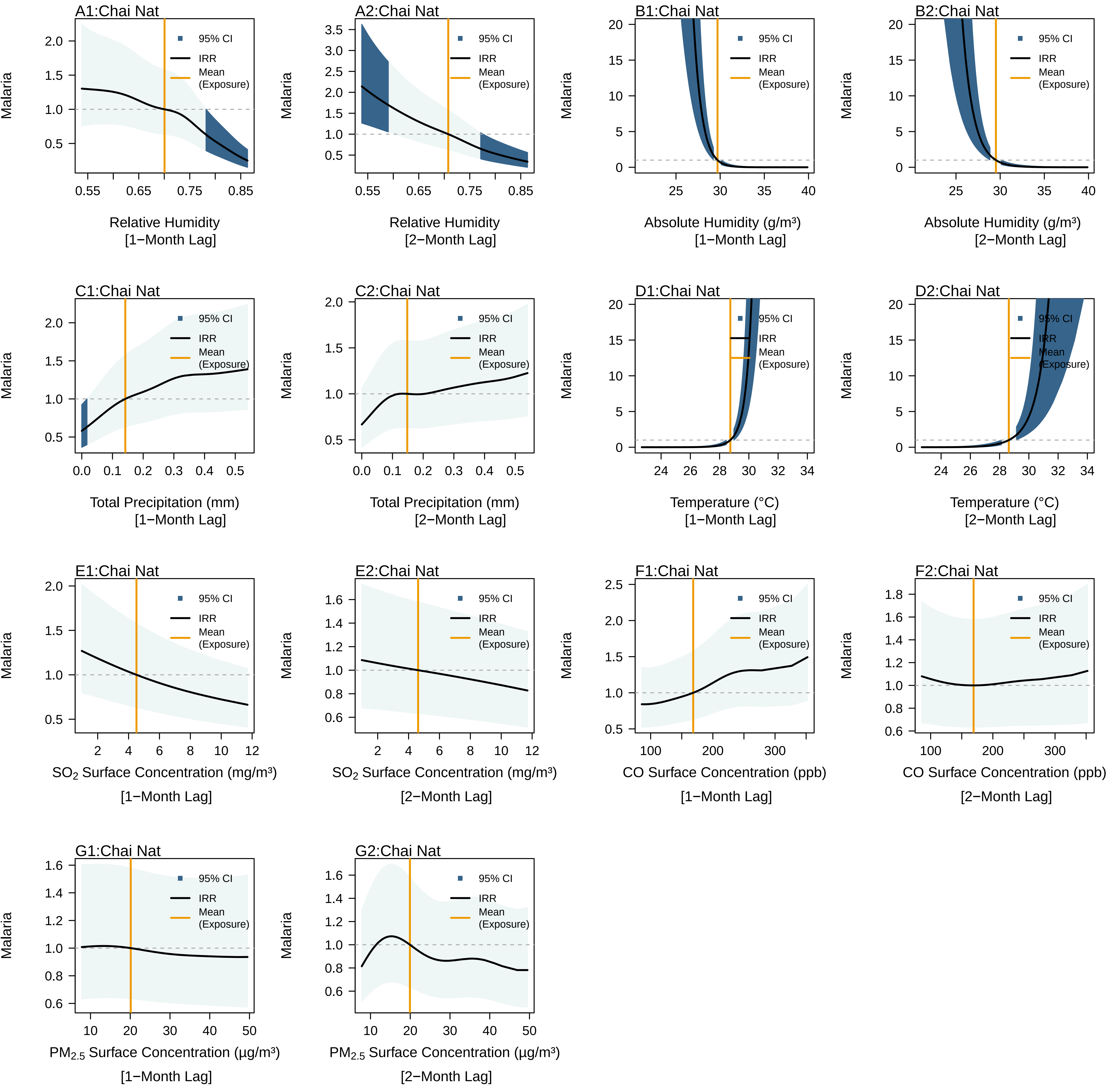
A1:Chai Nat
Malaria
Relative Humidity
[1−Month Lag]
A2:Chai Nat
Malaria
Relative Humidity
[2−Month Lag]
B1:Chai Nat
Malaria
Absolute Humidity (g/m³)
[1−Month Lag]
B2:Chai Nat
Malaria
Absolute Humidity (g/m³)
[2−Month Lag]
C1:Chai Nat
Malaria
Total Precipitation (mm)
[1−Month Lag]
C2:Chai Nat
Malaria
Total Precipitation (mm)
[2−Month Lag]
D1:Chai Nat
Malaria
Temperature (°C)
[1−Month Lag]
D2:Chai Nat
Malaria
Temperature (°C)
[2−Month Lag]
E1:Chai Nat
Malaria
SO2 Surface Concentration (mg/m³)
[1−Month Lag]
E2:Chai Nat
Malaria
SO2 Surface Concentration (mg/m³)
[2−Month Lag]
F1:Chai Nat
Malaria
CO Surface Concentration (ppb)
[1−Month Lag]
F2:Chai Nat
Malaria
CO Surface Concentration (ppb)
[2−Month Lag]
G1:Chai Nat
Malaria
PM2.5 Surface Concentration (µg/m³)
[1−Month Lag]
G2:Chai Nat
Malaria
PM2.5 Surface Concentration (µg/m³)
[2−Month Lag]
95% CI
IRR
Mean
(Exposure)

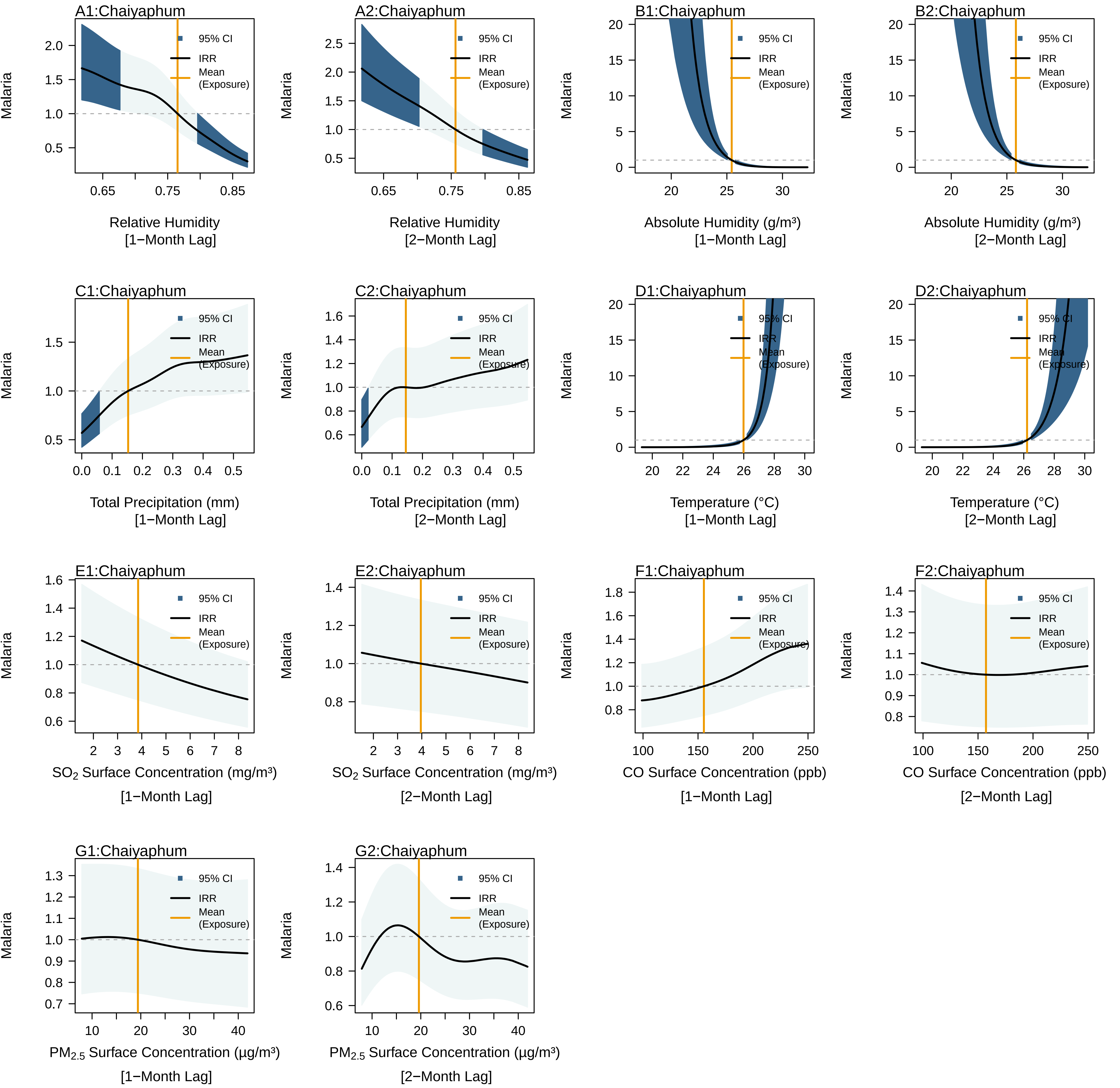
A1:Chaiyaphum
Malaria
95% CI
IRR
Mean (Exposure)
Relative Humidity [1−Month Lag]
A2:Chaiyaphum
Relative Humidity [2−Month Lag]
B1:Chaiyaphum
Absolute Humidity (g/m³) [1−Month Lag]
B2:Chaiyaphum
Absolute Humidity (g/m³) [2−Month Lag]
C1:Chaiyaphum
Total Precipitation (mm) [1−Month Lag]
C2:Chaiyaphum
Total Precipitation (mm) [2−Month Lag]
D1:Chaiyaphum
Temperature (°C) [1−Month Lag]
D2:Chaiyaphum
Temperature (°C) [2−Month Lag]
E1:Chaiyaphum
SO_2 Surface Concentration (mg/m³) [1−Month Lag]
E2:Chaiyaphum
SO_2 Surface Concentration (mg/m³) [2−Month Lag]
F1:Chaiyaphum
CO Surface Concentration (ppb) [1−Month Lag]
F2:Chaiyaphum
CO Surface Concentration (ppb) [2−Month Lag]
G1:Chaiyaphum
$PM_{2.5}$ Surface Concentration (µg/m³) [1−Month Lag]
G2:Chaiyaphum
$PM_{2.5}$ Surface Concentration (µg/m³) [2−Month Lag]

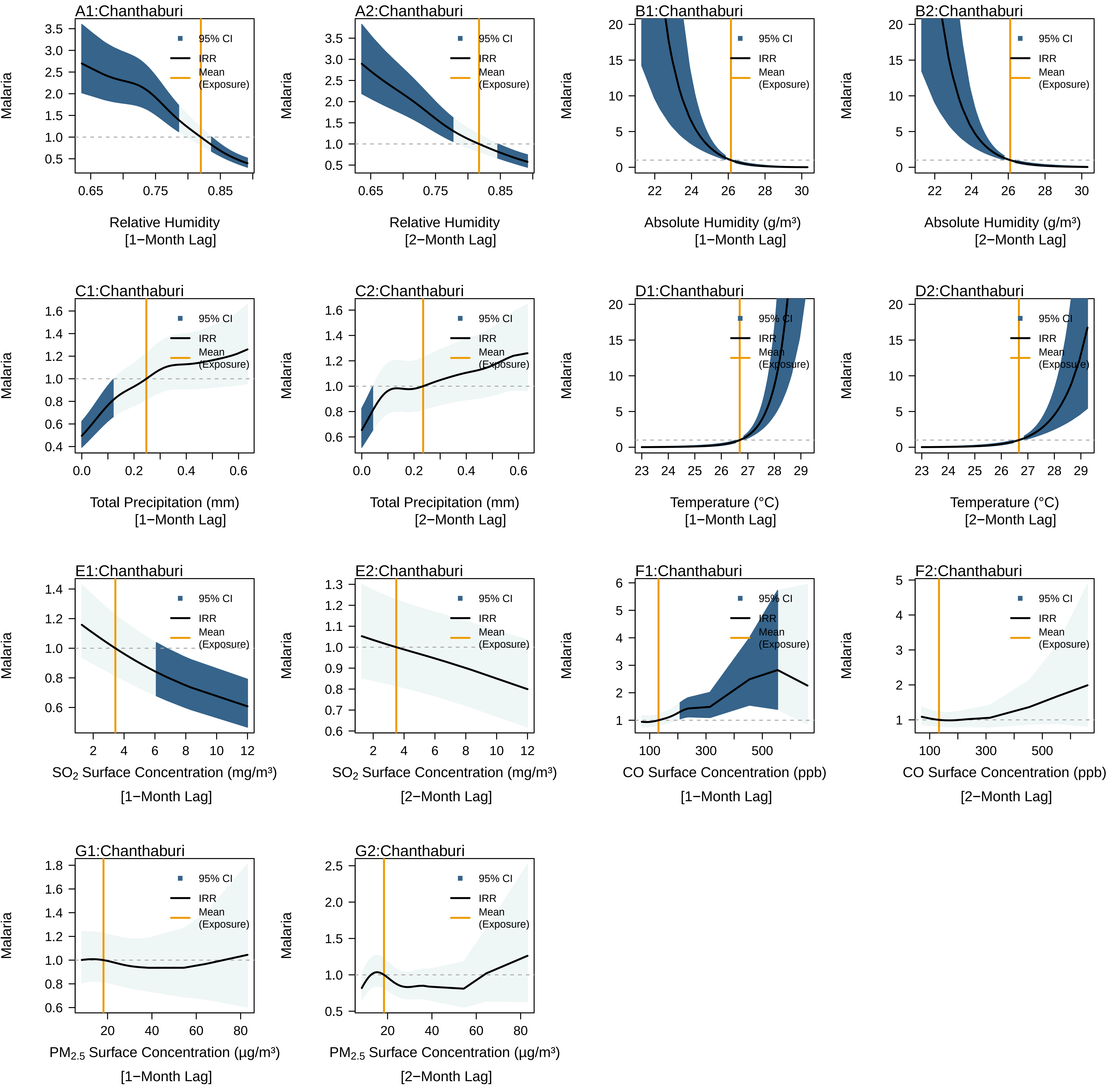
A1:Chanthaburi
A2:Chanthaburi
B1:Chanthaburi
B2:Chanthaburi
C1:Chanthaburi
C2:Chanthaburi
D1:Chanthaburi
D2:Chanthaburi
E1:Chanthaburi
E2:Chanthaburi
F1:Chanthaburi
F2:Chanthaburi
G1:Chanthaburi
G2:Chanthaburi
Malaria
95% CI
IRR
Mean (Exposure)
Relative Humidity [1−Month Lag]
Relative Humidity [2−Month Lag]
Absolute Humidity (g/m³) [1−Month Lag]
Absolute Humidity (g/m³) [2−Month Lag]
Total Precipitation (mm) [1−Month Lag]
Total Precipitation (mm) [2−Month Lag]
Temperature (°C) [1−Month Lag]
Temperature (°C) [2−Month Lag]
SO_2 Surface Concentration (mg/m³) [1−Month Lag]
SO_2 Surface Concentration (mg/m³) [2−Month Lag]
CO Surface Concentration (ppb) [1−Month Lag]
CO Surface Concentration (ppb) [2−Month Lag]
$PM_{2.5}$ Surface Concentration (µg/m³) [1−Month Lag]
$PM_{2.5}$ Surface Concentration (µg/m³) [2−Month Lag]

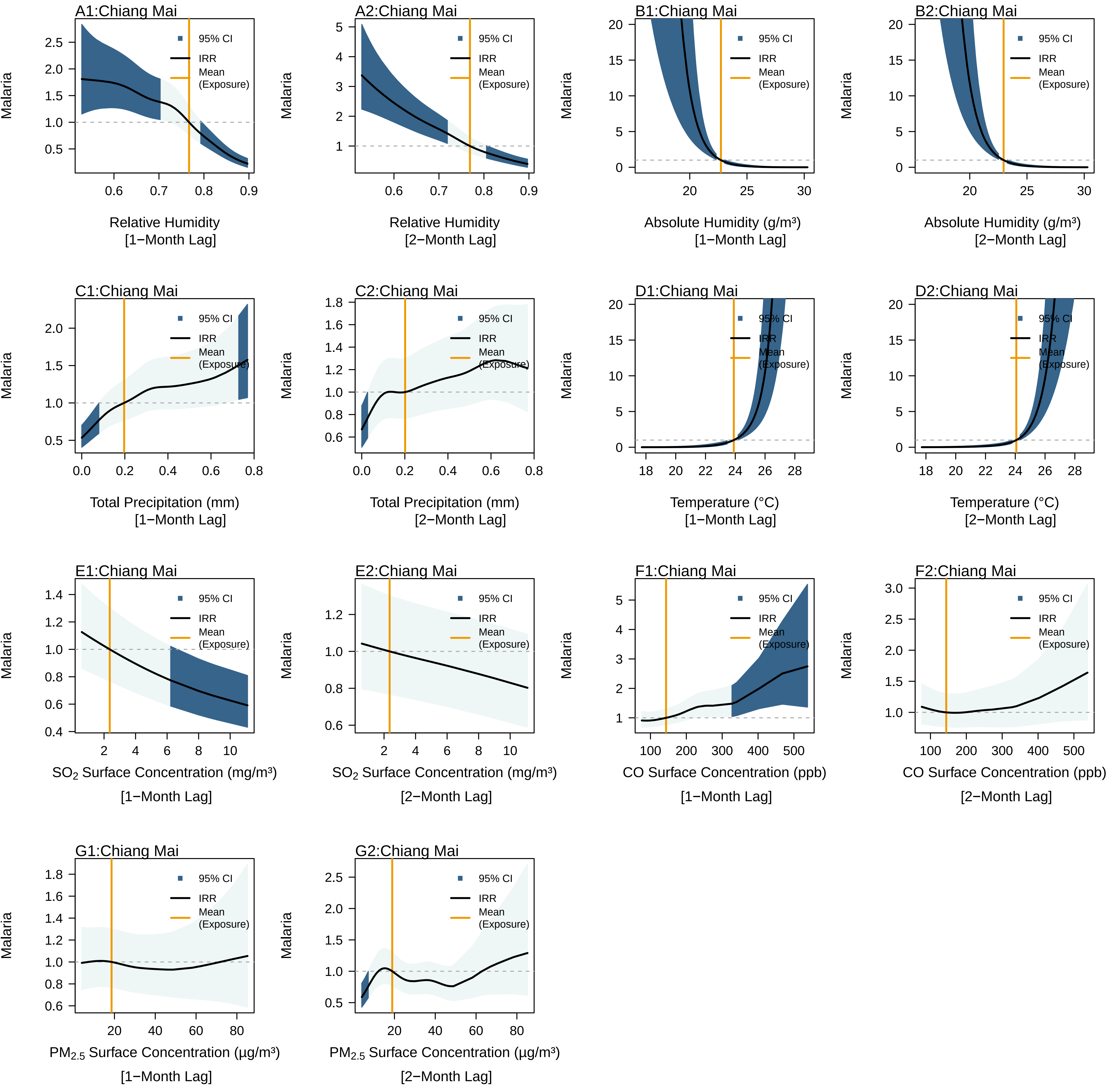

A1:Chiang Mai
A2:Chiang Mai
B1:Chiang Mai
B2:Chiang Mai
C1:Chiang Mai
C2:Chiang Mai
D1:Chiang Mai
D2:Chiang Mai
E1:Chiang Mai
E2:Chiang Mai
F1:Chiang Mai
F2:Chiang Mai
G1:Chiang Mai
G2:Chiang Mai
Malaria
95% CI
IRR
Mean (Exposure)
Relative Humidity [1−Month Lag]
Relative Humidity [2−Month Lag]
Absolute Humidity (g/m³) [1−Month Lag]
Absolute Humidity (g/m³) [2−Month Lag]
Total Precipitation (mm) [1−Month Lag]
Total Precipitation (mm) [2−Month Lag]
Temperature (°C) [1−Month Lag]
Temperature (°C) [2−Month Lag]
SO_2 Surface Concentration (mg/m³) [1−Month Lag]
SO_2 Surface Concentration (mg/m³) [2−Month Lag]
CO Surface Concentration (ppb) [1−Month Lag]
CO Surface Concentration (ppb) [2−Month Lag]
$PM_{2.5}$ Surface Concentration (µg/m³) [1−Month Lag]
$PM_{2.5}$ Surface Concentration (µg/m³) [2−Month Lag]

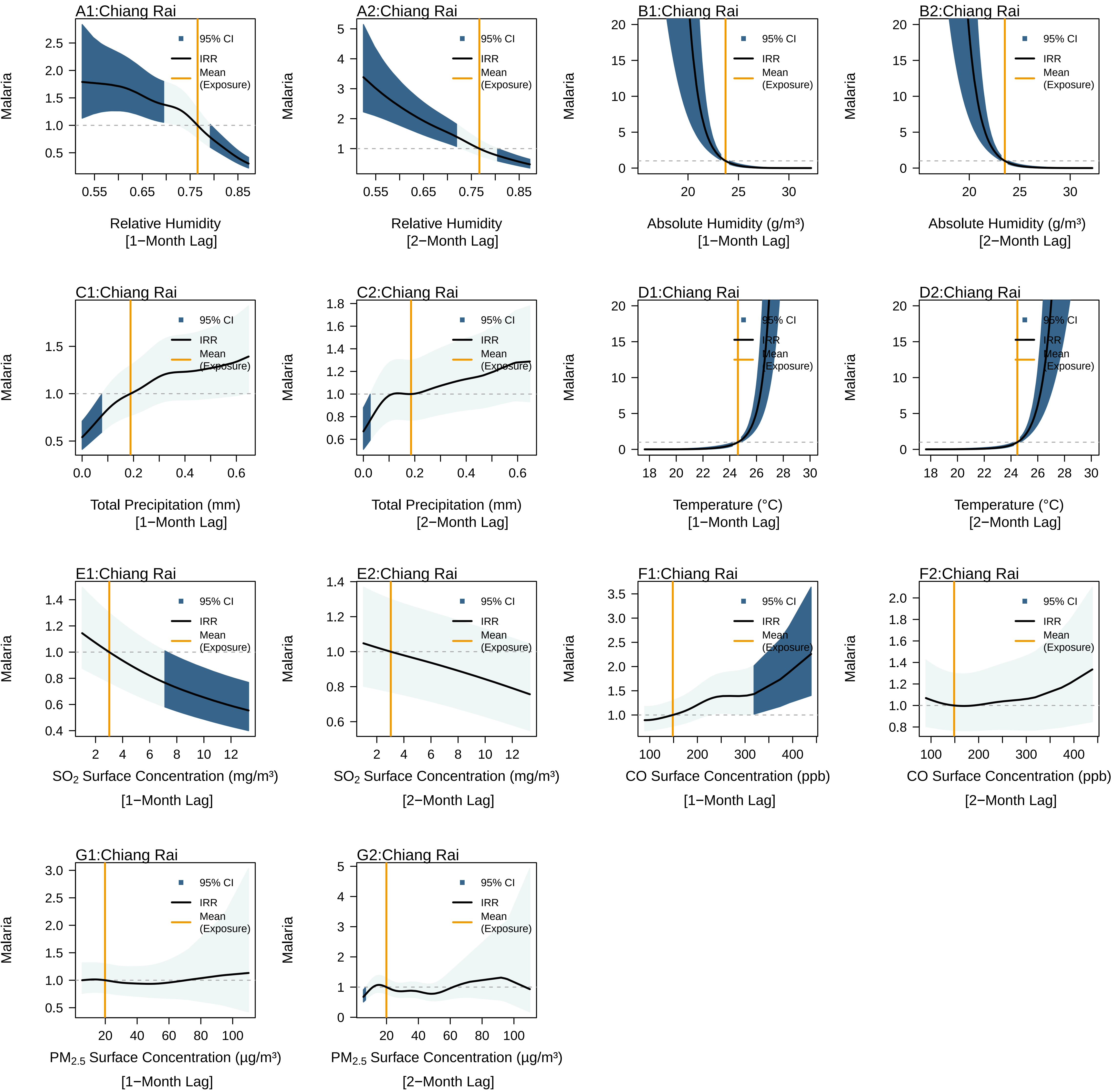
A1:Chiang Rai
A2:Chiang Rai
B1:Chiang Rai
B2:Chiang Rai
C1:Chiang Rai
C2:Chiang Rai
D1:Chiang Rai
D2:Chiang Rai
E1:Chiang Rai
E2:Chiang Rai
F1:Chiang Rai
F2:Chiang Rai
G1:Chiang Rai
G2:Chiang Rai
95% CI
IRR
Mean (Exposure)
Malaria
Relative Humidity [1−Month Lag]
Relative Humidity [2−Month Lag]
Absolute Humidity (g/m³) [1−Month Lag]
Absolute Humidity (g/m³) [2−Month Lag]
Total Precipitation (mm) [1−Month Lag]
Total Precipitation (mm) [2−Month Lag]
Temperature (°C) [1−Month Lag]
Temperature (°C) [2−Month Lag]
SO2 Surface Concentration (mg/m³) [1−Month Lag]
SO2 Surface Concentration (mg/m³) [2−Month Lag]
CO Surface Concentration (ppb) [1−Month Lag]
CO Surface Concentration (ppb) [2−Month Lag]
PM2.5 Surface Concentration (µg/m³) [1−Month Lag]
PM2.5 Surface Concentration (µg/m³) [2−Month Lag]

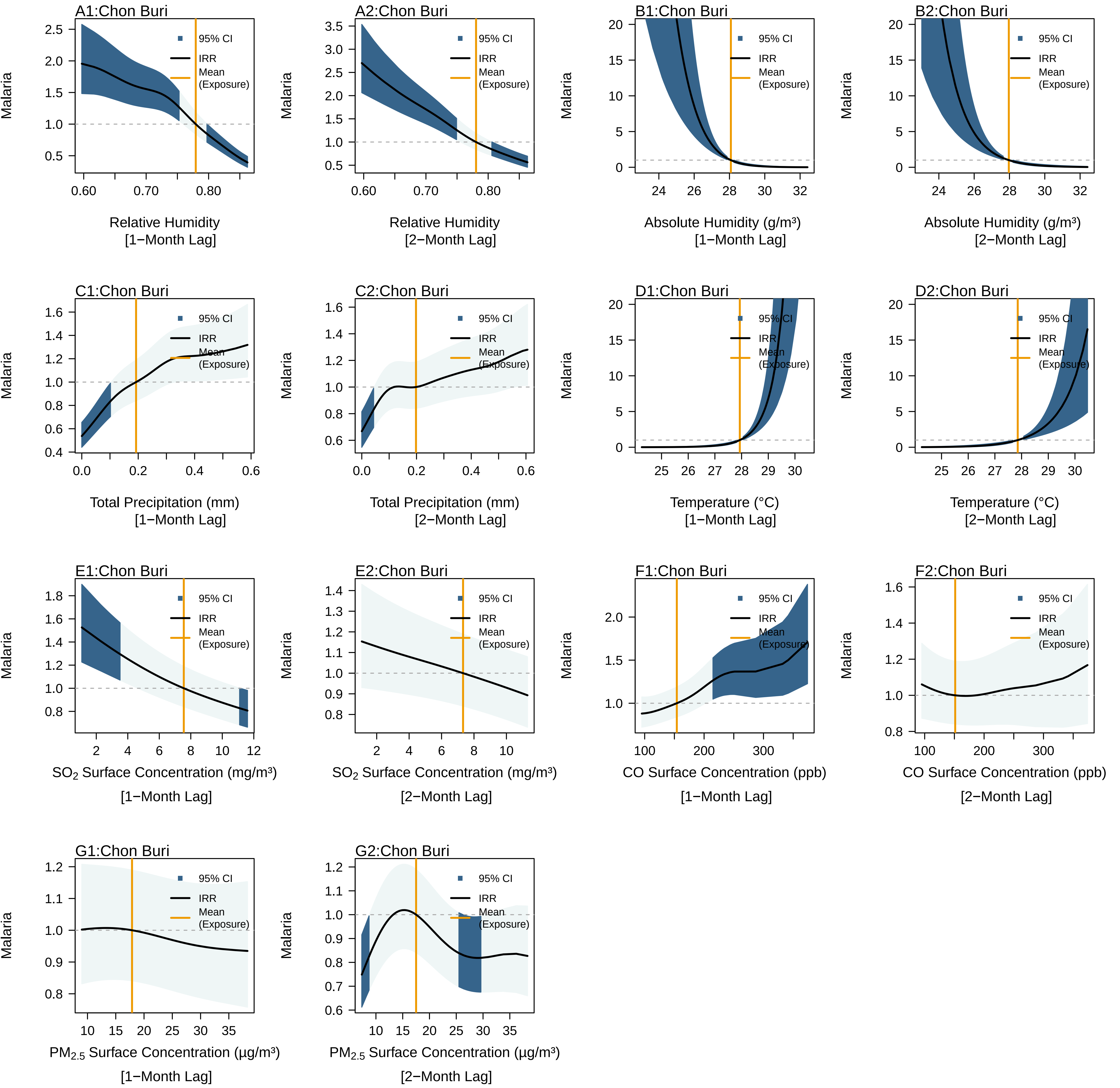
A1:Chon Buri
Malaria
95% CI
IRR
Mean
(Exposure)
Relative Humidity
[1−Month Lag]
A2:Chon Buri
Relative Humidity
[2−Month Lag]
B1:Chon Buri
Absolute Humidity (g/m³)
[1−Month Lag]
B2:Chon Buri
Absolute Humidity (g/m³)
[2−Month Lag]
C1:Chon Buri
Total Precipitation (mm)
[1−Month Lag]
C2:Chon Buri
Total Precipitation (mm)
[2−Month Lag]
D1:Chon Buri
Temperature (°C)
[1−Month Lag]
D2:Chon Buri
Temperature (°C)
[2−Month Lag]
E1:Chon Buri
SO_2 Surface Concentration (mg/m³)
[1−Month Lag]
E2:Chon Buri
SO_2 Surface Concentration (mg/m³)
[2−Month Lag]
F1:Chon Buri
CO Surface Concentration (ppb)
[1−Month Lag]
F2:Chon Buri
CO Surface Concentration (ppb)
[2−Month Lag]
G1:Chon Buri
$PM_{2.5}$ Surface Concentration (µg/m³)
[1−Month Lag]
G2:Chon Buri
$PM_{2.5}$ Surface Concentration (µg/m³)
[2−Month Lag]

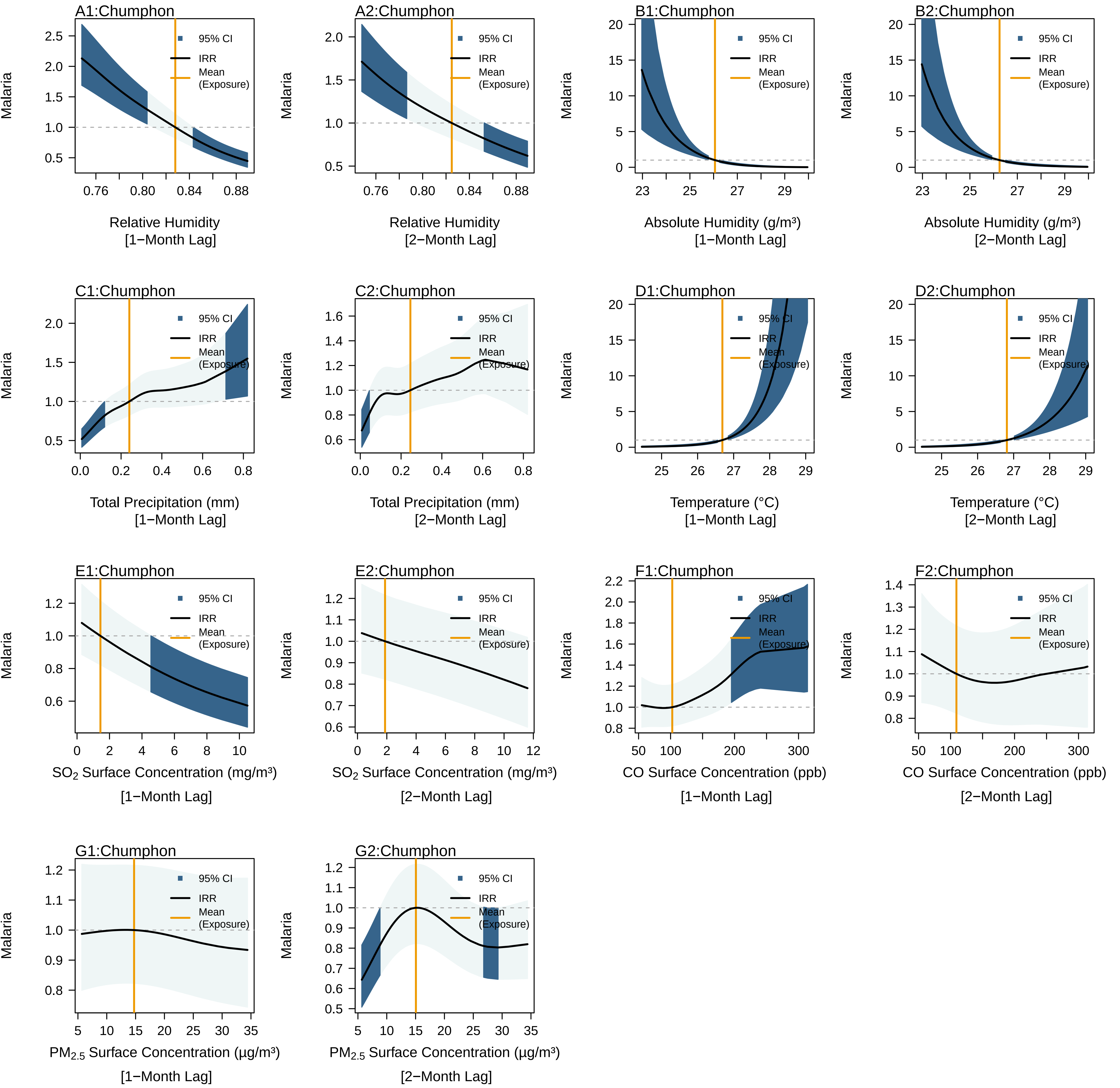
A1:Chumphon
Malaria
95% CI
IRR
Mean (Exposure)
2.5
2.0
1.5
1.0
0.5
0.76
0.80
0.84
0.88
Relative Humidity [1−Month Lag]
A2:Chumphon
Malaria
95% CI
IRR
Mean (Exposure)
2.0
1.5
1.0
0.5
0.76
0.80
0.84
0.88
Relative Humidity [2−Month Lag]
B1:Chumphon
Malaria
95% CI
IRR
Mean (Exposure)
20
15
10
5
0
23
25
27
29
Absolute Humidity (g/m³) [1−Month Lag]
B2:Chumphon
Malaria
95% CI
IRR
Mean (Exposure)
20
15
10
5
0
23
25
27
29
Absolute Humidity (g/m³) [2−Month Lag]
C1:Chumphon
Malaria
95% CI
IRR
Mean (Exposure)
2.0
1.5
1.0
0.5
0.0
0.2
0.4
0.6
0.8
Total Precipitation (mm) [1−Month Lag]
C2:Chumphon
Malaria
95% CI
IRR
Mean (Exposure)
1.6
1.4
1.2
1.0
0.8
0.6
0.0
0.2
0.4
0.6
0.8
Total Precipitation (mm) [2−Month Lag]
D1:Chumphon
Malaria
95% CI
IRR
Mean (Exposure)
20
15
10
5
0
25
26
27
28
29
Temperature (°C) [1−Month Lag]
D2:Chumphon
Malaria
95% CI
IRR
Mean (Exposure)
20
15
10
5
0
25
26
27
28
29
Temperature (°C) [2−Month Lag]
E1:Chumphon
Malaria
95% CI
IRR
Mean (Exposure)
1.2
1.0
0.8
0.6
0
2
4
6
8
10
SO2 Surface Concentration (mg/m³) [1−Month Lag]
E2:Chumphon
Malaria
95% CI
IRR
Mean (Exposure)
1.2
1.1
1.0
0.9
0.8
0.7
0.6
0
2
4
6
8
10
12
SO2 Surface Concentration (mg/m³) [2−Month Lag]
F1:Chumphon
Malaria
95% CI
IRR
Mean (Exposure)
2.2
2.0
1.8
1.6
1.4
1.2
1.0
0.8
50
100
200
300
CO Surface Concentration (ppb) [1−Month Lag]
F2:Chumphon
Malaria
95% CI
IRR
Mean (Exposure)
1.4
1.3
1.2
1.1
1.0
0.9
0.8
50
100
200
300
CO Surface Concentration (ppb) [2−Month Lag]
G1:Chumphon
Malaria
95% CI
IRR
Mean (Exposure)
1.2
1.1
1.0
0.9
0.8
5
10
15
20
25
30
35
PM2.5 Surface Concentration (μg/m³) [1−Month Lag]
G2:Chumphon
Malaria
95% CI
IRR
Mean (Exposure)
1.2
1.1
1.0
0.9
0.8
0.7
0.6
0.5
5
10
15
20
25
30
35
PM2.5 Surface Concentration (μg/m³) [2−Month Lag]

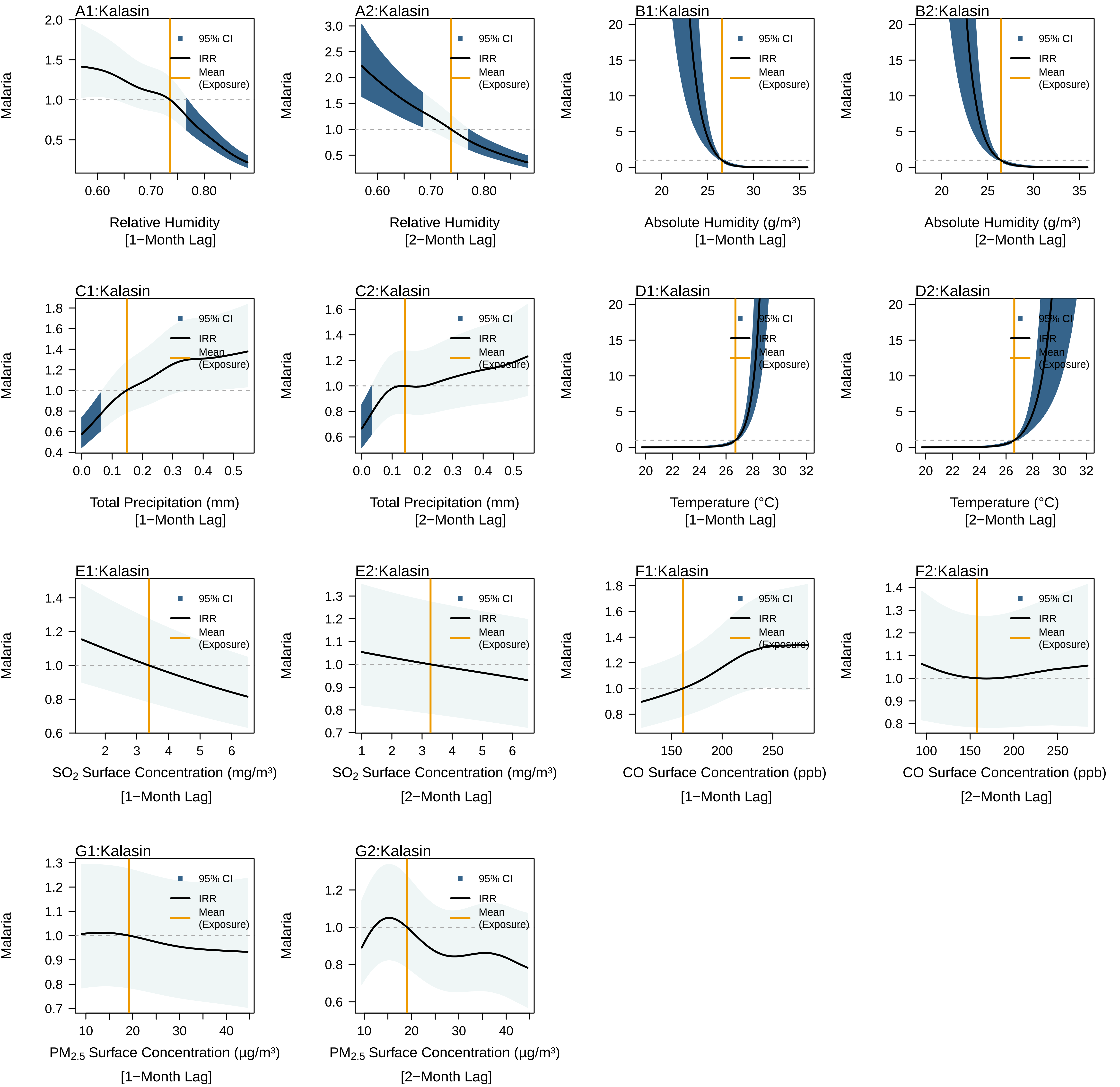
A1:Kalasin
Malaria
95% CI
IRR
Mean (Exposure)
Relative Humidity [1−Month Lag]
A2:Kalasin
Malaria
95% CI
IRR
Mean (Exposure)
Relative Humidity [2−Month Lag]
B1:Kalasin
Malaria
95% CI
IRR
Mean (Exposure)
Absolute Humidity (g/m³) [1−Month Lag]
B2:Kalasin
Malaria
95% CI
IRR
Mean (Exposure)
Absolute Humidity (g/m³) [2−Month Lag]
C1:Kalasin
Malaria
95% CI
IRR
Mean (Exposure)
Total Precipitation (mm) [1−Month Lag]
C2:Kalasin
Malaria
95% CI
IRR
Mean (Exposure)
Total Precipitation (mm) [2−Month Lag]
D1:Kalasin
Malaria
95% CI
IRR
Mean (Exposure)
Temperature (°C) [1−Month Lag]
D2:Kalasin
Malaria
95% CI
IRR
Mean (Exposure)
Temperature (°C) [2−Month Lag]
E1:Kalasin
Malaria
95% CI
IRR
Mean (Exposure)
SO_2 Surface Concentration (mg/m³) [1−Month Lag]
E2:Kalasin
Malaria
95% CI
IRR
Mean (Exposure)
SO_2 Surface Concentration (mg/m³) [2−Month Lag]
F1:Kalasin
Malaria
95% CI
IRR
Mean (Exposure)
CO Surface Concentration (ppb) [1−Month Lag]
F2:Kalasin
Malaria
95% CI
IRR
Mean (Exposure)
CO Surface Concentration (ppb) [2−Month Lag]
G1:Kalasin
Malaria
95% CI
IRR
Mean (Exposure)
$PM_{2.5}$ Surface Concentration (µg/m³) [1−Month Lag]
G2:Kalasin
Malaria
95% CI
IRR
Mean (Exposure)
$PM_{2.5}$ Surface Concentration (µg/m³) [2−Month Lag]

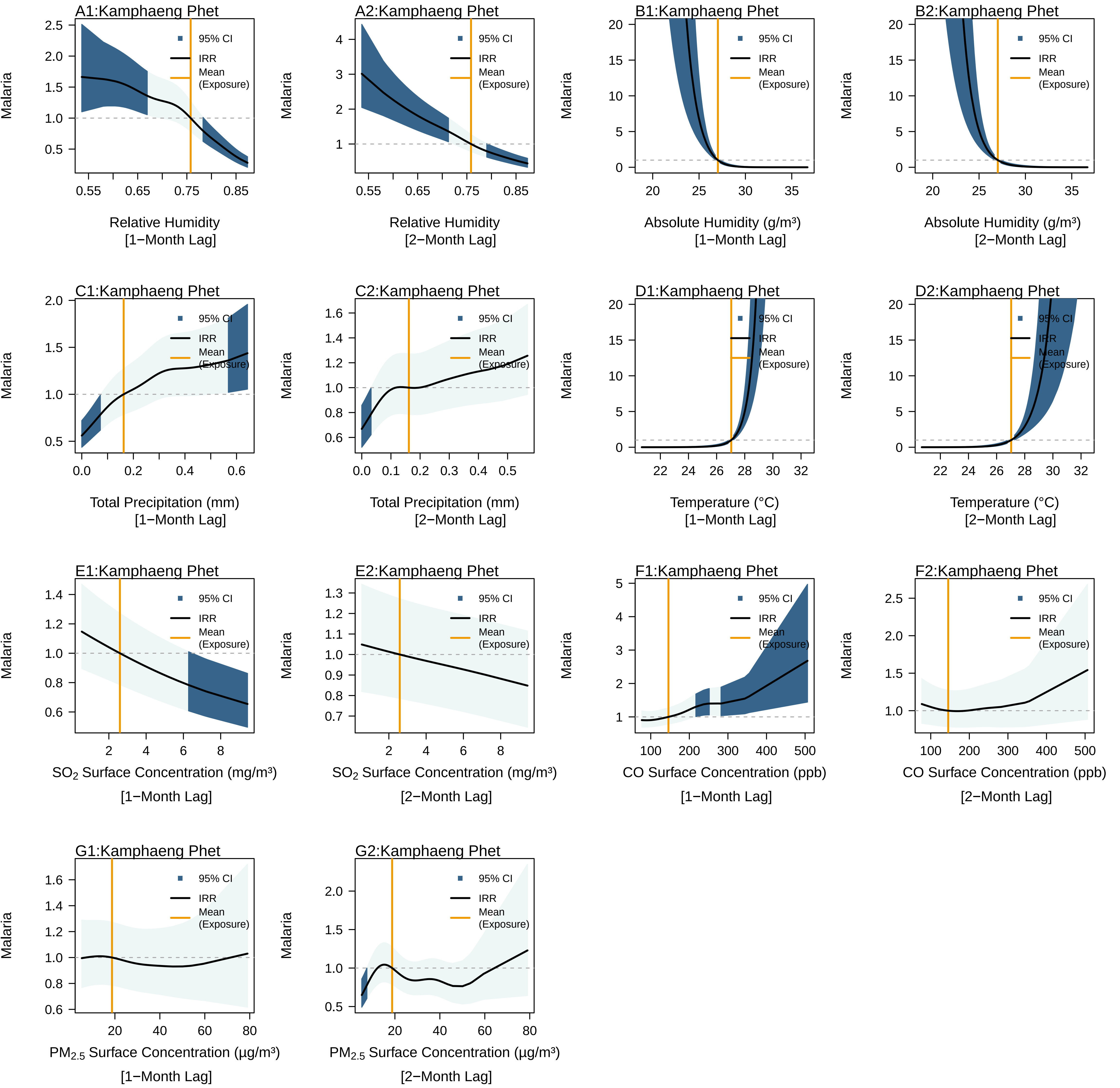

A1:Kamphaeng Phet
A2:Kamphaeng Phet
B1:Kamphaeng Phet
B2:Kamphaeng Phet
C1:Kamphaeng Phet
C2:Kamphaeng Phet
D1:Kamphaeng Phet
D2:Kamphaeng Phet
E1:Kamphaeng Phet
E2:Kamphaeng Phet
F1:Kamphaeng Phet
F2:Kamphaeng Phet
G1:Kamphaeng Phet
G2:Kamphaeng Phet
95% CI
IRR
Mean (Exposure)
Malaria
Relative Humidity [1−Month Lag]
Relative Humidity [2−Month Lag]
Absolute Humidity (g/m³) [1−Month Lag]
Absolute Humidity (g/m³) [2−Month Lag]
Total Precipitation (mm) [1−Month Lag]
Total Precipitation (mm) [2−Month Lag]
Temperature (°C) [1−Month Lag]
Temperature (°C) [2−Month Lag]
SO_2 Surface Concentration (mg/m³) [1−Month Lag]
SO_2 Surface Concentration (mg/m³) [2−Month Lag]
CO Surface Concentration (ppb) [1−Month Lag]
CO Surface Concentration (ppb) [2−Month Lag]
$PM_{2.5}$ Surface Concentration (µg/m³) [1−Month Lag]
$PM_{2.5}$ Surface Concentration (µg/m³) [2−Month Lag]

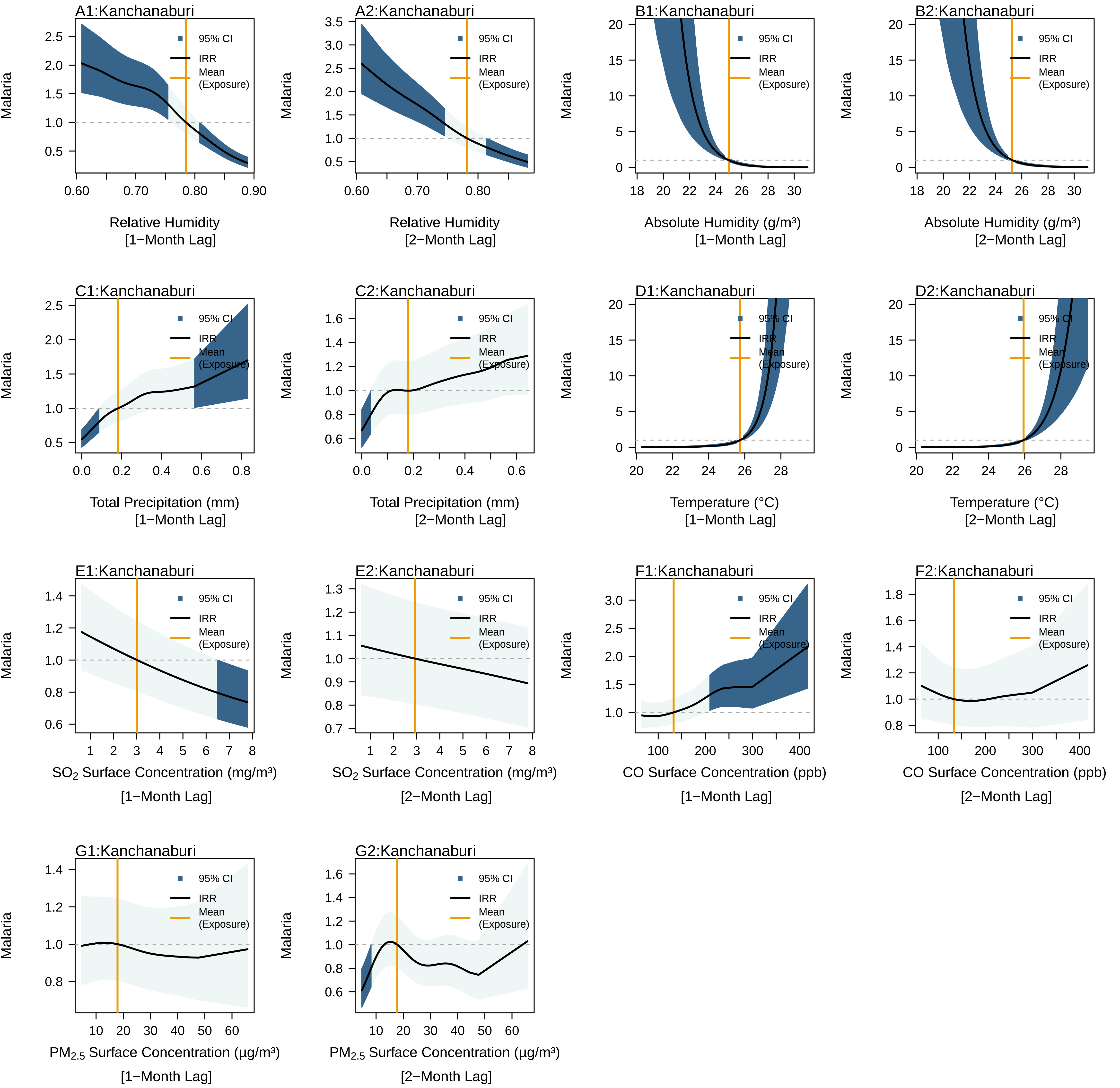

A1:Kanchanaburi
Malaria
95% CI
IRR
Mean (Exposure)
Relative Humidity [1−Month Lag]
A2:Kanchanaburi
Malaria
95% CI
IRR
Mean (Exposure)
Relative Humidity [2−Month Lag]
B1:Kanchanaburi
Malaria
95% CI
IRR
Mean (Exposure)
Absolute Humidity (g/m³) [1−Month Lag]
B2:Kanchanaburi
Malaria
95% CI
IRR
Mean (Exposure)
Absolute Humidity (g/m³) [2−Month Lag]
C1:Kanchanaburi
Malaria
95% CI
IRR
Mean (Exposure)
Total Precipitation (mm) [1−Month Lag]
C2:Kanchanaburi
Malaria
95% CI
IRR
Mean (Exposure)
Total Precipitation (mm) [2−Month Lag]
D1:Kanchanaburi
Malaria
95% CI
IRR
Mean (Exposure)
Temperature (°C) [1−Month Lag]
D2:Kanchanaburi
Malaria
95% CI
IRR
Mean (Exposure)
Temperature (°C) [2−Month Lag]
E1:Kanchanaburi
Malaria
95% CI
IRR
Mean (Exposure)
SO2 Surface Concentration (mg/m³) [1−Month Lag]
E2:Kanchanaburi
Malaria
95% CI
IRR
Mean (Exposure)
SO2 Surface Concentration (mg/m³) [2−Month Lag]
F1:Kanchanaburi
Malaria
95% CI
IRR
Mean (Exposure)
CO Surface Concentration (ppb) [1−Month Lag]
F2:Kanchanaburi
Malaria
95% CI
IRR
Mean (Exposure)
CO Surface Concentration (ppb) [2−Month Lag]
G1:Kanchanaburi
Malaria
95% CI
IRR
Mean (Exposure)
PM2.5 Surface Concentration (µg/m³) [1−Month Lag]
G2:Kanchanaburi
Malaria
95% CI
IRR
Mean (Exposure)
PM2.5 Surface Concentration (µg/m³) [2−Month Lag]

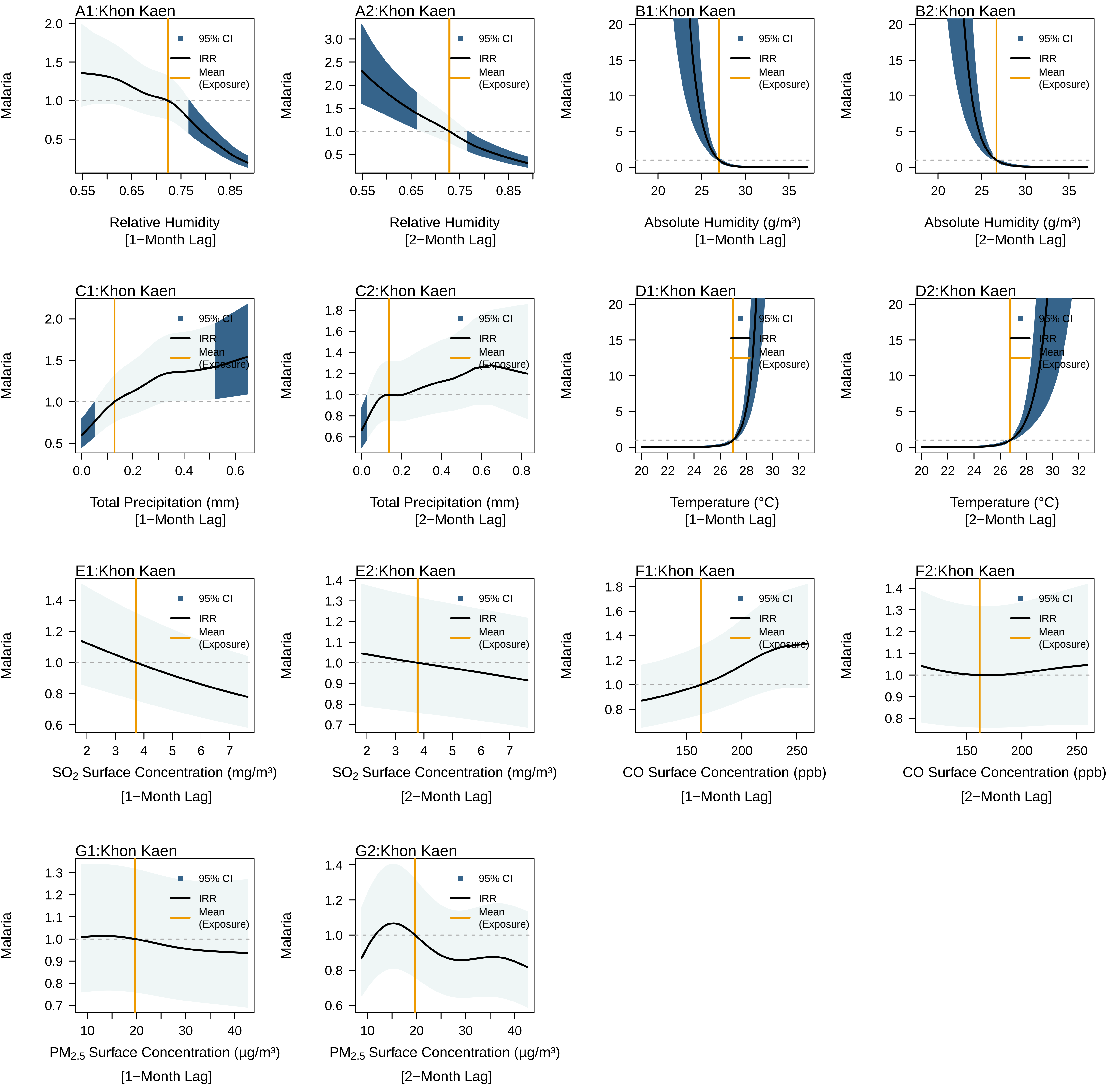

A1:Khon Kaen
Malaria
Relative Humidity
[1−Month Lag]
A2:Khon Kaen
Relative Humidity
[2−Month Lag]
B1:Khon Kaen
Absolute Humidity (g/m³)
[1−Month Lag]
B2:Khon Kaen
Absolute Humidity (g/m³)
[2−Month Lag]
C1:Khon Kaen
Total Precipitation (mm)
[1−Month Lag]
C2:Khon Kaen
Total Precipitation (mm)
[2−Month Lag]
D1:Khon Kaen
Temperature (°C)
[1−Month Lag]
D2:Khon Kaen
Temperature (°C)
[2−Month Lag]
E1:Khon Kaen
SO2 Surface Concentration (mg/m³)
[1−Month Lag]
E2:Khon Kaen
SO2 Surface Concentration (mg/m³)
[2−Month Lag]
F1:Khon Kaen
CO Surface Concentration (ppb)
[1−Month Lag]
F2:Khon Kaen
CO Surface Concentration (ppb)
[2−Month Lag]
G1:Khon Kaen
PM2.5 Surface Concentration (µg/m³)
[1−Month Lag]
G2:Khon Kaen
PM2.5 Surface Concentration (µg/m³)
[2−Month Lag]
95% CI
IRR
Mean
(Exposure)

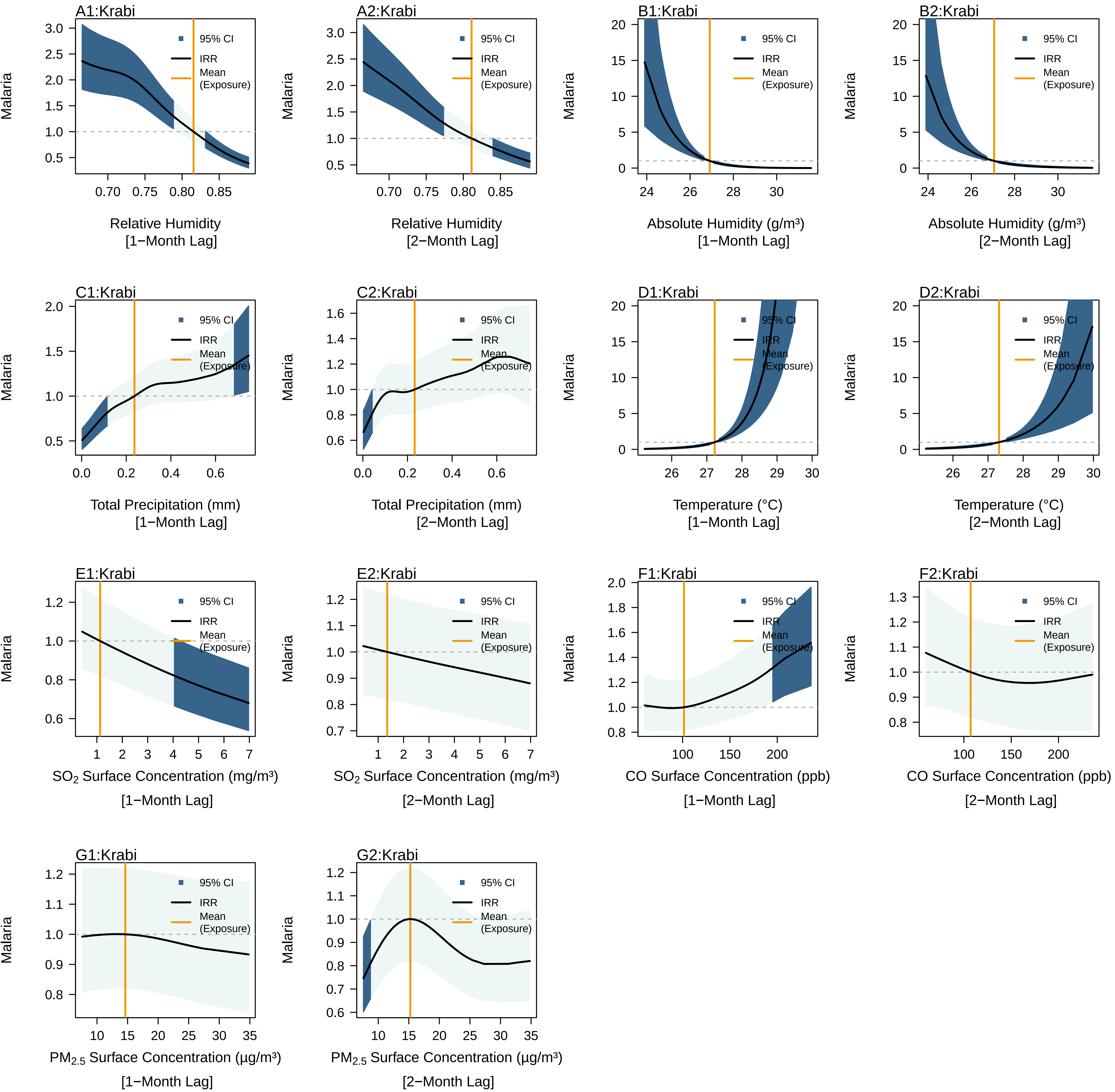
A1:Krabi
95% CI
IRR
Mean (Exposure)
Malaria
Relative Humidity [1−Month Lag]
A2:Krabi
Relative Humidity [2−Month Lag]
B1:Krabi
Absolute Humidity (g/m³) [1−Month Lag]
B2:Krabi
Absolute Humidity (g/m³) [2−Month Lag]
C1:Krabi
Total Precipitation (mm) [1−Month Lag]
C2:Krabi
Total Precipitation (mm) [2−Month Lag]
D1:Krabi
Temperature (°C) [1−Month Lag]
D2:Krabi
Temperature (°C) [2−Month Lag]
E1:Krabi
SO_2 Surface Concentration (mg/m³) [1−Month Lag]
E2:Krabi
SO_2 Surface Concentration (mg/m³) [2−Month Lag]
F1:Krabi
CO Surface Concentration (ppb) [1−Month Lag]
F2:Krabi
CO Surface Concentration (ppb) [2−Month Lag]
G1:Krabi
$PM_{2.5}$ Surface Concentration (µg/m³) [1−Month Lag]
G2:Krabi
$PM_{2.5}$ Surface Concentration (µg/m³) [2−Month Lag]

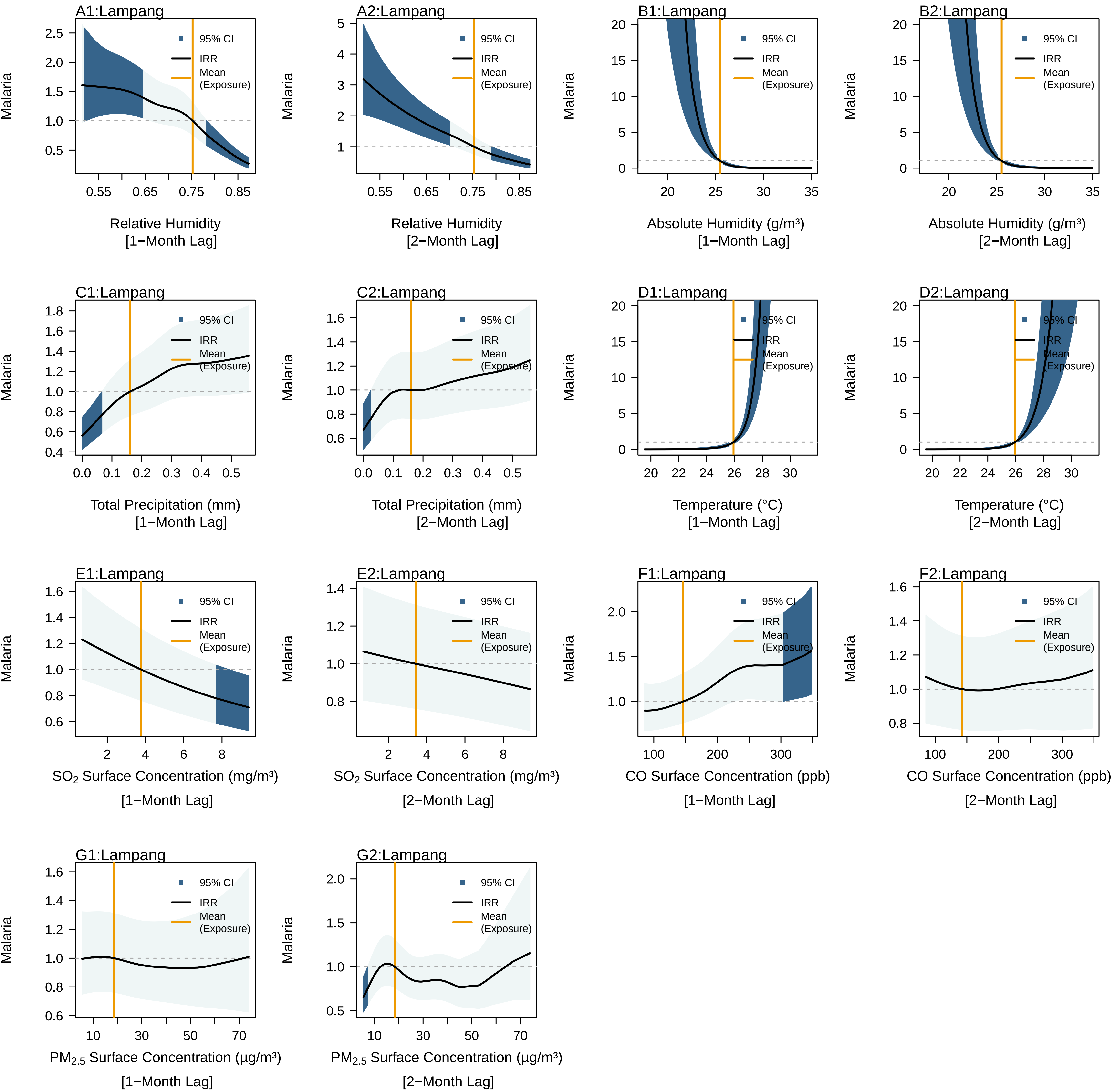
A1:Lampang
A2:Lampang
B1:Lampang
B2:Lampang
C1:Lampang
C2:Lampang
D1:Lampang
D2:Lampang
E1:Lampang
E2:Lampang
F1:Lampang
F2:Lampang
G1:Lampang
G2:Lampang
95% CI
IRR
Mean (Exposure)
Malaria
Relative Humidity [1−Month Lag]
Relative Humidity [2−Month Lag]
Absolute Humidity (g/m³) [1−Month Lag]
Absolute Humidity (g/m³) [2−Month Lag]
Total Precipitation (mm) [1−Month Lag]
Total Precipitation (mm) [2−Month Lag]
Temperature (°C) [1−Month Lag]
Temperature (°C) [2−Month Lag]
SO_2 Surface Concentration (mg/m³) [1−Month Lag]
SO_2 Surface Concentration (mg/m³) [2−Month Lag]
CO Surface Concentration (ppb) [1−Month Lag]
CO Surface Concentration (ppb) [2−Month Lag]
$PM_{2.5}$ Surface Concentration (µg/m³) [1−Month Lag]
$PM_{2.5}$ Surface Concentration (µg/m³) [2−Month Lag]

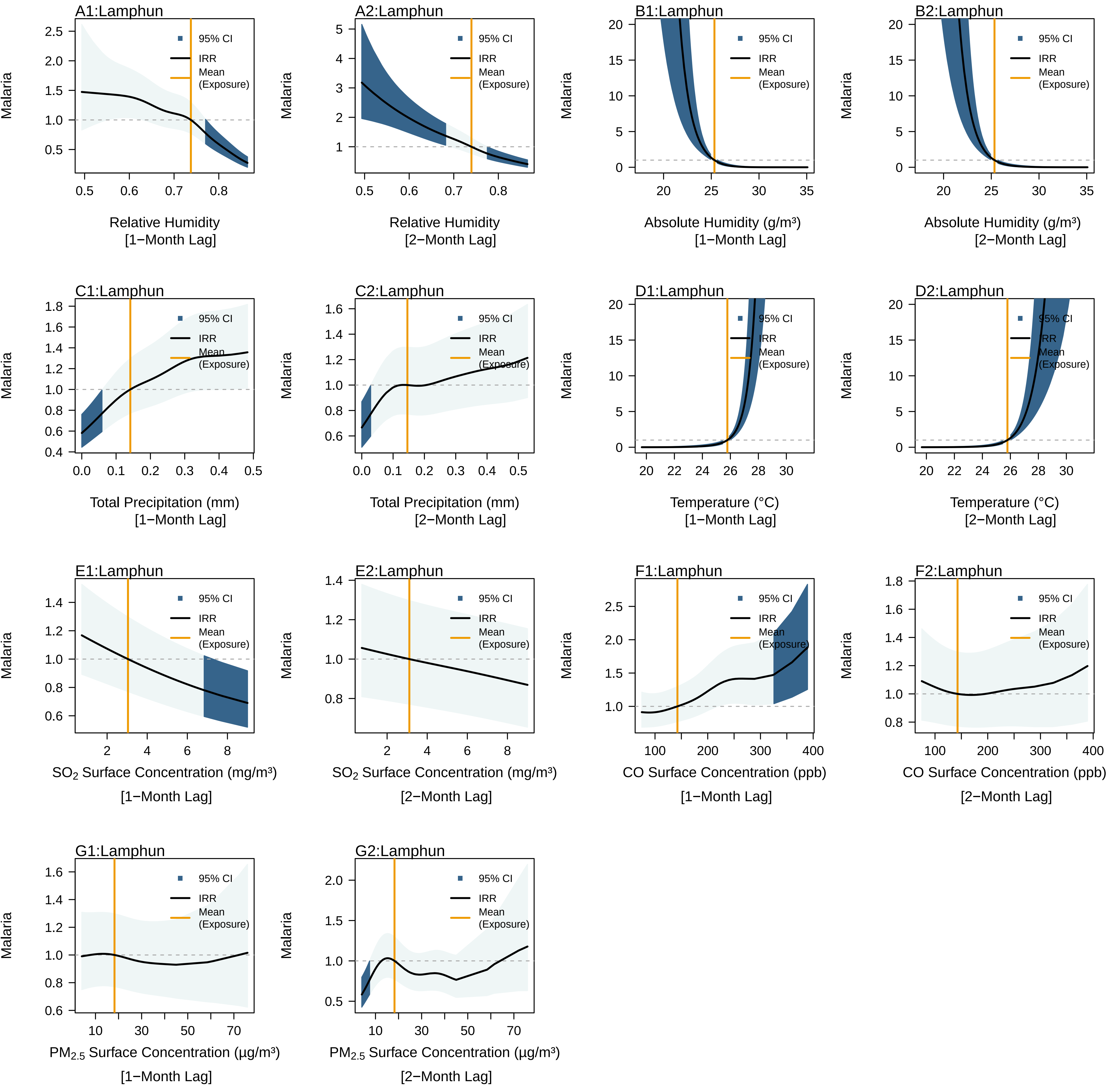
A1:Lamphun
95% CI
IRR
Mean (Exposure)
Malaria
Relative Humidity [1−Month Lag]
A2:Lamphun
Relative Humidity [2−Month Lag]
B1:Lamphun
Absolute Humidity (g/m³) [1−Month Lag]
B2:Lamphun
Absolute Humidity (g/m³) [2−Month Lag]
C1:Lamphun
Total Precipitation (mm) [1−Month Lag]
C2:Lamphun
Total Precipitation (mm) [2−Month Lag]
D1:Lamphun
Temperature (°C) [1−Month Lag]
D2:Lamphun
Temperature (°C) [2−Month Lag]
E1:Lamphun
SO_2 Surface Concentration (mg/m³) [1−Month Lag]
E2:Lamphun
SO_2 Surface Concentration (mg/m³) [2−Month Lag]
F1:Lamphun
CO Surface Concentration (ppb) [1−Month Lag]
F2:Lamphun
CO Surface Concentration (ppb) [2−Month Lag]
G1:Lamphun
$PM_{2.5}$ Surface Concentration (µg/m³) [1−Month Lag]
G2:Lamphun
$PM_{2.5}$ Surface Concentration (µg/m³) [2−Month Lag]

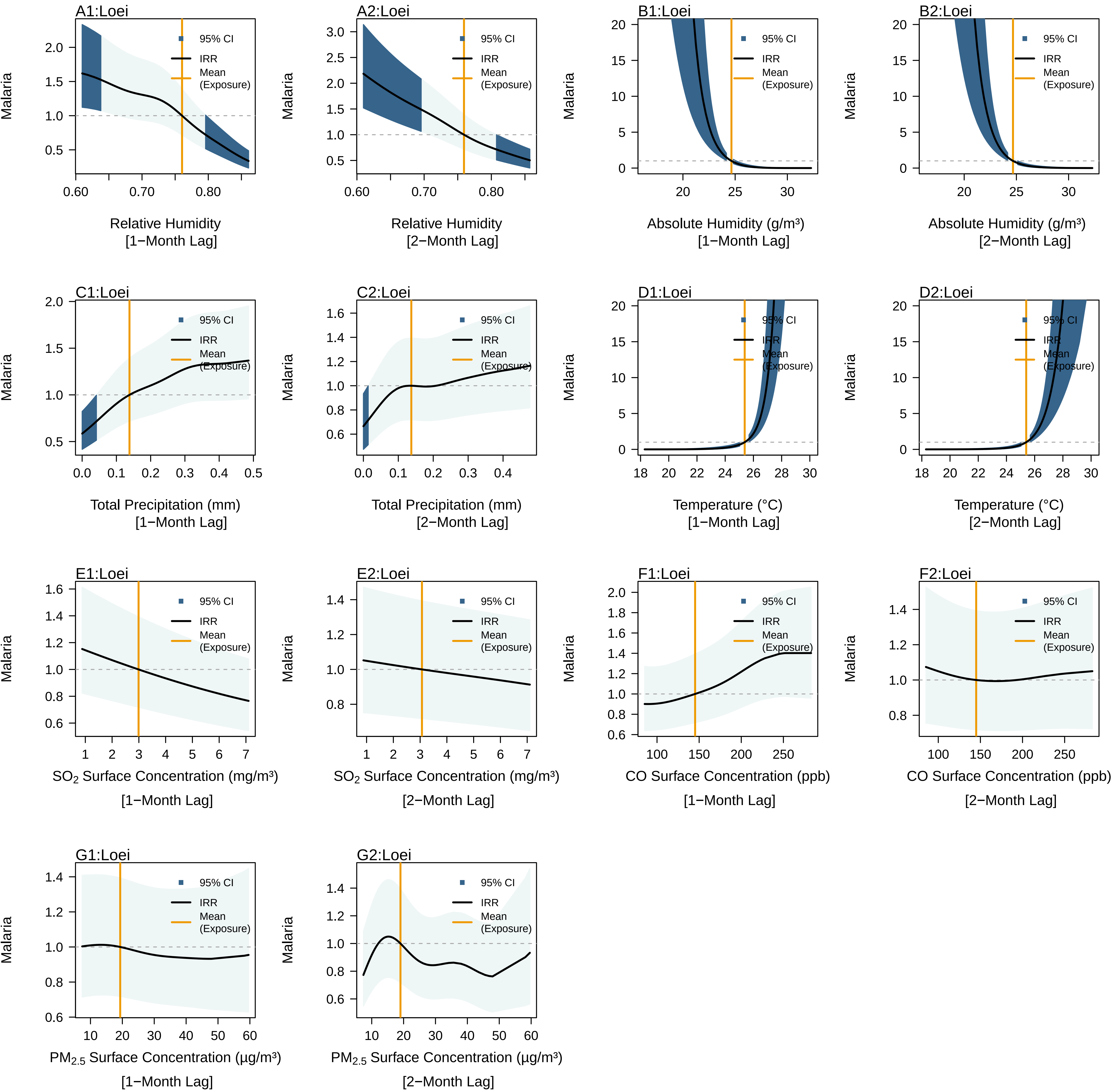
A1:Loei
A2:Loei
B1:Loei
B2:Loei
95% CI
IRR
Mean (Exposure)
Malaria
Relative Humidity [1−Month Lag]
Relative Humidity [2−Month Lag]
Absolute Humidity (g/m³) [1−Month Lag]
Absolute Humidity (g/m³) [2−Month Lag]
C1:Loei
C2:Loei
D1:Loei
D2:Loei
Total Precipitation (mm) [1−Month Lag]
Total Precipitation (mm) [2−Month Lag]
Temperature (°C) [1−Month Lag]
Temperature (°C) [2−Month Lag]
E1:Loei
E2:Loei
F1:Loei
F2:Loei
SO_2 Surface Concentration (mg/m³) [1−Month Lag]
SO_2 Surface Concentration (mg/m³) [2−Month Lag]
CO Surface Concentration (ppb) [1−Month Lag]
CO Surface Concentration (ppb) [2−Month Lag]
G1:Loei
G2:Loei
$PM_{2.5}$ Surface Concentration (µg/m³) [1−Month Lag]
$PM_{2.5}$ Surface Concentration (µg/m³) [2−Month Lag]

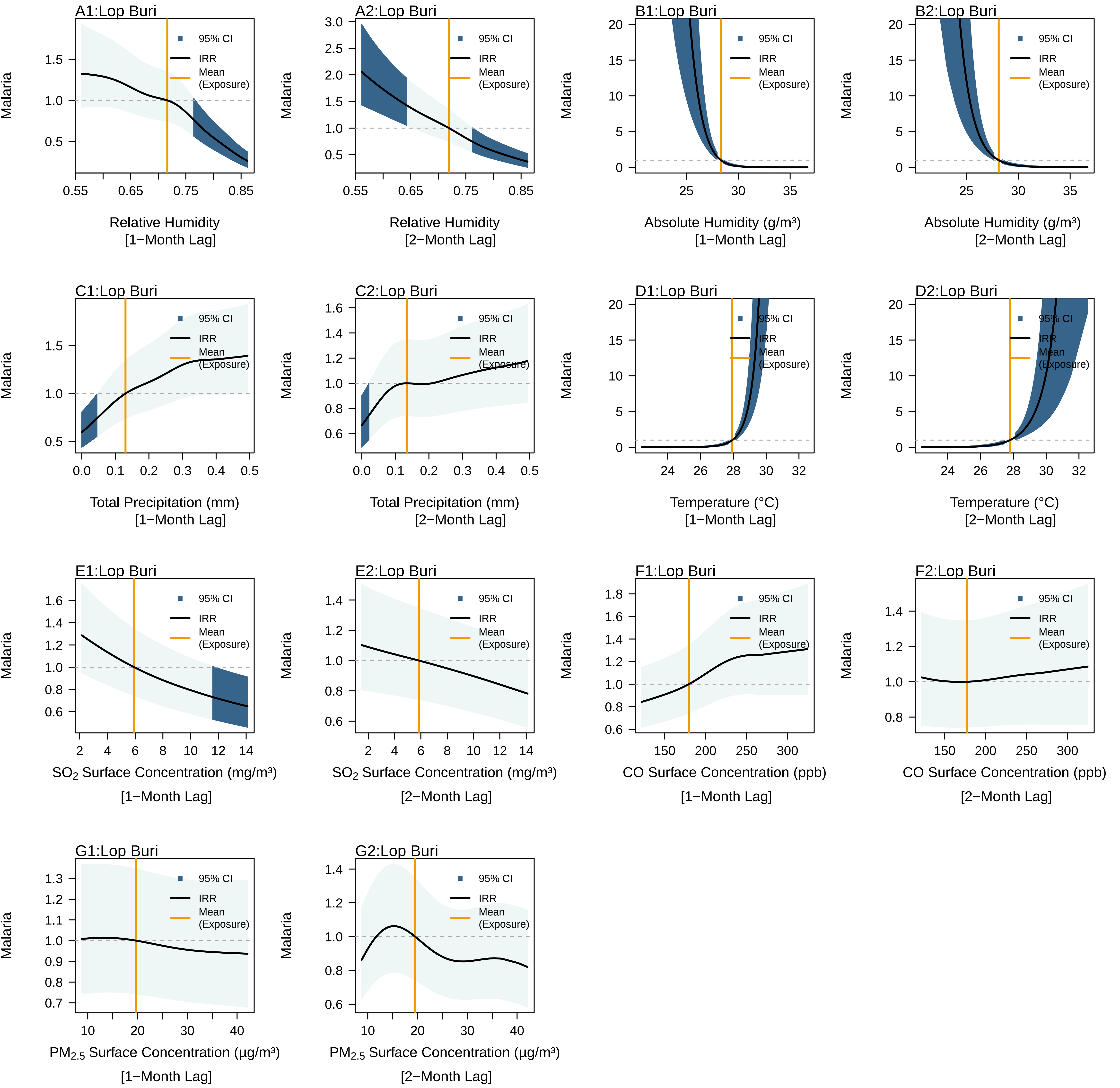
A1:Lop Buri
Malaria
Relative Humidity
[1−Month Lag]
A2:Lop Buri
Relative Humidity
[2−Month Lag]
B1:Lop Buri
Absolute Humidity (g/m³)
[1−Month Lag]
B2:Lop Buri
Absolute Humidity (g/m³)
[2−Month Lag]
C1:Lop Buri
Total Precipitation (mm)
[1−Month Lag]
C2:Lop Buri
Total Precipitation (mm)
[2−Month Lag]
D1:Lop Buri
Temperature (°C)
[1−Month Lag]
D2:Lop Buri
Temperature (°C)
[2−Month Lag]
E1:Lop Buri
SO_2 Surface Concentration (mg/m³)
[1−Month Lag]
E2:Lop Buri
SO_2 Surface Concentration (mg/m³)
[2−Month Lag]
F1:Lop Buri
CO Surface Concentration (ppb)
[1−Month Lag]
F2:Lop Buri
CO Surface Concentration (ppb)
[2−Month Lag]
G1:Lop Buri
$PM_{2.5}$ Surface Concentration (μg/m³)
[1−Month Lag]
G2:Lop Buri
$PM_{2.5}$ Surface Concentration (μg/m³)
[2−Month Lag]
95% CI
IRR
Mean (Exposure)

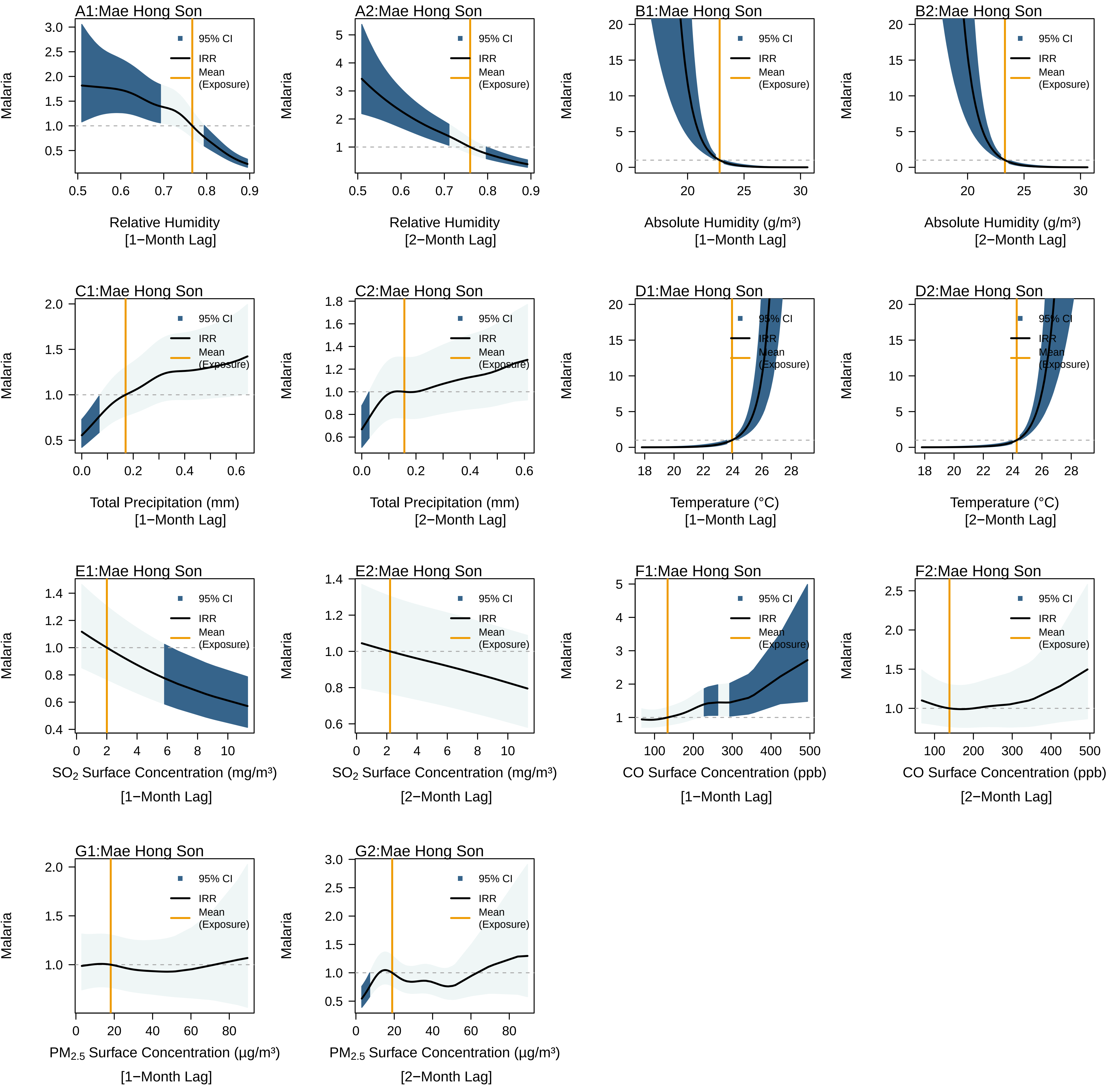
A1:Mae Hong Son
95% CI
IRR
Mean (Exposure)
Malaria
Relative Humidity [1−Month Lag]
A2:Mae Hong Son
Relative Humidity [2−Month Lag]
B1:Mae Hong Son
Absolute Humidity (g/m³) [1−Month Lag]
B2:Mae Hong Son
Absolute Humidity (g/m³) [2−Month Lag]
C1:Mae Hong Son
Total Precipitation (mm) [1−Month Lag]
C2:Mae Hong Son
Total Precipitation (mm) [2−Month Lag]
D1:Mae Hong Son
Temperature (°C) [1−Month Lag]
D2:Mae Hong Son
Temperature (°C) [2−Month Lag]
E1:Mae Hong Son
SO_2 Surface Concentration (mg/m³) [1−Month Lag]
E2:Mae Hong Son
SO_2 Surface Concentration (mg/m³) [2−Month Lag]
F1:Mae Hong Son
CO Surface Concentration (ppb) [1−Month Lag]
F2:Mae Hong Son
CO Surface Concentration (ppb) [2−Month Lag]
G1:Mae Hong Son
$PM_{2.5}$ Surface Concentration (µg/m³) [1−Month Lag]
G2:Mae Hong Son
$PM_{2.5}$ Surface Concentration (µg/m³) [2−Month Lag]

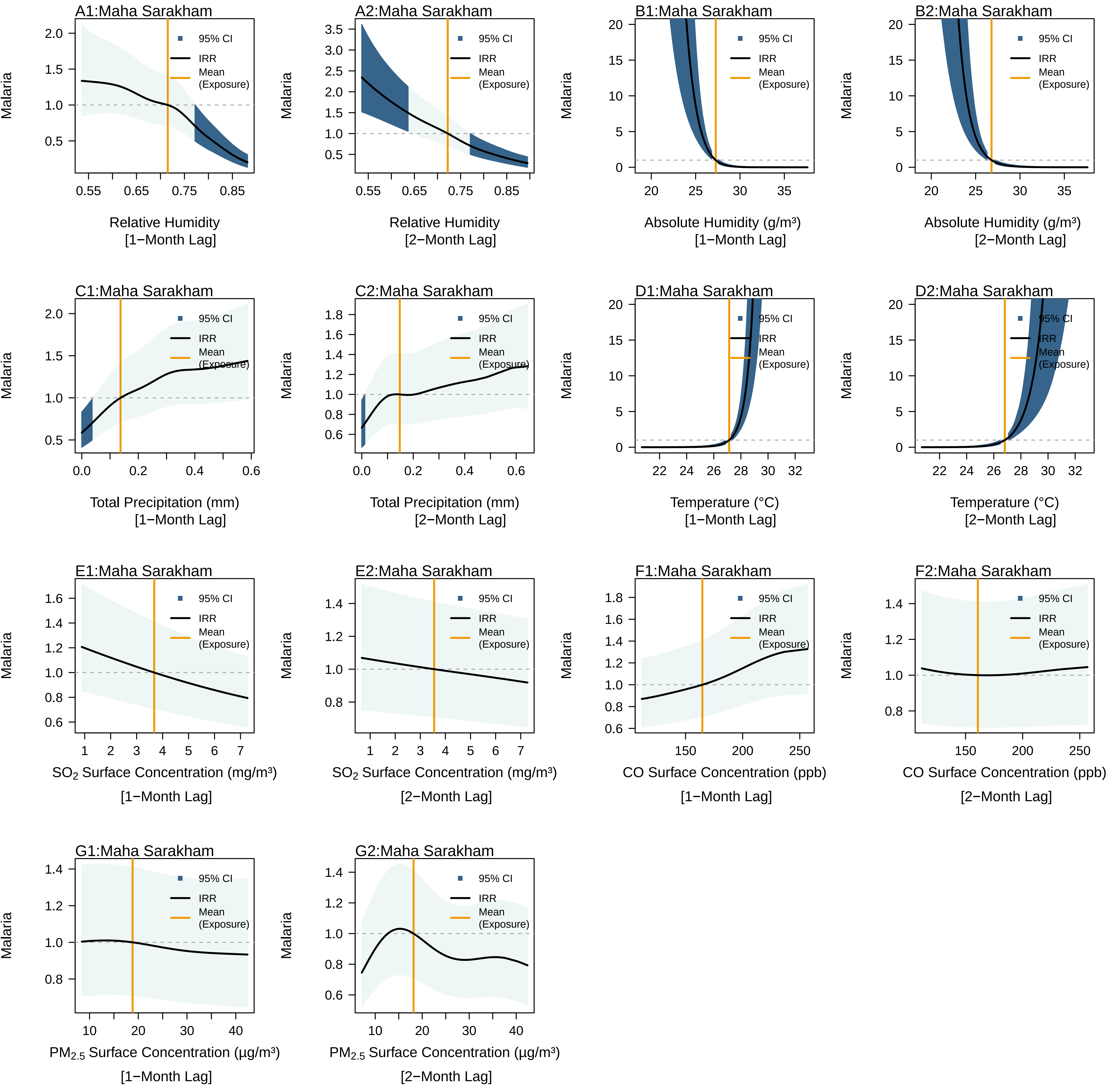

A1:Maha Sarakham
Malaria
2.0 1.5 1.0 0.5
0.55 0.65 0.75 0.85
95% CI
IRR
Mean (Exposure)
Relative Humidity [1−Month Lag]
A2:Maha Sarakham
Malaria
3.5 3.0 2.5 2.0 1.5 1.0 0.5
0.55 0.65 0.75 0.85
95% CI
IRR
Mean (Exposure)
Relative Humidity [2−Month Lag]
B1:Maha Sarakham
Malaria
20 15 10 5 0
20 25 30 35
95% CI
IRR
Mean (Exposure)
Absolute Humidity (g/m³) [1−Month Lag]
B2:Maha Sarakham
Malaria
20 15 10 5 0
20 25 30 35
95% CI
IRR
Mean (Exposure)
Absolute Humidity (g/m³) [2−Month Lag]
C1:Maha Sarakham
Malaria
2.0 1.5 1.0 0.5
0.0 0.2 0.4 0.6
95% CI
IRR
Mean (Exposure)
Total Precipitation (mm) [1−Month Lag]
C2:Maha Sarakham
Malaria
1.8 1.6 1.4 1.2 1.0 0.8 0.6
0.0 0.2 0.4 0.6
95% CI
IRR
Mean (Exposure)
Total Precipitation (mm) [2−Month Lag]
D1:Maha Sarakham
Malaria
20 15 10 5 0
22 24 26 28 30 32
95% CI
IRR
Mean (Exposure)
Temperature (°C) [1−Month Lag]
D2:Maha Sarakham
Malaria
20 15 10 5 0
22 24 26 28 30 32
95% CI
IRR
Mean (Exposure)
Temperature (°C) [2−Month Lag]
E1:Maha Sarakham
Malaria
1.6 1.4 1.2 1.0 0.8 0.6
1 2 3 4 5 6 7
95% CI
IRR
Mean (Exposure)
SO2 Surface Concentration (mg/m³) [1−Month Lag]
E2:Maha Sarakham
Malaria
1.4 1.2 1.0 0.8
1 2 3 4 5 6 7
95% CI
IRR
Mean (Exposure)
SO2 Surface Concentration (mg/m³) [2−Month Lag]
F1:Maha Sarakham
Malaria
1.8 1.6 1.4 1.2 1.0 0.8 0.6
150 200 250
95% CI
IRR
Mean (Exposure)
CO Surface Concentration (ppb) [1−Month Lag]
F2:Maha Sarakham
Malaria
1.4 1.2 1.0 0.8
150 200 250
95% CI
IRR
Mean (Exposure)
CO Surface Concentration (ppb) [2−Month Lag]
G1:Maha Sarakham
Malaria
1.4 1.2 1.0 0.8
10 20 30 40
95% CI
IRR
Mean (Exposure)
PM2.5 Surface Concentration (µg/m³) [1−Month Lag]
G2:Maha Sarakham
Malaria
1.4 1.2 1.0 0.8 0.6
10 20 30 40
95% CI
IRR
Mean (Exposure)
PM2.5 Surface Concentration (µg/m³) [2−Month Lag]

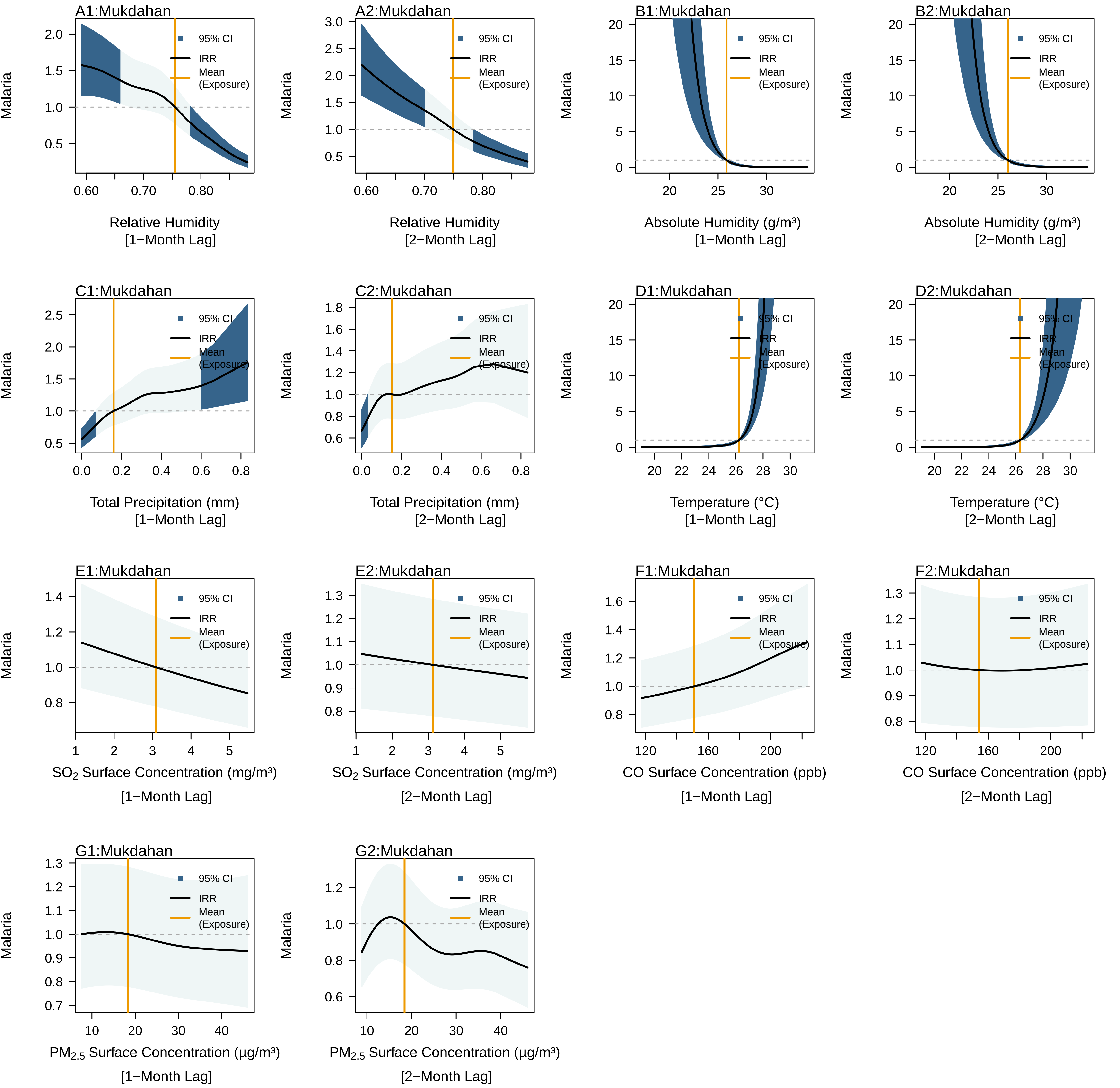
A1:Mukdahan
A2:Mukdahan
B1:Mukdahan
B2:Mukdahan
C1:Mukdahan
C2:Mukdahan
D1:Mukdahan
D2:Mukdahan
E1:Mukdahan
E2:Mukdahan
F1:Mukdahan
F2:Mukdahan
G1:Mukdahan
G2:Mukdahan
Malaria
95% CI
IRR
Mean (Exposure)
Relative Humidity [1−Month Lag]
Relative Humidity [2−Month Lag]
Absolute Humidity (g/m³) [1−Month Lag]
Absolute Humidity (g/m³) [2−Month Lag]
Total Precipitation (mm) [1−Month Lag]
Total Precipitation (mm) [2−Month Lag]
Temperature (°C) [1−Month Lag]
Temperature (°C) [2−Month Lag]
SO_2 Surface Concentration (mg/m³) [1−Month Lag]
SO_2 Surface Concentration (mg/m³) [2−Month Lag]
CO Surface Concentration (ppb) [1−Month Lag]
CO Surface Concentration (ppb) [2−Month Lag]
$PM_{2.5}$ Surface Concentration (µg/m³) [1−Month Lag]
$PM_{2.5}$ Surface Concentration (µg/m³) [2−Month Lag]

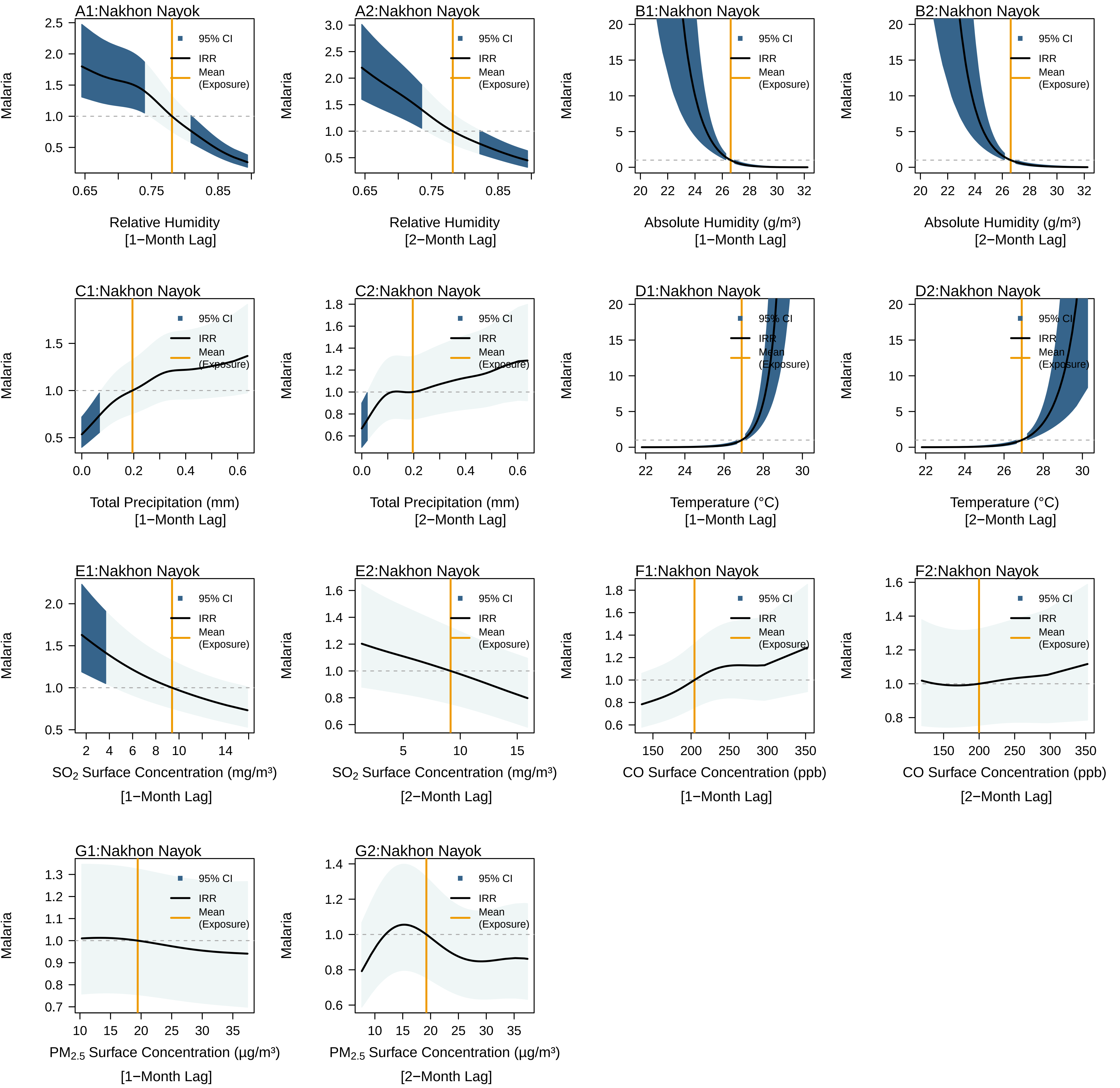
A1:Nakhon Nayok
Malaria
95% CI
IRR
Mean (Exposure)
Relative Humidity [1−Month Lag]
A2:Nakhon Nayok
Malaria
95% CI
IRR
Mean (Exposure)
Relative Humidity [2−Month Lag]
B1:Nakhon Nayok
Malaria
95% CI
IRR
Mean (Exposure)
Absolute Humidity (g/m³) [1−Month Lag]
B2:Nakhon Nayok
Malaria
95% CI
IRR
Mean (Exposure)
Absolute Humidity (g/m³) [2−Month Lag]
C1:Nakhon Nayok
Malaria
95% CI
IRR
Mean (Exposure)
Total Precipitation (mm) [1−Month Lag]
C2:Nakhon Nayok
Malaria
95% CI
IRR
Mean (Exposure)
Total Precipitation (mm) [2−Month Lag]
D1:Nakhon Nayok
Malaria
95% CI
IRR
Mean (Exposure)
Temperature (°C) [1−Month Lag]
D2:Nakhon Nayok
Malaria
95% CI
IRR
Mean (Exposure)
Temperature (°C) [2−Month Lag]
E1:Nakhon Nayok
Malaria
95% CI
IRR
Mean (Exposure)
SO2 Surface Concentration (mg/m³) [1−Month Lag]
E2:Nakhon Nayok
Malaria
95% CI
IRR
Mean (Exposure)
SO2 Surface Concentration (mg/m³) [2−Month Lag]
F1:Nakhon Nayok
Malaria
95% CI
IRR
Mean (Exposure)
CO Surface Concentration (ppb) [1−Month Lag]
F2:Nakhon Nayok
Malaria
95% CI
IRR
Mean (Exposure)
CO Surface Concentration (ppb) [2−Month Lag]
G1:Nakhon Nayok
Malaria
95% CI
IRR
Mean (Exposure)
PM2.5 Surface Concentration (µg/m³) [1−Month Lag]
G2:Nakhon Nayok
Malaria
95% CI
IRR
Mean (Exposure)
PM2.5 Surface Concentration (µg/m³) [2−Month Lag]

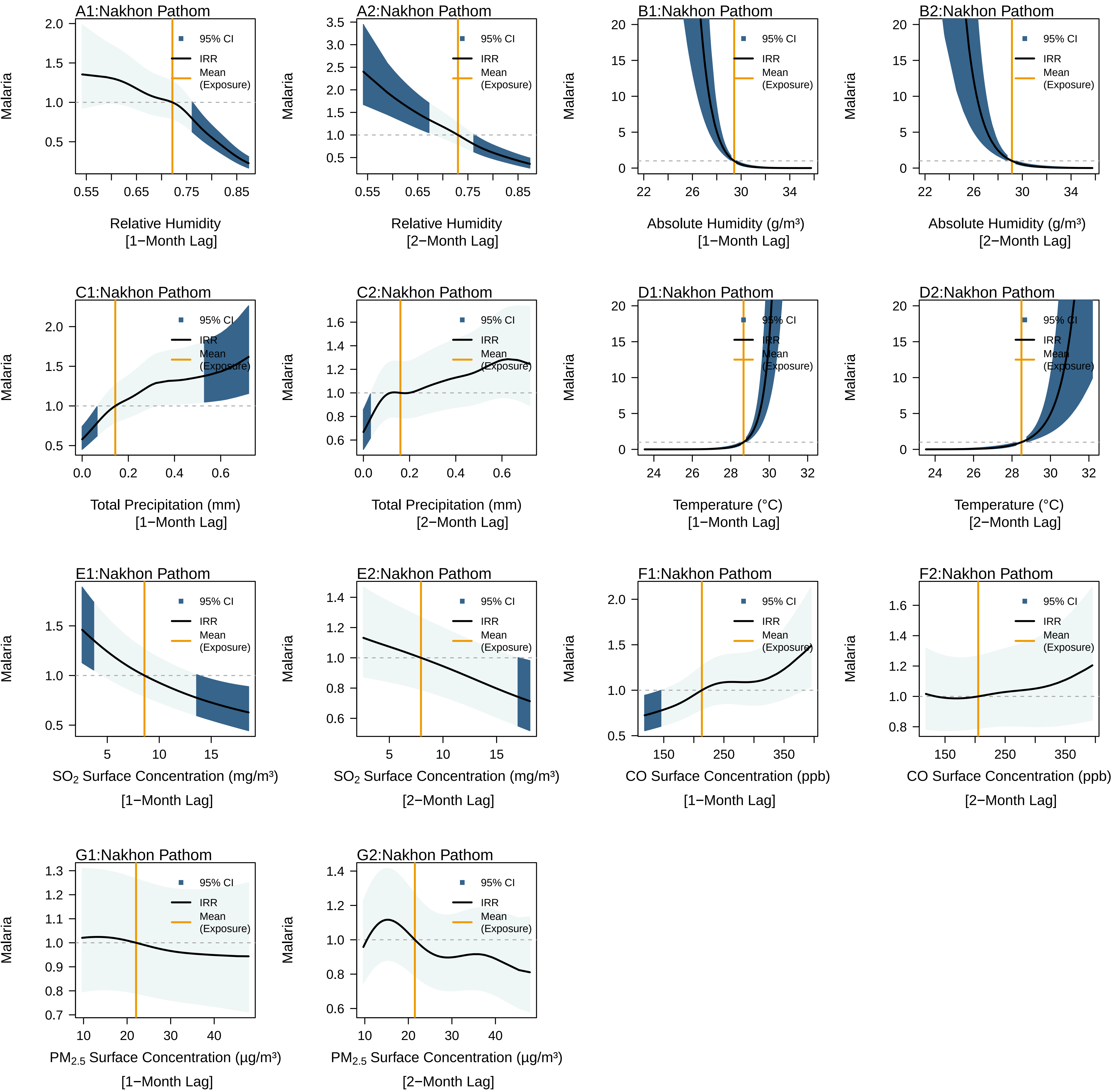

A1:Nakhon Pathom
A2:Nakhon Pathom
B1:Nakhon Pathom
B2:Nakhon Pathom
Malaria
95% CI
IRR
Mean
(Exposure)
Relative Humidity
[1−Month Lag]
Relative Humidity
[2−Month Lag]
Absolute Humidity (g/m³)
[1−Month Lag]
Absolute Humidity (g/m³)
[2−Month Lag]
C1:Nakhon Pathom
C2:Nakhon Pathom
D1:Nakhon Pathom
D2:Nakhon Pathom
Total Precipitation (mm)
[1−Month Lag]
Total Precipitation (mm)
[2−Month Lag]
Temperature (°C)
[1−Month Lag]
Temperature (°C)
[2−Month Lag]
E1:Nakhon Pathom
E2:Nakhon Pathom
F1:Nakhon Pathom
F2:Nakhon Pathom
SO_2 Surface Concentration (mg/m³)
[1−Month Lag]
SO_2 Surface Concentration (mg/m³)
[2−Month Lag]
CO Surface Concentration (ppb)
[1−Month Lag]
CO Surface Concentration (ppb)
[2−Month Lag]
G1:Nakhon Pathom
G2:Nakhon Pathom
$PM_{2.5}$ Surface Concentration (µg/m³)
[1−Month Lag]
$PM_{2.5}$ Surface Concentration (µg/m³)
[2−Month Lag]

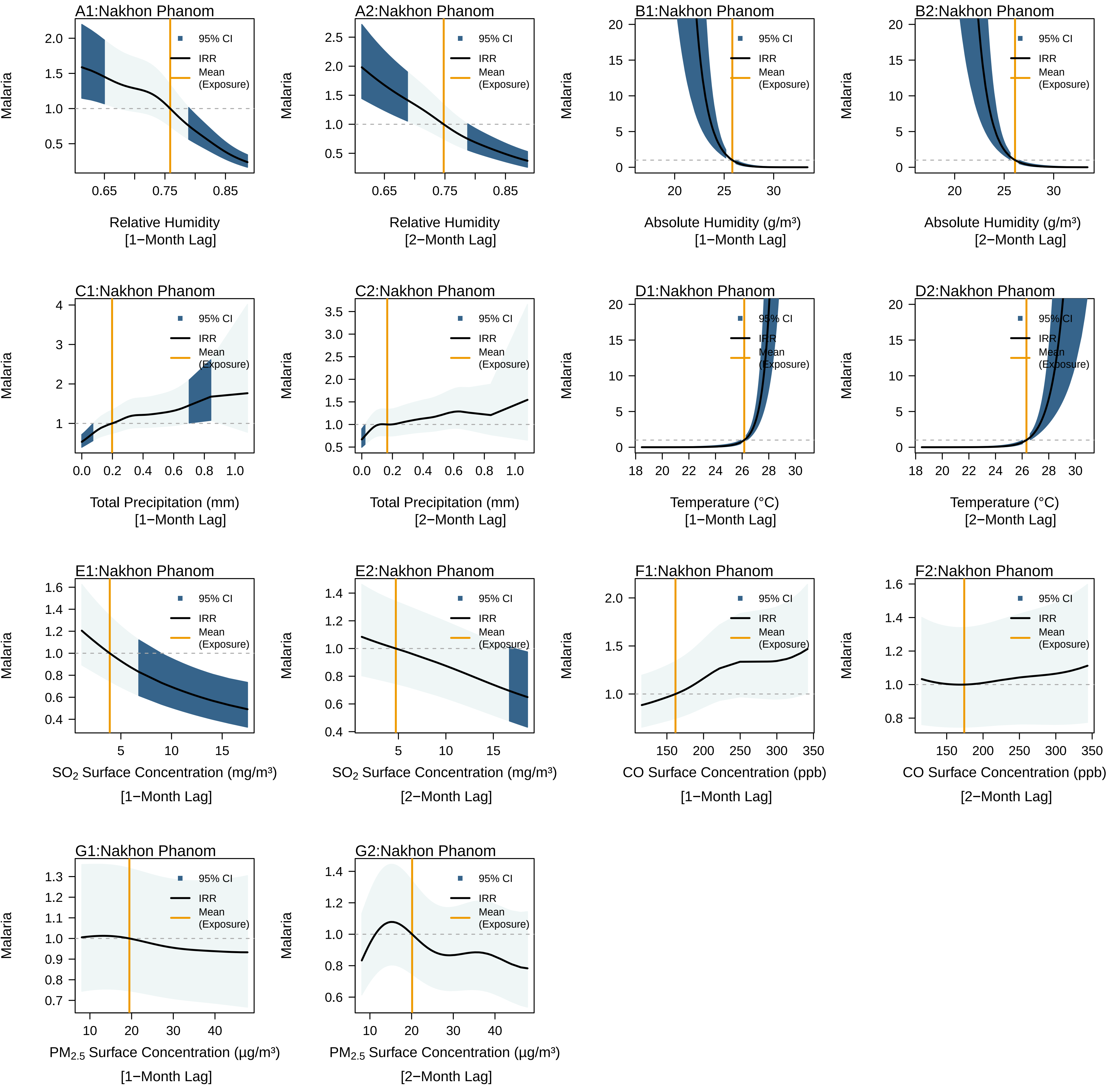
A1:Nakhon Phanom
A2:Nakhon Phanom
B1:Nakhon Phanom
B2:Nakhon Phanom
C1:Nakhon Phanom
C2:Nakhon Phanom
D1:Nakhon Phanom
D2:Nakhon Phanom
E1:Nakhon Phanom
E2:Nakhon Phanom
F1:Nakhon Phanom
F2:Nakhon Phanom
G1:Nakhon Phanom
G2:Nakhon Phanom
Malaria
95% CI
IRR
Mean (Exposure)
Relative Humidity [1−Month Lag]
Relative Humidity [2−Month Lag]
Absolute Humidity (g/m³) [1−Month Lag]
Absolute Humidity (g/m³) [2−Month Lag]
Total Precipitation (mm) [1−Month Lag]
Total Precipitation (mm) [2−Month Lag]
Temperature (°C) [1−Month Lag]
Temperature (°C) [2−Month Lag]
SO2 Surface Concentration (mg/m³) [1−Month Lag]
SO2 Surface Concentration (mg/m³) [2−Month Lag]
CO Surface Concentration (ppb) [1−Month Lag]
CO Surface Concentration (ppb) [2−Month Lag]
PM2.5 Surface Concentration (µg/m³) [1−Month Lag]
PM2.5 Surface Concentration (µg/m³) [2−Month Lag]

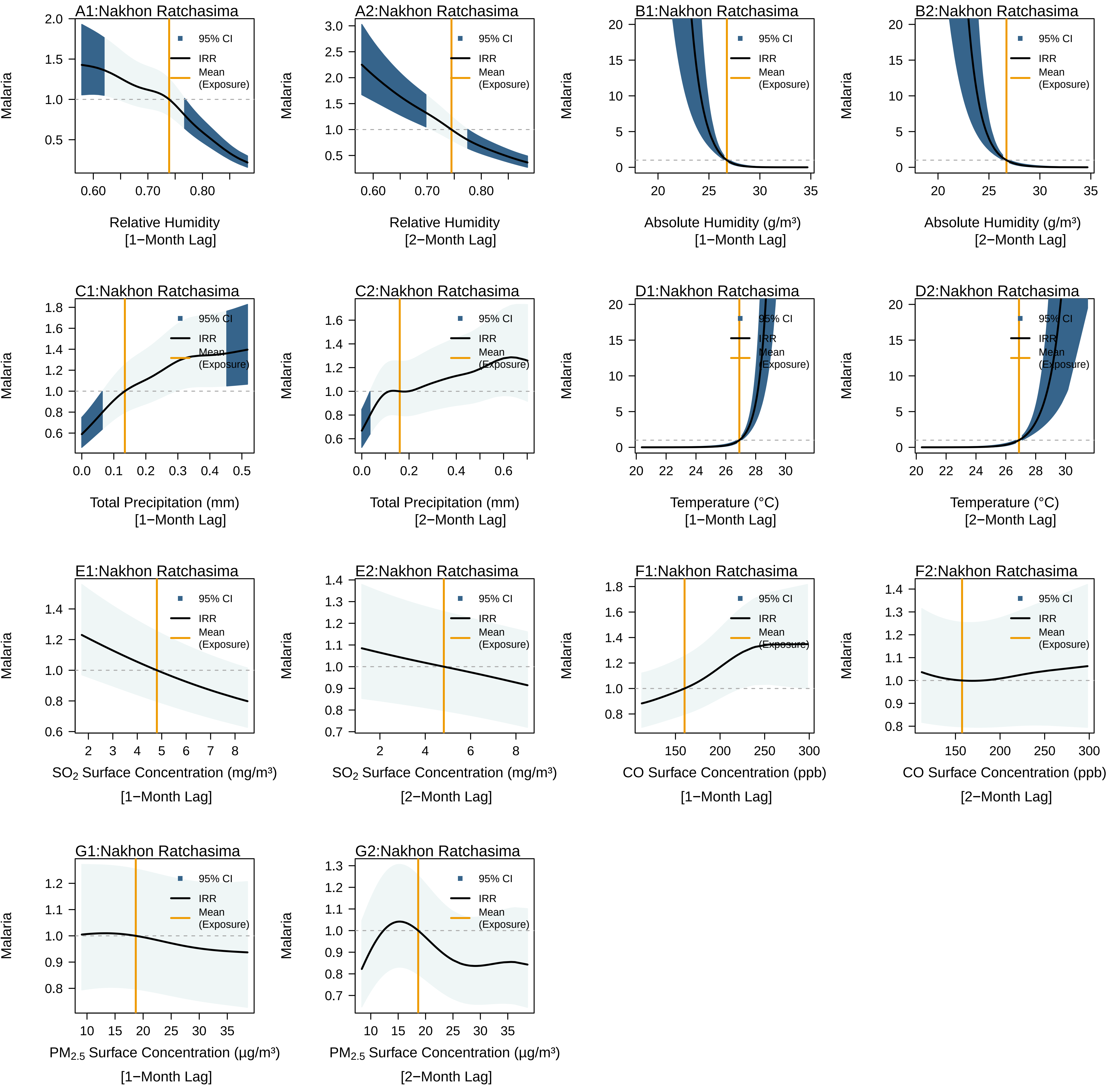
A1:Nakhon Ratchasima
Malaria
2.0 1.5 1.0 0.5
0.60 0.70 0.80
95% CI
IRR
Mean (Exposure)
Relative Humidity [1−Month Lag]
A2:Nakhon Ratchasima
Malaria
3.0 2.5 2.0 1.5 1.0 0.5
0.60 0.70 0.80
95% CI
IRR
Mean (Exposure)
Relative Humidity [2−Month Lag]
B1:Nakhon Ratchasima
Malaria
20 15 10 5 0
20 25 30 35
95% CI
IRR
Mean (Exposure)
Absolute Humidity (g/m³) [1−Month Lag]
B2:Nakhon Ratchasima
Malaria
20 15 10 5 0
20 25 30 35
95% CI
IRR
Mean (Exposure)
Absolute Humidity (g/m³) [2−Month Lag]
C1:Nakhon Ratchasima
Malaria
1.8 1.6 1.4 1.2 1.0 0.8 0.6
0.0 0.1 0.2 0.3 0.4 0.5
95% CI
IRR
Mean (Exposure)
Total Precipitation (mm) [1−Month Lag]
C2:Nakhon Ratchasima
Malaria
1.6 1.4 1.2 1.0 0.8 0.6
0.0 0.2 0.4 0.6
95% CI
IRR
Mean (Exposure)
Total Precipitation (mm) [2−Month Lag]
D1:Nakhon Ratchasima
Malaria
20 15 10 5 0
20 22 24 26 28 30
95% CI
IRR
Mean (Exposure)
Temperature (°C) [1−Month Lag]
D2:Nakhon Ratchasima
Malaria
20 15 10 5 0
20 22 24 26 28 30
95% CI
IRR
Mean (Exposure)
Temperature (°C) [2−Month Lag]
E1:Nakhon Ratchasima
Malaria
1.4 1.2 1.0 0.8 0.6
2 3 4 5 6 7 8
95% CI
IRR
Mean (Exposure)
SO2 Surface Concentration (mg/m³) [1−Month Lag]
E2:Nakhon Ratchasima
Malaria
1.4 1.3 1.2 1.1 1.0 0.9 0.8 0.7
2 4 6 8
95% CI
IRR
Mean (Exposure)
SO2 Surface Concentration (mg/m³) [2−Month Lag]
F1:Nakhon Ratchasima
Malaria
1.8 1.6 1.4 1.2 1.0 0.8
150 200 250 300
95% CI
IRR
Mean (Exposure)
CO Surface Concentration (ppb) [1−Month Lag]
F2:Nakhon Ratchasima
Malaria
1.4 1.3 1.2 1.1 1.0 0.9 0.8
150 200 250 300
95% CI
IRR
Mean (Exposure)
CO Surface Concentration (ppb) [2−Month Lag]
G1:Nakhon Ratchasima
Malaria
1.2 1.1 1.0 0.9 0.8
10 15 20 25 30 35
95% CI
IRR
Mean (Exposure)
PM2.5 Surface Concentration (µg/m³) [1−Month Lag]
G2:Nakhon Ratchasima
Malaria
1.3 1.2 1.1 1.0 0.9 0.8 0.7
10 15 20 25 30 35
95% CI
IRR
Mean (Exposure)
PM2.5 Surface Concentration (µg/m³) [2−Month Lag]

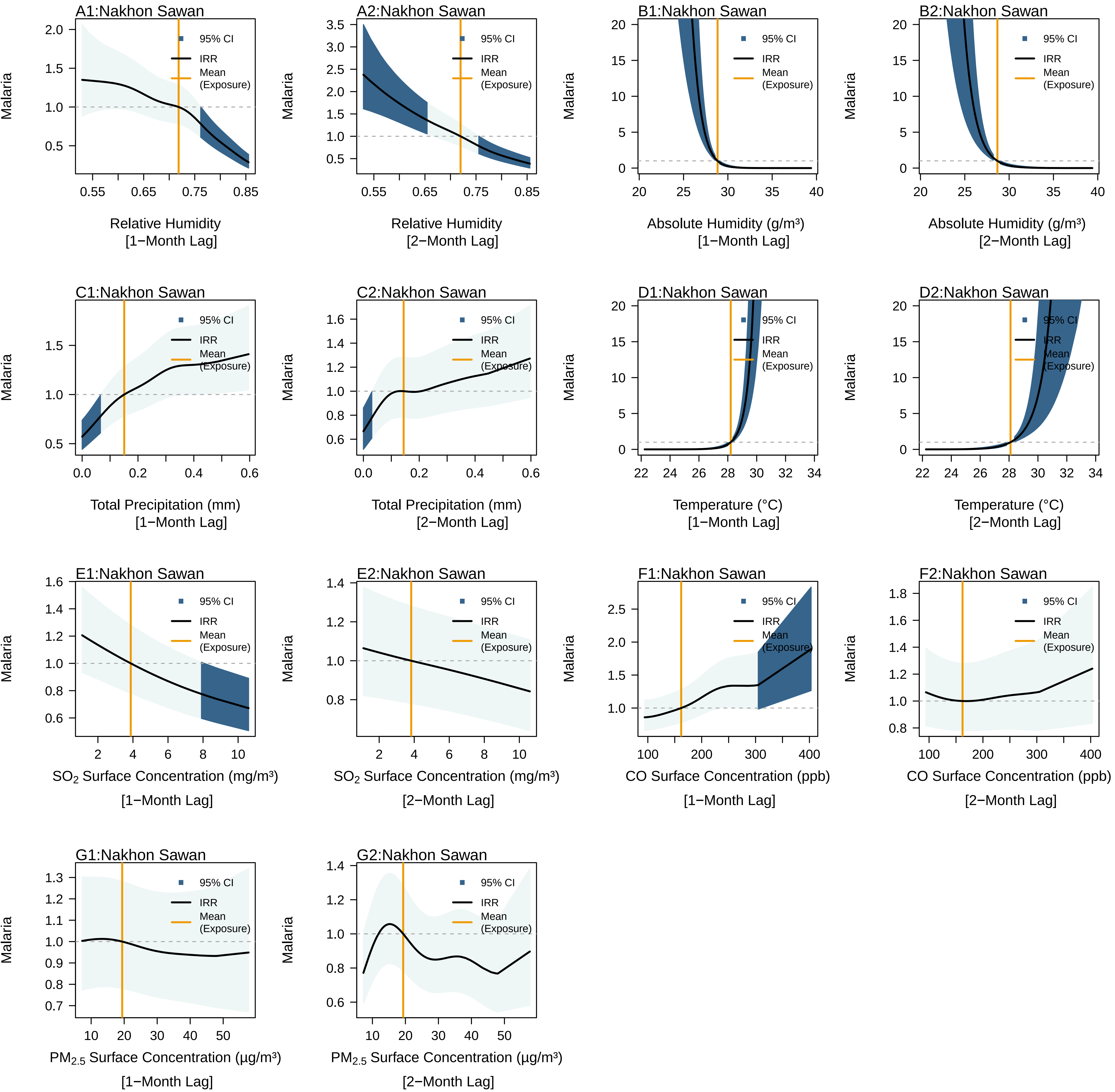

A1:Nakhon Sawan
Malaria
95% CI
IRR
Mean (Exposure)
Relative Humidity [1−Month Lag]
A2:Nakhon Sawan
Malaria
95% CI
IRR
Mean (Exposure)
Relative Humidity [2−Month Lag]
B1:Nakhon Sawan
Malaria
95% CI
IRR
Mean (Exposure)
Absolute Humidity (g/m³) [1−Month Lag]
B2:Nakhon Sawan
Malaria
95% CI
IRR
Mean (Exposure)
Absolute Humidity (g/m³) [2−Month Lag]
C1:Nakhon Sawan
Malaria
95% CI
IRR
Mean (Exposure)
Total Precipitation (mm) [1−Month Lag]
C2:Nakhon Sawan
Malaria
95% CI
IRR
Mean (Exposure)
Total Precipitation (mm) [2−Month Lag]
D1:Nakhon Sawan
Malaria
95% CI
IRR
Mean (Exposure)
Temperature (°C) [1−Month Lag]
D2:Nakhon Sawan
Malaria
95% CI
IRR
Mean (Exposure)
Temperature (°C) [2−Month Lag]
E1:Nakhon Sawan
Malaria
95% CI
IRR
Mean (Exposure)
SO_2 Surface Concentration (mg/m³) [1−Month Lag]
E2:Nakhon Sawan
Malaria
95% CI
IRR
Mean (Exposure)
SO_2 Surface Concentration (mg/m³) [2−Month Lag]
F1:Nakhon Sawan
Malaria
95% CI
IRR
Mean (Exposure)
CO Surface Concentration (ppb) [1−Month Lag]
F2:Nakhon Sawan
Malaria
95% CI
IRR
Mean (Exposure)
CO Surface Concentration (ppb) [2−Month Lag]
G1:Nakhon Sawan
Malaria
95% CI
IRR
Mean (Exposure)
$PM_{2.5}$ Surface Concentration (μg/m³) [1−Month Lag]
G2:Nakhon Sawan
Malaria
95% CI
IRR
Mean (Exposure)
$PM_{2.5}$ Surface Concentration (μg/m³) [2−Month Lag]

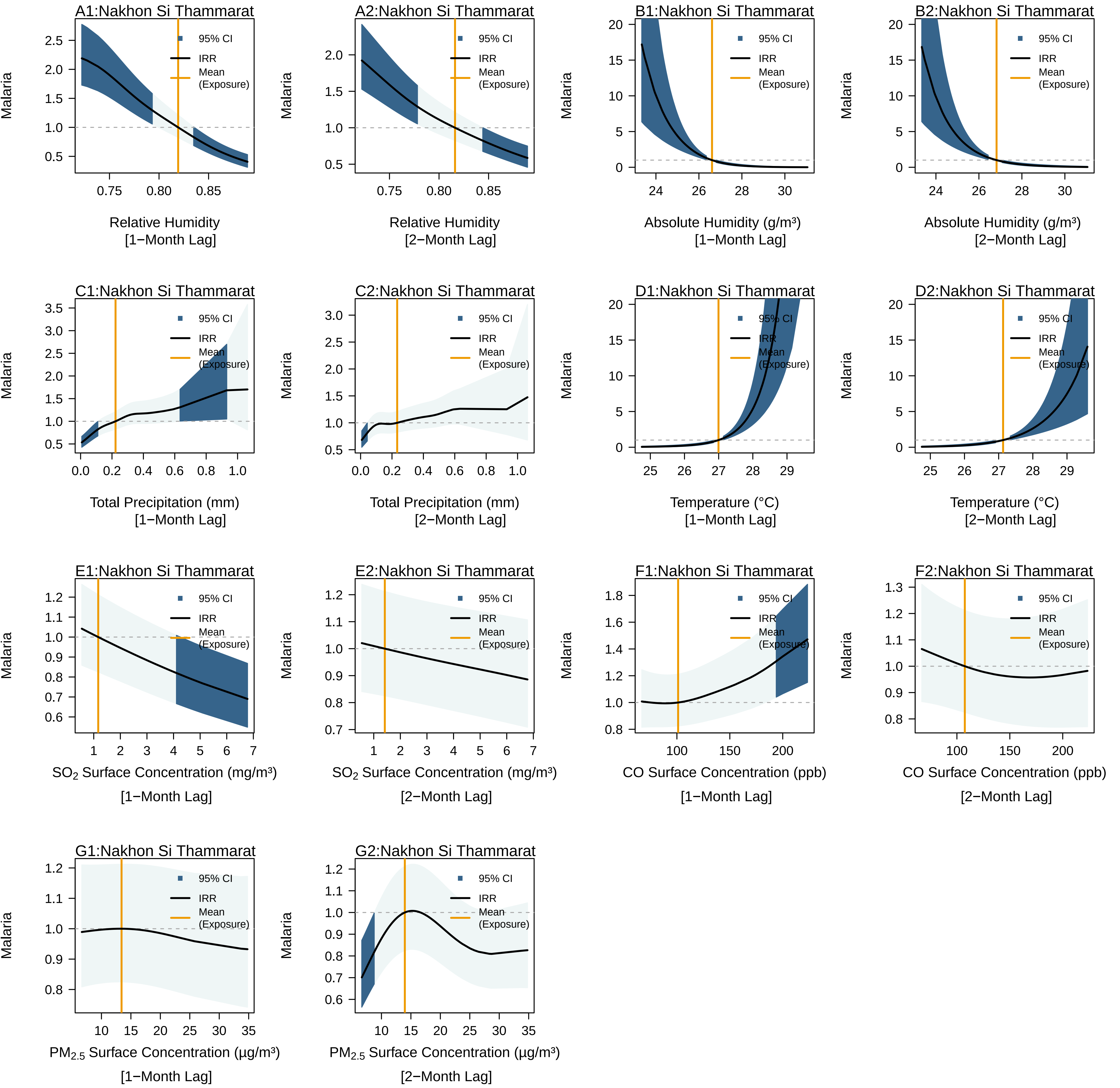
A1:Nakhon Si Thammarat
Malaria
Relative Humidity
[1−Month Lag]
A2:Nakhon Si Thammarat
Relative Humidity
[2−Month Lag]
B1:Nakhon Si Thammarat
Absolute Humidity (g/m³)
[1−Month Lag]
B2:Nakhon Si Thammarat
Absolute Humidity (g/m³)
[2−Month Lag]
C1:Nakhon Si Thammarat
Total Precipitation (mm)
[1−Month Lag]
C2:Nakhon Si Thammarat
Total Precipitation (mm)
[2−Month Lag]
D1:Nakhon Si Thammarat
Temperature (°C)
[1−Month Lag]
D2:Nakhon Si Thammarat
Temperature (°C)
[2−Month Lag]
E1:Nakhon Si Thammarat
SO_2 Surface Concentration (mg/m³)
[1−Month Lag]
E2:Nakhon Si Thammarat
SO_2 Surface Concentration (mg/m³)
[2−Month Lag]
F1:Nakhon Si Thammarat
CO Surface Concentration (ppb)
[1−Month Lag]
F2:Nakhon Si Thammarat
CO Surface Concentration (ppb)
[2−Month Lag]
G1:Nakhon Si Thammarat
$PM_{2.5}$ Surface Concentration (µg/m³)
[1−Month Lag]
G2:Nakhon Si Thammarat
$PM_{2.5}$ Surface Concentration (µg/m³)
[2−Month Lag]
95% CI
IRR
Mean
(Exposure)

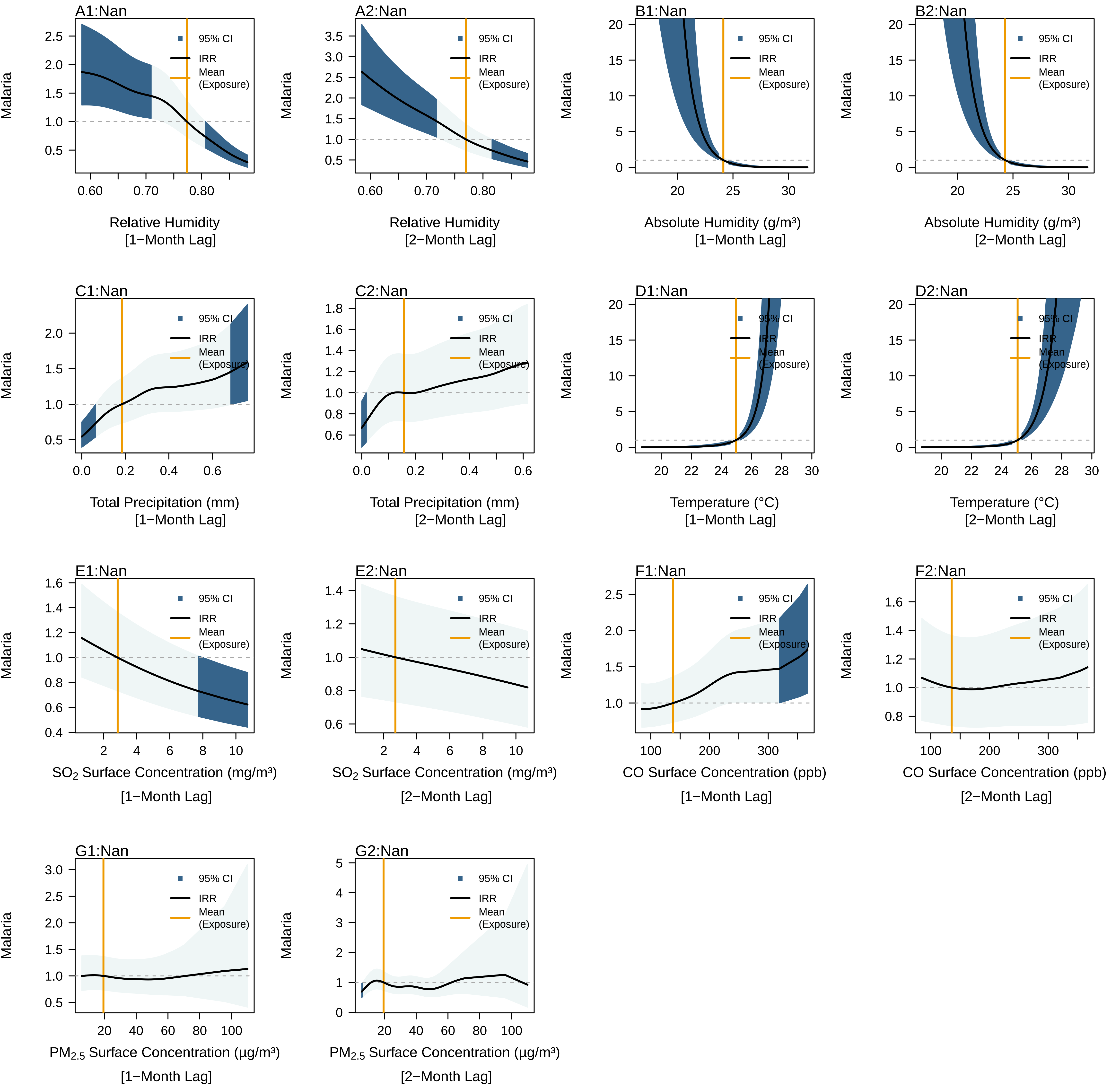
A1:Nan
A2:Nan
B1:Nan
B2:Nan
C1:Nan
C2:Nan
D1:Nan
D2:Nan
E1:Nan
E2:Nan
F1:Nan
F2:Nan
G1:Nan
G2:Nan
Malaria
95% CI
IRR
Mean (Exposure)
Relative Humidity [1−Month Lag]
Relative Humidity [2−Month Lag]
Absolute Humidity (g/m³) [1−Month Lag]
Absolute Humidity (g/m³) [2−Month Lag]
Total Precipitation (mm) [1−Month Lag]
Total Precipitation (mm) [2−Month Lag]
Temperature (°C) [1−Month Lag]
Temperature (°C) [2−Month Lag]
SO_2 Surface Concentration (mg/m³) [1−Month Lag]
SO_2 Surface Concentration (mg/m³) [2−Month Lag]
CO Surface Concentration (ppb) [1−Month Lag]
CO Surface Concentration (ppb) [2−Month Lag]
$PM_{2.5}$ Surface Concentration (µg/m³) [1−Month Lag]
$PM_{2.5}$ Surface Concentration (µg/m³) [2−Month Lag]

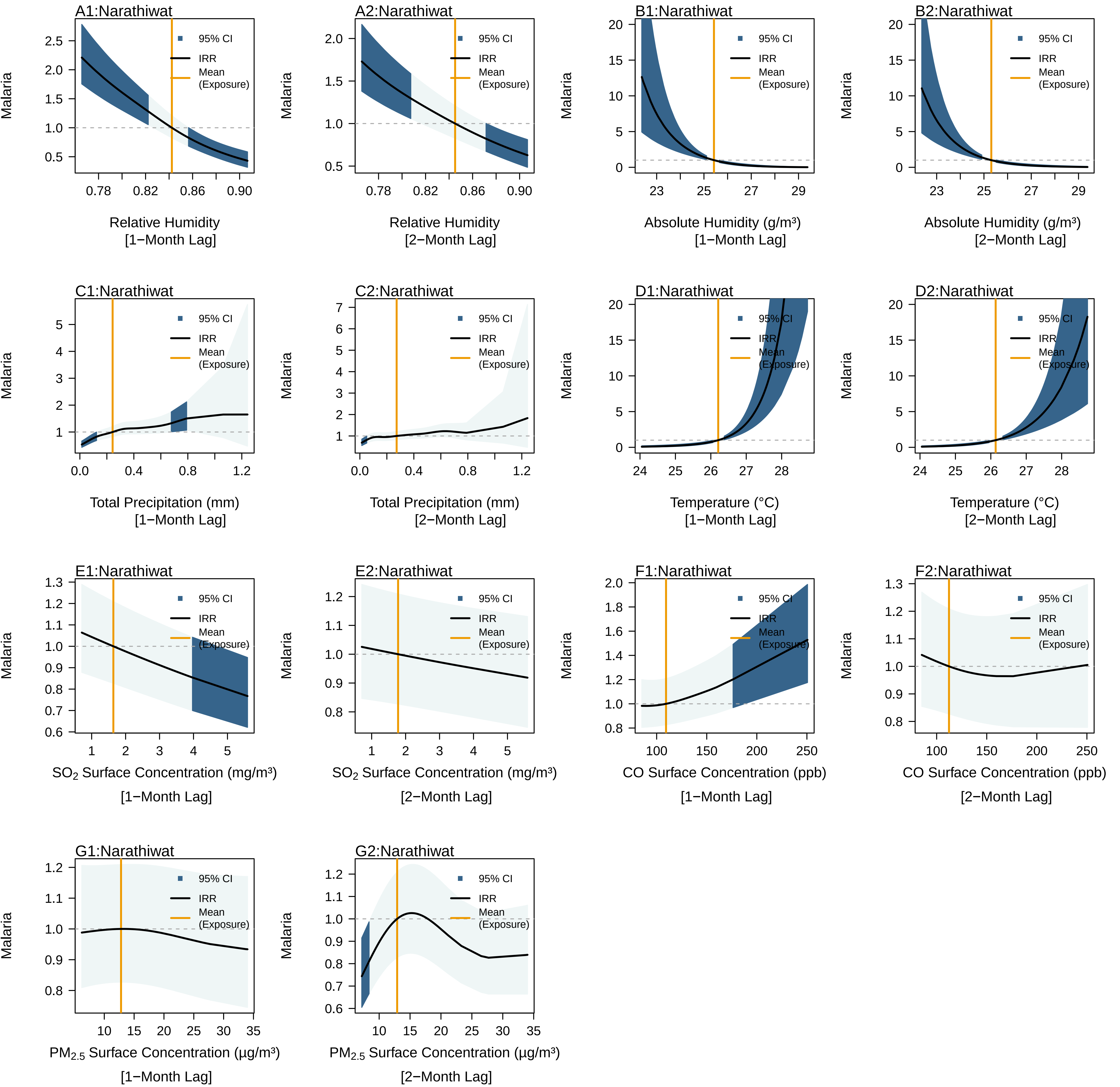
A1:Narathiwat
A2:Narathiwat
B1:Narathiwat
B2:Narathiwat
C1:Narathiwat
C2:Narathiwat
D1:Narathiwat
D2:Narathiwat
E1:Narathiwat
E2:Narathiwat
F1:Narathiwat
F2:Narathiwat
G1:Narathiwat
G2:Narathiwat
Malaria
95% CI
IRR
Mean (Exposure)
Relative Humidity [1−Month Lag]
Relative Humidity [2−Month Lag]
Absolute Humidity (g/m³) [1−Month Lag]
Absolute Humidity (g/m³) [2−Month Lag]
Total Precipitation (mm) [1−Month Lag]
Total Precipitation (mm) [2−Month Lag]
Temperature (°C) [1−Month Lag]
Temperature (°C) [2−Month Lag]
SO_2 Surface Concentration (mg/m³) [1−Month Lag]
SO_2 Surface Concentration (mg/m³) [2−Month Lag]
CO Surface Concentration (ppb) [1−Month Lag]
CO Surface Concentration (ppb) [2−Month Lag]
$PM_{2.5}$ Surface Concentration (µg/m³) [1−Month Lag]
$PM_{2.5}$ Surface Concentration (µg/m³) [2−Month Lag]

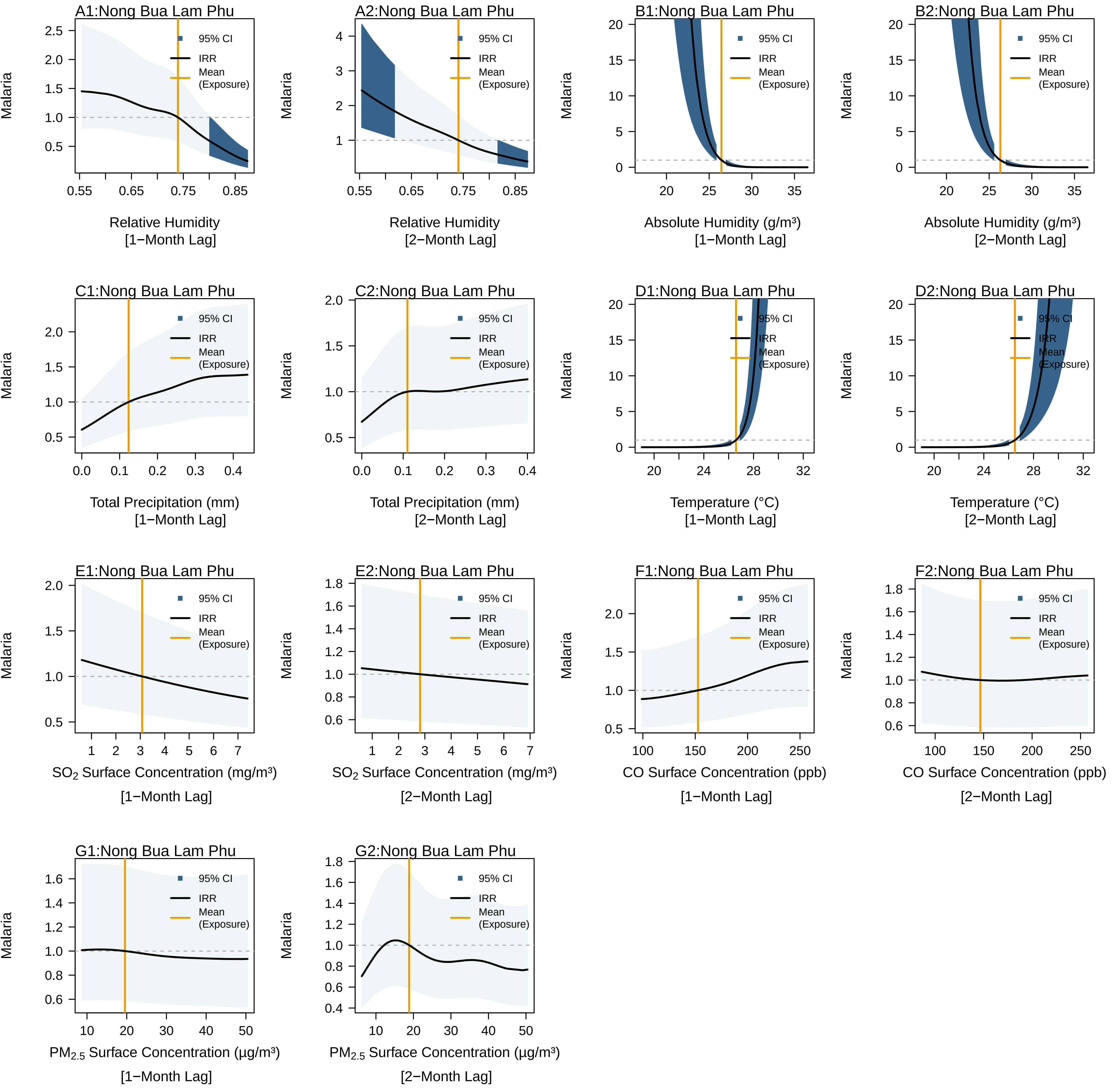

A1:Nong Bua Lam Phu
A2:Nong Bua Lam Phu
B1:Nong Bua Lam Phu
B2:Nong Bua Lam Phu
C1:Nong Bua Lam Phu
C2:Nong Bua Lam Phu
D1:Nong Bua Lam Phu
D2:Nong Bua Lam Phu
E1:Nong Bua Lam Phu
E2:Nong Bua Lam Phu
F1:Nong Bua Lam Phu
F2:Nong Bua Lam Phu
G1:Nong Bua Lam Phu
G2:Nong Bua Lam Phu
Malaria
95% CI
IRR
Mean (Exposure)
Relative Humidity [1−Month Lag]
Relative Humidity [2−Month Lag]
Absolute Humidity (g/m³) [1−Month Lag]
Absolute Humidity (g/m³) [2−Month Lag]
Total Precipitation (mm) [1−Month Lag]
Total Precipitation (mm) [2−Month Lag]
Temperature (°C) [1−Month Lag]
Temperature (°C) [2−Month Lag]
SO2 Surface Concentration (mg/m³) [1−Month Lag]
SO2 Surface Concentration (mg/m³) [2−Month Lag]
CO Surface Concentration (ppb) [1−Month Lag]
CO Surface Concentration (ppb) [2−Month Lag]
PM2.5 Surface Concentration (µg/m³) [1−Month Lag]
PM2.5 Surface Concentration (µg/m³) [2−Month Lag]

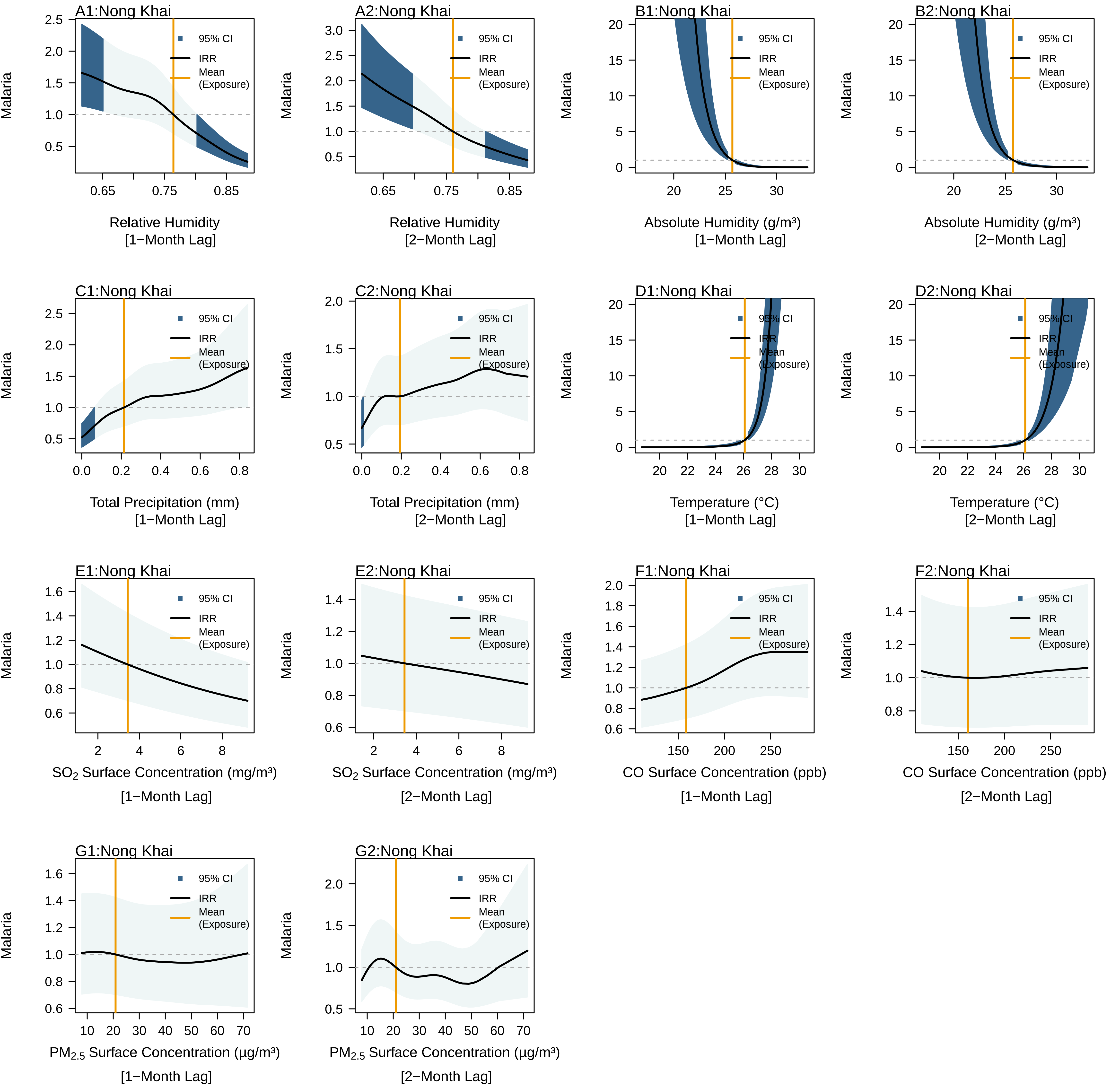
A1:Nong Khai
Malaria
95% CI
IRR
Mean (Exposure)
Relative Humidity [1−Month Lag]
A2:Nong Khai
Relative Humidity [2−Month Lag]
B1:Nong Khai
Absolute Humidity (g/m³) [1−Month Lag]
B2:Nong Khai
Absolute Humidity (g/m³) [2−Month Lag]
C1:Nong Khai
Total Precipitation (mm) [1−Month Lag]
C2:Nong Khai
Total Precipitation (mm) [2−Month Lag]
D1:Nong Khai
Temperature (°C) [1−Month Lag]
D2:Nong Khai
Temperature (°C) [2−Month Lag]
E1:Nong Khai
SO_2 Surface Concentration (mg/m³) [1−Month Lag]
E2:Nong Khai
SO_2 Surface Concentration (mg/m³) [2−Month Lag]
F1:Nong Khai
CO Surface Concentration (ppb) [1−Month Lag]
F2:Nong Khai
CO Surface Concentration (ppb) [2−Month Lag]
G1:Nong Khai
$PM_{2.5}$ Surface Concentration (µg/m³) [1−Month Lag]
G2:Nong Khai
$PM_{2.5}$ Surface Concentration (µg/m³) [2−Month Lag]

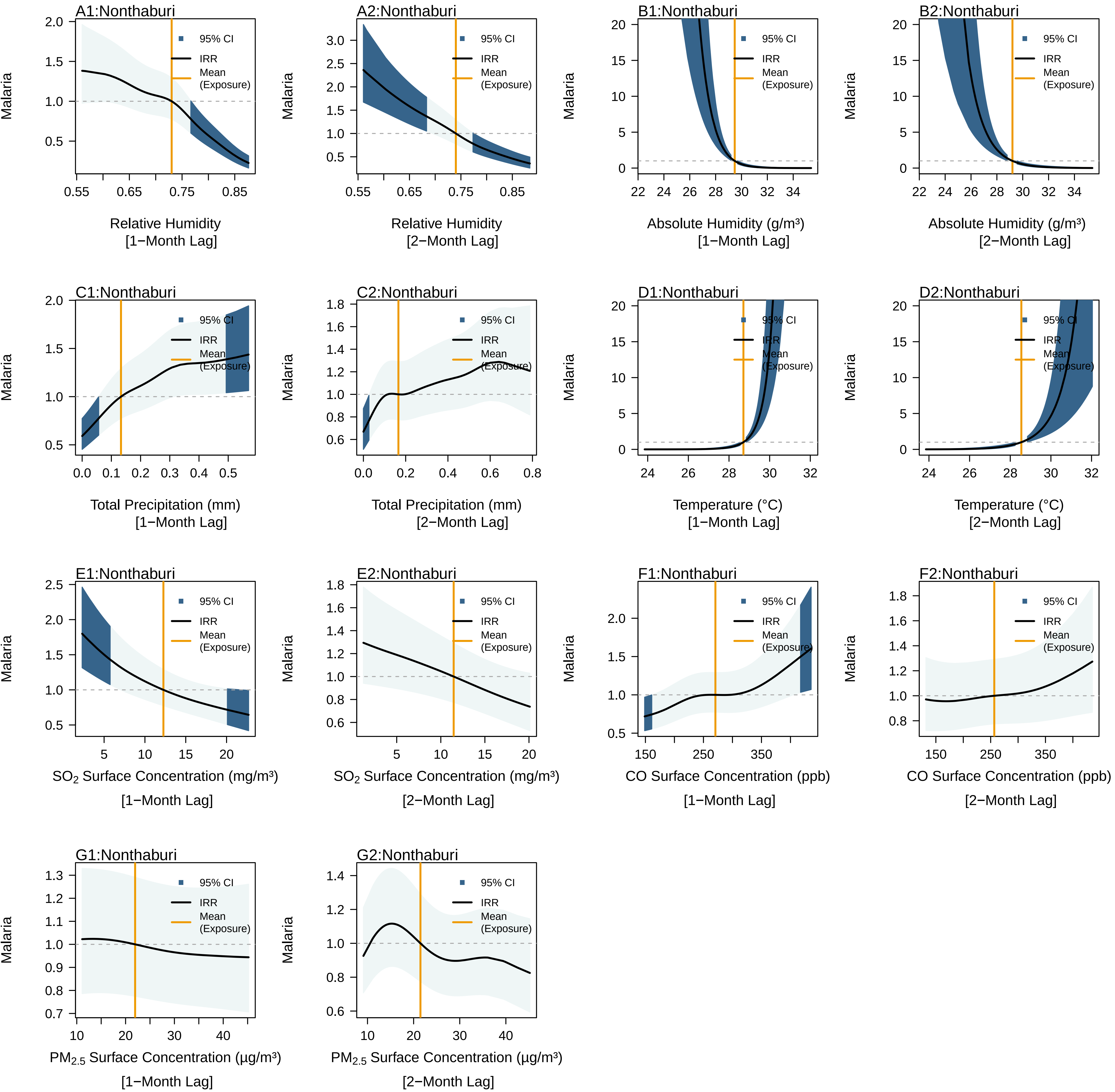
A1:Nonthaburi
Malaria
95% CI
IRR
Mean (Exposure)
Relative Humidity [1−Month Lag]
A2:Nonthaburi
Malaria
95% CI
IRR
Mean (Exposure)
Relative Humidity [2−Month Lag]
B1:Nonthaburi
Malaria
95% CI
IRR
Mean (Exposure)
Absolute Humidity (g/m³) [1−Month Lag]
B2:Nonthaburi
Malaria
95% CI
IRR
Mean (Exposure)
Absolute Humidity (g/m³) [2−Month Lag]
C1:Nonthaburi
Malaria
95% CI
IRR
Mean (Exposure)
Total Precipitation (mm) [1−Month Lag]
C2:Nonthaburi
Malaria
95% CI
IRR
Mean (Exposure)
Total Precipitation (mm) [2−Month Lag]
D1:Nonthaburi
Malaria
95% CI
IRR
Mean (Exposure)
Temperature (°C) [1−Month Lag]
D2:Nonthaburi
Malaria
95% CI
IRR
Mean (Exposure)
Temperature (°C) [2−Month Lag]
E1:Nonthaburi
Malaria
95% CI
IRR
Mean (Exposure)
SO2 Surface Concentration (mg/m³) [1−Month Lag]
E2:Nonthaburi
Malaria
95% CI
IRR
Mean (Exposure)
SO2 Surface Concentration (mg/m³) [2−Month Lag]
F1:Nonthaburi
Malaria
95% CI
IRR
Mean (Exposure)
CO Surface Concentration (ppb) [1−Month Lag]
F2:Nonthaburi
Malaria
95% CI
IRR
Mean (Exposure)
CO Surface Concentration (ppb) [2−Month Lag]
G1:Nonthaburi
Malaria
95% CI
IRR
Mean (Exposure)
PM2.5 Surface Concentration (µg/m³) [1−Month Lag]
G2:Nonthaburi
Malaria
95% CI
IRR
Mean (Exposure)
PM2.5 Surface Concentration (µg/m³) [2−Month Lag]

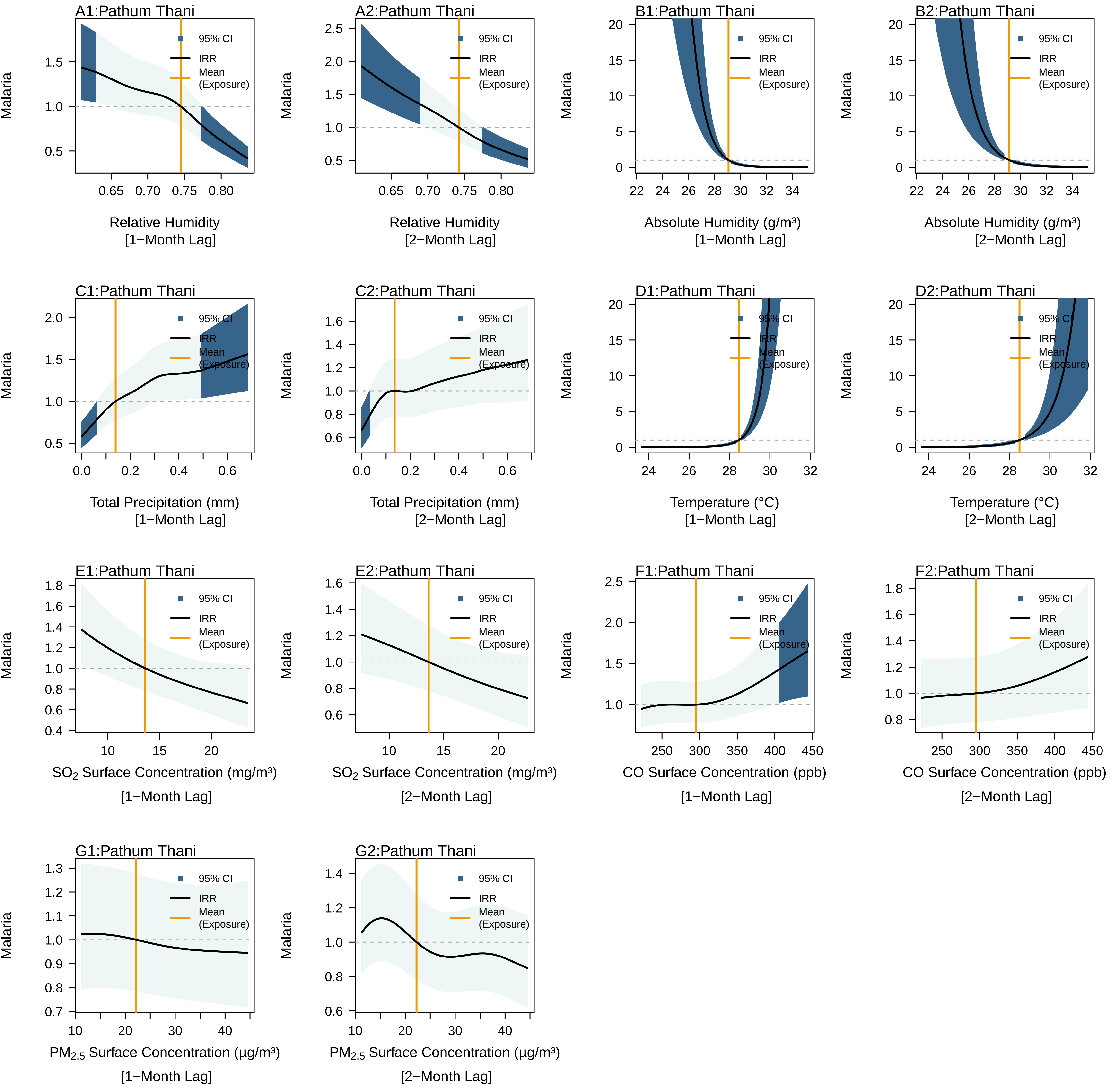

A1:Pathum Thani
A2:Pathum Thani
B1:Pathum Thani
B2:Pathum Thani
C1:Pathum Thani
C2:Pathum Thani
D1:Pathum Thani
D2:Pathum Thani
E1:Pathum Thani
E2:Pathum Thani
F1:Pathum Thani
F2:Pathum Thani
G1:Pathum Thani
G2:Pathum Thani
Malaria
95% CI
IRR
Mean
(Exposure)
Relative Humidity
[1−Month Lag]
[2−Month Lag]
Absolute Humidity (g/m³)
Total Precipitation (mm)
Temperature (°C)
SO2 Surface Concentration (mg/m³)
CO Surface Concentration (ppb)
PM2.5 Surface Concentration (µg/m³)

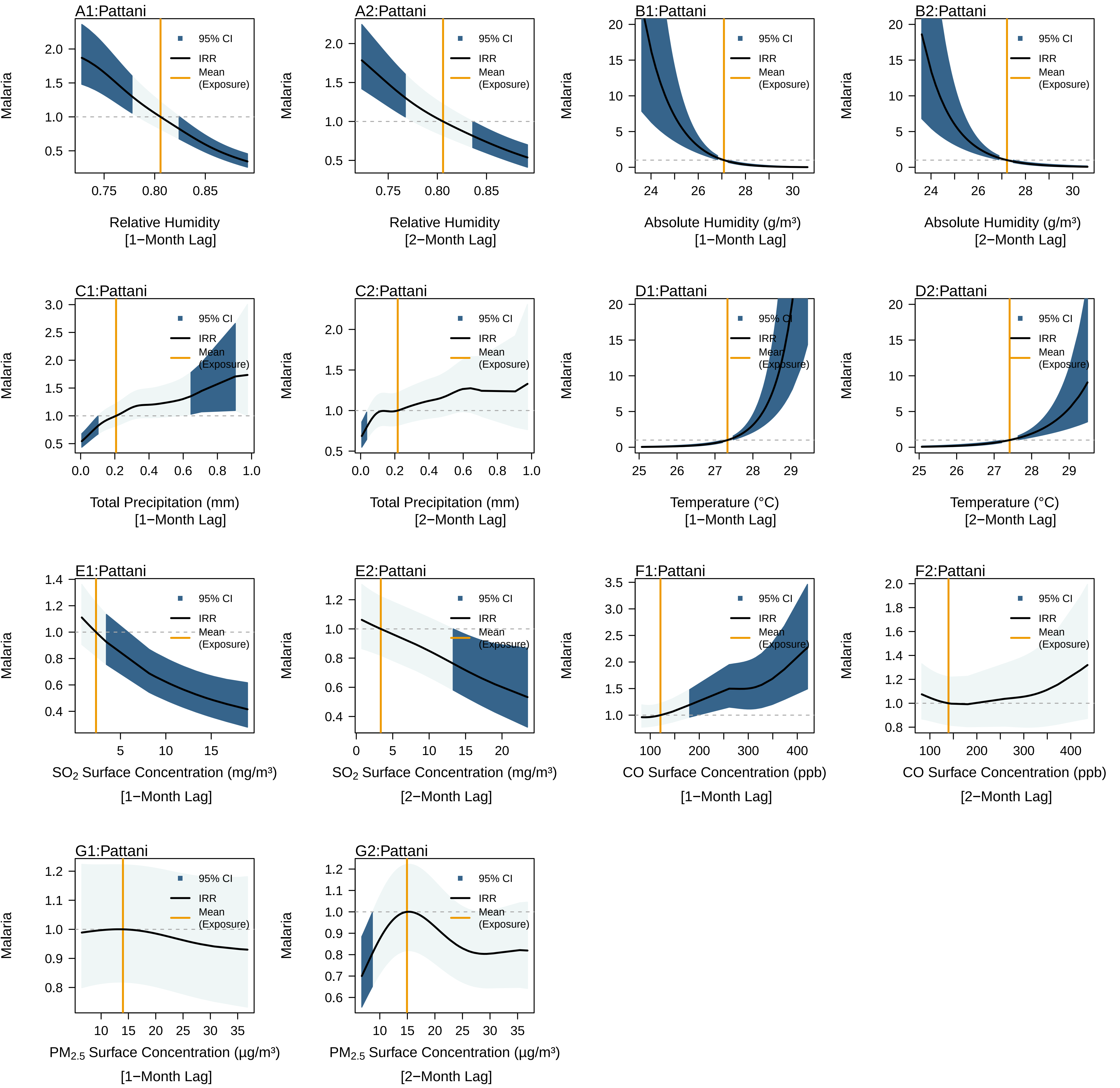
A1:Pattani
Malaria
95% CI
IRR
Mean
(Exposure)
Relative Humidity
[1−Month Lag]
A2:Pattani
Relative Humidity
[2−Month Lag]
B1:Pattani
Absolute Humidity (g/m³)
[1−Month Lag]
B2:Pattani
Absolute Humidity (g/m³)
[2−Month Lag]
C1:Pattani
Total Precipitation (mm)
[1−Month Lag]
C2:Pattani
Total Precipitation (mm)
[2−Month Lag]
D1:Pattani
Temperature (°C)
[1−Month Lag]
D2:Pattani
Temperature (°C)
[2−Month Lag]
E1:Pattani
SO_2 Surface Concentration (mg/m³)
[1−Month Lag]
E2:Pattani
SO_2 Surface Concentration (mg/m³)
[2−Month Lag]
F1:Pattani
CO Surface Concentration (ppb)
[1−Month Lag]
F2:Pattani
CO Surface Concentration (ppb)
[2−Month Lag]
G1:Pattani
$PM_{2.5}$ Surface Concentration (μg/m³)
[1−Month Lag]
G2:Pattani
$PM_{2.5}$ Surface Concentration (μg/m³)
[2−Month Lag]

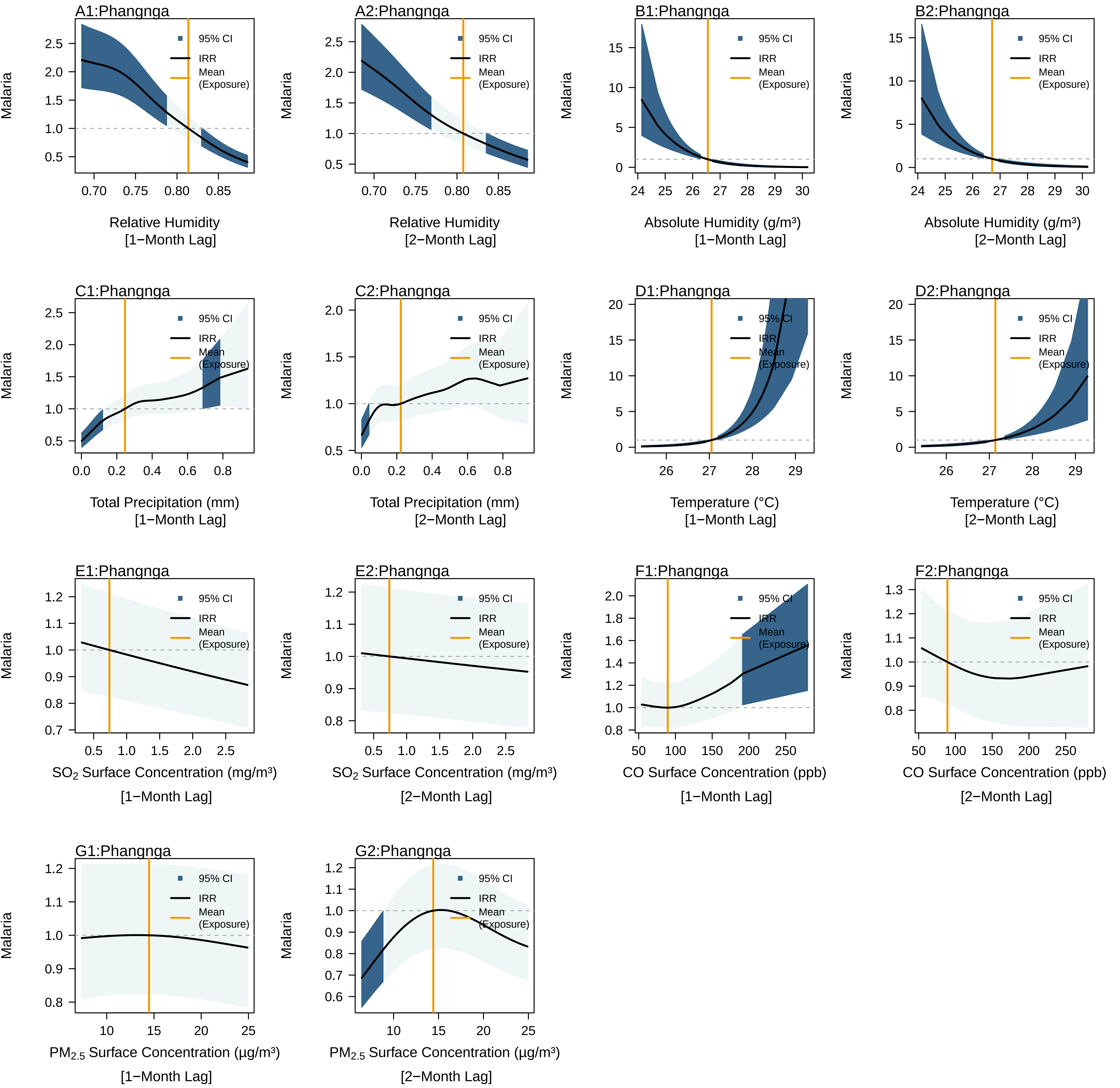
A1:Phangnga
Malaria
2.5
2.0
1.5
1.0
0.5
95% CI
IRR
Mean
(Exposure)
0.70
0.75
0.80
0.85
Relative Humidity
[1−Month Lag]
A2:Phangnga
Malaria
2.5
2.0
1.5
1.0
0.5
95% CI
IRR
Mean
(Exposure)
0.70
0.75
0.80
0.85
Relative Humidity
[2−Month Lag]
B1:Phangnga
Malaria
15
10
5
0
95% CI
IRR
Mean
(Exposure)
24
25
26
27
28
29
30
Absolute Humidity (g/m³)
[1−Month Lag]
B2:Phangnga
Malaria
15
10
5
0
95% CI
IRR
Mean
(Exposure)
24
25
26
27
28
29
30
Absolute Humidity (g/m³)
[2−Month Lag]
C1:Phangnga
Malaria
2.5
2.0
1.5
1.0
0.5
95% CI
IRR
Mean
(Exposure)
0.0
0.2
0.4
0.6
0.8
Total Precipitation (mm)
[1−Month Lag]
C2:Phangnga
Malaria
2.0
1.5
1.0
0.5
95% CI
IRR
Mean
(Exposure)
0.0
0.2
0.4
0.6
0.8
Total Precipitation (mm)
[2−Month Lag]
D1:Phangnga
Malaria
20
15
10
5
0
95% CI
IRR
Mean
(Exposure)
26
27
28
29
Temperature (°C)
[1−Month Lag]
D2:Phangnga
Malaria
20
15
10
5
0
95% CI
IRR
Mean
(Exposure)
26
27
28
29
Temperature (°C)
[2−Month Lag]
E1:Phangnga
Malaria
1.2
1.1
1.0
0.9
0.8
0.7
95% CI
IRR
Mean
(Exposure)
0.5
1.0
1.5
2.0
2.5
SO2 Surface Concentration (mg/m³)
[1−Month Lag]
E2:Phangnga
Malaria
1.2
1.1
1.0
0.9
0.8
95% CI
IRR
Mean
(Exposure)
0.5
1.0
1.5
2.0
2.5
SO2 Surface Concentration (mg/m³)
[2−Month Lag]
F1:Phangnga
Malaria
2.0
1.8
1.6
1.4
1.2
1.0
0.8
95% CI
IRR
Mean
(Exposure)
50
100
150
200
250
CO Surface Concentration (ppb)
[1−Month Lag]
F2:Phangnga
Malaria
1.3
1.2
1.1
1.0
0.9
0.8
95% CI
IRR
Mean
(Exposure)
50
100
150
200
250
CO Surface Concentration (ppb)
[2−Month Lag]
G1:Phangnga
Malaria
1.2
1.1
1.0
0.9
0.8
95% CI
IRR
Mean
(Exposure)
10
15
20
25
PM2.5 Surface Concentration (µg/m³)
[1−Month Lag]
G2:Phangnga
Malaria
1.2
1.1
1.0
0.9
0.8
0.7
0.6
95% CI
IRR
Mean
(Exposure)
10
15
20
25
PM2.5 Surface Concentration (µg/m³)
[2−Month Lag]

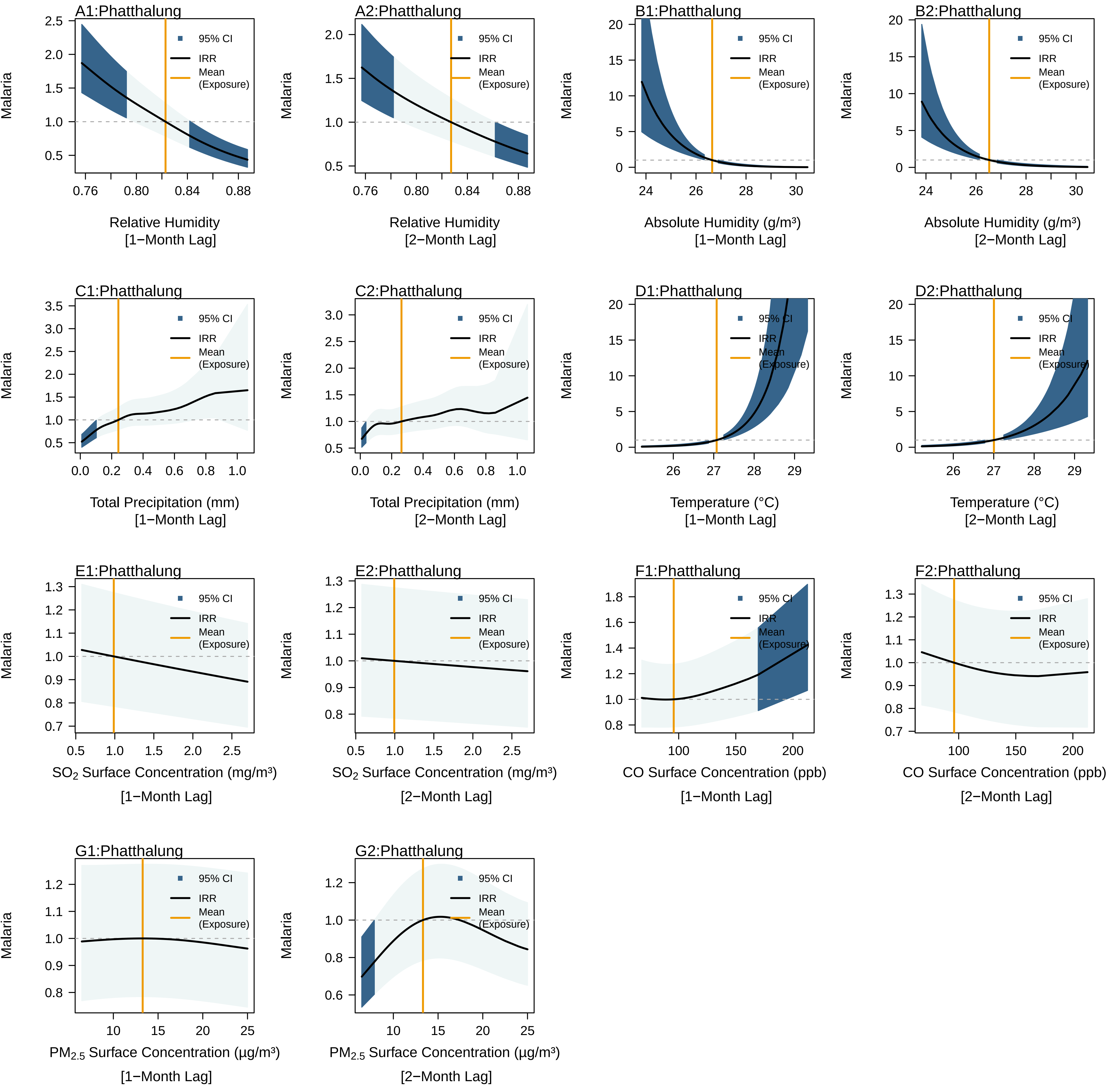
A1:Phatthalung
A2:Phatthalung
B1:Phatthalung
B2:Phatthalung
C1:Phatthalung
C2:Phatthalung
D1:Phatthalung
D2:Phatthalung
E1:Phatthalung
E2:Phatthalung
F1:Phatthalung
F2:Phatthalung
G1:Phatthalung
G2:Phatthalung
Malaria
95% CI
IRR
Mean (Exposure)
Relative Humidity [1−Month Lag]
Relative Humidity [2−Month Lag]
Absolute Humidity (g/m³) [1−Month Lag]
Absolute Humidity (g/m³) [2−Month Lag]
Total Precipitation (mm) [1−Month Lag]
Total Precipitation (mm) [2−Month Lag]
Temperature (°C) [1−Month Lag]
Temperature (°C) [2−Month Lag]
SO_2 Surface Concentration (mg/m³) [1−Month Lag]
SO_2 Surface Concentration (mg/m³) [2−Month Lag]
CO Surface Concentration (ppb) [1−Month Lag]
CO Surface Concentration (ppb) [2−Month Lag]
$PM_{2.5}$ Surface Concentration (µg/m³) [1−Month Lag]
$PM_{2.5}$ Surface Concentration (µg/m³) [2−Month Lag]

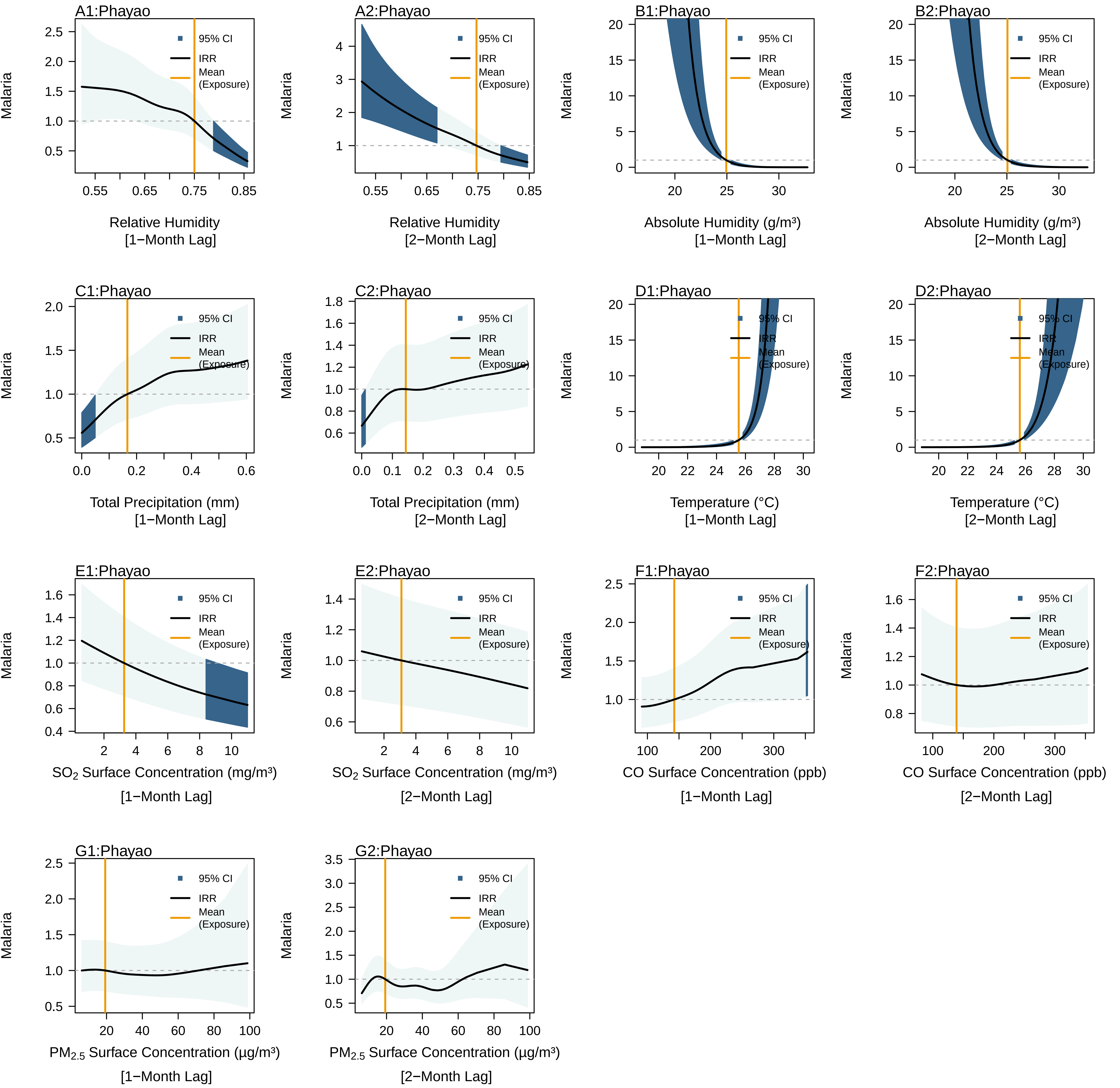
A1:Phayao
A2:Phayao
B1:Phayao
B2:Phayao
C1:Phayao
C2:Phayao
D1:Phayao
D2:Phayao
E1:Phayao
E2:Phayao
F1:Phayao
F2:Phayao
G1:Phayao
G2:Phayao
Malaria
95% CI
IRR
Mean (Exposure)
Relative Humidity [1−Month Lag]
Relative Humidity [2−Month Lag]
Absolute Humidity (g/m³) [1−Month Lag]
Absolute Humidity (g/m³) [2−Month Lag]
Total Precipitation (mm) [1−Month Lag]
Total Precipitation (mm) [2−Month Lag]
Temperature (°C) [1−Month Lag]
Temperature (°C) [2−Month Lag]
SO_2 Surface Concentration (mg/m³) [1−Month Lag]
SO_2 Surface Concentration (mg/m³) [2−Month Lag]
CO Surface Concentration (ppb) [1−Month Lag]
CO Surface Concentration (ppb) [2−Month Lag]
$PM_{2.5}$ Surface Concentration (μg/m³) [1−Month Lag]
$PM_{2.5}$ Surface Concentration (μg/m³) [2−Month Lag]

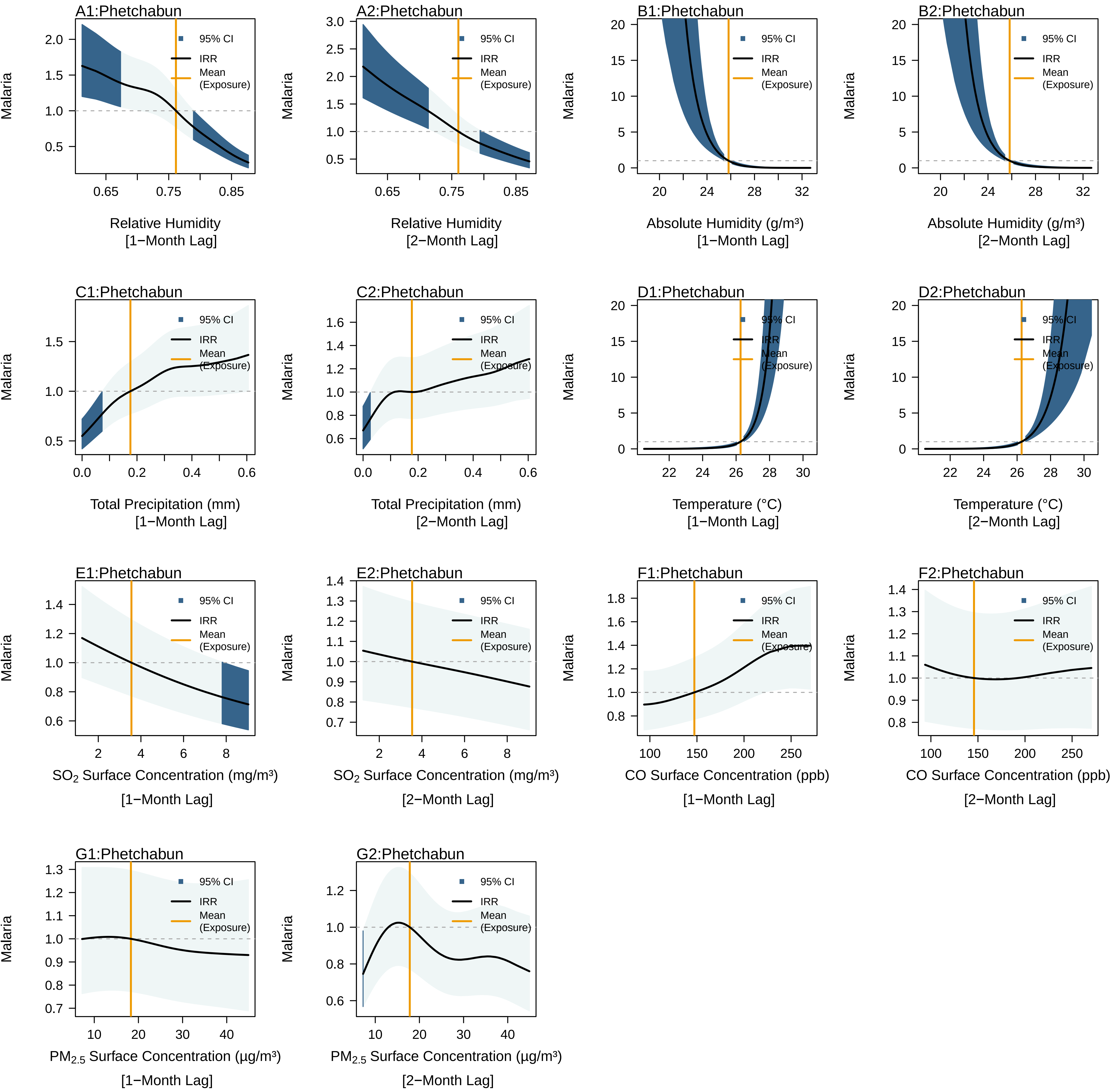

A1:Phetchabun
A2:Phetchabun
B1:Phetchabun
B2:Phetchabun
C1:Phetchabun
C2:Phetchabun
D1:Phetchabun
D2:Phetchabun
E1:Phetchabun
E2:Phetchabun
F1:Phetchabun
F2:Phetchabun
G1:Phetchabun
G2:Phetchabun
Malaria
95% CI
IRR
Mean (Exposure)
Relative Humidity [1−Month Lag]
Relative Humidity [2−Month Lag]
Absolute Humidity (g/m³) [1−Month Lag]
Absolute Humidity (g/m³) [2−Month Lag]
Total Precipitation (mm) [1−Month Lag]
Total Precipitation (mm) [2−Month Lag]
Temperature (°C) [1−Month Lag]
Temperature (°C) [2−Month Lag]
SO_2 Surface Concentration (mg/m³) [1−Month Lag]
SO_2 Surface Concentration (mg/m³) [2−Month Lag]
CO Surface Concentration (ppb) [1−Month Lag]
CO Surface Concentration (ppb) [2−Month Lag]
$PM_{2.5}$ Surface Concentration (µg/m³) [1−Month Lag]
$PM_{2.5}$ Surface Concentration (µg/m³) [2−Month Lag]

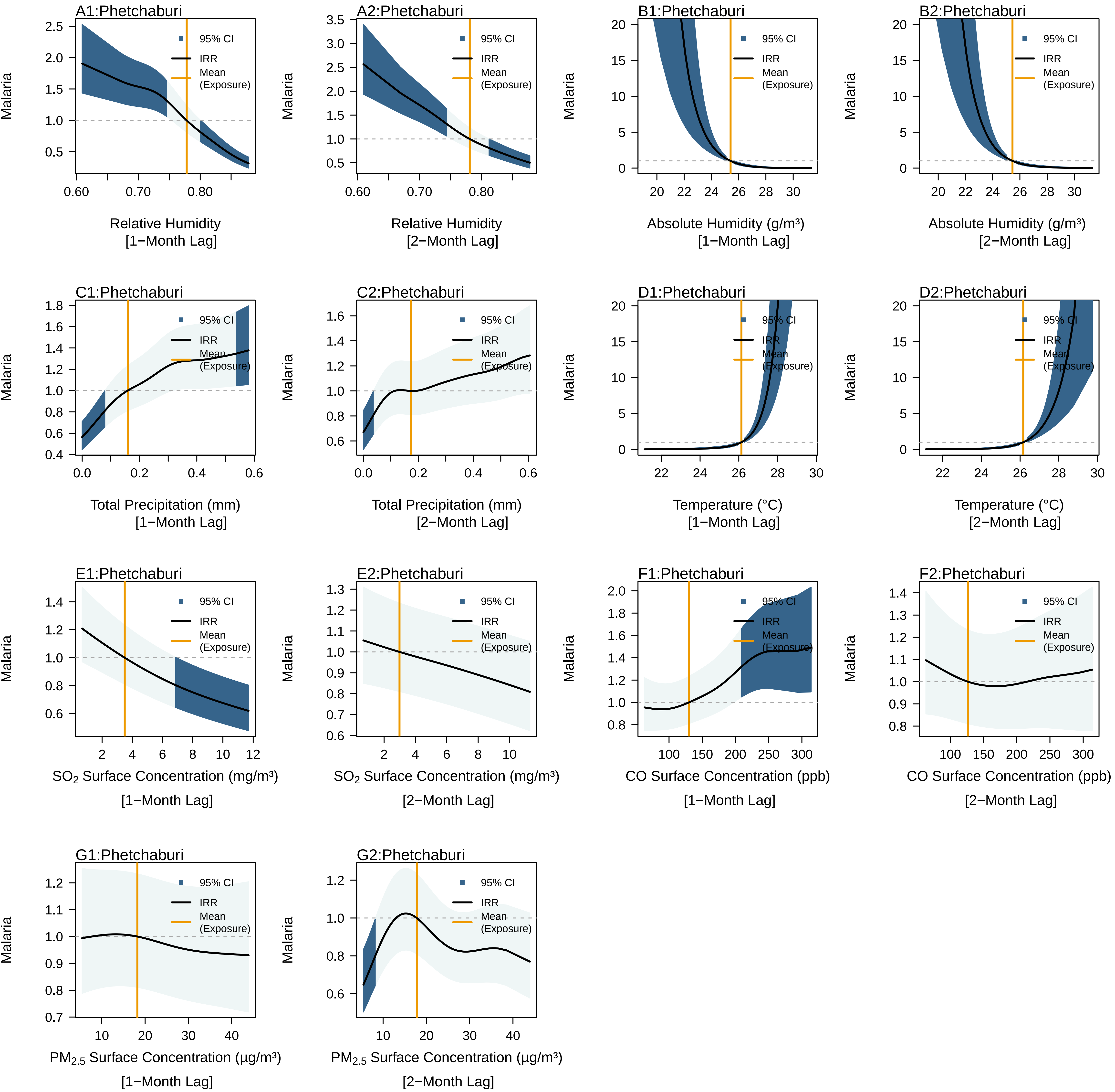
A1:Phetchaburi
Malaria
95% CI
IRR
Mean
(Exposure)
Relative Humidity
[1−Month Lag]
A2:Phetchaburi
Malaria
Relative Humidity
[2−Month Lag]
B1:Phetchaburi
Malaria
Absolute Humidity (g/m³)
[1−Month Lag]
B2:Phetchaburi
Malaria
Absolute Humidity (g/m³)
[2−Month Lag]
C1:Phetchaburi
Malaria
Total Precipitation (mm)
[1−Month Lag]
C2:Phetchaburi
Malaria
Total Precipitation (mm)
[2−Month Lag]
D1:Phetchaburi
Malaria
Temperature (°C)
[1−Month Lag]
D2:Phetchaburi
Malaria
Temperature (°C)
[2−Month Lag]
E1:Phetchaburi
Malaria
SO_2 Surface Concentration (mg/m³)
[1−Month Lag]
E2:Phetchaburi
Malaria
SO_2 Surface Concentration (mg/m³)
[2−Month Lag]
F1:Phetchaburi
Malaria
CO Surface Concentration (ppb)
[1−Month Lag]
F2:Phetchaburi
Malaria
CO Surface Concentration (ppb)
[2−Month Lag]
G1:Phetchaburi
Malaria
$PM_{2.5}$ Surface Concentration (µg/m³)
[1−Month Lag]
G2:Phetchaburi
Malaria
$PM_{2.5}$ Surface Concentration (µg/m³)
[2−Month Lag]

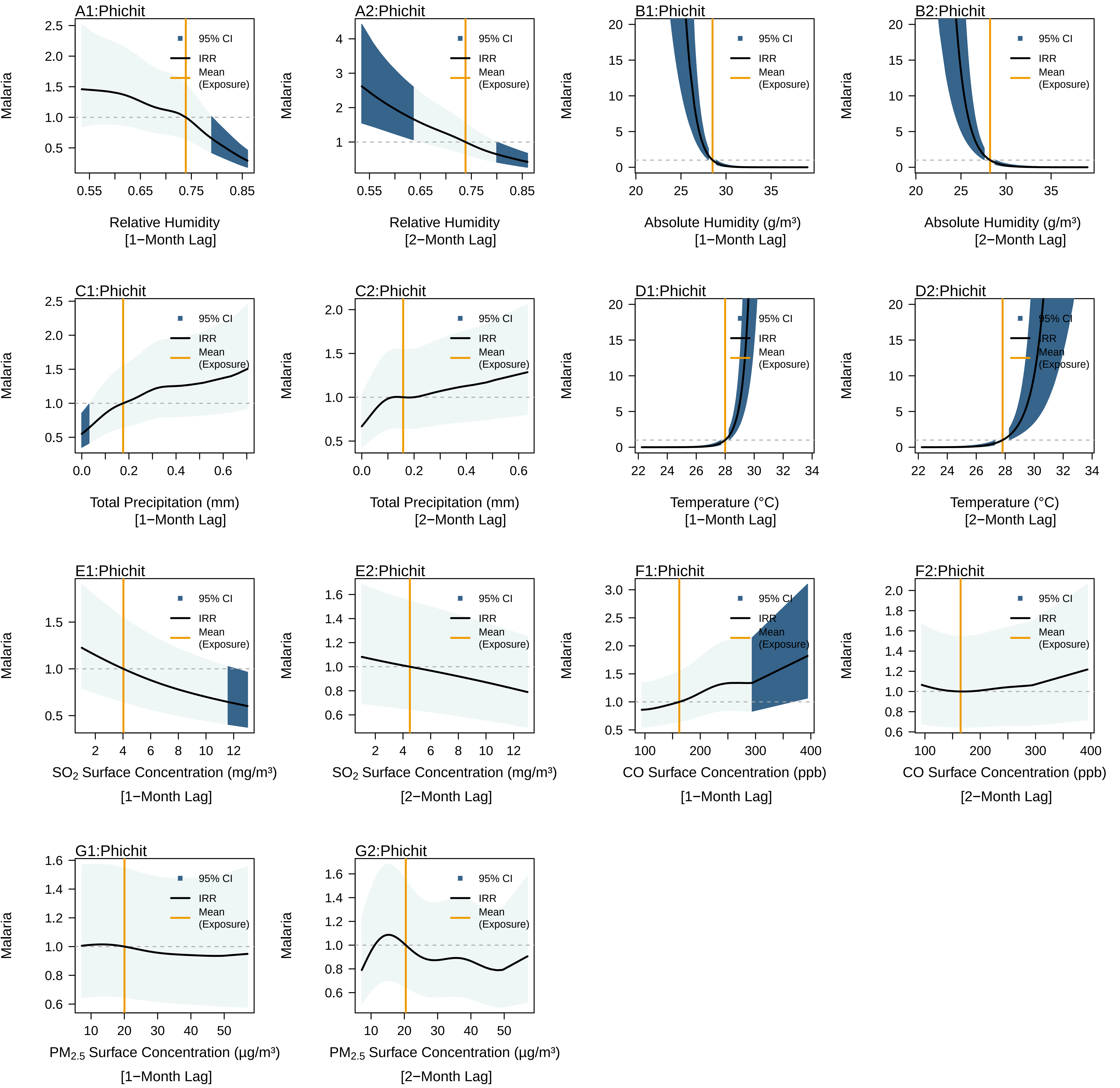
A1:Phichit
A2:Phichit
B1:Phichit
B2:Phichit
C1:Phichit
C2:Phichit
D1:Phichit
D2:Phichit
E1:Phichit
E2:Phichit
F1:Phichit
F2:Phichit
G1:Phichit
G2:Phichit
Malaria
95% CI
IRR
Mean
(Exposure)
Relative Humidity
[1−Month Lag]
[2−Month Lag]
Absolute Humidity (g/m³)
Total Precipitation (mm)
Temperature (°C)
SO_2 Surface Concentration (mg/m³)
CO Surface Concentration (ppb)
$PM_{2.5}$ Surface Concentration (µg/m³)

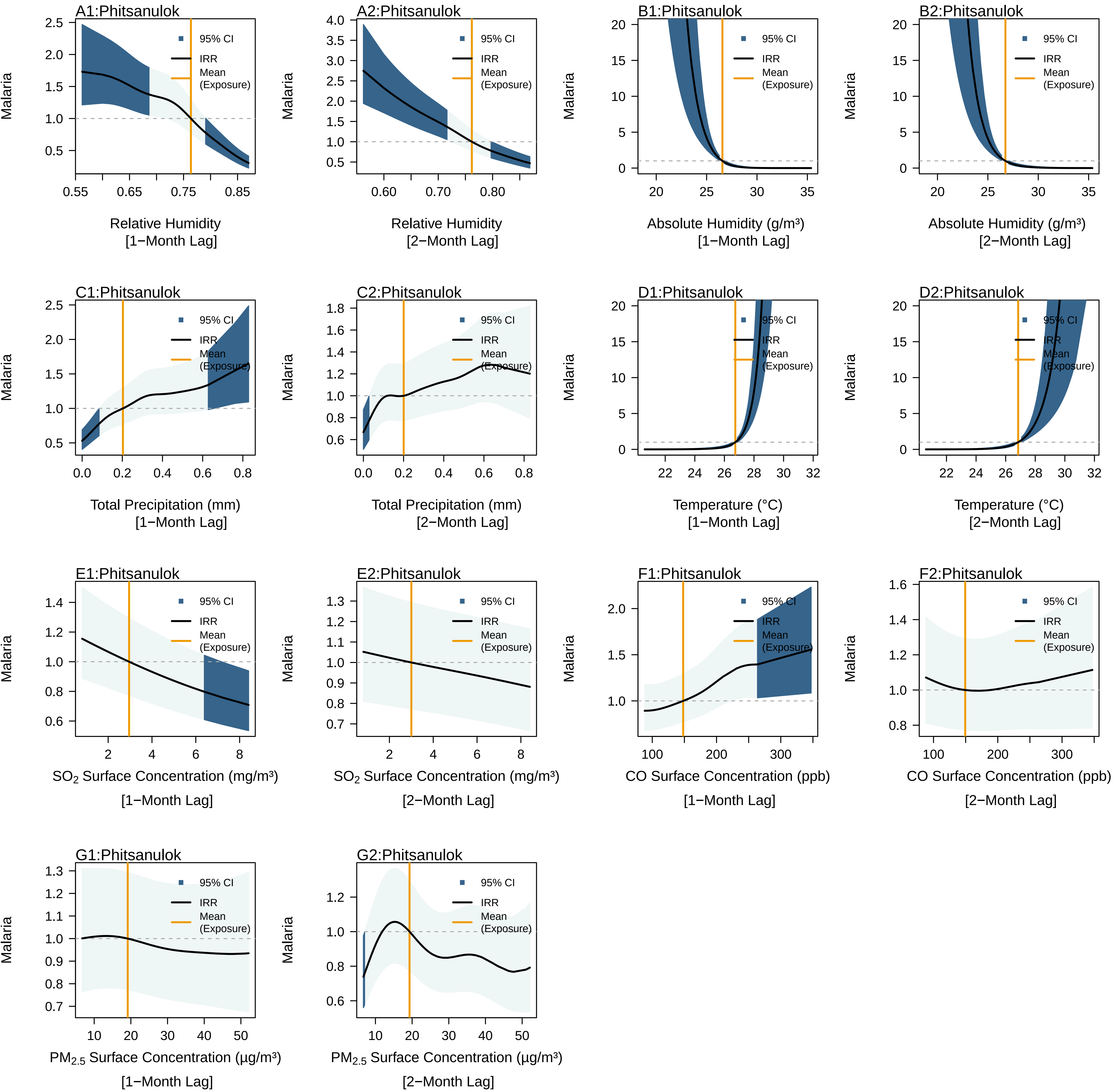
A1:Phitsanulok
Malaria
95% CI
IRR
Mean
(Exposure)
Relative Humidity
[1−Month Lag]
A2:Phitsanulok
Relative Humidity
[2−Month Lag]
B1:Phitsanulok
Absolute Humidity (g/m³)
[1−Month Lag]
B2:Phitsanulok
Absolute Humidity (g/m³)
[2−Month Lag]
C1:Phitsanulok
Total Precipitation (mm)
[1−Month Lag]
C2:Phitsanulok
Total Precipitation (mm)
[2−Month Lag]
D1:Phitsanulok
Temperature (°C)
[1−Month Lag]
D2:Phitsanulok
Temperature (°C)
[2−Month Lag]
E1:Phitsanulok
SO_2 Surface Concentration (mg/m³)
[1−Month Lag]
E2:Phitsanulok
SO_2 Surface Concentration (mg/m³)
[2−Month Lag]
F1:Phitsanulok
CO Surface Concentration (ppb)
[1−Month Lag]
F2:Phitsanulok
CO Surface Concentration (ppb)
[2−Month Lag]
G1:Phitsanulok
$PM_{2.5}$ Surface Concentration (µg/m³)
[1−Month Lag]
G2:Phitsanulok
$PM_{2.5}$ Surface Concentration (µg/m³)
[2−Month Lag]

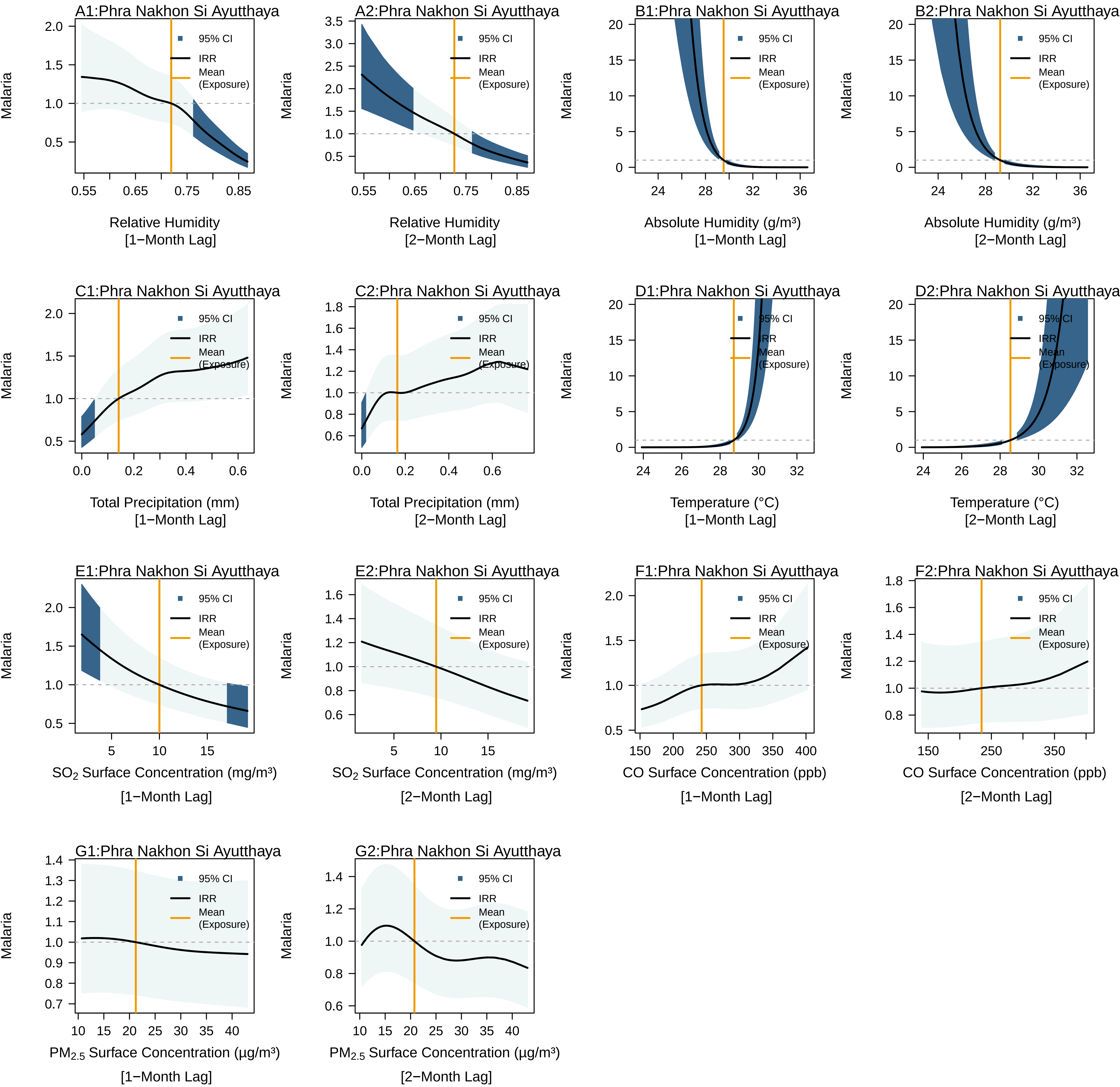
A1:Phra Nakhon Si Ayutthaya
A2:Phra Nakhon Si Ayutthaya
B1:Phra Nakhon Si Ayutthaya
B2:Phra Nakhon Si Ayutthaya
C1:Phra Nakhon Si Ayutthaya
C2:Phra Nakhon Si Ayutthaya
D1:Phra Nakhon Si Ayutthaya
D2:Phra Nakhon Si Ayutthaya
E1:Phra Nakhon Si Ayutthaya
E2:Phra Nakhon Si Ayutthaya
F1:Phra Nakhon Si Ayutthaya
F2:Phra Nakhon Si Ayutthaya
G1:Phra Nakhon Si Ayutthaya
G2:Phra Nakhon Si Ayutthaya
95% CI
IRR
Mean (Exposure)
Malaria
Relative Humidity [1−Month Lag]
Relative Humidity [2−Month Lag]
Absolute Humidity (g/m³) [1−Month Lag]
Absolute Humidity (g/m³) [2−Month Lag]
Total Precipitation (mm) [1−Month Lag]
Total Precipitation (mm) [2−Month Lag]
Temperature (°C) [1−Month Lag]
Temperature (°C) [2−Month Lag]
SO_2 Surface Concentration (mg/m³) [1−Month Lag]
SO_2 Surface Concentration (mg/m³) [2−Month Lag]
CO Surface Concentration (ppb) [1−Month Lag]
CO Surface Concentration (ppb) [2−Month Lag]
$PM_{2.5}$ Surface Concentration (µg/m³) [1−Month Lag]
$PM_{2.5}$ Surface Concentration (µg/m³) [2−Month Lag]

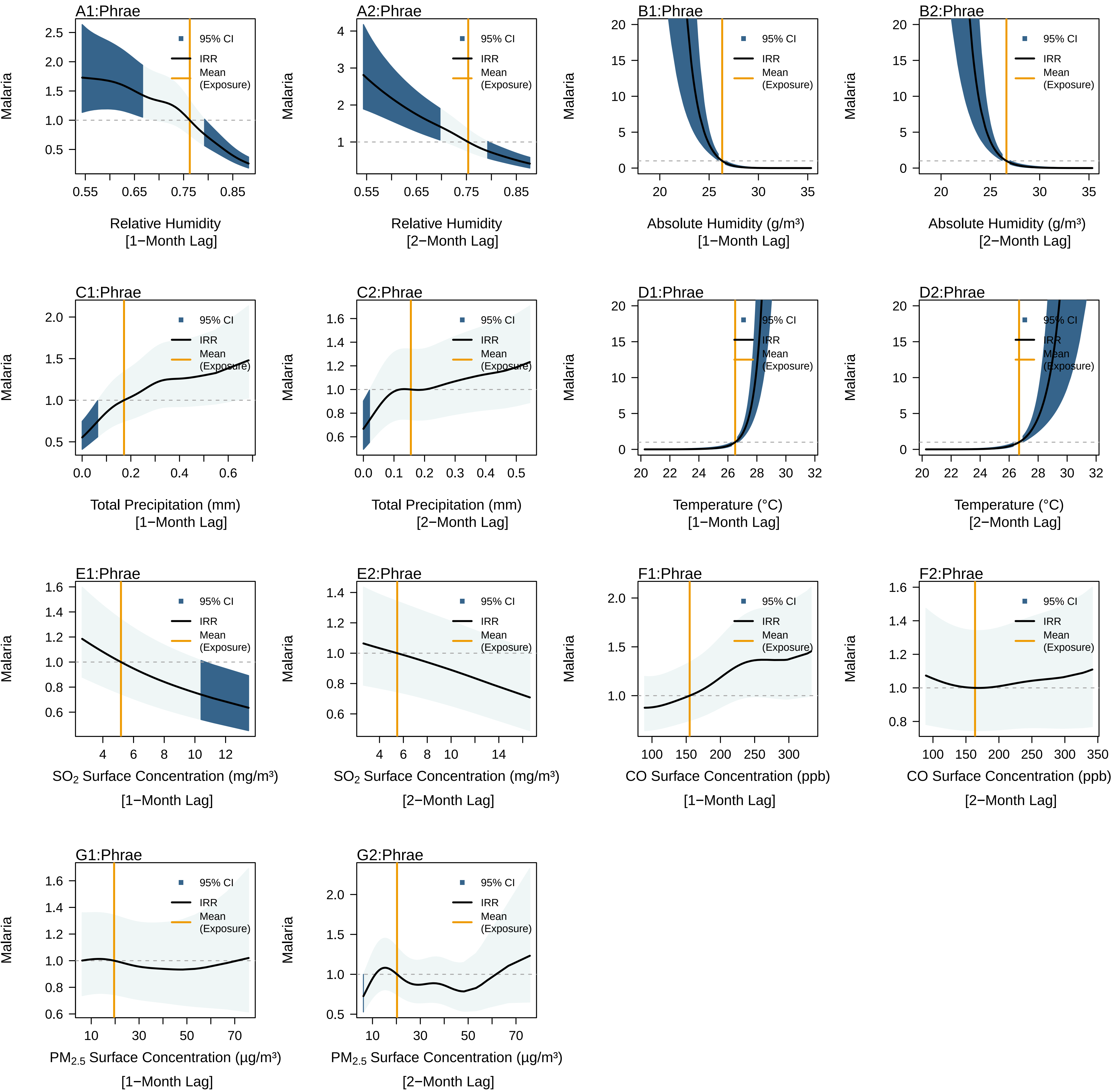

A1:Phrae
A2:Phrae
B1:Phrae
B2:Phrae
95% CI
IRR
Mean (Exposure)
Malaria
Relative Humidity [1−Month Lag]
Relative Humidity [2−Month Lag]
Absolute Humidity (g/m³) [1−Month Lag]
Absolute Humidity (g/m³) [2−Month Lag]
C1:Phrae
C2:Phrae
D1:Phrae
D2:Phrae
Total Precipitation (mm) [1−Month Lag]
Total Precipitation (mm) [2−Month Lag]
Temperature (°C) [1−Month Lag]
Temperature (°C) [2−Month Lag]
E1:Phrae
E2:Phrae
F1:Phrae
F2:Phrae
SO2 Surface Concentration (mg/m³) [1−Month Lag]
SO2 Surface Concentration (mg/m³) [2−Month Lag]
CO Surface Concentration (ppb) [1−Month Lag]
CO Surface Concentration (ppb) [2−Month Lag]
G1:Phrae
G2:Phrae
PM2.5 Surface Concentration (µg/m³) [1−Month Lag]
PM2.5 Surface Concentration (µg/m³) [2−Month Lag]

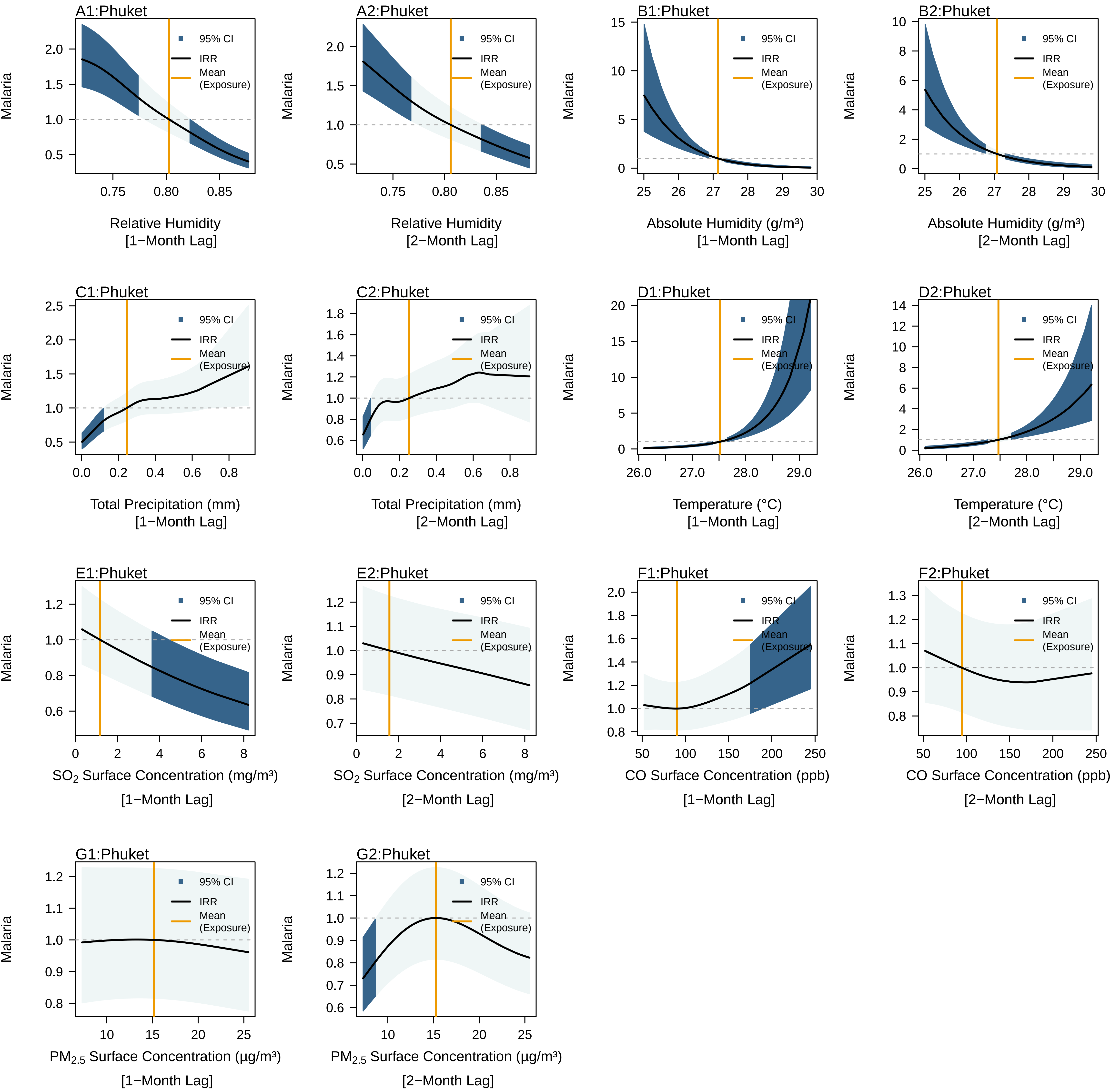
A1:Phuket
A2:Phuket
B1:Phuket
B2:Phuket
C1:Phuket
C2:Phuket
D1:Phuket
D2:Phuket
E1:Phuket
E2:Phuket
F1:Phuket
F2:Phuket
G1:Phuket
G2:Phuket
Malaria
95% CI
IRR
Mean (Exposure)
Relative Humidity [1−Month Lag]
Relative Humidity [2−Month Lag]
Absolute Humidity (g/m³) [1−Month Lag]
Absolute Humidity (g/m³) [2−Month Lag]
Total Precipitation (mm) [1−Month Lag]
Total Precipitation (mm) [2−Month Lag]
Temperature (°C) [1−Month Lag]
Temperature (°C) [2−Month Lag]
SO_2 Surface Concentration (mg/m³) [1−Month Lag]
SO_2 Surface Concentration (mg/m³) [2−Month Lag]
CO Surface Concentration (ppb) [1−Month Lag]
CO Surface Concentration (ppb) [2−Month Lag]
$PM_{2.5}$ Surface Concentration (µg/m³) [1−Month Lag]
$PM_{2.5}$ Surface Concentration (µg/m³) [2−Month Lag]

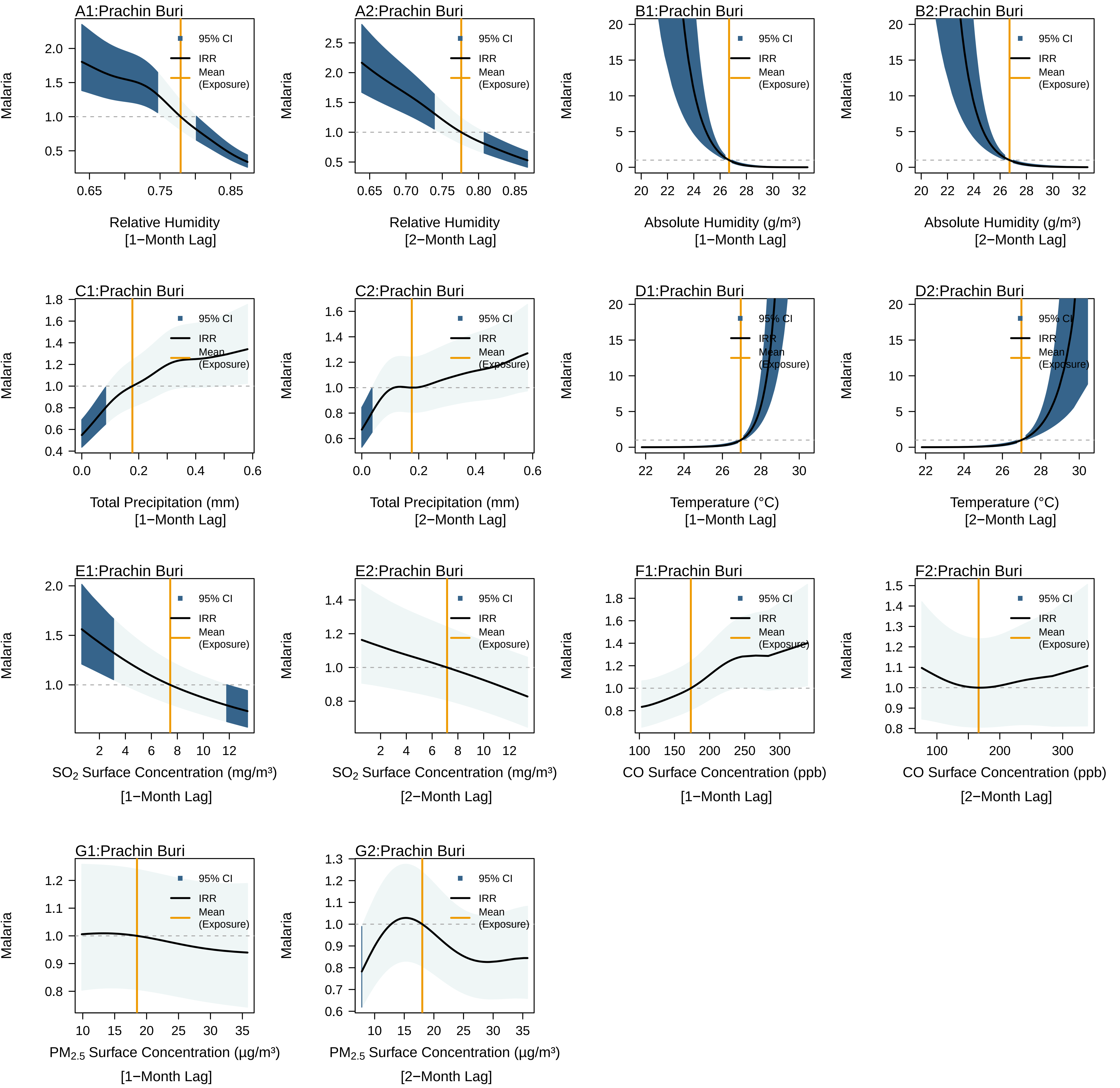
A1:Prachin Buri
Malaria
95% CI
IRR
Mean (Exposure)
Relative Humidity [1−Month Lag]
A2:Prachin Buri
Malaria
95% CI
IRR
Mean (Exposure)
Relative Humidity [2−Month Lag]
B1:Prachin Buri
Malaria
95% CI
IRR
Mean (Exposure)
Absolute Humidity (g/m³) [1−Month Lag]
B2:Prachin Buri
Malaria
95% CI
IRR
Mean (Exposure)
Absolute Humidity (g/m³) [2−Month Lag]
C1:Prachin Buri
Malaria
95% CI
IRR
Mean (Exposure)
Total Precipitation (mm) [1−Month Lag]
C2:Prachin Buri
Malaria
95% CI
IRR
Mean (Exposure)
Total Precipitation (mm) [2−Month Lag]
D1:Prachin Buri
Malaria
95% CI
IRR
Mean (Exposure)
Temperature (°C) [1−Month Lag]
D2:Prachin Buri
Malaria
95% CI
IRR
Mean (Exposure)
Temperature (°C) [2−Month Lag]
E1:Prachin Buri
Malaria
95% CI
IRR
Mean (Exposure)
SO2 Surface Concentration (mg/m³) [1−Month Lag]
E2:Prachin Buri
Malaria
95% CI
IRR
Mean (Exposure)
SO2 Surface Concentration (mg/m³) [2−Month Lag]
F1:Prachin Buri
Malaria
95% CI
IRR
Mean (Exposure)
CO Surface Concentration (ppb) [1−Month Lag]
F2:Prachin Buri
Malaria
95% CI
IRR
Mean (Exposure)
CO Surface Concentration (ppb) [2−Month Lag]
G1:Prachin Buri
Malaria
95% CI
IRR
Mean (Exposure)
PM2.5 Surface Concentration (μg/m³) [1−Month Lag]
G2:Prachin Buri
Malaria
95% CI
IRR
Mean (Exposure)
PM2.5 Surface Concentration (μg/m³) [2−Month Lag]

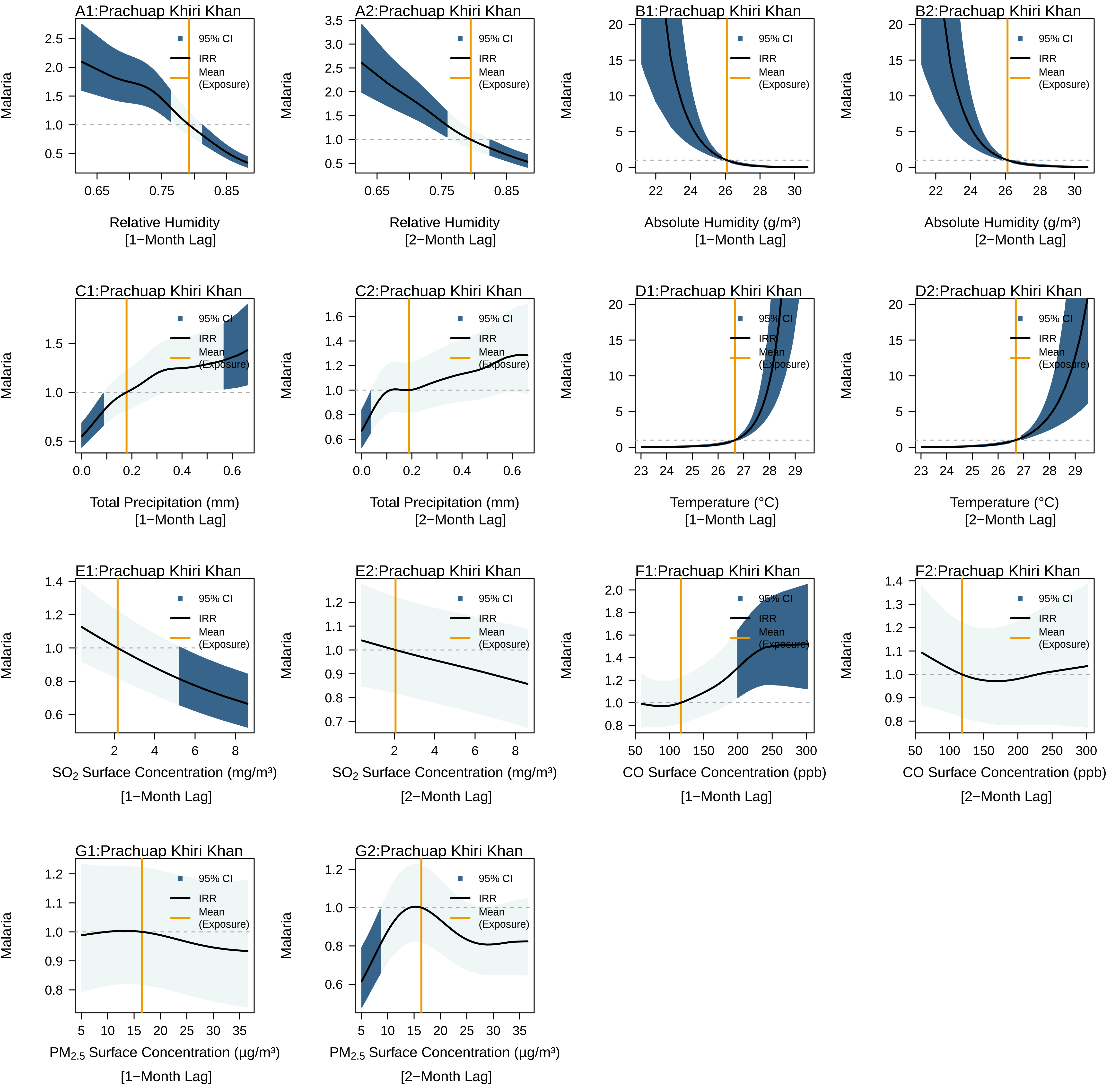

A1:Prachuap Khiri Khan
95% CI
IRR
Mean (Exposure)
Malaria
Relative Humidity [1−Month Lag]
A2:Prachuap Khiri Khan
Relative Humidity [2−Month Lag]
B1:Prachuap Khiri Khan
Absolute Humidity (g/m³) [1−Month Lag]
B2:Prachuap Khiri Khan
Absolute Humidity (g/m³) [2−Month Lag]
C1:Prachuap Khiri Khan
Total Precipitation (mm) [1−Month Lag]
C2:Prachuap Khiri Khan
Total Precipitation (mm) [2−Month Lag]
D1:Prachuap Khiri Khan
Temperature (°C) [1−Month Lag]
D2:Prachuap Khiri Khan
Temperature (°C) [2−Month Lag]
E1:Prachuap Khiri Khan
SO_2 Surface Concentration (mg/m³) [1−Month Lag]
E2:Prachuap Khiri Khan
SO_2 Surface Concentration (mg/m³) [2−Month Lag]
F1:Prachuap Khiri Khan
CO Surface Concentration (ppb) [1−Month Lag]
F2:Prachuap Khiri Khan
CO Surface Concentration (ppb) [2−Month Lag]
G1:Prachuap Khiri Khan
$PM_{2.5}$ Surface Concentration (µg/m³) [1−Month Lag]
G2:Prachuap Khiri Khan
$PM_{2.5}$ Surface Concentration (µg/m³) [2−Month Lag]

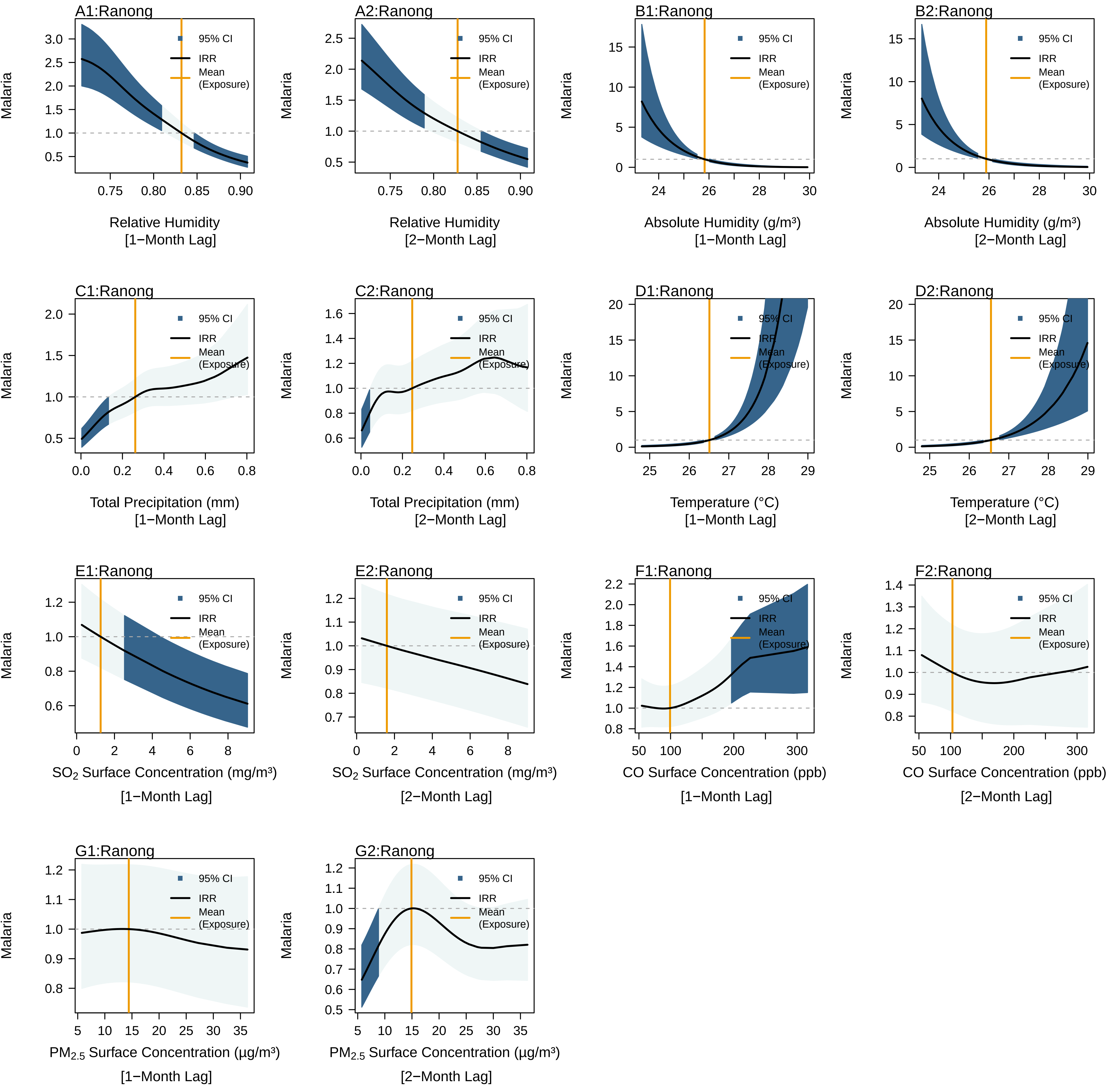
A1:Ranong
95% CI
IRR
Mean (Exposure)
Malaria
Relative Humidity [1−Month Lag]
A2:Ranong
Relative Humidity [2−Month Lag]
B1:Ranong
Absolute Humidity (g/m³) [1−Month Lag]
B2:Ranong
Absolute Humidity (g/m³) [2−Month Lag]
C1:Ranong
Total Precipitation (mm) [1−Month Lag]
C2:Ranong
Total Precipitation (mm) [2−Month Lag]
D1:Ranong
Temperature (°C) [1−Month Lag]
D2:Ranong
Temperature (°C) [2−Month Lag]
E1:Ranong
SO_2 Surface Concentration (mg/m³) [1−Month Lag]
E2:Ranong
SO_2 Surface Concentration (mg/m³) [2−Month Lag]
F1:Ranong
CO Surface Concentration (ppb) [1−Month Lag]
F2:Ranong
CO Surface Concentration (ppb) [2−Month Lag]
G1:Ranong
$PM_{2.5}$ Surface Concentration (μg/m³) [1−Month Lag]
G2:Ranong
$PM_{2.5}$ Surface Concentration (μg/m³) [2−Month Lag]

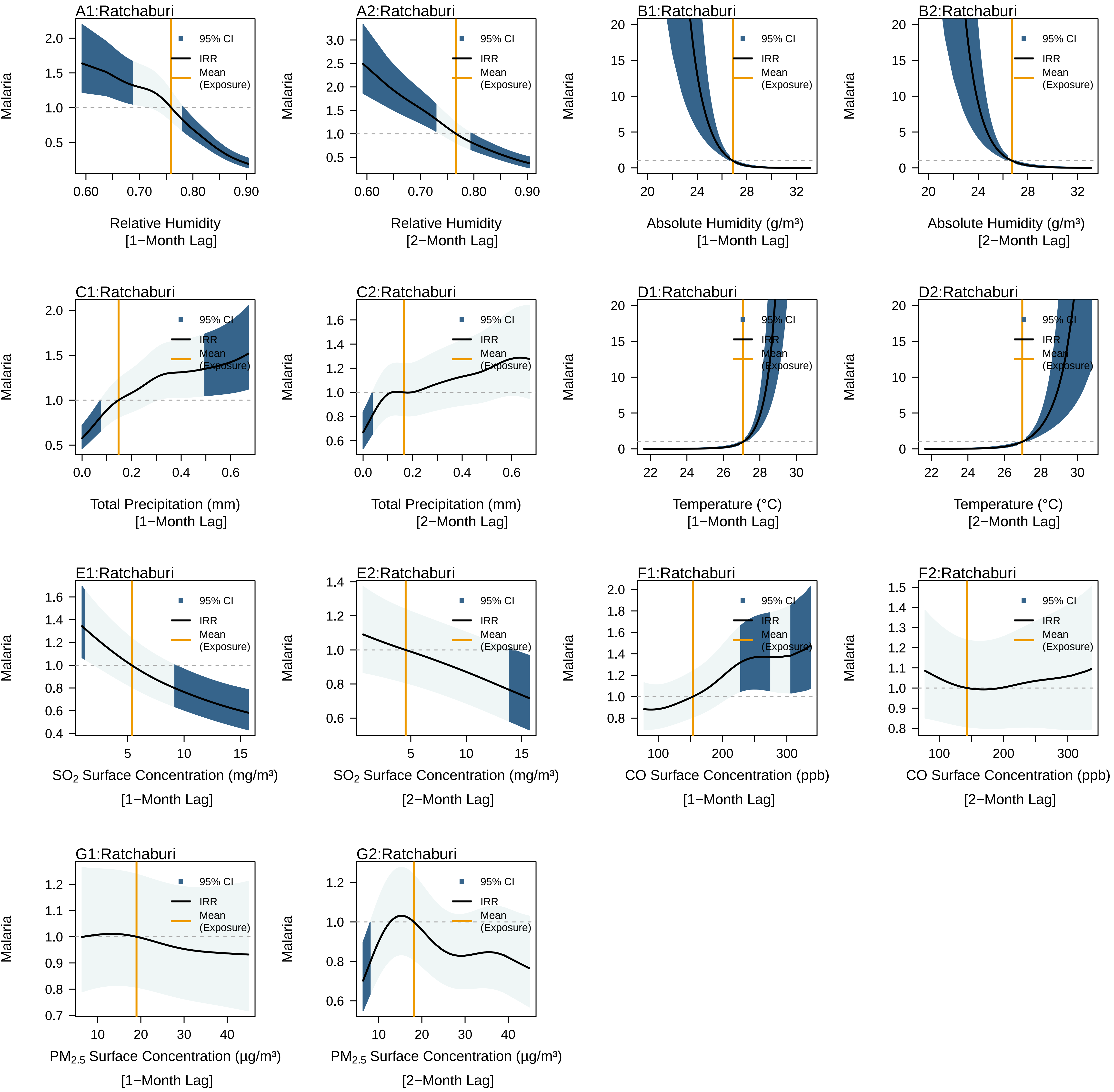

A1:Ratchaburi
Malaria
95% CI
IRR
Mean (Exposure)
Relative Humidity [1−Month Lag]
A2:Ratchaburi
Malaria
95% CI
IRR
Mean (Exposure)
Relative Humidity [2−Month Lag]
B1:Ratchaburi
Malaria
95% CI
IRR
Mean (Exposure)
Absolute Humidity (g/m³) [1−Month Lag]
B2:Ratchaburi
Malaria
95% CI
IRR
Mean (Exposure)
Absolute Humidity (g/m³) [2−Month Lag]
C1:Ratchaburi
Malaria
95% CI
IRR
Mean (Exposure)
Total Precipitation (mm) [1−Month Lag]
C2:Ratchaburi
Malaria
95% CI
IRR
Mean (Exposure)
Total Precipitation (mm) [2−Month Lag]
D1:Ratchaburi
Malaria
95% CI
IRR
Mean (Exposure)
Temperature (°C) [1−Month Lag]
D2:Ratchaburi
Malaria
95% CI
IRR
Mean (Exposure)
Temperature (°C) [2−Month Lag]
E1:Ratchaburi
Malaria
95% CI
IRR
Mean (Exposure)
SO_2 Surface Concentration (mg/m³) [1−Month Lag]
E2:Ratchaburi
Malaria
95% CI
IRR
Mean (Exposure)
SO_2 Surface Concentration (mg/m³) [2−Month Lag]
F1:Ratchaburi
Malaria
95% CI
IRR
Mean (Exposure)
CO Surface Concentration (ppb) [1−Month Lag]
F2:Ratchaburi
Malaria
95% CI
IRR
Mean (Exposure)
CO Surface Concentration (ppb) [2−Month Lag]
G1:Ratchaburi
Malaria
95% CI
IRR
Mean (Exposure)
$PM_{2.5}$ Surface Concentration (µg/m³) [1−Month Lag]
G2:Ratchaburi
Malaria
95% CI
IRR
Mean (Exposure)
$PM_{2.5}$ Surface Concentration (µg/m³) [2−Month Lag]

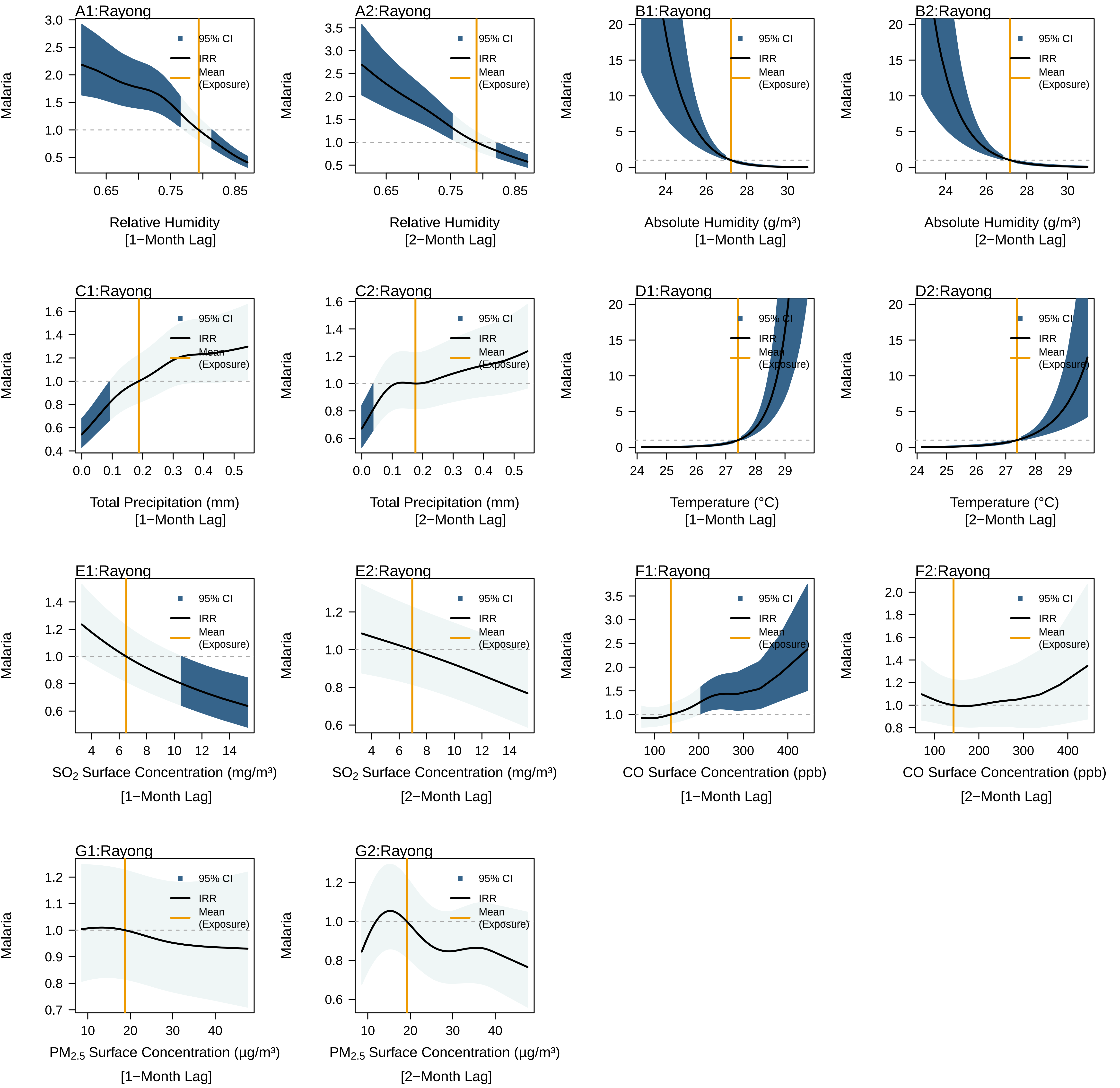
A1:Rayong
Relative Humidity
[1−Month Lag]
A2:Rayong
Relative Humidity
[2−Month Lag]
B1:Rayong
Absolute Humidity (g/m³)
[1−Month Lag]
B2:Rayong
Absolute Humidity (g/m³)
[2−Month Lag]
C1:Rayong
Total Precipitation (mm)
[1−Month Lag]
C2:Rayong
Total Precipitation (mm)
[2−Month Lag]
D1:Rayong
Temperature (°C)
[1−Month Lag]
D2:Rayong
Temperature (°C)
[2−Month Lag]
E1:Rayong
SO_2 Surface Concentration (mg/m³)
[1−Month Lag]
E2:Rayong
SO_2 Surface Concentration (mg/m³)
[2−Month Lag]
F1:Rayong
CO Surface Concentration (ppb)
[1−Month Lag]
F2:Rayong
CO Surface Concentration (ppb)
[2−Month Lag]
G1:Rayong
$PM_{2.5}$ Surface Concentration (µg/m³)
[1−Month Lag]
G2:Rayong
$PM_{2.5}$ Surface Concentration (µg/m³)
[2−Month Lag]
Malaria
95% CI
IRR
Mean
(Exposure)

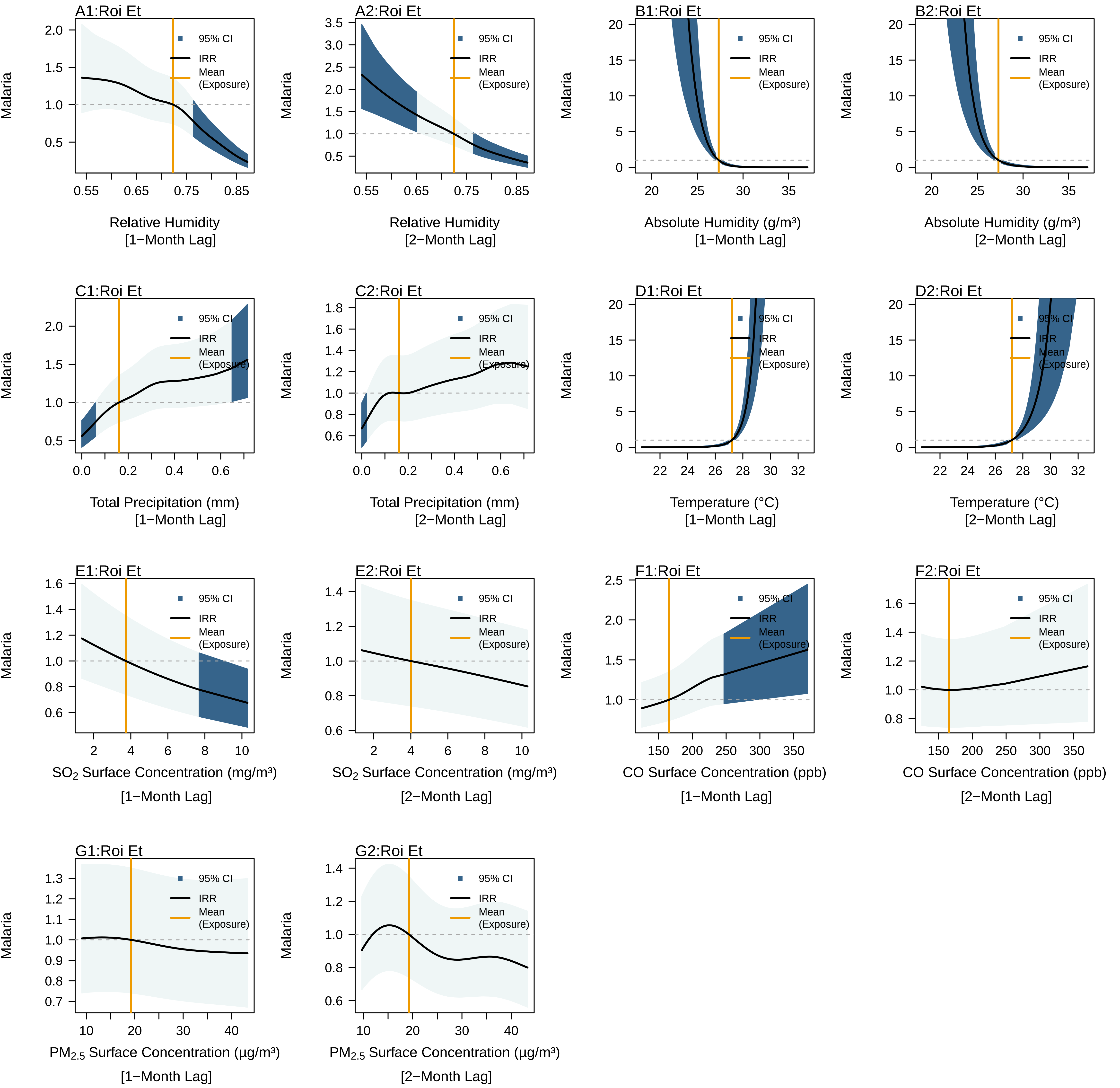
A1:Roi Et
A2:Roi Et
B1:Roi Et
B2:Roi Et
C1:Roi Et
C2:Roi Et
D1:Roi Et
D2:Roi Et
E1:Roi Et
E2:Roi Et
F1:Roi Et
F2:Roi Et
G1:Roi Et
G2:Roi Et
Malaria
95% CI
IRR
Mean (Exposure)
Relative Humidity [1−Month Lag]
Relative Humidity [2−Month Lag]
Absolute Humidity (g/m³) [1−Month Lag]
Absolute Humidity (g/m³) [2−Month Lag]
Total Precipitation (mm) [1−Month Lag]
Total Precipitation (mm) [2−Month Lag]
Temperature (°C) [1−Month Lag]
Temperature (°C) [2−Month Lag]
SO2 Surface Concentration (mg/m³) [1−Month Lag]
SO2 Surface Concentration (mg/m³) [2−Month Lag]
CO Surface Concentration (ppb) [1−Month Lag]
CO Surface Concentration (ppb) [2−Month Lag]
PM2.5 Surface Concentration (μg/m³) [1−Month Lag]
PM2.5 Surface Concentration (μg/m³) [2−Month Lag]

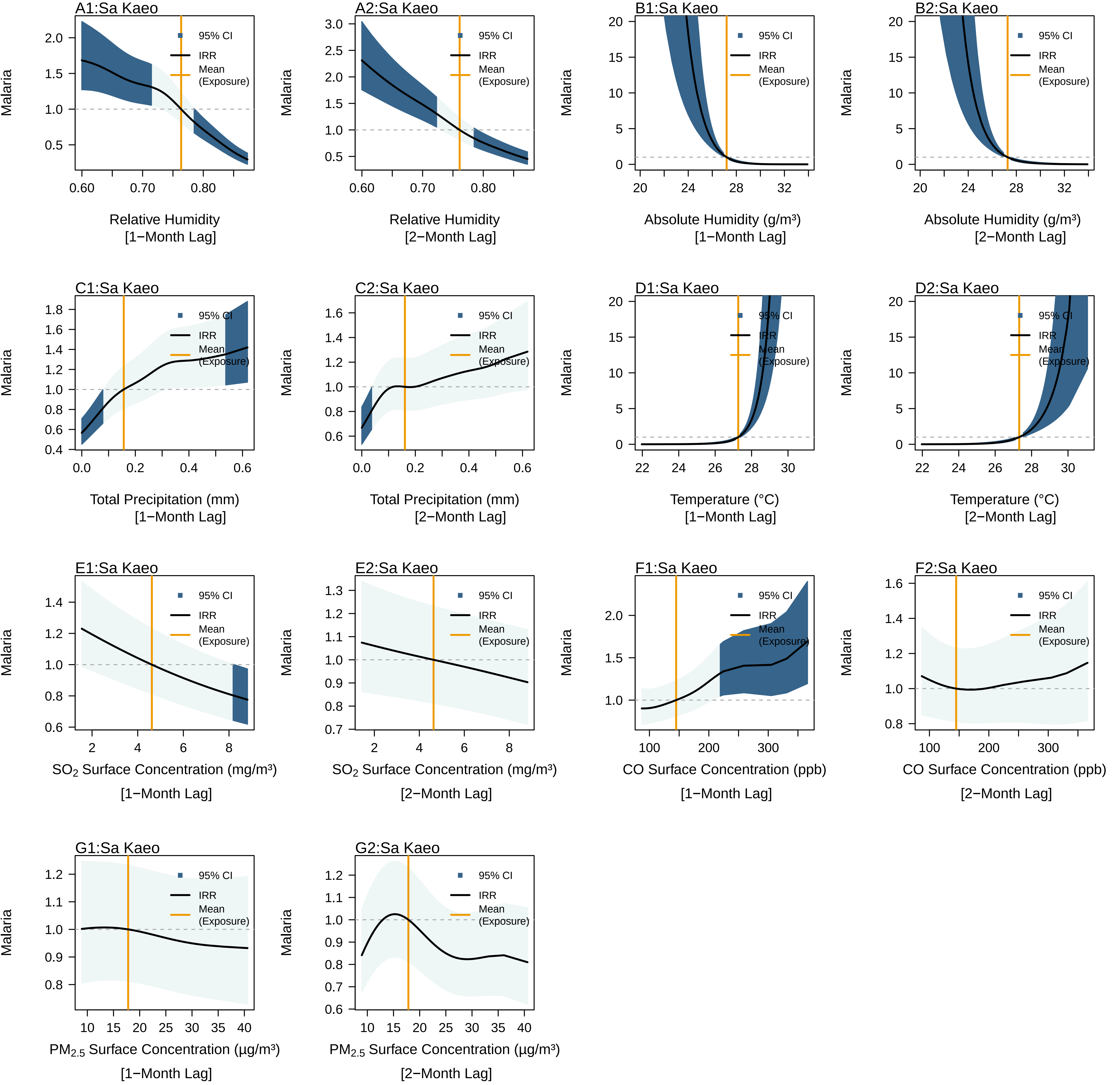
A1:Sa Kaeo
Malaria
95% CI
IRR
Mean (Exposure)
Relative Humidity [1−Month Lag]
A2:Sa Kaeo
Relative Humidity [2−Month Lag]
B1:Sa Kaeo
Absolute Humidity (g/m³) [1−Month Lag]
B2:Sa Kaeo
Absolute Humidity (g/m³) [2−Month Lag]
C1:Sa Kaeo
Total Precipitation (mm) [1−Month Lag]
C2:Sa Kaeo
Total Precipitation (mm) [2−Month Lag]
D1:Sa Kaeo
Temperature (°C) [1−Month Lag]
D2:Sa Kaeo
Temperature (°C) [2−Month Lag]
E1:Sa Kaeo
SO2 Surface Concentration (mg/m³) [1−Month Lag]
E2:Sa Kaeo
SO2 Surface Concentration (mg/m³) [2−Month Lag]
F1:Sa Kaeo
CO Surface Concentration (ppb) [1−Month Lag]
F2:Sa Kaeo
CO Surface Concentration (ppb) [2−Month Lag]
G1:Sa Kaeo
PM2.5 Surface Concentration (µg/m³) [1−Month Lag]
G2:Sa Kaeo
PM2.5 Surface Concentration (µg/m³) [2−Month Lag]

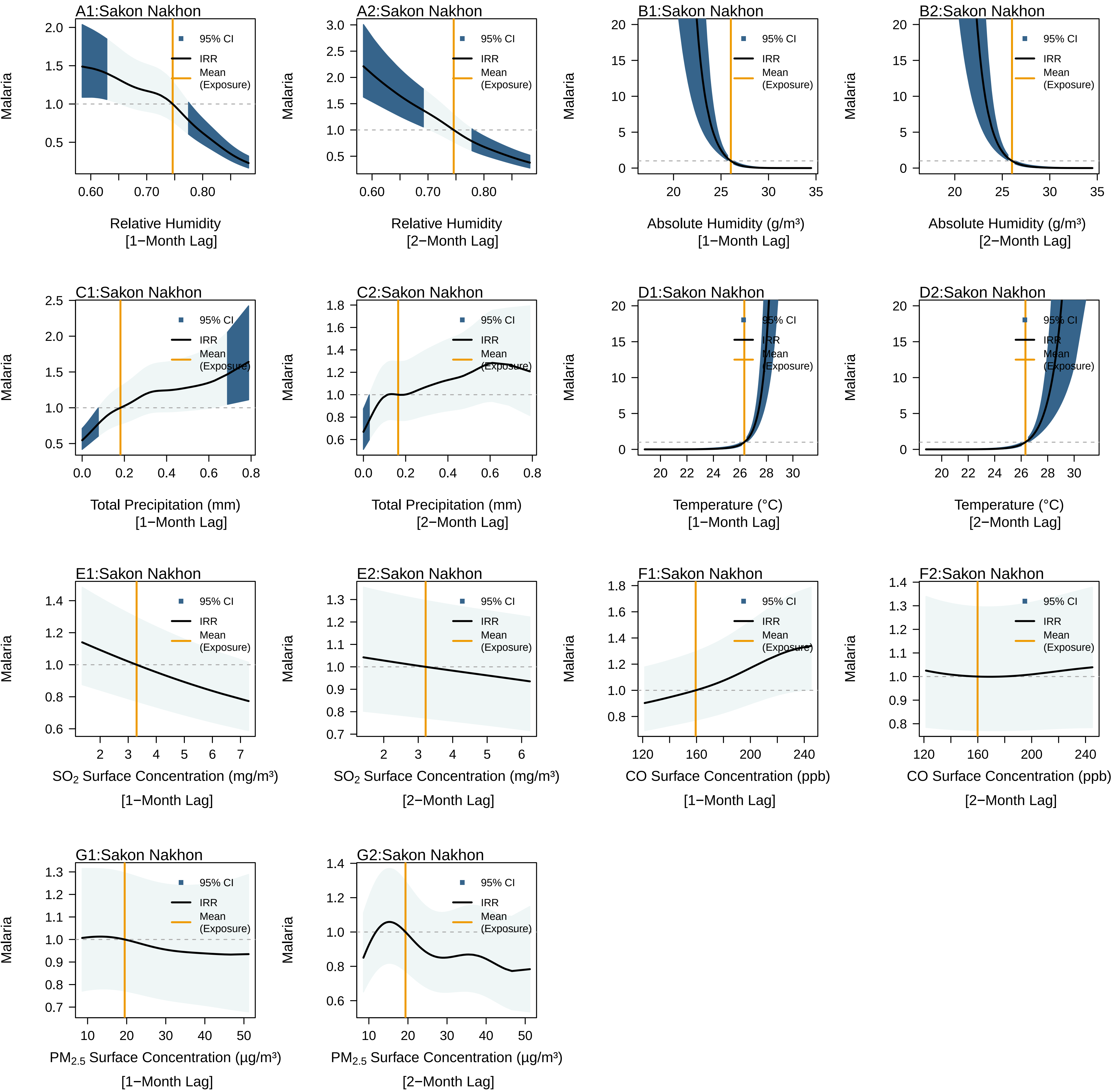
A1:Sakon Nakhon
A2:Sakon Nakhon
B1:Sakon Nakhon
B2:Sakon Nakhon
C1:Sakon Nakhon
C2:Sakon Nakhon
D1:Sakon Nakhon
D2:Sakon Nakhon
E1:Sakon Nakhon
E2:Sakon Nakhon
F1:Sakon Nakhon
F2:Sakon Nakhon
G1:Sakon Nakhon
G2:Sakon Nakhon
95% CI
IRR
Mean (Exposure)
Malaria
Relative Humidity [1−Month Lag]
Relative Humidity [2−Month Lag]
Absolute Humidity (g/m³) [1−Month Lag]
Absolute Humidity (g/m³) [2−Month Lag]
Total Precipitation (mm) [1−Month Lag]
Total Precipitation (mm) [2−Month Lag]
Temperature (°C) [1−Month Lag]
Temperature (°C) [2−Month Lag]
SO2 Surface Concentration (mg/m³) [1−Month Lag]
SO2 Surface Concentration (mg/m³) [2−Month Lag]
CO Surface Concentration (ppb) [1−Month Lag]
CO Surface Concentration (ppb) [2−Month Lag]
PM2.5 Surface Concentration (µg/m³) [1−Month Lag]
PM2.5 Surface Concentration (µg/m³) [2−Month Lag]

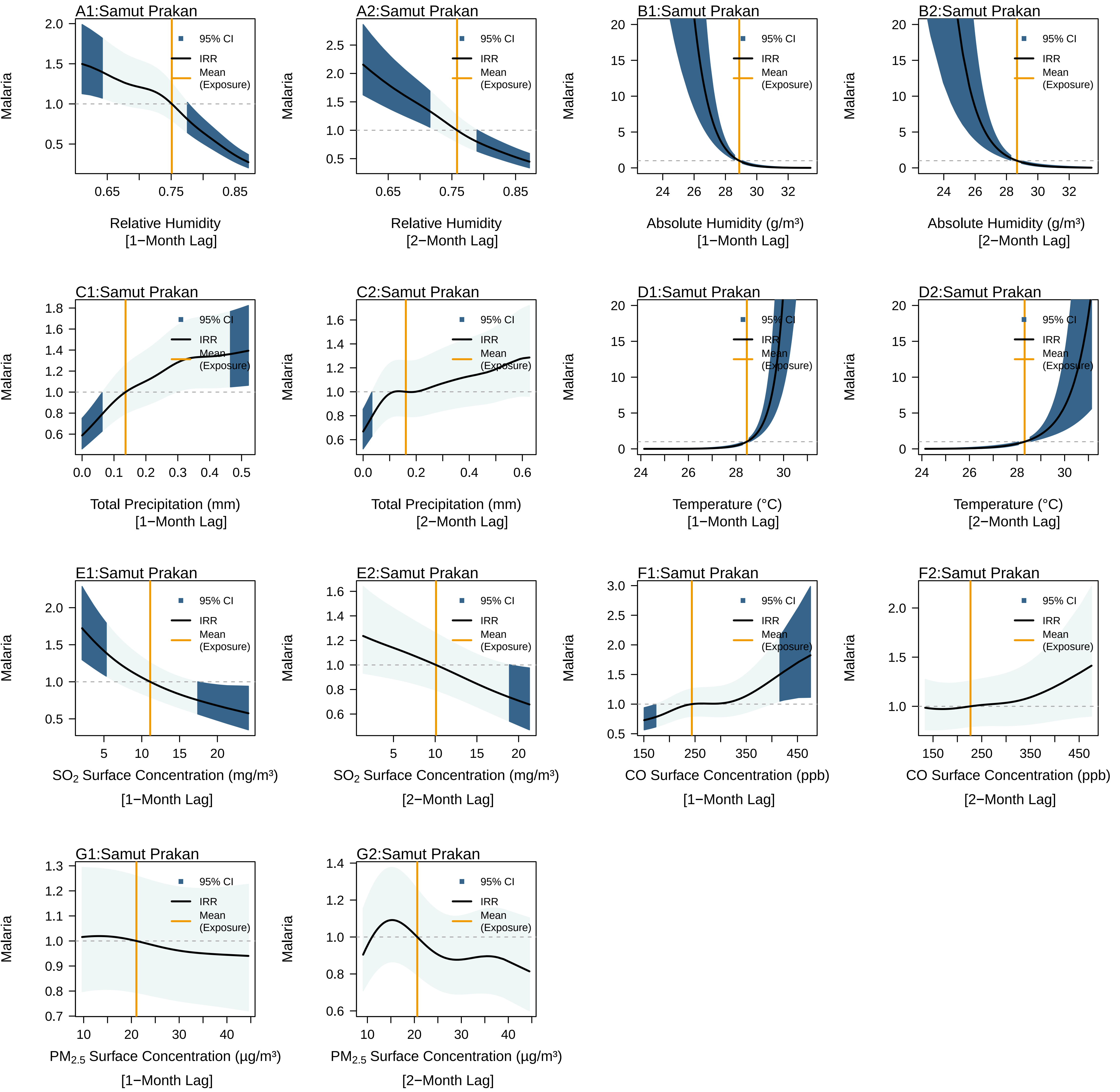
A1:Samut Prakan
Malaria
95% CI
IRR
Mean (Exposure)
Relative Humidity [1−Month Lag]
A2:Samut Prakan
Malaria
95% CI
IRR
Mean (Exposure)
Relative Humidity [2−Month Lag]
B1:Samut Prakan
Malaria
95% CI
IRR
Mean (Exposure)
Absolute Humidity (g/m³) [1−Month Lag]
B2:Samut Prakan
Malaria
95% CI
IRR
Mean (Exposure)
Absolute Humidity (g/m³) [2−Month Lag]
C1:Samut Prakan
Malaria
95% CI
IRR
Mean (Exposure)
Total Precipitation (mm) [1−Month Lag]
C2:Samut Prakan
Malaria
95% CI
IRR
Mean (Exposure)
Total Precipitation (mm) [2−Month Lag]
D1:Samut Prakan
Malaria
95% CI
IRR
Mean (Exposure)
Temperature (°C) [1−Month Lag]
D2:Samut Prakan
Malaria
95% CI
IRR
Mean (Exposure)
Temperature (°C) [2−Month Lag]
E1:Samut Prakan
Malaria
95% CI
IRR
Mean (Exposure)
SO2 Surface Concentration (mg/m³) [1−Month Lag]
E2:Samut Prakan
Malaria
95% CI
IRR
Mean (Exposure)
SO2 Surface Concentration (mg/m³) [2−Month Lag]
F1:Samut Prakan
Malaria
95% CI
IRR
Mean (Exposure)
CO Surface Concentration (ppb) [1−Month Lag]
F2:Samut Prakan
Malaria
95% CI
IRR
Mean (Exposure)
CO Surface Concentration (ppb) [2−Month Lag]
G1:Samut Prakan
Malaria
95% CI
IRR
Mean (Exposure)
PM2.5 Surface Concentration (µg/m³) [1−Month Lag]
G2:Samut Prakan
Malaria
95% CI
IRR
Mean (Exposure)
PM2.5 Surface Concentration (µg/m³) [2−Month Lag]

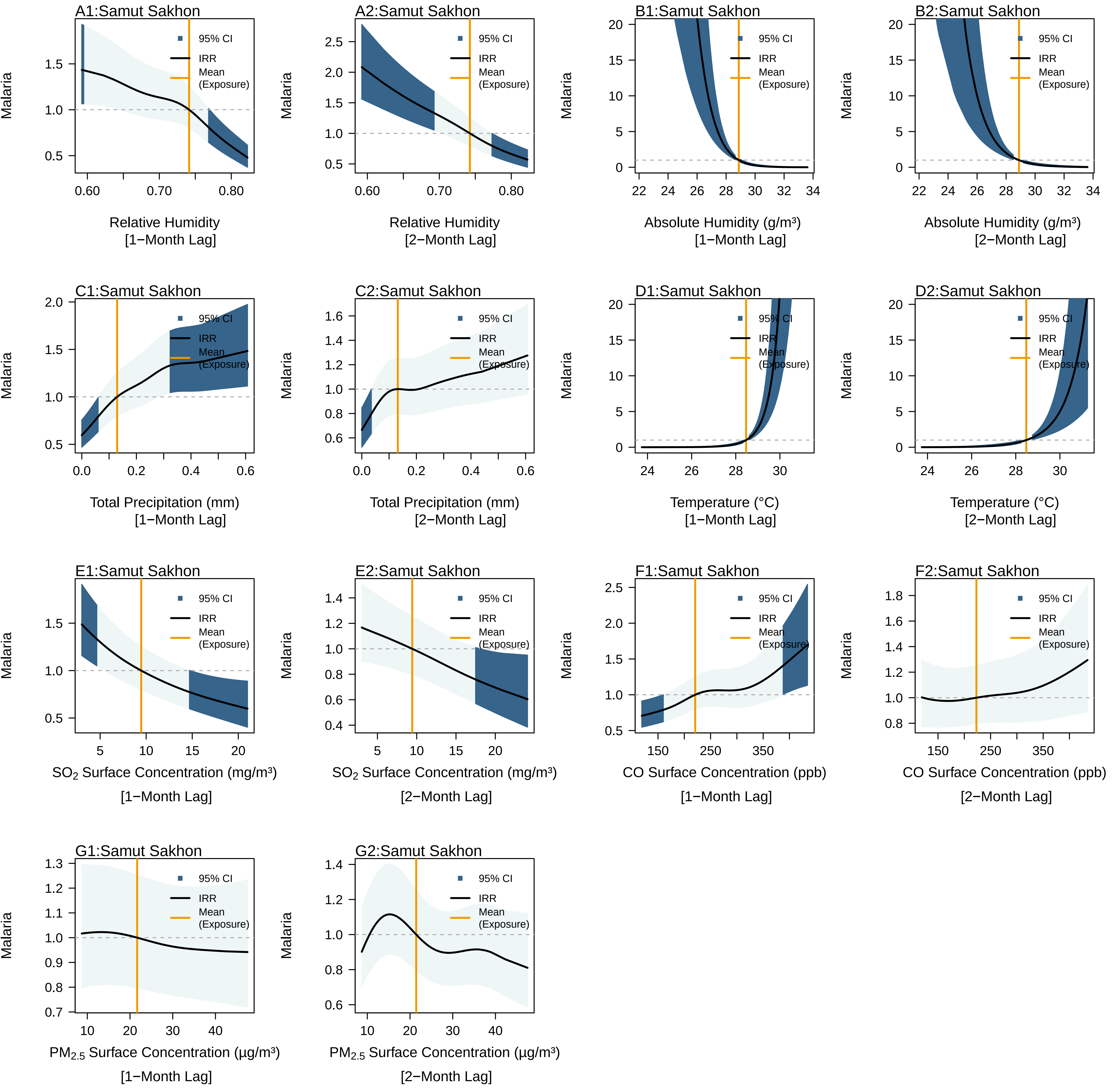
A1:Samut Sakhon
Malaria
0.5 1.0 1.5
0.60 0.70 0.80
95% CI
IRR
Mean (Exposure)
Relative Humidity [1−Month Lag]
A2:Samut Sakhon
Malaria
0.5 1.0 1.5 2.0 2.5
0.60 0.70 0.80
95% CI
IRR
Mean (Exposure)
Relative Humidity [2−Month Lag]
B1:Samut Sakhon
Malaria
0 5 10 15 20
22 24 26 28 30 32 34
95% CI
IRR
Mean (Exposure)
Absolute Humidity (g/m³) [1−Month Lag]
B2:Samut Sakhon
Malaria
0 5 10 15 20
22 24 26 28 30 32 34
95% CI
IRR
Mean (Exposure)
Absolute Humidity (g/m³) [2−Month Lag]
C1:Samut Sakhon
Malaria
0.5 1.0 1.5 2.0
0.0 0.2 0.4 0.6
95% CI
IRR
Mean (Exposure)
Total Precipitation (mm) [1−Month Lag]
C2:Samut Sakhon
Malaria
0.6 0.8 1.0 1.2 1.4 1.6
0.0 0.2 0.4 0.6
95% CI
IRR
Mean (Exposure)
Total Precipitation (mm) [2−Month Lag]
D1:Samut Sakhon
Malaria
0 5 10 15 20
24 26 28 30
95% CI
IRR
Mean (Exposure)
Temperature (°C) [1−Month Lag]
D2:Samut Sakhon
Malaria
0 5 10 15 20
24 26 28 30
95% CI
IRR
Mean (Exposure)
Temperature (°C) [2−Month Lag]
E1:Samut Sakhon
Malaria
0.5 1.0 1.5
5 10 15 20
95% CI
IRR
Mean (Exposure)
SO2 Surface Concentration (mg/m³) [1−Month Lag]
E2:Samut Sakhon
Malaria
0.4 0.6 0.8 1.0 1.2 1.4
5 10 15 20
95% CI
IRR
Mean (Exposure)
SO2 Surface Concentration (mg/m³) [2−Month Lag]
F1:Samut Sakhon
Malaria
0.5 1.0 1.5 2.0 2.5
150 250 350
95% CI
IRR
Mean (Exposure)
CO Surface Concentration (ppb) [1−Month Lag]
F2:Samut Sakhon
Malaria
0.8 1.0 1.2 1.4 1.6 1.8
150 250 350
95% CI
IRR
Mean (Exposure)
CO Surface Concentration (ppb) [2−Month Lag]
G1:Samut Sakhon
Malaria
0.7 0.8 0.9 1.0 1.1 1.2 1.3
10 20 30 40
95% CI
IRR
Mean (Exposure)
PM2.5 Surface Concentration (µg/m³) [1−Month Lag]
G2:Samut Sakhon
Malaria
0.6 0.8 1.0 1.2 1.4
10 20 30 40
95% CI
IRR
Mean (Exposure)
PM2.5 Surface Concentration (µg/m³) [2−Month Lag]

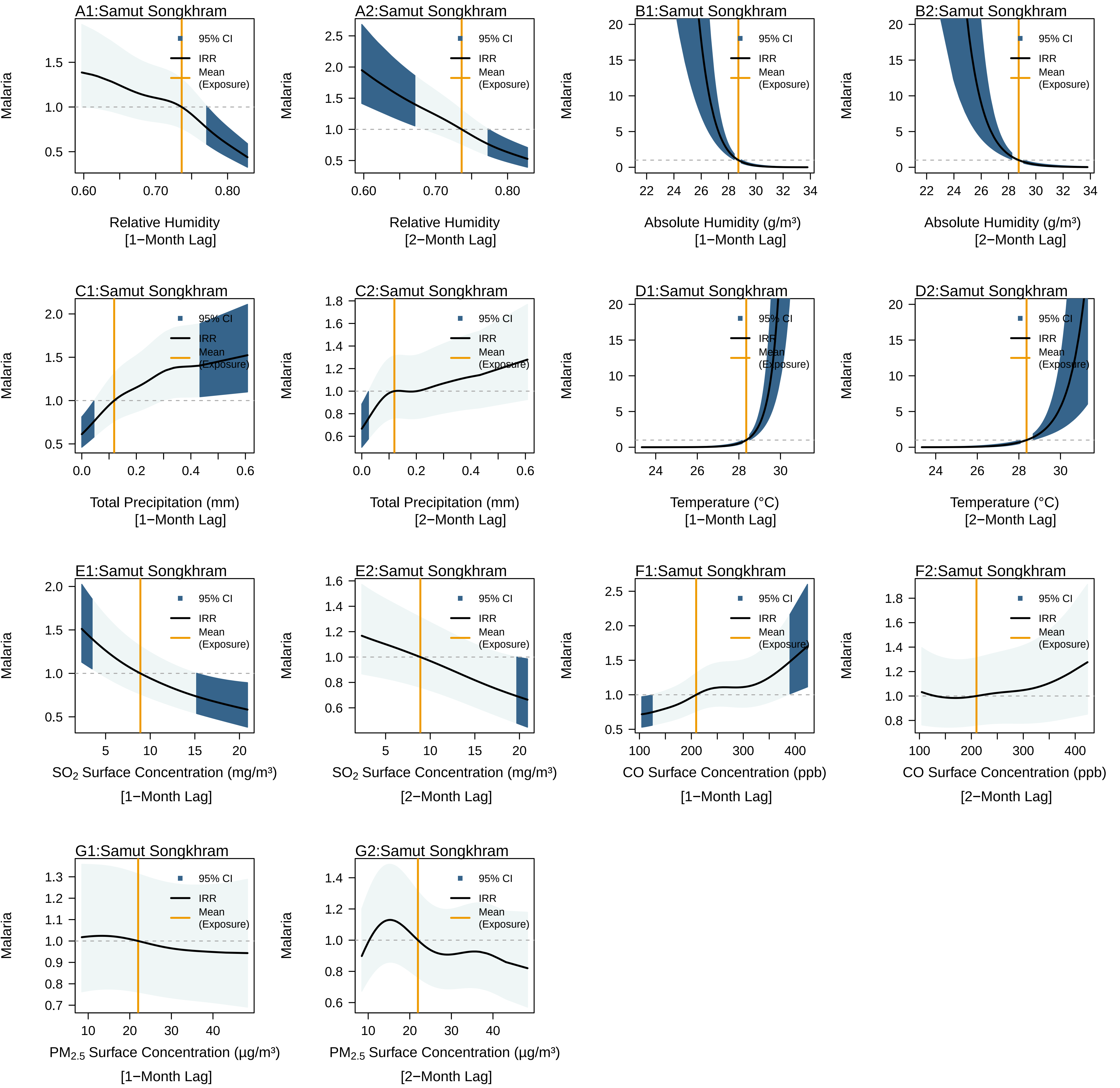
A1:Samut Songkhram
A2:Samut Songkhram
B1:Samut Songkhram
B2:Samut Songkhram
C1:Samut Songkhram
C2:Samut Songkhram
D1:Samut Songkhram
D2:Samut Songkhram
E1:Samut Songkhram
E2:Samut Songkhram
F1:Samut Songkhram
F2:Samut Songkhram
G1:Samut Songkhram
G2:Samut Songkhram
95% CI
IRR
Mean (Exposure)
Malaria
Relative Humidity [1−Month Lag]
Relative Humidity [2−Month Lag]
Absolute Humidity (g/m³) [1−Month Lag]
Absolute Humidity (g/m³) [2−Month Lag]
Total Precipitation (mm) [1−Month Lag]
Total Precipitation (mm) [2−Month Lag]
Temperature (°C) [1−Month Lag]
Temperature (°C) [2−Month Lag]
SO2 Surface Concentration (mg/m³) [1−Month Lag]
SO2 Surface Concentration (mg/m³) [2−Month Lag]
CO Surface Concentration (ppb) [1−Month Lag]
CO Surface Concentration (ppb) [2−Month Lag]
PM2.5 Surface Concentration (µg/m³) [1−Month Lag]
PM2.5 Surface Concentration (µg/m³) [2−Month Lag]

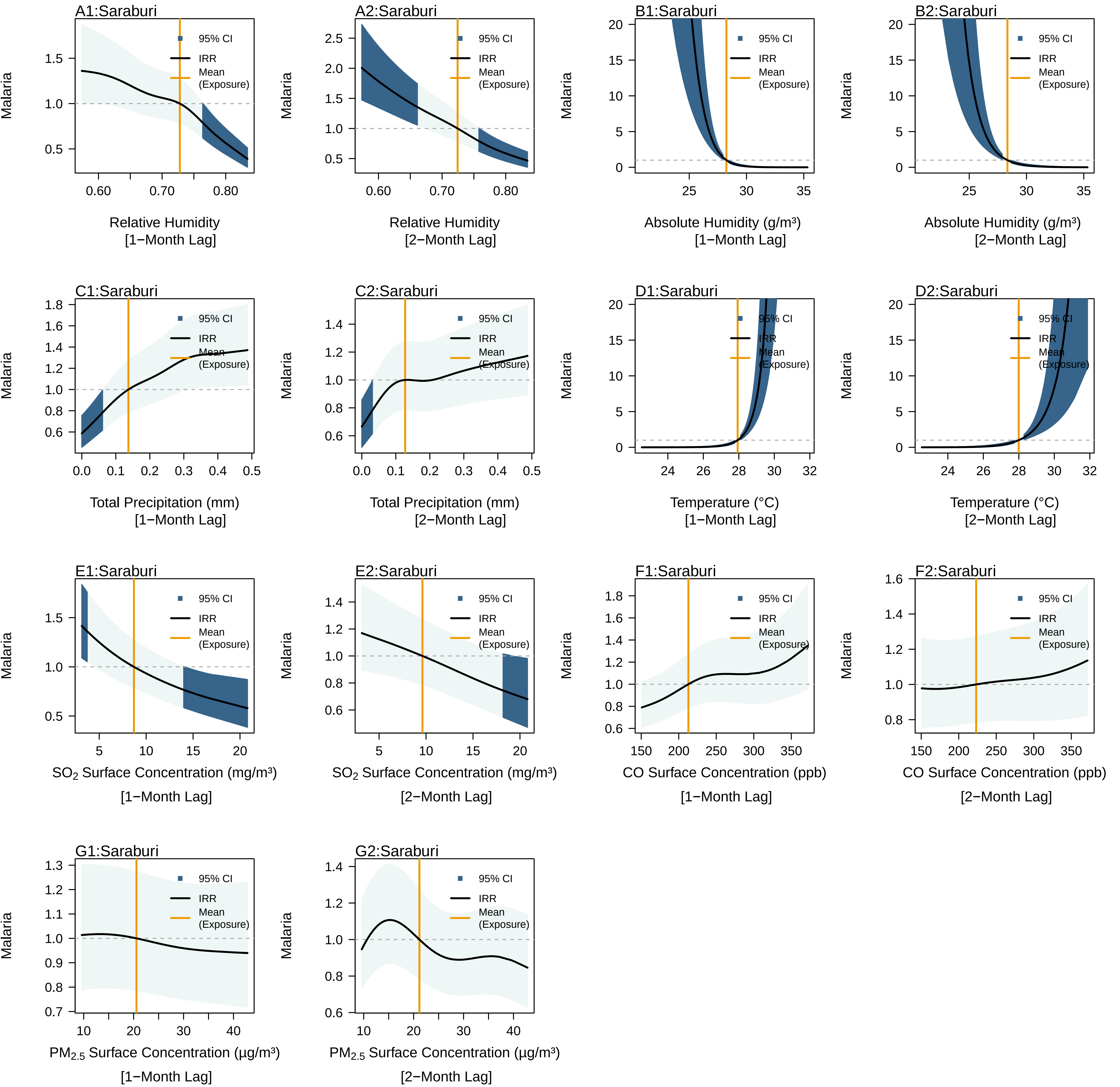

A1:Saraburi
Malaria
95% CI
IRR
Mean (Exposure)
Relative Humidity [1−Month Lag]
A2:Saraburi
Malaria
95% CI
IRR
Mean (Exposure)
Relative Humidity [2−Month Lag]
B1:Saraburi
Malaria
95% CI
IRR
Mean (Exposure)
Absolute Humidity (g/m³) [1−Month Lag]
B2:Saraburi
Malaria
95% CI
IRR
Mean (Exposure)
Absolute Humidity (g/m³) [2−Month Lag]
C1:Saraburi
Malaria
95% CI
IRR
Mean (Exposure)
Total Precipitation (mm) [1−Month Lag]
C2:Saraburi
Malaria
95% CI
IRR
Mean (Exposure)
Total Precipitation (mm) [2−Month Lag]
D1:Saraburi
Malaria
95% CI
IRR
Mean (Exposure)
Temperature (°C) [1−Month Lag]
D2:Saraburi
Malaria
95% CI
IRR
Mean (Exposure)
Temperature (°C) [2−Month Lag]
E1:Saraburi
Malaria
95% CI
IRR
Mean (Exposure)
SO2 Surface Concentration (mg/m³) [1−Month Lag]
E2:Saraburi
Malaria
95% CI
IRR
Mean (Exposure)
SO2 Surface Concentration (mg/m³) [2−Month Lag]
F1:Saraburi
Malaria
95% CI
IRR
Mean (Exposure)
CO Surface Concentration (ppb) [1−Month Lag]
F2:Saraburi
Malaria
95% CI
IRR
Mean (Exposure)
CO Surface Concentration (ppb) [2−Month Lag]
G1:Saraburi
Malaria
95% CI
IRR
Mean (Exposure)
PM2.5 Surface Concentration (µg/m³) [1−Month Lag]
G2:Saraburi
Malaria
95% CI
IRR
Mean (Exposure)
PM2.5 Surface Concentration (µg/m³) [2−Month Lag]

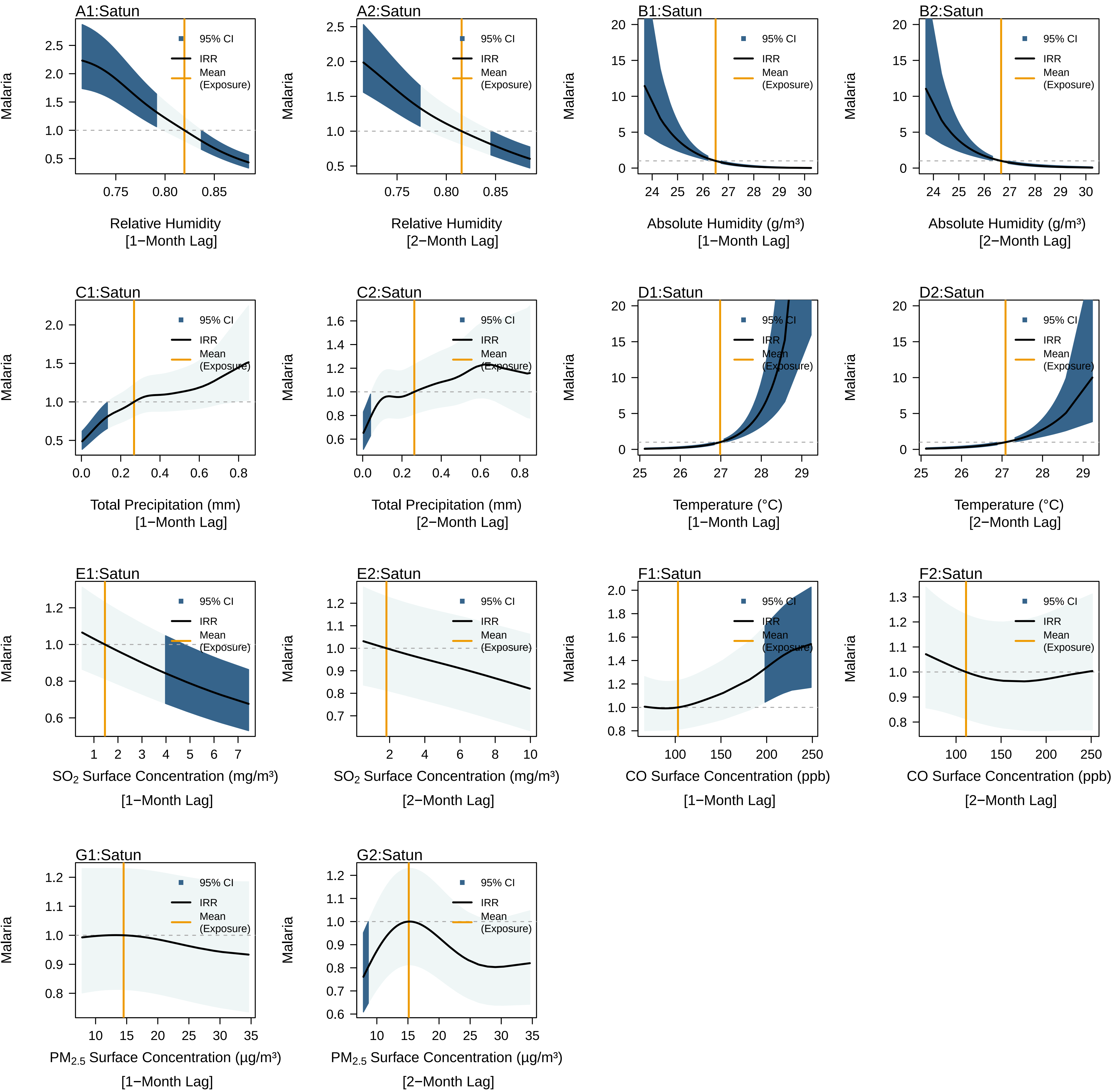

A1:Satun
95% CI
IRR
Mean
(Exposure)
Malaria
Relative Humidity
[1−Month Lag]
A2:Satun
Malaria
Relative Humidity
[2−Month Lag]
B1:Satun
Malaria
Absolute Humidity (g/m³)
[1−Month Lag]
B2:Satun
Malaria
Absolute Humidity (g/m³)
[2−Month Lag]
C1:Satun
Malaria
Total Precipitation (mm)
[1−Month Lag]
C2:Satun
Malaria
Total Precipitation (mm)
[2−Month Lag]
D1:Satun
Malaria
Temperature (°C)
[1−Month Lag]
D2:Satun
Malaria
Temperature (°C)
[2−Month Lag]
E1:Satun
Malaria
SO_2 Surface Concentration (mg/m³)
[1−Month Lag]
E2:Satun
Malaria
SO_2 Surface Concentration (mg/m³)
[2−Month Lag]
F1:Satun
Malaria
CO Surface Concentration (ppb)
[1−Month Lag]
F2:Satun
Malaria
CO Surface Concentration (ppb)
[2−Month Lag]
G1:Satun
Malaria
$PM_{2.5}$ Surface Concentration (µg/m³)
[1−Month Lag]
G2:Satun
Malaria
$PM_{2.5}$ Surface Concentration (µg/m³)
[2−Month Lag]

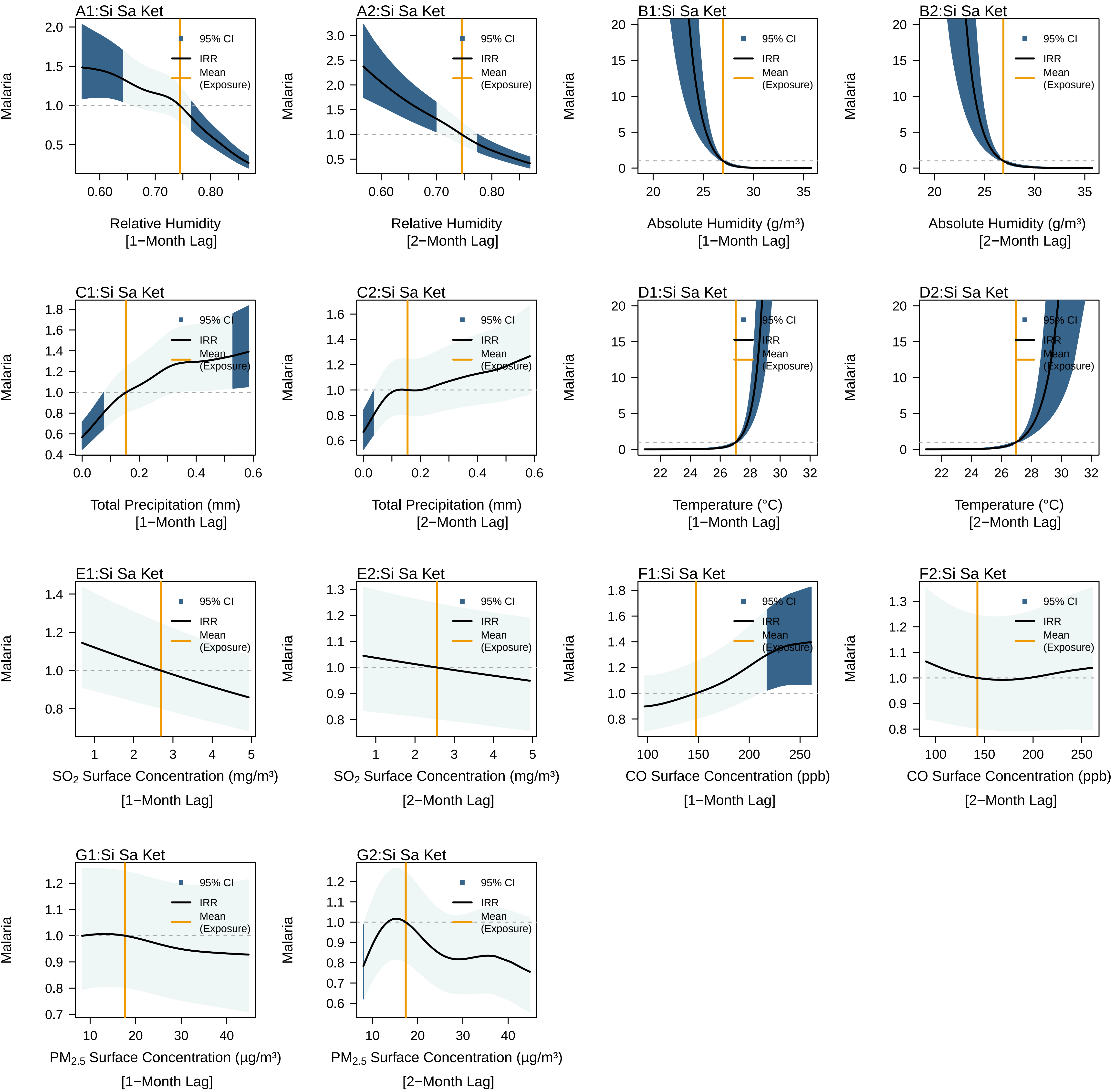
A1:Si Sa Ket
A2:Si Sa Ket
B1:Si Sa Ket
B2:Si Sa Ket
95% CI
IRR
Mean (Exposure)
Malaria
Relative Humidity [1−Month Lag]
Relative Humidity [2−Month Lag]
Absolute Humidity (g/m³) [1−Month Lag]
Absolute Humidity (g/m³) [2−Month Lag]
C1:Si Sa Ket
C2:Si Sa Ket
D1:Si Sa Ket
D2:Si Sa Ket
Total Precipitation (mm) [1−Month Lag]
Total Precipitation (mm) [2−Month Lag]
Temperature (°C) [1−Month Lag]
Temperature (°C) [2−Month Lag]
E1:Si Sa Ket
E2:Si Sa Ket
F1:Si Sa Ket
F2:Si Sa Ket
SO2 Surface Concentration (mg/m³) [1−Month Lag]
SO2 Surface Concentration (mg/m³) [2−Month Lag]
CO Surface Concentration (ppb) [1−Month Lag]
CO Surface Concentration (ppb) [2−Month Lag]
G1:Si Sa Ket
G2:Si Sa Ket
PM2.5 Surface Concentration (µg/m³) [1−Month Lag]
PM2.5 Surface Concentration (µg/m³) [2−Month Lag]

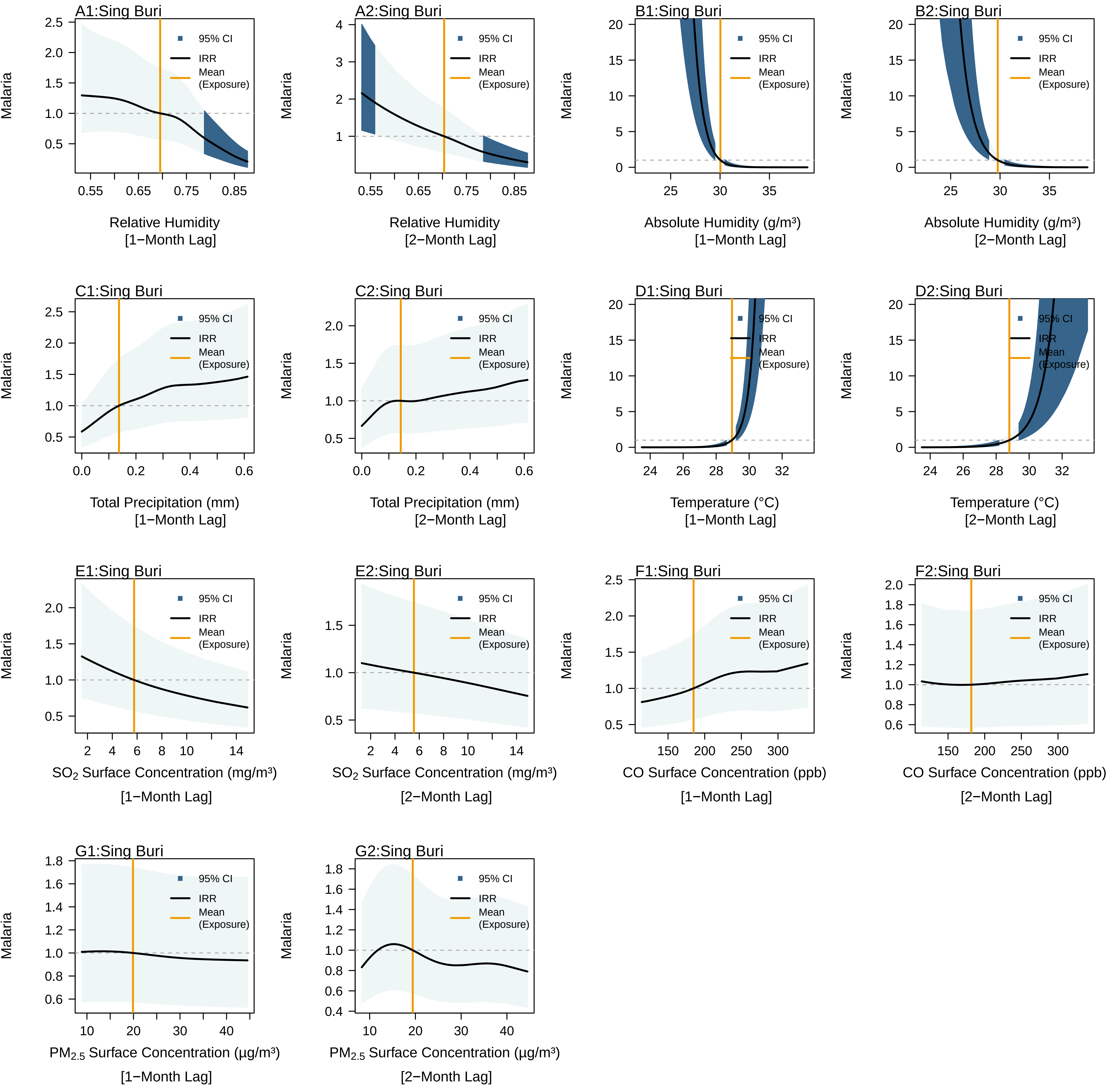
A1:Sing Buri
A2:Sing Buri
B1:Sing Buri
B2:Sing Buri
C1:Sing Buri
C2:Sing Buri
D1:Sing Buri
D2:Sing Buri
E1:Sing Buri
E2:Sing Buri
F1:Sing Buri
F2:Sing Buri
G1:Sing Buri
G2:Sing Buri
Malaria
95% CI
IRR
Mean (Exposure)
Relative Humidity [1−Month Lag]
Relative Humidity [2−Month Lag]
Absolute Humidity (g/m³) [1−Month Lag]
Absolute Humidity (g/m³) [2−Month Lag]
Total Precipitation (mm) [1−Month Lag]
Total Precipitation (mm) [2−Month Lag]
Temperature (°C) [1−Month Lag]
Temperature (°C) [2−Month Lag]
SO_2 Surface Concentration (mg/m³) [1−Month Lag]
SO_2 Surface Concentration (mg/m³) [2−Month Lag]
CO Surface Concentration (ppb) [1−Month Lag]
CO Surface Concentration (ppb) [2−Month Lag]
$PM_{2.5}$ Surface Concentration (μg/m³) [1−Month Lag]
$PM_{2.5}$ Surface Concentration (μg/m³) [2−Month Lag]

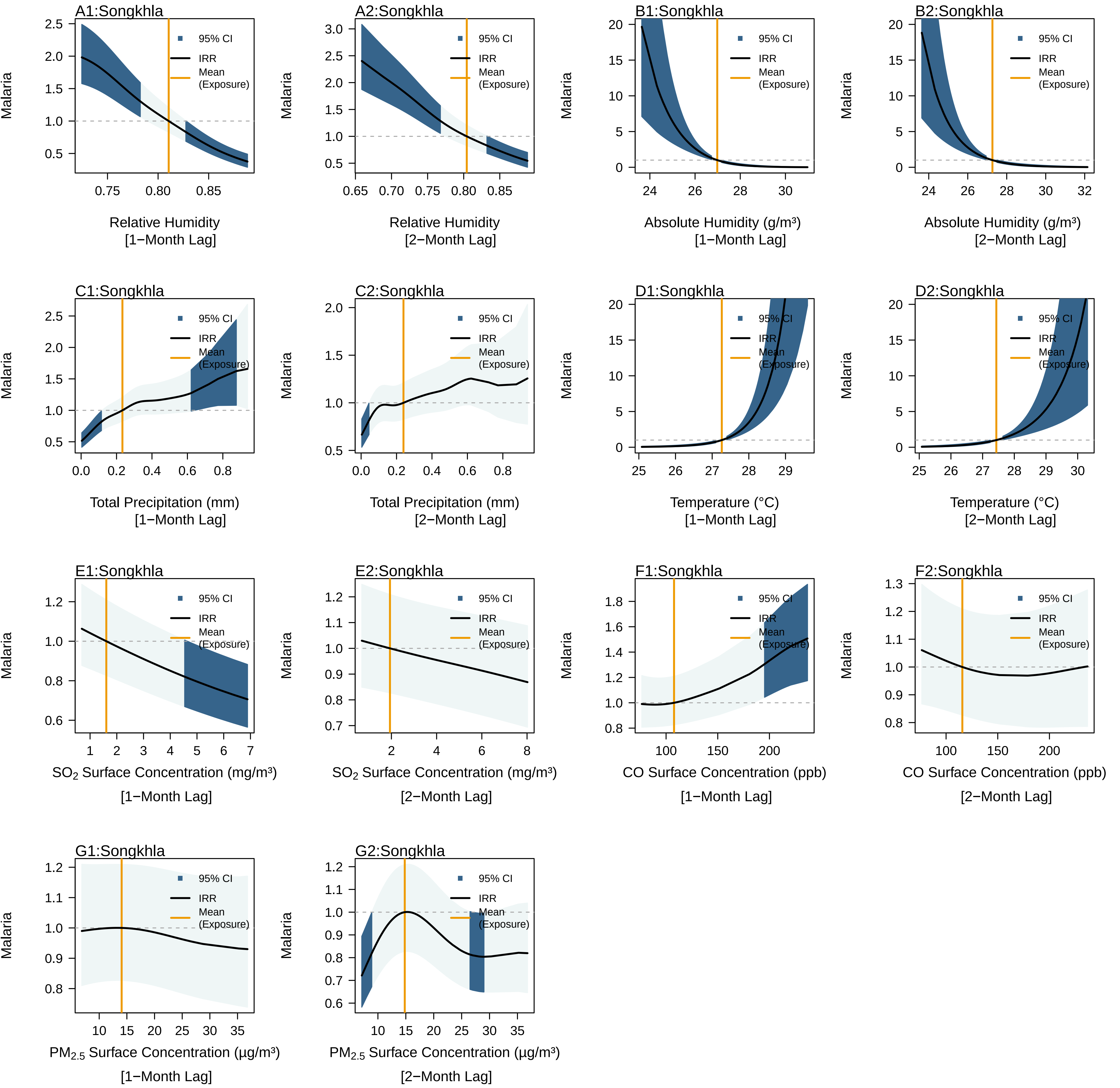

A1:Songkhla
A2:Songkhla
B1:Songkhla
B2:Songkhla
C1:Songkhla
C2:Songkhla
D1:Songkhla
D2:Songkhla
E1:Songkhla
E2:Songkhla
F1:Songkhla
F2:Songkhla
G1:Songkhla
G2:Songkhla
Malaria
95% CI
IRR
Mean (Exposure)
Relative Humidity [1−Month Lag]
Relative Humidity [2−Month Lag]
Absolute Humidity (g/m³) [1−Month Lag]
Absolute Humidity (g/m³) [2−Month Lag]
Total Precipitation (mm) [1−Month Lag]
Total Precipitation (mm) [2−Month Lag]
Temperature (°C) [1−Month Lag]
Temperature (°C) [2−Month Lag]
SO_2 Surface Concentration (mg/m³) [1−Month Lag]
SO_2 Surface Concentration (mg/m³) [2−Month Lag]
CO Surface Concentration (ppb) [1−Month Lag]
CO Surface Concentration (ppb) [2−Month Lag]
$PM_{2.5}$ Surface Concentration (µg/m³) [1−Month Lag]
$PM_{2.5}$ Surface Concentration (µg/m³) [2−Month Lag]

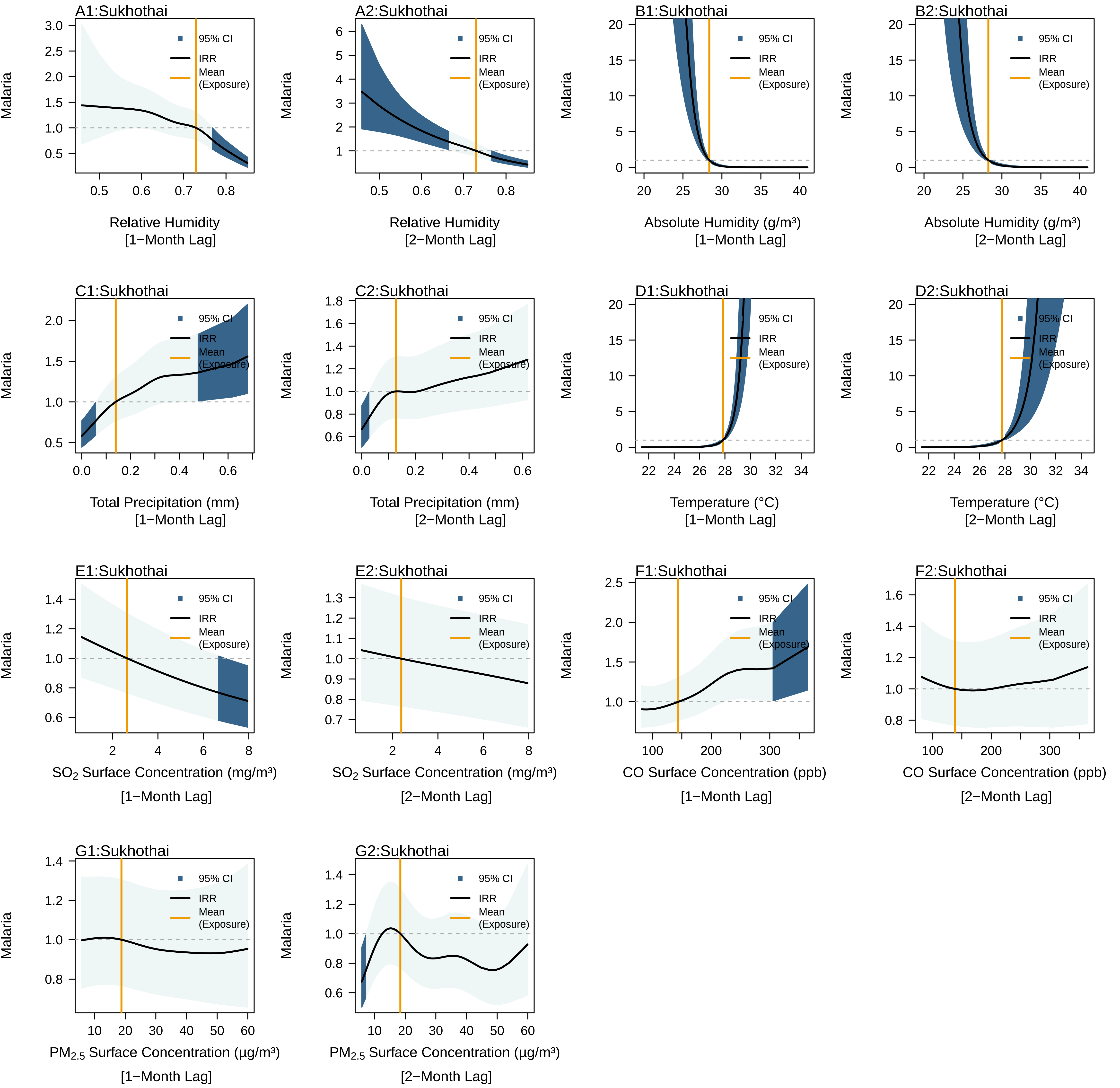
A1:Sukhothai
95% CI
IRR
Mean (Exposure)
Malaria
Relative Humidity [1−Month Lag]
A2:Sukhothai
Relative Humidity [2−Month Lag]
B1:Sukhothai
Absolute Humidity (g/m³) [1−Month Lag]
B2:Sukhothai
Absolute Humidity (g/m³) [2−Month Lag]
C1:Sukhothai
Total Precipitation (mm) [1−Month Lag]
C2:Sukhothai
Total Precipitation (mm) [2−Month Lag]
D1:Sukhothai
Temperature (°C) [1−Month Lag]
D2:Sukhothai
Temperature (°C) [2−Month Lag]
E1:Sukhothai
SO_2 Surface Concentration (mg/m³) [1−Month Lag]
E2:Sukhothai
SO_2 Surface Concentration (mg/m³) [2−Month Lag]
F1:Sukhothai
CO Surface Concentration (ppb) [1−Month Lag]
F2:Sukhothai
CO Surface Concentration (ppb) [2−Month Lag]
G1:Sukhothai
$PM_{2.5}$ Surface Concentration (µg/m³) [1−Month Lag]
G2:Sukhothai
$PM_{2.5}$ Surface Concentration (µg/m³) [2−Month Lag]

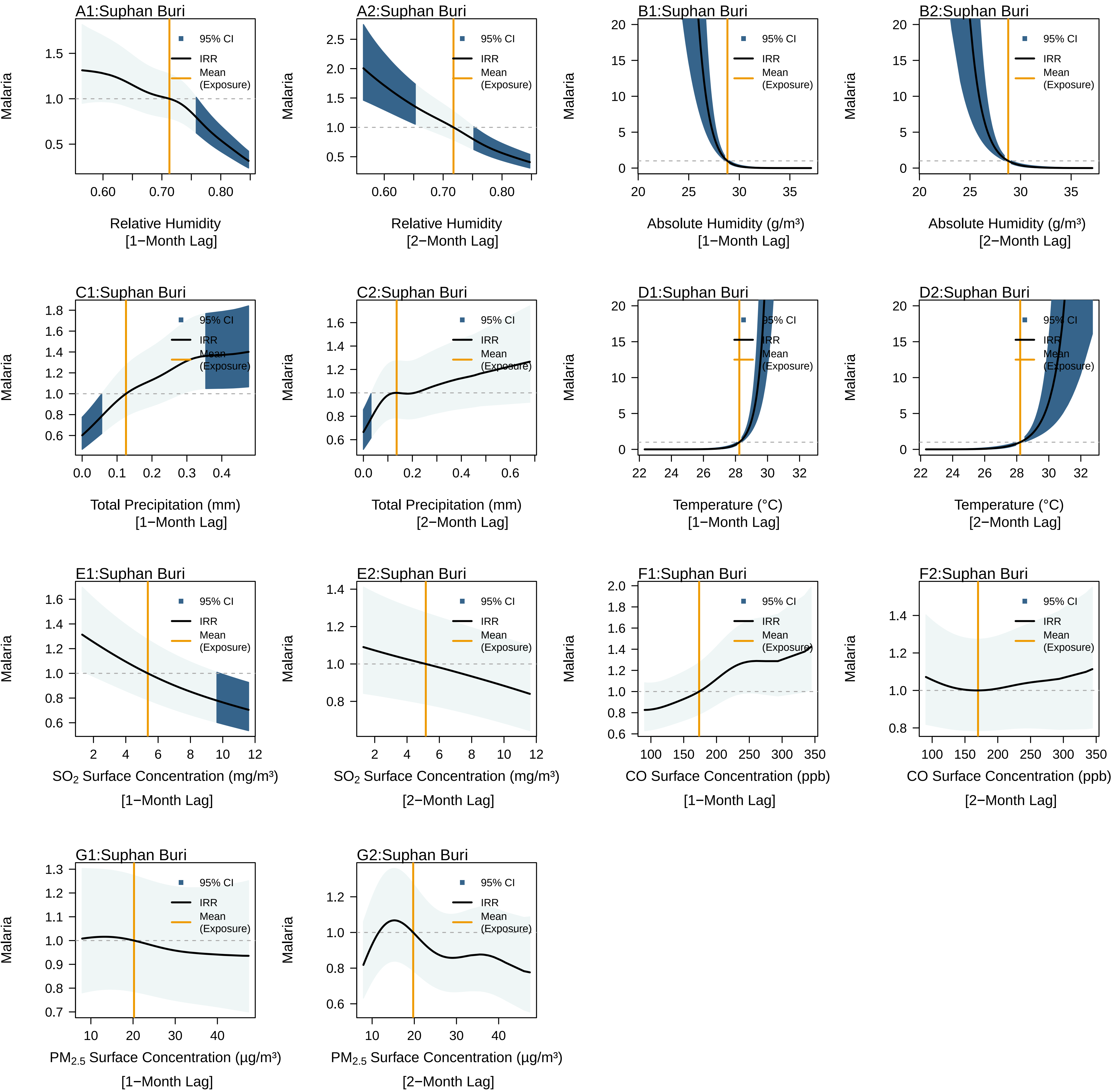
A1:Suphan Buri
Malaria
95% CI
IRR
Mean (Exposure)
Relative Humidity [1−Month Lag]
A2:Suphan Buri
Malaria
95% CI
IRR
Mean (Exposure)
Relative Humidity [2−Month Lag]
B1:Suphan Buri
Malaria
95% CI
IRR
Mean (Exposure)
Absolute Humidity (g/m³) [1−Month Lag]
B2:Suphan Buri
Malaria
95% CI
IRR
Mean (Exposure)
Absolute Humidity (g/m³) [2−Month Lag]
C1:Suphan Buri
Malaria
95% CI
IRR
Mean (Exposure)
Total Precipitation (mm) [1−Month Lag]
C2:Suphan Buri
Malaria
95% CI
IRR
Mean (Exposure)
Total Precipitation (mm) [2−Month Lag]
D1:Suphan Buri
Malaria
95% CI
IRR
Mean (Exposure)
Temperature (°C) [1−Month Lag]
D2:Suphan Buri
Malaria
95% CI
IRR
Mean (Exposure)
Temperature (°C) [2−Month Lag]
E1:Suphan Buri
Malaria
95% CI
IRR
Mean (Exposure)
SO_2 Surface Concentration (mg/m³) [1−Month Lag]
E2:Suphan Buri
Malaria
95% CI
IRR
Mean (Exposure)
SO_2 Surface Concentration (mg/m³) [2−Month Lag]
F1:Suphan Buri
Malaria
95% CI
IRR
Mean (Exposure)
CO Surface Concentration (ppb) [1−Month Lag]
F2:Suphan Buri
Malaria
95% CI
IRR
Mean (Exposure)
CO Surface Concentration (ppb) [2−Month Lag]
G1:Suphan Buri
Malaria
95% CI
IRR
Mean (Exposure)
$PM_{2.5}$ Surface Concentration (µg/m³) [1−Month Lag]
G2:Suphan Buri
Malaria
95% CI
IRR
Mean (Exposure)
$PM_{2.5}$ Surface Concentration (µg/m³) [2−Month Lag]

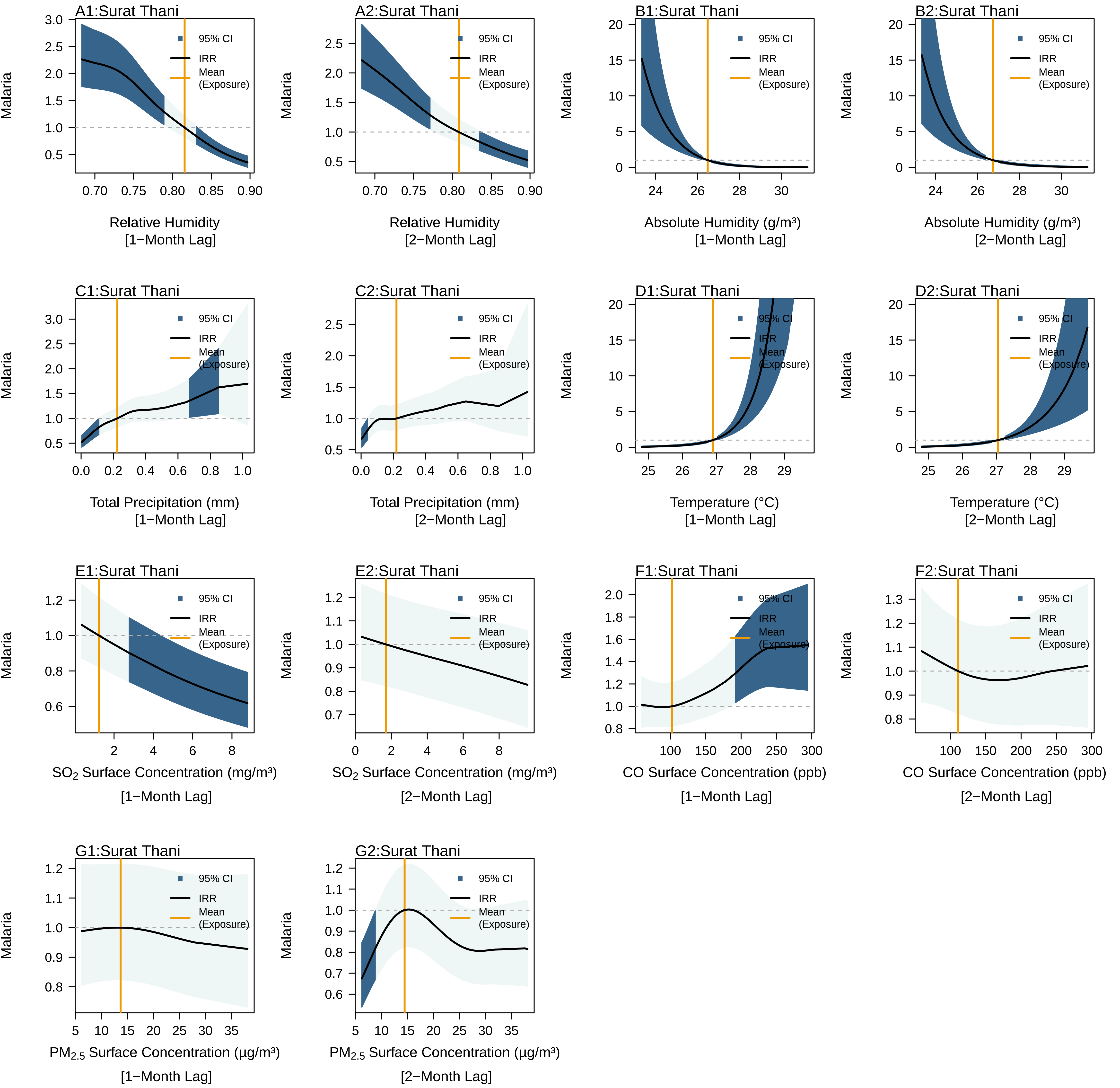
A1:Surat Thani
A2:Surat Thani
B1:Surat Thani
B2:Surat Thani
95% CI
IRR
Mean
(Exposure)
Malaria
Relative Humidity
[1−Month Lag]
Relative Humidity
[2−Month Lag]
Absolute Humidity (g/m³)
[1−Month Lag]
Absolute Humidity (g/m³)
[2−Month Lag]
C1:Surat Thani
C2:Surat Thani
D1:Surat Thani
D2:Surat Thani
Total Precipitation (mm)
[1−Month Lag]
Total Precipitation (mm)
[2−Month Lag]
Temperature (°C)
[1−Month Lag]
Temperature (°C)
[2−Month Lag]
E1:Surat Thani
E2:Surat Thani
F1:Surat Thani
F2:Surat Thani
SO_2 Surface Concentration (mg/m³)
[1−Month Lag]
SO_2 Surface Concentration (mg/m³)
[2−Month Lag]
CO Surface Concentration (ppb)
[1−Month Lag]
CO Surface Concentration (ppb)
[2−Month Lag]
G1:Surat Thani
G2:Surat Thani
$PM_{2.5}$ Surface Concentration (µg/m³)
[1−Month Lag]
$PM_{2.5}$ Surface Concentration (µg/m³)
[2−Month Lag]

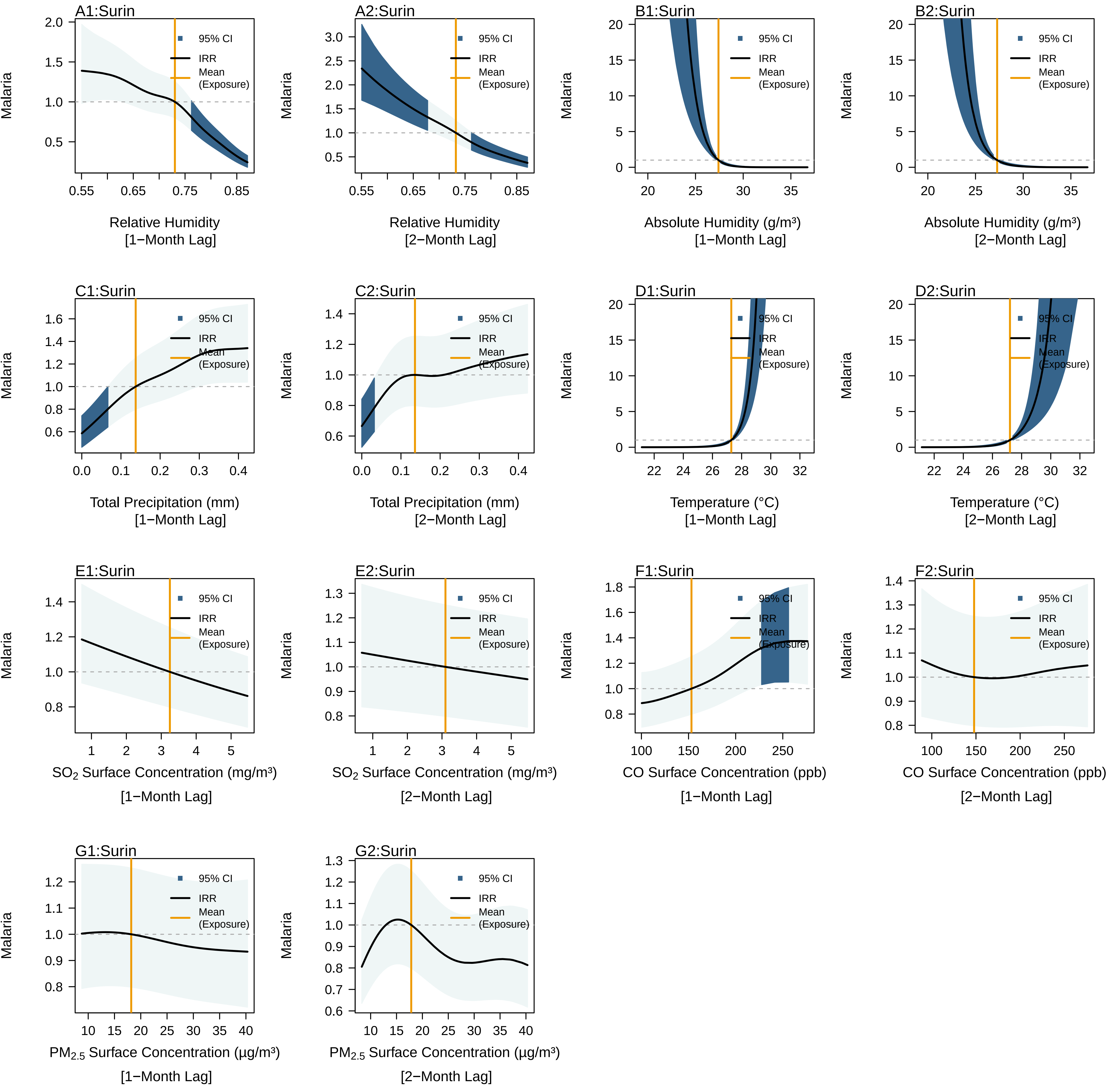
A1:Surin
Malaria
Relative Humidity
[1−Month Lag]
A2:Surin
Malaria
Relative Humidity
[2−Month Lag]
B1:Surin
Malaria
Absolute Humidity (g/m³)
[1−Month Lag]
B2:Surin
Malaria
Absolute Humidity (g/m³)
[2−Month Lag]
C1:Surin
Malaria
Total Precipitation (mm)
[1−Month Lag]
C2:Surin
Malaria
Total Precipitation (mm)
[2−Month Lag]
D1:Surin
Malaria
Temperature (°C)
[1−Month Lag]
D2:Surin
Malaria
Temperature (°C)
[2−Month Lag]
E1:Surin
Malaria
SO_2 Surface Concentration (mg/m³)
[1−Month Lag]
E2:Surin
Malaria
SO_2 Surface Concentration (mg/m³)
[2−Month Lag]
F1:Surin
Malaria
CO Surface Concentration (ppb)
[1−Month Lag]
F2:Surin
Malaria
CO Surface Concentration (ppb)
[2−Month Lag]
G1:Surin
Malaria
$PM_{2.5}$ Surface Concentration (μg/m³)
[1−Month Lag]
G2:Surin
Malaria
$PM_{2.5}$ Surface Concentration (μg/m³)
[2−Month Lag]
95% CI
IRR
Mean
(Exposure)

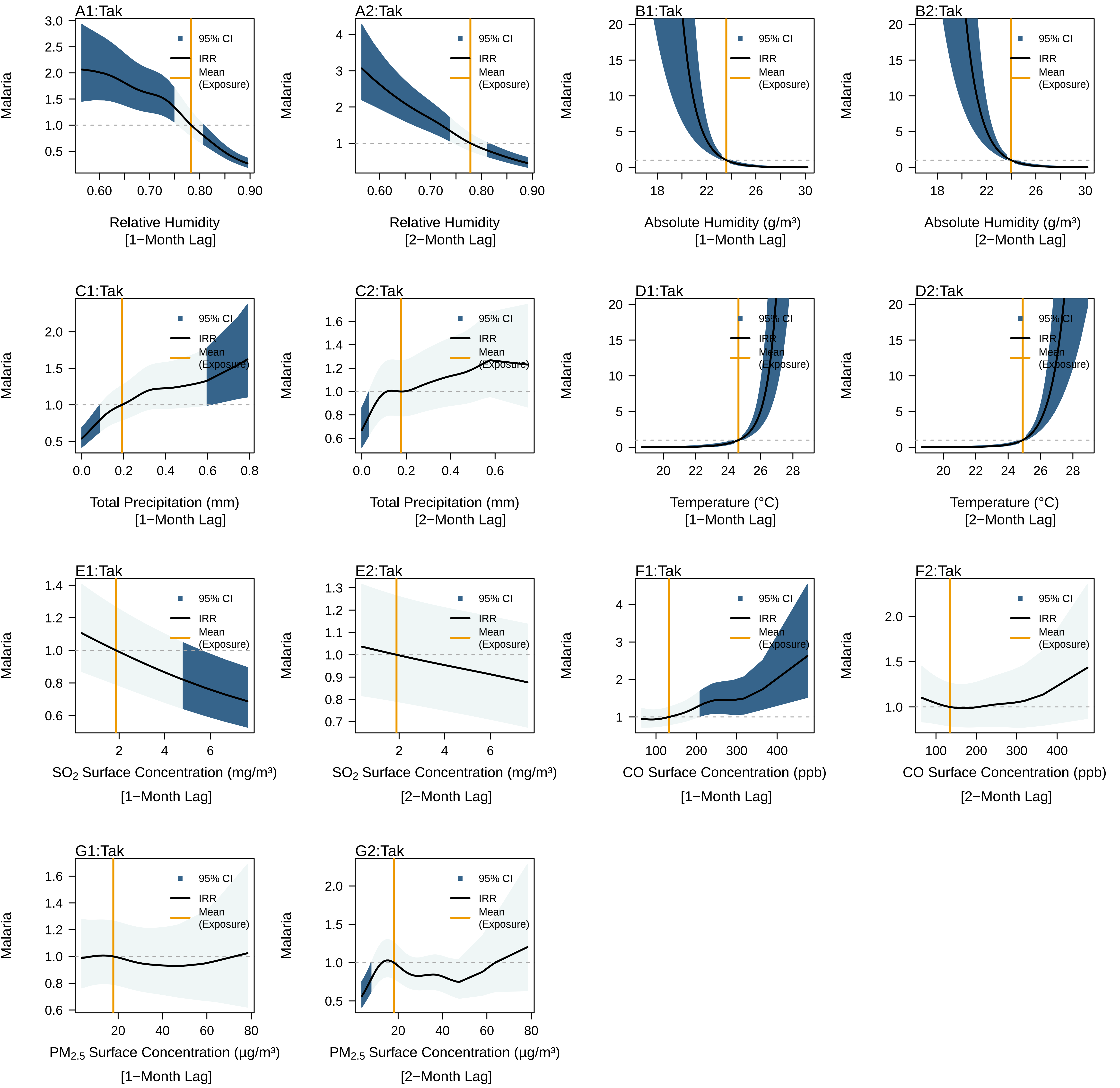

A1:Tak
A2:Tak
B1:Tak
B2:Tak
C1:Tak
C2:Tak
D1:Tak
D2:Tak
E1:Tak
E2:Tak
F1:Tak
F2:Tak
G1:Tak
G2:Tak
Malaria
95% CI
IRR
Mean (Exposure)
Relative Humidity [1−Month Lag]
Relative Humidity [2−Month Lag]
Absolute Humidity (g/m³) [1−Month Lag]
Absolute Humidity (g/m³) [2−Month Lag]
Total Precipitation (mm) [1−Month Lag]
Total Precipitation (mm) [2−Month Lag]
Temperature (°C) [1−Month Lag]
Temperature (°C) [2−Month Lag]
SO2 Surface Concentration (mg/m³) [1−Month Lag]
SO2 Surface Concentration (mg/m³) [2−Month Lag]
CO Surface Concentration (ppb) [1−Month Lag]
CO Surface Concentration (ppb) [2−Month Lag]
PM2.5 Surface Concentration (µg/m³) [1−Month Lag]
PM2.5 Surface Concentration (µg/m³) [2−Month Lag]

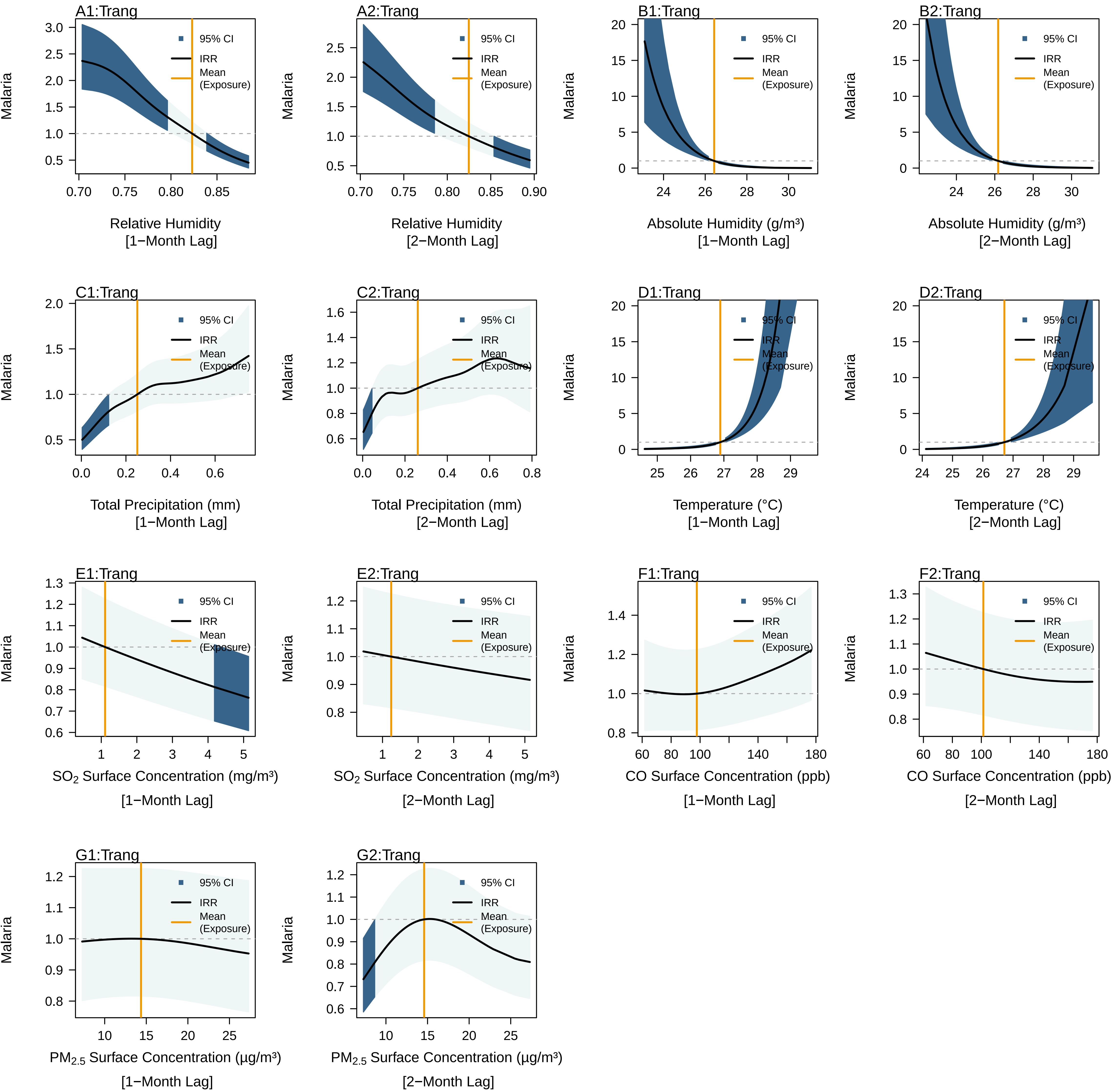
A1:Trang
95% CI
IRR
Mean (Exposure)
Malaria
3.0 2.5 2.0 1.5 1.0 0.5
0.70 0.75 0.80 0.85
Relative Humidity [1−Month Lag]
A2:Trang
95% CI
IRR
Mean (Exposure)
Malaria
2.5 2.0 1.5 1.0 0.5
0.70 0.75 0.80 0.85 0.90
Relative Humidity [2−Month Lag]
B1:Trang
95% CI
IRR
Mean (Exposure)
Malaria
20 15 10 5 0
24 26 28 30
Absolute Humidity (g/m³) [1−Month Lag]
B2:Trang
95% CI
IRR
Mean (Exposure)
Malaria
20 15 10 5 0
24 26 28 30
Absolute Humidity (g/m³) [2−Month Lag]
C1:Trang
95% CI
IRR
Mean (Exposure)
Malaria
2.0 1.5 1.0 0.5
0.0 0.2 0.4 0.6
Total Precipitation (mm) [1−Month Lag]
C2:Trang
95% CI
IRR
Mean (Exposure)
Malaria
1.6 1.4 1.2 1.0 0.8 0.6
0.0 0.2 0.4 0.6 0.8
Total Precipitation (mm) [2−Month Lag]
D1:Trang
95% CI
IRR
Mean (Exposure)
Malaria
20 15 10 5 0
25 26 27 28 29
Temperature (°C) [1−Month Lag]
D2:Trang
95% CI
IRR
Mean (Exposure)
Malaria
20 15 10 5 0
24 25 26 27 28 29
Temperature (°C) [2−Month Lag]
E1:Trang
95% CI
IRR
Mean (Exposure)
Malaria
1.3 1.2 1.1 1.0 0.9 0.8 0.7 0.6
1 2 3 4 5
SO2 Surface Concentration (mg/m³) [1−Month Lag]
E2:Trang
95% CI
IRR
Mean (Exposure)
Malaria
1.2 1.1 1.0 0.9 0.8
1 2 3 4 5
SO2 Surface Concentration (mg/m³) [2−Month Lag]
F1:Trang
95% CI
IRR
Mean (Exposure)
Malaria
1.4 1.2 1.0 0.8
60 80 100 140 180
CO Surface Concentration (ppb) [1−Month Lag]
F2:Trang
95% CI
IRR
Mean (Exposure)
Malaria
1.3 1.2 1.1 1.0 0.9 0.8
60 80 100 140 180
CO Surface Concentration (ppb) [2−Month Lag]
G1:Trang
95% CI
IRR
Mean (Exposure)
Malaria
1.2 1.1 1.0 0.9 0.8
10 15 20 25
PM2.5 Surface Concentration (µg/m³) [1−Month Lag]
G2:Trang
95% CI
IRR
Mean (Exposure)
Malaria
1.2 1.1 1.0 0.9 0.8 0.7 0.6
10 15 20 25
PM2.5 Surface Concentration (µg/m³) [2−Month Lag]

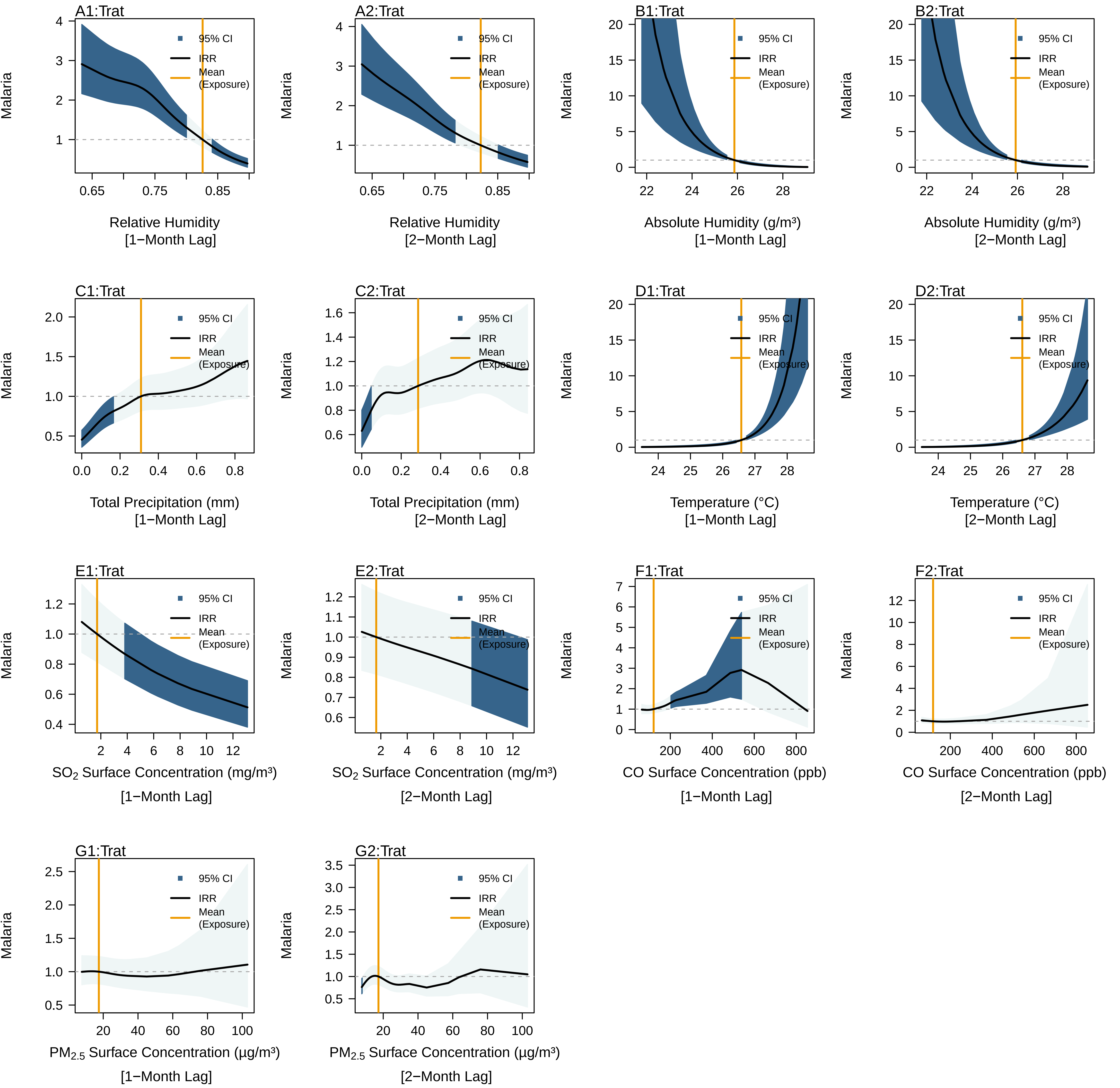

A1:Trat
95% CI
IRR
Mean (Exposure)
Malaria
Relative Humidity [1−Month Lag]
A2:Trat
Relative Humidity [2−Month Lag]
B1:Trat
Absolute Humidity (g/m³) [1−Month Lag]
B2:Trat
Absolute Humidity (g/m³) [2−Month Lag]
C1:Trat
Total Precipitation (mm) [1−Month Lag]
C2:Trat
Total Precipitation (mm) [2−Month Lag]
D1:Trat
Temperature (°C) [1−Month Lag]
D2:Trat
Temperature (°C) [2−Month Lag]
E1:Trat
SO_2 Surface Concentration (mg/m³) [1−Month Lag]
E2:Trat
SO_2 Surface Concentration (mg/m³) [2−Month Lag]
F1:Trat
CO Surface Concentration (ppb) [1−Month Lag]
F2:Trat
CO Surface Concentration (ppb) [2−Month Lag]
G1:Trat
$PM_{2.5}$ Surface Concentration (µg/m³) [1−Month Lag]
G2:Trat
$PM_{2.5}$ Surface Concentration (µg/m³) [2−Month Lag]

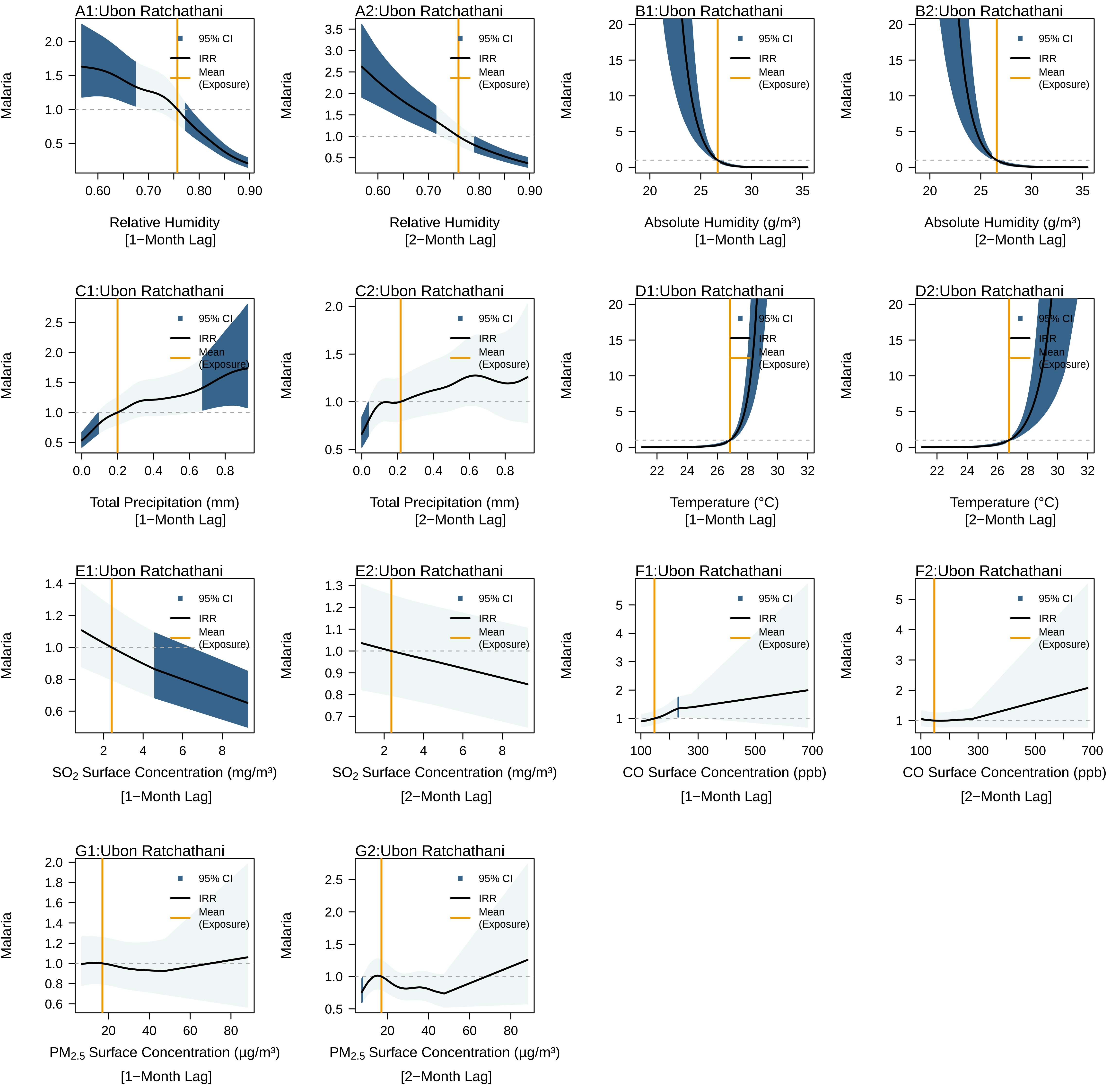

A1:Ubon Ratchathani
Malaria
95% CI
IRR
Mean (Exposure)
0.60 0.70 0.80 0.90
0.5 1.0 1.5 2.0
Relative Humidity [1−Month Lag]
A2:Ubon Ratchathani
Malaria
95% CI
IRR
Mean (Exposure)
0.60 0.70 0.80 0.90
0.5 1.0 1.5 2.0 2.5 3.0 3.5
Relative Humidity [2−Month Lag]
B1:Ubon Ratchathani
Malaria
95% CI
IRR
Mean (Exposure)
20 25 30 35
0 5 10 15 20
Absolute Humidity (g/m³) [1−Month Lag]
B2:Ubon Ratchathani
Malaria
95% CI
IRR
Mean (Exposure)
20 25 30 35
0 5 10 15 20
Absolute Humidity (g/m³) [2−Month Lag]
C1:Ubon Ratchathani
Malaria
95% CI
IRR
Mean (Exposure)
0.0 0.2 0.4 0.6 0.8
0.5 1.0 1.5 2.0 2.5
Total Precipitation (mm) [1−Month Lag]
C2:Ubon Ratchathani
Malaria
95% CI
IRR
Mean (Exposure)
0.0 0.2 0.4 0.6 0.8
0.5 1.0 1.5 2.0
Total Precipitation (mm) [2−Month Lag]
D1:Ubon Ratchathani
Malaria
95% CI
IRR
Mean (Exposure)
22 24 26 28 30 32
0 5 10 15 20
Temperature (°C) [1−Month Lag]
D2:Ubon Ratchathani
Malaria
95% CI
IRR
Mean (Exposure)
22 24 26 28 30 32
0 5 10 15 20
Temperature (°C) [2−Month Lag]
E1:Ubon Ratchathani
Malaria
95% CI
IRR
Mean (Exposure)
2 4 6 8
0.6 0.8 1.0 1.2 1.4
SO2 Surface Concentration (mg/m³) [1−Month Lag]
E2:Ubon Ratchathani
Malaria
95% CI
IRR
Mean (Exposure)
2 4 6 8
0.7 0.8 0.9 1.0 1.1 1.2 1.3
SO2 Surface Concentration (mg/m³) [2−Month Lag]
F1:Ubon Ratchathani
Malaria
95% CI
IRR
Mean (Exposure)
100 300 500 700
1 2 3 4 5
CO Surface Concentration (ppb) [1−Month Lag]
F2:Ubon Ratchathani
Malaria
95% CI
IRR
Mean (Exposure)
100 300 500 700
1 2 3 4 5
CO Surface Concentration (ppb) [2−Month Lag]
G1:Ubon Ratchathani
Malaria
95% CI
IRR
Mean (Exposure)
20 40 60 80
0.6 0.8 1.0 1.2 1.4 1.6 1.8 2.0
PM2.5 Surface Concentration (µg/m³) [1−Month Lag]
G2:Ubon Ratchathani
Malaria
95% CI
IRR
Mean (Exposure)
20 40 60 80
0.5 1.0 1.5 2.0 2.5
PM2.5 Surface Concentration (µg/m³) [2−Month Lag]

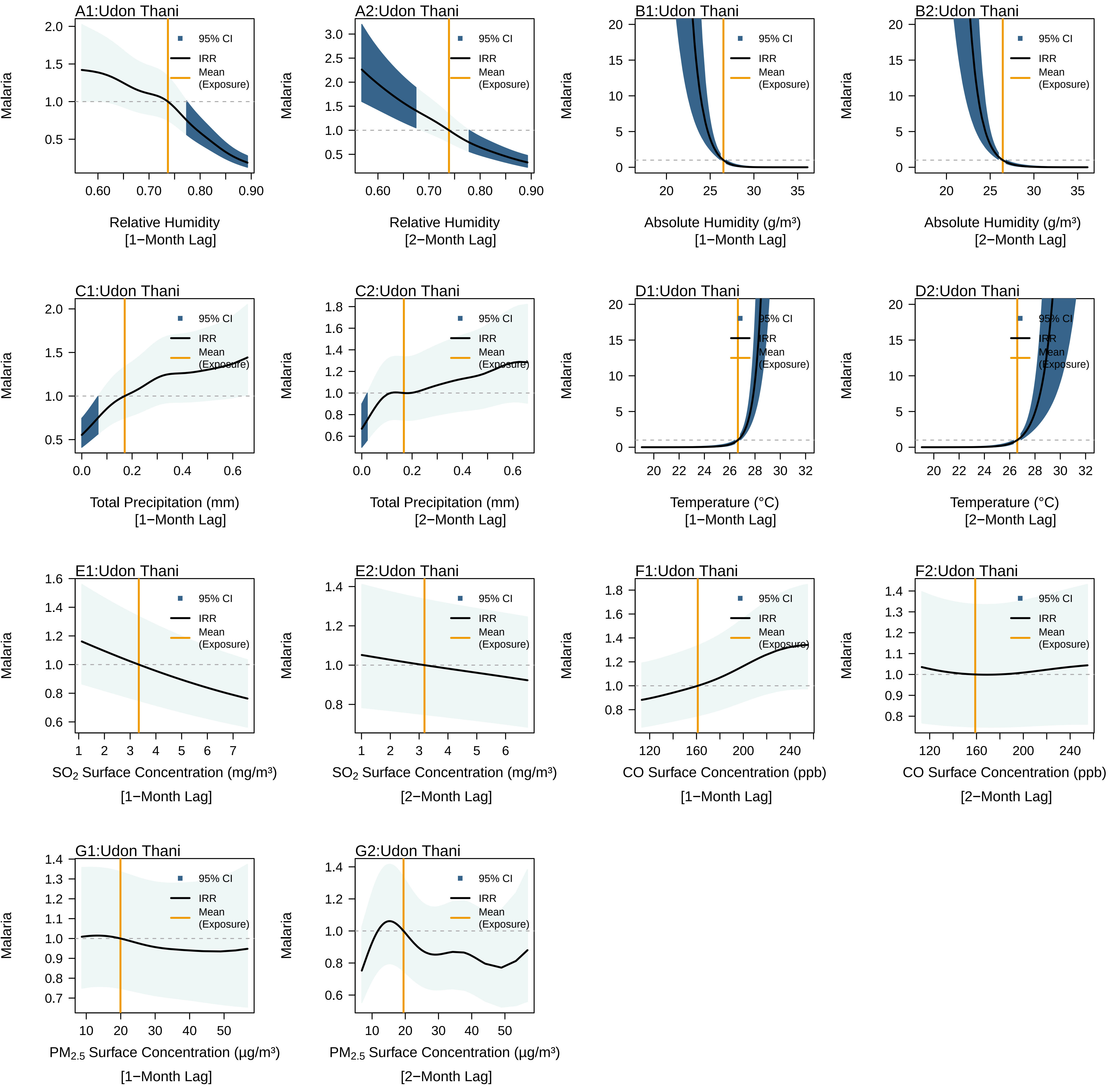
A1:Udon Thani
A2:Udon Thani
B1:Udon Thani
B2:Udon Thani
C1:Udon Thani
C2:Udon Thani
D1:Udon Thani
D2:Udon Thani
E1:Udon Thani
E2:Udon Thani
F1:Udon Thani
F2:Udon Thani
G1:Udon Thani
G2:Udon Thani
Malaria
95% CI
IRR
Mean (Exposure)
Relative Humidity [1−Month Lag]
Relative Humidity [2−Month Lag]
Absolute Humidity (g/m³) [1−Month Lag]
Absolute Humidity (g/m³) [2−Month Lag]
Total Precipitation (mm) [1−Month Lag]
Total Precipitation (mm) [2−Month Lag]
Temperature (°C) [1−Month Lag]
Temperature (°C) [2−Month Lag]
SO2 Surface Concentration (mg/m³) [1−Month Lag]
SO2 Surface Concentration (mg/m³) [2−Month Lag]
CO Surface Concentration (ppb) [1−Month Lag]
CO Surface Concentration (ppb) [2−Month Lag]
PM2.5 Surface Concentration (µg/m³) [1−Month Lag]
PM2.5 Surface Concentration (µg/m³) [2−Month Lag]

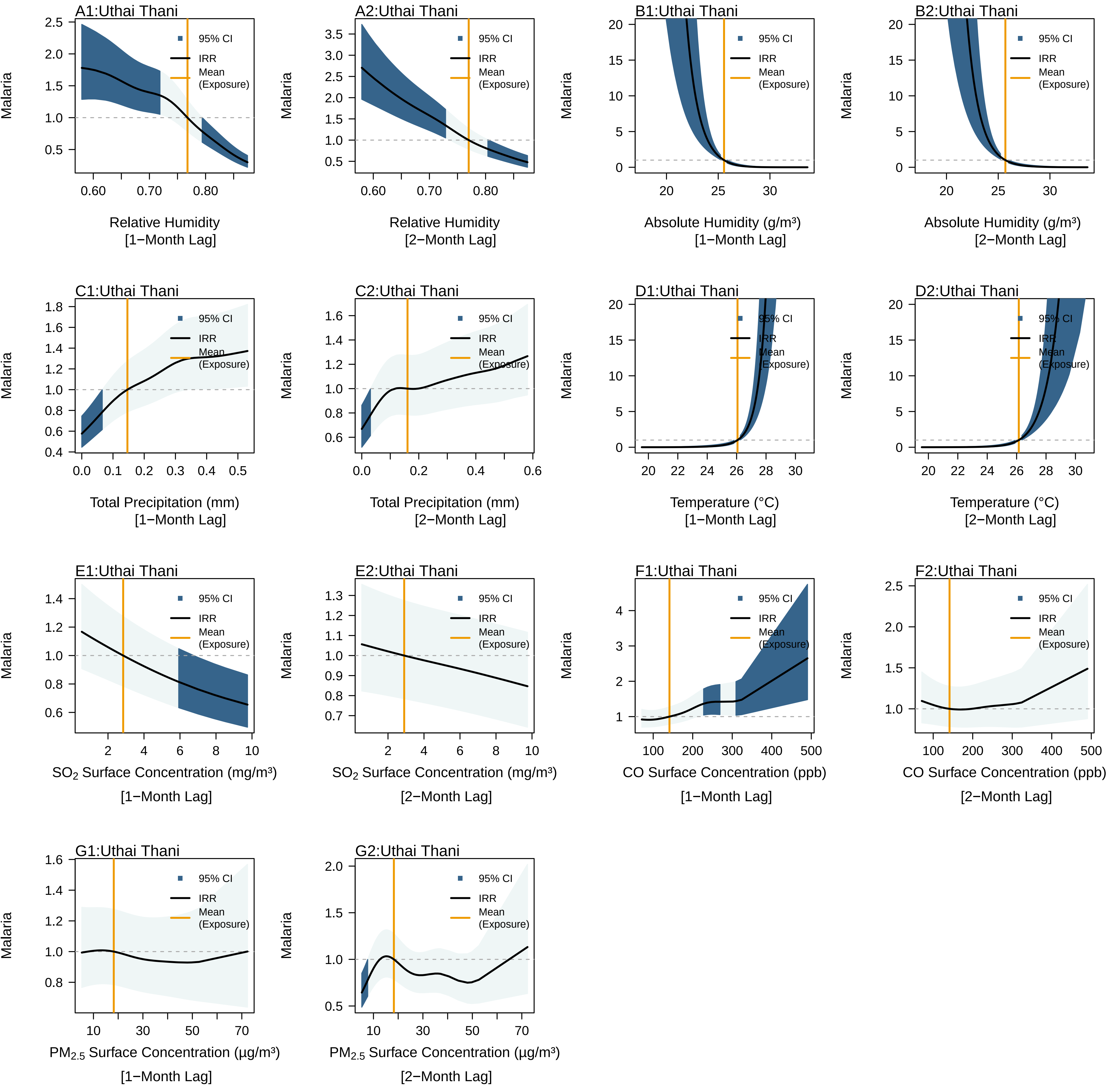
A1:Uthai Thani
Malaria
95% CI
IRR
Mean (Exposure)
Relative Humidity [1−Month Lag]
A2:Uthai Thani
Malaria
95% CI
IRR
Mean (Exposure)
Relative Humidity [2−Month Lag]
B1:Uthai Thani
Malaria
95% CI
IRR
Mean (Exposure)
Absolute Humidity (g/m³) [1−Month Lag]
B2:Uthai Thani
Malaria
95% CI
IRR
Mean (Exposure)
Absolute Humidity (g/m³) [2−Month Lag]
C1:Uthai Thani
Malaria
95% CI
IRR
Mean (Exposure)
Total Precipitation (mm) [1−Month Lag]
C2:Uthai Thani
Malaria
95% CI
IRR
Mean (Exposure)
Total Precipitation (mm) [2−Month Lag]
D1:Uthai Thani
Malaria
95% CI
IRR
Mean (Exposure)
Temperature (°C) [1−Month Lag]
D2:Uthai Thani
Malaria
95% CI
IRR
Mean (Exposure)
Temperature (°C) [2−Month Lag]
E1:Uthai Thani
Malaria
95% CI
IRR
Mean (Exposure)
SO₂ Surface Concentration (mg/m³) [1−Month Lag]
E2:Uthai Thani
Malaria
95% CI
IRR
Mean (Exposure)
SO₂ Surface Concentration (mg/m³) [2−Month Lag]
F1:Uthai Thani
Malaria
95% CI
IRR
Mean (Exposure)
CO Surface Concentration (ppb) [1−Month Lag]
F2:Uthai Thani
Malaria
95% CI
IRR
Mean (Exposure)
CO Surface Concentration (ppb) [2−Month Lag]
G1:Uthai Thani
Malaria
95% CI
IRR
Mean (Exposure)
PM2.5 Surface Concentration (µg/m³) [1−Month Lag]
G2:Uthai Thani
Malaria
95% CI
IRR
Mean (Exposure)
PM2.5 Surface Concentration (µg/m³) [2−Month Lag]

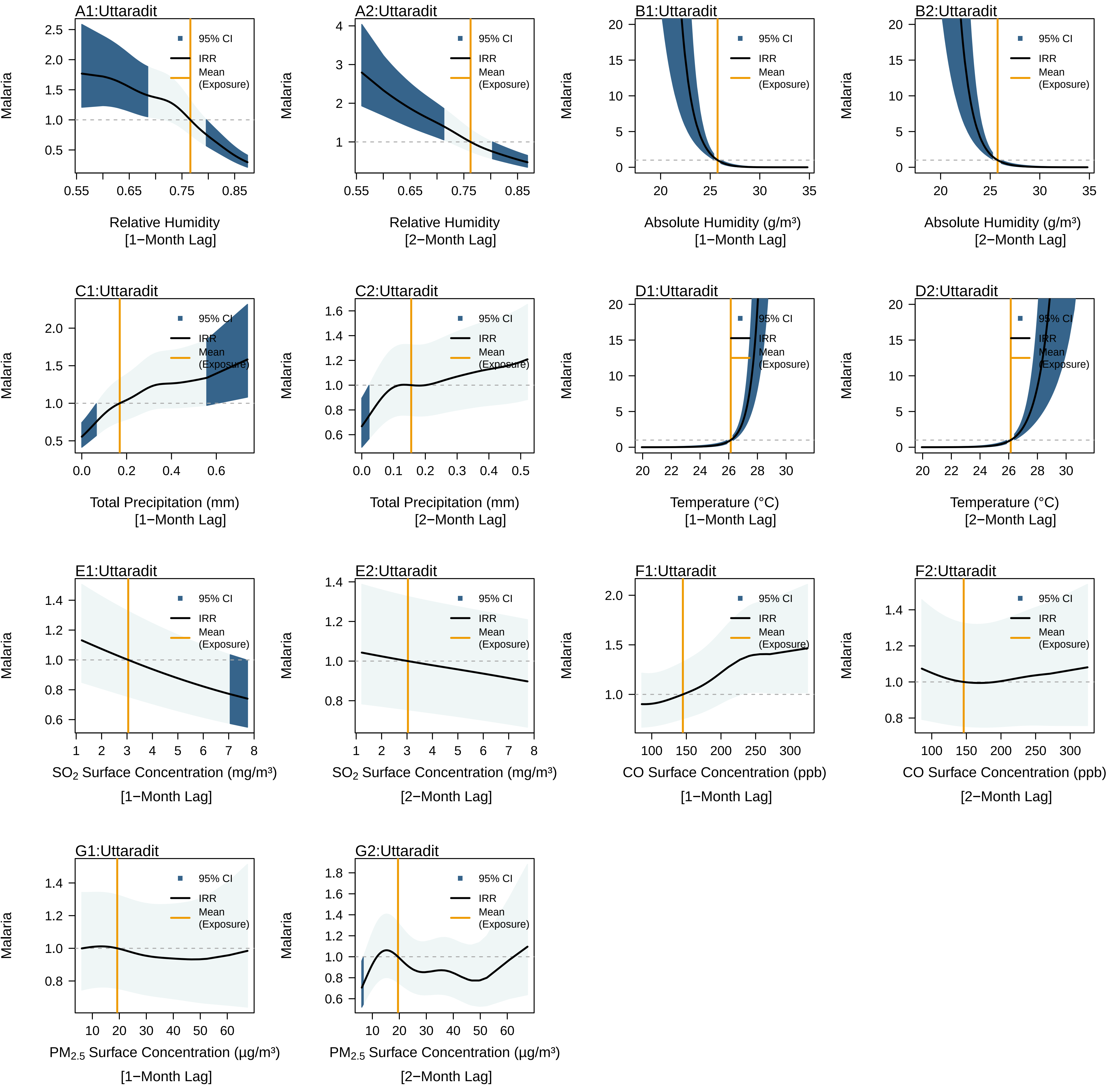
A1:Uttaradit
95% CI
IRR
Mean (Exposure)
Malaria
2.5 2.0 1.5 1.0 0.5
0.55 0.65 0.75 0.85
Relative Humidity [1−Month Lag]
A2:Uttaradit
95% CI
IRR
Mean (Exposure)
Malaria
4 3 2 1
0.55 0.65 0.75 0.85
Relative Humidity [2−Month Lag]
B1:Uttaradit
95% CI
IRR
Mean (Exposure)
Malaria
20 15 10 5 0
20 25 30 35
Absolute Humidity (g/m³) [1−Month Lag]
B2:Uttaradit
95% CI
IRR
Mean (Exposure)
Malaria
20 15 10 5 0
20 25 30 35
Absolute Humidity (g/m³) [2−Month Lag]
C1:Uttaradit
95% CI
IRR
Mean (Exposure)
Malaria
2.0 1.5 1.0 0.5
0.0 0.2 0.4 0.6
Total Precipitation (mm) [1−Month Lag]
C2:Uttaradit
95% CI
IRR
Mean (Exposure)
Malaria
1.6 1.4 1.2 1.0 0.8 0.6
0.0 0.1 0.2 0.3 0.4 0.5
Total Precipitation (mm) [2−Month Lag]
D1:Uttaradit
95% CI
IRR
Mean (Exposure)
Malaria
20 15 10 5 0
20 22 24 26 28 30
Temperature (°C) [1−Month Lag]
D2:Uttaradit
95% CI
IRR
Mean (Exposure)
Malaria
20 15 10 5 0
20 22 24 26 28 30
Temperature (°C) [2−Month Lag]
E1:Uttaradit
95% CI
IRR
Mean (Exposure)
Malaria
1.4 1.2 1.0 0.8 0.6
1 2 3 4 5 6 7 8
SO_2 Surface Concentration (mg/m³) [1−Month Lag]
E2:Uttaradit
95% CI
IRR
Mean (Exposure)
Malaria
1.4 1.2 1.0 0.8
1 2 3 4 5 6 7 8
SO_2 Surface Concentration (mg/m³) [2−Month Lag]
F1:Uttaradit
95% CI
IRR
Mean (Exposure)
Malaria
2.0 1.5 1.0
100 150 200 250 300
CO Surface Concentration (ppb) [1−Month Lag]
F2:Uttaradit
95% CI
IRR
Mean (Exposure)
Malaria
1.4 1.2 1.0 0.8
100 150 200 250 300
CO Surface Concentration (ppb) [2−Month Lag]
G1:Uttaradit
95% CI
IRR
Mean (Exposure)
Malaria
1.4 1.2 1.0 0.8
10 20 30 40 50 60
$PM_{2.5}$ Surface Concentration (µg/m³) [1−Month Lag]
G2:Uttaradit
95% CI
IRR
Mean (Exposure)
Malaria
1.8 1.6 1.4 1.2 1.0 0.8 0.6
10 20 30 40 50 60
$PM_{2.5}$ Surface Concentration (µg/m³) [2−Month Lag]

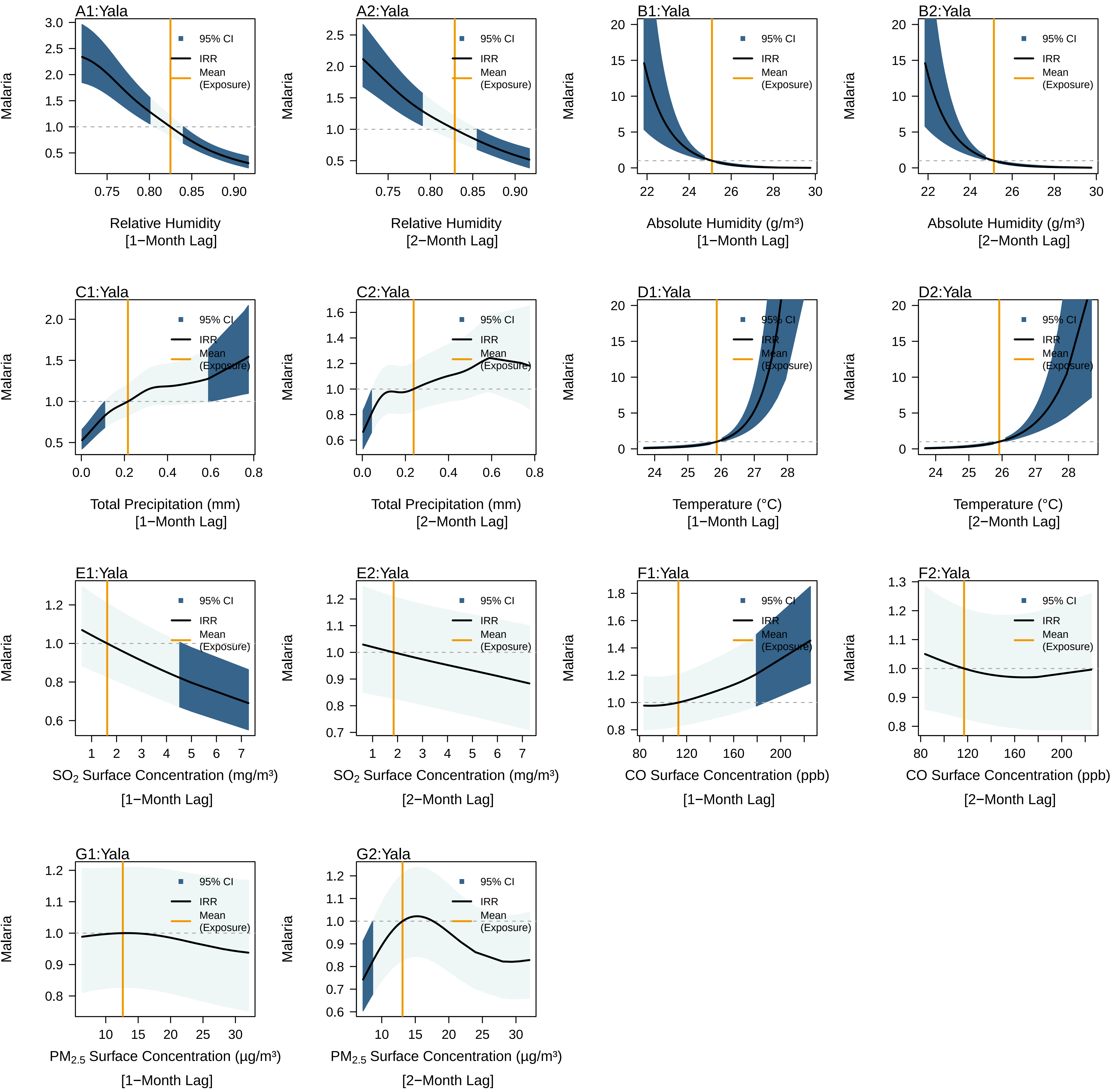
A1:Yala
A2:Yala
B1:Yala
B2:Yala
C1:Yala
C2:Yala
D1:Yala
D2:Yala
E1:Yala
E2:Yala
F1:Yala
F2:Yala
G1:Yala
G2:Yala
Malaria
95% CI
IRR
Mean
(Exposure)
Relative Humidity
[1−Month Lag]
[2−Month Lag]
Absolute Humidity (g/m³)
Total Precipitation (mm)
Temperature (°C)
SO_2 Surface Concentration (mg/m³)
CO Surface Concentration (ppb)
$PM_{2.5}$ Surface Concentration (μg/m³)

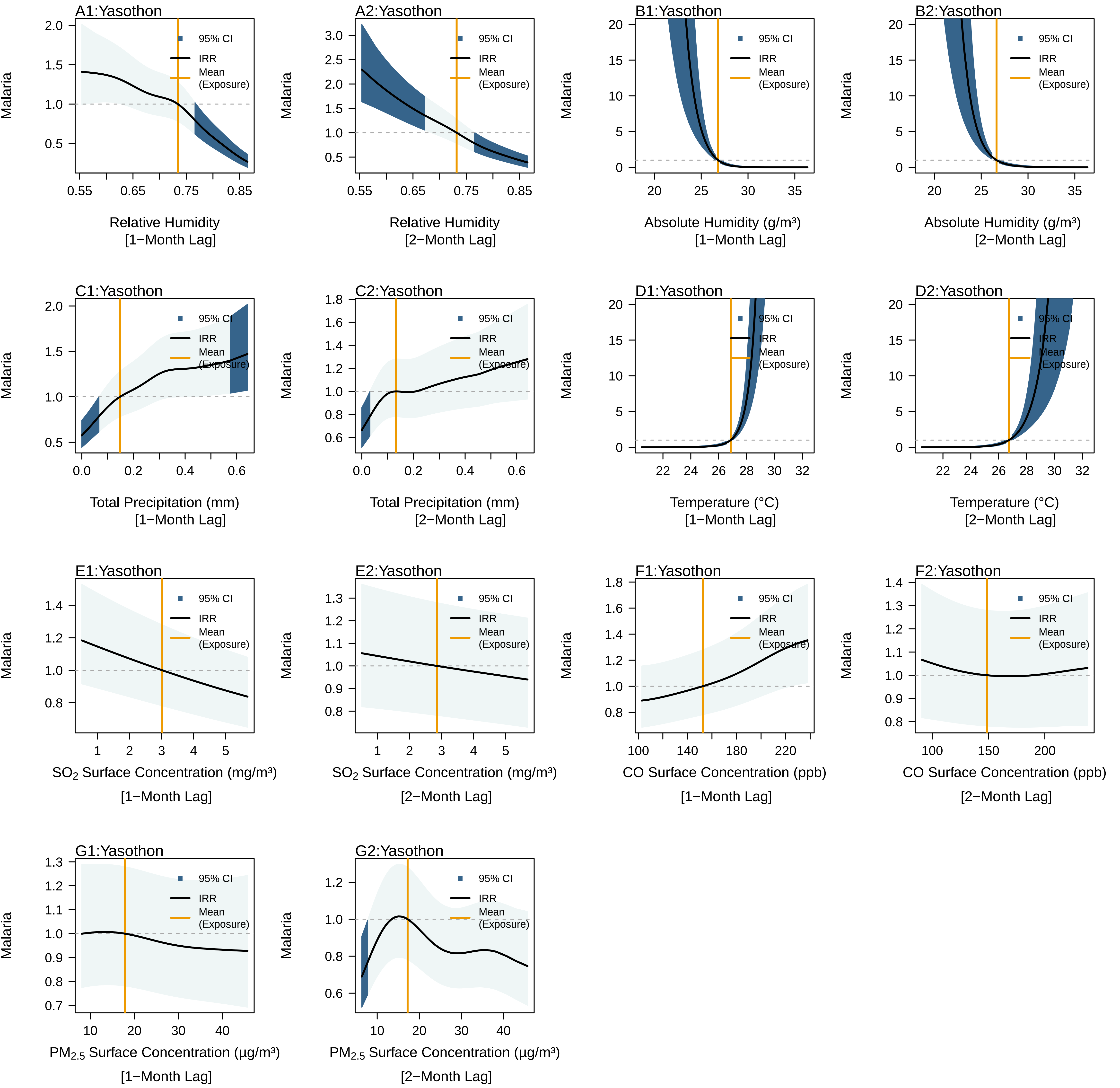

A1:Yasothon
95% CI
IRR
Mean (Exposure)
Malaria
Relative Humidity [1−Month Lag]
A2:Yasothon
95% CI
IRR
Mean (Exposure)
Malaria
Relative Humidity [2−Month Lag]
B1:Yasothon
95% CI
IRR
Mean (Exposure)
Malaria
Absolute Humidity (g/m³) [1−Month Lag]
B2:Yasothon
95% CI
IRR
Mean (Exposure)
Malaria
Absolute Humidity (g/m³) [2−Month Lag]
C1:Yasothon
95% CI
IRR
Mean (Exposure)
Malaria
Total Precipitation (mm) [1−Month Lag]
C2:Yasothon
95% CI
IRR
Mean (Exposure)
Malaria
Total Precipitation (mm) [2−Month Lag]
D1:Yasothon
95% CI
IRR
Mean (Exposure)
Malaria
Temperature (°C) [1−Month Lag]
D2:Yasothon
95% CI
IRR
Mean (Exposure)
Malaria
Temperature (°C) [2−Month Lag]
E1:Yasothon
95% CI
IRR
Mean (Exposure)
Malaria
SO2 Surface Concentration (mg/m³) [1−Month Lag]
E2:Yasothon
95% CI
IRR
Mean (Exposure)
Malaria
SO2 Surface Concentration (mg/m³) [2−Month Lag]
F1:Yasothon
95% CI
IRR
Mean (Exposure)
Malaria
CO Surface Concentration (ppb) [1−Month Lag]
F2:Yasothon
95% CI
IRR
Mean (Exposure)
Malaria
CO Surface Concentration (ppb) [2−Month Lag]
G1:Yasothon
95% CI
IRR
Mean (Exposure)
Malaria
PM2.5 Surface Concentration (µg/m³) [1−Month Lag]
G2:Yasothon
95% CI
IRR
Mean (Exposure)
Malaria
PM2.5 Surface Concentration (µg/m³) [2−Month Lag]

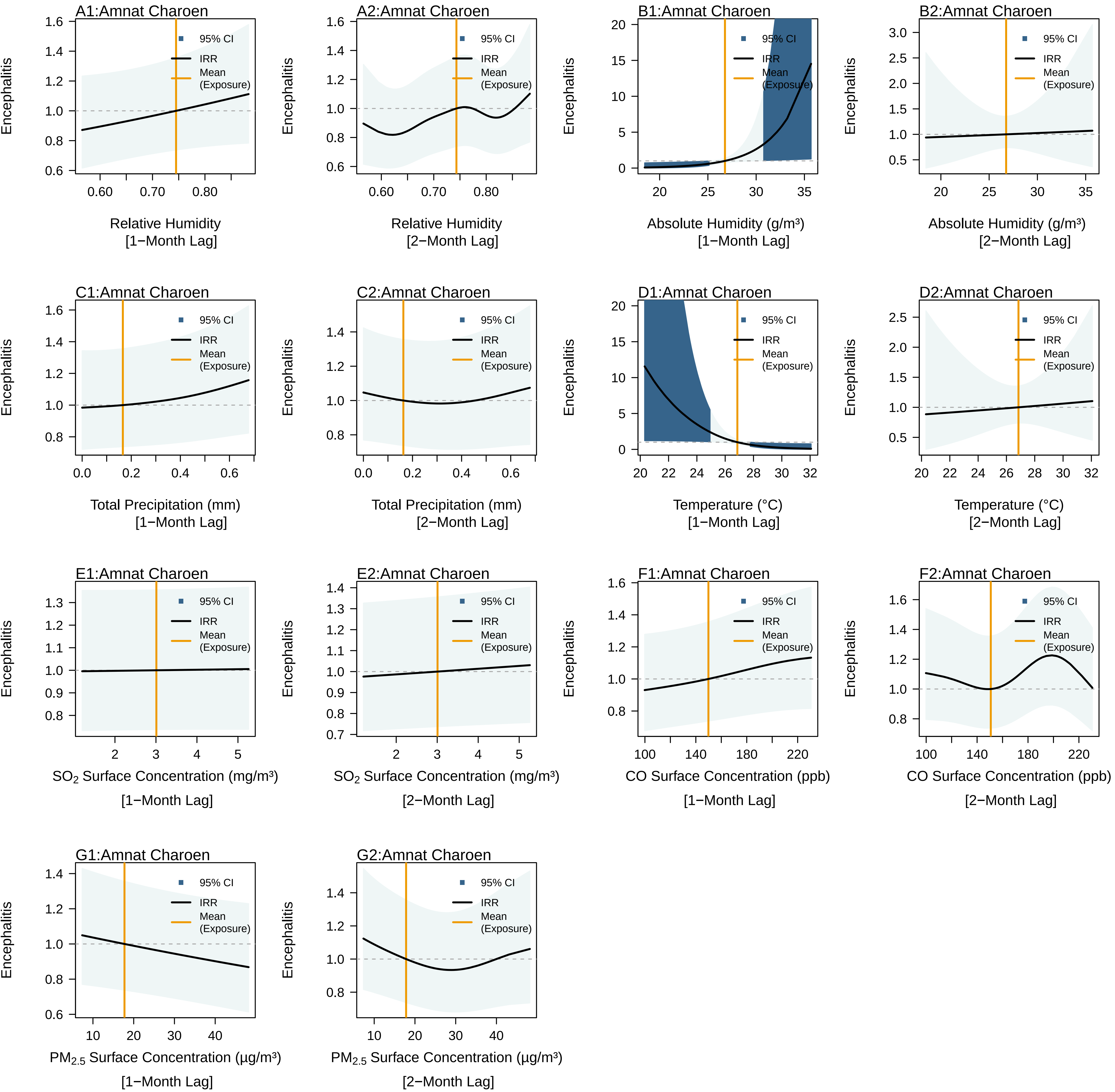
A1:Amnat Charoen
A2:Amnat Charoen
B1:Amnat Charoen
B2:Amnat Charoen
C1:Amnat Charoen
C2:Amnat Charoen
D1:Amnat Charoen
D2:Amnat Charoen
E1:Amnat Charoen
E2:Amnat Charoen
F1:Amnat Charoen
F2:Amnat Charoen
G1:Amnat Charoen
G2:Amnat Charoen
Encephalitis
95% CI
IRR
Mean
(Exposure)
Relative Humidity
[1−Month Lag]
[2−Month Lag]
Absolute Humidity (g/m³)
Total Precipitation (mm)
Temperature (°C)
SO2 Surface Concentration (mg/m³)
CO Surface Concentration (ppb)
PM2.5 Surface Concentration (µg/m³)

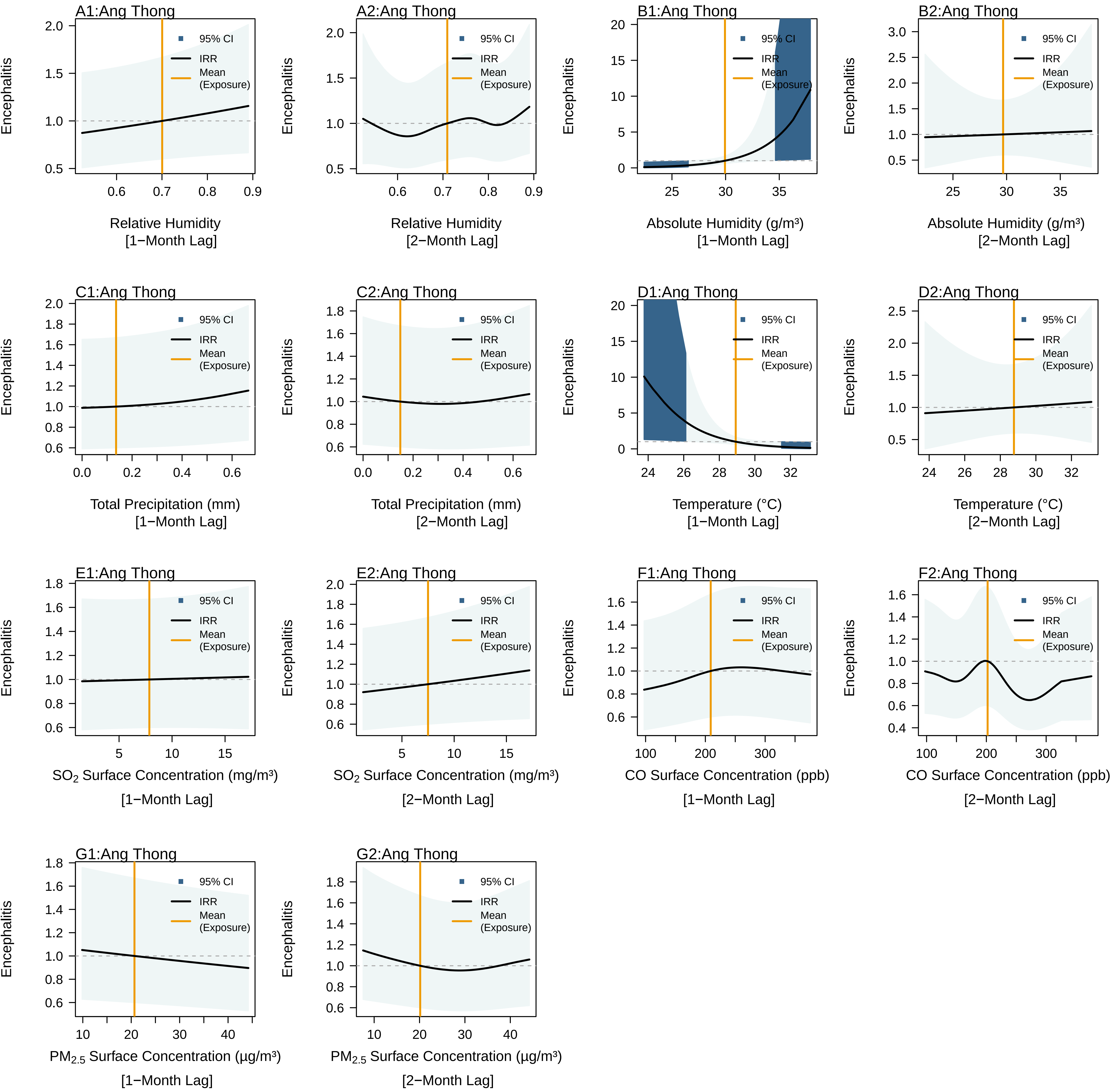
A1:Ang Thong
Encephalitis
95% CI
IRR
Mean (Exposure)
Relative Humidity [1−Month Lag]
A2:Ang Thong
Relative Humidity [2−Month Lag]
B1:Ang Thong
Absolute Humidity (g/m³) [1−Month Lag]
B2:Ang Thong
Absolute Humidity (g/m³) [2−Month Lag]
C1:Ang Thong
Total Precipitation (mm) [1−Month Lag]
C2:Ang Thong
Total Precipitation (mm) [2−Month Lag]
D1:Ang Thong
Temperature (°C) [1−Month Lag]
D2:Ang Thong
Temperature (°C) [2−Month Lag]
E1:Ang Thong
SO_2 Surface Concentration (mg/m³) [1−Month Lag]
E2:Ang Thong
SO_2 Surface Concentration (mg/m³) [2−Month Lag]
F1:Ang Thong
CO Surface Concentration (ppb) [1−Month Lag]
F2:Ang Thong
CO Surface Concentration (ppb) [2−Month Lag]
G1:Ang Thong
$PM_{2.5}$ Surface Concentration (µg/m³) [1−Month Lag]
G2:Ang Thong
$PM_{2.5}$ Surface Concentration (µg/m³) [2−Month Lag]

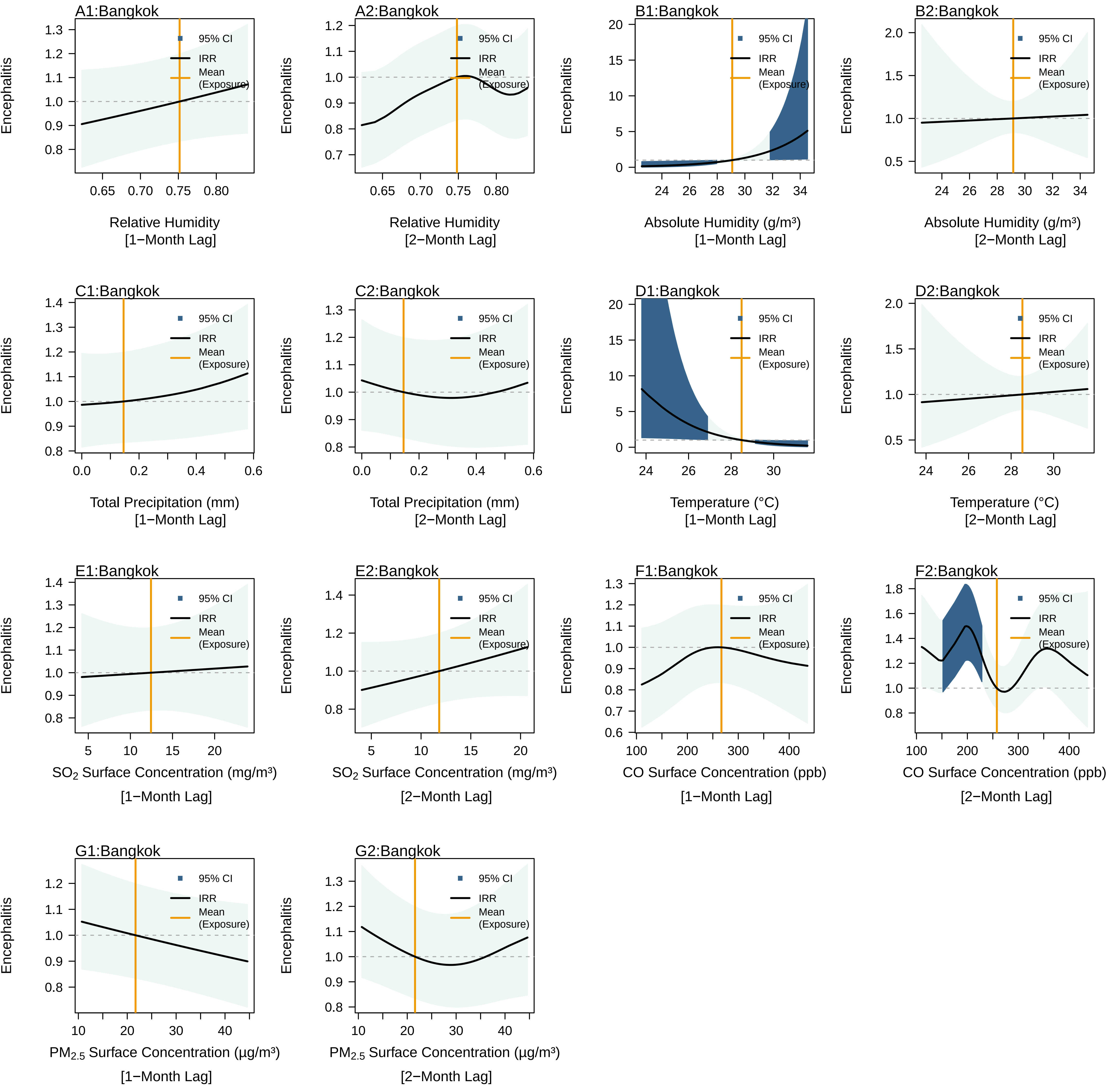

A1:Bangkok
Encephalitis
95% CI
IRR
Mean (Exposure)
Relative Humidity [1−Month Lag]
A2:Bangkok
Relative Humidity [2−Month Lag]
B1:Bangkok
Absolute Humidity (g/m³) [1−Month Lag]
B2:Bangkok
Absolute Humidity (g/m³) [2−Month Lag]
C1:Bangkok
Total Precipitation (mm) [1−Month Lag]
C2:Bangkok
Total Precipitation (mm) [2−Month Lag]
D1:Bangkok
Temperature (°C) [1−Month Lag]
D2:Bangkok
Temperature (°C) [2−Month Lag]
E1:Bangkok
SO_2 Surface Concentration (mg/m³) [1−Month Lag]
E2:Bangkok
SO_2 Surface Concentration (mg/m³) [2−Month Lag]
F1:Bangkok
CO Surface Concentration (ppb) [1−Month Lag]
F2:Bangkok
CO Surface Concentration (ppb) [2−Month Lag]
G1:Bangkok
$PM_{2.5}$ Surface Concentration (µg/m³) [1−Month Lag]
G2:Bangkok
$PM_{2.5}$ Surface Concentration (µg/m³) [2−Month Lag]

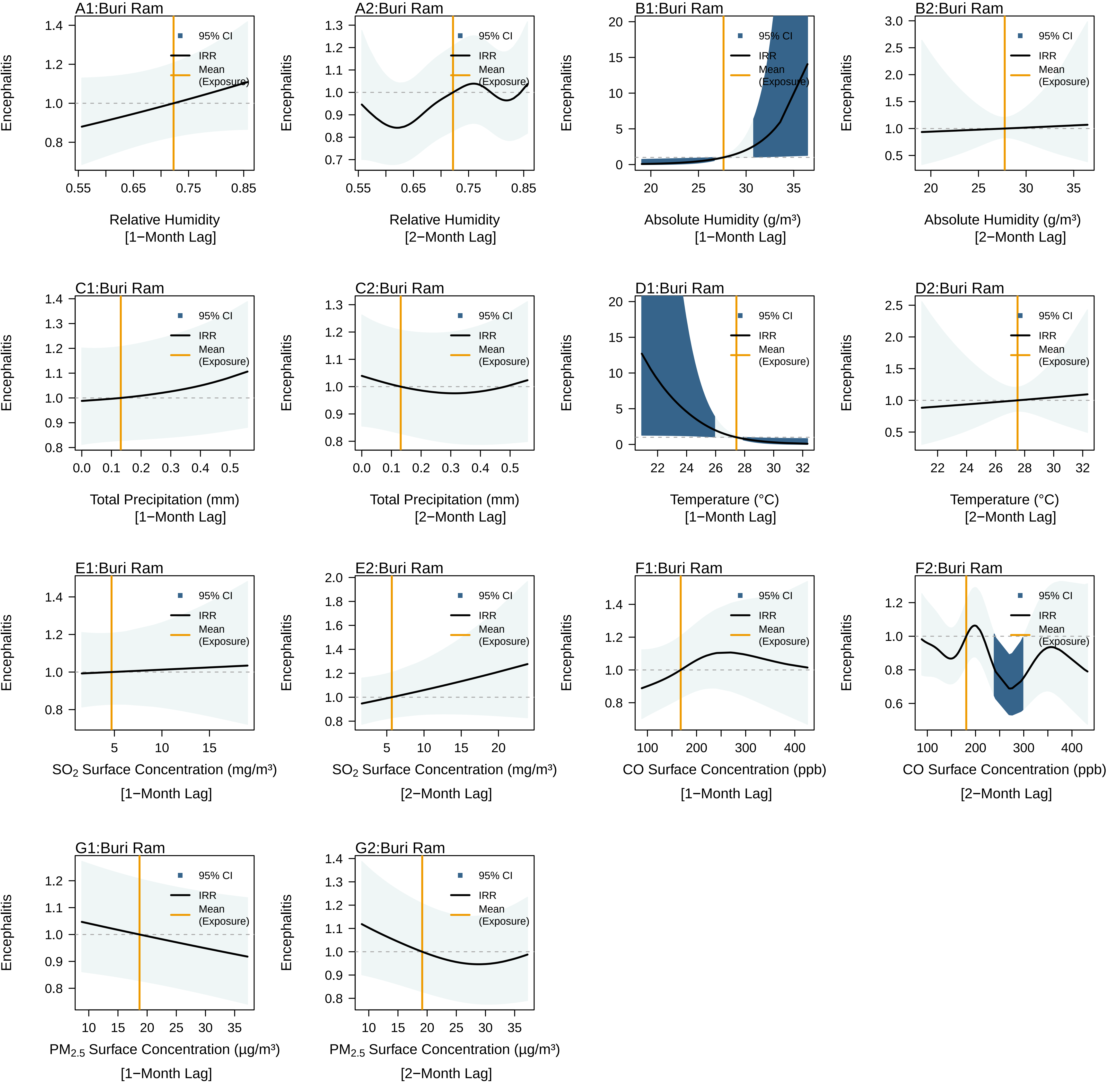
A1:Buri Ram
A2:Buri Ram
B1:Buri Ram
B2:Buri Ram
C1:Buri Ram
C2:Buri Ram
D1:Buri Ram
D2:Buri Ram
E1:Buri Ram
E2:Buri Ram
F1:Buri Ram
F2:Buri Ram
G1:Buri Ram
G2:Buri Ram
Encephalitis
95% CI
IRR
Mean (Exposure)
Relative Humidity [1−Month Lag]
Relative Humidity [2−Month Lag]
Absolute Humidity (g/m³) [1−Month Lag]
Absolute Humidity (g/m³) [2−Month Lag]
Total Precipitation (mm) [1−Month Lag]
Total Precipitation (mm) [2−Month Lag]
Temperature (°C) [1−Month Lag]
Temperature (°C) [2−Month Lag]
SO2 Surface Concentration (mg/m³) [1−Month Lag]
SO2 Surface Concentration (mg/m³) [2−Month Lag]
CO Surface Concentration (ppb) [1−Month Lag]
CO Surface Concentration (ppb) [2−Month Lag]
PM2.5 Surface Concentration (µg/m³) [1−Month Lag]
PM2.5 Surface Concentration (µg/m³) [2−Month Lag]

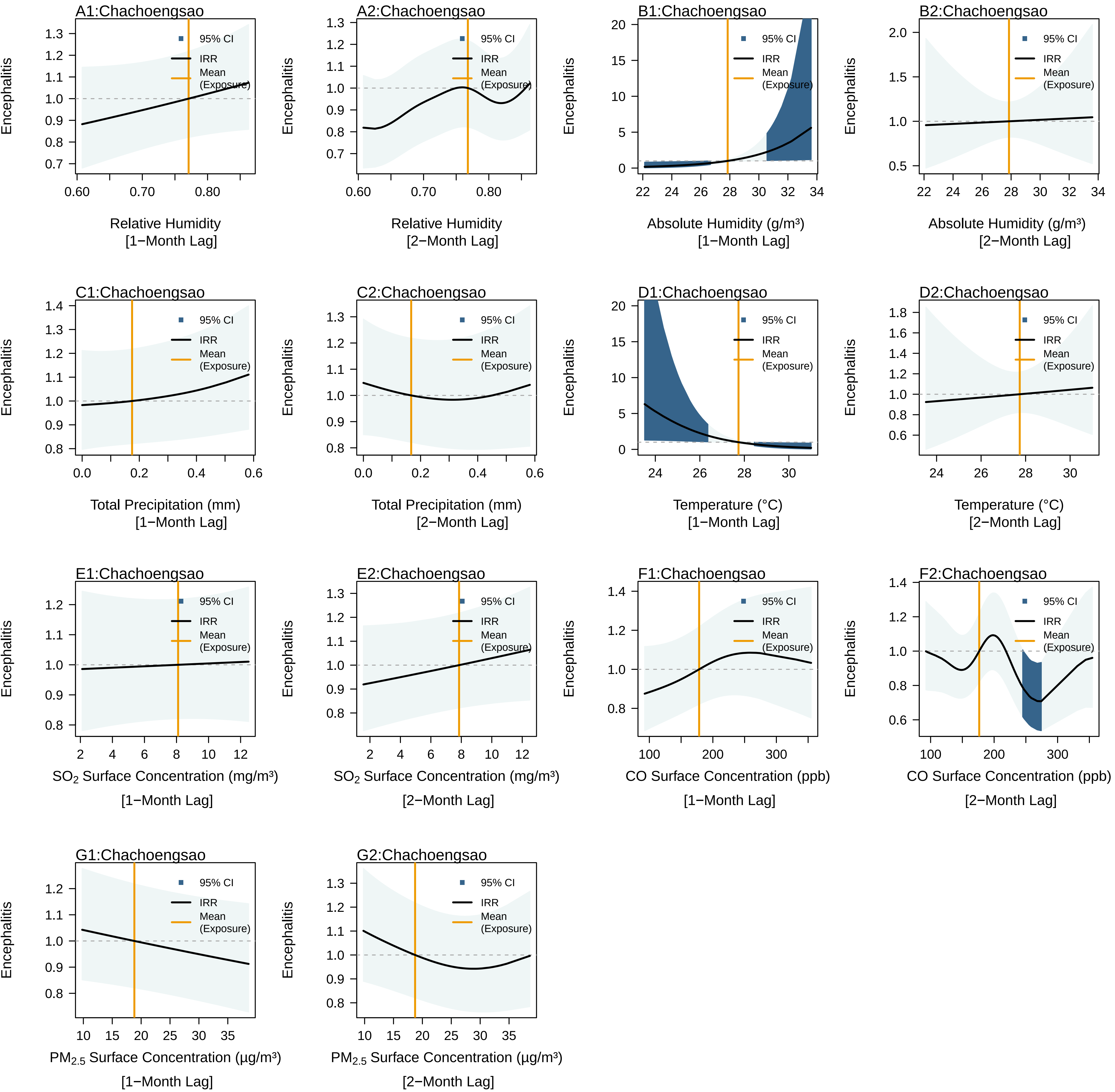
A1:Chachoengsao
Encephalitis
95% CI
IRR
Mean (Exposure)
Relative Humidity [1−Month Lag]
A2:Chachoengsao
Encephalitis
95% CI
IRR
Mean (Exposure)
Relative Humidity [2−Month Lag]
B1:Chachoengsao
Encephalitis
95% CI
IRR
Mean (Exposure)
Absolute Humidity (g/m³) [1−Month Lag]
B2:Chachoengsao
Encephalitis
95% CI
IRR
Mean (Exposure)
Absolute Humidity (g/m³) [2−Month Lag]
C1:Chachoengsao
Encephalitis
95% CI
IRR
Mean (Exposure)
Total Precipitation (mm) [1−Month Lag]
C2:Chachoengsao
Encephalitis
95% CI
IRR
Mean (Exposure)
Total Precipitation (mm) [2−Month Lag]
D1:Chachoengsao
Encephalitis
95% CI
IRR
Mean (Exposure)
Temperature (°C) [1−Month Lag]
D2:Chachoengsao
Encephalitis
95% CI
IRR
Mean (Exposure)
Temperature (°C) [2−Month Lag]
E1:Chachoengsao
Encephalitis
95% CI
IRR
Mean (Exposure)
SO_2 Surface Concentration (mg/m³) [1−Month Lag]
E2:Chachoengsao
Encephalitis
95% CI
IRR
Mean (Exposure)
SO_2 Surface Concentration (mg/m³) [2−Month Lag]
F1:Chachoengsao
Encephalitis
95% CI
IRR
Mean (Exposure)
CO Surface Concentration (ppb) [1−Month Lag]
F2:Chachoengsao
Encephalitis
95% CI
IRR
Mean (Exposure)
CO Surface Concentration (ppb) [2−Month Lag]
G1:Chachoengsao
Encephalitis
95% CI
IRR
Mean (Exposure)
$PM_{2.5}$ Surface Concentration (µg/m³) [1−Month Lag]
G2:Chachoengsao
Encephalitis
95% CI
IRR
Mean (Exposure)
$PM_{2.5}$ Surface Concentration (µg/m³) [2−Month Lag]

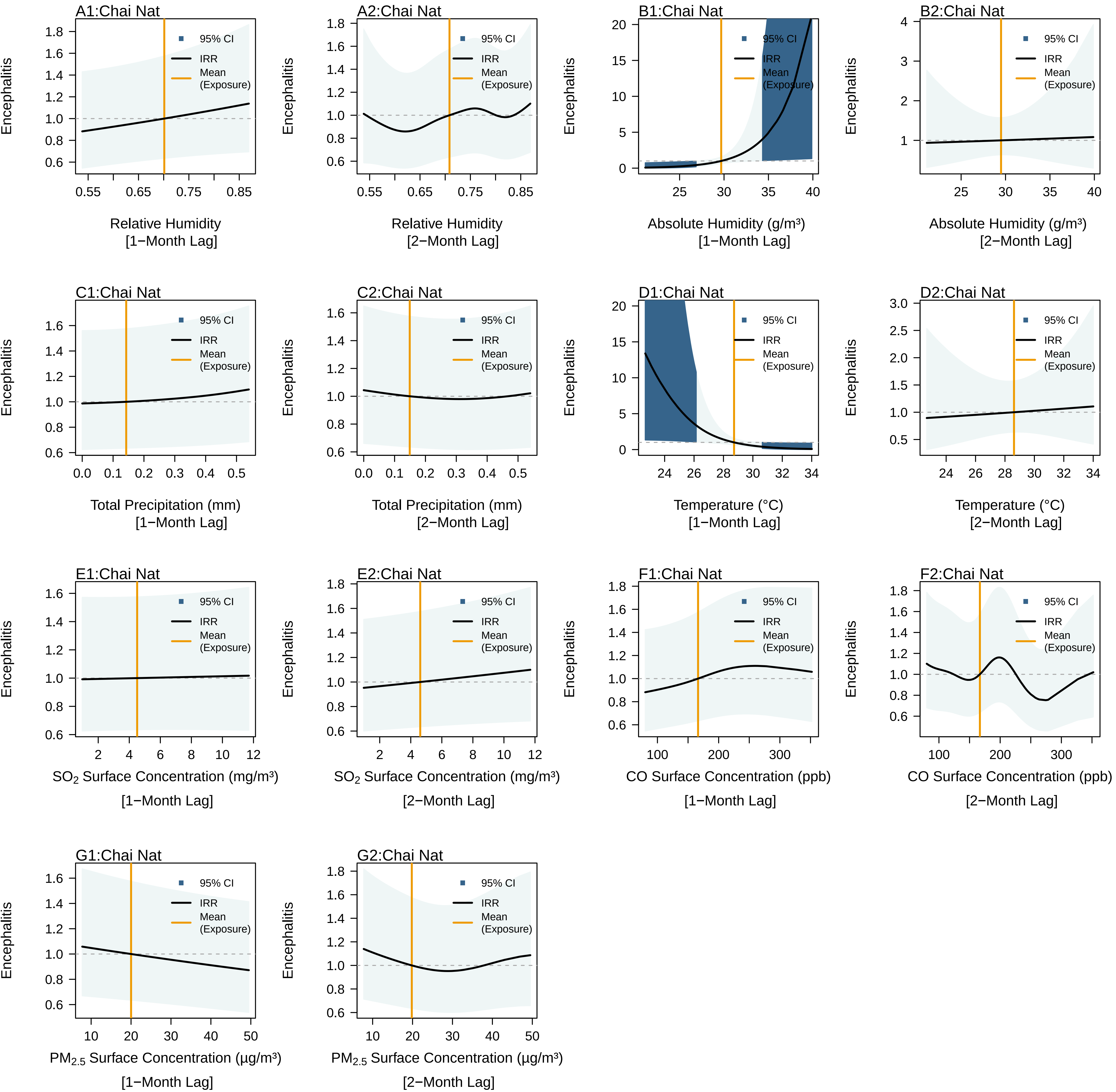
A1:Chai Nat
A2:Chai Nat
B1:Chai Nat
B2:Chai Nat
C1:Chai Nat
C2:Chai Nat
D1:Chai Nat
D2:Chai Nat
E1:Chai Nat
E2:Chai Nat
F1:Chai Nat
F2:Chai Nat
G1:Chai Nat
G2:Chai Nat
Encephalitis
95% CI
IRR
Mean (Exposure)
Relative Humidity [1−Month Lag]
Relative Humidity [2−Month Lag]
Absolute Humidity (g/m³) [1−Month Lag]
Absolute Humidity (g/m³) [2−Month Lag]
Total Precipitation (mm) [1−Month Lag]
Total Precipitation (mm) [2−Month Lag]
Temperature (°C) [1−Month Lag]
Temperature (°C) [2−Month Lag]
SO2 Surface Concentration (mg/m³) [1−Month Lag]
SO2 Surface Concentration (mg/m³) [2−Month Lag]
CO Surface Concentration (ppb) [1−Month Lag]
CO Surface Concentration (ppb) [2−Month Lag]
PM2.5 Surface Concentration (µg/m³) [1−Month Lag]
PM2.5 Surface Concentration (µg/m³) [2−Month Lag]

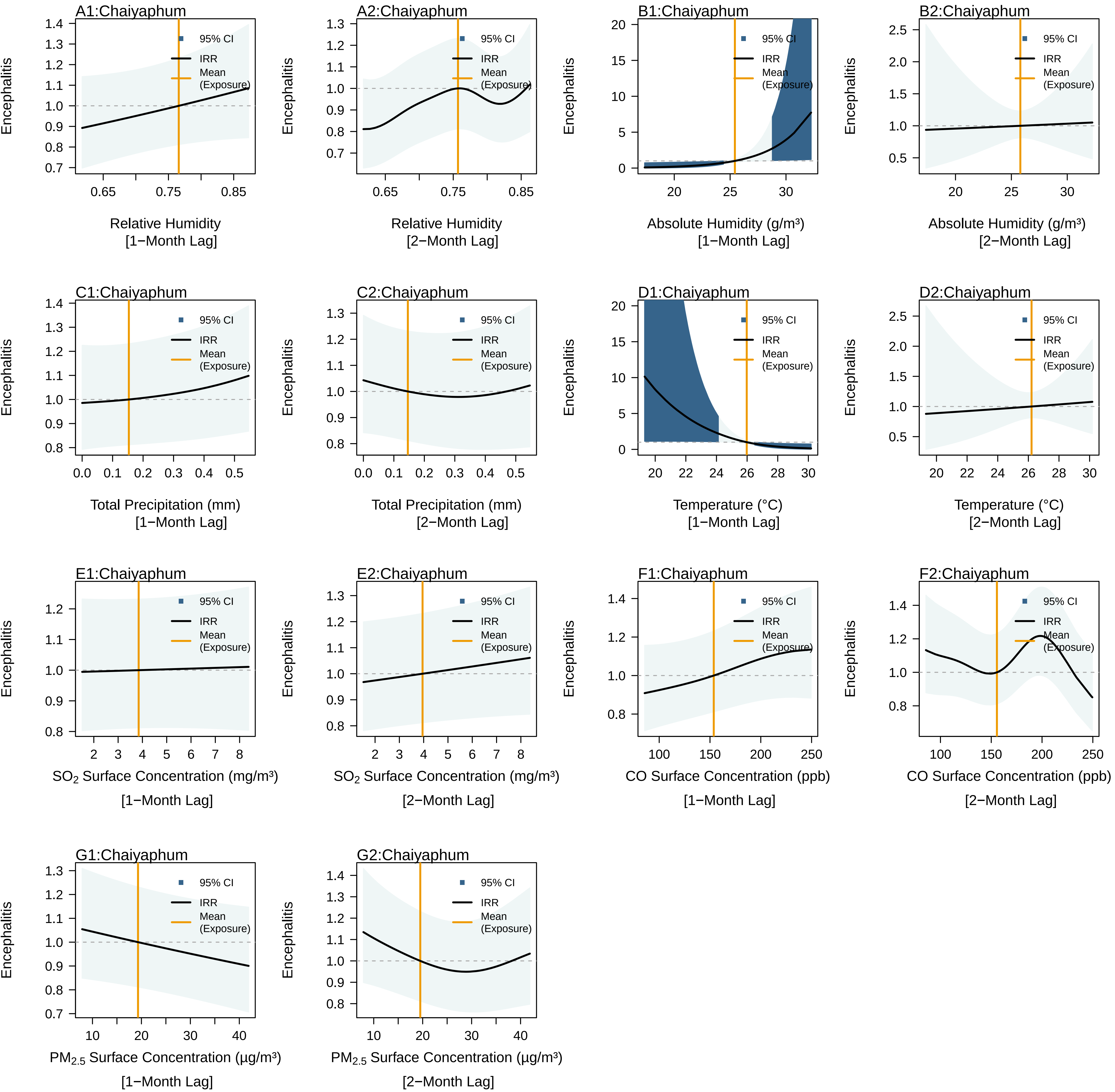

A1:Chaiyaphum
Encephalitis
95% CI
IRR
Mean (Exposure)
Relative Humidity [1−Month Lag]
A2:Chaiyaphum
Relative Humidity [2−Month Lag]
B1:Chaiyaphum
Absolute Humidity (g/m³) [1−Month Lag]
B2:Chaiyaphum
Absolute Humidity (g/m³) [2−Month Lag]
C1:Chaiyaphum
Total Precipitation (mm) [1−Month Lag]
C2:Chaiyaphum
Total Precipitation (mm) [2−Month Lag]
D1:Chaiyaphum
Temperature (°C) [1−Month Lag]
D2:Chaiyaphum
Temperature (°C) [2−Month Lag]
E1:Chaiyaphum
SO_2 Surface Concentration (mg/m³) [1−Month Lag]
E2:Chaiyaphum
SO_2 Surface Concentration (mg/m³) [2−Month Lag]
F1:Chaiyaphum
CO Surface Concentration (ppb) [1−Month Lag]
F2:Chaiyaphum
CO Surface Concentration (ppb) [2−Month Lag]
G1:Chaiyaphum
$PM_{2.5}$ Surface Concentration (µg/m³) [1−Month Lag]
G2:Chaiyaphum
$PM_{2.5}$ Surface Concentration (µg/m³) [2−Month Lag]

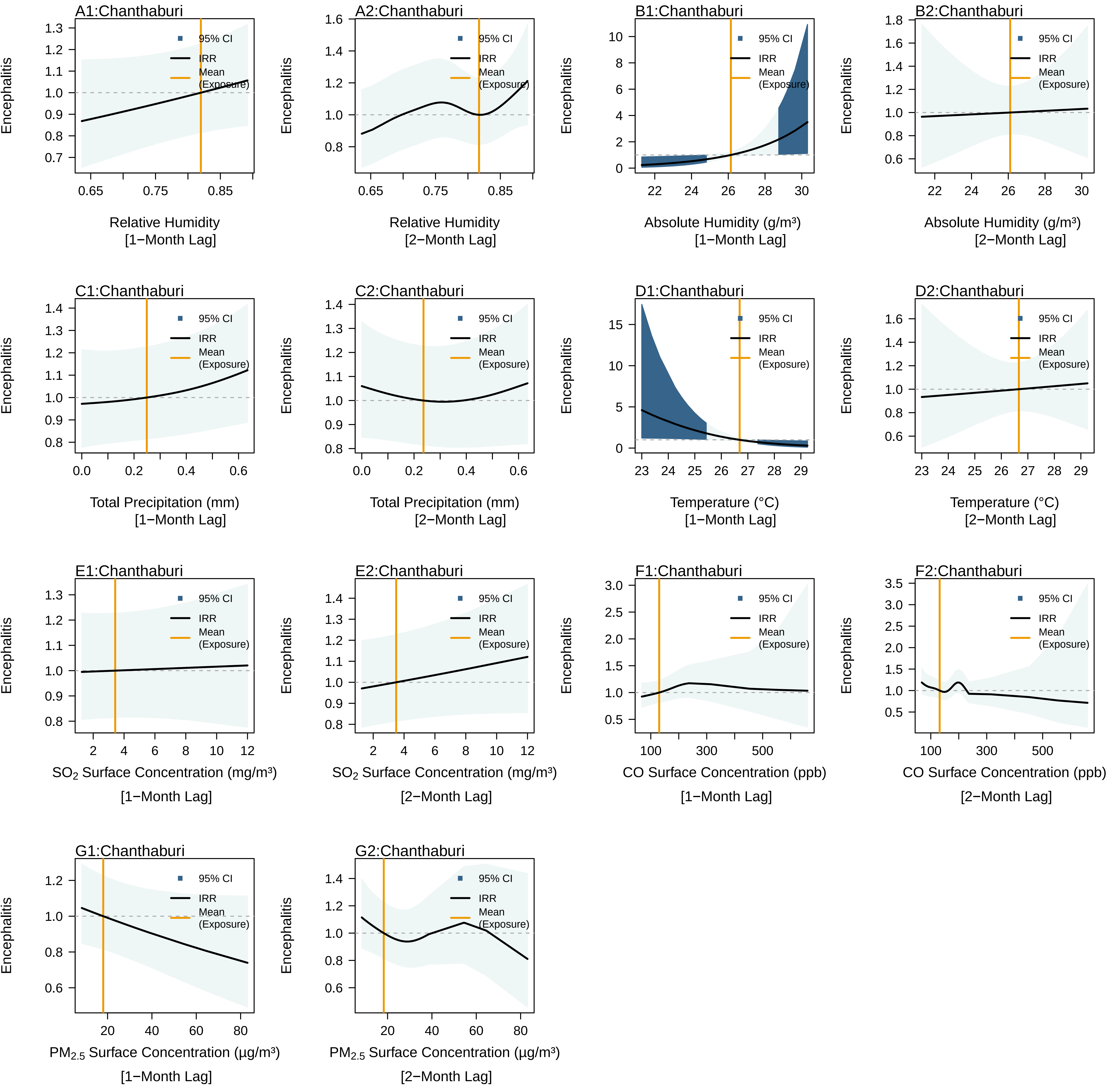

A1:Chanthaburi
Relative Humidity [1−Month Lag]
A2:Chanthaburi
Relative Humidity [2−Month Lag]
B1:Chanthaburi
Absolute Humidity (g/m³) [1−Month Lag]
B2:Chanthaburi
Absolute Humidity (g/m³) [2−Month Lag]
C1:Chanthaburi
Total Precipitation (mm) [1−Month Lag]
C2:Chanthaburi
Total Precipitation (mm) [2−Month Lag]
D1:Chanthaburi
Temperature (°C) [1−Month Lag]
D2:Chanthaburi
Temperature (°C) [2−Month Lag]
E1:Chanthaburi
SO_2 Surface Concentration (mg/m³) [1−Month Lag]
E2:Chanthaburi
SO_2 Surface Concentration (mg/m³) [2−Month Lag]
F1:Chanthaburi
CO Surface Concentration (ppb) [1−Month Lag]
F2:Chanthaburi
CO Surface Concentration (ppb) [2−Month Lag]
G1:Chanthaburi
$PM_{2.5}$ Surface Concentration (µg/m³) [1−Month Lag]
G2:Chanthaburi
$PM_{2.5}$ Surface Concentration (µg/m³) [2−Month Lag]
Encephalitis
95% CI
IRR
Mean (Exposure)

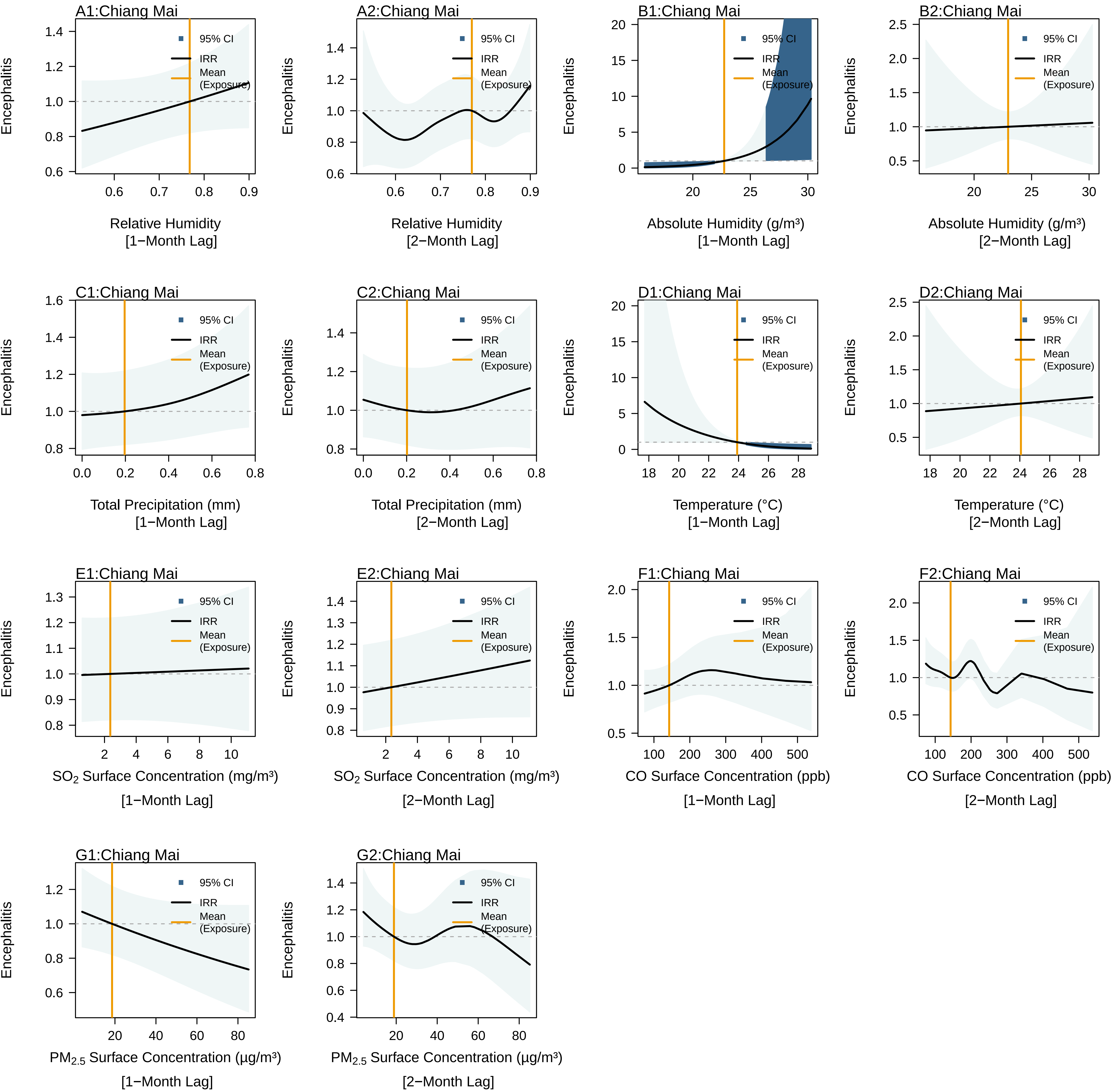

A1:Chiang Mai
95% CI
IRR
Mean (Exposure)
Encephalitis
Relative Humidity [1−Month Lag]
A2:Chiang Mai
95% CI
IRR
Mean (Exposure)
Encephalitis
Relative Humidity [2−Month Lag]
B1:Chiang Mai
95% CI
IRR
Mean (Exposure)
Encephalitis
Absolute Humidity (g/m³) [1−Month Lag]
B2:Chiang Mai
95% CI
IRR
Mean (Exposure)
Encephalitis
Absolute Humidity (g/m³) [2−Month Lag]
C1:Chiang Mai
95% CI
IRR
Mean (Exposure)
Encephalitis
Total Precipitation (mm) [1−Month Lag]
C2:Chiang Mai
95% CI
IRR
Mean (Exposure)
Encephalitis
Total Precipitation (mm) [2−Month Lag]
D1:Chiang Mai
95% CI
IRR
Mean (Exposure)
Encephalitis
Temperature (°C) [1−Month Lag]
D2:Chiang Mai
95% CI
IRR
Mean (Exposure)
Encephalitis
Temperature (°C) [2−Month Lag]
E1:Chiang Mai
95% CI
IRR
Mean (Exposure)
Encephalitis
SO_2 Surface Concentration (mg/m³) [1−Month Lag]
E2:Chiang Mai
95% CI
IRR
Mean (Exposure)
Encephalitis
SO_2 Surface Concentration (mg/m³) [2−Month Lag]
F1:Chiang Mai
95% CI
IRR
Mean (Exposure)
Encephalitis
CO Surface Concentration (ppb) [1−Month Lag]
F2:Chiang Mai
95% CI
IRR
Mean (Exposure)
Encephalitis
CO Surface Concentration (ppb) [2−Month Lag]
G1:Chiang Mai
95% CI
IRR
Mean (Exposure)
Encephalitis
$PM_{2.5}$ Surface Concentration (µg/m³) [1−Month Lag]
G2:Chiang Mai
95% CI
IRR
Mean (Exposure)
Encephalitis
$PM_{2.5}$ Surface Concentration (µg/m³) [2−Month Lag]

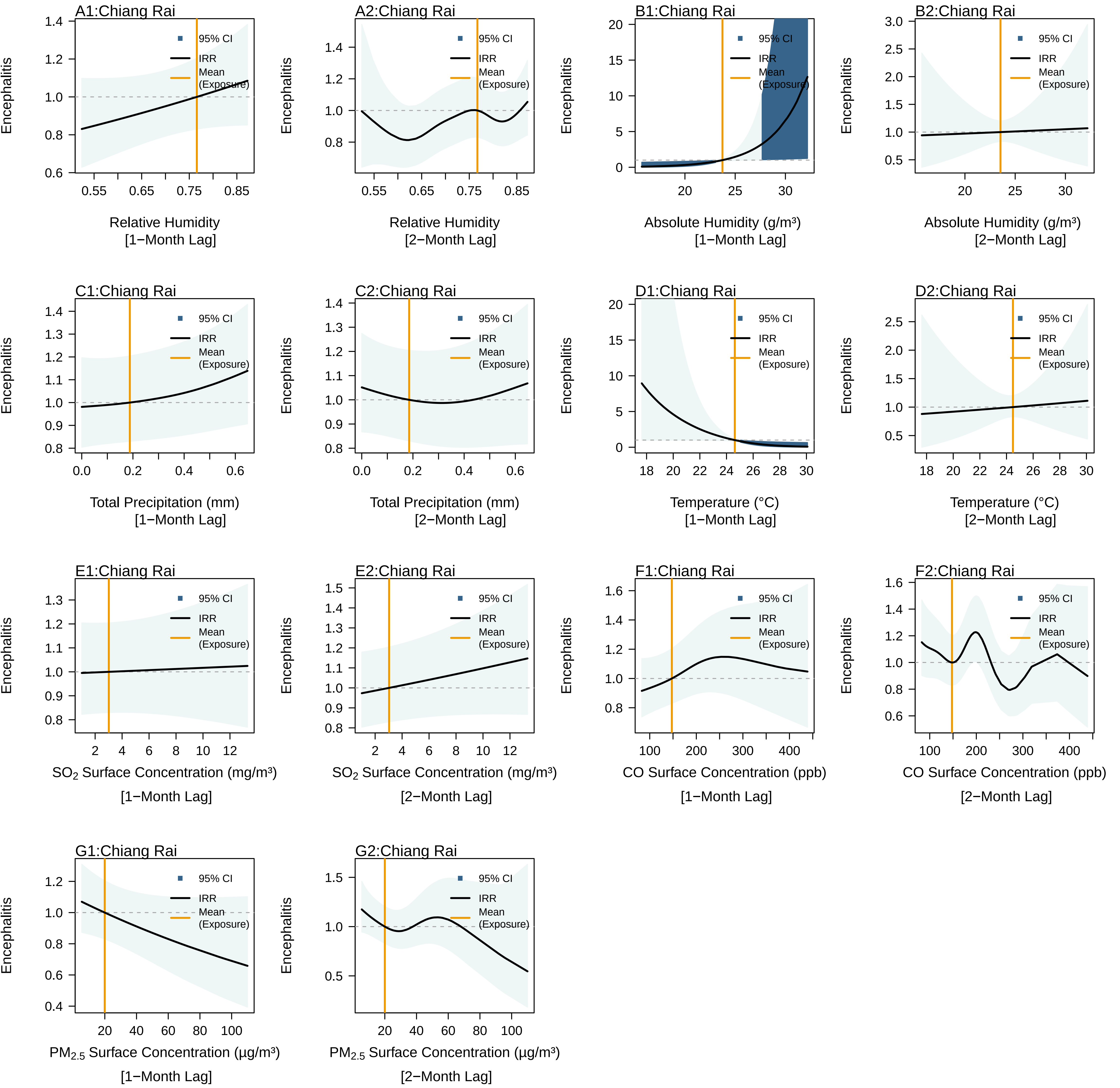
A1:Chiang Rai
Encephalitis
95% CI
IRR
Mean (Exposure)
Relative Humidity [1−Month Lag]
A2:Chiang Rai
Relative Humidity [2−Month Lag]
B1:Chiang Rai
Absolute Humidity (g/m³) [1−Month Lag]
B2:Chiang Rai
Absolute Humidity (g/m³) [2−Month Lag]
C1:Chiang Rai
Total Precipitation (mm) [1−Month Lag]
C2:Chiang Rai
Total Precipitation (mm) [2−Month Lag]
D1:Chiang Rai
Temperature (°C) [1−Month Lag]
D2:Chiang Rai
Temperature (°C) [2−Month Lag]
E1:Chiang Rai
SO_2 Surface Concentration (mg/m³) [1−Month Lag]
E2:Chiang Rai
SO_2 Surface Concentration (mg/m³) [2−Month Lag]
F1:Chiang Rai
CO Surface Concentration (ppb) [1−Month Lag]
F2:Chiang Rai
CO Surface Concentration (ppb) [2−Month Lag]
G1:Chiang Rai
$PM_{2.5}$ Surface Concentration (µg/m³) [1−Month Lag]
G2:Chiang Rai
$PM_{2.5}$ Surface Concentration (µg/m³) [2−Month Lag]

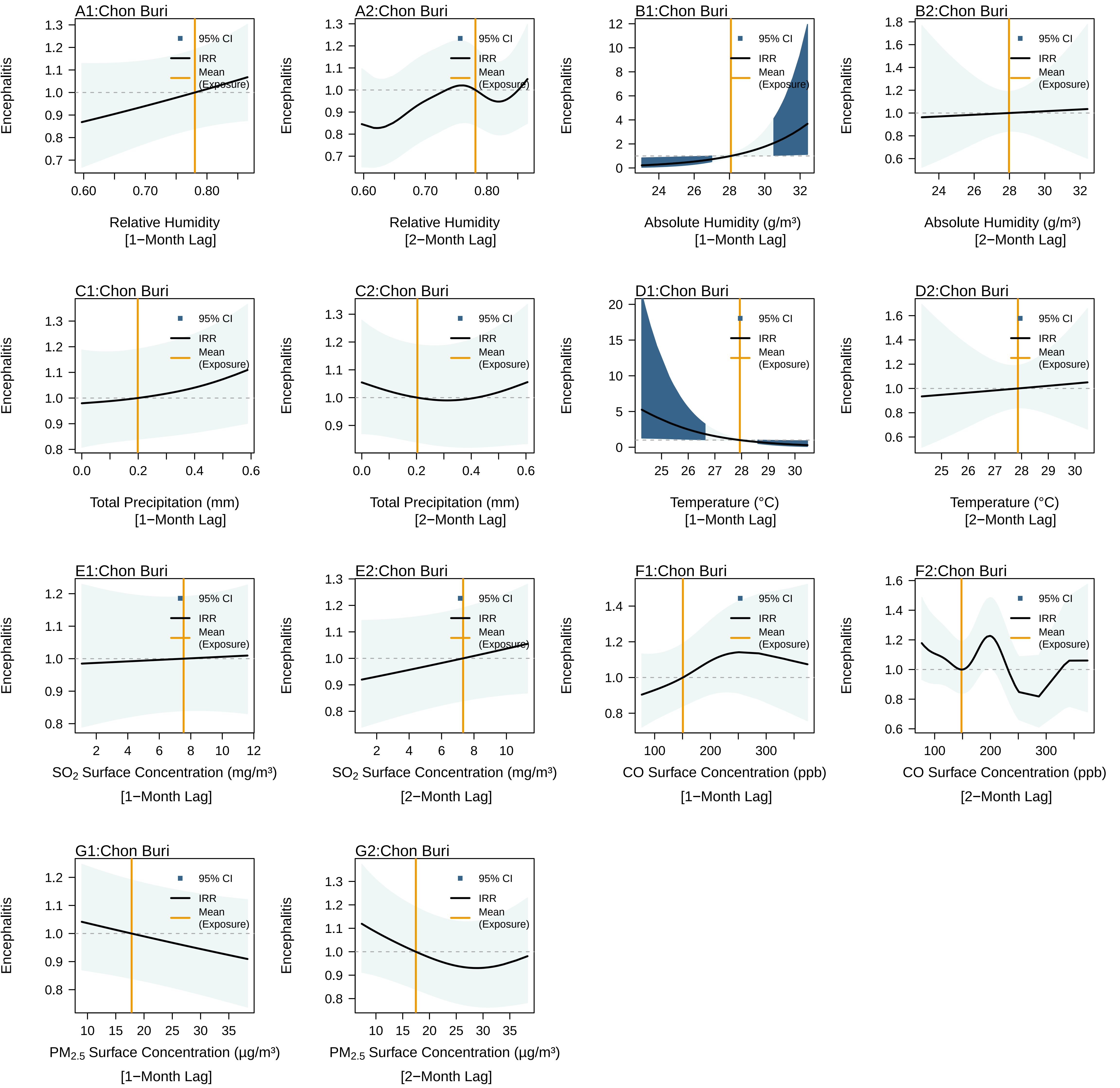

A1:Chon Buri
A2:Chon Buri
B1:Chon Buri
B2:Chon Buri
C1:Chon Buri
C2:Chon Buri
D1:Chon Buri
D2:Chon Buri
E1:Chon Buri
E2:Chon Buri
F1:Chon Buri
F2:Chon Buri
G1:Chon Buri
G2:Chon Buri
95% CI
IRR
Mean (Exposure)
Encephalitis
Relative Humidity [1−Month Lag]
Relative Humidity [2−Month Lag]
Absolute Humidity (g/m³) [1−Month Lag]
Absolute Humidity (g/m³) [2−Month Lag]
Total Precipitation (mm) [1−Month Lag]
Total Precipitation (mm) [2−Month Lag]
Temperature (°C) [1−Month Lag]
Temperature (°C) [2−Month Lag]
SO2 Surface Concentration (mg/m³) [1−Month Lag]
SO2 Surface Concentration (mg/m³) [2−Month Lag]
CO Surface Concentration (ppb) [1−Month Lag]
CO Surface Concentration (ppb) [2−Month Lag]
PM2.5 Surface Concentration (µg/m³) [1−Month Lag]
PM2.5 Surface Concentration (µg/m³) [2−Month Lag]

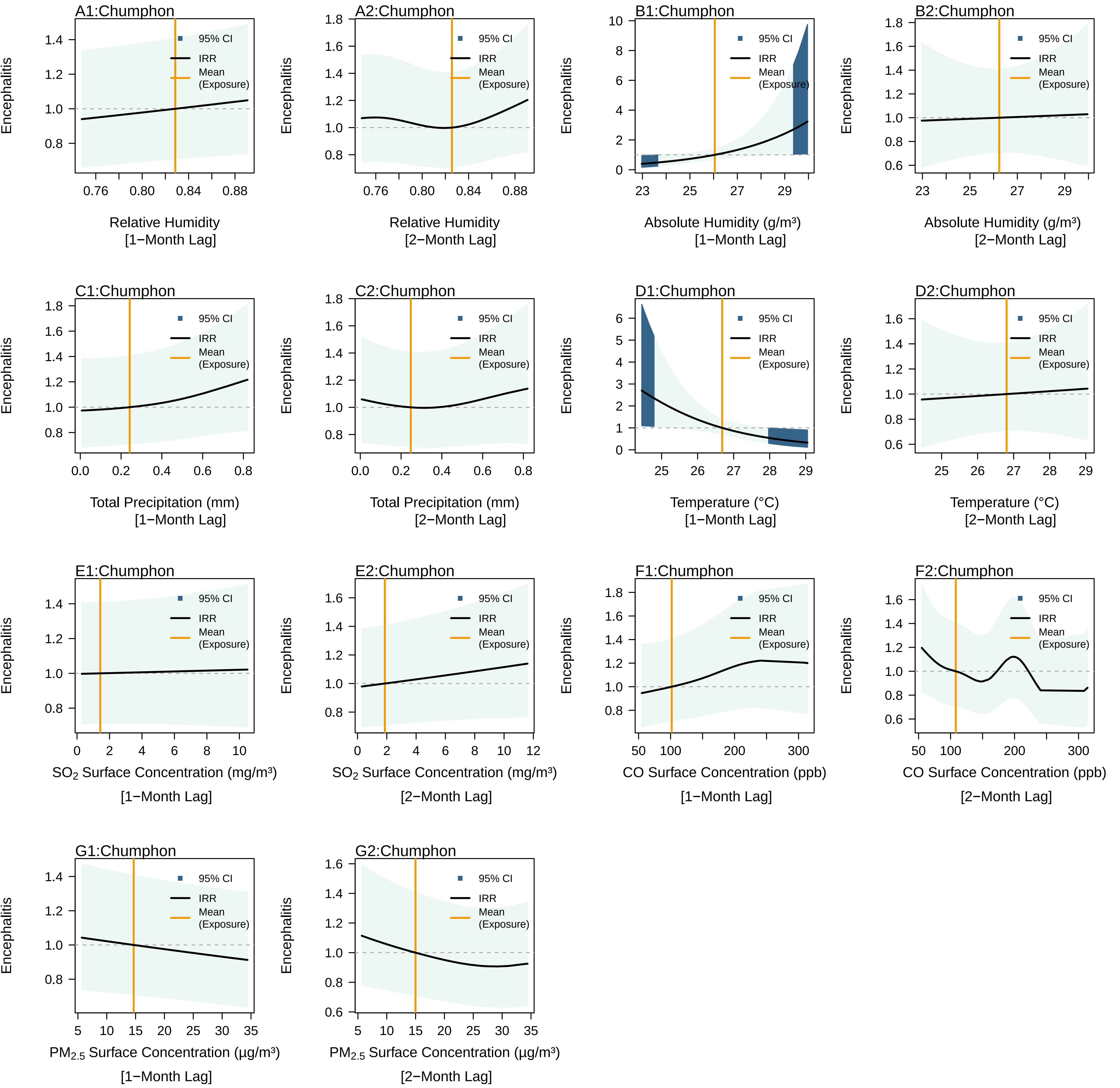

A1:Chumphon
A2:Chumphon
B1:Chumphon
B2:Chumphon
95% CI
IRR
Mean (Exposure)
Encephalitis
Relative Humidity [1−Month Lag]
Relative Humidity [2−Month Lag]
Absolute Humidity (g/m³) [1−Month Lag]
Absolute Humidity (g/m³) [2−Month Lag]
C1:Chumphon
C2:Chumphon
D1:Chumphon
D2:Chumphon
Total Precipitation (mm) [1−Month Lag]
Total Precipitation (mm) [2−Month Lag]
Temperature (°C) [1−Month Lag]
Temperature (°C) [2−Month Lag]
E1:Chumphon
E2:Chumphon
F1:Chumphon
F2:Chumphon
SO2 Surface Concentration (mg/m³) [1−Month Lag]
SO2 Surface Concentration (mg/m³) [2−Month Lag]
CO Surface Concentration (ppb) [1−Month Lag]
CO Surface Concentration (ppb) [2−Month Lag]
G1:Chumphon
G2:Chumphon
PM2.5 Surface Concentration (µg/m³) [1−Month Lag]
PM2.5 Surface Concentration (µg/m³) [2−Month Lag]

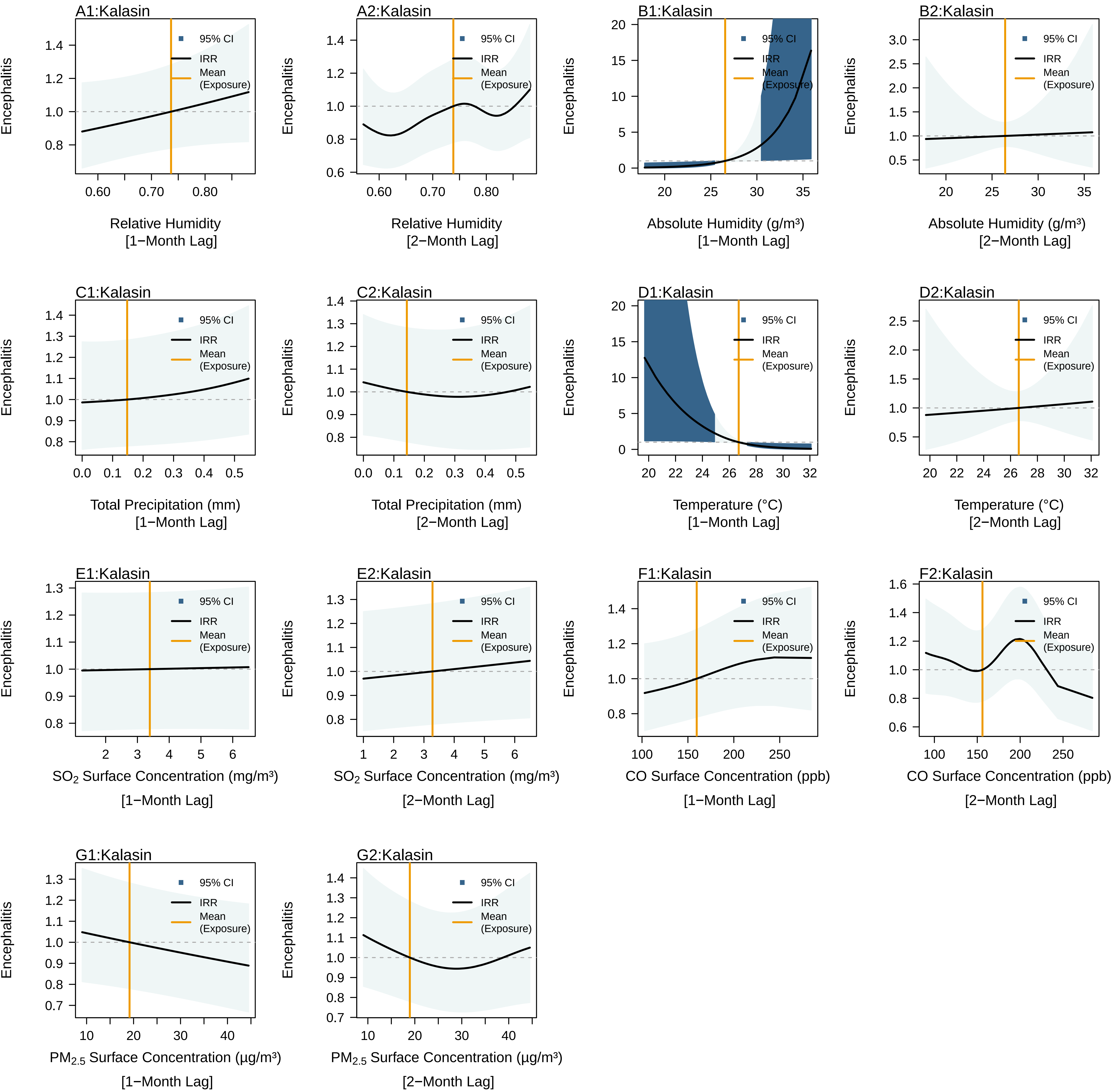
A1:Kalasin
Encephalitis
95% CI
IRR
Mean (Exposure)
Relative Humidity [1−Month Lag]
A2:Kalasin
Relative Humidity [2−Month Lag]
B1:Kalasin
Absolute Humidity (g/m³) [1−Month Lag]
B2:Kalasin
Absolute Humidity (g/m³) [2−Month Lag]
C1:Kalasin
Total Precipitation (mm) [1−Month Lag]
C2:Kalasin
Total Precipitation (mm) [2−Month Lag]
D1:Kalasin
Temperature (°C) [1−Month Lag]
D2:Kalasin
Temperature (°C) [2−Month Lag]
E1:Kalasin
SO_2 Surface Concentration (mg/m³) [1−Month Lag]
E2:Kalasin
SO_2 Surface Concentration (mg/m³) [2−Month Lag]
F1:Kalasin
CO Surface Concentration (ppb) [1−Month Lag]
F2:Kalasin
CO Surface Concentration (ppb) [2−Month Lag]
G1:Kalasin
$PM_{2.5}$ Surface Concentration (µg/m³) [1−Month Lag]
G2:Kalasin
$PM_{2.5}$ Surface Concentration (µg/m³) [2−Month Lag]

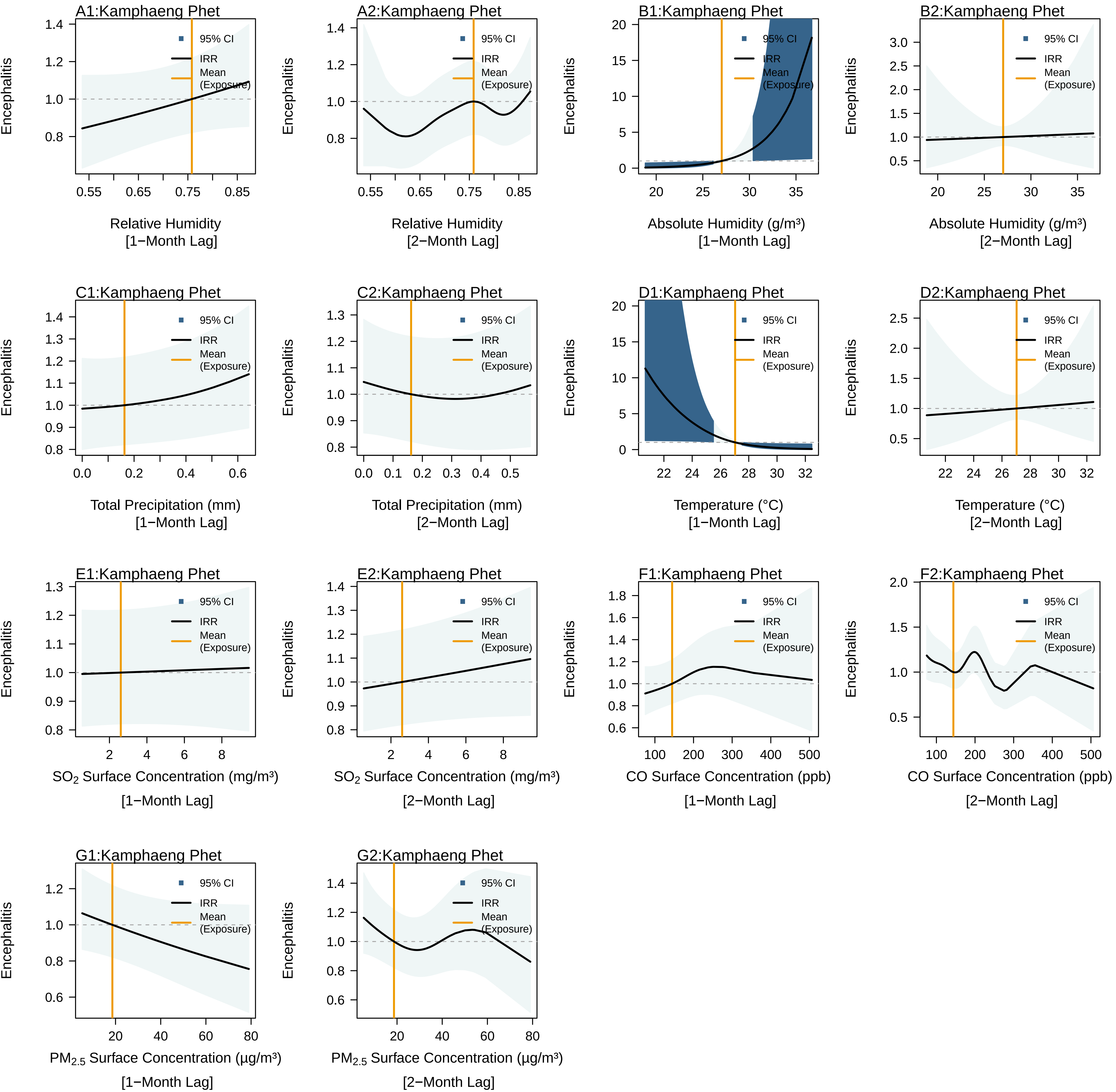

A1:Kamphaeng Phet
Encephalitis
95% CI
IRR
Mean (Exposure)
Relative Humidity [1−Month Lag]
A2:Kamphaeng Phet
Encephalitis
95% CI
IRR
Mean (Exposure)
Relative Humidity [2−Month Lag]
B1:Kamphaeng Phet
Encephalitis
95% CI
IRR
Mean (Exposure)
Absolute Humidity (g/m³) [1−Month Lag]
B2:Kamphaeng Phet
Encephalitis
95% CI
IRR
Mean (Exposure)
Absolute Humidity (g/m³) [2−Month Lag]
C1:Kamphaeng Phet
Encephalitis
95% CI
IRR
Mean (Exposure)
Total Precipitation (mm) [1−Month Lag]
C2:Kamphaeng Phet
Encephalitis
95% CI
IRR
Mean (Exposure)
Total Precipitation (mm) [2−Month Lag]
D1:Kamphaeng Phet
Encephalitis
95% CI
IRR
Mean (Exposure)
Temperature (°C) [1−Month Lag]
D2:Kamphaeng Phet
Encephalitis
95% CI
IRR
Mean (Exposure)
Temperature (°C) [2−Month Lag]
E1:Kamphaeng Phet
Encephalitis
95% CI
IRR
Mean (Exposure)
SO2 Surface Concentration (mg/m³) [1−Month Lag]
E2:Kamphaeng Phet
Encephalitis
95% CI
IRR
Mean (Exposure)
SO2 Surface Concentration (mg/m³) [2−Month Lag]
F1:Kamphaeng Phet
Encephalitis
95% CI
IRR
Mean (Exposure)
CO Surface Concentration (ppb) [1−Month Lag]
F2:Kamphaeng Phet
Encephalitis
95% CI
IRR
Mean (Exposure)
CO Surface Concentration (ppb) [2−Month Lag]
G1:Kamphaeng Phet
Encephalitis
95% CI
IRR
Mean (Exposure)
PM2.5 Surface Concentration (µg/m³) [1−Month Lag]
G2:Kamphaeng Phet
Encephalitis
95% CI
IRR
Mean (Exposure)
PM2.5 Surface Concentration (µg/m³) [2−Month Lag]

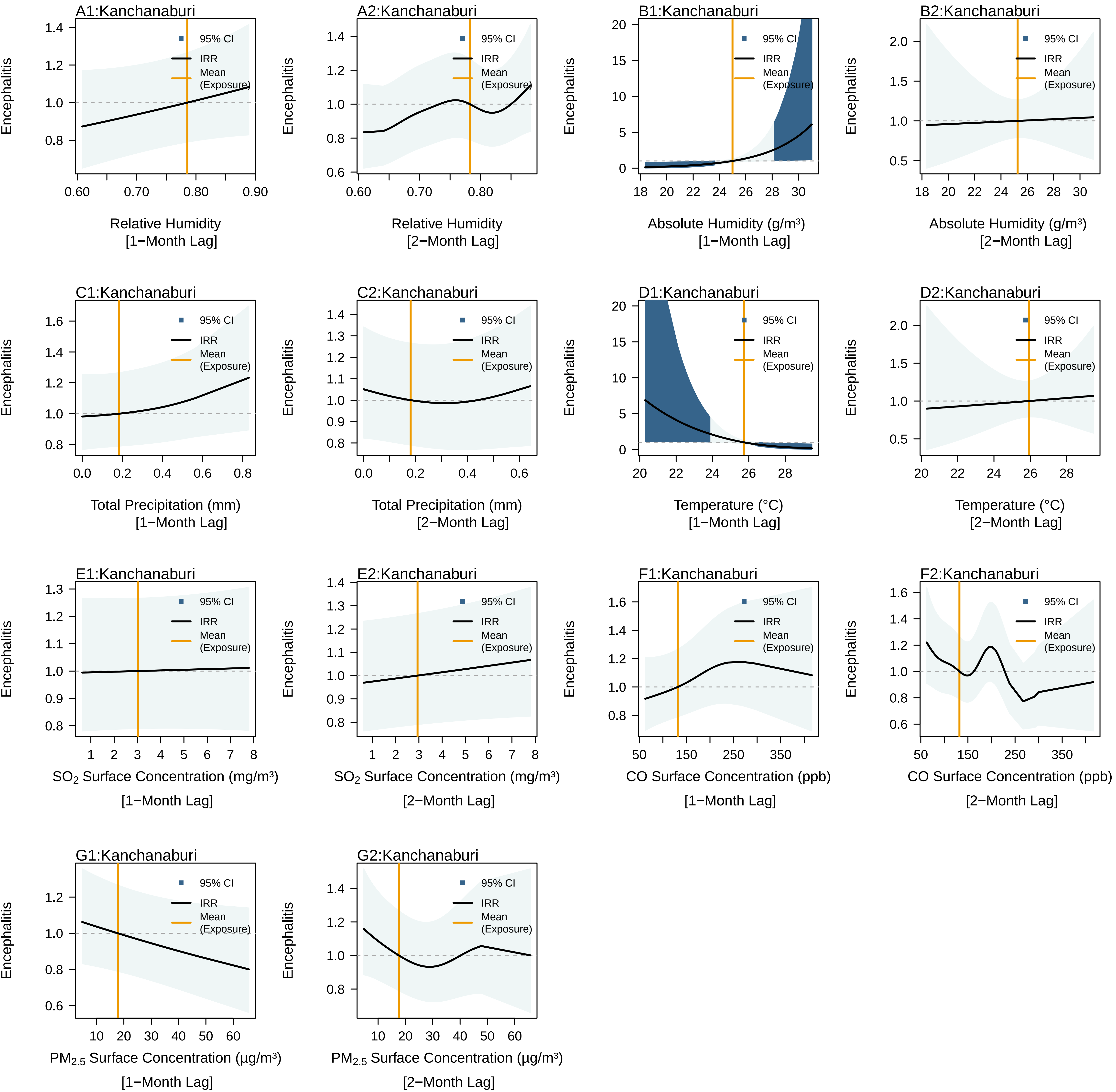
A1:Kanchanaburi
A2:Kanchanaburi
B1:Kanchanaburi
B2:Kanchanaburi
C1:Kanchanaburi
C2:Kanchanaburi
D1:Kanchanaburi
D2:Kanchanaburi
E1:Kanchanaburi
E2:Kanchanaburi
F1:Kanchanaburi
F2:Kanchanaburi
G1:Kanchanaburi
G2:Kanchanaburi
Encephalitis
95% CI
IRR
Mean (Exposure)
Relative Humidity [1−Month Lag]
Relative Humidity [2−Month Lag]
Absolute Humidity (g/m³) [1−Month Lag]
Absolute Humidity (g/m³) [2−Month Lag]
Total Precipitation (mm) [1−Month Lag]
Total Precipitation (mm) [2−Month Lag]
Temperature (°C) [1−Month Lag]
Temperature (°C) [2−Month Lag]
SO_2 Surface Concentration (mg/m³) [1−Month Lag]
SO_2 Surface Concentration (mg/m³) [2−Month Lag]
CO Surface Concentration (ppb) [1−Month Lag]
CO Surface Concentration (ppb) [2−Month Lag]
$PM_{2.5}$ Surface Concentration (µg/m³) [1−Month Lag]
$PM_{2.5}$ Surface Concentration (µg/m³) [2−Month Lag]

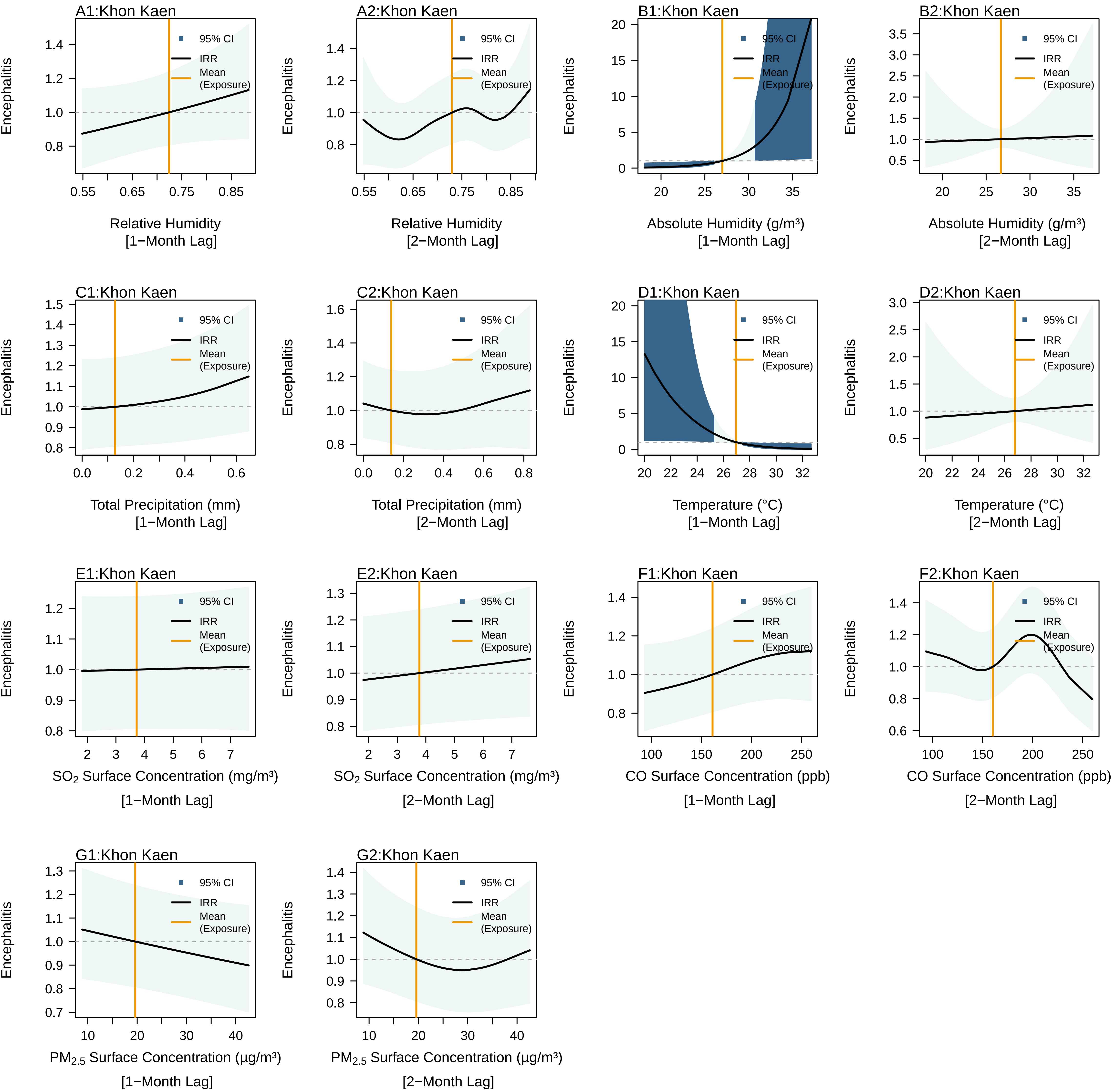
A1:Khon Kaen
A2:Khon Kaen
B1:Khon Kaen
B2:Khon Kaen
C1:Khon Kaen
C2:Khon Kaen
D1:Khon Kaen
D2:Khon Kaen
E1:Khon Kaen
E2:Khon Kaen
F1:Khon Kaen
F2:Khon Kaen
G1:Khon Kaen
G2:Khon Kaen
Encephalitis
95% CI
IRR
Mean (Exposure)
Relative Humidity [1−Month Lag]
Relative Humidity [2−Month Lag]
Absolute Humidity (g/m³) [1−Month Lag]
Absolute Humidity (g/m³) [2−Month Lag]
Total Precipitation (mm) [1−Month Lag]
Total Precipitation (mm) [2−Month Lag]
Temperature (°C) [1−Month Lag]
Temperature (°C) [2−Month Lag]
SO_2 Surface Concentration (mg/m³) [1−Month Lag]
SO_2 Surface Concentration (mg/m³) [2−Month Lag]
CO Surface Concentration (ppb) [1−Month Lag]
CO Surface Concentration (ppb) [2−Month Lag]
$PM_{2.5}$ Surface Concentration (µg/m³) [1−Month Lag]
$PM_{2.5}$ Surface Concentration (µg/m³) [2−Month Lag]

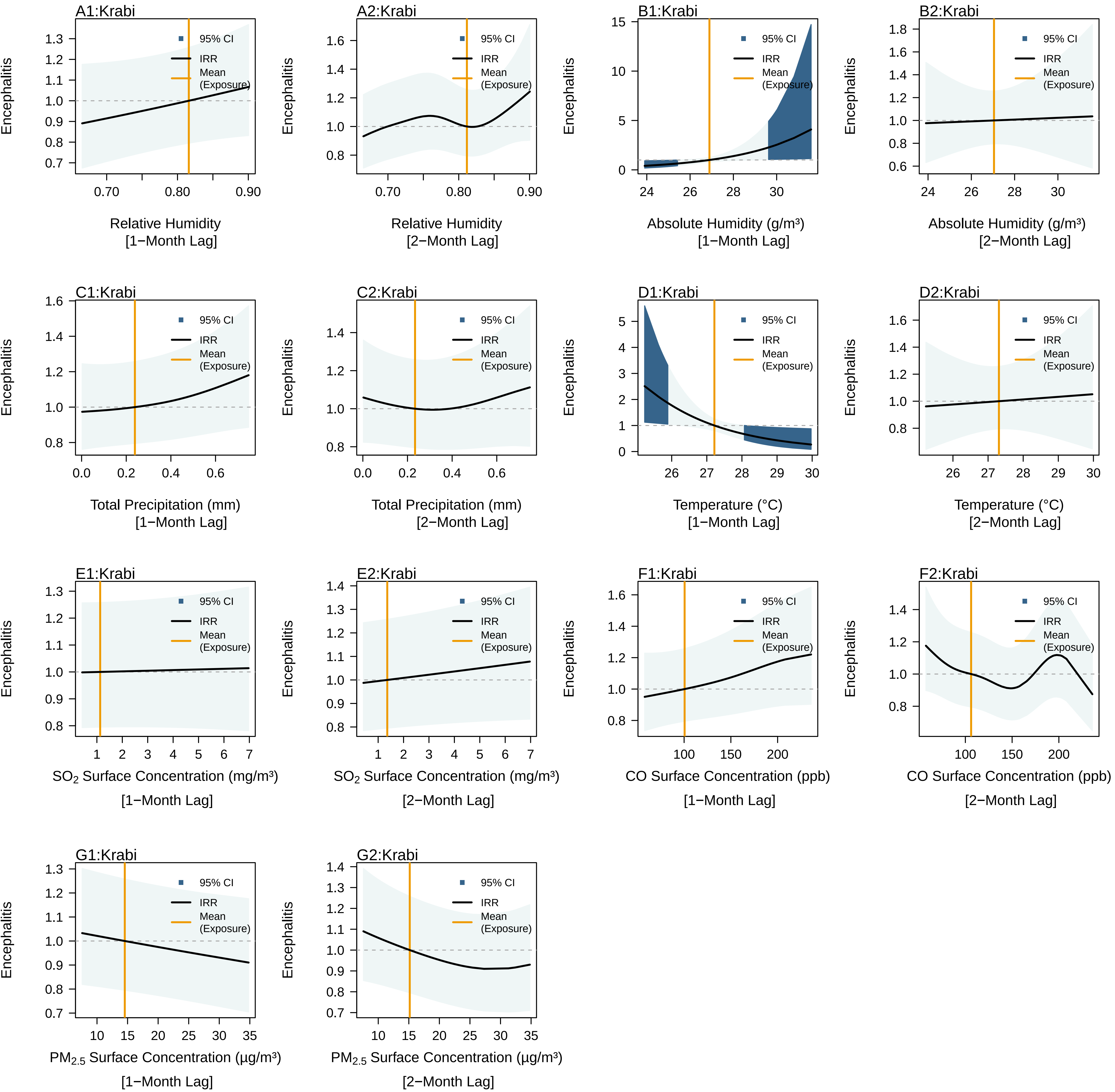

A1:Krabi
A2:Krabi
B1:Krabi
B2:Krabi
C1:Krabi
C2:Krabi
D1:Krabi
D2:Krabi
E1:Krabi
E2:Krabi
F1:Krabi
F2:Krabi
G1:Krabi
G2:Krabi
95% CI
IRR
Mean (Exposure)
Encephalitis
Relative Humidity [1−Month Lag]
Relative Humidity [2−Month Lag]
Absolute Humidity (g/m³) [1−Month Lag]
Absolute Humidity (g/m³) [2−Month Lag]
Total Precipitation (mm) [1−Month Lag]
Total Precipitation (mm) [2−Month Lag]
Temperature (°C) [1−Month Lag]
Temperature (°C) [2−Month Lag]
SO2 Surface Concentration (mg/m³) [1−Month Lag]
SO2 Surface Concentration (mg/m³) [2−Month Lag]
CO Surface Concentration (ppb) [1−Month Lag]
CO Surface Concentration (ppb) [2−Month Lag]
PM2.5 Surface Concentration (µg/m³) [1−Month Lag]
PM2.5 Surface Concentration (µg/m³) [2−Month Lag]

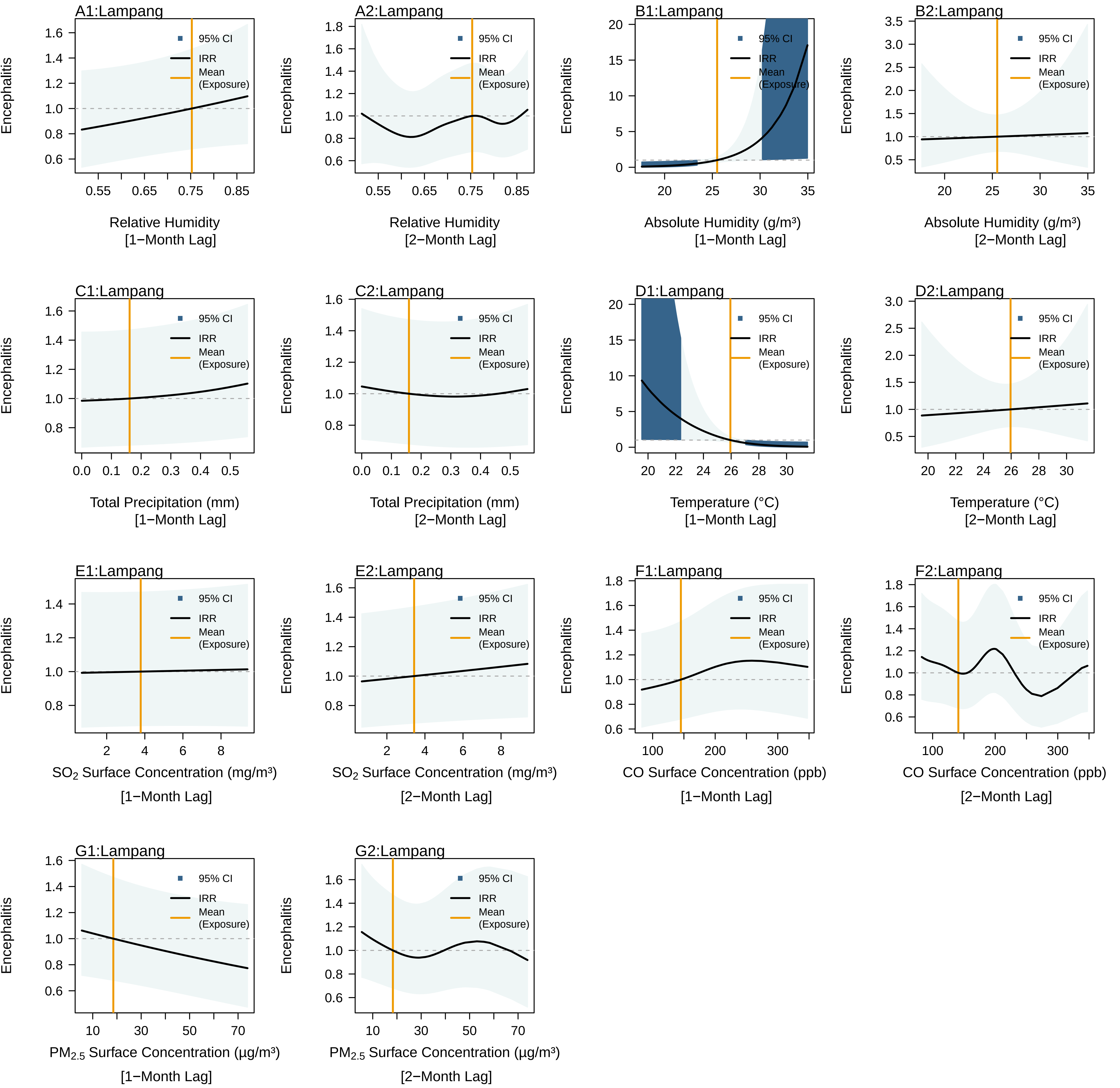
A1:Lampang
A2:Lampang
B1:Lampang
B2:Lampang
C1:Lampang
C2:Lampang
D1:Lampang
D2:Lampang
E1:Lampang
E2:Lampang
F1:Lampang
F2:Lampang
G1:Lampang
G2:Lampang
Encephalitis
95% CI
IRR
Mean (Exposure)
Relative Humidity [1−Month Lag]
Relative Humidity [2−Month Lag]
Absolute Humidity (g/m³) [1−Month Lag]
Absolute Humidity (g/m³) [2−Month Lag]
Total Precipitation (mm) [1−Month Lag]
Total Precipitation (mm) [2−Month Lag]
Temperature (°C) [1−Month Lag]
Temperature (°C) [2−Month Lag]
SO_2 Surface Concentration (mg/m³) [1−Month Lag]
SO_2 Surface Concentration (mg/m³) [2−Month Lag]
CO Surface Concentration (ppb) [1−Month Lag]
CO Surface Concentration (ppb) [2−Month Lag]
$PM_{2.5}$ Surface Concentration (µg/m³) [1−Month Lag]
$PM_{2.5}$ Surface Concentration (µg/m³) [2−Month Lag]

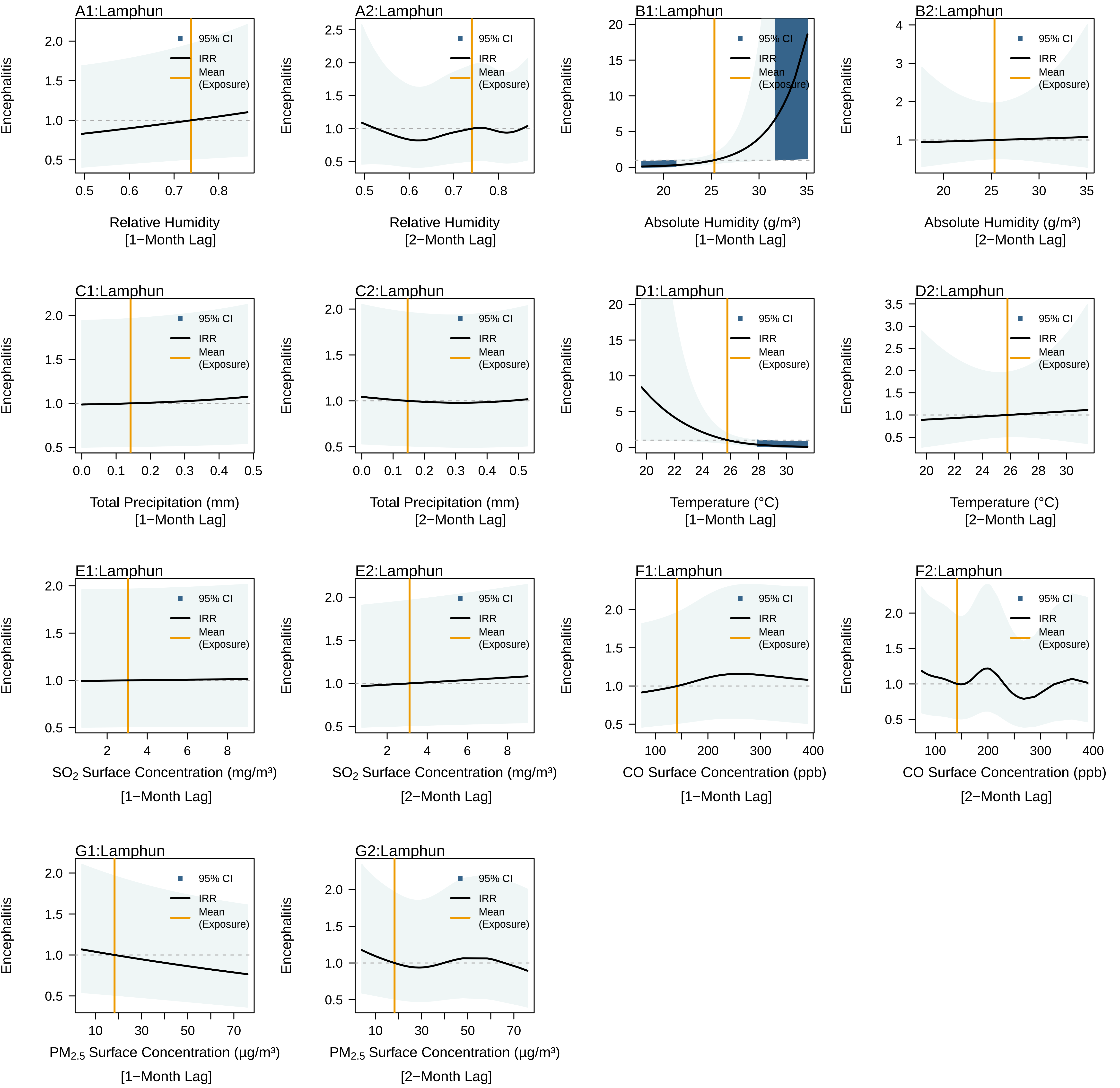

A1:Lamphun
A2:Lamphun
B1:Lamphun
B2:Lamphun
C1:Lamphun
C2:Lamphun
D1:Lamphun
D2:Lamphun
E1:Lamphun
E2:Lamphun
F1:Lamphun
F2:Lamphun
G1:Lamphun
G2:Lamphun
95% CI
IRR
Mean (Exposure)
Encephalitis
Relative Humidity [1−Month Lag]
Relative Humidity [2−Month Lag]
Absolute Humidity (g/m³) [1−Month Lag]
Absolute Humidity (g/m³) [2−Month Lag]
Total Precipitation (mm) [1−Month Lag]
Total Precipitation (mm) [2−Month Lag]
Temperature (°C) [1−Month Lag]
Temperature (°C) [2−Month Lag]
SO_2 Surface Concentration (mg/m³) [1−Month Lag]
SO_2 Surface Concentration (mg/m³) [2−Month Lag]
CO Surface Concentration (ppb) [1−Month Lag]
CO Surface Concentration (ppb) [2−Month Lag]
$PM_{2.5}$ Surface Concentration (µg/m³) [1−Month Lag]
$PM_{2.5}$ Surface Concentration (µg/m³) [2−Month Lag]

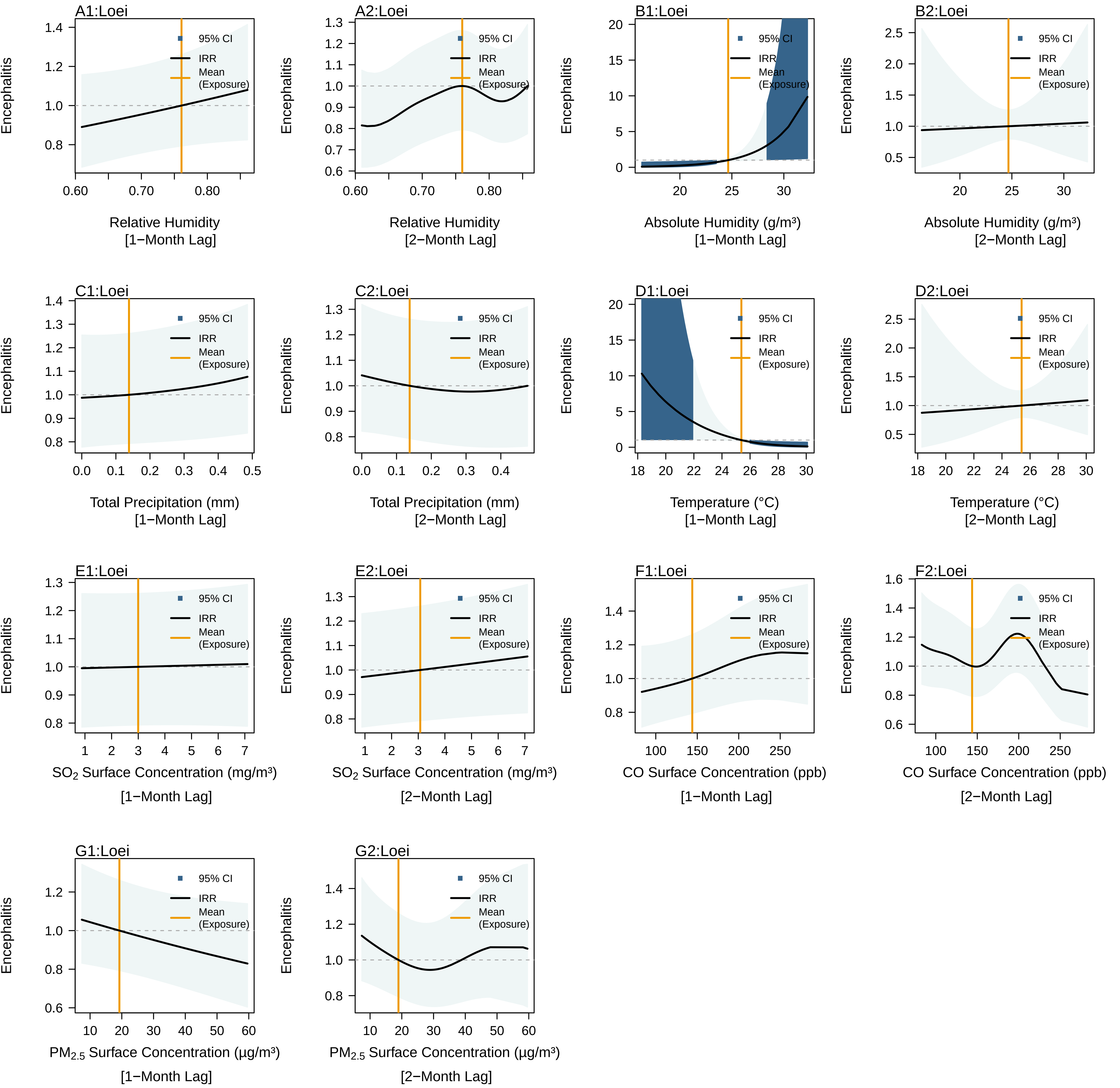
A1:Loei
A2:Loei
B1:Loei
B2:Loei
C1:Loei
C2:Loei
D1:Loei
D2:Loei
E1:Loei
E2:Loei
F1:Loei
F2:Loei
G1:Loei
G2:Loei
Encephalitis
95% CI
IRR
Mean (Exposure)
Relative Humidity [1−Month Lag]
Relative Humidity [2−Month Lag]
Absolute Humidity (g/m³) [1−Month Lag]
Absolute Humidity (g/m³) [2−Month Lag]
Total Precipitation (mm) [1−Month Lag]
Total Precipitation (mm) [2−Month Lag]
Temperature (°C) [1−Month Lag]
Temperature (°C) [2−Month Lag]
SO2 Surface Concentration (mg/m³) [1−Month Lag]
SO2 Surface Concentration (mg/m³) [2−Month Lag]
CO Surface Concentration (ppb) [1−Month Lag]
CO Surface Concentration (ppb) [2−Month Lag]
PM2.5 Surface Concentration (µg/m³) [1−Month Lag]
PM2.5 Surface Concentration (µg/m³) [2−Month Lag]

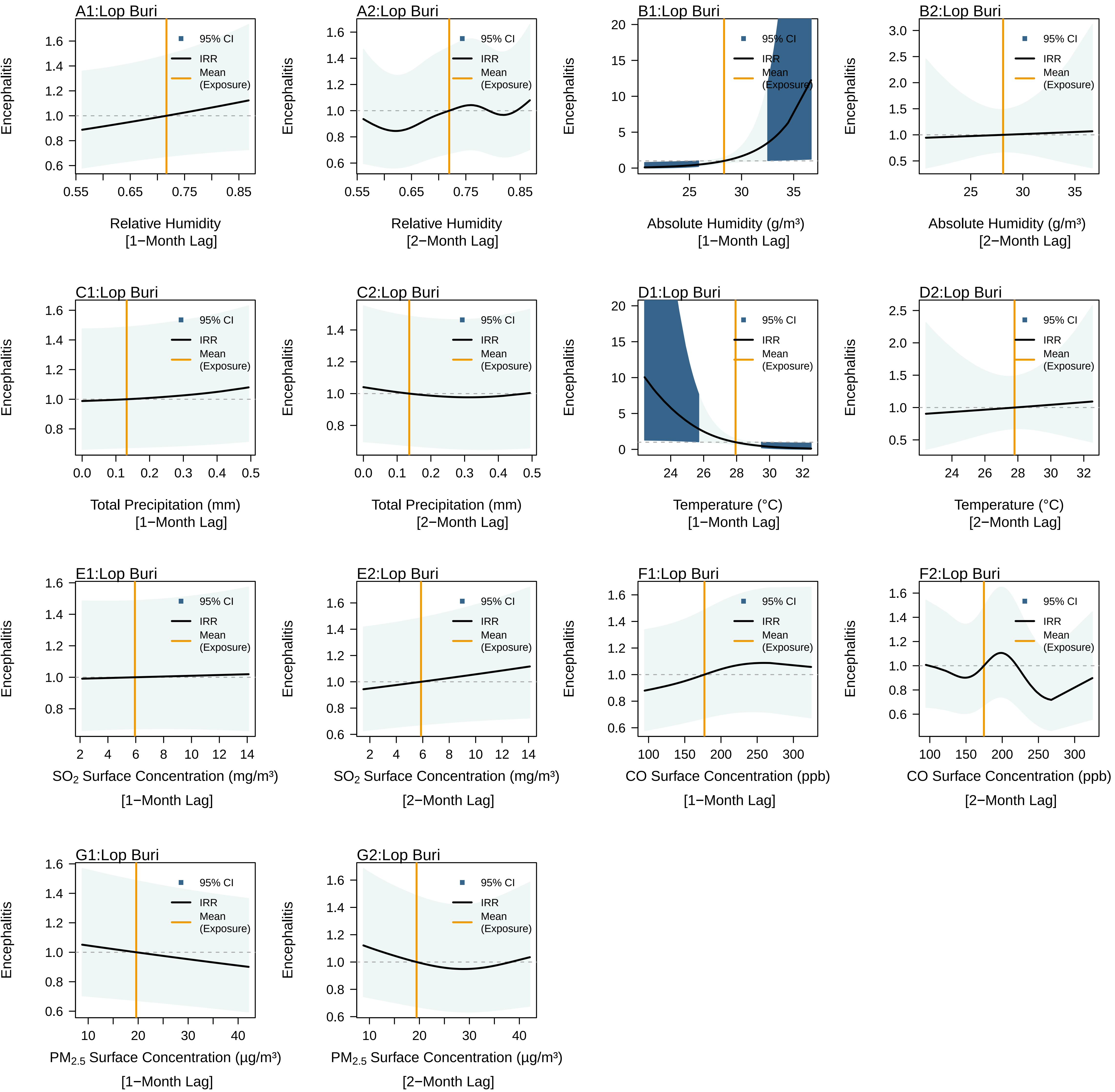
A1:Lop Buri
A2:Lop Buri
B1:Lop Buri
B2:Lop Buri
C1:Lop Buri
C2:Lop Buri
D1:Lop Buri
D2:Lop Buri
E1:Lop Buri
E2:Lop Buri
F1:Lop Buri
F2:Lop Buri
G1:Lop Buri
G2:Lop Buri
95% CI
IRR
Mean
(Exposure)
Encephalitis
Relative Humidity
[1−Month Lag]
[2−Month Lag]
Absolute Humidity (g/m³)
Total Precipitation (mm)
Temperature (°C)
SO2 Surface Concentration (mg/m³)
CO Surface Concentration (ppb)
PM2.5 Surface Concentration (µg/m³)

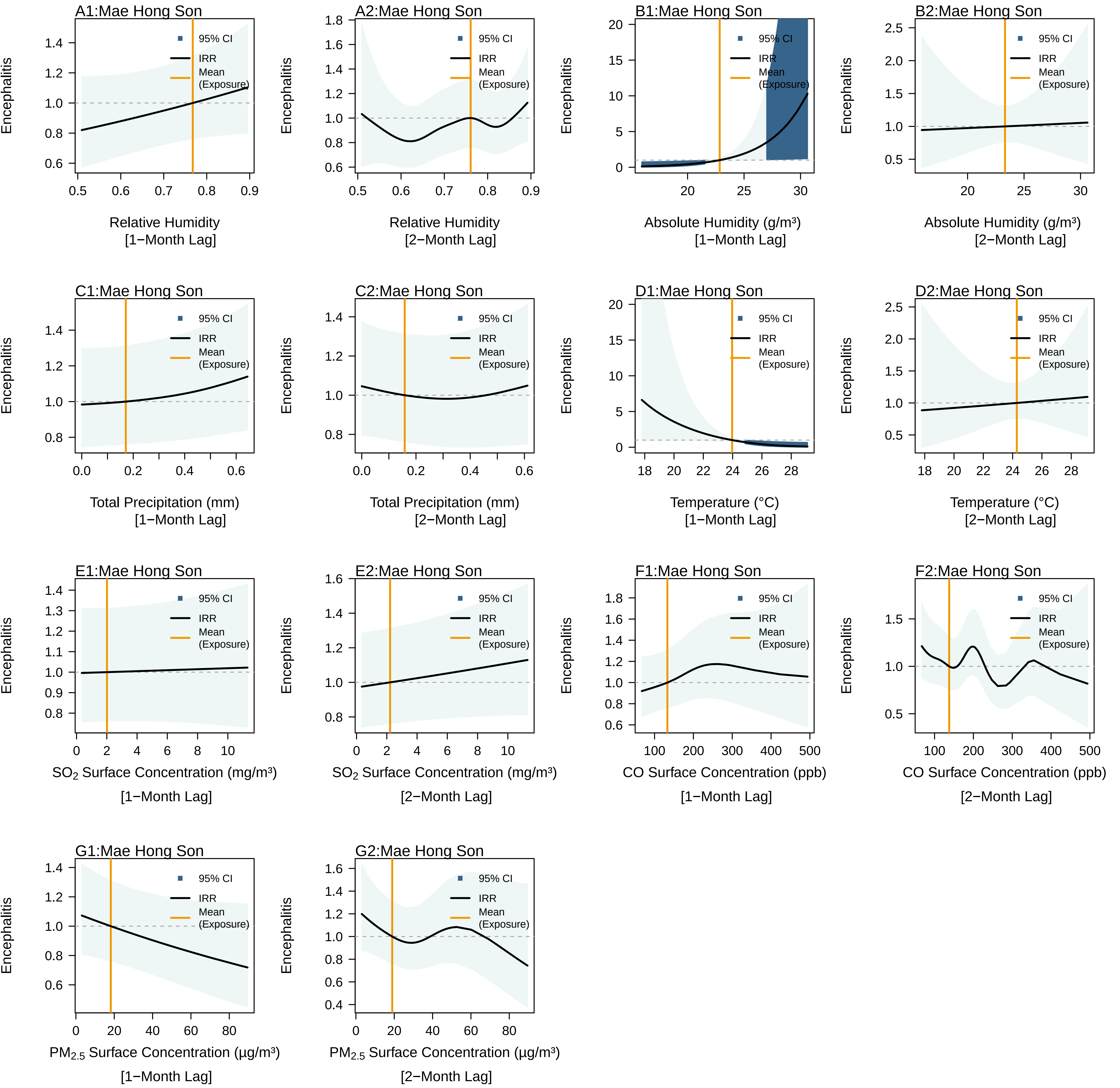
A1:Mae Hong Son
95% CI
IRR
Mean (Exposure)
Encephalitis
Relative Humidity [1−Month Lag]
A2:Mae Hong Son
Relative Humidity [2−Month Lag]
B1:Mae Hong Son
Absolute Humidity (g/m³) [1−Month Lag]
B2:Mae Hong Son
Absolute Humidity (g/m³) [2−Month Lag]
C1:Mae Hong Son
Total Precipitation (mm) [1−Month Lag]
C2:Mae Hong Son
Total Precipitation (mm) [2−Month Lag]
D1:Mae Hong Son
Temperature (°C) [1−Month Lag]
D2:Mae Hong Son
Temperature (°C) [2−Month Lag]
E1:Mae Hong Son
SO_2 Surface Concentration (mg/m³) [1−Month Lag]
E2:Mae Hong Son
SO_2 Surface Concentration (mg/m³) [2−Month Lag]
F1:Mae Hong Son
CO Surface Concentration (ppb) [1−Month Lag]
F2:Mae Hong Son
CO Surface Concentration (ppb) [2−Month Lag]
G1:Mae Hong Son
$PM_{2.5}$ Surface Concentration (µg/m³) [1−Month Lag]
G2:Mae Hong Son
$PM_{2.5}$ Surface Concentration (µg/m³) [2−Month Lag]

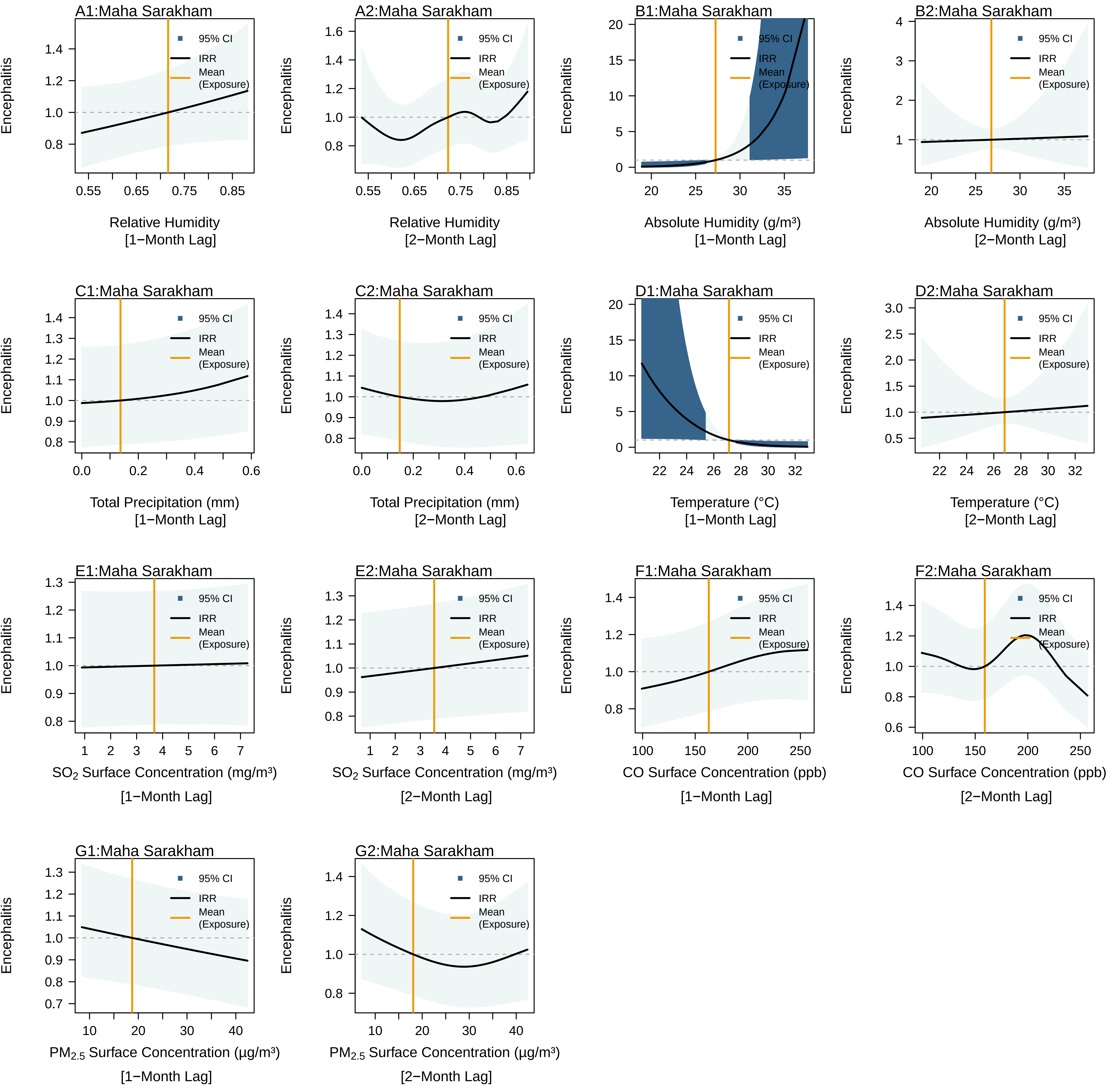

A1:Maha Sarakham
Encephalitis
95% CI
IRR
Mean (Exposure)
Relative Humidity [1−Month Lag]
A2:Maha Sarakham
Relative Humidity [2−Month Lag]
B1:Maha Sarakham
Absolute Humidity (g/m³) [1−Month Lag]
B2:Maha Sarakham
Absolute Humidity (g/m³) [2−Month Lag]
C1:Maha Sarakham
Total Precipitation (mm) [1−Month Lag]
C2:Maha Sarakham
Total Precipitation (mm) [2−Month Lag]
D1:Maha Sarakham
Temperature (°C) [1−Month Lag]
D2:Maha Sarakham
Temperature (°C) [2−Month Lag]
E1:Maha Sarakham
SO2 Surface Concentration (mg/m³) [1−Month Lag]
E2:Maha Sarakham
SO2 Surface Concentration (mg/m³) [2−Month Lag]
F1:Maha Sarakham
CO Surface Concentration (ppb) [1−Month Lag]
F2:Maha Sarakham
CO Surface Concentration (ppb) [2−Month Lag]
G1:Maha Sarakham
PM2.5 Surface Concentration (µg/m³) [1−Month Lag]
G2:Maha Sarakham
PM2.5 Surface Concentration (µg/m³) [2−Month Lag]

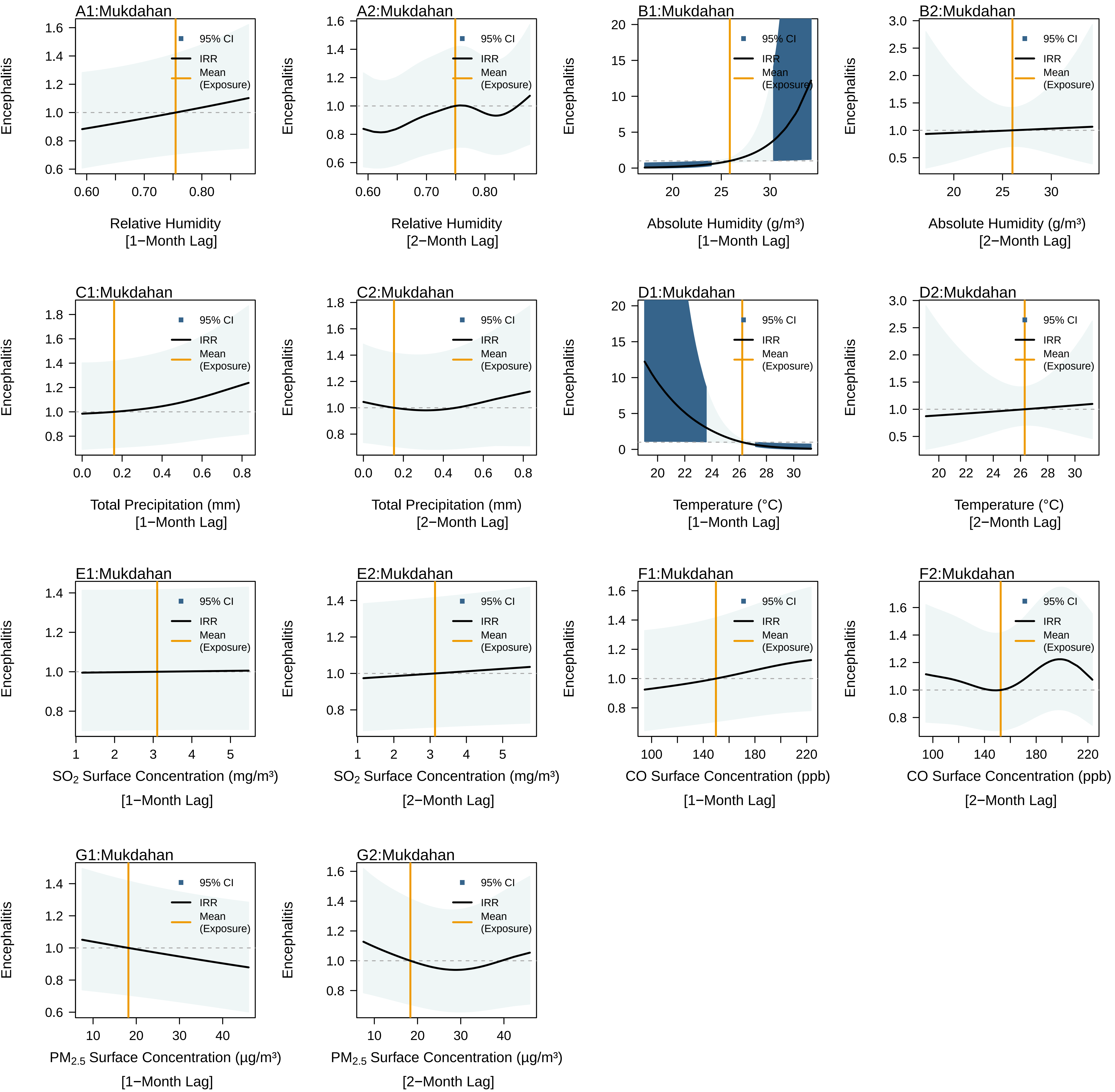
A1:Mukdahan
Encephalitis
Relative Humidity [1−Month Lag]
95% CI
IRR
Mean (Exposure)
A2:Mukdahan
Encephalitis
Relative Humidity [2−Month Lag]
B1:Mukdahan
Encephalitis
Absolute Humidity (g/m³) [1−Month Lag]
B2:Mukdahan
Encephalitis
Absolute Humidity (g/m³) [2−Month Lag]
C1:Mukdahan
Encephalitis
Total Precipitation (mm) [1−Month Lag]
C2:Mukdahan
Encephalitis
Total Precipitation (mm) [2−Month Lag]
D1:Mukdahan
Encephalitis
Temperature (°C) [1−Month Lag]
D2:Mukdahan
Encephalitis
Temperature (°C) [2−Month Lag]
E1:Mukdahan
Encephalitis
SO_2 Surface Concentration (mg/m³) [1−Month Lag]
E2:Mukdahan
Encephalitis
SO_2 Surface Concentration (mg/m³) [2−Month Lag]
F1:Mukdahan
Encephalitis
CO Surface Concentration (ppb) [1−Month Lag]
F2:Mukdahan
Encephalitis
CO Surface Concentration (ppb) [2−Month Lag]
G1:Mukdahan
Encephalitis
$PM_{2.5}$ Surface Concentration (µg/m³) [1−Month Lag]
G2:Mukdahan
Encephalitis
$PM_{2.5}$ Surface Concentration (µg/m³) [2−Month Lag]

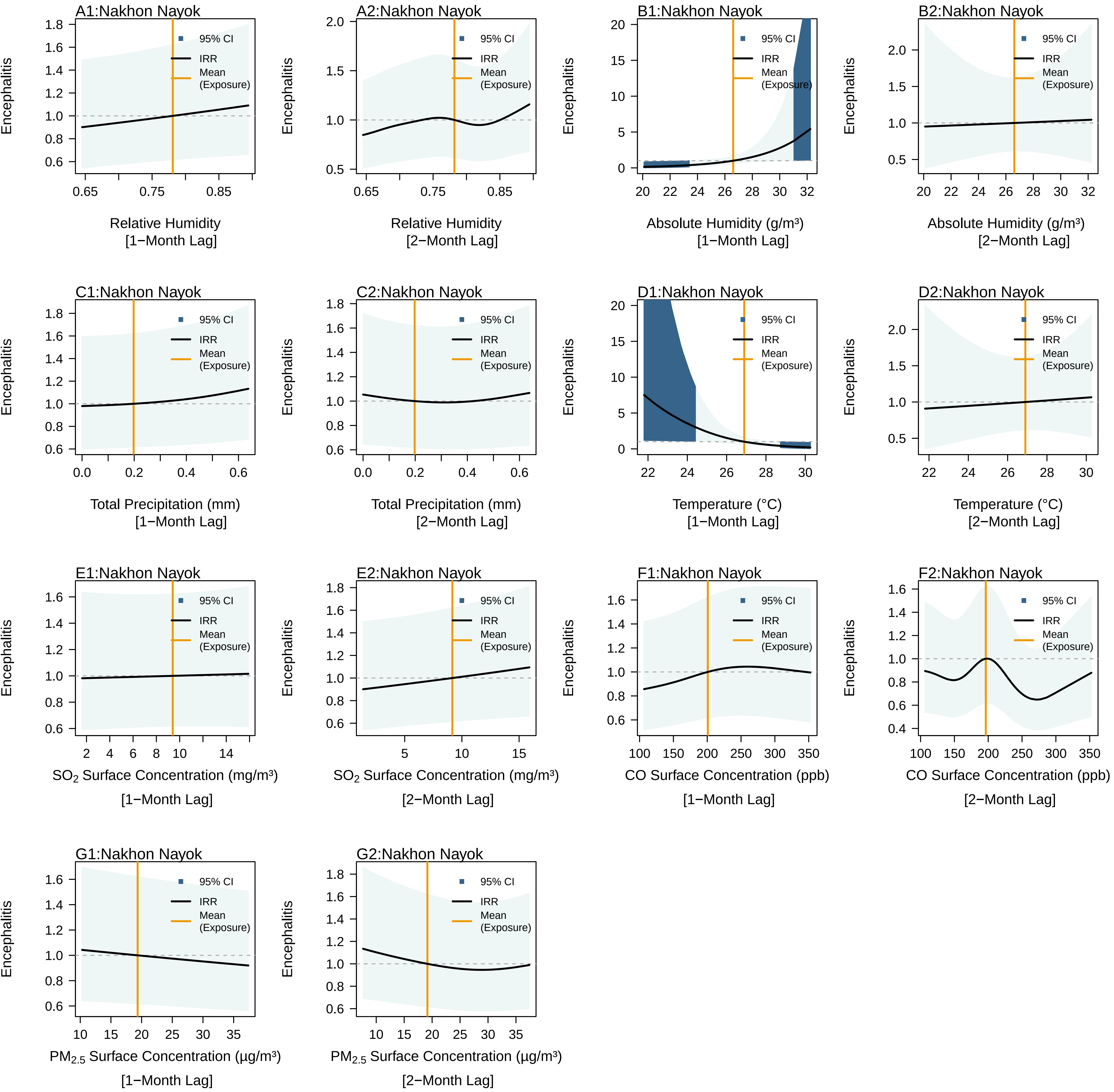
A1:Nakhon Nayok
95% CI
IRR
Mean (Exposure)
Encephalitis
Relative Humidity [1−Month Lag]
A2:Nakhon Nayok
95% CI
IRR
Mean (Exposure)
Encephalitis
Relative Humidity [2−Month Lag]
B1:Nakhon Nayok
95% CI
IRR
Mean (Exposure)
Encephalitis
Absolute Humidity (g/m³) [1−Month Lag]
B2:Nakhon Nayok
95% CI
IRR
Mean (Exposure)
Encephalitis
Absolute Humidity (g/m³) [2−Month Lag]
C1:Nakhon Nayok
95% CI
IRR
Mean (Exposure)
Encephalitis
Total Precipitation (mm) [1−Month Lag]
C2:Nakhon Nayok
95% CI
IRR
Mean (Exposure)
Encephalitis
Total Precipitation (mm) [2−Month Lag]
D1:Nakhon Nayok
95% CI
IRR
Mean (Exposure)
Encephalitis
Temperature (°C) [1−Month Lag]
D2:Nakhon Nayok
95% CI
IRR
Mean (Exposure)
Encephalitis
Temperature (°C) [2−Month Lag]
E1:Nakhon Nayok
95% CI
IRR
Mean (Exposure)
Encephalitis
SO_2 Surface Concentration (mg/m³) [1−Month Lag]
E2:Nakhon Nayok
95% CI
IRR
Mean (Exposure)
Encephalitis
SO_2 Surface Concentration (mg/m³) [2−Month Lag]
F1:Nakhon Nayok
95% CI
IRR
Mean (Exposure)
Encephalitis
CO Surface Concentration (ppb) [1−Month Lag]
F2:Nakhon Nayok
95% CI
IRR
Mean (Exposure)
Encephalitis
CO Surface Concentration (ppb) [2−Month Lag]
G1:Nakhon Nayok
95% CI
IRR
Mean (Exposure)
Encephalitis
$PM_{2.5}$ Surface Concentration (µg/m³) [1−Month Lag]
G2:Nakhon Nayok
95% CI
IRR
Mean (Exposure)
Encephalitis
$PM_{2.5}$ Surface Concentration (µg/m³) [2−Month Lag]

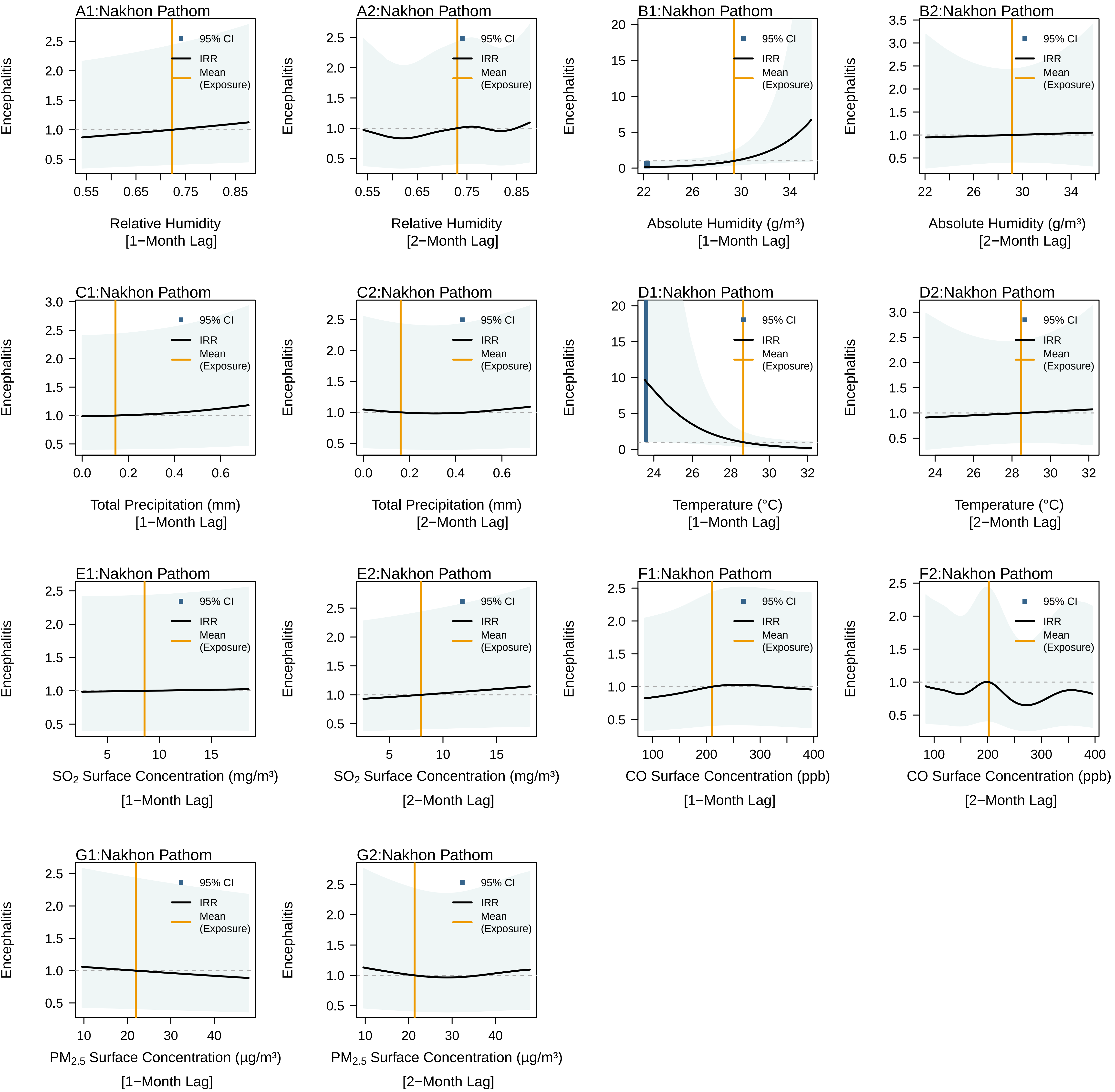
A1:Nakhon Pathom
A2:Nakhon Pathom
B1:Nakhon Pathom
B2:Nakhon Pathom
C1:Nakhon Pathom
C2:Nakhon Pathom
D1:Nakhon Pathom
D2:Nakhon Pathom
E1:Nakhon Pathom
E2:Nakhon Pathom
F1:Nakhon Pathom
F2:Nakhon Pathom
G1:Nakhon Pathom
G2:Nakhon Pathom
Encephalitis
95% CI
IRR
Mean (Exposure)
Relative Humidity [1−Month Lag]
Relative Humidity [2−Month Lag]
Absolute Humidity (g/m³) [1−Month Lag]
Absolute Humidity (g/m³) [2−Month Lag]
Total Precipitation (mm) [1−Month Lag]
Total Precipitation (mm) [2−Month Lag]
Temperature (°C) [1−Month Lag]
Temperature (°C) [2−Month Lag]
SO_2 Surface Concentration (mg/m³) [1−Month Lag]
SO_2 Surface Concentration (mg/m³) [2−Month Lag]
CO Surface Concentration (ppb) [1−Month Lag]
CO Surface Concentration (ppb) [2−Month Lag]
$PM_{2.5}$ Surface Concentration (µg/m³) [1−Month Lag]
$PM_{2.5}$ Surface Concentration (µg/m³) [2−Month Lag]

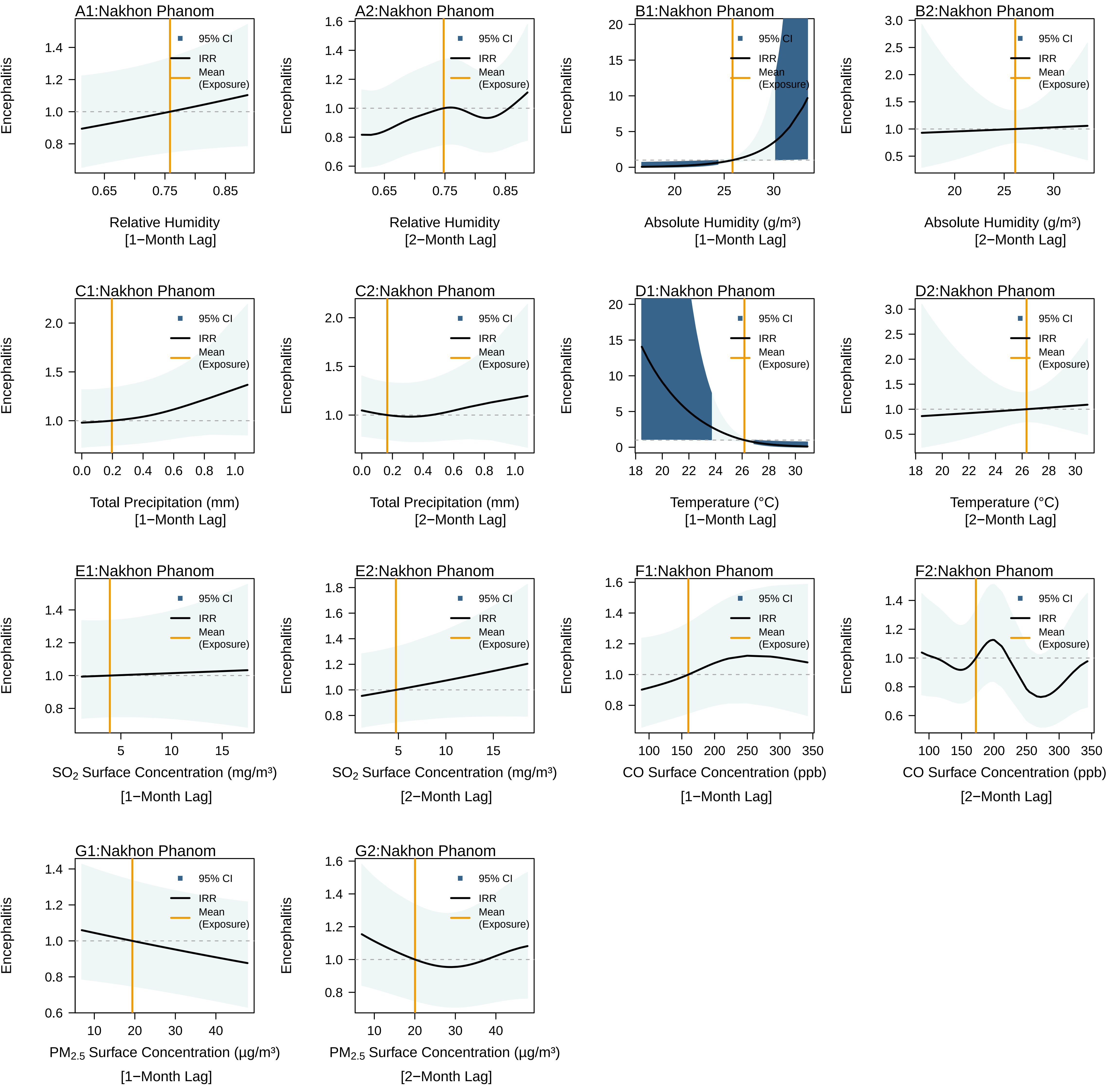
A1:Nakhon Phanom
95% CI
IRR
Mean (Exposure)
Encephalitis
Relative Humidity [1−Month Lag]
A2:Nakhon Phanom
Relative Humidity [2−Month Lag]
B1:Nakhon Phanom
Absolute Humidity (g/m³) [1−Month Lag]
B2:Nakhon Phanom
Absolute Humidity (g/m³) [2−Month Lag]
C1:Nakhon Phanom
Total Precipitation (mm) [1−Month Lag]
C2:Nakhon Phanom
Total Precipitation (mm) [2−Month Lag]
D1:Nakhon Phanom
Temperature (°C) [1−Month Lag]
D2:Nakhon Phanom
Temperature (°C) [2−Month Lag]
E1:Nakhon Phanom
SO_2 Surface Concentration (mg/m³) [1−Month Lag]
E2:Nakhon Phanom
SO_2 Surface Concentration (mg/m³) [2−Month Lag]
F1:Nakhon Phanom
CO Surface Concentration (ppb) [1−Month Lag]
F2:Nakhon Phanom
CO Surface Concentration (ppb) [2−Month Lag]
G1:Nakhon Phanom
$PM_{2.5}$ Surface Concentration (µg/m³) [1−Month Lag]
G2:Nakhon Phanom
$PM_{2.5}$ Surface Concentration (µg/m³) [2−Month Lag]

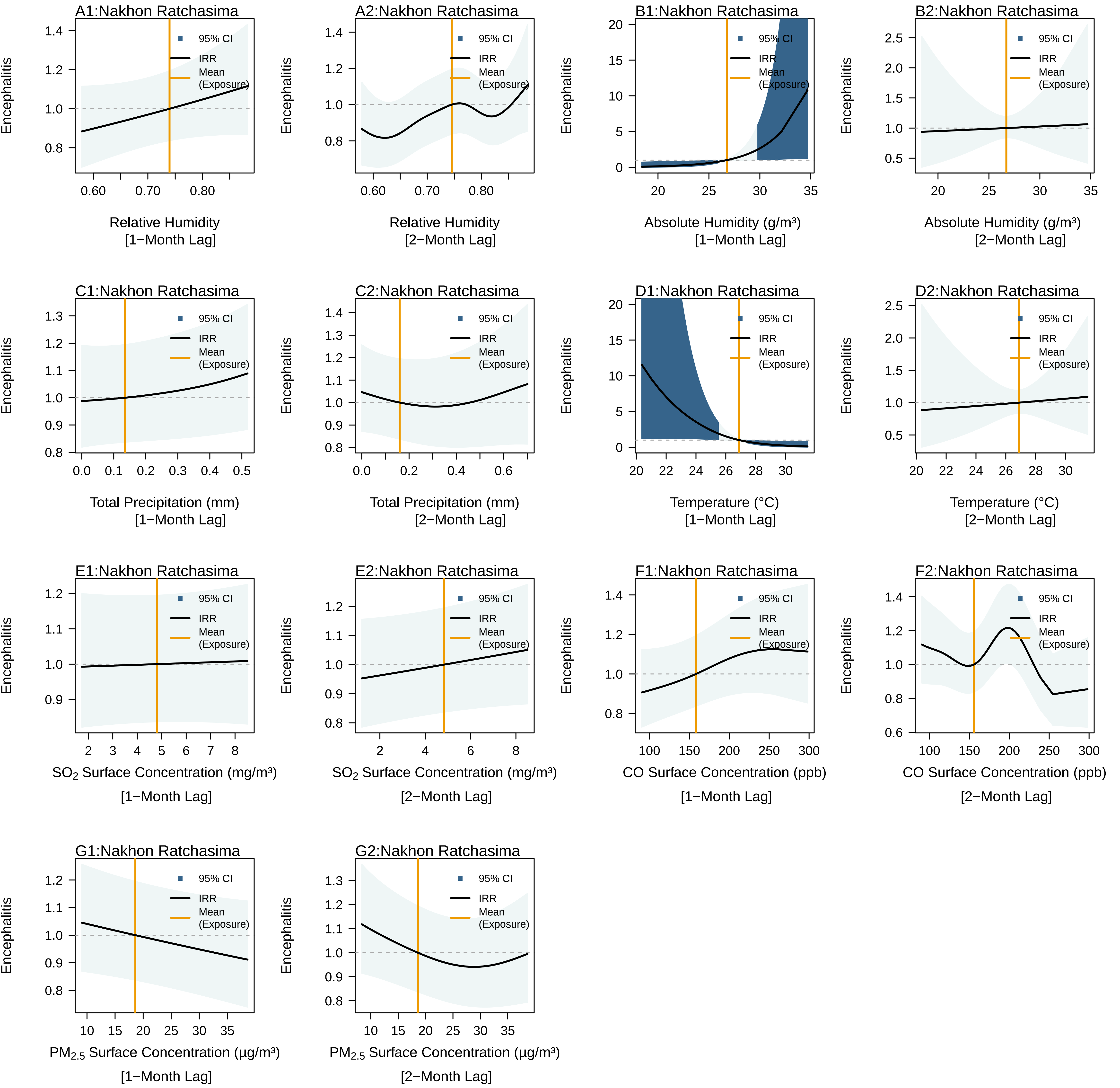
A1:Nakhon Ratchasima
Relative Humidity [1−Month Lag]
A2:Nakhon Ratchasima
Relative Humidity [2−Month Lag]
B1:Nakhon Ratchasima
Absolute Humidity (g/m³) [1−Month Lag]
B2:Nakhon Ratchasima
Absolute Humidity (g/m³) [2−Month Lag]
C1:Nakhon Ratchasima
Total Precipitation (mm) [1−Month Lag]
C2:Nakhon Ratchasima
Total Precipitation (mm) [2−Month Lag]
D1:Nakhon Ratchasima
Temperature (°C) [1−Month Lag]
D2:Nakhon Ratchasima
Temperature (°C) [2−Month Lag]
E1:Nakhon Ratchasima
SO_2 Surface Concentration (mg/m³) [1−Month Lag]
E2:Nakhon Ratchasima
SO_2 Surface Concentration (mg/m³) [2−Month Lag]
F1:Nakhon Ratchasima
CO Surface Concentration (ppb) [1−Month Lag]
F2:Nakhon Ratchasima
CO Surface Concentration (ppb) [2−Month Lag]
G1:Nakhon Ratchasima
$PM_{2.5}$ Surface Concentration (µg/m³) [1−Month Lag]
G2:Nakhon Ratchasima
$PM_{2.5}$ Surface Concentration (µg/m³) [2−Month Lag]
Encephalitis
95% CI
IRR
Mean (Exposure)

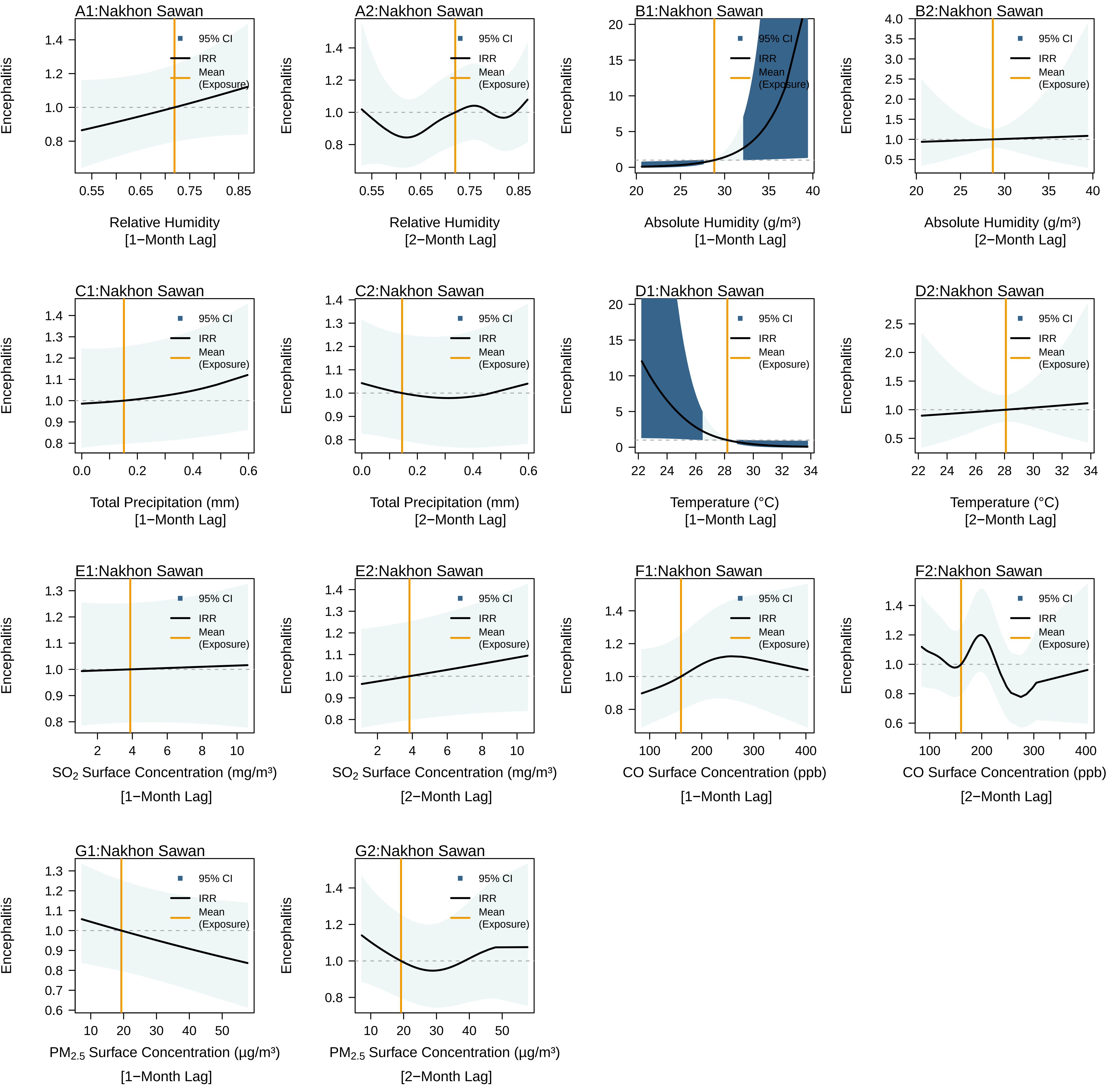

A1:Nakhon Sawan
95% CI
IRR
Mean (Exposure)
Encephalitis
Relative Humidity [1−Month Lag]
A2:Nakhon Sawan
95% CI
IRR
Mean (Exposure)
Encephalitis
Relative Humidity [2−Month Lag]
B1:Nakhon Sawan
95% CI
IRR
Mean (Exposure)
Encephalitis
Absolute Humidity (g/m³) [1−Month Lag]
B2:Nakhon Sawan
95% CI
IRR
Mean (Exposure)
Encephalitis
Absolute Humidity (g/m³) [2−Month Lag]
C1:Nakhon Sawan
95% CI
IRR
Mean (Exposure)
Encephalitis
Total Precipitation (mm) [1−Month Lag]
C2:Nakhon Sawan
95% CI
IRR
Mean (Exposure)
Encephalitis
Total Precipitation (mm) [2−Month Lag]
D1:Nakhon Sawan
95% CI
IRR
Mean (Exposure)
Encephalitis
Temperature (°C) [1−Month Lag]
D2:Nakhon Sawan
95% CI
IRR
Mean (Exposure)
Encephalitis
Temperature (°C) [2−Month Lag]
E1:Nakhon Sawan
95% CI
IRR
Mean (Exposure)
Encephalitis
SO_2 Surface Concentration (mg/m³) [1−Month Lag]
E2:Nakhon Sawan
95% CI
IRR
Mean (Exposure)
Encephalitis
SO_2 Surface Concentration (mg/m³) [2−Month Lag]
F1:Nakhon Sawan
95% CI
IRR
Mean (Exposure)
Encephalitis
CO Surface Concentration (ppb) [1−Month Lag]
F2:Nakhon Sawan
95% CI
IRR
Mean (Exposure)
Encephalitis
CO Surface Concentration (ppb) [2−Month Lag]
G1:Nakhon Sawan
95% CI
IRR
Mean (Exposure)
Encephalitis
$PM_{2.5}$ Surface Concentration (µg/m³) [1−Month Lag]
G2:Nakhon Sawan
95% CI
IRR
Mean (Exposure)
Encephalitis
$PM_{2.5}$ Surface Concentration (µg/m³) [2−Month Lag]

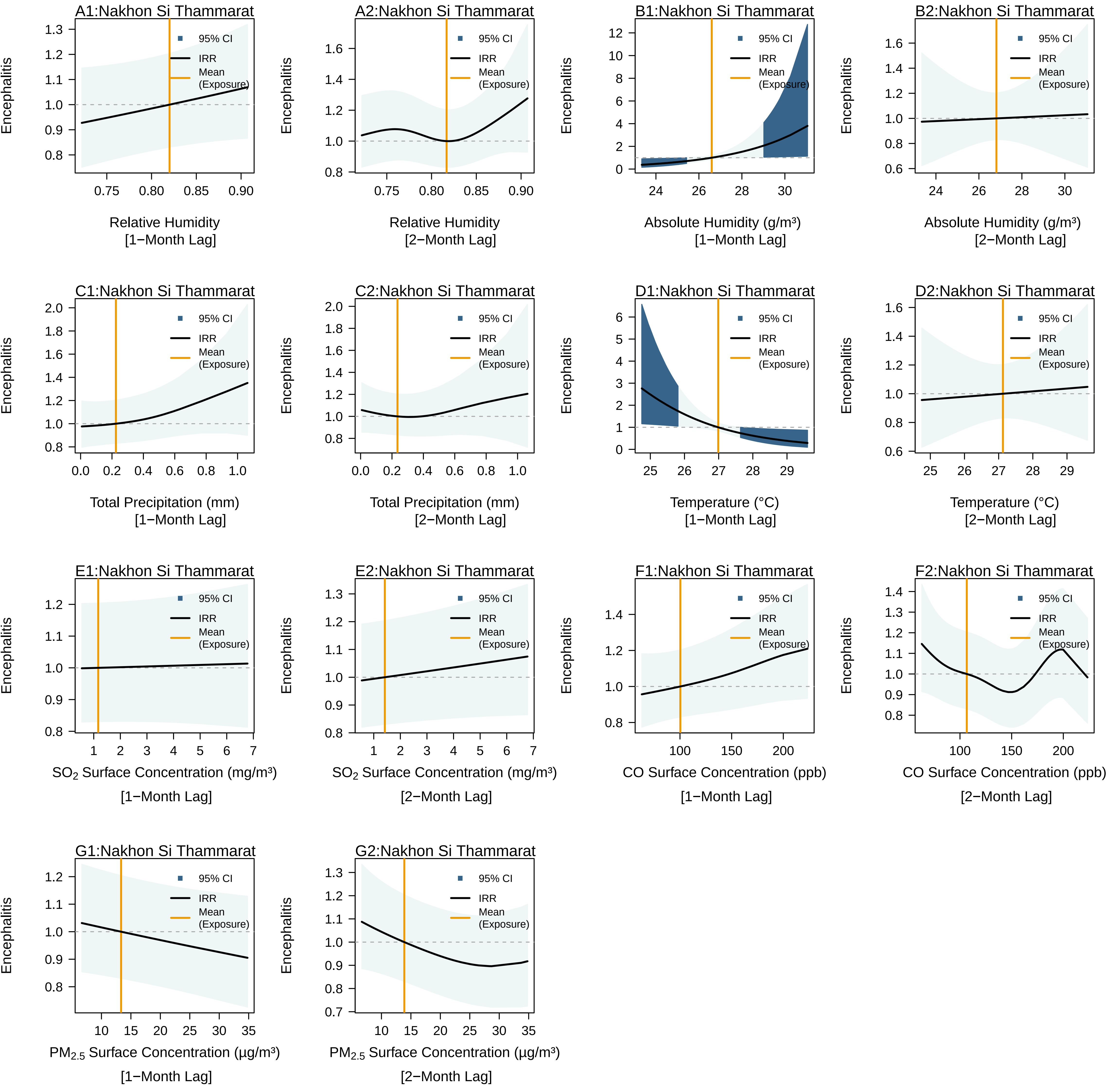
A1:Nakhon Si Thammarat
Encephalitis
95% CI
IRR
Mean (Exposure)
Relative Humidity [1−Month Lag]
A2:Nakhon Si Thammarat
Relative Humidity [2−Month Lag]
B1:Nakhon Si Thammarat
Absolute Humidity (g/m³) [1−Month Lag]
B2:Nakhon Si Thammarat
Absolute Humidity (g/m³) [2−Month Lag]
C1:Nakhon Si Thammarat
Total Precipitation (mm) [1−Month Lag]
C2:Nakhon Si Thammarat
Total Precipitation (mm) [2−Month Lag]
D1:Nakhon Si Thammarat
Temperature (°C) [1−Month Lag]
D2:Nakhon Si Thammarat
Temperature (°C) [2−Month Lag]
E1:Nakhon Si Thammarat
SO2 Surface Concentration (mg/m³) [1−Month Lag]
E2:Nakhon Si Thammarat
SO2 Surface Concentration (mg/m³) [2−Month Lag]
F1:Nakhon Si Thammarat
CO Surface Concentration (ppb) [1−Month Lag]
F2:Nakhon Si Thammarat
CO Surface Concentration (ppb) [2−Month Lag]
G1:Nakhon Si Thammarat
PM2.5 Surface Concentration (µg/m³) [1−Month Lag]
G2:Nakhon Si Thammarat
PM2.5 Surface Concentration (µg/m³) [2−Month Lag]

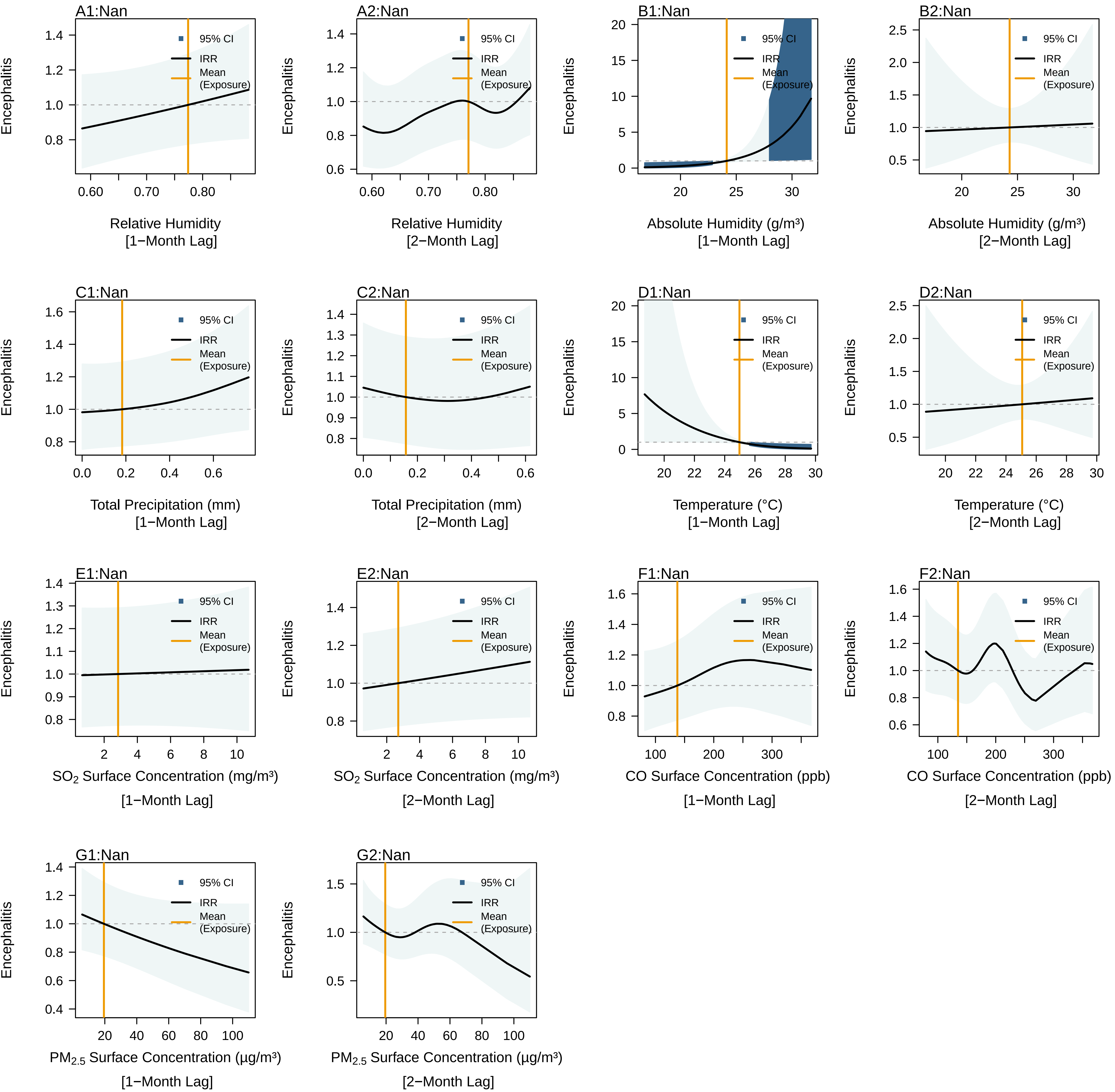

A1:Nan
A2:Nan
B1:Nan
B2:Nan
C1:Nan
C2:Nan
D1:Nan
D2:Nan
E1:Nan
E2:Nan
F1:Nan
F2:Nan
G1:Nan
G2:Nan
Encephalitis
95% CI
IRR
Mean (Exposure)
Relative Humidity [1−Month Lag]
Relative Humidity [2−Month Lag]
Absolute Humidity (g/m³) [1−Month Lag]
Absolute Humidity (g/m³) [2−Month Lag]
Total Precipitation (mm) [1−Month Lag]
Total Precipitation (mm) [2−Month Lag]
Temperature (°C) [1−Month Lag]
Temperature (°C) [2−Month Lag]
SO2 Surface Concentration (mg/m³) [1−Month Lag]
SO2 Surface Concentration (mg/m³) [2−Month Lag]
CO Surface Concentration (ppb) [1−Month Lag]
CO Surface Concentration (ppb) [2−Month Lag]
PM2.5 Surface Concentration (µg/m³) [1−Month Lag]
PM2.5 Surface Concentration (µg/m³) [2−Month Lag]

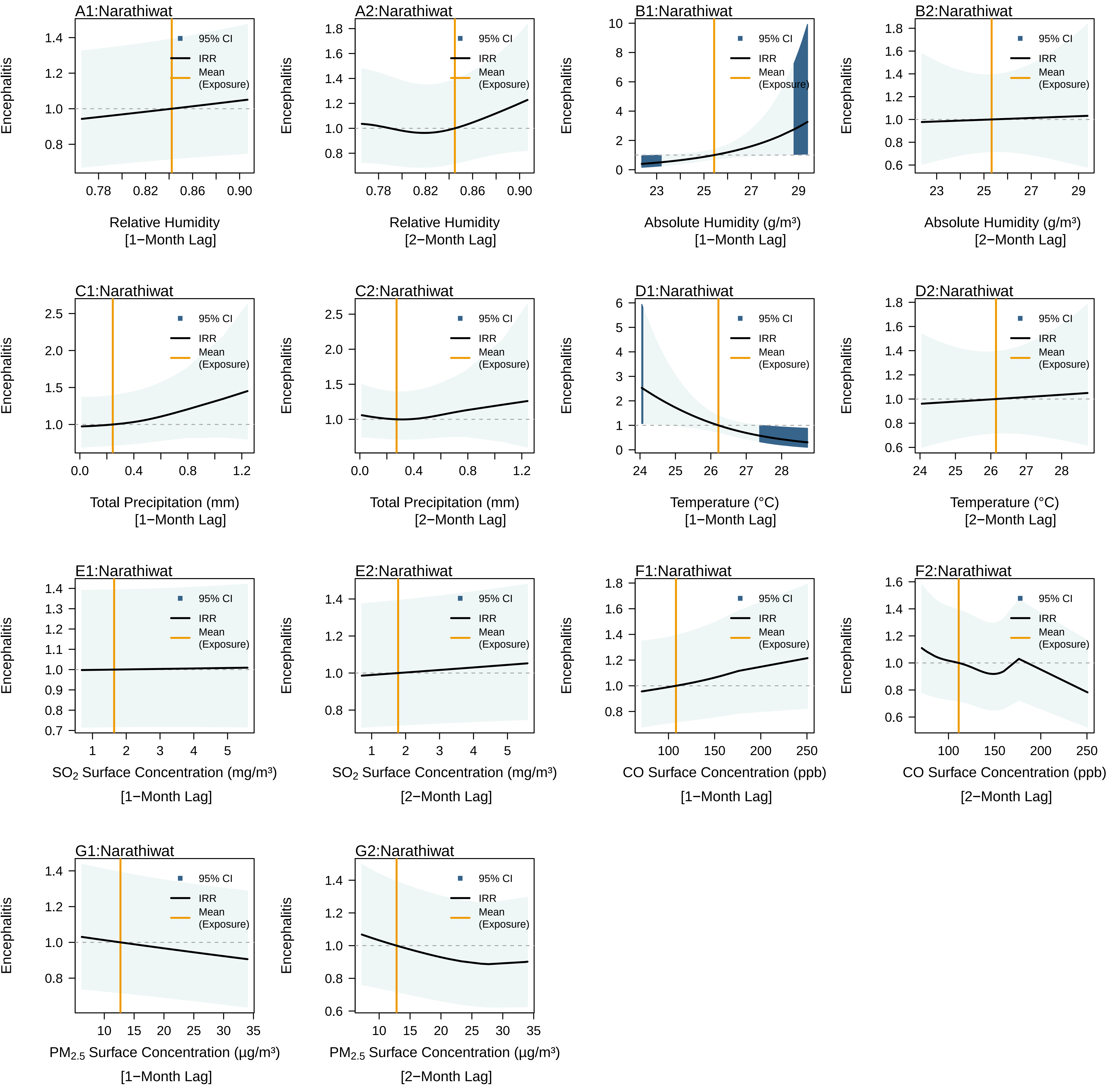

A1:Narathiwat
A2:Narathiwat
B1:Narathiwat
B2:Narathiwat
95% CI
IRR
Mean (Exposure)
Encephalitis
Relative Humidity [1−Month Lag]
Relative Humidity [2−Month Lag]
Absolute Humidity (g/m³) [1−Month Lag]
Absolute Humidity (g/m³) [2−Month Lag]
C1:Narathiwat
C2:Narathiwat
D1:Narathiwat
D2:Narathiwat
Total Precipitation (mm) [1−Month Lag]
Total Precipitation (mm) [2−Month Lag]
Temperature (°C) [1−Month Lag]
Temperature (°C) [2−Month Lag]
E1:Narathiwat
E2:Narathiwat
F1:Narathiwat
F2:Narathiwat
SO_2 Surface Concentration (mg/m³) [1−Month Lag]
SO_2 Surface Concentration (mg/m³) [2−Month Lag]
CO Surface Concentration (ppb) [1−Month Lag]
CO Surface Concentration (ppb) [2−Month Lag]
G1:Narathiwat
G2:Narathiwat
$PM_{2.5}$ Surface Concentration (µg/m³) [1−Month Lag]
$PM_{2.5}$ Surface Concentration (µg/m³) [2−Month Lag]

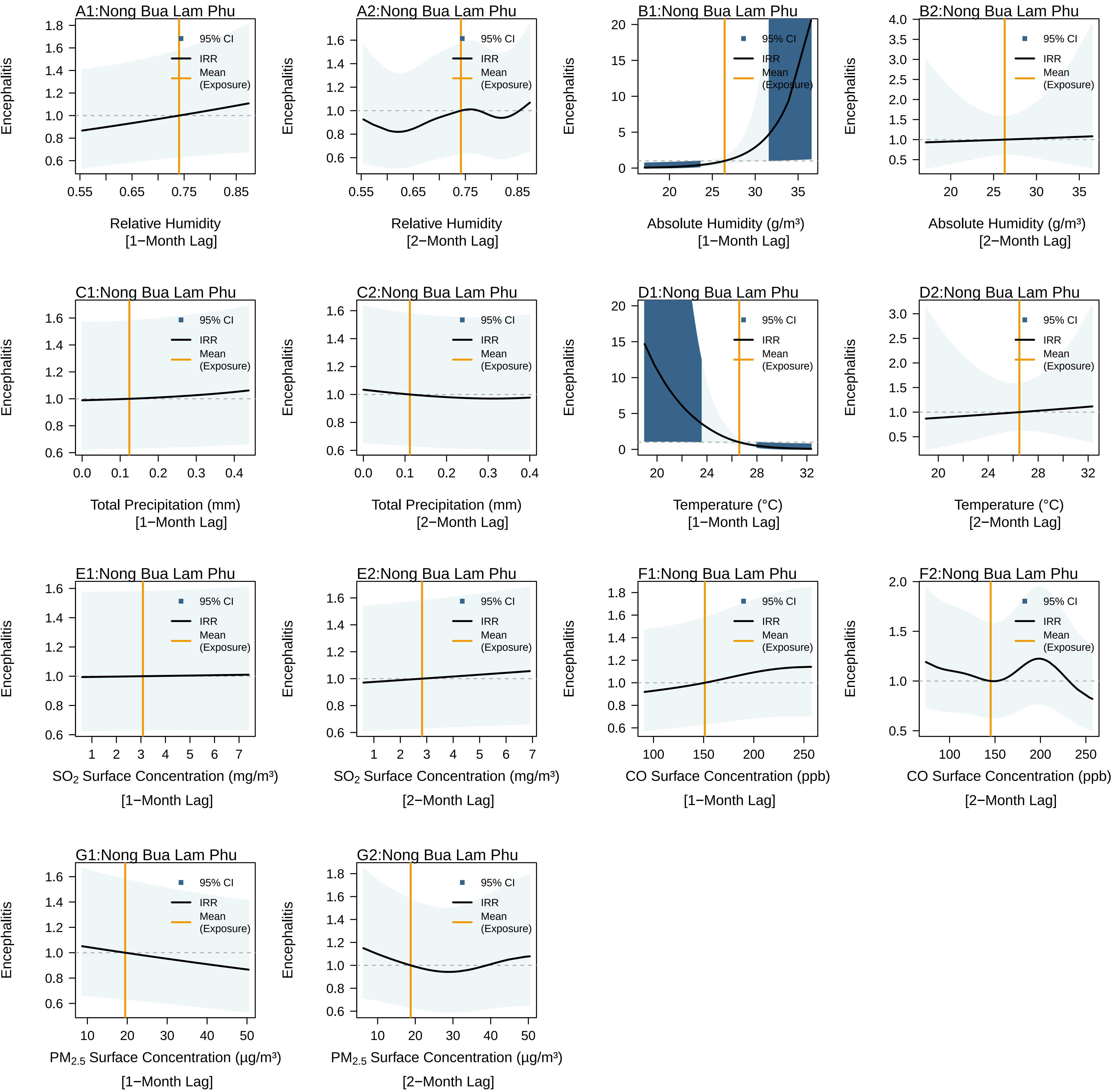
A1:Nong Bua Lam Phu
Encephalitis
95% CI
IRR
Mean (Exposure)
Relative Humidity [1−Month Lag]
A2:Nong Bua Lam Phu
Encephalitis
95% CI
IRR
Mean (Exposure)
Relative Humidity [2−Month Lag]
B1:Nong Bua Lam Phu
Encephalitis
95% CI
IRR
Mean (Exposure)
Absolute Humidity (g/m³) [1−Month Lag]
B2:Nong Bua Lam Phu
Encephalitis
95% CI
IRR
Mean (Exposure)
Absolute Humidity (g/m³) [2−Month Lag]
C1:Nong Bua Lam Phu
Encephalitis
95% CI
IRR
Mean (Exposure)
Total Precipitation (mm) [1−Month Lag]
C2:Nong Bua Lam Phu
Encephalitis
95% CI
IRR
Mean (Exposure)
Total Precipitation (mm) [2−Month Lag]
D1:Nong Bua Lam Phu
Encephalitis
95% CI
IRR
Mean (Exposure)
Temperature (°C) [1−Month Lag]
D2:Nong Bua Lam Phu
Encephalitis
95% CI
IRR
Mean (Exposure)
Temperature (°C) [2−Month Lag]
E1:Nong Bua Lam Phu
Encephalitis
95% CI
IRR
Mean (Exposure)
SO_2 Surface Concentration (mg/m³) [1−Month Lag]
E2:Nong Bua Lam Phu
Encephalitis
95% CI
IRR
Mean (Exposure)
SO_2 Surface Concentration (mg/m³) [2−Month Lag]
F1:Nong Bua Lam Phu
Encephalitis
95% CI
IRR
Mean (Exposure)
CO Surface Concentration (ppb) [1−Month Lag]
F2:Nong Bua Lam Phu
Encephalitis
95% CI
IRR
Mean (Exposure)
CO Surface Concentration (ppb) [2−Month Lag]
G1:Nong Bua Lam Phu
Encephalitis
95% CI
IRR
Mean (Exposure)
$PM_{2.5}$ Surface Concentration (µg/m³) [1−Month Lag]
G2:Nong Bua Lam Phu
Encephalitis
95% CI
IRR
Mean (Exposure)
$PM_{2.5}$ Surface Concentration (µg/m³) [2−Month Lag]

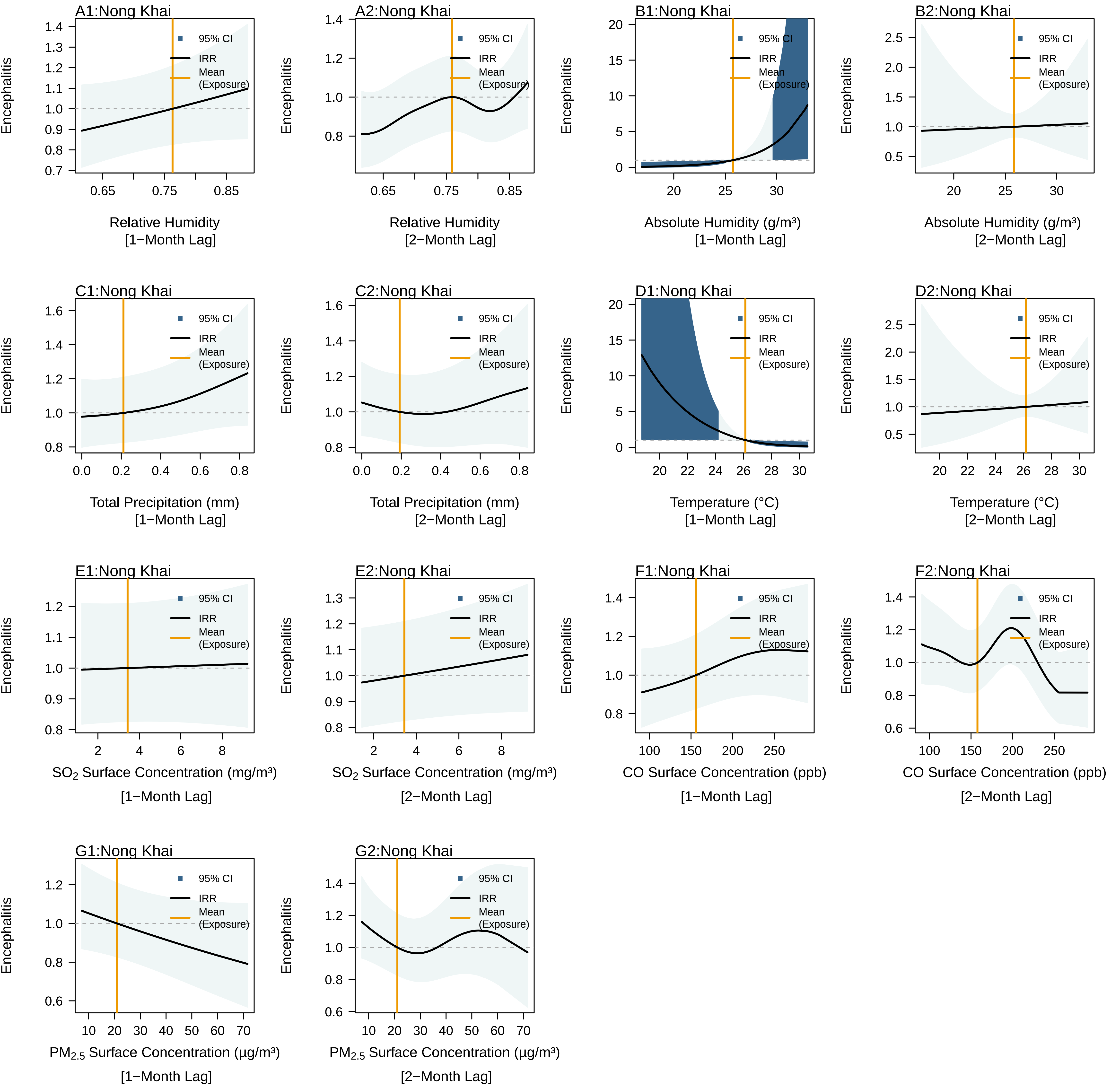

A1:Nong Khai
A2:Nong Khai
B1:Nong Khai
B2:Nong Khai
C1:Nong Khai
C2:Nong Khai
D1:Nong Khai
D2:Nong Khai
E1:Nong Khai
E2:Nong Khai
F1:Nong Khai
F2:Nong Khai
G1:Nong Khai
G2:Nong Khai
Encephalitis
95% CI
IRR
Mean (Exposure)
Relative Humidity [1−Month Lag]
Relative Humidity [2−Month Lag]
Absolute Humidity (g/m³) [1−Month Lag]
Absolute Humidity (g/m³) [2−Month Lag]
Total Precipitation (mm) [1−Month Lag]
Total Precipitation (mm) [2−Month Lag]
Temperature (°C) [1−Month Lag]
Temperature (°C) [2−Month Lag]
SO_2 Surface Concentration (mg/m³) [1−Month Lag]
SO_2 Surface Concentration (mg/m³) [2−Month Lag]
CO Surface Concentration (ppb) [1−Month Lag]
CO Surface Concentration (ppb) [2−Month Lag]
$PM_{2.5}$ Surface Concentration (µg/m³) [1−Month Lag]
$PM_{2.5}$ Surface Concentration (µg/m³) [2−Month Lag]

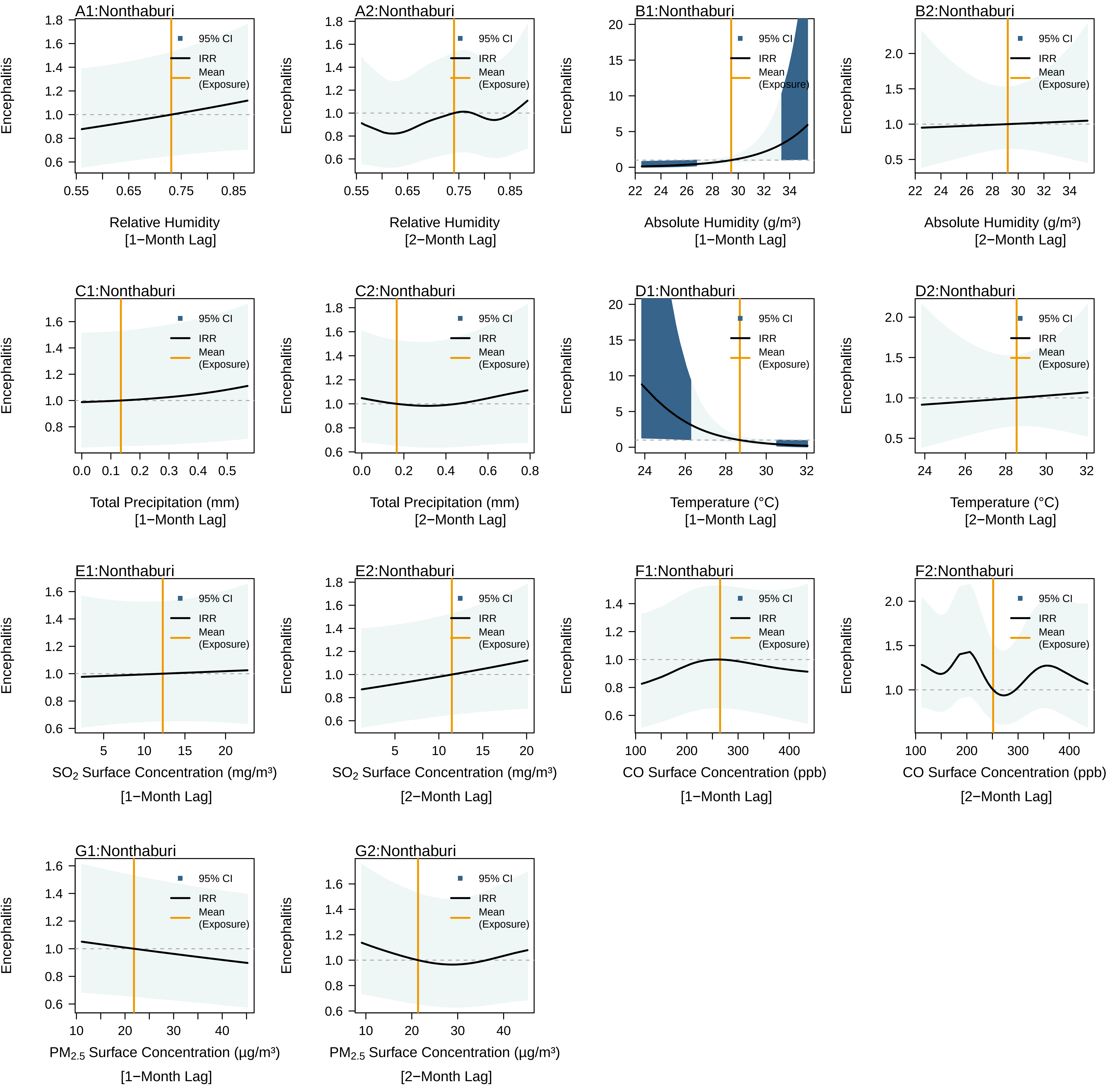
A1:Nonthaburi
Encephalitis
95% CI
IRR
Mean (Exposure)
Relative Humidity [1−Month Lag]
A2:Nonthaburi
Encephalitis
95% CI
IRR
Mean (Exposure)
Relative Humidity [2−Month Lag]
B1:Nonthaburi
Encephalitis
95% CI
IRR
Mean (Exposure)
Absolute Humidity (g/m³) [1−Month Lag]
B2:Nonthaburi
Encephalitis
95% CI
IRR
Mean (Exposure)
Absolute Humidity (g/m³) [2−Month Lag]
C1:Nonthaburi
Encephalitis
95% CI
IRR
Mean (Exposure)
Total Precipitation (mm) [1−Month Lag]
C2:Nonthaburi
Encephalitis
95% CI
IRR
Mean (Exposure)
Total Precipitation (mm) [2−Month Lag]
D1:Nonthaburi
Encephalitis
95% CI
IRR
Mean (Exposure)
Temperature (°C) [1−Month Lag]
D2:Nonthaburi
Encephalitis
95% CI
IRR
Mean (Exposure)
Temperature (°C) [2−Month Lag]
E1:Nonthaburi
Encephalitis
95% CI
IRR
Mean (Exposure)
SO_2 Surface Concentration (mg/m³) [1−Month Lag]
E2:Nonthaburi
Encephalitis
95% CI
IRR
Mean (Exposure)
SO_2 Surface Concentration (mg/m³) [2−Month Lag]
F1:Nonthaburi
Encephalitis
95% CI
IRR
Mean (Exposure)
CO Surface Concentration (ppb) [1−Month Lag]
F2:Nonthaburi
Encephalitis
95% CI
IRR
Mean (Exposure)
CO Surface Concentration (ppb) [2−Month Lag]
G1:Nonthaburi
Encephalitis
95% CI
IRR
Mean (Exposure)
$PM_{2.5}$ Surface Concentration (µg/m³) [1−Month Lag]
G2:Nonthaburi
Encephalitis
95% CI
IRR
Mean (Exposure)
$PM_{2.5}$ Surface Concentration (µg/m³) [2−Month Lag]

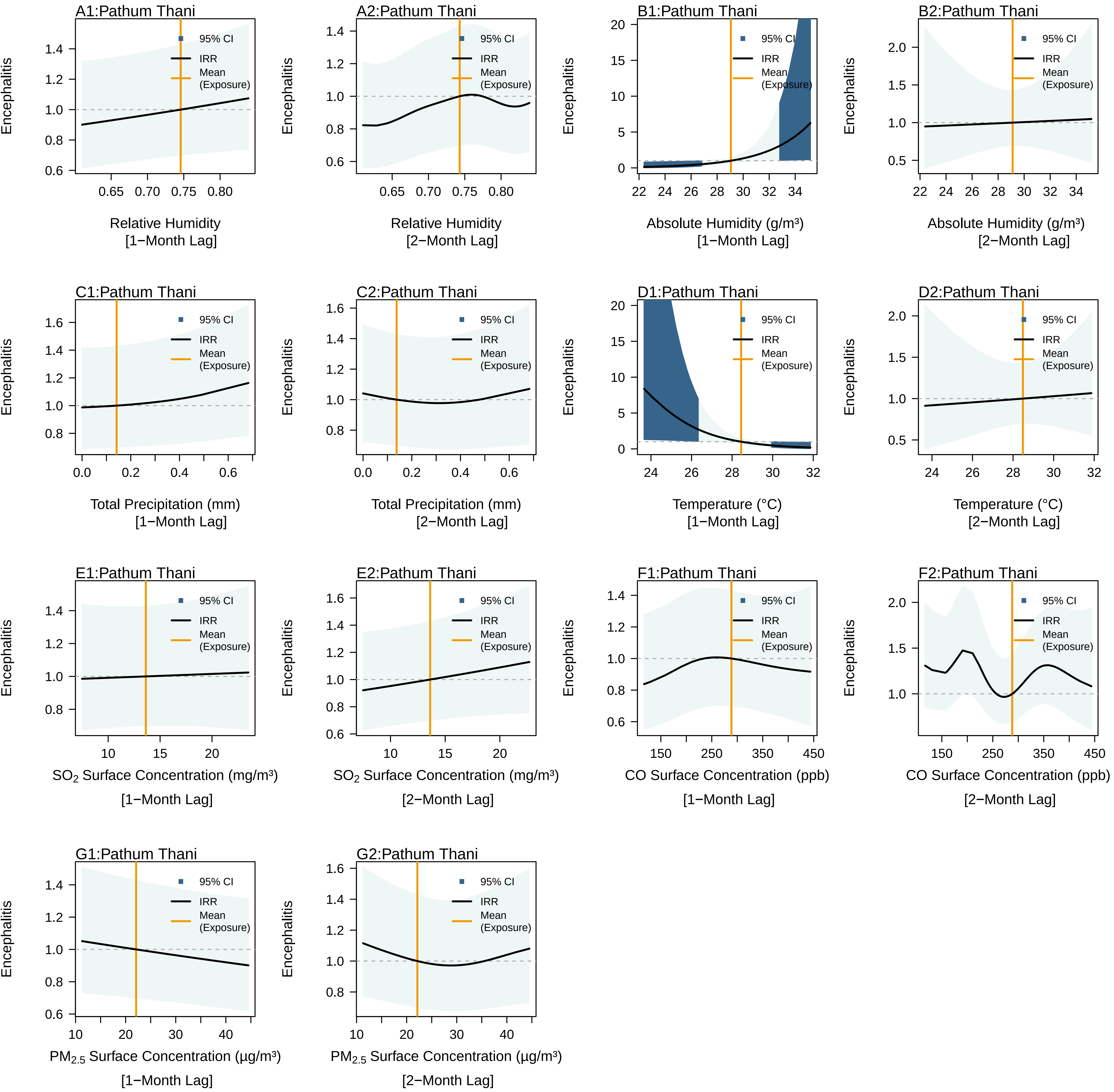
A1:Pathum Thani
Encephalitis
95% CI
IRR
Mean (Exposure)
Relative Humidity [1−Month Lag]
A2:Pathum Thani
Encephalitis
95% CI
IRR
Mean (Exposure)
Relative Humidity [2−Month Lag]
B1:Pathum Thani
Encephalitis
95% CI
IRR
Mean (Exposure)
Absolute Humidity (g/m³) [1−Month Lag]
B2:Pathum Thani
Encephalitis
95% CI
IRR
Mean (Exposure)
Absolute Humidity (g/m³) [2−Month Lag]
C1:Pathum Thani
Encephalitis
95% CI
IRR
Mean (Exposure)
Total Precipitation (mm) [1−Month Lag]
C2:Pathum Thani
Encephalitis
95% CI
IRR
Mean (Exposure)
Total Precipitation (mm) [2−Month Lag]
D1:Pathum Thani
Encephalitis
95% CI
IRR
Mean (Exposure)
Temperature (°C) [1−Month Lag]
D2:Pathum Thani
Encephalitis
95% CI
IRR
Mean (Exposure)
Temperature (°C) [2−Month Lag]
E1:Pathum Thani
Encephalitis
95% CI
IRR
Mean (Exposure)
SO2 Surface Concentration (mg/m³) [1−Month Lag]
E2:Pathum Thani
Encephalitis
95% CI
IRR
Mean (Exposure)
SO2 Surface Concentration (mg/m³) [2−Month Lag]
F1:Pathum Thani
Encephalitis
95% CI
IRR
Mean (Exposure)
CO Surface Concentration (ppb) [1−Month Lag]
F2:Pathum Thani
Encephalitis
95% CI
IRR
Mean (Exposure)
CO Surface Concentration (ppb) [2−Month Lag]
G1:Pathum Thani
Encephalitis
95% CI
IRR
Mean (Exposure)
PM2.5 Surface Concentration (µg/m³) [1−Month Lag]
G2:Pathum Thani
Encephalitis
95% CI
IRR
Mean (Exposure)
PM2.5 Surface Concentration (µg/m³) [2−Month Lag]

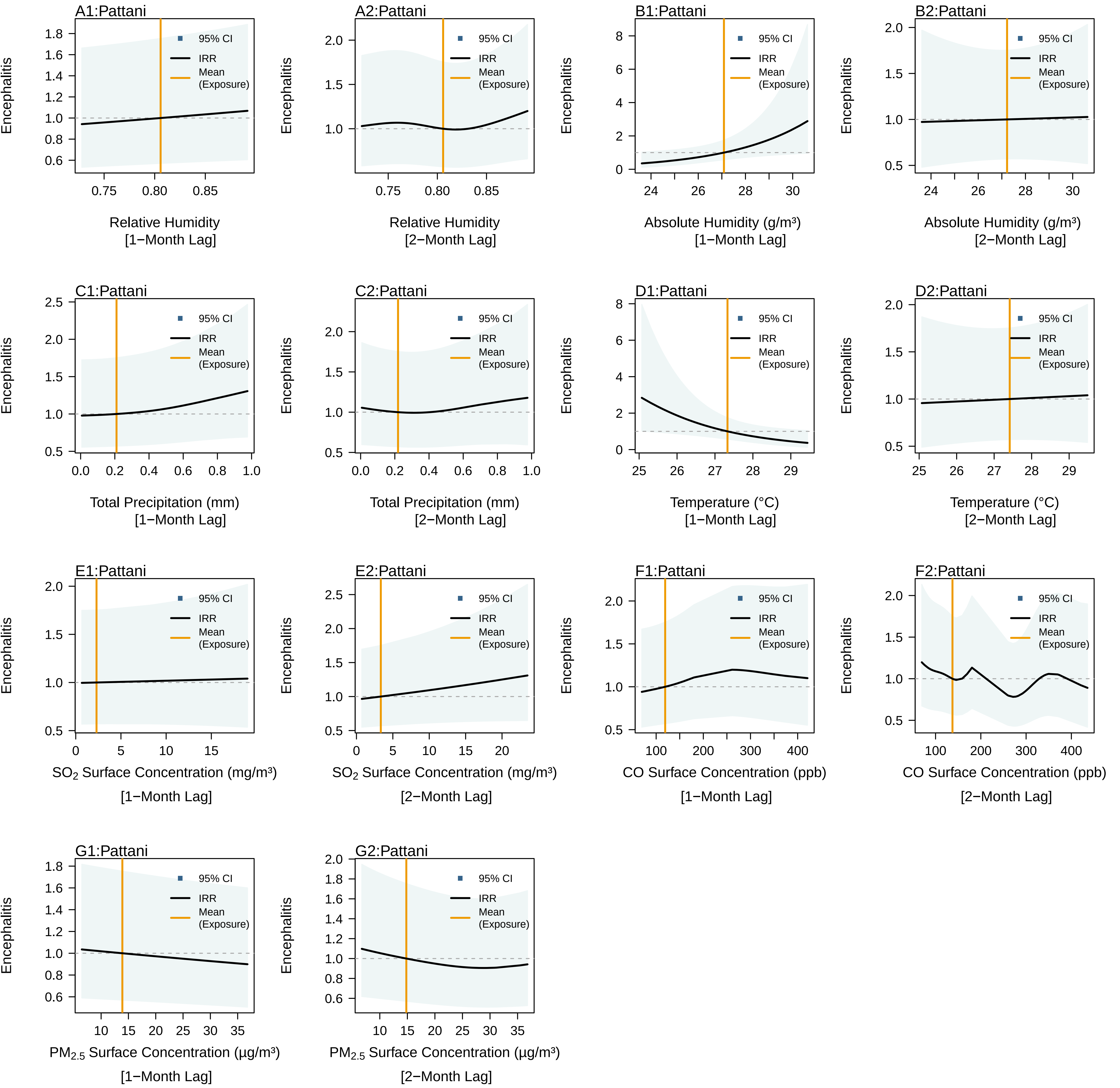
A1:Pattani
A2:Pattani
B1:Pattani
B2:Pattani
C1:Pattani
C2:Pattani
D1:Pattani
D2:Pattani
E1:Pattani
E2:Pattani
F1:Pattani
F2:Pattani
G1:Pattani
G2:Pattani
Encephalitis
95% CI
IRR
Mean
(Exposure)
Relative Humidity
[1−Month Lag]
Relative Humidity
[2−Month Lag]
Absolute Humidity (g/m³)
[1−Month Lag]
Absolute Humidity (g/m³)
[2−Month Lag]
Total Precipitation (mm)
[1−Month Lag]
Total Precipitation (mm)
[2−Month Lag]
Temperature (°C)
[1−Month Lag]
Temperature (°C)
[2−Month Lag]
SO2 Surface Concentration (mg/m³)
[1−Month Lag]
SO2 Surface Concentration (mg/m³)
[2−Month Lag]
CO Surface Concentration (ppb)
[1−Month Lag]
CO Surface Concentration (ppb)
[2−Month Lag]
PM2.5 Surface Concentration (µg/m³)
[1−Month Lag]
PM2.5 Surface Concentration (µg/m³)
[2−Month Lag]

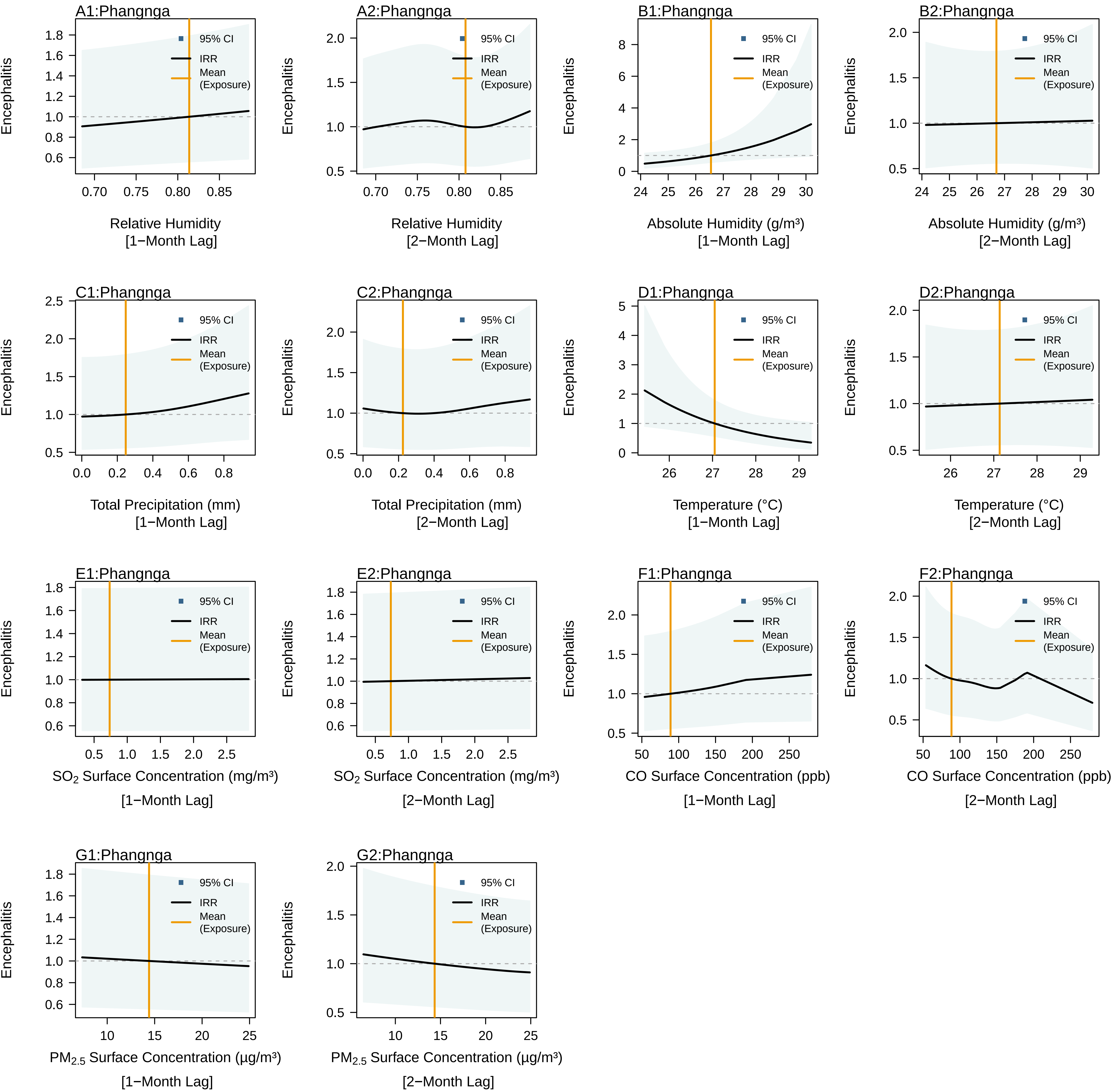
A1:Phangnga
A2:Phangnga
B1:Phangnga
B2:Phangnga
C1:Phangnga
C2:Phangnga
D1:Phangnga
D2:Phangnga
E1:Phangnga
E2:Phangnga
F1:Phangnga
F2:Phangnga
G1:Phangnga
G2:Phangnga
Encephalitis
95% CI
IRR
Mean (Exposure)
Relative Humidity [1−Month Lag]
Relative Humidity [2−Month Lag]
Absolute Humidity (g/m³) [1−Month Lag]
Absolute Humidity (g/m³) [2−Month Lag]
Total Precipitation (mm) [1−Month Lag]
Total Precipitation (mm) [2−Month Lag]
Temperature (°C) [1−Month Lag]
Temperature (°C) [2−Month Lag]
SO_2 Surface Concentration (mg/m³) [1−Month Lag]
SO_2 Surface Concentration (mg/m³) [2−Month Lag]
CO Surface Concentration (ppb) [1−Month Lag]
CO Surface Concentration (ppb) [2−Month Lag]
$PM_{2.5}$ Surface Concentration (µg/m³) [1−Month Lag]
$PM_{2.5}$ Surface Concentration (µg/m³) [2−Month Lag]

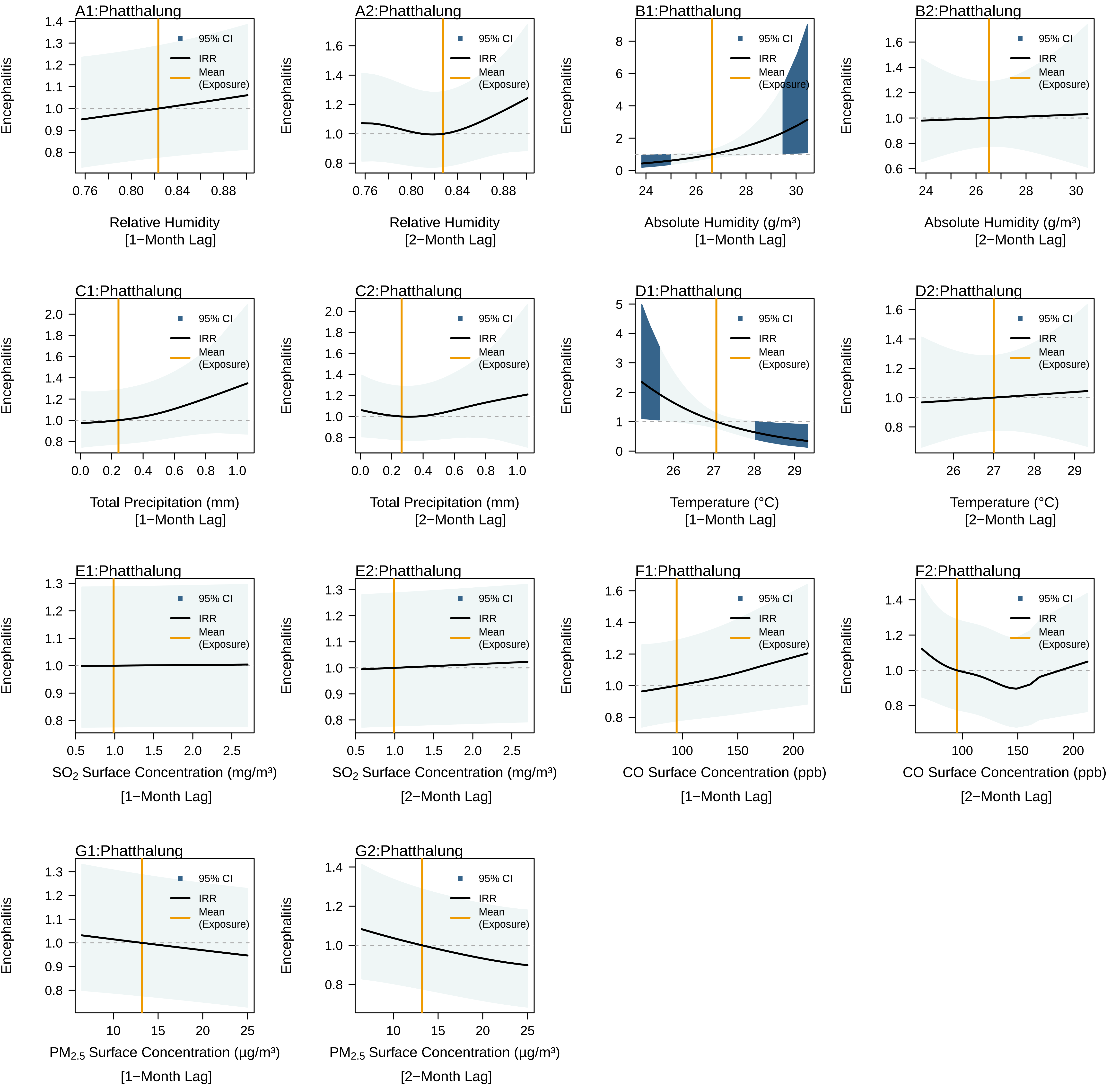
A1:Phatthalung
A2:Phatthalung
B1:Phatthalung
B2:Phatthalung
C1:Phatthalung
C2:Phatthalung
D1:Phatthalung
D2:Phatthalung
E1:Phatthalung
E2:Phatthalung
F1:Phatthalung
F2:Phatthalung
G1:Phatthalung
G2:Phatthalung
Encephalitis
95% CI
IRR
Mean (Exposure)
Relative Humidity [1−Month Lag]
Relative Humidity [2−Month Lag]
Absolute Humidity (g/m³) [1−Month Lag]
Absolute Humidity (g/m³) [2−Month Lag]
Total Precipitation (mm) [1−Month Lag]
Total Precipitation (mm) [2−Month Lag]
Temperature (°C) [1−Month Lag]
Temperature (°C) [2−Month Lag]
SO_2 Surface Concentration (mg/m³) [1−Month Lag]
SO_2 Surface Concentration (mg/m³) [2−Month Lag]
CO Surface Concentration (ppb) [1−Month Lag]
CO Surface Concentration (ppb) [2−Month Lag]
$PM_{2.5}$ Surface Concentration (µg/m³) [1−Month Lag]
$PM_{2.5}$ Surface Concentration (µg/m³) [2−Month Lag]

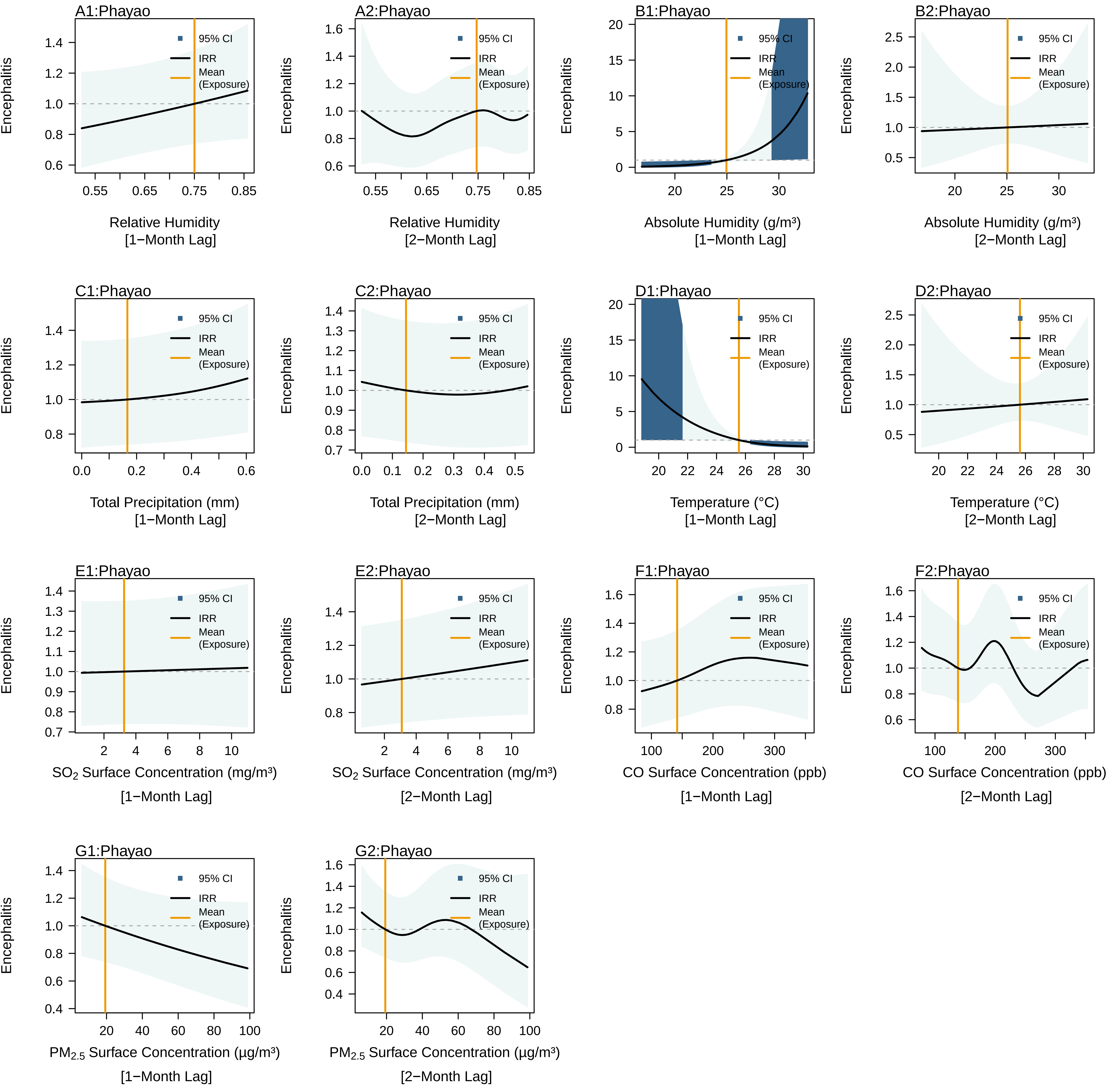
A1:Phayao
Encephalitis
95% CI
IRR
Mean (Exposure)
0.6 0.8 1.0 1.2 1.4
0.55 0.65 0.75 0.85
Relative Humidity [1−Month Lag]
A2:Phayao
Encephalitis
95% CI
IRR
Mean (Exposure)
0.6 0.8 1.0 1.2 1.4 1.6
0.55 0.65 0.75 0.85
Relative Humidity [2−Month Lag]
B1:Phayao
Encephalitis
95% CI
IRR
Mean (Exposure)
0 5 10 15 20
20 25 30
Absolute Humidity (g/m³) [1−Month Lag]
B2:Phayao
Encephalitis
95% CI
IRR
Mean (Exposure)
0.5 1.0 1.5 2.0 2.5
20 25 30
Absolute Humidity (g/m³) [2−Month Lag]
C1:Phayao
Encephalitis
95% CI
IRR
Mean (Exposure)
0.8 1.0 1.2 1.4
0.0 0.2 0.4 0.6
Total Precipitation (mm) [1−Month Lag]
C2:Phayao
Encephalitis
95% CI
IRR
Mean (Exposure)
0.7 0.8 0.9 1.0 1.1 1.2 1.3 1.4
0.0 0.1 0.2 0.3 0.4 0.5
Total Precipitation (mm) [2−Month Lag]
D1:Phayao
Encephalitis
95% CI
IRR
Mean (Exposure)
0 5 10 15 20
20 22 24 26 28 30
Temperature (°C) [1−Month Lag]
D2:Phayao
Encephalitis
95% CI
IRR
Mean (Exposure)
0.5 1.0 1.5 2.0 2.5
20 22 24 26 28 30
Temperature (°C) [2−Month Lag]
E1:Phayao
Encephalitis
95% CI
IRR
Mean (Exposure)
0.7 0.8 0.9 1.0 1.1 1.2 1.3 1.4
2 4 6 8 10
SO2 Surface Concentration (mg/m³) [1−Month Lag]
E2:Phayao
Encephalitis
95% CI
IRR
Mean (Exposure)
0.8 1.0 1.2 1.4
2 4 6 8 10
SO2 Surface Concentration (mg/m³) [2−Month Lag]
F1:Phayao
Encephalitis
95% CI
IRR
Mean (Exposure)
0.8 1.0 1.2 1.4 1.6
100 200 300
CO Surface Concentration (ppb) [1−Month Lag]
F2:Phayao
Encephalitis
95% CI
IRR
Mean (Exposure)
0.6 0.8 1.0 1.2 1.4 1.6
100 200 300
CO Surface Concentration (ppb) [2−Month Lag]
G1:Phayao
Encephalitis
95% CI
IRR
Mean (Exposure)
0.4 0.6 0.8 1.0 1.2 1.4
20 40 60 80 100
PM2.5 Surface Concentration (µg/m³) [1−Month Lag]
G2:Phayao
Encephalitis
95% CI
IRR
Mean (Exposure)
0.4 0.6 0.8 1.0 1.2 1.4 1.6
20 40 60 80 100
PM2.5 Surface Concentration (µg/m³) [2−Month Lag]

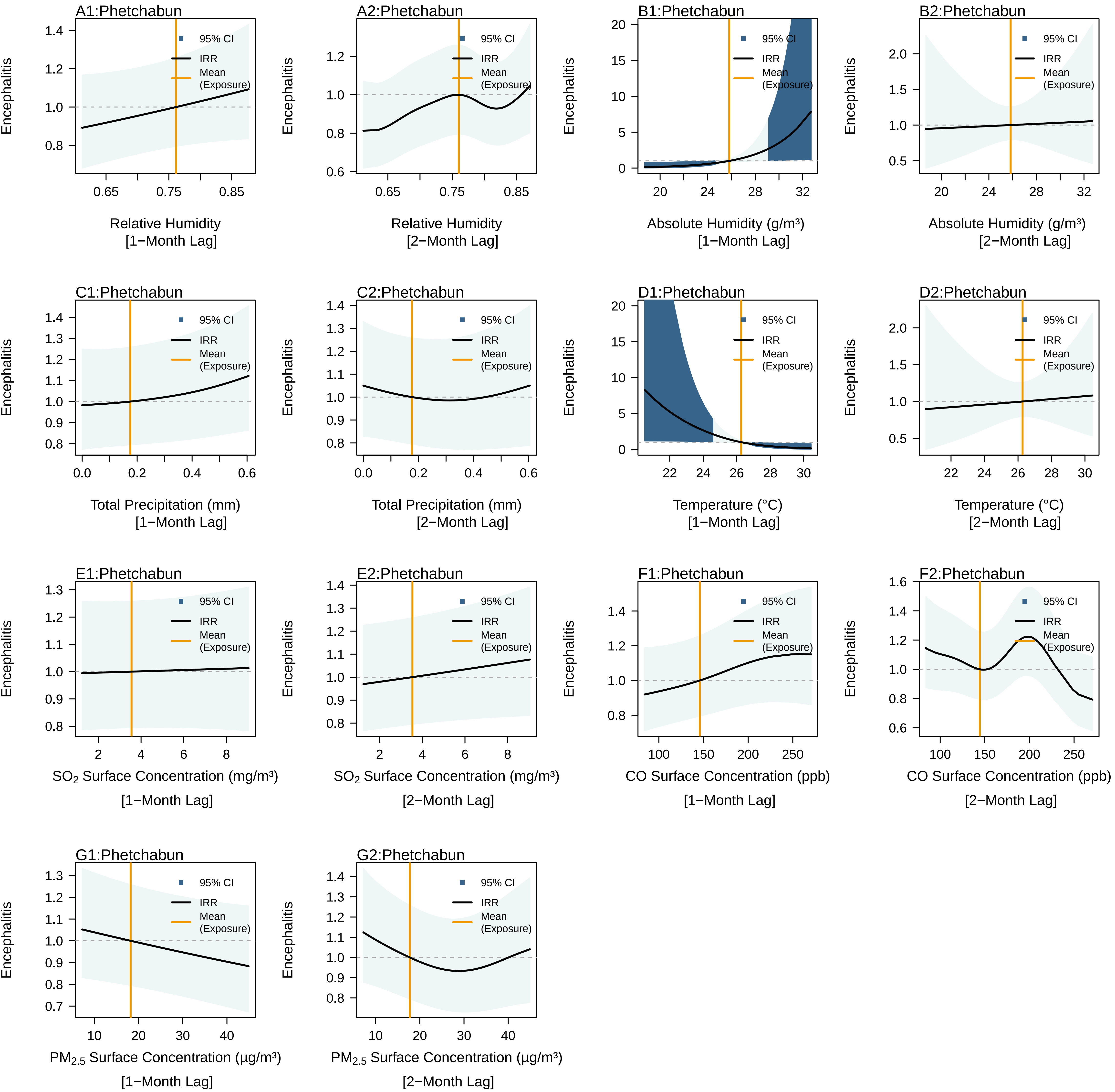

A1:Phetchabun
Encephalitis
95% CI
IRR
Mean (Exposure)
Relative Humidity [1−Month Lag]
A2:Phetchabun
Encephalitis
95% CI
IRR
Mean (Exposure)
Relative Humidity [2−Month Lag]
B1:Phetchabun
Encephalitis
95% CI
IRR
Mean (Exposure)
Absolute Humidity (g/m³) [1−Month Lag]
B2:Phetchabun
Encephalitis
95% CI
IRR
Mean (Exposure)
Absolute Humidity (g/m³) [2−Month Lag]
C1:Phetchabun
Encephalitis
95% CI
IRR
Mean (Exposure)
Total Precipitation (mm) [1−Month Lag]
C2:Phetchabun
Encephalitis
95% CI
IRR
Mean (Exposure)
Total Precipitation (mm) [2−Month Lag]
D1:Phetchabun
Encephalitis
95% CI
IRR
Mean (Exposure)
Temperature (°C) [1−Month Lag]
D2:Phetchabun
Encephalitis
95% CI
IRR
Mean (Exposure)
Temperature (°C) [2−Month Lag]
E1:Phetchabun
Encephalitis
95% CI
IRR
Mean (Exposure)
SO_2 Surface Concentration (mg/m³) [1−Month Lag]
E2:Phetchabun
Encephalitis
95% CI
IRR
Mean (Exposure)
SO_2 Surface Concentration (mg/m³) [2−Month Lag]
F1:Phetchabun
Encephalitis
95% CI
IRR
Mean (Exposure)
CO Surface Concentration (ppb) [1−Month Lag]
F2:Phetchabun
Encephalitis
95% CI
IRR
Mean (Exposure)
CO Surface Concentration (ppb) [2−Month Lag]
G1:Phetchabun
Encephalitis
95% CI
IRR
Mean (Exposure)
$PM_{2.5}$ Surface Concentration (µg/m³) [1−Month Lag]
G2:Phetchabun
Encephalitis
95% CI
IRR
Mean (Exposure)
$PM_{2.5}$ Surface Concentration (µg/m³) [2−Month Lag]

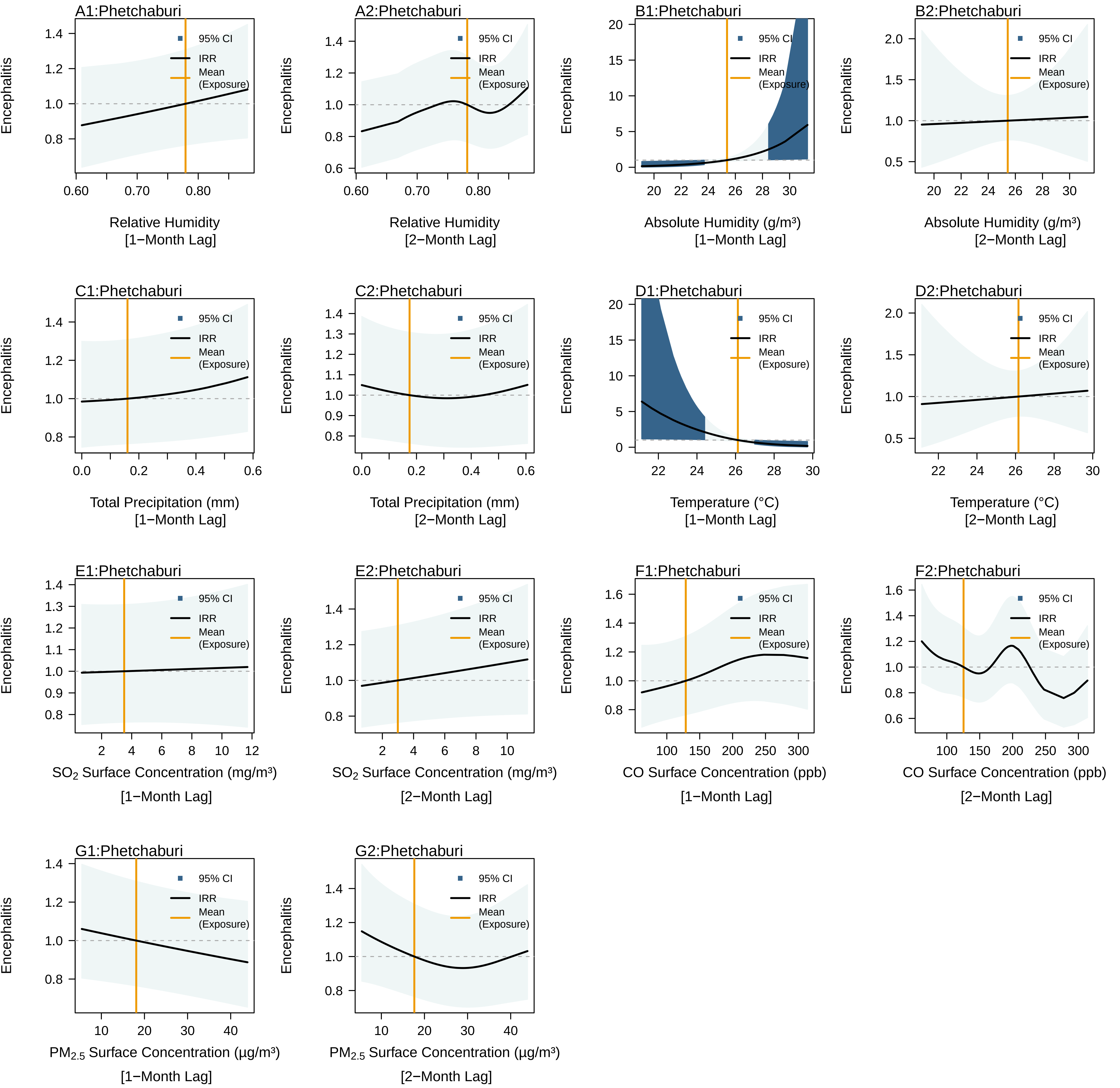
A1:Phetchaburi
A2:Phetchaburi
B1:Phetchaburi
B2:Phetchaburi
Encephalitis
95% CI
IRR
Mean
(Exposure)
Relative Humidity
[1−Month Lag]
Relative Humidity
[2−Month Lag]
Absolute Humidity (g/m³)
[1−Month Lag]
Absolute Humidity (g/m³)
[2−Month Lag]
C1:Phetchaburi
C2:Phetchaburi
D1:Phetchaburi
D2:Phetchaburi
Total Precipitation (mm)
[1−Month Lag]
Total Precipitation (mm)
[2−Month Lag]
Temperature (°C)
[1−Month Lag]
Temperature (°C)
[2−Month Lag]
E1:Phetchaburi
E2:Phetchaburi
F1:Phetchaburi
F2:Phetchaburi
SO_2 Surface Concentration (mg/m³)
[1−Month Lag]
SO_2 Surface Concentration (mg/m³)
[2−Month Lag]
CO Surface Concentration (ppb)
[1−Month Lag]
CO Surface Concentration (ppb)
[2−Month Lag]
G1:Phetchaburi
G2:Phetchaburi
$PM_{2.5}$ Surface Concentration (µg/m³)
[1−Month Lag]
$PM_{2.5}$ Surface Concentration (µg/m³)
[2−Month Lag]

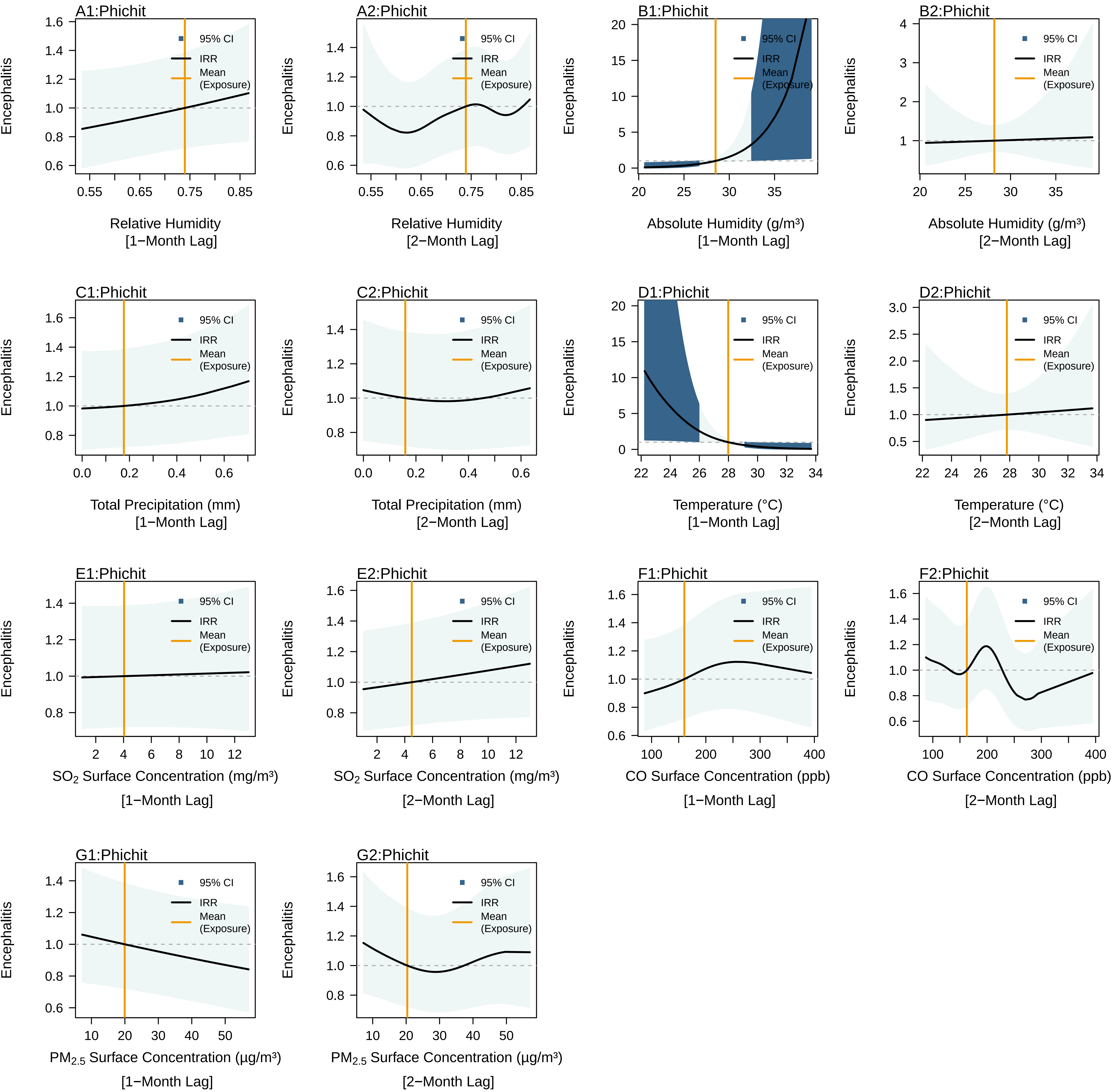
A1:Phichit
A2:Phichit
B1:Phichit
B2:Phichit
95% CI
IRR
Mean
(Exposure)
Encephalitis
Relative Humidity
[1−Month Lag]
Relative Humidity
[2−Month Lag]
Absolute Humidity (g/m³)
[1−Month Lag]
Absolute Humidity (g/m³)
[2−Month Lag]
C1:Phichit
C2:Phichit
D1:Phichit
D2:Phichit
Total Precipitation (mm)
[1−Month Lag]
Total Precipitation (mm)
[2−Month Lag]
Temperature (°C)
[1−Month Lag]
Temperature (°C)
[2−Month Lag]
E1:Phichit
E2:Phichit
F1:Phichit
F2:Phichit
SO_2 Surface Concentration (mg/m³)
[1−Month Lag]
SO_2 Surface Concentration (mg/m³)
[2−Month Lag]
CO Surface Concentration (ppb)
[1−Month Lag]
CO Surface Concentration (ppb)
[2−Month Lag]
G1:Phichit
G2:Phichit
$PM_{2.5}$ Surface Concentration (µg/m³)
[1−Month Lag]
$PM_{2.5}$ Surface Concentration (µg/m³)
[2−Month Lag]

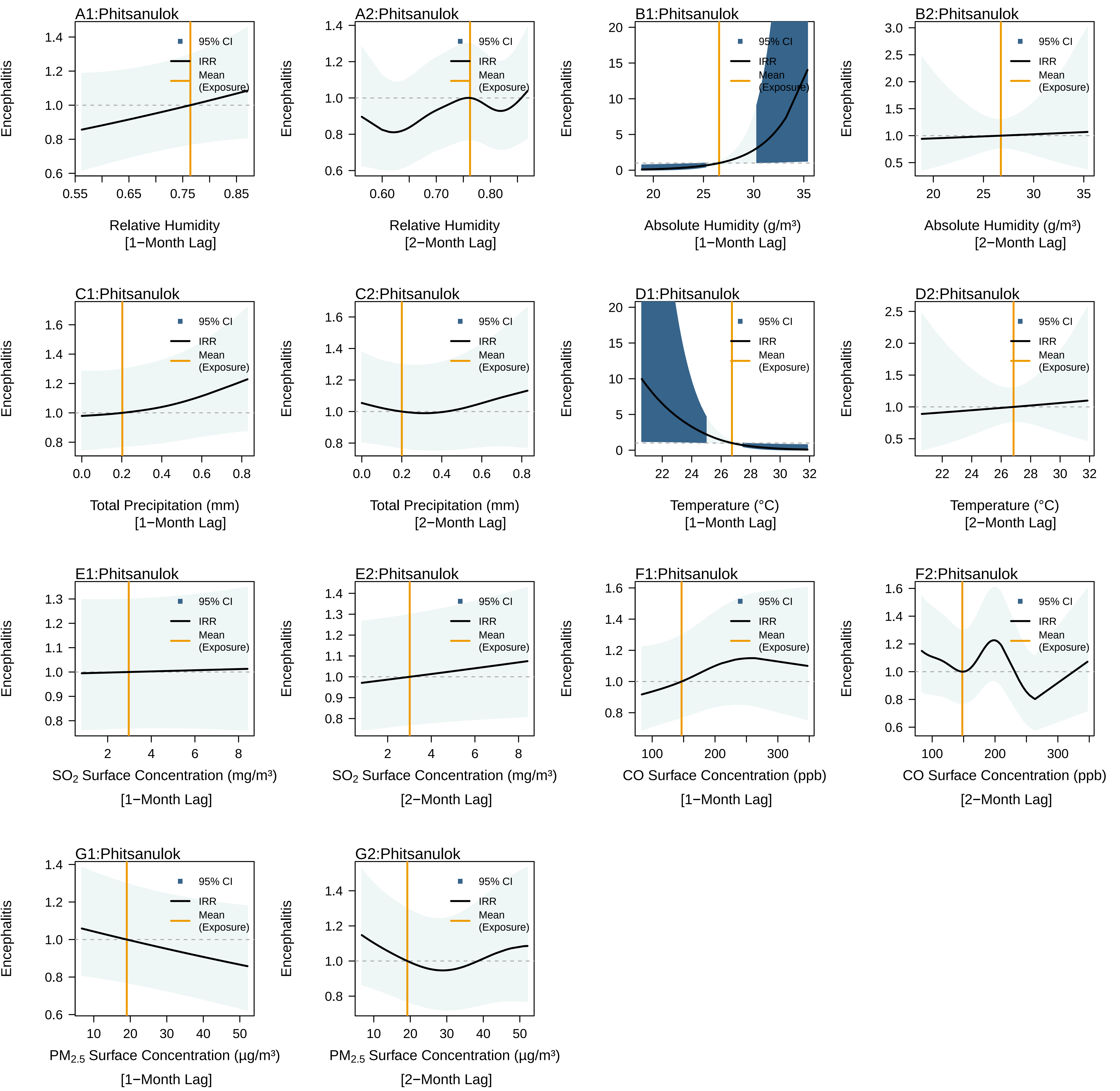
A1:Phitsanulok
A2:Phitsanulok
B1:Phitsanulok
B2:Phitsanulok
C1:Phitsanulok
C2:Phitsanulok
D1:Phitsanulok
D2:Phitsanulok
E1:Phitsanulok
E2:Phitsanulok
F1:Phitsanulok
F2:Phitsanulok
G1:Phitsanulok
G2:Phitsanulok
Encephalitis
95% CI
IRR
Mean (Exposure)
Relative Humidity [1−Month Lag]
Relative Humidity [2−Month Lag]
Absolute Humidity (g/m³) [1−Month Lag]
Absolute Humidity (g/m³) [2−Month Lag]
Total Precipitation (mm) [1−Month Lag]
Total Precipitation (mm) [2−Month Lag]
Temperature (°C) [1−Month Lag]
Temperature (°C) [2−Month Lag]
SO2 Surface Concentration (mg/m³) [1−Month Lag]
SO2 Surface Concentration (mg/m³) [2−Month Lag]
CO Surface Concentration (ppb) [1−Month Lag]
CO Surface Concentration (ppb) [2−Month Lag]
PM2.5 Surface Concentration (µg/m³) [1−Month Lag]
PM2.5 Surface Concentration (µg/m³) [2−Month Lag]

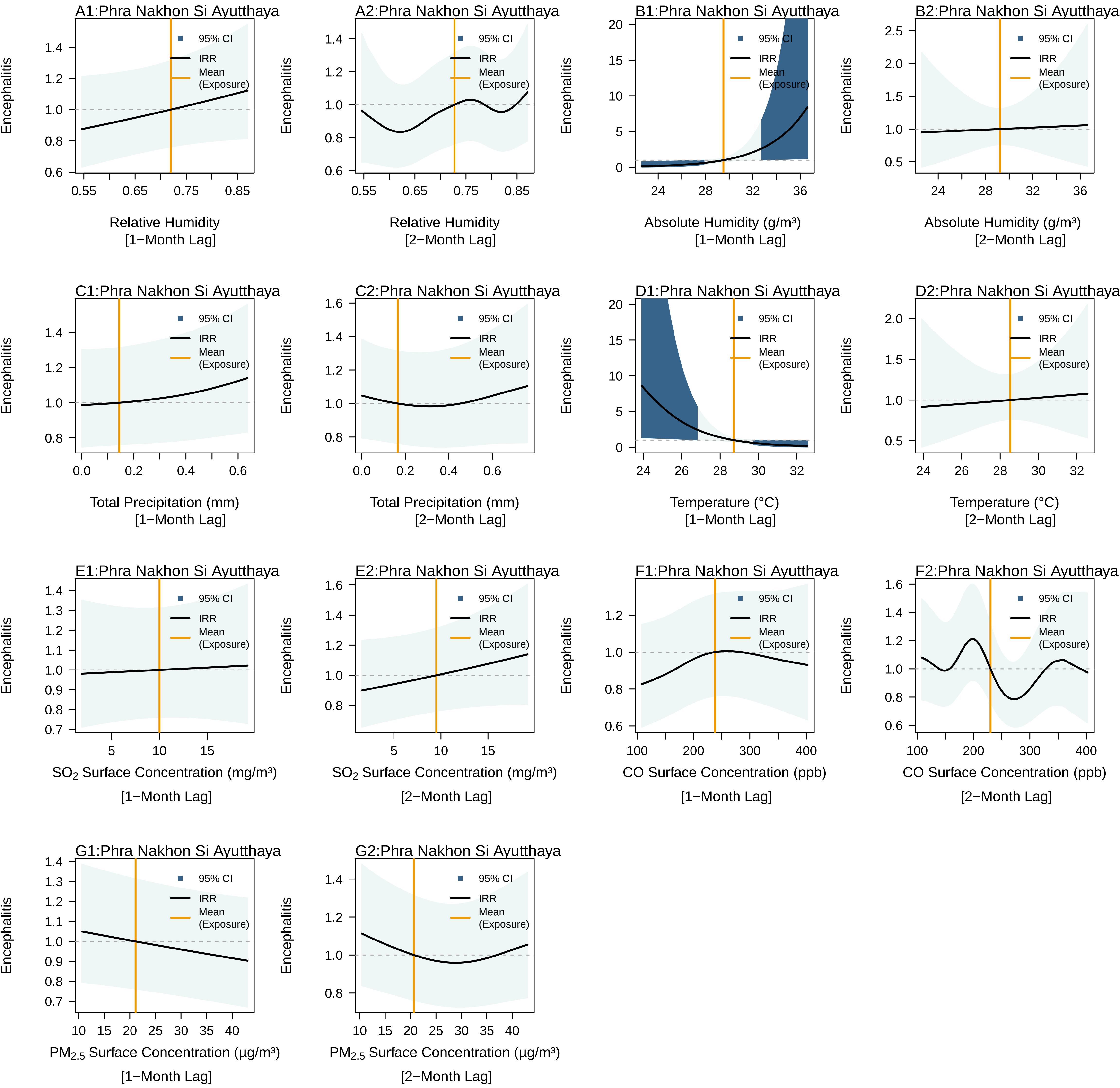
A1:Phra Nakhon Si Ayutthaya
Encephalitis
95% CI
IRR
Mean (Exposure)
Relative Humidity [1−Month Lag]
A2:Phra Nakhon Si Ayutthaya
Encephalitis
95% CI
IRR
Mean (Exposure)
Relative Humidity [2−Month Lag]
B1:Phra Nakhon Si Ayutthaya
Encephalitis
95% CI
IRR
Mean (Exposure)
Absolute Humidity (g/m³) [1−Month Lag]
B2:Phra Nakhon Si Ayutthaya
Encephalitis
95% CI
IRR
Mean (Exposure)
Absolute Humidity (g/m³) [2−Month Lag]
C1:Phra Nakhon Si Ayutthaya
Encephalitis
95% CI
IRR
Mean (Exposure)
Total Precipitation (mm) [1−Month Lag]
C2:Phra Nakhon Si Ayutthaya
Encephalitis
95% CI
IRR
Mean (Exposure)
Total Precipitation (mm) [2−Month Lag]
D1:Phra Nakhon Si Ayutthaya
Encephalitis
95% CI
IRR
Mean (Exposure)
Temperature (°C) [1−Month Lag]
D2:Phra Nakhon Si Ayutthaya
Encephalitis
95% CI
IRR
Mean (Exposure)
Temperature (°C) [2−Month Lag]
E1:Phra Nakhon Si Ayutthaya
Encephalitis
95% CI
IRR
Mean (Exposure)
SO2 Surface Concentration (mg/m³) [1−Month Lag]
E2:Phra Nakhon Si Ayutthaya
Encephalitis
95% CI
IRR
Mean (Exposure)
SO2 Surface Concentration (mg/m³) [2−Month Lag]
F1:Phra Nakhon Si Ayutthaya
Encephalitis
95% CI
IRR
Mean (Exposure)
CO Surface Concentration (ppb) [1−Month Lag]
F2:Phra Nakhon Si Ayutthaya
Encephalitis
95% CI
IRR
Mean (Exposure)
CO Surface Concentration (ppb) [2−Month Lag]
G1:Phra Nakhon Si Ayutthaya
Encephalitis
95% CI
IRR
Mean (Exposure)
PM2.5 Surface Concentration (µg/m³) [1−Month Lag]
G2:Phra Nakhon Si Ayutthaya
Encephalitis
95% CI
IRR
Mean (Exposure)
PM2.5 Surface Concentration (µg/m³) [2−Month Lag]

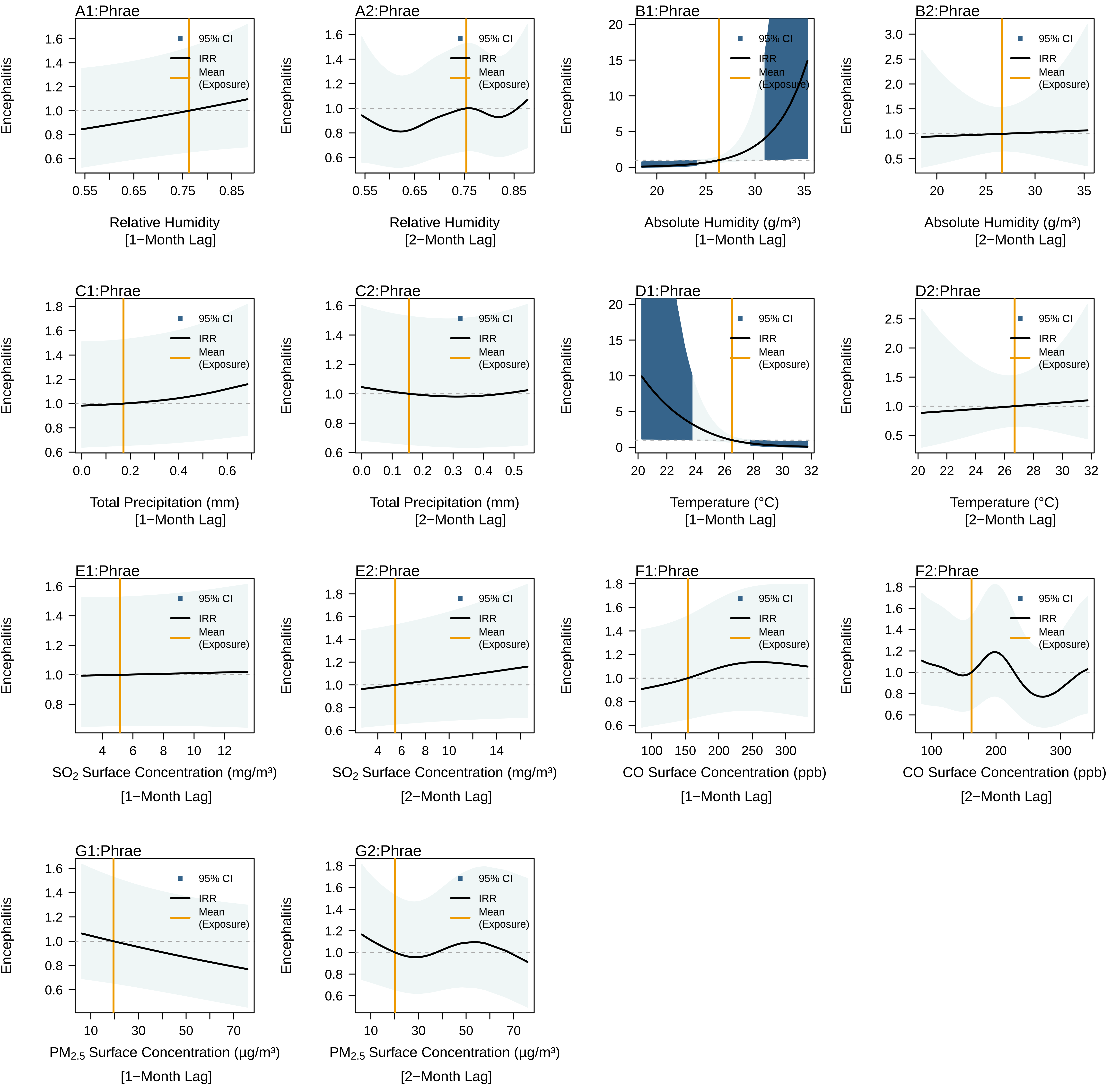

A1:Phrae
Encephalitis
95% CI
IRR
Mean (Exposure)
Relative Humidity [1−Month Lag]
A2:Phrae
Encephalitis
Relative Humidity [2−Month Lag]
B1:Phrae
Encephalitis
Absolute Humidity (g/m³) [1−Month Lag]
B2:Phrae
Encephalitis
Absolute Humidity (g/m³) [2−Month Lag]
C1:Phrae
Encephalitis
Total Precipitation (mm) [1−Month Lag]
C2:Phrae
Encephalitis
Total Precipitation (mm) [2−Month Lag]
D1:Phrae
Encephalitis
Temperature (°C) [1−Month Lag]
D2:Phrae
Encephalitis
Temperature (°C) [2−Month Lag]
E1:Phrae
Encephalitis
SO_2 Surface Concentration (mg/m³) [1−Month Lag]
E2:Phrae
Encephalitis
SO_2 Surface Concentration (mg/m³) [2−Month Lag]
F1:Phrae
Encephalitis
CO Surface Concentration (ppb) [1−Month Lag]
F2:Phrae
Encephalitis
CO Surface Concentration (ppb) [2−Month Lag]
G1:Phrae
Encephalitis
$PM_{2.5}$ Surface Concentration (µg/m³) [1−Month Lag]
G2:Phrae
Encephalitis
$PM_{2.5}$ Surface Concentration (µg/m³) [2−Month Lag]

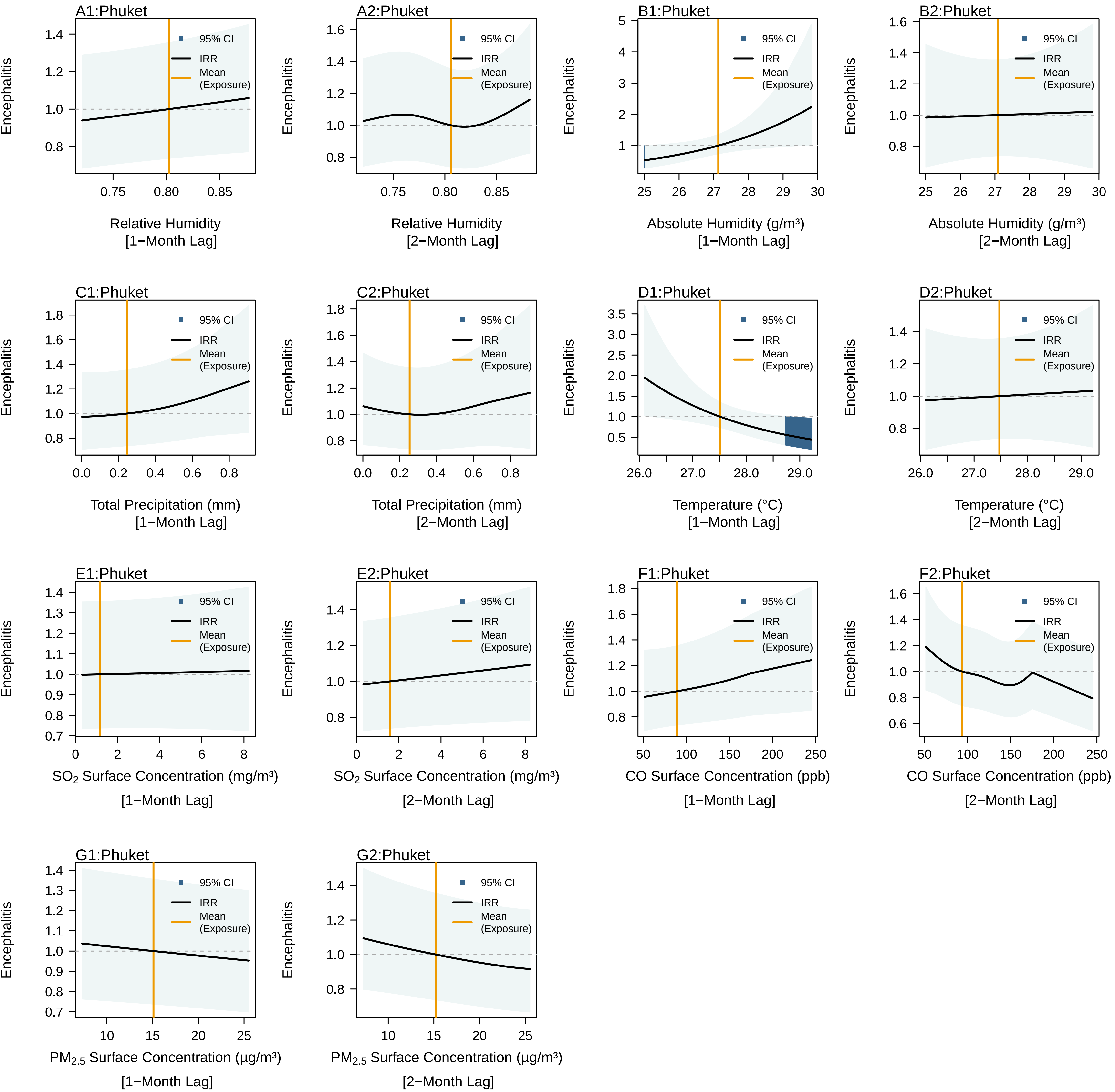
A1:Phuket
95% CI
IRR
Mean (Exposure)
Encephalitis
Relative Humidity [1−Month Lag]
A2:Phuket
Relative Humidity [2−Month Lag]
B1:Phuket
Absolute Humidity (g/m³) [1−Month Lag]
B2:Phuket
Absolute Humidity (g/m³) [2−Month Lag]
C1:Phuket
Total Precipitation (mm) [1−Month Lag]
C2:Phuket
Total Precipitation (mm) [2−Month Lag]
D1:Phuket
Temperature (°C) [1−Month Lag]
D2:Phuket
Temperature (°C) [2−Month Lag]
E1:Phuket
SO2 Surface Concentration (mg/m³) [1−Month Lag]
E2:Phuket
SO2 Surface Concentration (mg/m³) [2−Month Lag]
F1:Phuket
CO Surface Concentration (ppb) [1−Month Lag]
F2:Phuket
CO Surface Concentration (ppb) [2−Month Lag]
G1:Phuket
PM2.5 Surface Concentration (µg/m³) [1−Month Lag]
G2:Phuket
PM2.5 Surface Concentration (µg/m³) [2−Month Lag]

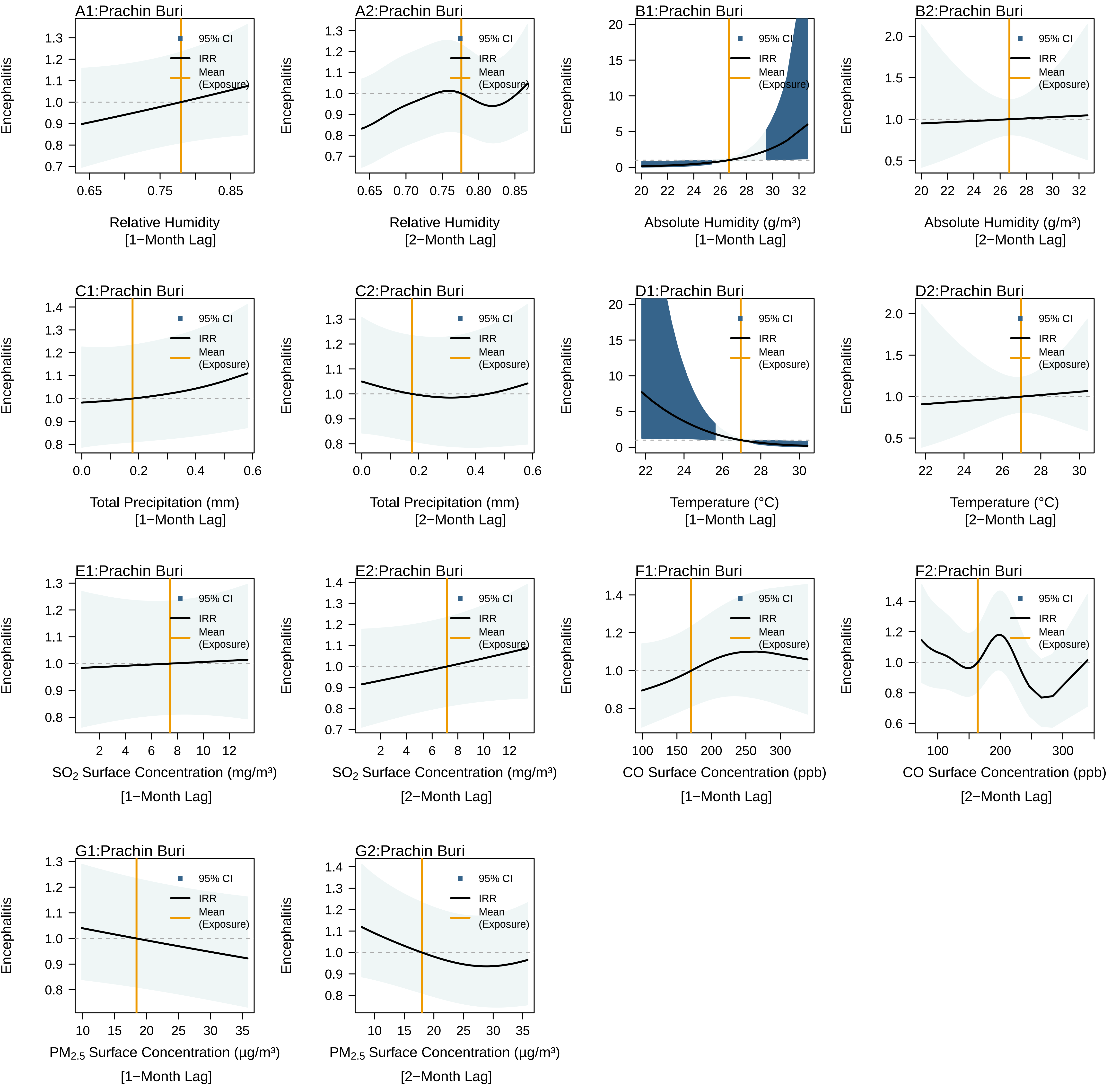
A1:Prachin Buri
Encephalitis
95% CI
IRR
Mean
(Exposure)
1.3
1.2
1.1
1.0
0.9
0.8
0.7
0.65
0.75
0.85
Relative Humidity
[1−Month Lag]
A2:Prachin Buri
Encephalitis
95% CI
IRR
Mean
(Exposure)
1.3
1.2
1.1
1.0
0.9
0.8
0.7
0.65
0.70
0.75
0.80
0.85
Relative Humidity
[2−Month Lag]
B1:Prachin Buri
Encephalitis
95% CI
IRR
Mean
(Exposure)
20
15
10
5
0
20
22
24
26
28
30
32
Absolute Humidity (g/m³)
[1−Month Lag]
B2:Prachin Buri
Encephalitis
95% CI
IRR
Mean
(Exposure)
2.0
1.5
1.0
0.5
20
22
24
26
28
30
32
Absolute Humidity (g/m³)
[2−Month Lag]
C1:Prachin Buri
Encephalitis
95% CI
IRR
Mean
(Exposure)
1.4
1.3
1.2
1.1
1.0
0.9
0.8
0.0
0.2
0.4
0.6
Total Precipitation (mm)
[1−Month Lag]
C2:Prachin Buri
Encephalitis
95% CI
IRR
Mean
(Exposure)
1.3
1.2
1.1
1.0
0.9
0.8
0.0
0.2
0.4
0.6
Total Precipitation (mm)
[2−Month Lag]
D1:Prachin Buri
Encephalitis
95% CI
IRR
Mean
(Exposure)
20
15
10
5
0
22
24
26
28
30
Temperature (°C)
[1−Month Lag]
D2:Prachin Buri
Encephalitis
95% CI
IRR
Mean
(Exposure)
2.0
1.5
1.0
0.5
22
24
26
28
30
Temperature (°C)
[2−Month Lag]
E1:Prachin Buri
Encephalitis
95% CI
IRR
Mean
(Exposure)
1.3
1.2
1.1
1.0
0.9
0.8
2
4
6
8
10
12
SO2 Surface Concentration (mg/m³)
[1−Month Lag]
E2:Prachin Buri
Encephalitis
95% CI
IRR
Mean
(Exposure)
1.4
1.3
1.2
1.1
1.0
0.9
0.8
0.7
2
4
6
8
10
12
SO2 Surface Concentration (mg/m³)
[2−Month Lag]
F1:Prachin Buri
Encephalitis
95% CI
IRR
Mean
(Exposure)
1.4
1.2
1.0
0.8
100
150
200
250
300
CO Surface Concentration (ppb)
[1−Month Lag]
F2:Prachin Buri
Encephalitis
95% CI
IRR
Mean
(Exposure)
1.4
1.2
1.0
0.8
0.6
100
200
300
CO Surface Concentration (ppb)
[2−Month Lag]
G1:Prachin Buri
Encephalitis
95% CI
IRR
Mean
(Exposure)
1.3
1.2
1.1
1.0
0.9
0.8
10
15
20
25
30
35
PM2.5 Surface Concentration (µg/m³)
[1−Month Lag]
G2:Prachin Buri
Encephalitis
95% CI
IRR
Mean
(Exposure)
1.4
1.3
1.2
1.1
1.0
0.9
0.8
10
15
20
25
30
35
PM2.5 Surface Concentration (µg/m³)
[2−Month Lag]

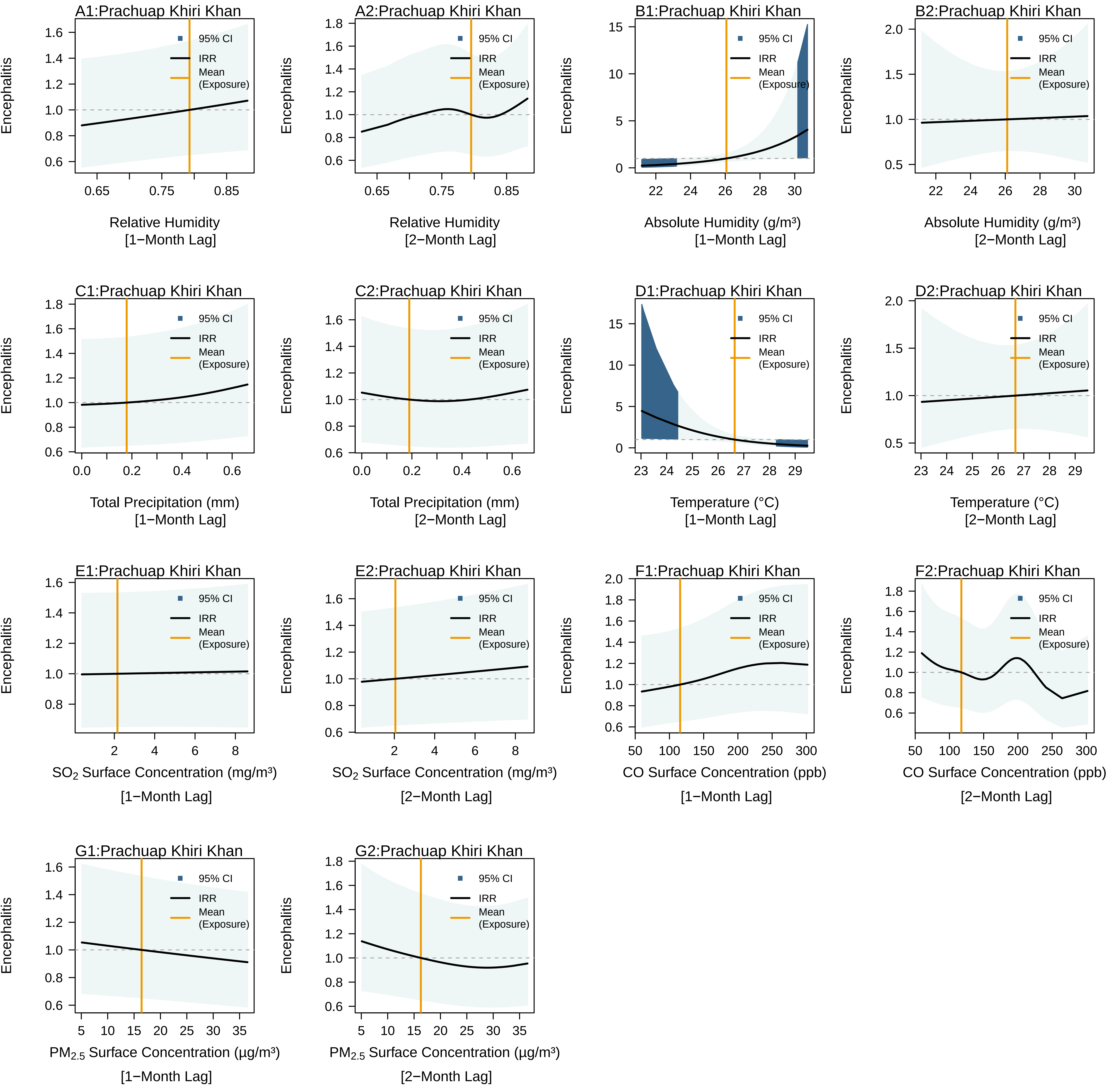

A1:Prachuap Khiri Khan
Encephalitis
95% CI
IRR
Mean (Exposure)
Relative Humidity [1−Month Lag]
A2:Prachuap Khiri Khan
Relative Humidity [2−Month Lag]
B1:Prachuap Khiri Khan
Absolute Humidity (g/m³) [1−Month Lag]
B2:Prachuap Khiri Khan
Absolute Humidity (g/m³) [2−Month Lag]
C1:Prachuap Khiri Khan
Total Precipitation (mm) [1−Month Lag]
C2:Prachuap Khiri Khan
Total Precipitation (mm) [2−Month Lag]
D1:Prachuap Khiri Khan
Temperature (°C) [1−Month Lag]
D2:Prachuap Khiri Khan
Temperature (°C) [2−Month Lag]
E1:Prachuap Khiri Khan
SO_2 Surface Concentration (mg/m³) [1−Month Lag]
E2:Prachuap Khiri Khan
SO_2 Surface Concentration (mg/m³) [2−Month Lag]
F1:Prachuap Khiri Khan
CO Surface Concentration (ppb) [1−Month Lag]
F2:Prachuap Khiri Khan
CO Surface Concentration (ppb) [2−Month Lag]
G1:Prachuap Khiri Khan
$PM_{2.5}$ Surface Concentration (µg/m³) [1−Month Lag]
G2:Prachuap Khiri Khan
$PM_{2.5}$ Surface Concentration (µg/m³) [2−Month Lag]

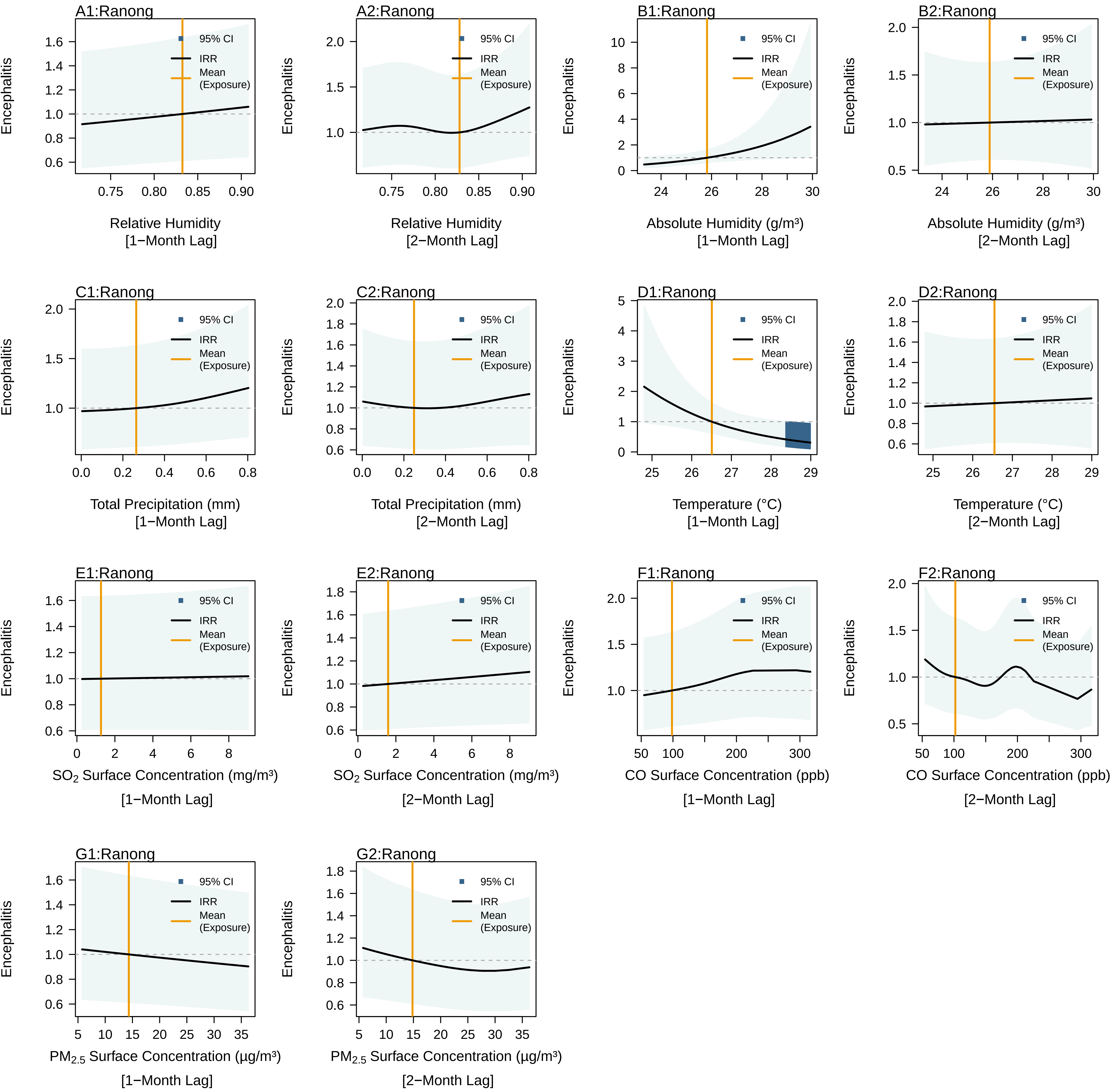
A1:Ranong
Encephalitis
95% CI
IRR
Mean
(Exposure)
Relative Humidity
[1−Month Lag]
A2:Ranong
Encephalitis
Relative Humidity
[2−Month Lag]
B1:Ranong
Encephalitis
Absolute Humidity (g/m³)
[1−Month Lag]
B2:Ranong
Encephalitis
Absolute Humidity (g/m³)
[2−Month Lag]
C1:Ranong
Encephalitis
Total Precipitation (mm)
[1−Month Lag]
C2:Ranong
Encephalitis
Total Precipitation (mm)
[2−Month Lag]
D1:Ranong
Encephalitis
Temperature (°C)
[1−Month Lag]
D2:Ranong
Encephalitis
Temperature (°C)
[2−Month Lag]
E1:Ranong
Encephalitis
SO_2 Surface Concentration (mg/m³)
[1−Month Lag]
E2:Ranong
Encephalitis
SO_2 Surface Concentration (mg/m³)
[2−Month Lag]
F1:Ranong
Encephalitis
CO Surface Concentration (ppb)
[1−Month Lag]
F2:Ranong
Encephalitis
CO Surface Concentration (ppb)
[2−Month Lag]
G1:Ranong
Encephalitis
$PM_{2.5}$ Surface Concentration (µg/m³)
[1−Month Lag]
G2:Ranong
Encephalitis
$PM_{2.5}$ Surface Concentration (µg/m³)
[2−Month Lag]

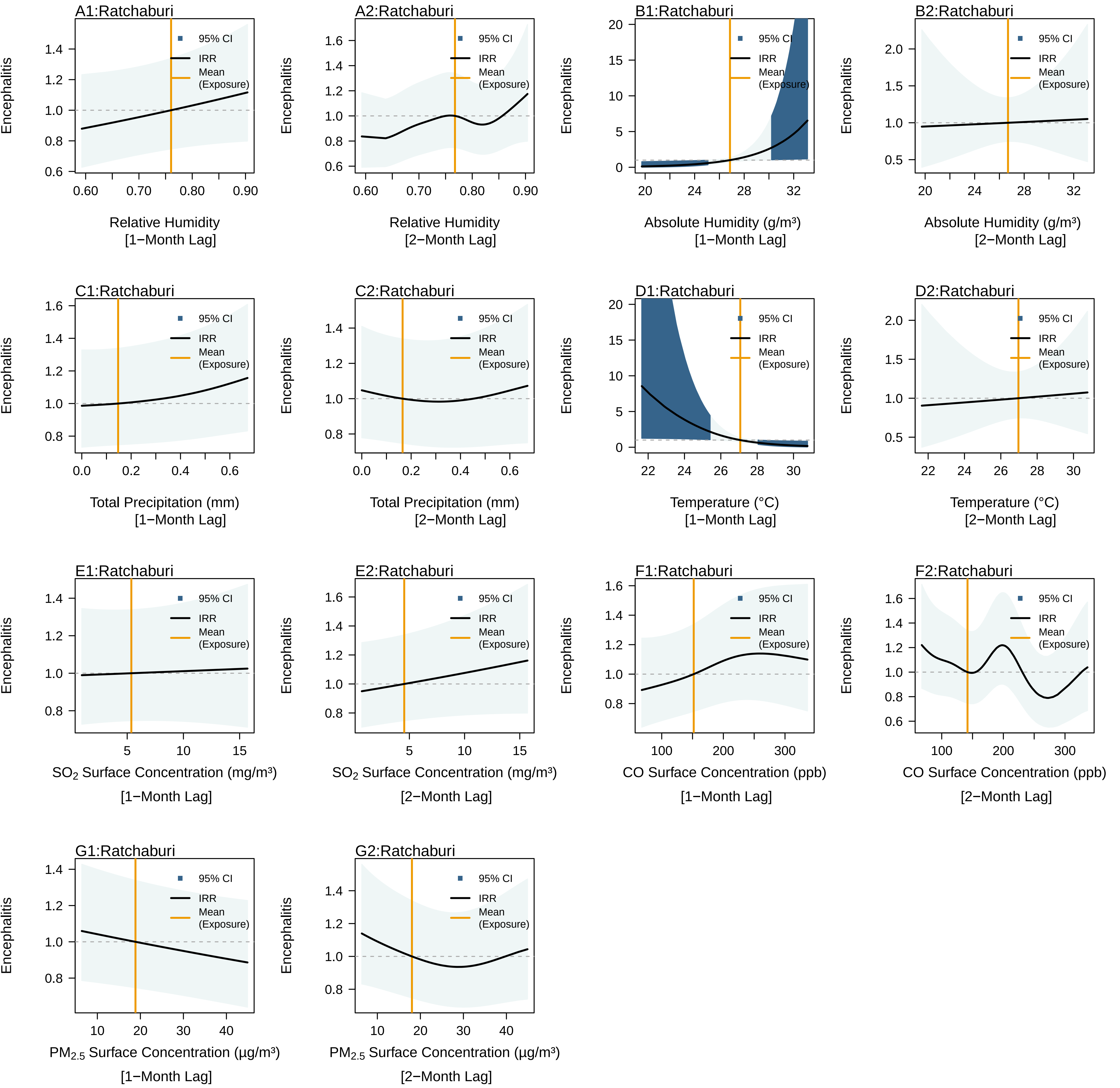

A1:Ratchaburi
Encephalitis
95% CI
IRR
Mean (Exposure)
0.60 0.70 0.80 0.90
Relative Humidity
[1−Month Lag]
A2:Ratchaburi
Encephalitis
95% CI
IRR
Mean (Exposure)
0.60 0.70 0.80 0.90
Relative Humidity
[2−Month Lag]
B1:Ratchaburi
Encephalitis
95% CI
IRR
Mean (Exposure)
20 24 28 32
Absolute Humidity (g/m³)
[1−Month Lag]
B2:Ratchaburi
Encephalitis
95% CI
IRR
Mean (Exposure)
20 24 28 32
Absolute Humidity (g/m³)
[2−Month Lag]
C1:Ratchaburi
Encephalitis
95% CI
IRR
Mean (Exposure)
0.0 0.2 0.4 0.6
Total Precipitation (mm)
[1−Month Lag]
C2:Ratchaburi
Encephalitis
95% CI
IRR
Mean (Exposure)
0.0 0.2 0.4 0.6
Total Precipitation (mm)
[2−Month Lag]
D1:Ratchaburi
Encephalitis
95% CI
IRR
Mean (Exposure)
22 24 26 28 30
Temperature (°C)
[1−Month Lag]
D2:Ratchaburi
Encephalitis
95% CI
IRR
Mean (Exposure)
22 24 26 28 30
Temperature (°C)
[2−Month Lag]
E1:Ratchaburi
Encephalitis
95% CI
IRR
Mean (Exposure)
5 10 15
SO2 Surface Concentration (mg/m³)
[1−Month Lag]
E2:Ratchaburi
Encephalitis
95% CI
IRR
Mean (Exposure)
5 10 15
SO2 Surface Concentration (mg/m³)
[2−Month Lag]
F1:Ratchaburi
Encephalitis
95% CI
IRR
Mean (Exposure)
100 200 300
CO Surface Concentration (ppb)
[1−Month Lag]
F2:Ratchaburi
Encephalitis
95% CI
IRR
Mean (Exposure)
100 200 300
CO Surface Concentration (ppb)
[2−Month Lag]
G1:Ratchaburi
Encephalitis
95% CI
IRR
Mean (Exposure)
10 20 30 40
PM2.5 Surface Concentration (µg/m³)
[1−Month Lag]
G2:Ratchaburi
Encephalitis
95% CI
IRR
Mean (Exposure)
10 20 30 40
PM2.5 Surface Concentration (µg/m³)
[2−Month Lag]

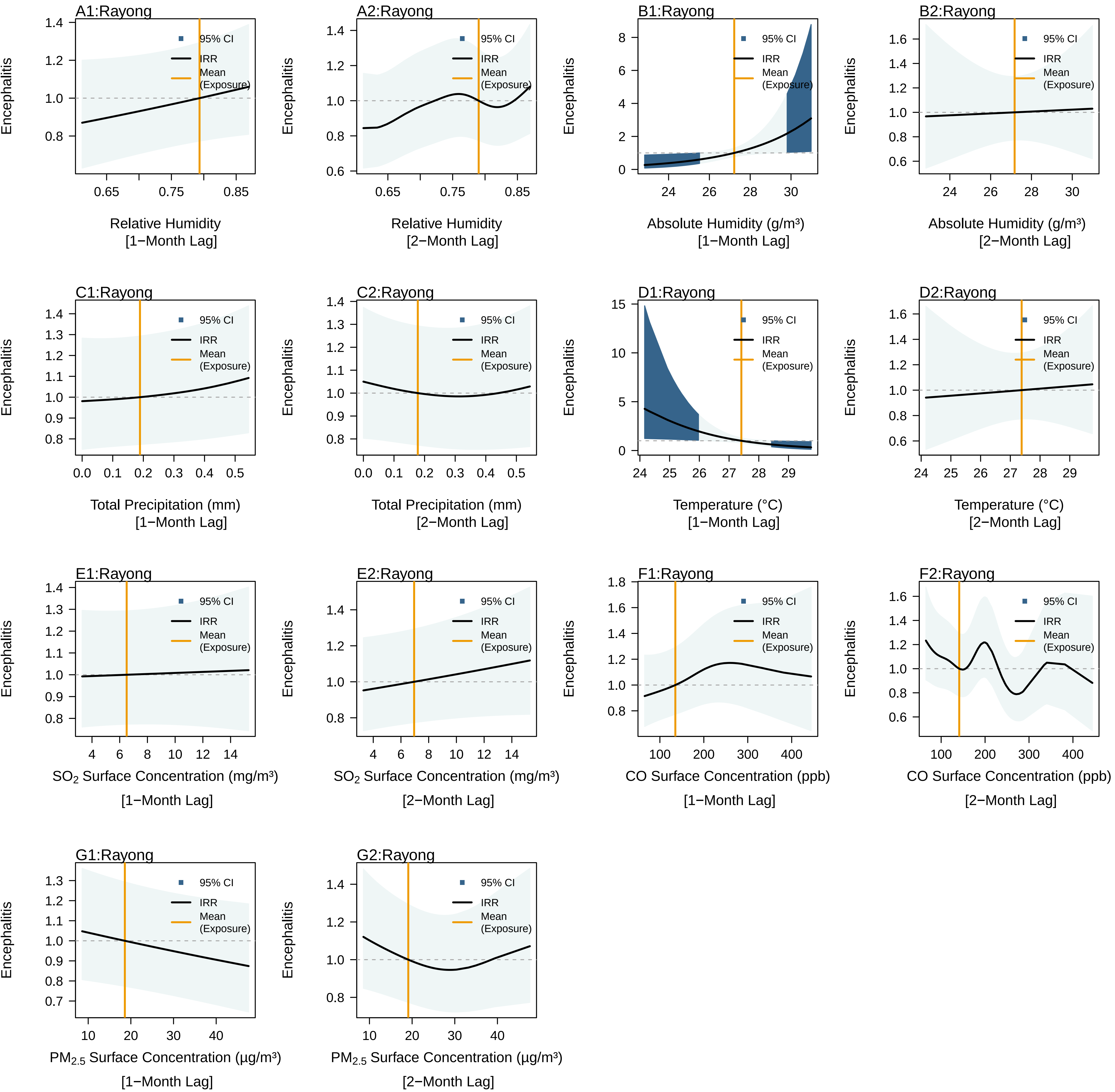
A1:Rayong
Encephalitis
95% CI
IRR
Mean
(Exposure)
Relative Humidity
[1−Month Lag]
A2:Rayong
Encephalitis
Relative Humidity
[2−Month Lag]
B1:Rayong
Encephalitis
Absolute Humidity (g/m³)
[1−Month Lag]
B2:Rayong
Encephalitis
Absolute Humidity (g/m³)
[2−Month Lag]
C1:Rayong
Encephalitis
Total Precipitation (mm)
[1−Month Lag]
C2:Rayong
Encephalitis
Total Precipitation (mm)
[2−Month Lag]
D1:Rayong
Encephalitis
Temperature (°C)
[1−Month Lag]
D2:Rayong
Encephalitis
Temperature (°C)
[2−Month Lag]
E1:Rayong
Encephalitis
SO_2 Surface Concentration (mg/m³)
[1−Month Lag]
E2:Rayong
Encephalitis
SO_2 Surface Concentration (mg/m³)
[2−Month Lag]
F1:Rayong
Encephalitis
CO Surface Concentration (ppb)
[1−Month Lag]
F2:Rayong
Encephalitis
CO Surface Concentration (ppb)
[2−Month Lag]
G1:Rayong
Encephalitis
$PM_{2.5}$ Surface Concentration (µg/m³)
[1−Month Lag]
G2:Rayong
Encephalitis
$PM_{2.5}$ Surface Concentration (µg/m³)
[2−Month Lag]

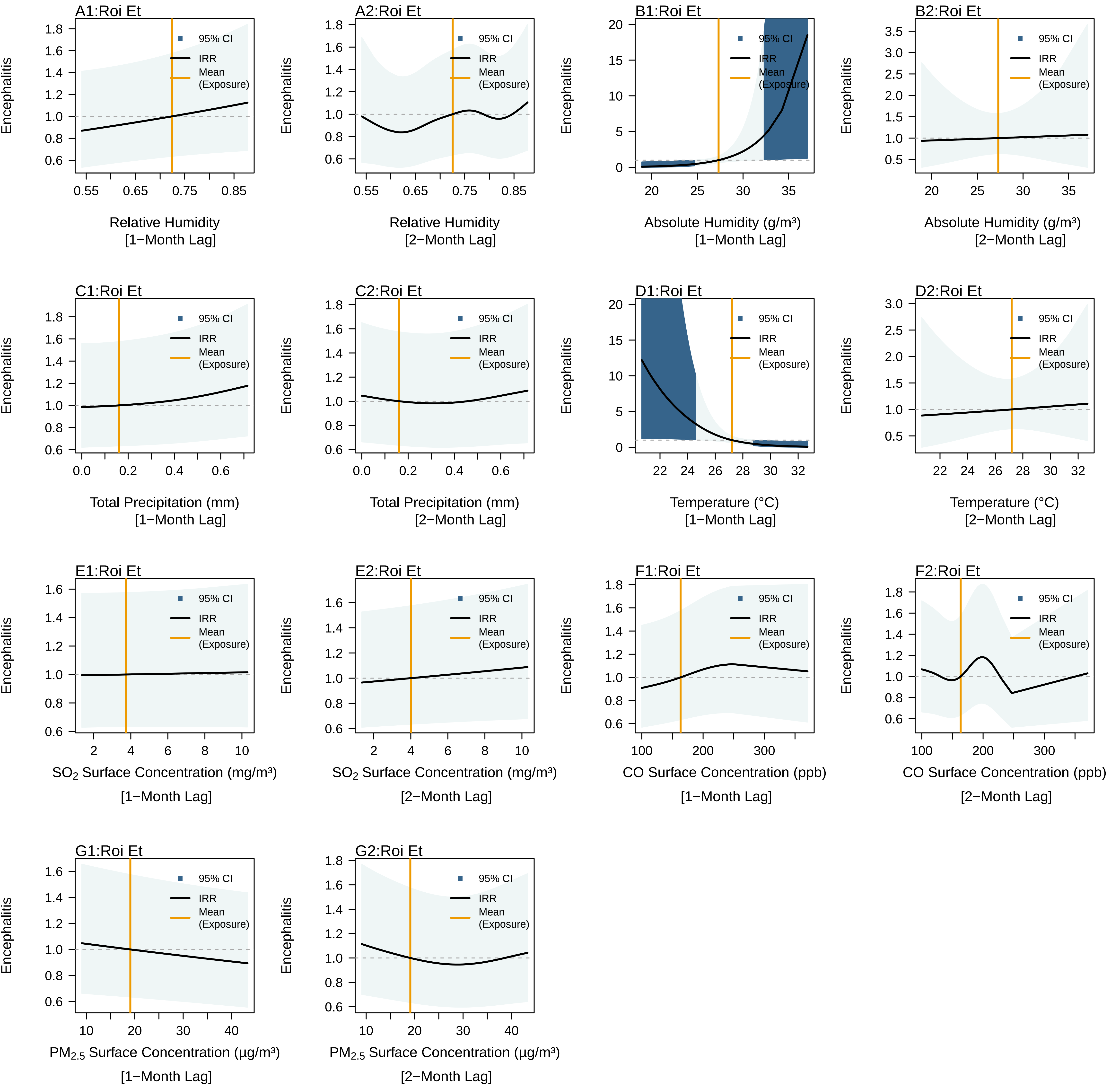
A1:Roi Et
A2:Roi Et
B1:Roi Et
B2:Roi Et
C1:Roi Et
C2:Roi Et
D1:Roi Et
D2:Roi Et
E1:Roi Et
E2:Roi Et
F1:Roi Et
F2:Roi Et
G1:Roi Et
G2:Roi Et
Encephalitis
95% CI
IRR
Mean (Exposure)
Relative Humidity [1−Month Lag]
Relative Humidity [2−Month Lag]
Absolute Humidity (g/m³) [1−Month Lag]
Absolute Humidity (g/m³) [2−Month Lag]
Total Precipitation (mm) [1−Month Lag]
Total Precipitation (mm) [2−Month Lag]
Temperature (°C) [1−Month Lag]
Temperature (°C) [2−Month Lag]
SO_2 Surface Concentration (mg/m³) [1−Month Lag]
SO_2 Surface Concentration (mg/m³) [2−Month Lag]
CO Surface Concentration (ppb) [1−Month Lag]
CO Surface Concentration (ppb) [2−Month Lag]
$PM_{2.5}$ Surface Concentration (µg/m³) [1−Month Lag]
$PM_{2.5}$ Surface Concentration (µg/m³) [2−Month Lag]

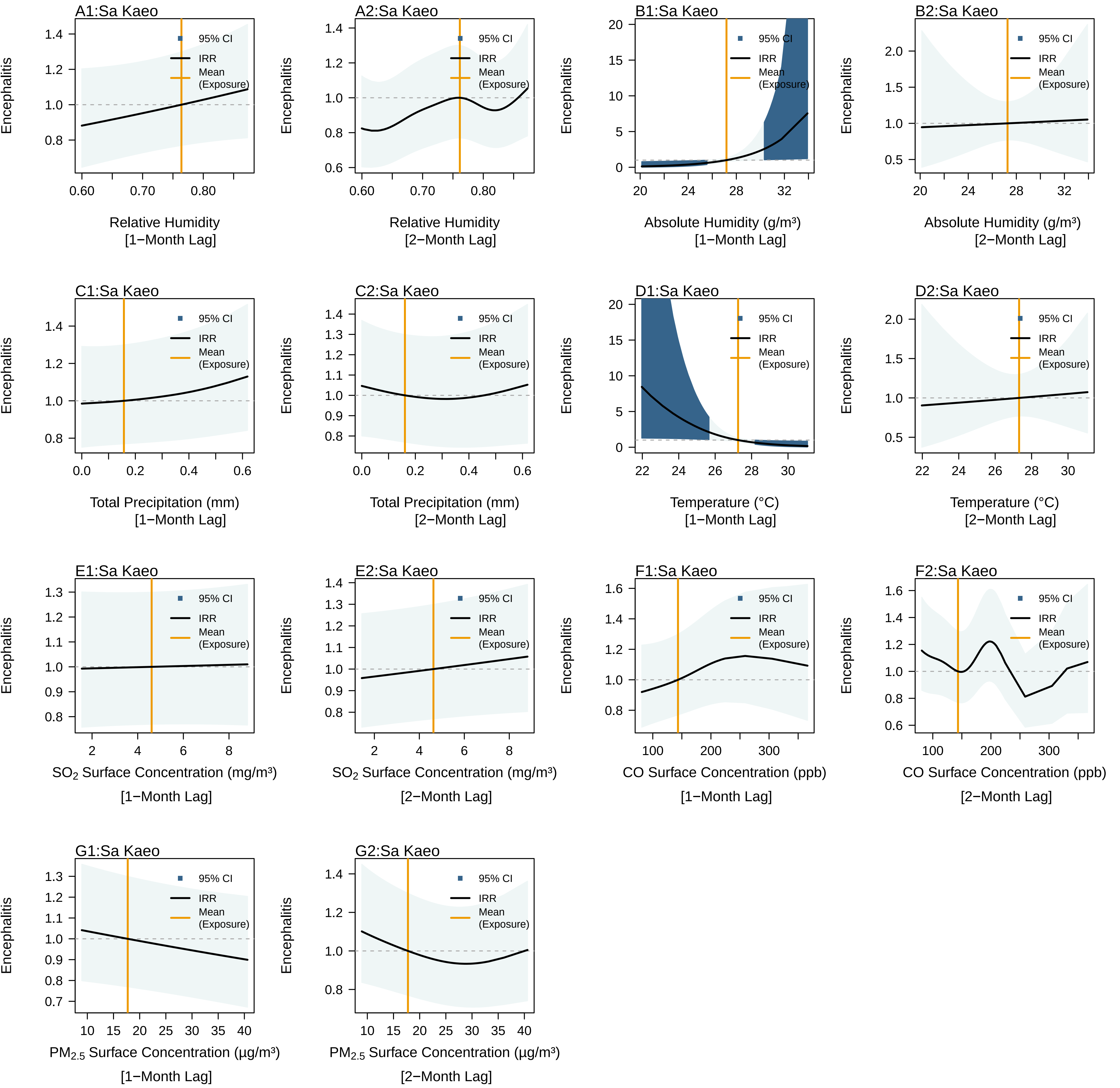

A1:Sa Kaeo
95% CI
IRR
Mean (Exposure)
Encephalitis
Relative Humidity [1−Month Lag]
A2:Sa Kaeo
Relative Humidity [2−Month Lag]
B1:Sa Kaeo
Absolute Humidity (g/m³) [1−Month Lag]
B2:Sa Kaeo
Absolute Humidity (g/m³) [2−Month Lag]
C1:Sa Kaeo
Total Precipitation (mm) [1−Month Lag]
C2:Sa Kaeo
Total Precipitation (mm) [2−Month Lag]
D1:Sa Kaeo
Temperature (°C) [1−Month Lag]
D2:Sa Kaeo
Temperature (°C) [2−Month Lag]
E1:Sa Kaeo
SO_2 Surface Concentration (mg/m³) [1−Month Lag]
E2:Sa Kaeo
SO_2 Surface Concentration (mg/m³) [2−Month Lag]
F1:Sa Kaeo
CO Surface Concentration (ppb) [1−Month Lag]
F2:Sa Kaeo
CO Surface Concentration (ppb) [2−Month Lag]
G1:Sa Kaeo
$PM_{2.5}$ Surface Concentration (µg/m³) [1−Month Lag]
G2:Sa Kaeo
$PM_{2.5}$ Surface Concentration (µg/m³) [2−Month Lag]

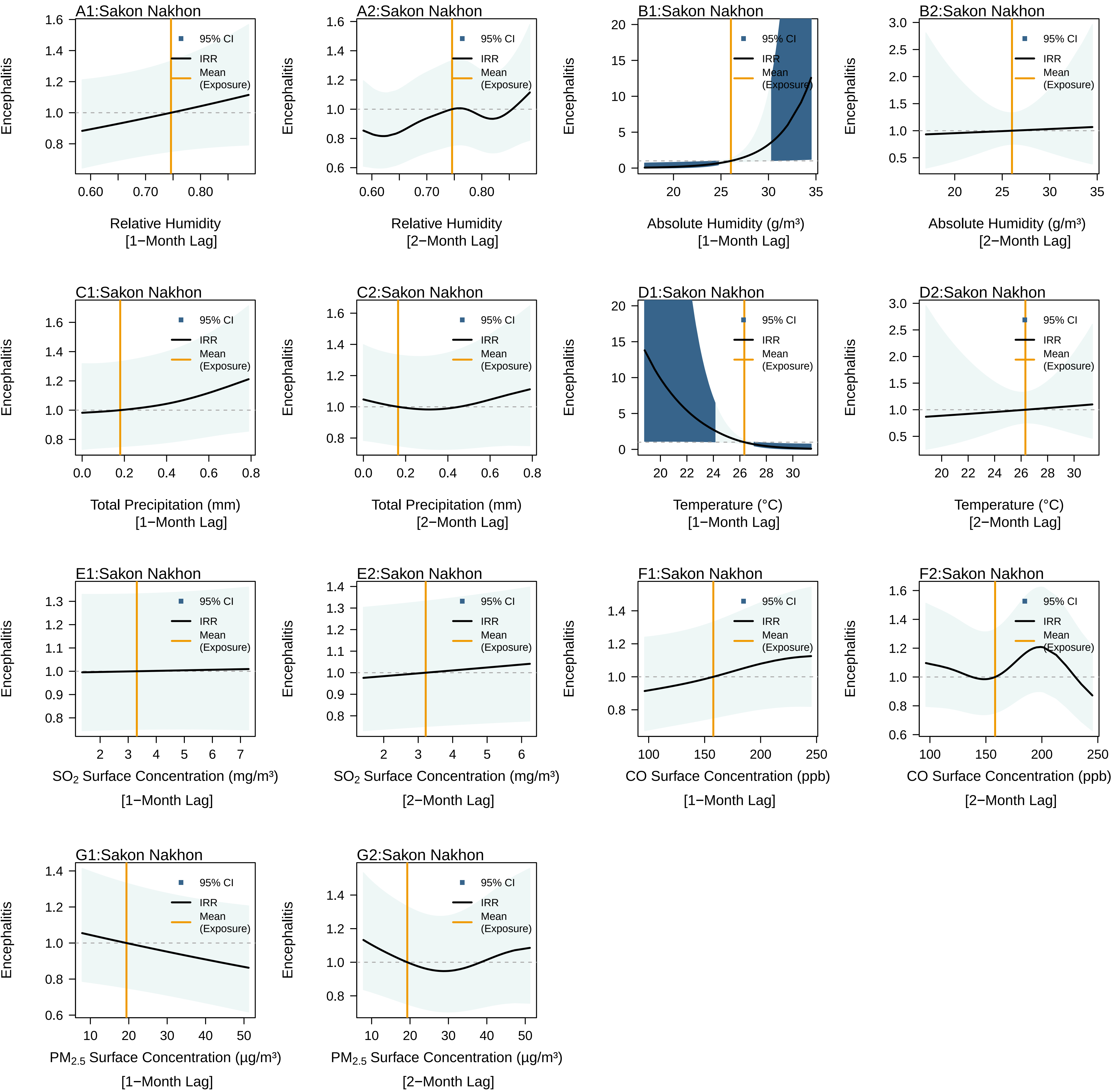
A1:Sakon Nakhon
A2:Sakon Nakhon
B1:Sakon Nakhon
B2:Sakon Nakhon
C1:Sakon Nakhon
C2:Sakon Nakhon
D1:Sakon Nakhon
D2:Sakon Nakhon
E1:Sakon Nakhon
E2:Sakon Nakhon
F1:Sakon Nakhon
F2:Sakon Nakhon
G1:Sakon Nakhon
G2:Sakon Nakhon
Encephalitis
95% CI
IRR
Mean
(Exposure)
Relative Humidity
[1−Month Lag]
Relative Humidity
[2−Month Lag]
Absolute Humidity (g/m³)
[1−Month Lag]
Absolute Humidity (g/m³)
[2−Month Lag]
Total Precipitation (mm)
[1−Month Lag]
Total Precipitation (mm)
[2−Month Lag]
Temperature (°C)
[1−Month Lag]
Temperature (°C)
[2−Month Lag]
SO_2 Surface Concentration (mg/m³)
[1−Month Lag]
SO_2 Surface Concentration (mg/m³)
[2−Month Lag]
CO Surface Concentration (ppb)
[1−Month Lag]
CO Surface Concentration (ppb)
[2−Month Lag]
$PM_{2.5}$ Surface Concentration (µg/m³)
[1−Month Lag]
$PM_{2.5}$ Surface Concentration (µg/m³)
[2−Month Lag]

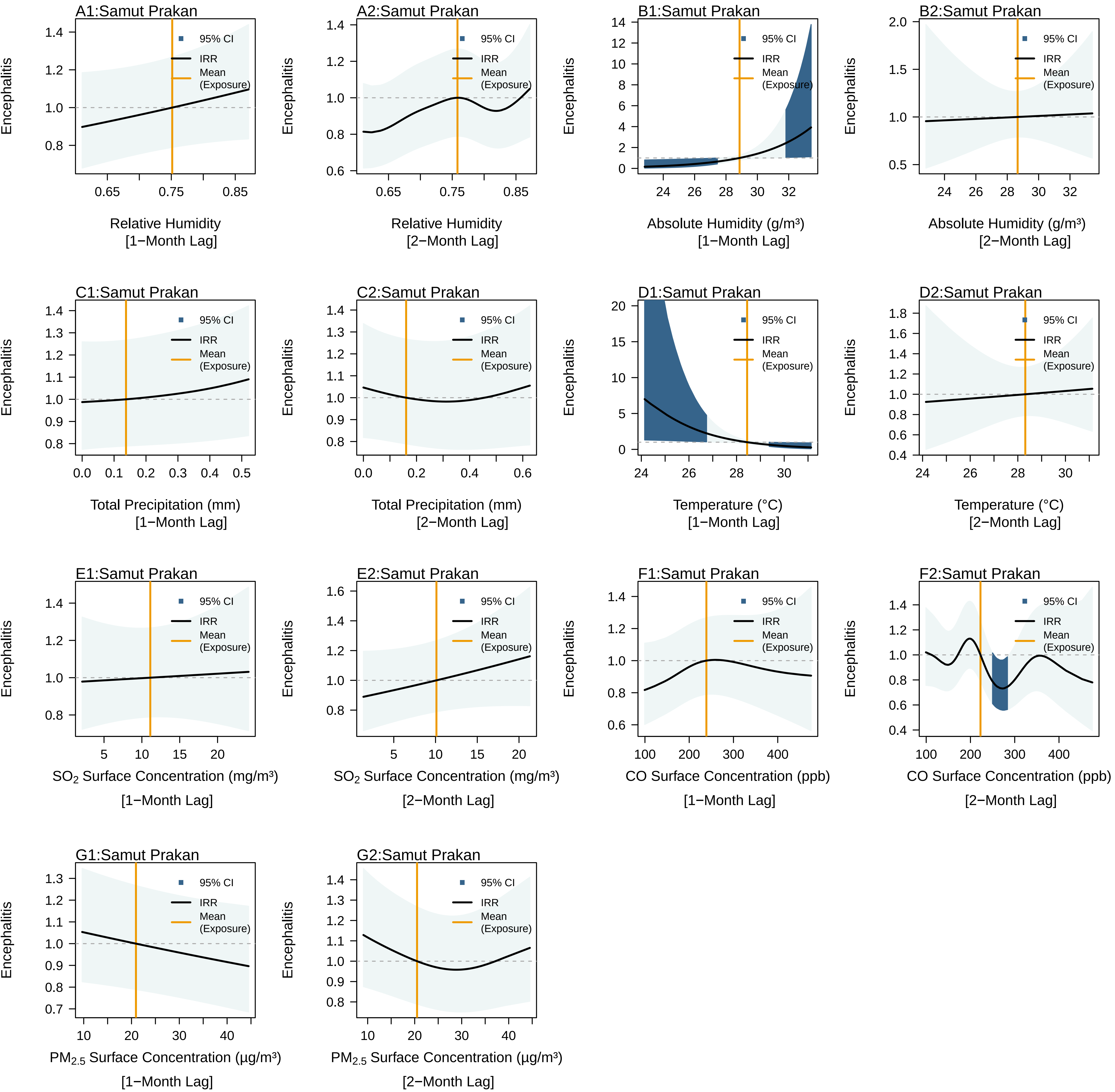
A1:Samut Prakan
Encephalitis
95% CI
IRR
Mean
(Exposure)
Relative Humidity
[1−Month Lag]
A2:Samut Prakan
Relative Humidity
[2−Month Lag]
B1:Samut Prakan
Absolute Humidity (g/m³)
[1−Month Lag]
B2:Samut Prakan
Absolute Humidity (g/m³)
[2−Month Lag]
C1:Samut Prakan
Total Precipitation (mm)
[1−Month Lag]
C2:Samut Prakan
Total Precipitation (mm)
[2−Month Lag]
D1:Samut Prakan
Temperature (°C)
[1−Month Lag]
D2:Samut Prakan
Temperature (°C)
[2−Month Lag]
E1:Samut Prakan
SO_2 Surface Concentration (mg/m³)
[1−Month Lag]
E2:Samut Prakan
SO_2 Surface Concentration (mg/m³)
[2−Month Lag]
F1:Samut Prakan
CO Surface Concentration (ppb)
[1−Month Lag]
F2:Samut Prakan
CO Surface Concentration (ppb)
[2−Month Lag]
G1:Samut Prakan
$PM_{2.5}$ Surface Concentration (µg/m³)
[1−Month Lag]
G2:Samut Prakan
$PM_{2.5}$ Surface Concentration (µg/m³)
[2−Month Lag]

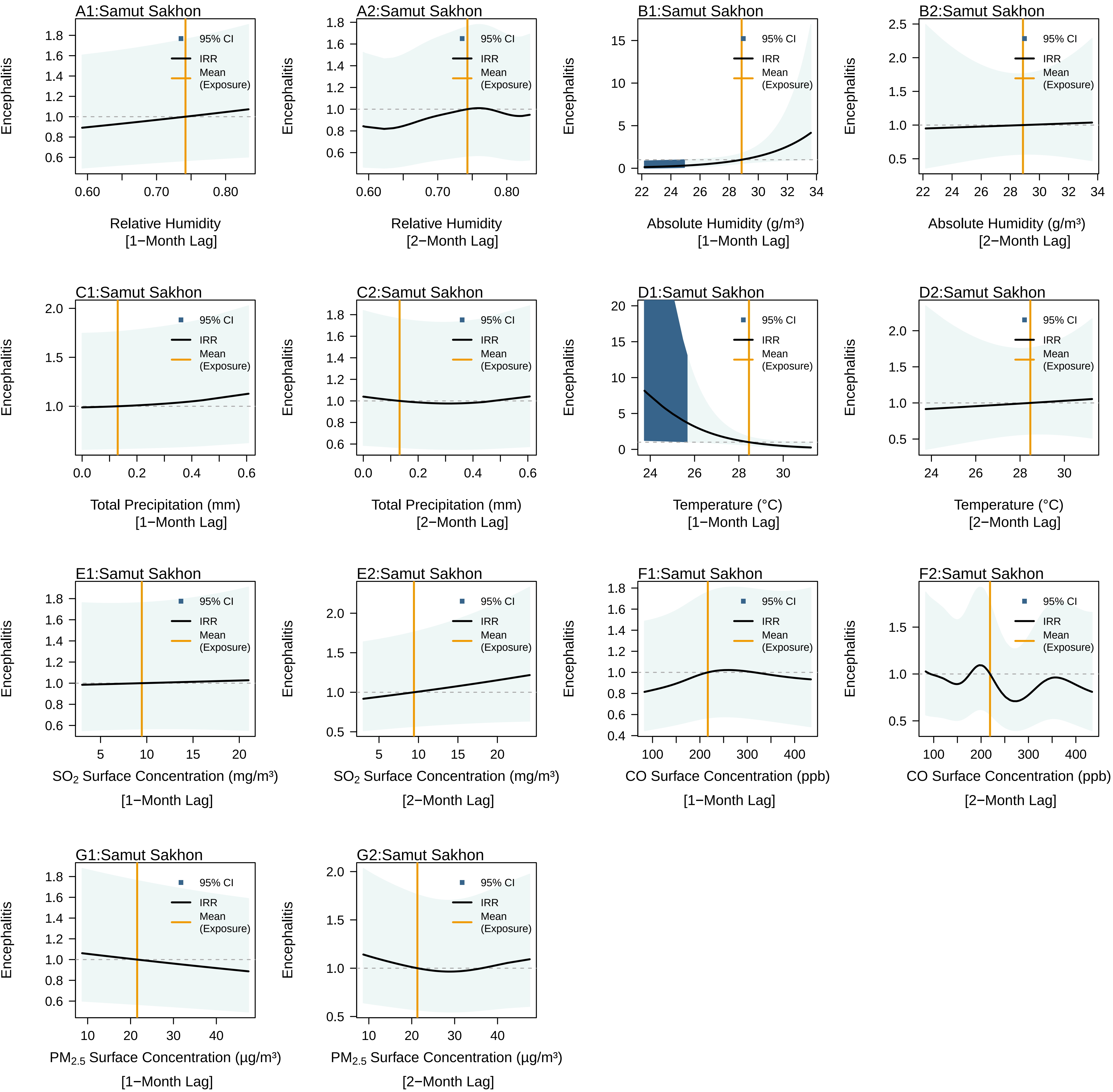
A1:Samut Sakhon
Encephalitis
95% CI
IRR
Mean (Exposure)
Relative Humidity [1−Month Lag]
A2:Samut Sakhon
Relative Humidity [2−Month Lag]
B1:Samut Sakhon
Absolute Humidity (g/m³) [1−Month Lag]
B2:Samut Sakhon
Absolute Humidity (g/m³) [2−Month Lag]
C1:Samut Sakhon
Total Precipitation (mm) [1−Month Lag]
C2:Samut Sakhon
Total Precipitation (mm) [2−Month Lag]
D1:Samut Sakhon
Temperature (°C) [1−Month Lag]
D2:Samut Sakhon
Temperature (°C) [2−Month Lag]
E1:Samut Sakhon
SO2 Surface Concentration (mg/m³) [1−Month Lag]
E2:Samut Sakhon
SO2 Surface Concentration (mg/m³) [2−Month Lag]
F1:Samut Sakhon
CO Surface Concentration (ppb) [1−Month Lag]
F2:Samut Sakhon
CO Surface Concentration (ppb) [2−Month Lag]
G1:Samut Sakhon
PM2.5 Surface Concentration (μg/m³) [1−Month Lag]
G2:Samut Sakhon
PM2.5 Surface Concentration (μg/m³) [2−Month Lag]

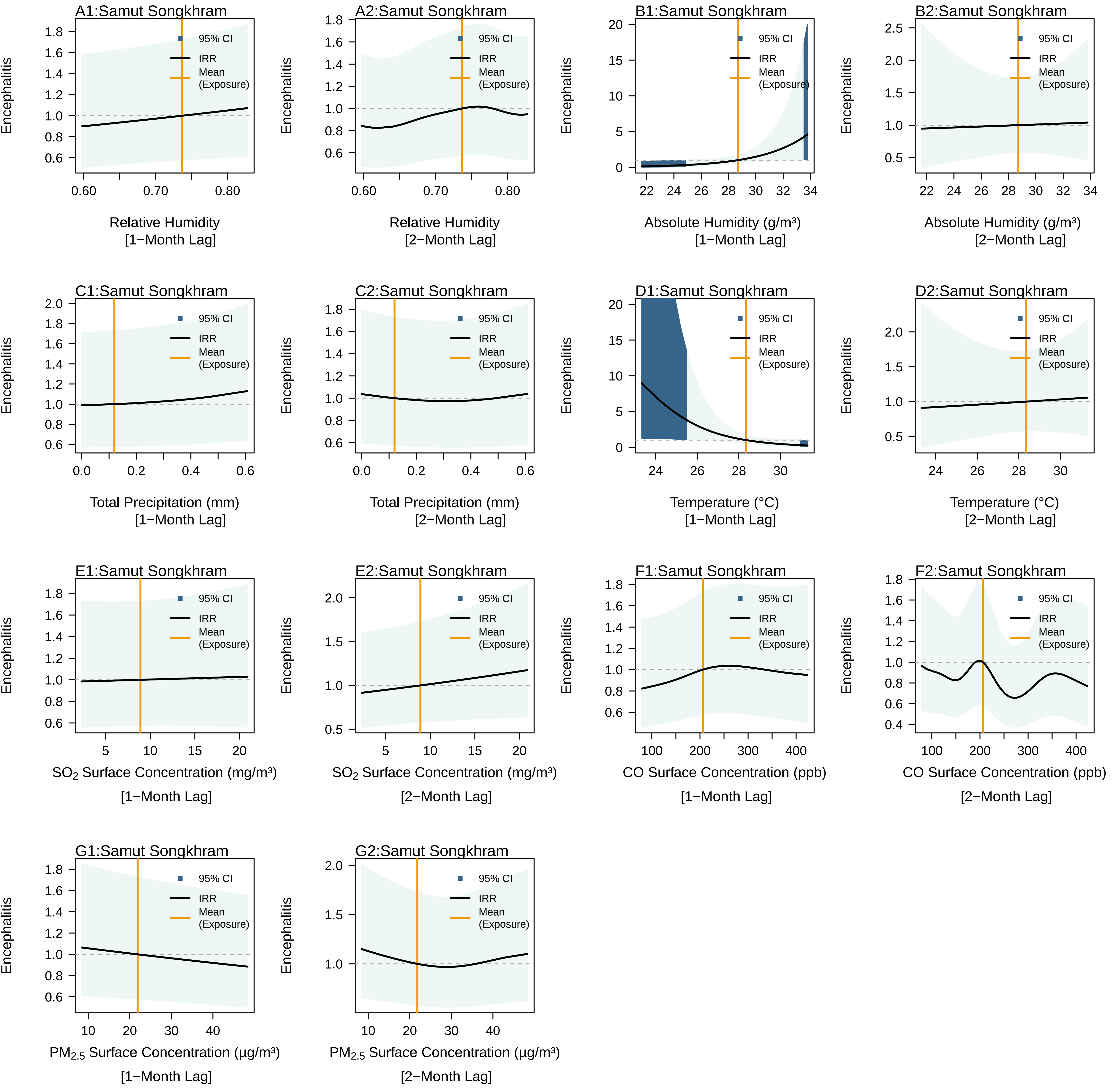
A1:Samut Songkhram
95% CI
IRR
Mean (Exposure)
Encephalitis
Relative Humidity [1−Month Lag]
A2:Samut Songkhram
Relative Humidity [2−Month Lag]
B1:Samut Songkhram
Absolute Humidity (g/m³) [1−Month Lag]
B2:Samut Songkhram
Absolute Humidity (g/m³) [2−Month Lag]
C1:Samut Songkhram
Total Precipitation (mm) [1−Month Lag]
C2:Samut Songkhram
Total Precipitation (mm) [2−Month Lag]
D1:Samut Songkhram
Temperature (°C) [1−Month Lag]
D2:Samut Songkhram
Temperature (°C) [2−Month Lag]
E1:Samut Songkhram
SO_2 Surface Concentration (mg/m³) [1−Month Lag]
E2:Samut Songkhram
SO_2 Surface Concentration (mg/m³) [2−Month Lag]
F1:Samut Songkhram
CO Surface Concentration (ppb) [1−Month Lag]
F2:Samut Songkhram
CO Surface Concentration (ppb) [2−Month Lag]
G1:Samut Songkhram
$PM_{2.5}$ Surface Concentration (μg/m³) [1−Month Lag]
G2:Samut Songkhram
$PM_{2.5}$ Surface Concentration (μg/m³) [2−Month Lag]

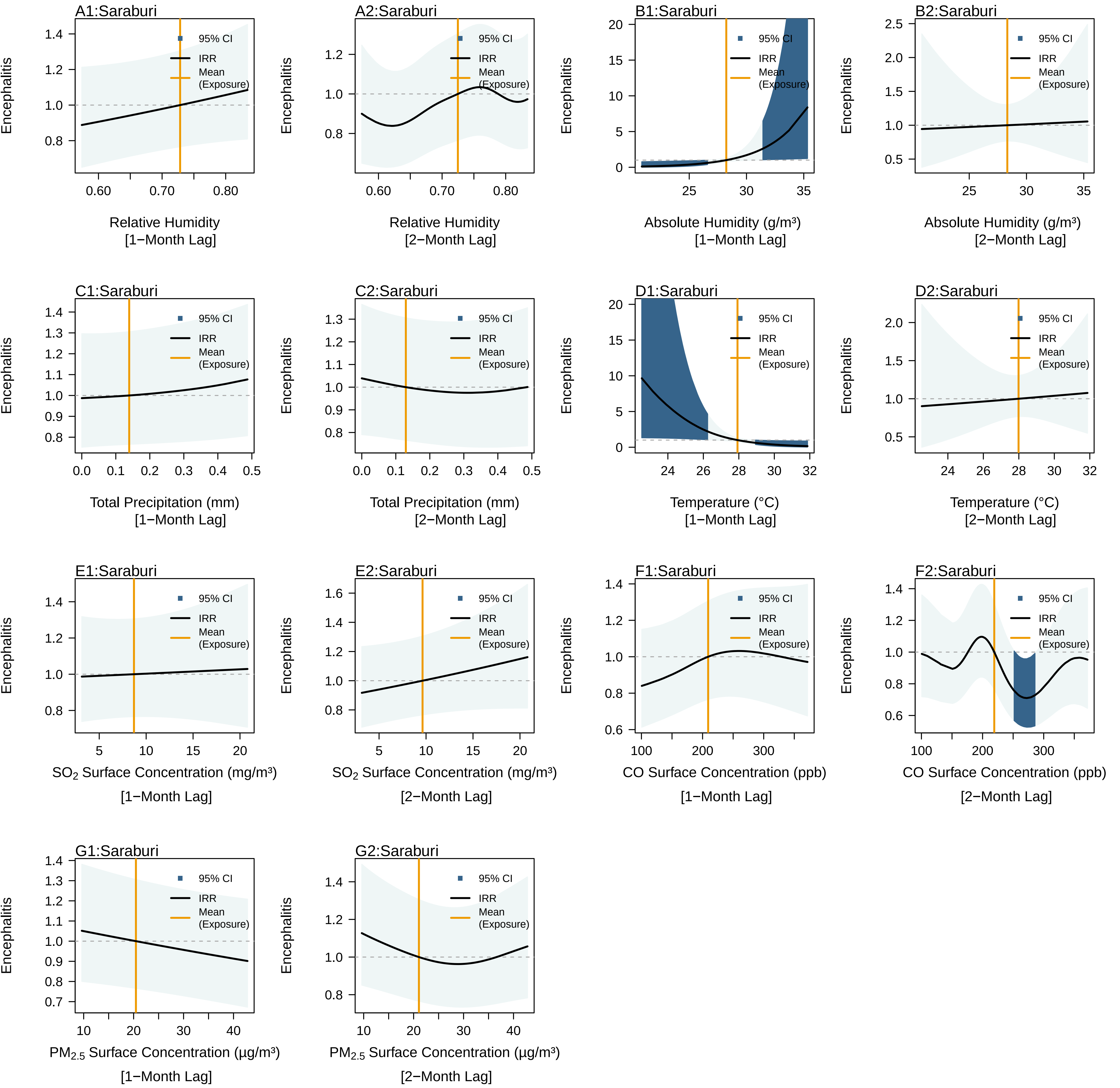
A1:Saraburi
Encephalitis
Relative Humidity [1−Month Lag]
A2:Saraburi
Relative Humidity [2−Month Lag]
B1:Saraburi
Absolute Humidity (g/m³) [1−Month Lag]
B2:Saraburi
Absolute Humidity (g/m³) [2−Month Lag]
C1:Saraburi
Total Precipitation (mm) [1−Month Lag]
C2:Saraburi
Total Precipitation (mm) [2−Month Lag]
D1:Saraburi
Temperature (°C) [1−Month Lag]
D2:Saraburi
Temperature (°C) [2−Month Lag]
E1:Saraburi
SO_2 Surface Concentration (mg/m³) [1−Month Lag]
E2:Saraburi
SO_2 Surface Concentration (mg/m³) [2−Month Lag]
F1:Saraburi
CO Surface Concentration (ppb) [1−Month Lag]
F2:Saraburi
CO Surface Concentration (ppb) [2−Month Lag]
G1:Saraburi
$PM_{2.5}$ Surface Concentration (µg/m³) [1−Month Lag]
G2:Saraburi
$PM_{2.5}$ Surface Concentration (µg/m³) [2−Month Lag]
95% CI
IRR
Mean (Exposure)

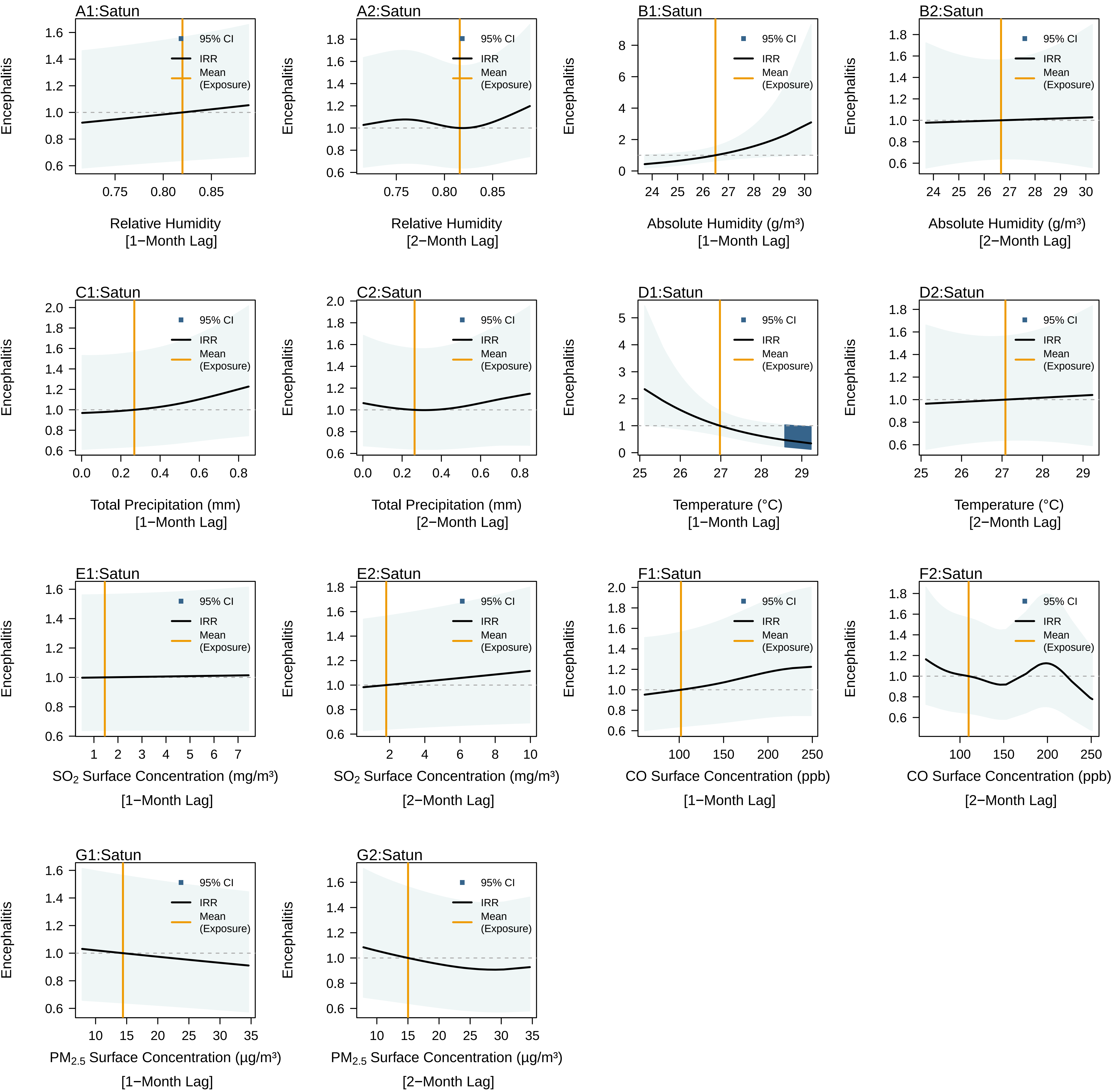
A1:Satun
Encephalitis
95% CI
IRR
Mean (Exposure)
Relative Humidity [1−Month Lag]
A2:Satun
Relative Humidity [2−Month Lag]
B1:Satun
Absolute Humidity (g/m³) [1−Month Lag]
B2:Satun
Absolute Humidity (g/m³) [2−Month Lag]
C1:Satun
Total Precipitation (mm) [1−Month Lag]
C2:Satun
Total Precipitation (mm) [2−Month Lag]
D1:Satun
Temperature (°C) [1−Month Lag]
D2:Satun
Temperature (°C) [2−Month Lag]
E1:Satun
SO2 Surface Concentration (mg/m³) [1−Month Lag]
E2:Satun
SO2 Surface Concentration (mg/m³) [2−Month Lag]
F1:Satun
CO Surface Concentration (ppb) [1−Month Lag]
F2:Satun
CO Surface Concentration (ppb) [2−Month Lag]
G1:Satun
PM2.5 Surface Concentration (µg/m³) [1−Month Lag]
G2:Satun
PM2.5 Surface Concentration (µg/m³) [2−Month Lag]

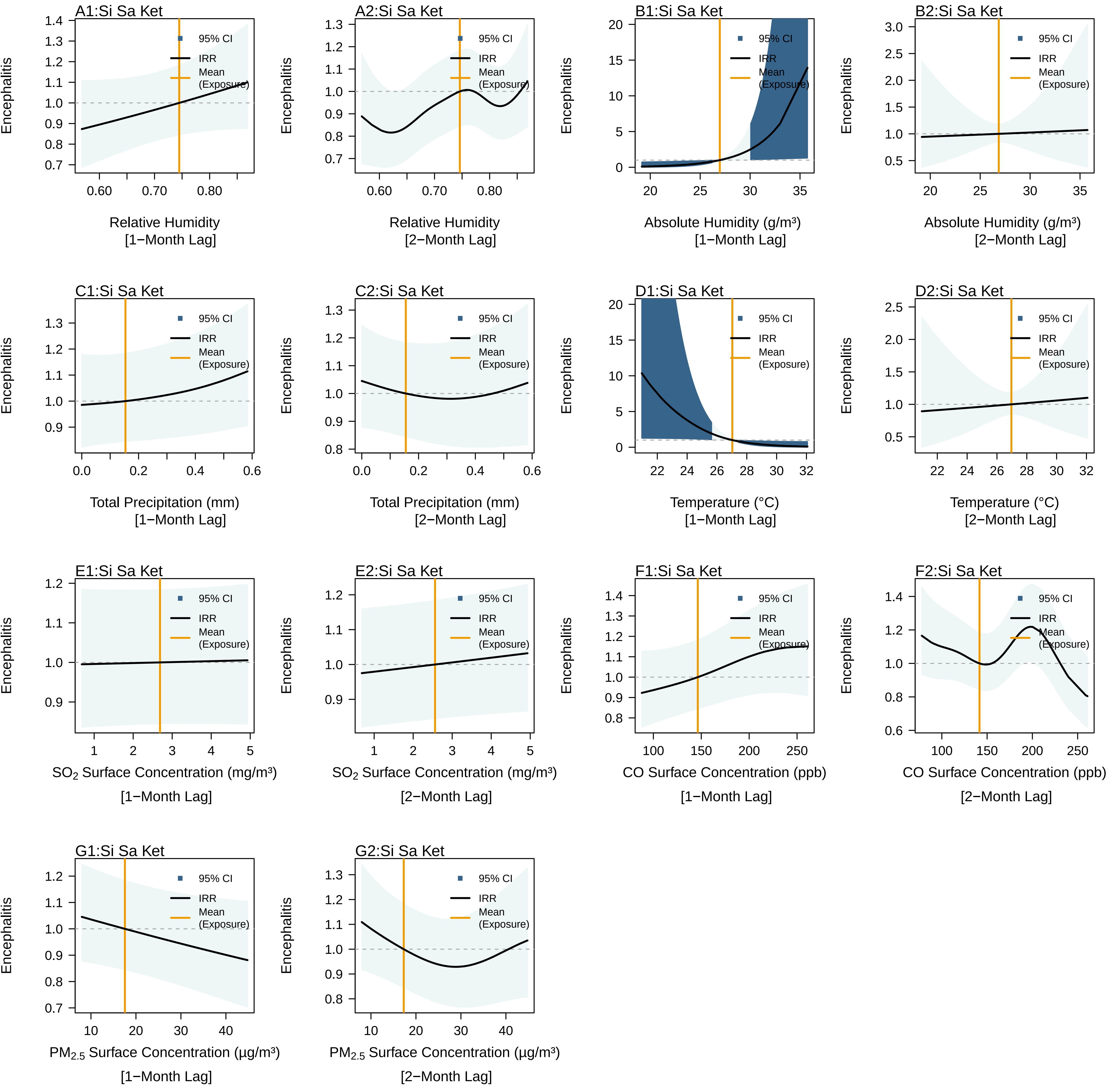

A1:Si Sa Ket
A2:Si Sa Ket
B1:Si Sa Ket
B2:Si Sa Ket
C1:Si Sa Ket
C2:Si Sa Ket
D1:Si Sa Ket
D2:Si Sa Ket
E1:Si Sa Ket
E2:Si Sa Ket
F1:Si Sa Ket
F2:Si Sa Ket
G1:Si Sa Ket
G2:Si Sa Ket
95% CI
IRR
Mean (Exposure)
Encephalitis
Relative Humidity [1−Month Lag]
Relative Humidity [2−Month Lag]
Absolute Humidity (g/m³) [1−Month Lag]
Absolute Humidity (g/m³) [2−Month Lag]
Total Precipitation (mm) [1−Month Lag]
Total Precipitation (mm) [2−Month Lag]
Temperature (°C) [1−Month Lag]
Temperature (°C) [2−Month Lag]
SO2 Surface Concentration (mg/m³) [1−Month Lag]
SO2 Surface Concentration (mg/m³) [2−Month Lag]
CO Surface Concentration (ppb) [1−Month Lag]
CO Surface Concentration (ppb) [2−Month Lag]
PM2.5 Surface Concentration (µg/m³) [1−Month Lag]
PM2.5 Surface Concentration (µg/m³) [2−Month Lag]

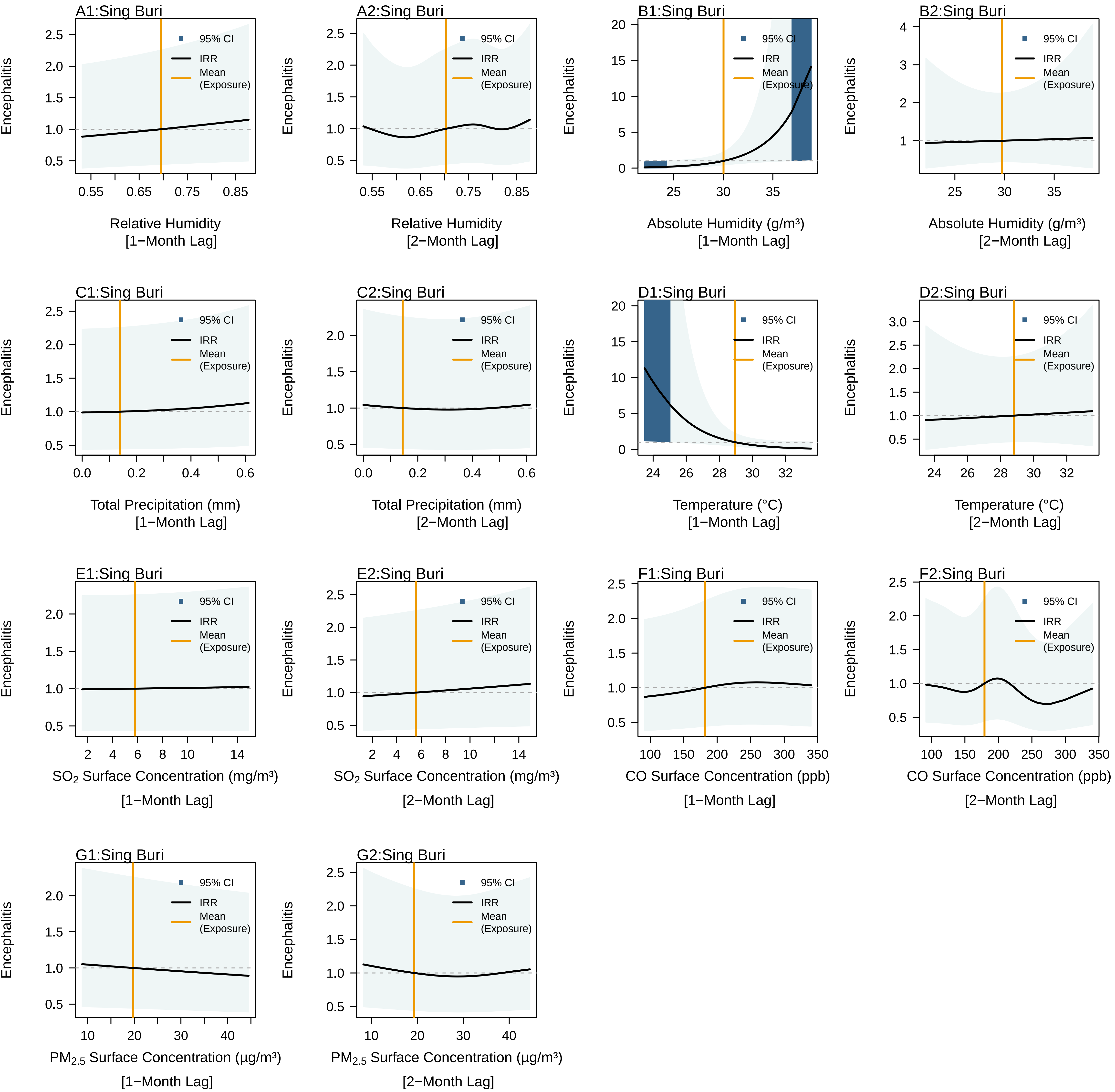
A1:Sing Buri
Encephalitis
95% CI
IRR
Mean (Exposure)
Relative Humidity [1−Month Lag]
A2:Sing Buri
Relative Humidity [2−Month Lag]
B1:Sing Buri
Absolute Humidity (g/m³) [1−Month Lag]
B2:Sing Buri
Absolute Humidity (g/m³) [2−Month Lag]
C1:Sing Buri
Total Precipitation (mm) [1−Month Lag]
C2:Sing Buri
Total Precipitation (mm) [2−Month Lag]
D1:Sing Buri
Temperature (°C) [1−Month Lag]
D2:Sing Buri
Temperature (°C) [2−Month Lag]
E1:Sing Buri
SO_2 Surface Concentration (mg/m³) [1−Month Lag]
E2:Sing Buri
SO_2 Surface Concentration (mg/m³) [2−Month Lag]
F1:Sing Buri
CO Surface Concentration (ppb) [1−Month Lag]
F2:Sing Buri
CO Surface Concentration (ppb) [2−Month Lag]
G1:Sing Buri
$PM_{2.5}$ Surface Concentration (µg/m³) [1−Month Lag]
G2:Sing Buri
$PM_{2.5}$ Surface Concentration (µg/m³) [2−Month Lag]

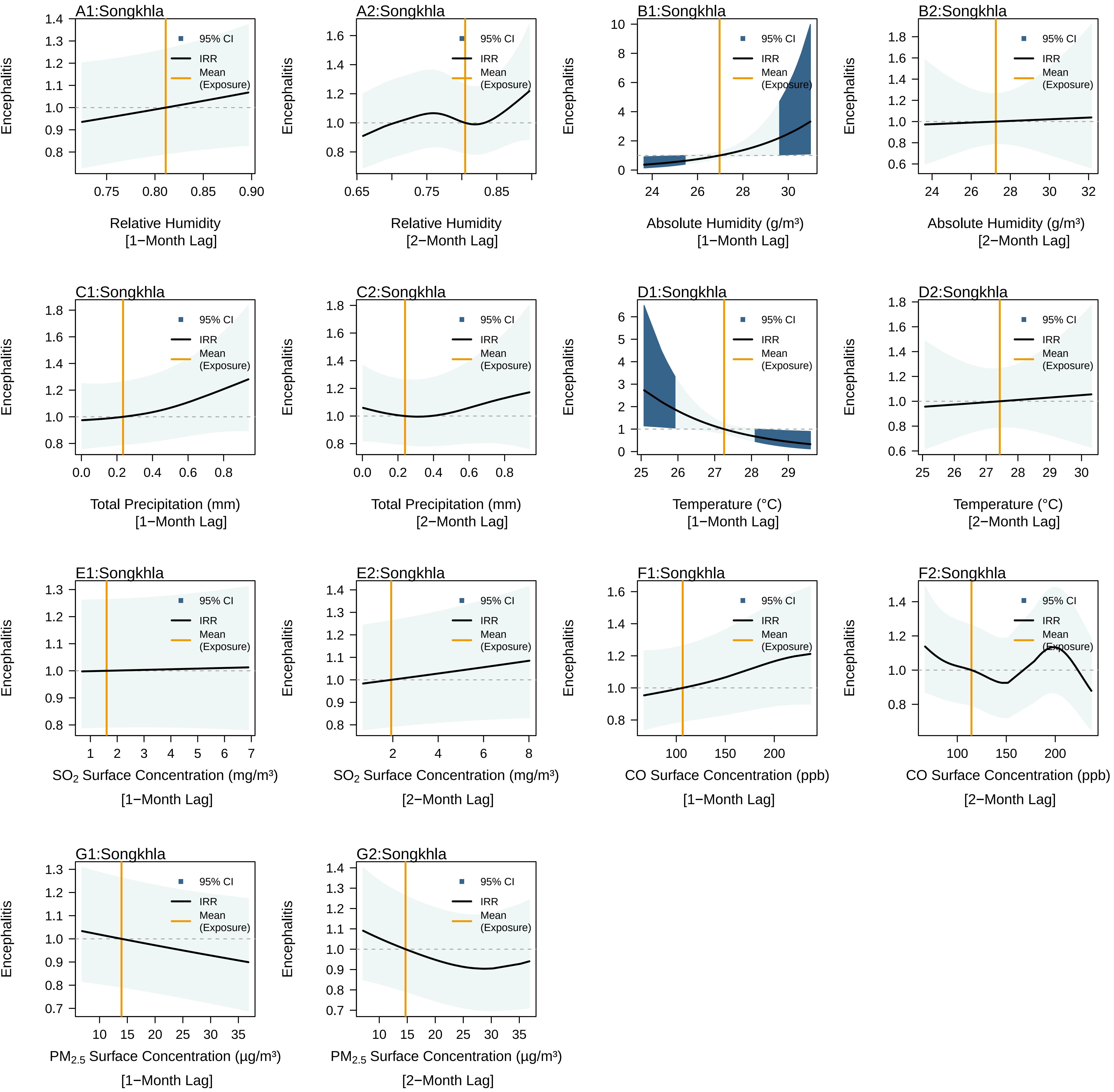
A1:Songkhla
A2:Songkhla
B1:Songkhla
B2:Songkhla
C1:Songkhla
C2:Songkhla
D1:Songkhla
D2:Songkhla
E1:Songkhla
E2:Songkhla
F1:Songkhla
F2:Songkhla
G1:Songkhla
G2:Songkhla
Encephalitis
95% CI
IRR
Mean (Exposure)
Relative Humidity [1−Month Lag]
Relative Humidity [2−Month Lag]
Absolute Humidity (g/m³) [1−Month Lag]
Absolute Humidity (g/m³) [2−Month Lag]
Total Precipitation (mm) [1−Month Lag]
Total Precipitation (mm) [2−Month Lag]
Temperature (°C) [1−Month Lag]
Temperature (°C) [2−Month Lag]
SO2 Surface Concentration (mg/m³) [1−Month Lag]
SO2 Surface Concentration (mg/m³) [2−Month Lag]
CO Surface Concentration (ppb) [1−Month Lag]
CO Surface Concentration (ppb) [2−Month Lag]
PM2.5 Surface Concentration (µg/m³) [1−Month Lag]
PM2.5 Surface Concentration (µg/m³) [2−Month Lag]

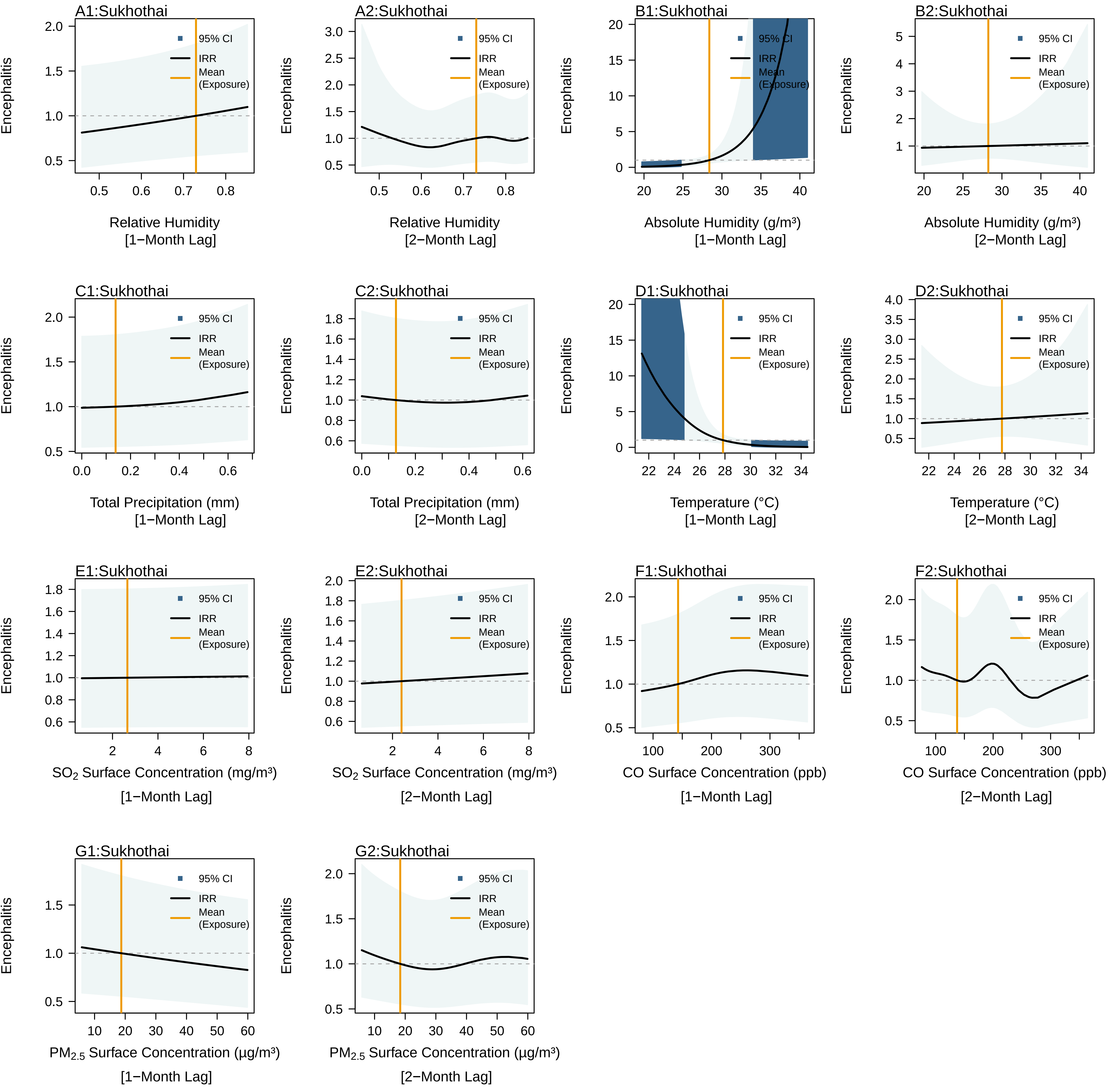
A1:Sukhothai
A2:Sukhothai
B1:Sukhothai
B2:Sukhothai
C1:Sukhothai
C2:Sukhothai
D1:Sukhothai
D2:Sukhothai
E1:Sukhothai
E2:Sukhothai
F1:Sukhothai
F2:Sukhothai
G1:Sukhothai
G2:Sukhothai
95% CI
IRR
Mean (Exposure)
Encephalitis
Relative Humidity [1−Month Lag]
Relative Humidity [2−Month Lag]
Absolute Humidity (g/m³) [1−Month Lag]
Absolute Humidity (g/m³) [2−Month Lag]
Total Precipitation (mm) [1−Month Lag]
Total Precipitation (mm) [2−Month Lag]
Temperature (°C) [1−Month Lag]
Temperature (°C) [2−Month Lag]
SO_2 Surface Concentration (mg/m³) [1−Month Lag]
SO_2 Surface Concentration (mg/m³) [2−Month Lag]
CO Surface Concentration (ppb) [1−Month Lag]
CO Surface Concentration (ppb) [2−Month Lag]
$PM_{2.5}$ Surface Concentration (µg/m³) [1−Month Lag]
$PM_{2.5}$ Surface Concentration (µg/m³) [2−Month Lag]

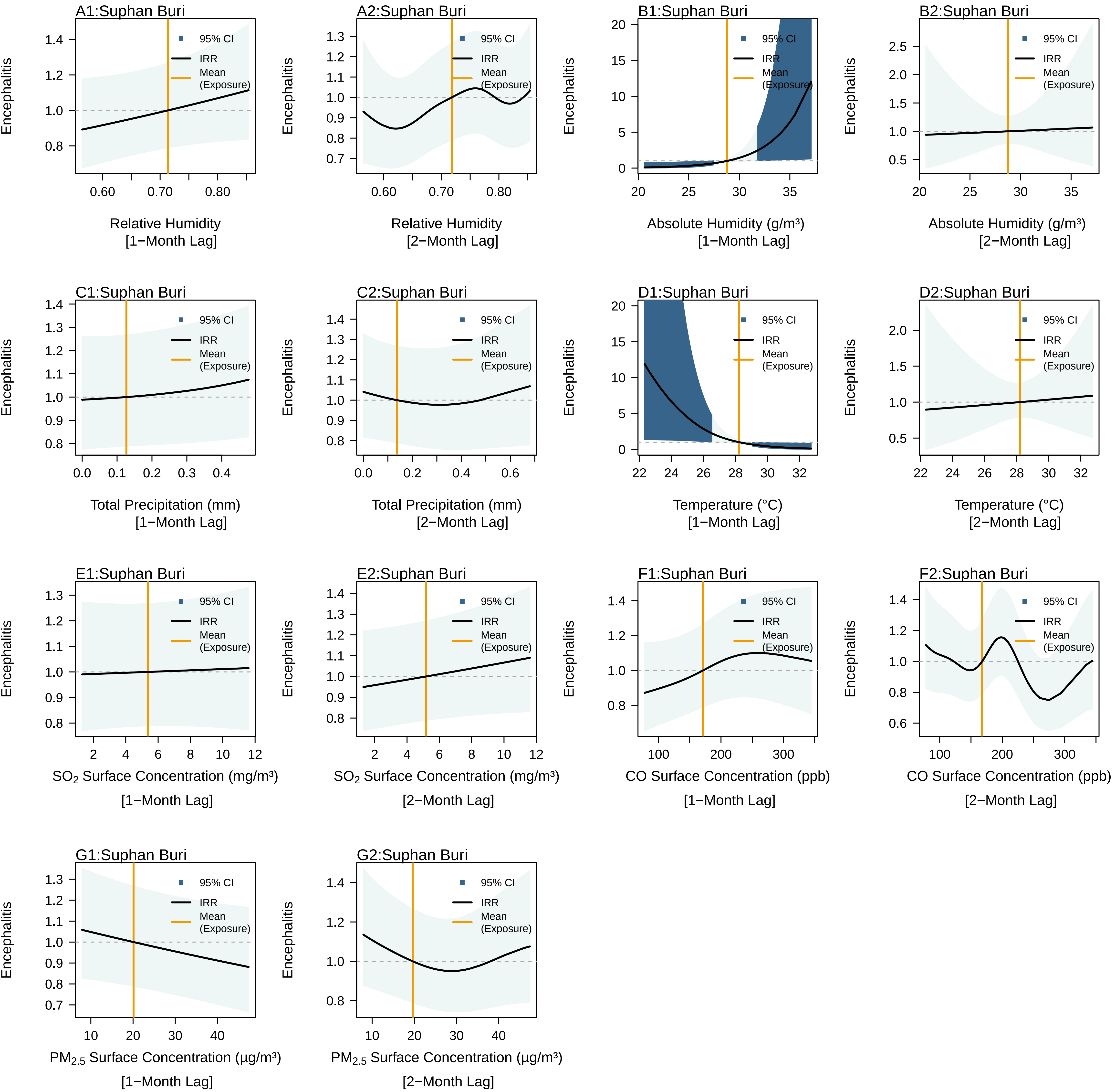
A1:Suphan Buri
Encephalitis
1.4
1.2
1.0
0.8
0.60
0.70
0.80
Relative Humidity
[1−Month Lag]
95% CI
IRR
Mean
(Exposure)
A2:Suphan Buri
Encephalitis
1.3
1.2
1.1
1.0
0.9
0.8
0.7
0.60
0.70
0.80
Relative Humidity
[2−Month Lag]
95% CI
IRR
Mean
(Exposure)
B1:Suphan Buri
Encephalitis
20
15
10
5
0
20
25
30
35
Absolute Humidity (g/m³)
[1−Month Lag]
95% CI
IRR
Mean
(Exposure)
B2:Suphan Buri
Encephalitis
2.5
2.0
1.5
1.0
0.5
20
25
30
35
Absolute Humidity (g/m³)
[2−Month Lag]
95% CI
IRR
Mean
(Exposure)
C1:Suphan Buri
Encephalitis
1.4
1.3
1.2
1.1
1.0
0.9
0.8
0.0
0.1
0.2
0.3
0.4
Total Precipitation (mm)
[1−Month Lag]
95% CI
IRR
Mean
(Exposure)
C2:Suphan Buri
Encephalitis
1.4
1.3
1.2
1.1
1.0
0.9
0.8
0.0
0.2
0.4
0.6
Total Precipitation (mm)
[2−Month Lag]
95% CI
IRR
Mean
(Exposure)
D1:Suphan Buri
Encephalitis
20
15
10
5
0
22
24
26
28
30
32
Temperature (°C)
[1−Month Lag]
95% CI
IRR
Mean
(Exposure)
D2:Suphan Buri
Encephalitis
2.0
1.5
1.0
0.5
22
24
26
28
30
32
Temperature (°C)
[2−Month Lag]
95% CI
IRR
Mean
(Exposure)
E1:Suphan Buri
Encephalitis
1.3
1.2
1.1
1.0
0.9
0.8
2
4
6
8
10
12
SO2 Surface Concentration (mg/m³)
[1−Month Lag]
95% CI
IRR
Mean
(Exposure)
E2:Suphan Buri
Encephalitis
1.4
1.3
1.2
1.1
1.0
0.9
0.8
2
4
6
8
10
12
SO2 Surface Concentration (mg/m³)
[2−Month Lag]
95% CI
IRR
Mean
(Exposure)
F1:Suphan Buri
Encephalitis
1.4
1.2
1.0
0.8
100
200
300
CO Surface Concentration (ppb)
[1−Month Lag]
95% CI
IRR
Mean
(Exposure)
F2:Suphan Buri
Encephalitis
1.4
1.2
1.0
0.8
0.6
100
200
300
CO Surface Concentration (ppb)
[2−Month Lag]
95% CI
IRR
Mean
(Exposure)
G1:Suphan Buri
Encephalitis
1.3
1.2
1.1
1.0
0.9
0.8
0.7
10
20
30
40
PM2.5 Surface Concentration (µg/m³)
[1−Month Lag]
95% CI
IRR
Mean
(Exposure)
G2:Suphan Buri
Encephalitis
1.4
1.2
1.0
0.8
10
20
30
40
PM2.5 Surface Concentration (µg/m³)
[2−Month Lag]
95% CI
IRR
Mean
(Exposure)

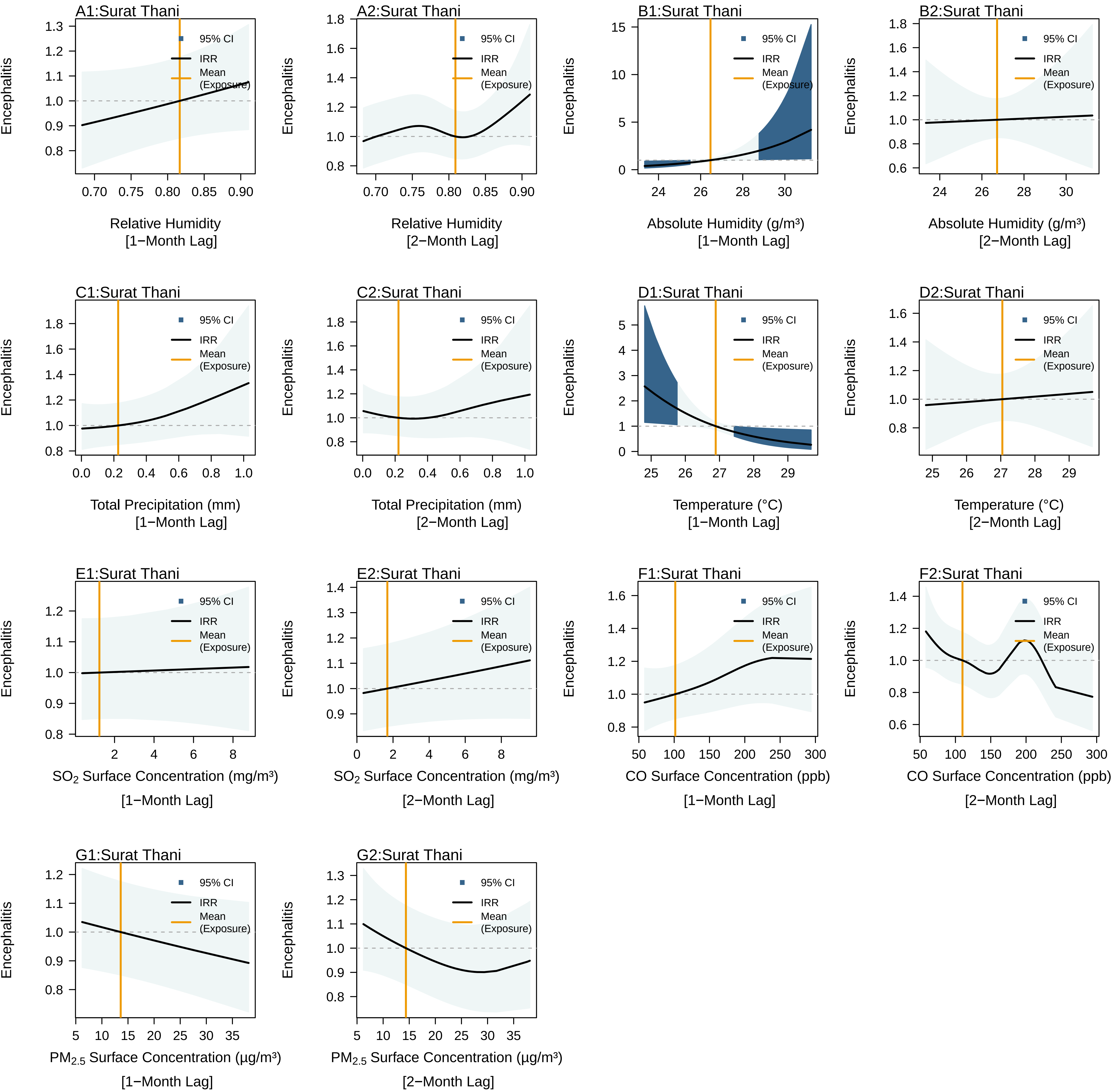
A1:Surat Thani
Encephalitis
Relative Humidity
[1−Month Lag]
95% CI
IRR
Mean
(Exposure)
A2:Surat Thani
Relative Humidity
[2−Month Lag]
B1:Surat Thani
Absolute Humidity (g/m³)
[1−Month Lag]
B2:Surat Thani
Absolute Humidity (g/m³)
[2−Month Lag]
C1:Surat Thani
Total Precipitation (mm)
[1−Month Lag]
C2:Surat Thani
Total Precipitation (mm)
[2−Month Lag]
D1:Surat Thani
Temperature (°C)
[1−Month Lag]
D2:Surat Thani
Temperature (°C)
[2−Month Lag]
E1:Surat Thani
SO_2 Surface Concentration (mg/m³)
[1−Month Lag]
E2:Surat Thani
SO_2 Surface Concentration (mg/m³)
[2−Month Lag]
F1:Surat Thani
CO Surface Concentration (ppb)
[1−Month Lag]
F2:Surat Thani
CO Surface Concentration (ppb)
[2−Month Lag]
G1:Surat Thani
$PM_{2.5}$ Surface Concentration (µg/m³)
[1−Month Lag]
G2:Surat Thani
$PM_{2.5}$ Surface Concentration (µg/m³)
[2−Month Lag]

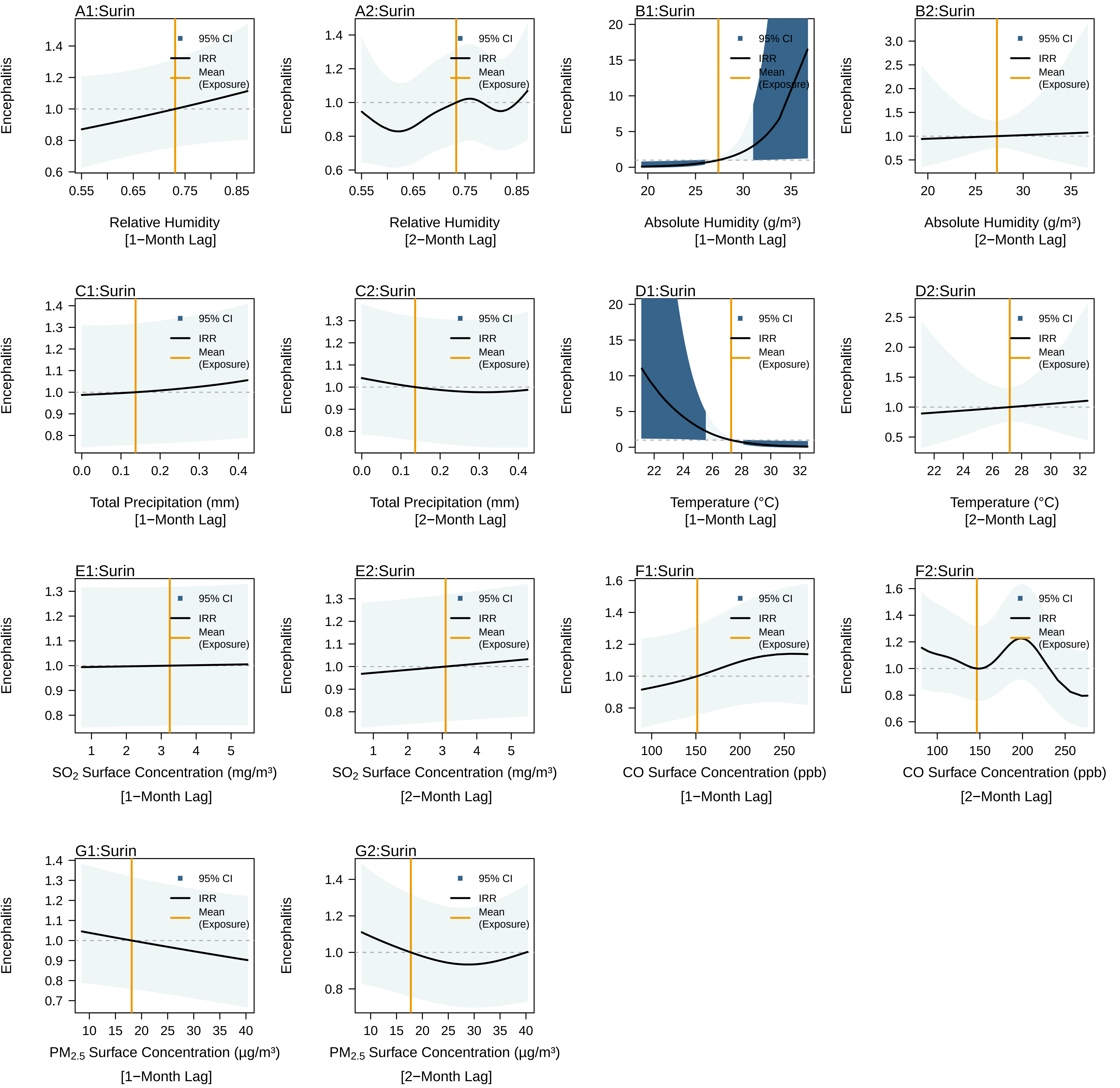
A1:Surin
Encephalitis
95% CI
IRR
Mean (Exposure)
Relative Humidity [1−Month Lag]
A2:Surin
Relative Humidity [2−Month Lag]
B1:Surin
Absolute Humidity (g/m³) [1−Month Lag]
B2:Surin
Absolute Humidity (g/m³) [2−Month Lag]
C1:Surin
Total Precipitation (mm) [1−Month Lag]
C2:Surin
Total Precipitation (mm) [2−Month Lag]
D1:Surin
Temperature (°C) [1−Month Lag]
D2:Surin
Temperature (°C) [2−Month Lag]
E1:Surin
SO2 Surface Concentration (mg/m³) [1−Month Lag]
E2:Surin
SO2 Surface Concentration (mg/m³) [2−Month Lag]
F1:Surin
CO Surface Concentration (ppb) [1−Month Lag]
F2:Surin
CO Surface Concentration (ppb) [2−Month Lag]
G1:Surin
PM2.5 Surface Concentration (µg/m³) [1−Month Lag]
G2:Surin
PM2.5 Surface Concentration (µg/m³) [2−Month Lag]

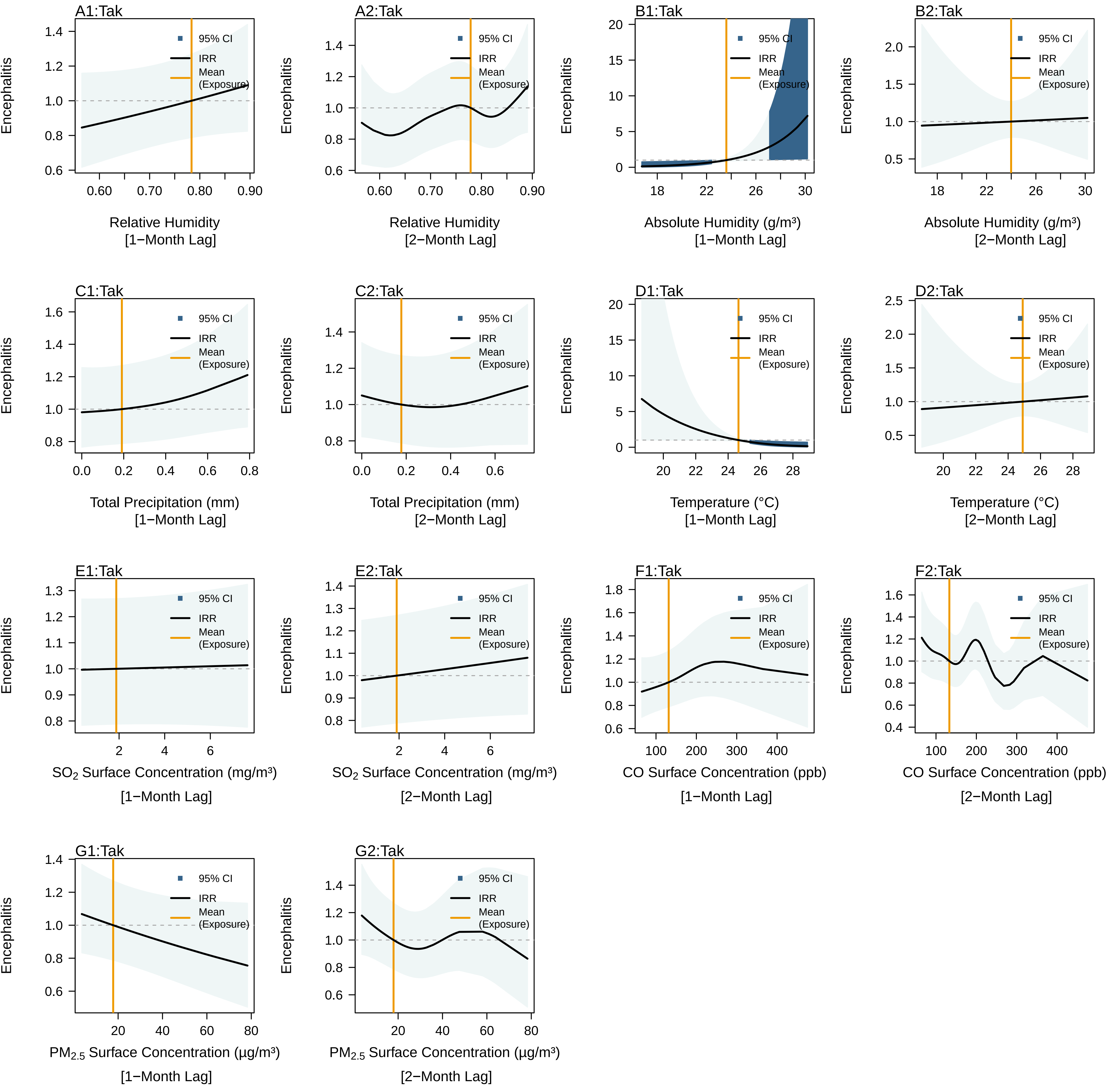
A1:Tak
Encephalitis
95% CI
IRR
Mean (Exposure)
Relative Humidity [1−Month Lag]
A2:Tak
Encephalitis
95% CI
IRR
Mean (Exposure)
Relative Humidity [2−Month Lag]
B1:Tak
Encephalitis
95% CI
IRR
Mean (Exposure)
Absolute Humidity (g/m³) [1−Month Lag]
B2:Tak
Encephalitis
95% CI
IRR
Mean (Exposure)
Absolute Humidity (g/m³) [2−Month Lag]
C1:Tak
Encephalitis
95% CI
IRR
Mean (Exposure)
Total Precipitation (mm) [1−Month Lag]
C2:Tak
Encephalitis
95% CI
IRR
Mean (Exposure)
Total Precipitation (mm) [2−Month Lag]
D1:Tak
Encephalitis
95% CI
IRR
Mean (Exposure)
Temperature (°C) [1−Month Lag]
D2:Tak
Encephalitis
95% CI
IRR
Mean (Exposure)
Temperature (°C) [2−Month Lag]
E1:Tak
Encephalitis
95% CI
IRR
Mean (Exposure)
SO_2 Surface Concentration (mg/m³) [1−Month Lag]
E2:Tak
Encephalitis
95% CI
IRR
Mean (Exposure)
SO_2 Surface Concentration (mg/m³) [2−Month Lag]
F1:Tak
Encephalitis
95% CI
IRR
Mean (Exposure)
CO Surface Concentration (ppb) [1−Month Lag]
F2:Tak
Encephalitis
95% CI
IRR
Mean (Exposure)
CO Surface Concentration (ppb) [2−Month Lag]
G1:Tak
Encephalitis
95% CI
IRR
Mean (Exposure)
$PM_{2.5}$ Surface Concentration (µg/m³) [1−Month Lag]
G2:Tak
Encephalitis
95% CI
IRR
Mean (Exposure)
$PM_{2.5}$ Surface Concentration (µg/m³) [2−Month Lag]

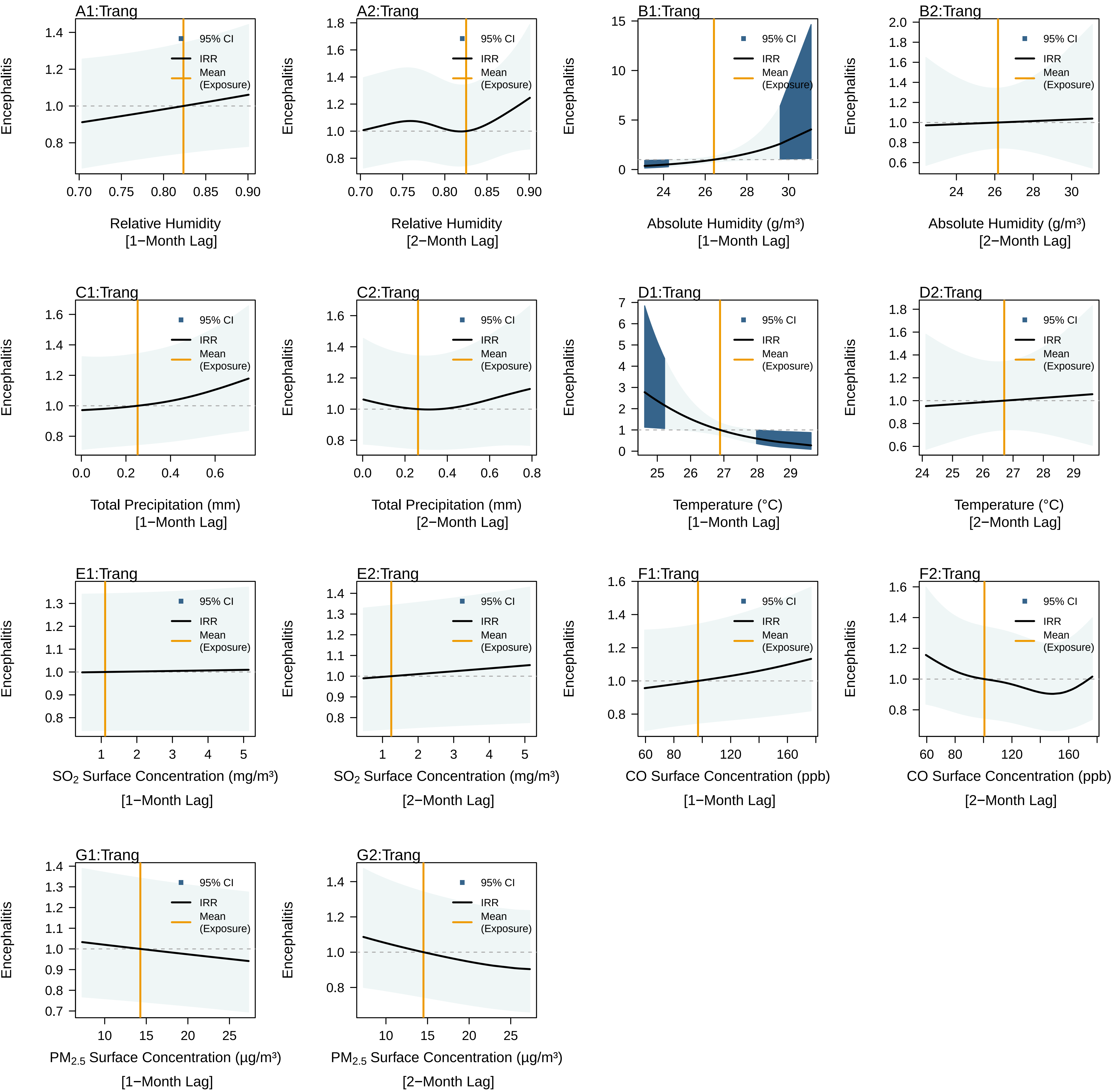

A1:Trang
Encephalitis
95% CI
IRR
Mean
(Exposure)
Relative Humidity
[1−Month Lag]
A2:Trang
Relative Humidity
[2−Month Lag]
B1:Trang
Absolute Humidity (g/m³)
[1−Month Lag]
B2:Trang
Absolute Humidity (g/m³)
[2−Month Lag]
C1:Trang
Total Precipitation (mm)
[1−Month Lag]
C2:Trang
Total Precipitation (mm)
[2−Month Lag]
D1:Trang
Temperature (°C)
[1−Month Lag]
D2:Trang
Temperature (°C)
[2−Month Lag]
E1:Trang
SO_2 Surface Concentration (mg/m³)
[1−Month Lag]
E2:Trang
SO_2 Surface Concentration (mg/m³)
[2−Month Lag]
F1:Trang
CO Surface Concentration (ppb)
[1−Month Lag]
F2:Trang
CO Surface Concentration (ppb)
[2−Month Lag]
G1:Trang
$PM_{2.5}$ Surface Concentration (µg/m³)
[1−Month Lag]
G2:Trang
$PM_{2.5}$ Surface Concentration (µg/m³)
[2−Month Lag]

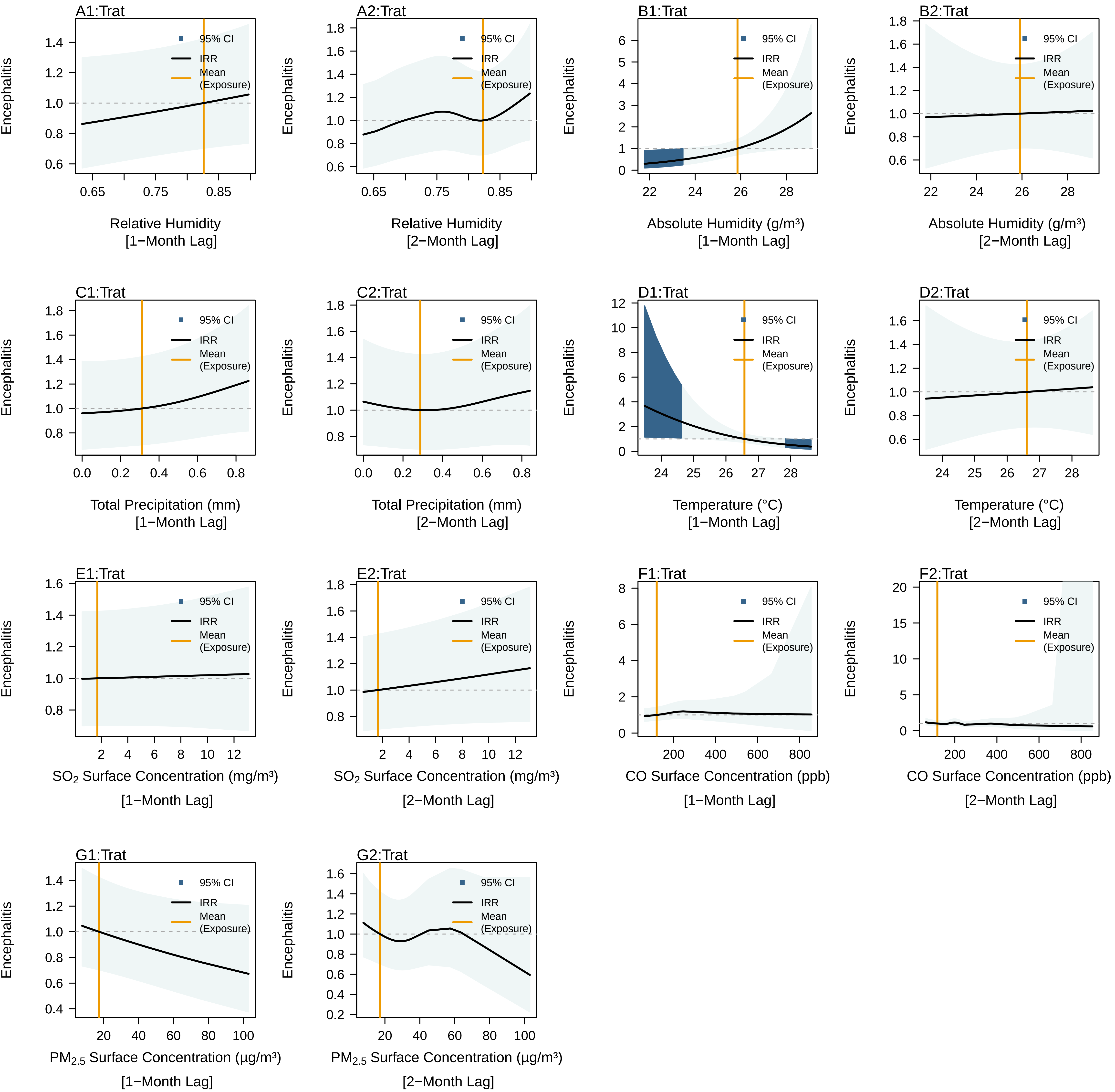
A1:Trat
A2:Trat
B1:Trat
B2:Trat
C1:Trat
C2:Trat
D1:Trat
D2:Trat
E1:Trat
E2:Trat
F1:Trat
F2:Trat
G1:Trat
G2:Trat
Encephalitis
95% CI
IRR
Mean
(Exposure)
Relative Humidity
[1−Month Lag]
Relative Humidity
[2−Month Lag]
Absolute Humidity (g/m³)
[1−Month Lag]
Absolute Humidity (g/m³)
[2−Month Lag]
Total Precipitation (mm)
[1−Month Lag]
Total Precipitation (mm)
[2−Month Lag]
Temperature (°C)
[1−Month Lag]
Temperature (°C)
[2−Month Lag]
SO_2 Surface Concentration (mg/m³)
[1−Month Lag]
SO_2 Surface Concentration (mg/m³)
[2−Month Lag]
CO Surface Concentration (ppb)
[1−Month Lag]
CO Surface Concentration (ppb)
[2−Month Lag]
$PM_{2.5}$ Surface Concentration (µg/m³)
[1−Month Lag]
$PM_{2.5}$ Surface Concentration (µg/m³)
[2−Month Lag]

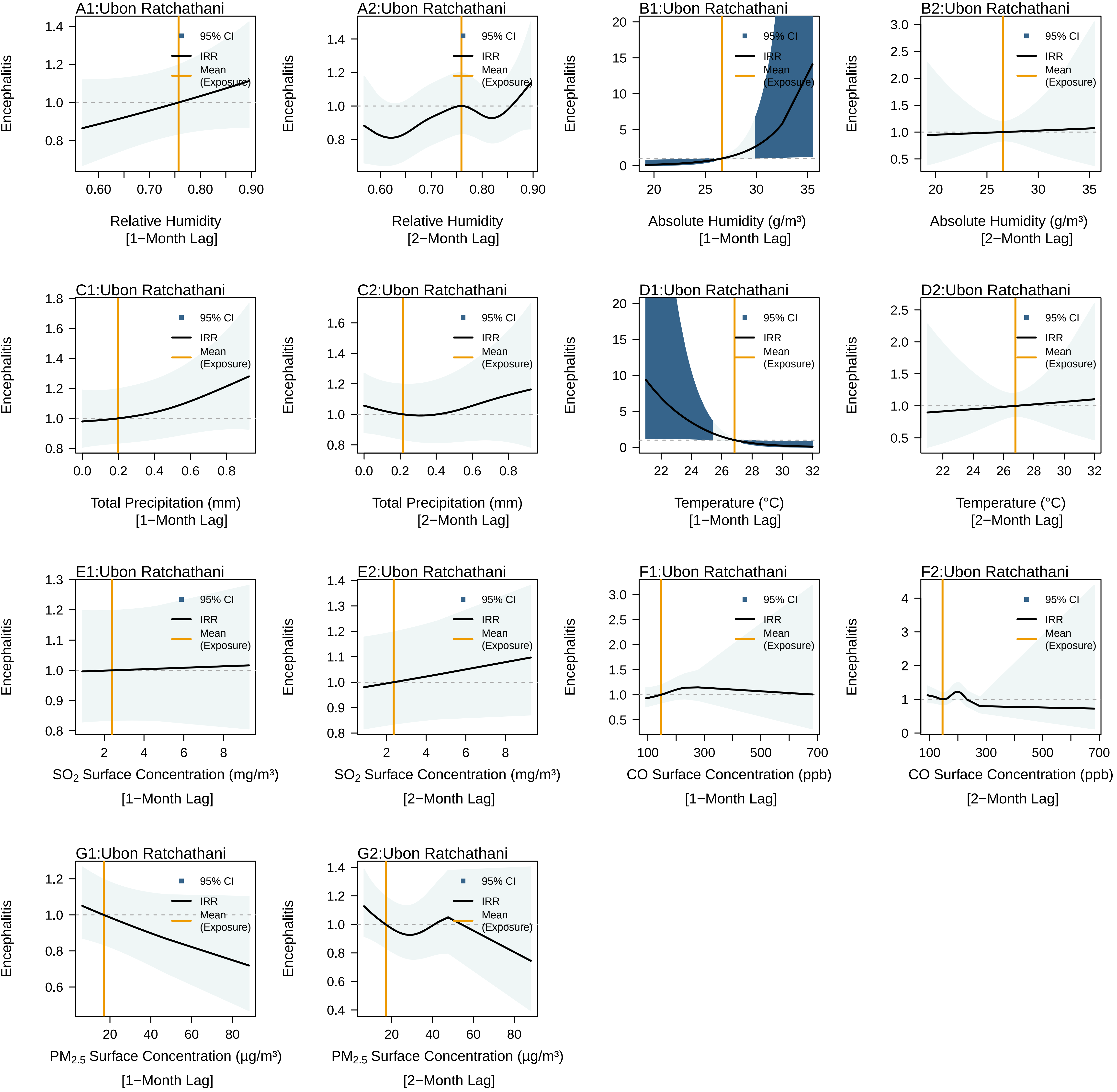
A1:Ubon Ratchathani
Encephalitis
95% CI
IRR
Mean (Exposure)
Relative Humidity [1−Month Lag]
A2:Ubon Ratchathani
Relative Humidity [2−Month Lag]
B1:Ubon Ratchathani
Absolute Humidity (g/m³) [1−Month Lag]
B2:Ubon Ratchathani
Absolute Humidity (g/m³) [2−Month Lag]
C1:Ubon Ratchathani
Total Precipitation (mm) [1−Month Lag]
C2:Ubon Ratchathani
Total Precipitation (mm) [2−Month Lag]
D1:Ubon Ratchathani
Temperature (°C) [1−Month Lag]
D2:Ubon Ratchathani
Temperature (°C) [2−Month Lag]
E1:Ubon Ratchathani
SO2 Surface Concentration (mg/m³) [1−Month Lag]
E2:Ubon Ratchathani
SO2 Surface Concentration (mg/m³) [2−Month Lag]
F1:Ubon Ratchathani
CO Surface Concentration (ppb) [1−Month Lag]
F2:Ubon Ratchathani
CO Surface Concentration (ppb) [2−Month Lag]
G1:Ubon Ratchathani
PM2.5 Surface Concentration (µg/m³) [1−Month Lag]
G2:Ubon Ratchathani
PM2.5 Surface Concentration (µg/m³) [2−Month Lag]

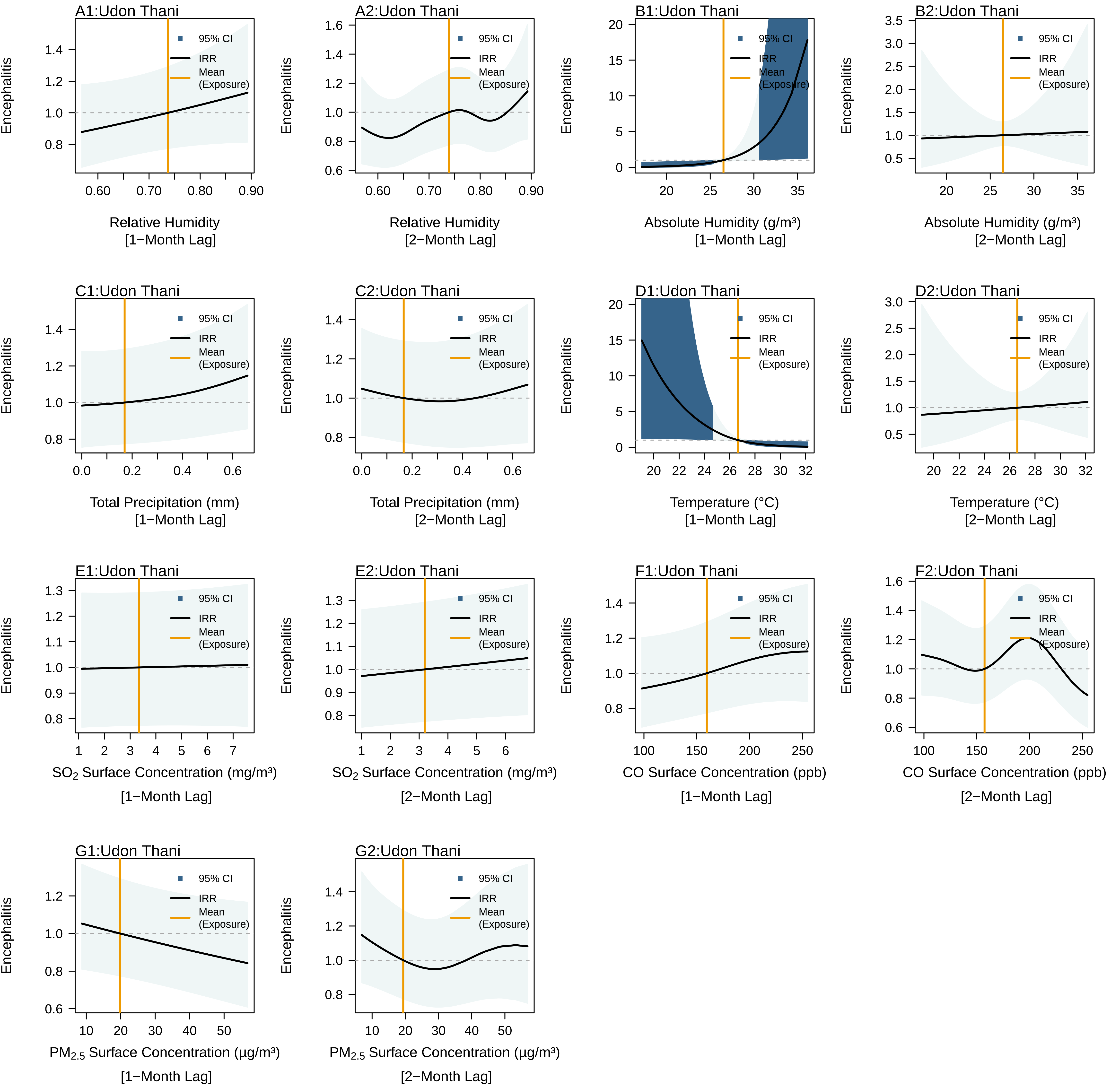
A1:Udon Thani
Relative Humidity [1−Month Lag]
A2:Udon Thani
Relative Humidity [2−Month Lag]
B1:Udon Thani
Absolute Humidity (g/m³) [1−Month Lag]
B2:Udon Thani
Absolute Humidity (g/m³) [2−Month Lag]
C1:Udon Thani
Total Precipitation (mm) [1−Month Lag]
C2:Udon Thani
Total Precipitation (mm) [2−Month Lag]
D1:Udon Thani
Temperature (°C) [1−Month Lag]
D2:Udon Thani
Temperature (°C) [2−Month Lag]
E1:Udon Thani
SO_2 Surface Concentration (mg/m³) [1−Month Lag]
E2:Udon Thani
SO_2 Surface Concentration (mg/m³) [2−Month Lag]
F1:Udon Thani
CO Surface Concentration (ppb) [1−Month Lag]
F2:Udon Thani
CO Surface Concentration (ppb) [2−Month Lag]
G1:Udon Thani
$PM_{2.5}$ Surface Concentration (µg/m³) [1−Month Lag]
G2:Udon Thani
$PM_{2.5}$ Surface Concentration (µg/m³) [2−Month Lag]
Encephalitis
95% CI
IRR
Mean (Exposure)

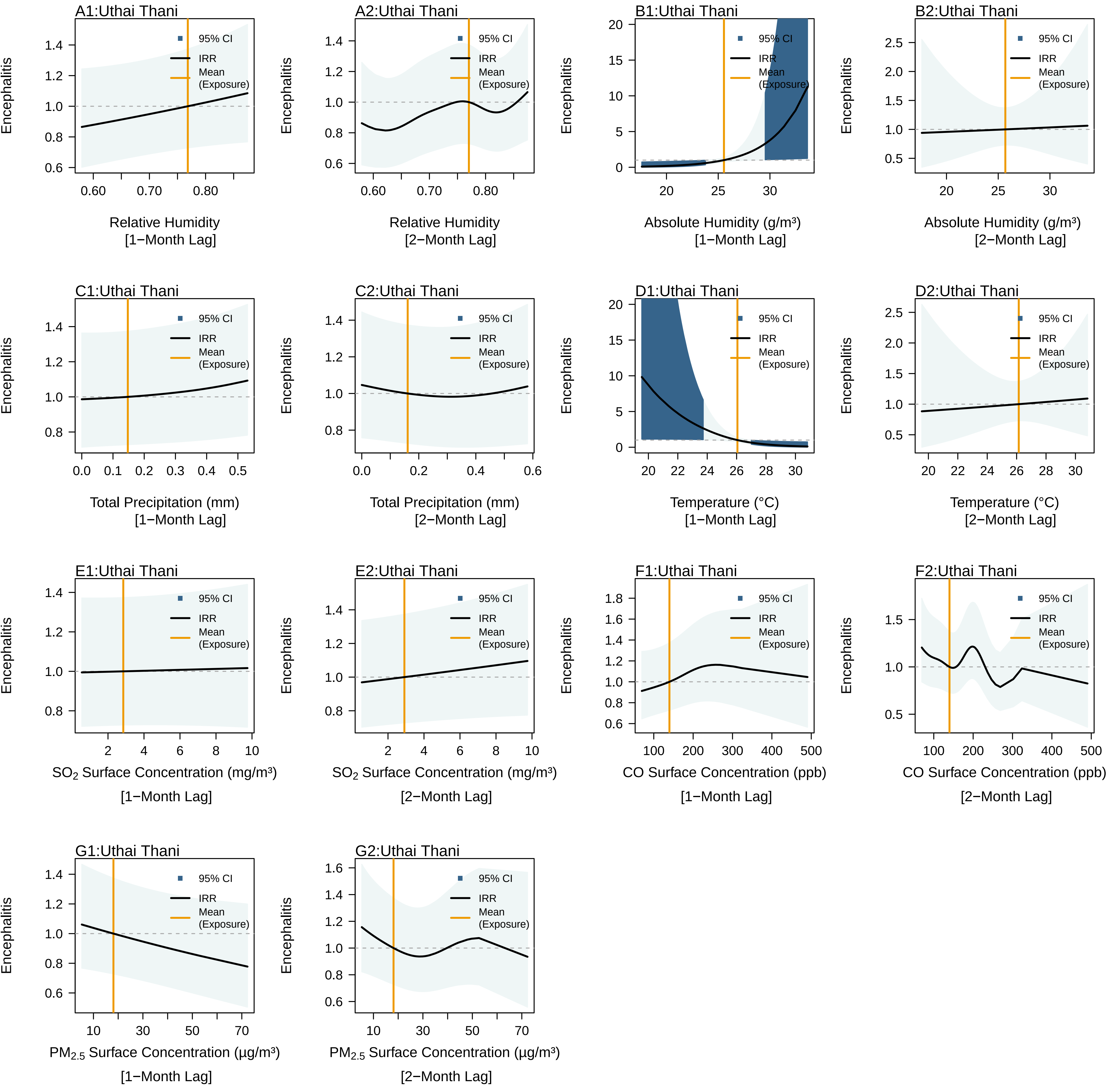
A1:Uthai Thani
Encephalitis
95% CI
IRR
Mean
(Exposure)
Relative Humidity
[1−Month Lag]
A2:Uthai Thani
Relative Humidity
[2−Month Lag]
B1:Uthai Thani
Absolute Humidity (g/m³)
[1−Month Lag]
B2:Uthai Thani
Absolute Humidity (g/m³)
[2−Month Lag]
C1:Uthai Thani
Total Precipitation (mm)
[1−Month Lag]
C2:Uthai Thani
Total Precipitation (mm)
[2−Month Lag]
D1:Uthai Thani
Temperature (°C)
[1−Month Lag]
D2:Uthai Thani
Temperature (°C)
[2−Month Lag]
E1:Uthai Thani
SO_2 Surface Concentration (mg/m³)
[1−Month Lag]
E2:Uthai Thani
SO_2 Surface Concentration (mg/m³)
[2−Month Lag]
F1:Uthai Thani
CO Surface Concentration (ppb)
[1−Month Lag]
F2:Uthai Thani
CO Surface Concentration (ppb)
[2−Month Lag]
G1:Uthai Thani
$PM_{2.5}$ Surface Concentration (µg/m³)
[1−Month Lag]
G2:Uthai Thani
$PM_{2.5}$ Surface Concentration (µg/m³)
[2−Month Lag]

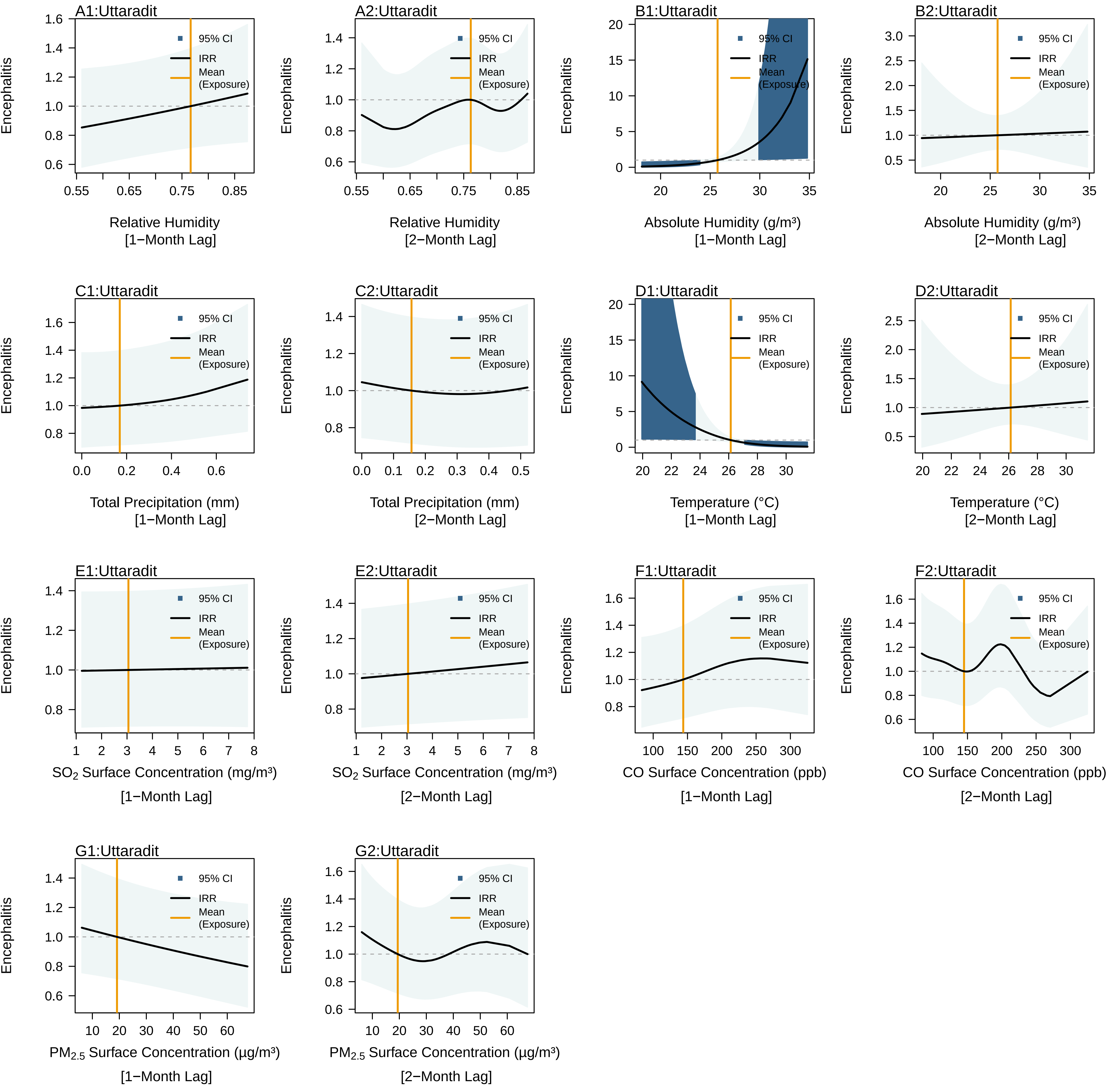
A1:Uttaradit
Encephalitis
95% CI
IRR
Mean (Exposure)
Relative Humidity [1−Month Lag]
A2:Uttaradit
Encephalitis
95% CI
IRR
Mean (Exposure)
Relative Humidity [2−Month Lag]
B1:Uttaradit
Encephalitis
95% CI
IRR
Mean (Exposure)
Absolute Humidity (g/m³) [1−Month Lag]
B2:Uttaradit
Encephalitis
95% CI
IRR
Mean (Exposure)
Absolute Humidity (g/m³) [2−Month Lag]
C1:Uttaradit
Encephalitis
95% CI
IRR
Mean (Exposure)
Total Precipitation (mm) [1−Month Lag]
C2:Uttaradit
Encephalitis
95% CI
IRR
Mean (Exposure)
Total Precipitation (mm) [2−Month Lag]
D1:Uttaradit
Encephalitis
95% CI
IRR
Mean (Exposure)
Temperature (°C) [1−Month Lag]
D2:Uttaradit
Encephalitis
95% CI
IRR
Mean (Exposure)
Temperature (°C) [2−Month Lag]
E1:Uttaradit
Encephalitis
95% CI
IRR
Mean (Exposure)
SO_2 Surface Concentration (mg/m³) [1−Month Lag]
E2:Uttaradit
Encephalitis
95% CI
IRR
Mean (Exposure)
SO_2 Surface Concentration (mg/m³) [2−Month Lag]
F1:Uttaradit
Encephalitis
95% CI
IRR
Mean (Exposure)
CO Surface Concentration (ppb) [1−Month Lag]
F2:Uttaradit
Encephalitis
95% CI
IRR
Mean (Exposure)
CO Surface Concentration (ppb) [2−Month Lag]
G1:Uttaradit
Encephalitis
95% CI
IRR
Mean (Exposure)
$PM_{2.5}$ Surface Concentration (μg/m³) [1−Month Lag]
G2:Uttaradit
Encephalitis
95% CI
IRR
Mean (Exposure)
$PM_{2.5}$ Surface Concentration (μg/m³) [2−Month Lag]

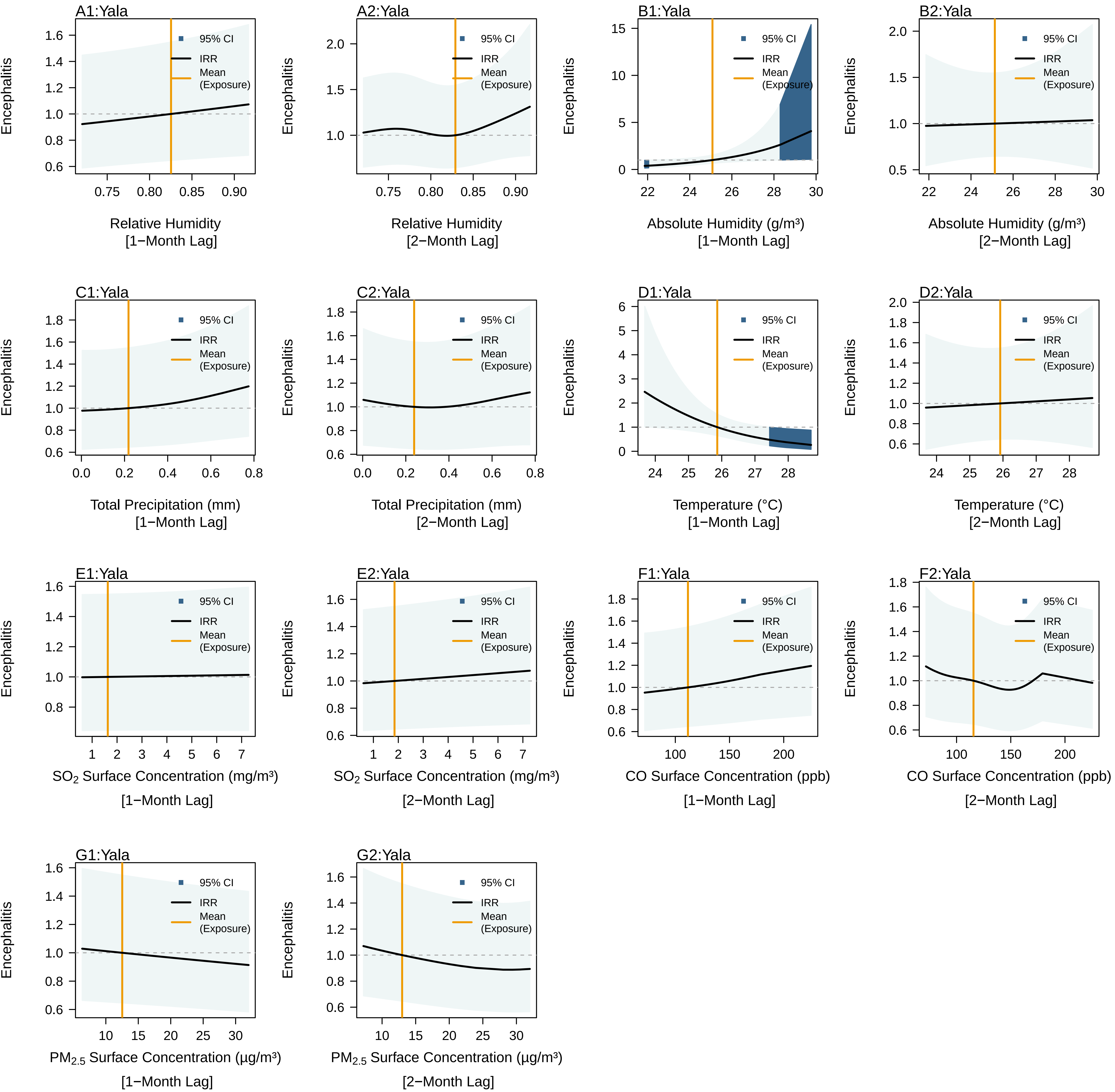
A1:Yala
95% CI
IRR
Mean (Exposure)
Encephalitis
Relative Humidity [1−Month Lag]
A2:Yala
95% CI
IRR
Mean (Exposure)
Encephalitis
Relative Humidity [2−Month Lag]
B1:Yala
95% CI
IRR
Mean (Exposure)
Encephalitis
Absolute Humidity (g/m³) [1−Month Lag]
B2:Yala
95% CI
IRR
Mean (Exposure)
Encephalitis
Absolute Humidity (g/m³) [2−Month Lag]
C1:Yala
95% CI
IRR
Mean (Exposure)
Encephalitis
Total Precipitation (mm) [1−Month Lag]
C2:Yala
95% CI
IRR
Mean (Exposure)
Encephalitis
Total Precipitation (mm) [2−Month Lag]
D1:Yala
95% CI
IRR
Mean (Exposure)
Encephalitis
Temperature (°C) [1−Month Lag]
D2:Yala
95% CI
IRR
Mean (Exposure)
Encephalitis
Temperature (°C) [2−Month Lag]
E1:Yala
95% CI
IRR
Mean (Exposure)
Encephalitis
SO_2 Surface Concentration (mg/m³) [1−Month Lag]
E2:Yala
95% CI
IRR
Mean (Exposure)
Encephalitis
SO_2 Surface Concentration (mg/m³) [2−Month Lag]
F1:Yala
95% CI
IRR
Mean (Exposure)
Encephalitis
CO Surface Concentration (ppb) [1−Month Lag]
F2:Yala
95% CI
IRR
Mean (Exposure)
Encephalitis
CO Surface Concentration (ppb) [2−Month Lag]
G1:Yala
95% CI
IRR
Mean (Exposure)
Encephalitis
$PM_{2.5}$ Surface Concentration (µg/m³) [1−Month Lag]
G2:Yala
95% CI
IRR
Mean (Exposure)
Encephalitis
$PM_{2.5}$ Surface Concentration (µg/m³) [2−Month Lag]

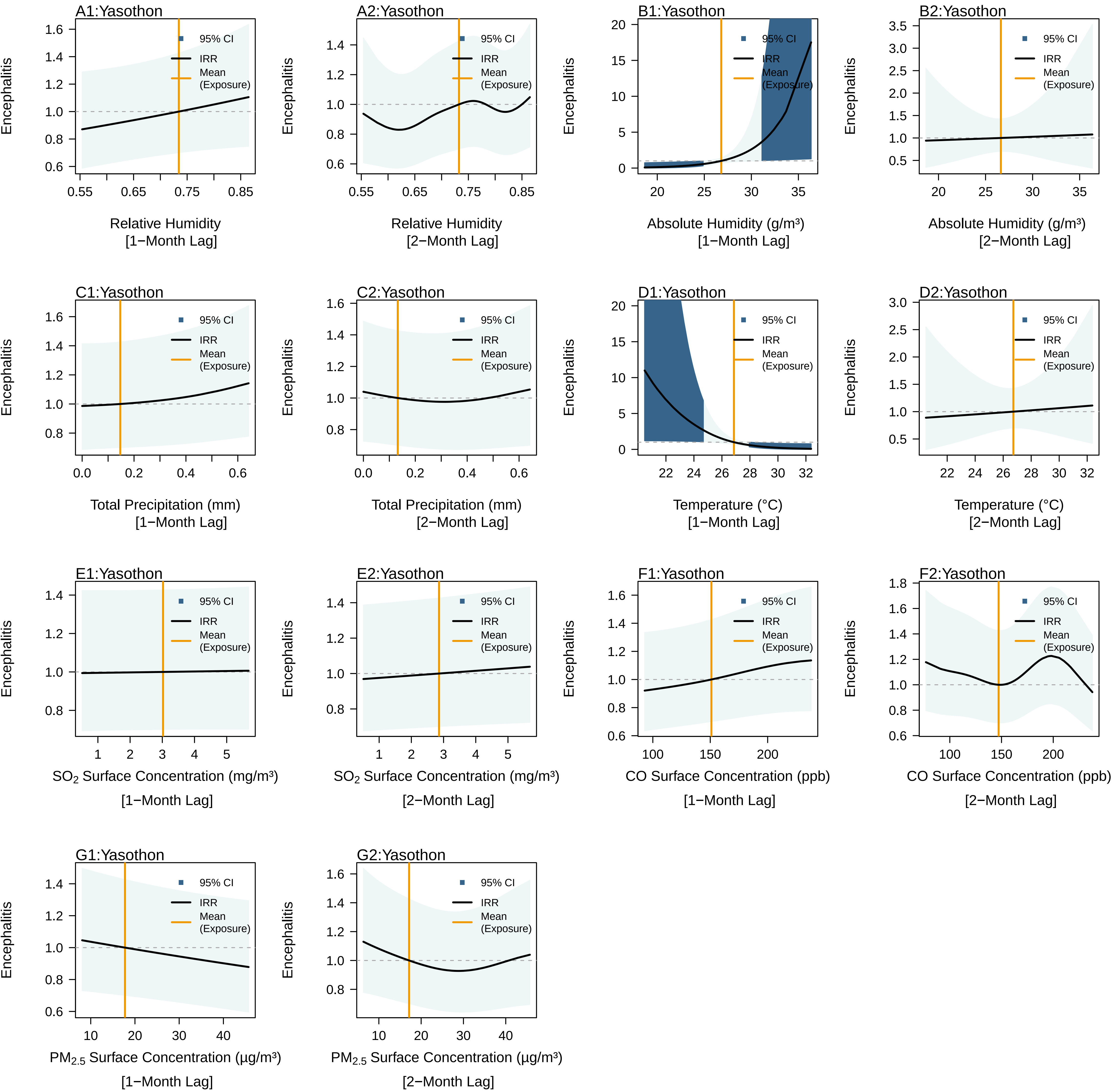
A1:Yasothon
Encephalitis
95% CI
IRR
Mean (Exposure)
Relative Humidity [1−Month Lag]
A2:Yasothon
Encephalitis
Relative Humidity [2−Month Lag]
B1:Yasothon
Encephalitis
Absolute Humidity (g/m³) [1−Month Lag]
B2:Yasothon
Encephalitis
Absolute Humidity (g/m³) [2−Month Lag]
C1:Yasothon
Encephalitis
Total Precipitation (mm) [1−Month Lag]
C2:Yasothon
Encephalitis
Total Precipitation (mm) [2−Month Lag]
D1:Yasothon
Encephalitis
Temperature (°C) [1−Month Lag]
D2:Yasothon
Encephalitis
Temperature (°C) [2−Month Lag]
E1:Yasothon
Encephalitis
SO2 Surface Concentration (mg/m³) [1−Month Lag]
E2:Yasothon
Encephalitis
SO2 Surface Concentration (mg/m³) [2−Month Lag]
F1:Yasothon
Encephalitis
CO Surface Concentration (ppb) [1−Month Lag]
F2:Yasothon
Encephalitis
CO Surface Concentration (ppb) [2−Month Lag]
G1:Yasothon
Encephalitis
PM2.5 Surface Concentration (µg/m³) [1−Month Lag]
G2:Yasothon
Encephalitis
PM2.5 Surface Concentration (µg/m³) [2−Month Lag]

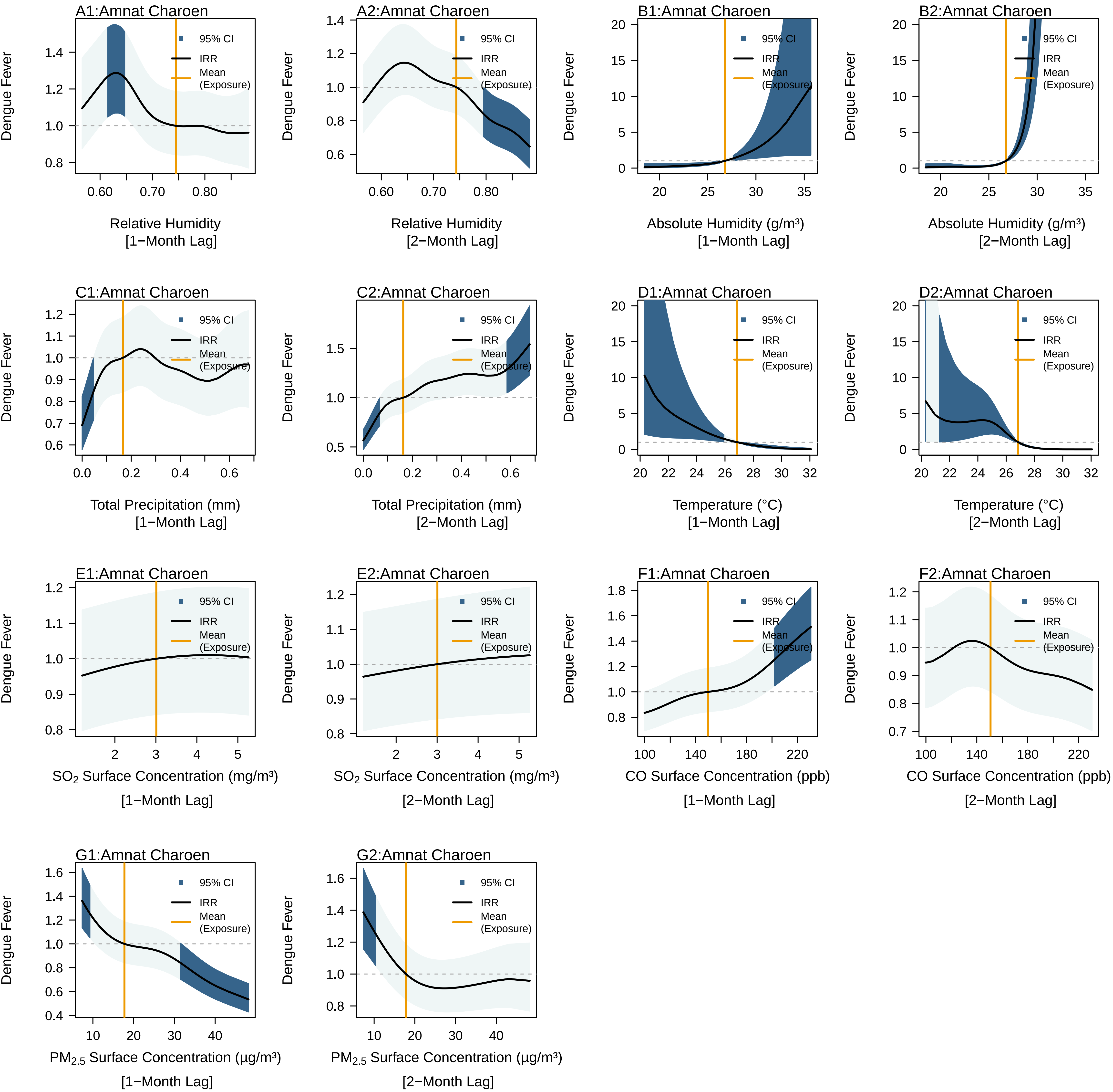
A1:Amnat Charoen
Dengue Fever
Relative Humidity [1−Month Lag]
A2:Amnat Charoen
Dengue Fever
Relative Humidity [2−Month Lag]
B1:Amnat Charoen
Dengue Fever
Absolute Humidity (g/m³) [1−Month Lag]
B2:Amnat Charoen
Dengue Fever
Absolute Humidity (g/m³) [2−Month Lag]
C1:Amnat Charoen
Dengue Fever
Total Precipitation (mm) [1−Month Lag]
C2:Amnat Charoen
Dengue Fever
Total Precipitation (mm) [2−Month Lag]
D1:Amnat Charoen
Dengue Fever
Temperature (°C) [1−Month Lag]
D2:Amnat Charoen
Dengue Fever
Temperature (°C) [2−Month Lag]
E1:Amnat Charoen
Dengue Fever
SO_2 Surface Concentration (mg/m³) [1−Month Lag]
E2:Amnat Charoen
Dengue Fever
SO_2 Surface Concentration (mg/m³) [2−Month Lag]
F1:Amnat Charoen
Dengue Fever
CO Surface Concentration (ppb) [1−Month Lag]
F2:Amnat Charoen
Dengue Fever
CO Surface Concentration (ppb) [2−Month Lag]
G1:Amnat Charoen
Dengue Fever
$PM_{2.5}$ Surface Concentration (μg/m³) [1−Month Lag]
G2:Amnat Charoen
Dengue Fever
$PM_{2.5}$ Surface Concentration (μg/m³) [2−Month Lag]
95% CI
IRR
Mean (Exposure)

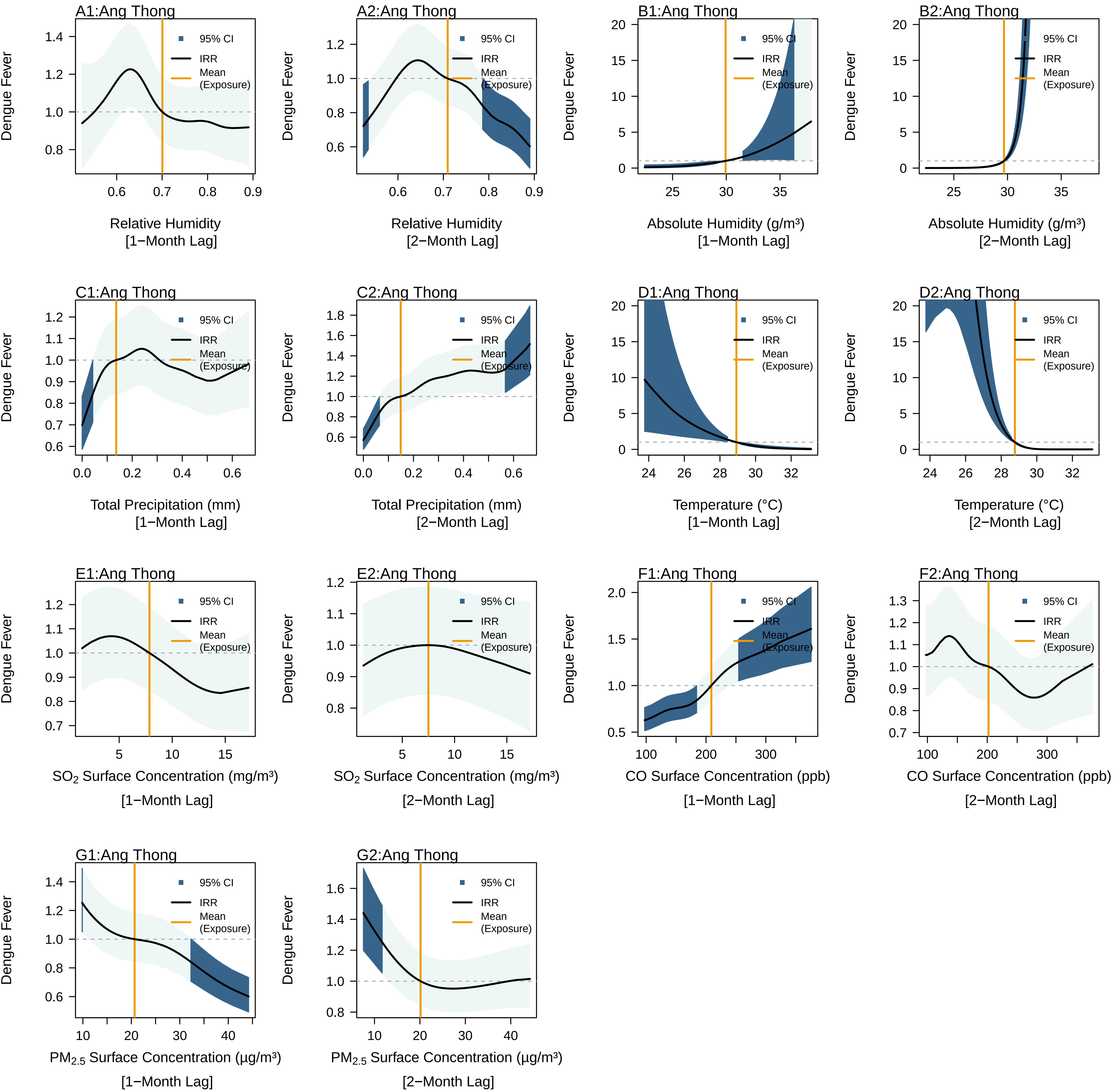
A1:Ang Thong
A2:Ang Thong
B1:Ang Thong
B2:Ang Thong
C1:Ang Thong
C2:Ang Thong
D1:Ang Thong
D2:Ang Thong
E1:Ang Thong
E2:Ang Thong
F1:Ang Thong
F2:Ang Thong
G1:Ang Thong
G2:Ang Thong
95% CI
IRR
Mean (Exposure)
Dengue Fever
Relative Humidity [1−Month Lag]
Relative Humidity [2−Month Lag]
Absolute Humidity (g/m³) [1−Month Lag]
Absolute Humidity (g/m³) [2−Month Lag]
Total Precipitation (mm) [1−Month Lag]
Total Precipitation (mm) [2−Month Lag]
Temperature (°C) [1−Month Lag]
Temperature (°C) [2−Month Lag]
SO_2 Surface Concentration (mg/m³) [1−Month Lag]
SO_2 Surface Concentration (mg/m³) [2−Month Lag]
CO Surface Concentration (ppb) [1−Month Lag]
CO Surface Concentration (ppb) [2−Month Lag]
$PM_{2.5}$ Surface Concentration (µg/m³) [1−Month Lag]
$PM_{2.5}$ Surface Concentration (µg/m³) [2−Month Lag]

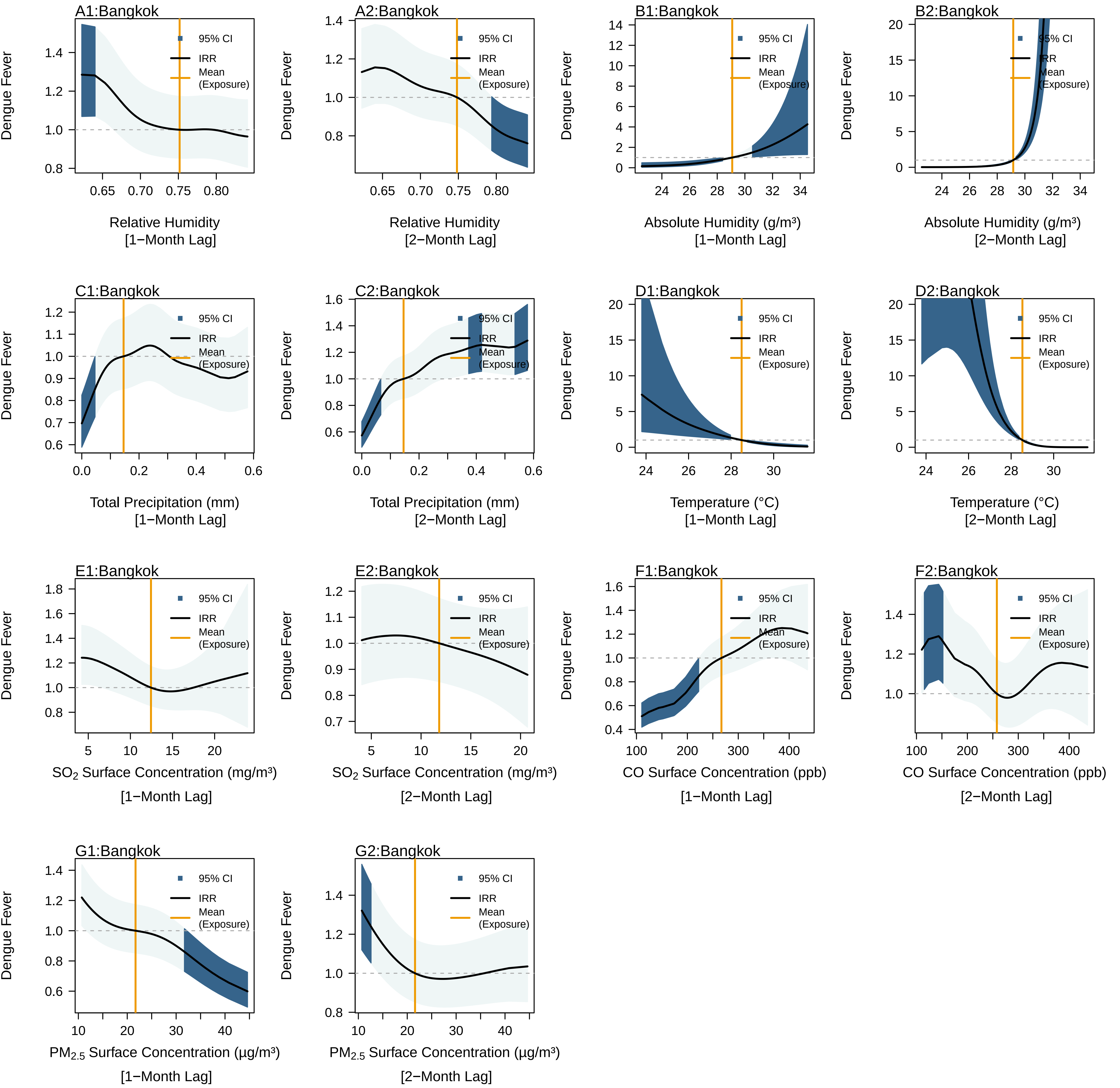

A1:Bangkok
A2:Bangkok
B1:Bangkok
B2:Bangkok
C1:Bangkok
C2:Bangkok
D1:Bangkok
D2:Bangkok
E1:Bangkok
E2:Bangkok
F1:Bangkok
F2:Bangkok
G1:Bangkok
G2:Bangkok
Dengue Fever
95% CI
IRR
Mean (Exposure)
Relative Humidity [1−Month Lag]
Relative Humidity [2−Month Lag]
Absolute Humidity (g/m³) [1−Month Lag]
Absolute Humidity (g/m³) [2−Month Lag]
Total Precipitation (mm) [1−Month Lag]
Total Precipitation (mm) [2−Month Lag]
Temperature (°C) [1−Month Lag]
Temperature (°C) [2−Month Lag]
SO_2 Surface Concentration (mg/m³) [1−Month Lag]
SO_2 Surface Concentration (mg/m³) [2−Month Lag]
CO Surface Concentration (ppb) [1−Month Lag]
CO Surface Concentration (ppb) [2−Month Lag]
$PM_{2.5}$ Surface Concentration (μg/m³) [1−Month Lag]
$PM_{2.5}$ Surface Concentration (μg/m³) [2−Month Lag]

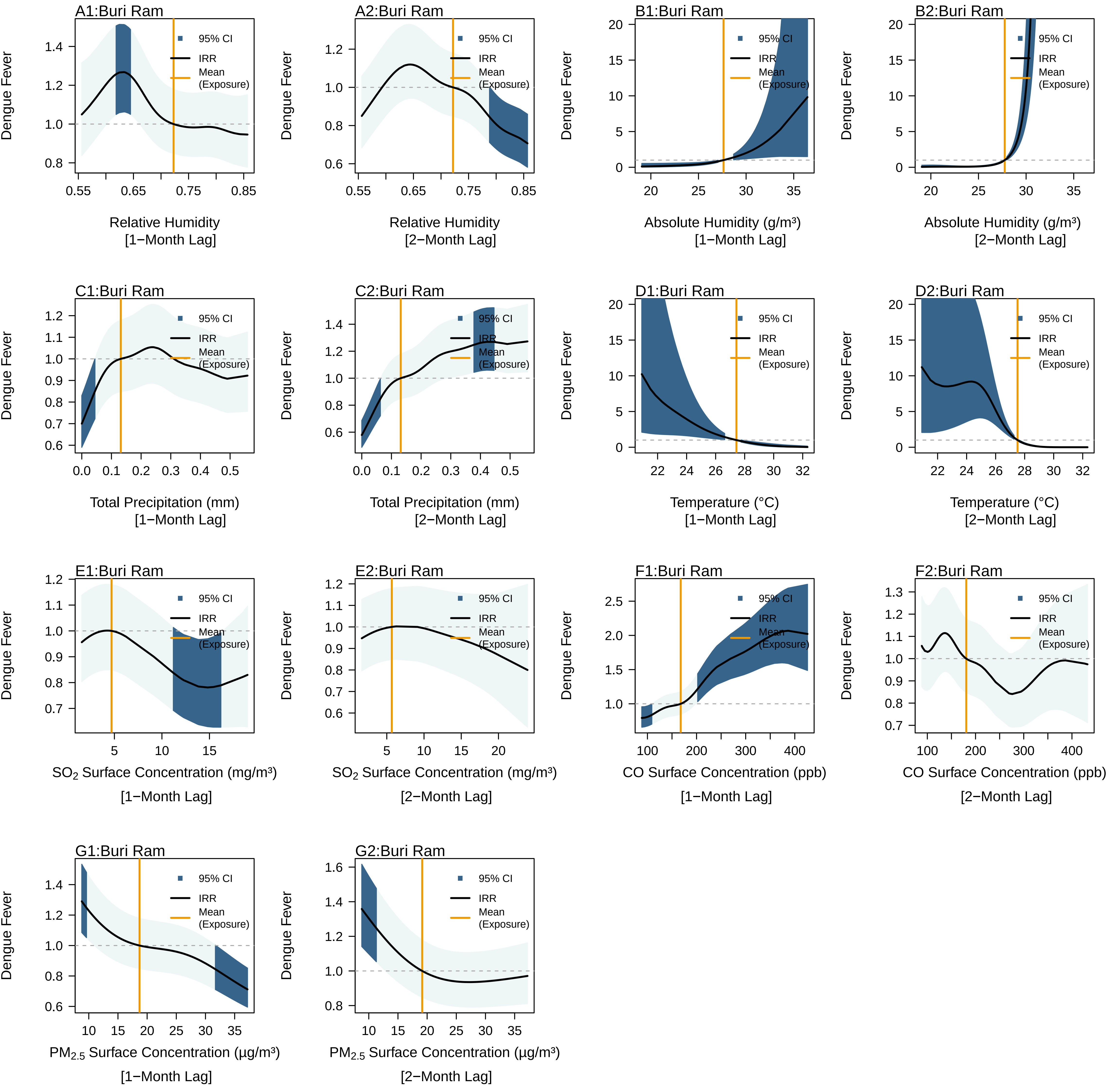
A1:Buri Ram
Dengue Fever
95% CI
IRR
Mean (Exposure)
Relative Humidity [1−Month Lag]
A2:Buri Ram
Relative Humidity [2−Month Lag]
B1:Buri Ram
Absolute Humidity (g/m³) [1−Month Lag]
B2:Buri Ram
Absolute Humidity (g/m³) [2−Month Lag]
C1:Buri Ram
Total Precipitation (mm) [1−Month Lag]
C2:Buri Ram
Total Precipitation (mm) [2−Month Lag]
D1:Buri Ram
Temperature (°C) [1−Month Lag]
D2:Buri Ram
Temperature (°C) [2−Month Lag]
E1:Buri Ram
SO2 Surface Concentration (mg/m³) [1−Month Lag]
E2:Buri Ram
SO2 Surface Concentration (mg/m³) [2−Month Lag]
F1:Buri Ram
CO Surface Concentration (ppb) [1−Month Lag]
F2:Buri Ram
CO Surface Concentration (ppb) [2−Month Lag]
G1:Buri Ram
PM2.5 Surface Concentration (µg/m³) [1−Month Lag]
G2:Buri Ram
PM2.5 Surface Concentration (µg/m³) [2−Month Lag]

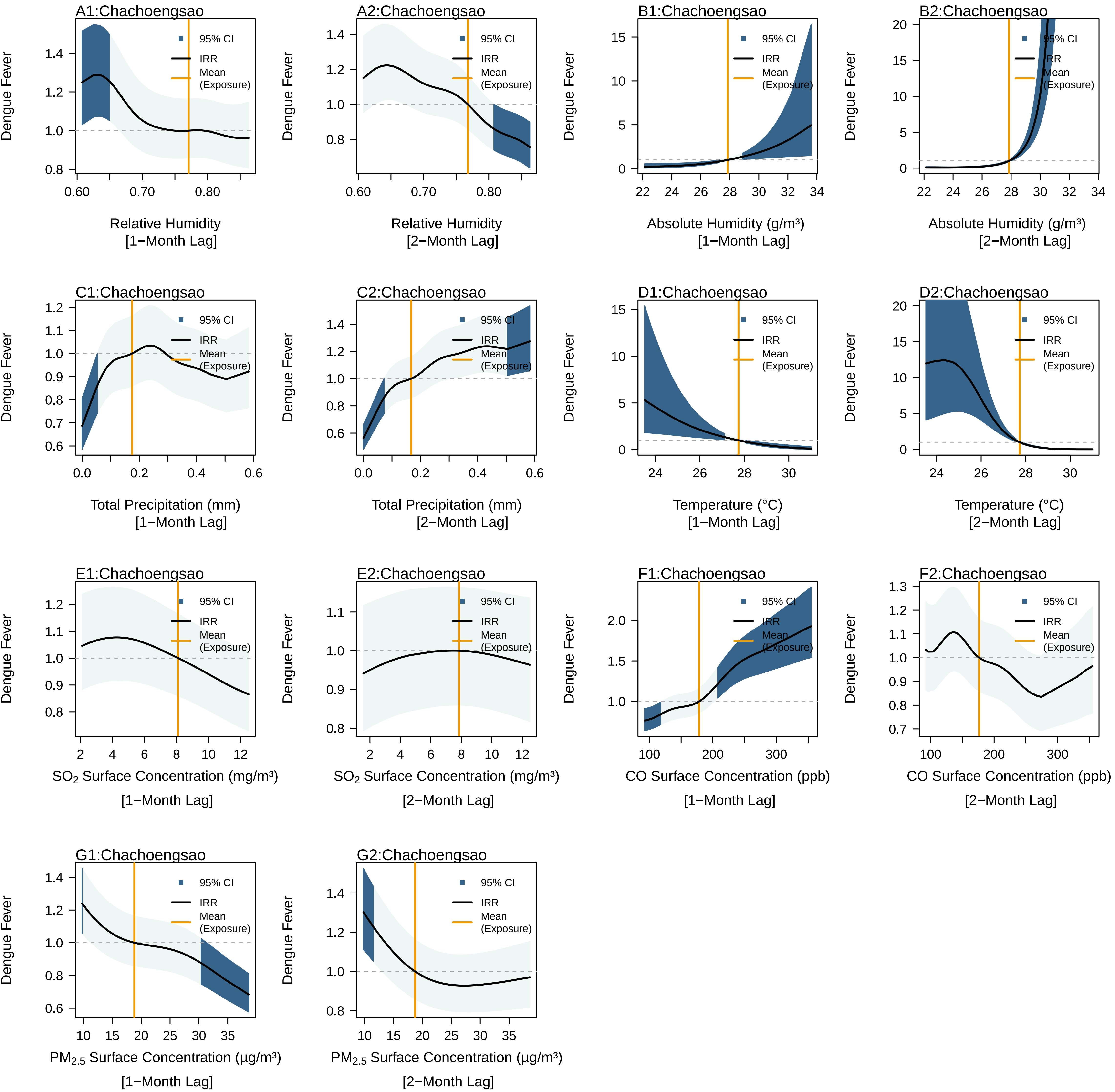
A1:Chachoengsao
Dengue Fever
1.4
1.2
1.0
0.8
95% CI
IRR
Mean
(Exposure)
0.60
0.70
0.80
Relative Humidity
[1−Month Lag]
A2:Chachoengsao
Dengue Fever
1.4
1.2
1.0
0.8
95% CI
IRR
Mean
(Exposure)
0.60
0.70
0.80
Relative Humidity
[2−Month Lag]
B1:Chachoengsao
Dengue Fever
15
10
5
0
95% CI
IRR
Mean
(Exposure)
22
24
26
28
30
32
34
Absolute Humidity (g/m³)
[1−Month Lag]
B2:Chachoengsao
Dengue Fever
20
15
10
5
0
95% CI
IRR
Mean
(Exposure)
22
24
26
28
30
32
34
Absolute Humidity (g/m³)
[2−Month Lag]
C1:Chachoengsao
Dengue Fever
1.2
1.1
1.0
0.9
0.8
0.7
0.6
95% CI
IRR
Mean
(Exposure)
0.0
0.2
0.4
0.6
Total Precipitation (mm)
[1−Month Lag]
C2:Chachoengsao
Dengue Fever
1.4
1.2
1.0
0.8
0.6
95% CI
IRR
Mean
(Exposure)
0.0
0.2
0.4
0.6
Total Precipitation (mm)
[2−Month Lag]
D1:Chachoengsao
Dengue Fever
15
10
5
0
95% CI
IRR
Mean
(Exposure)
24
26
28
30
Temperature (°C)
[1−Month Lag]
D2:Chachoengsao
Dengue Fever
20
15
10
5
0
95% CI
IRR
Mean
(Exposure)
24
26
28
30
Temperature (°C)
[2−Month Lag]
E1:Chachoengsao
Dengue Fever
1.2
1.1
1.0
0.9
0.8
95% CI
IRR
Mean
(Exposure)
2
4
6
8
10
12
SO_2 Surface Concentration (mg/m³)
[1−Month Lag]
E2:Chachoengsao
Dengue Fever
1.1
1.0
0.9
0.8
95% CI
IRR
Mean
(Exposure)
2
4
6
8
10
12
SO_2 Surface Concentration (mg/m³)
[2−Month Lag]
F1:Chachoengsao
Dengue Fever
2.0
1.5
1.0
95% CI
IRR
Mean
(Exposure)
100
200
300
CO Surface Concentration (ppb)
[1−Month Lag]
F2:Chachoengsao
Dengue Fever
1.3
1.2
1.1
1.0
0.9
0.8
0.7
95% CI
IRR
Mean
(Exposure)
100
200
300
CO Surface Concentration (ppb)
[2−Month Lag]
G1:Chachoengsao
Dengue Fever
1.4
1.2
1.0
0.8
0.6
95% CI
IRR
Mean
(Exposure)
10
15
20
25
30
35
$PM_{2.5}$ Surface Concentration (µg/m³)
[1−Month Lag]
G2:Chachoengsao
Dengue Fever
1.4
1.2
1.0
0.8
95% CI
IRR
Mean
(Exposure)
10
15
20
25
30
35
$PM_{2.5}$ Surface Concentration (µg/m³)
[2−Month Lag]

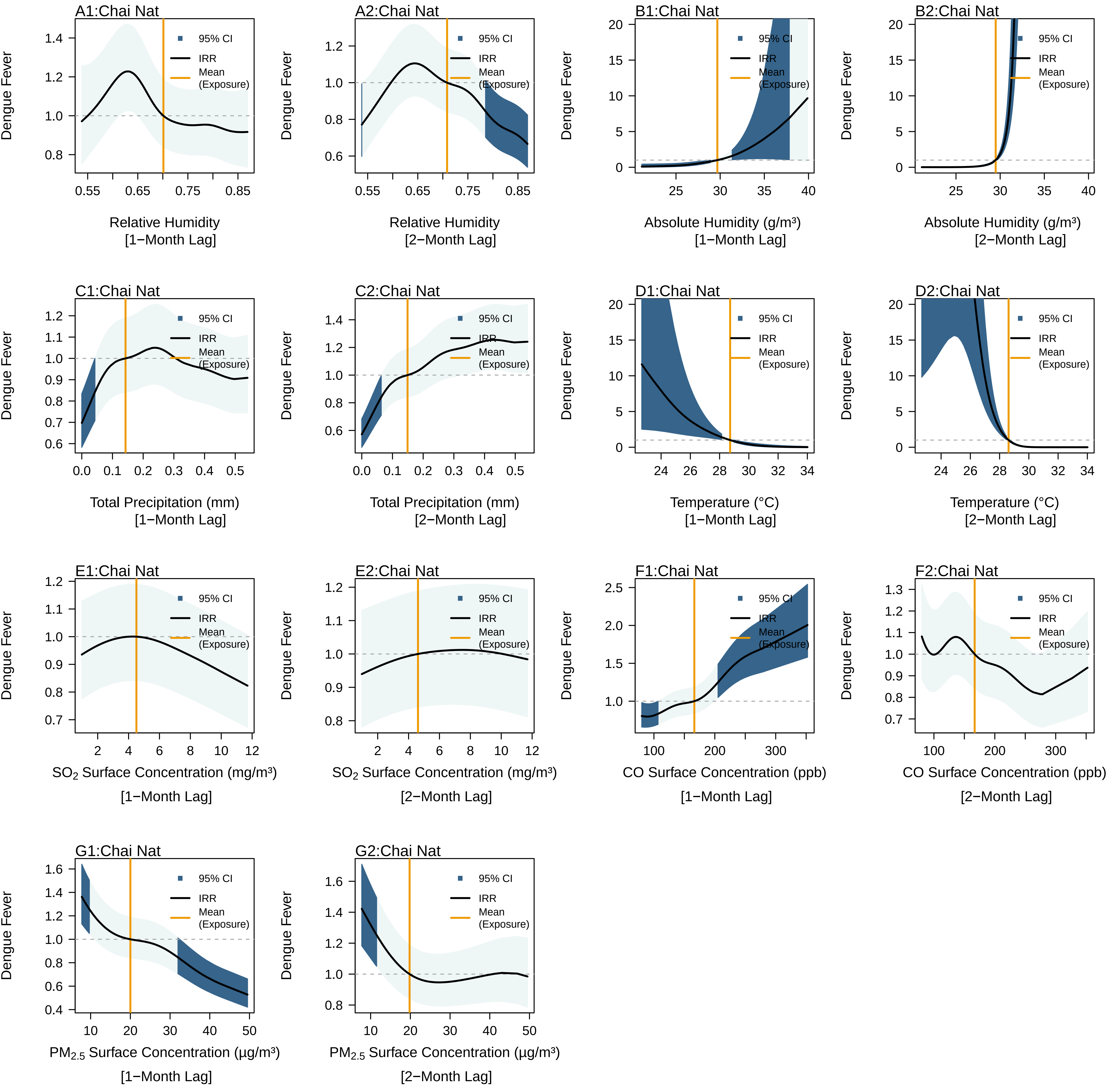
A1:Chai Nat
Dengue Fever
95% CI
IRR
Mean (Exposure)
Relative Humidity [1−Month Lag]
A2:Chai Nat
Relative Humidity [2−Month Lag]
B1:Chai Nat
Absolute Humidity (g/m³) [1−Month Lag]
B2:Chai Nat
Absolute Humidity (g/m³) [2−Month Lag]
C1:Chai Nat
Total Precipitation (mm) [1−Month Lag]
C2:Chai Nat
Total Precipitation (mm) [2−Month Lag]
D1:Chai Nat
Temperature (°C) [1−Month Lag]
D2:Chai Nat
Temperature (°C) [2−Month Lag]
E1:Chai Nat
SO2 Surface Concentration (mg/m³) [1−Month Lag]
E2:Chai Nat
SO2 Surface Concentration (mg/m³) [2−Month Lag]
F1:Chai Nat
CO Surface Concentration (ppb) [1−Month Lag]
F2:Chai Nat
CO Surface Concentration (ppb) [2−Month Lag]
G1:Chai Nat
PM2.5 Surface Concentration (µg/m³) [1−Month Lag]
G2:Chai Nat
PM2.5 Surface Concentration (µg/m³) [2−Month Lag]

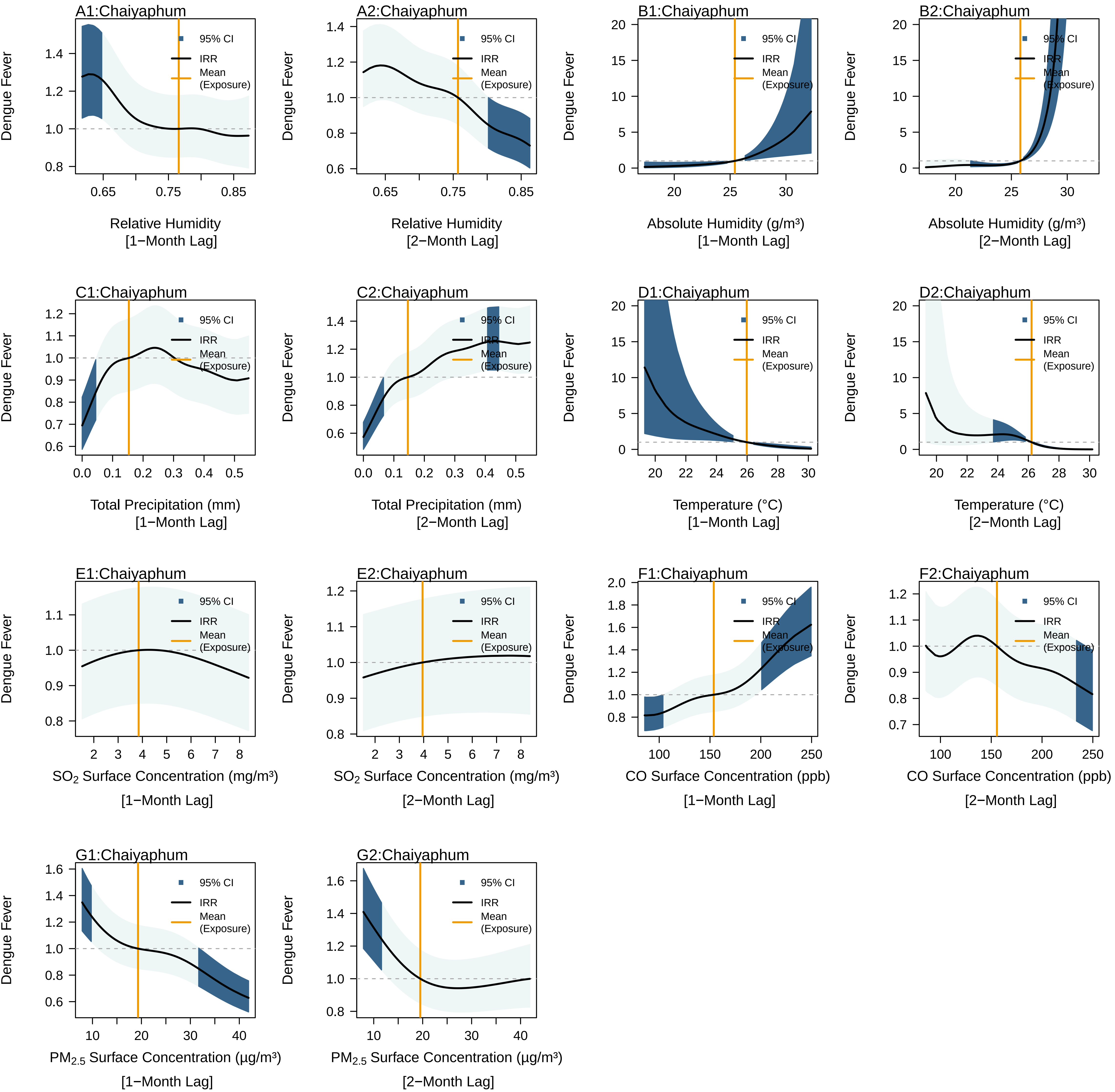
A1:Chaiyaphum
Dengue Fever
95% CI
IRR
Mean
(Exposure)
Relative Humidity
[1−Month Lag]
A2:Chaiyaphum
Dengue Fever
95% CI
IRR
Mean
(Exposure)
Relative Humidity
[2−Month Lag]
B1:Chaiyaphum
Dengue Fever
95% CI
IRR
Mean
(Exposure)
Absolute Humidity (g/m³)
[1−Month Lag]
B2:Chaiyaphum
Dengue Fever
95% CI
IRR
Mean
(Exposure)
Absolute Humidity (g/m³)
[2−Month Lag]
C1:Chaiyaphum
Dengue Fever
95% CI
IRR
Mean
(Exposure)
Total Precipitation (mm)
[1−Month Lag]
C2:Chaiyaphum
Dengue Fever
95% CI
IRR
Mean
(Exposure)
Total Precipitation (mm)
[2−Month Lag]
D1:Chaiyaphum
Dengue Fever
95% CI
IRR
Mean
(Exposure)
Temperature (°C)
[1−Month Lag]
D2:Chaiyaphum
Dengue Fever
95% CI
IRR
Mean
(Exposure)
Temperature (°C)
[2−Month Lag]
E1:Chaiyaphum
Dengue Fever
95% CI
IRR
Mean
(Exposure)
SO_2 Surface Concentration (mg/m³)
[1−Month Lag]
E2:Chaiyaphum
Dengue Fever
95% CI
IRR
Mean
(Exposure)
SO_2 Surface Concentration (mg/m³)
[2−Month Lag]
F1:Chaiyaphum
Dengue Fever
95% CI
IRR
Mean
(Exposure)
CO Surface Concentration (ppb)
[1−Month Lag]
F2:Chaiyaphum
Dengue Fever
95% CI
IRR
Mean
(Exposure)
CO Surface Concentration (ppb)
[2−Month Lag]
G1:Chaiyaphum
Dengue Fever
95% CI
IRR
Mean
(Exposure)
$PM_{2.5}$ Surface Concentration (µg/m³)
[1−Month Lag]
G2:Chaiyaphum
Dengue Fever
95% CI
IRR
Mean
(Exposure)
$PM_{2.5}$ Surface Concentration (µg/m³)
[2−Month Lag]

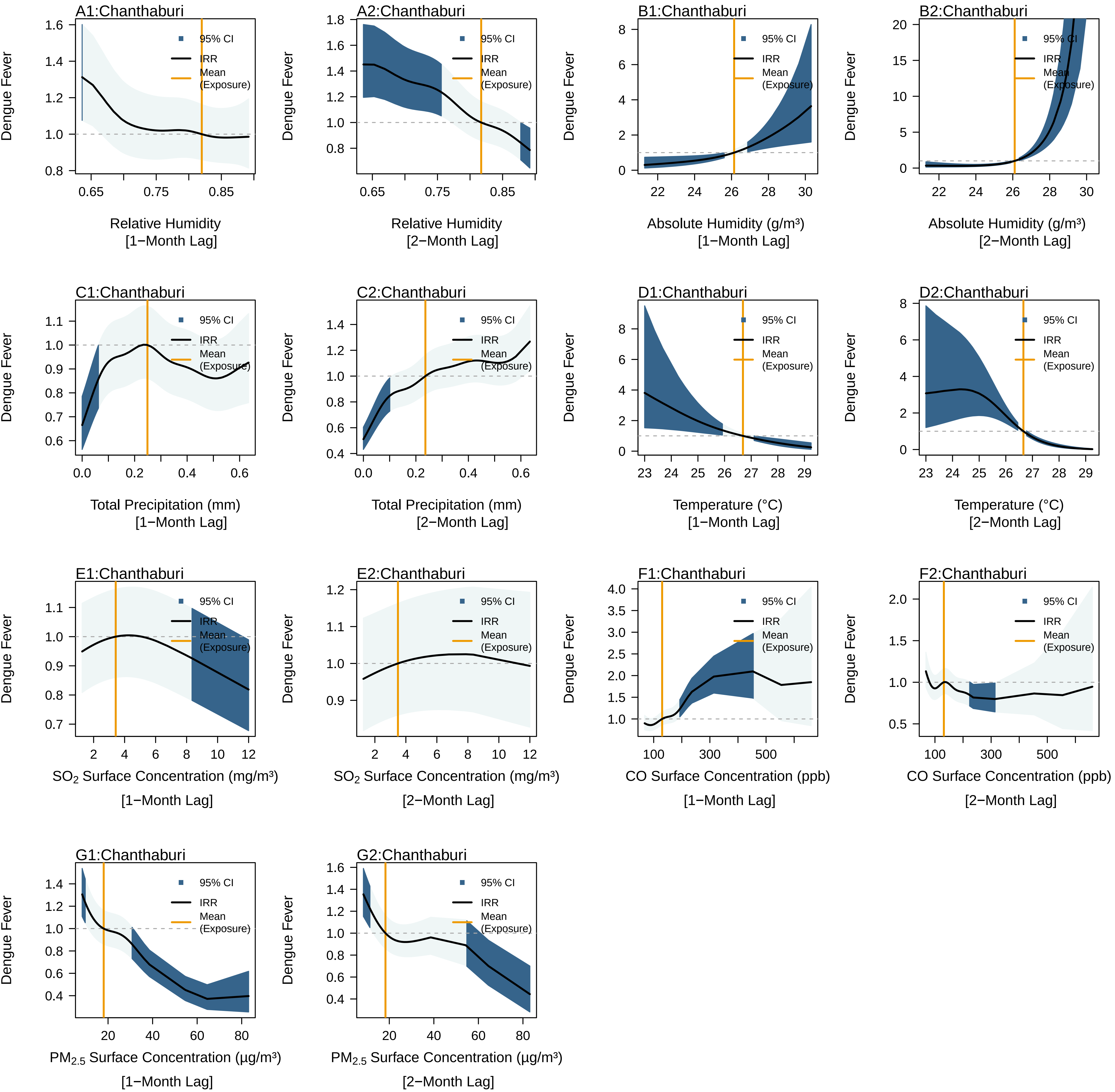
A1:Chanthaburi
A2:Chanthaburi
B1:Chanthaburi
B2:Chanthaburi
C1:Chanthaburi
C2:Chanthaburi
D1:Chanthaburi
D2:Chanthaburi
E1:Chanthaburi
E2:Chanthaburi
F1:Chanthaburi
F2:Chanthaburi
G1:Chanthaburi
G2:Chanthaburi
Dengue Fever
95% CI
IRR
Mean (Exposure)
Relative Humidity [1−Month Lag]
Relative Humidity [2−Month Lag]
Absolute Humidity (g/m³) [1−Month Lag]
Absolute Humidity (g/m³) [2−Month Lag]
Total Precipitation (mm) [1−Month Lag]
Total Precipitation (mm) [2−Month Lag]
Temperature (°C) [1−Month Lag]
Temperature (°C) [2−Month Lag]
SO_2 Surface Concentration (mg/m³) [1−Month Lag]
SO_2 Surface Concentration (mg/m³) [2−Month Lag]
CO Surface Concentration (ppb) [1−Month Lag]
CO Surface Concentration (ppb) [2−Month Lag]
$PM_{2.5}$ Surface Concentration (µg/m³) [1−Month Lag]
$PM_{2.5}$ Surface Concentration (µg/m³) [2−Month Lag]

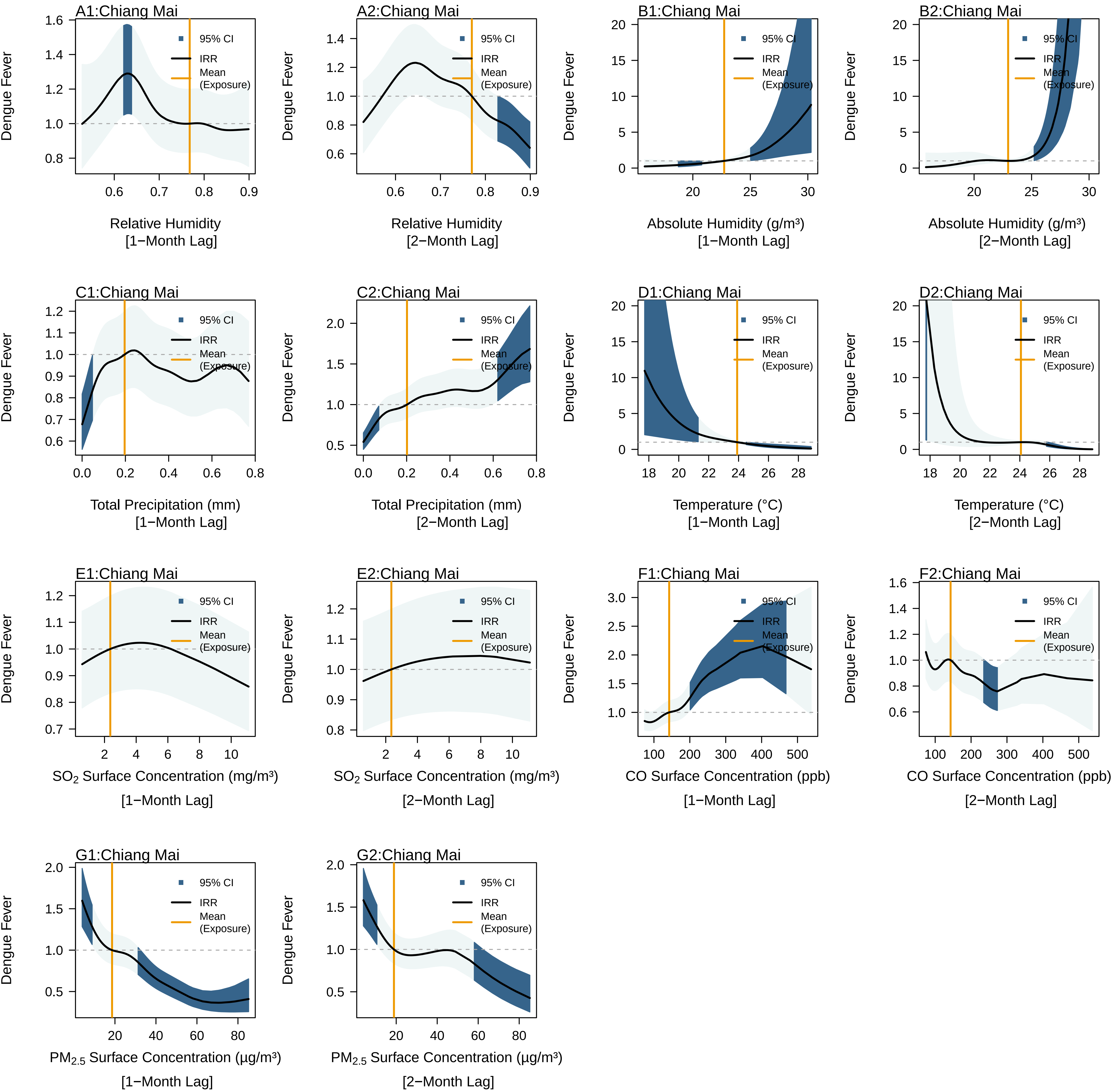
A1:Chiang Mai
Dengue Fever
Relative Humidity
[1−Month Lag]
A2:Chiang Mai
Dengue Fever
Relative Humidity
[2−Month Lag]
B1:Chiang Mai
Dengue Fever
Absolute Humidity (g/m³)
[1−Month Lag]
B2:Chiang Mai
Dengue Fever
Absolute Humidity (g/m³)
[2−Month Lag]
C1:Chiang Mai
Dengue Fever
Total Precipitation (mm)
[1−Month Lag]
C2:Chiang Mai
Dengue Fever
Total Precipitation (mm)
[2−Month Lag]
D1:Chiang Mai
Dengue Fever
Temperature (°C)
[1−Month Lag]
D2:Chiang Mai
Dengue Fever
Temperature (°C)
[2−Month Lag]
E1:Chiang Mai
Dengue Fever
SO_2 Surface Concentration (mg/m³)
[1−Month Lag]
E2:Chiang Mai
Dengue Fever
SO_2 Surface Concentration (mg/m³)
[2−Month Lag]
F1:Chiang Mai
Dengue Fever
CO Surface Concentration (ppb)
[1−Month Lag]
F2:Chiang Mai
Dengue Fever
CO Surface Concentration (ppb)
[2−Month Lag]
G1:Chiang Mai
Dengue Fever
$PM_{2.5}$ Surface Concentration (μg/m³)
[1−Month Lag]
G2:Chiang Mai
Dengue Fever
$PM_{2.5}$ Surface Concentration (μg/m³)
[2−Month Lag]
95% CI
IRR
Mean
(Exposure)

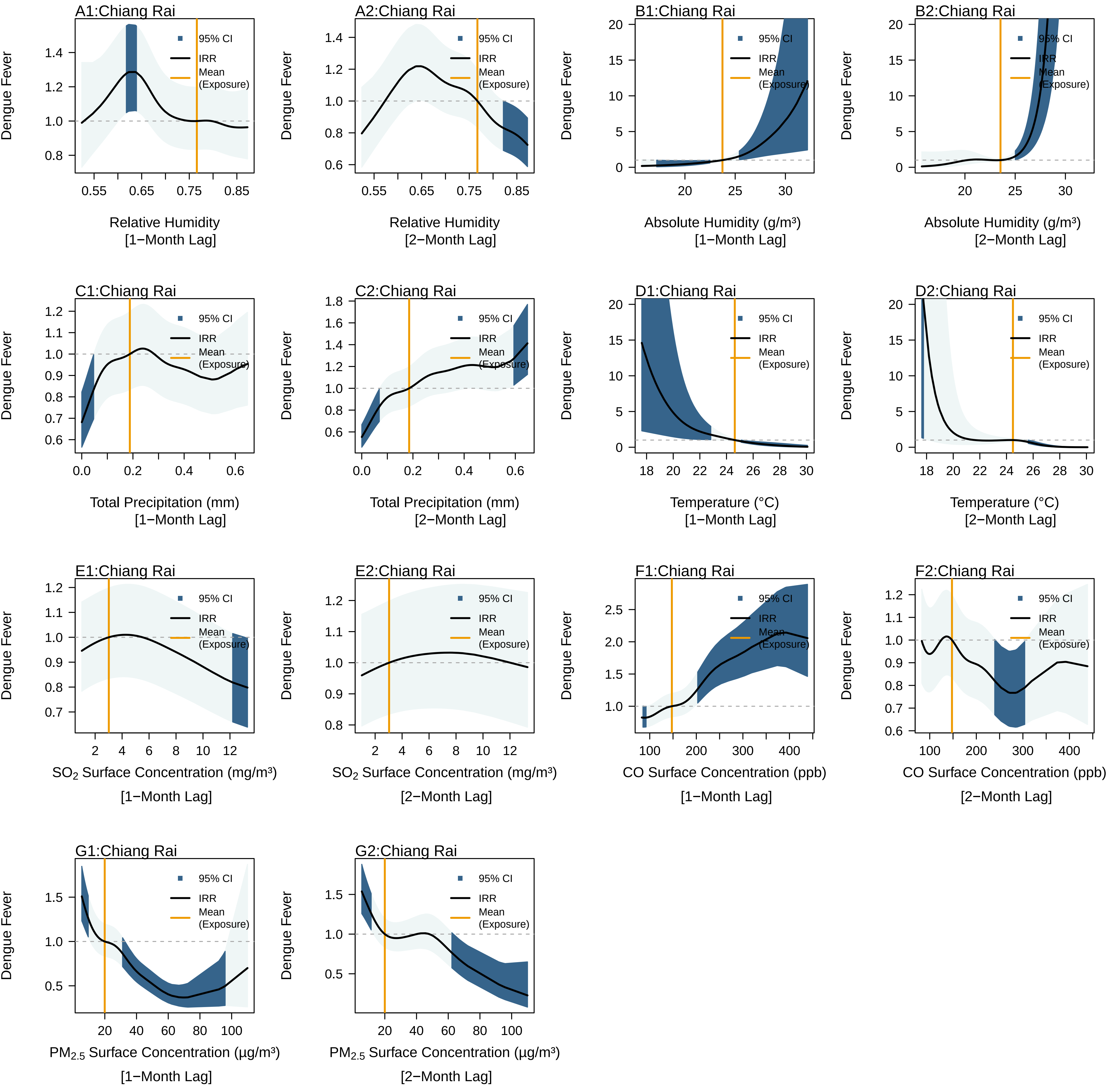
A1:Chiang Rai
A2:Chiang Rai
B1:Chiang Rai
B2:Chiang Rai
C1:Chiang Rai
C2:Chiang Rai
D1:Chiang Rai
D2:Chiang Rai
E1:Chiang Rai
E2:Chiang Rai
F1:Chiang Rai
F2:Chiang Rai
G1:Chiang Rai
G2:Chiang Rai
95% CI
IRR
Mean (Exposure)
Dengue Fever
Relative Humidity [1−Month Lag]
Relative Humidity [2−Month Lag]
Absolute Humidity (g/m³) [1−Month Lag]
Absolute Humidity (g/m³) [2−Month Lag]
Total Precipitation (mm) [1−Month Lag]
Total Precipitation (mm) [2−Month Lag]
Temperature (°C) [1−Month Lag]
Temperature (°C) [2−Month Lag]
SO_2 Surface Concentration (mg/m³) [1−Month Lag]
SO_2 Surface Concentration (mg/m³) [2−Month Lag]
CO Surface Concentration (ppb) [1−Month Lag]
CO Surface Concentration (ppb) [2−Month Lag]
$PM_{2.5}$ Surface Concentration (µg/m³) [1−Month Lag]
$PM_{2.5}$ Surface Concentration (µg/m³) [2−Month Lag]

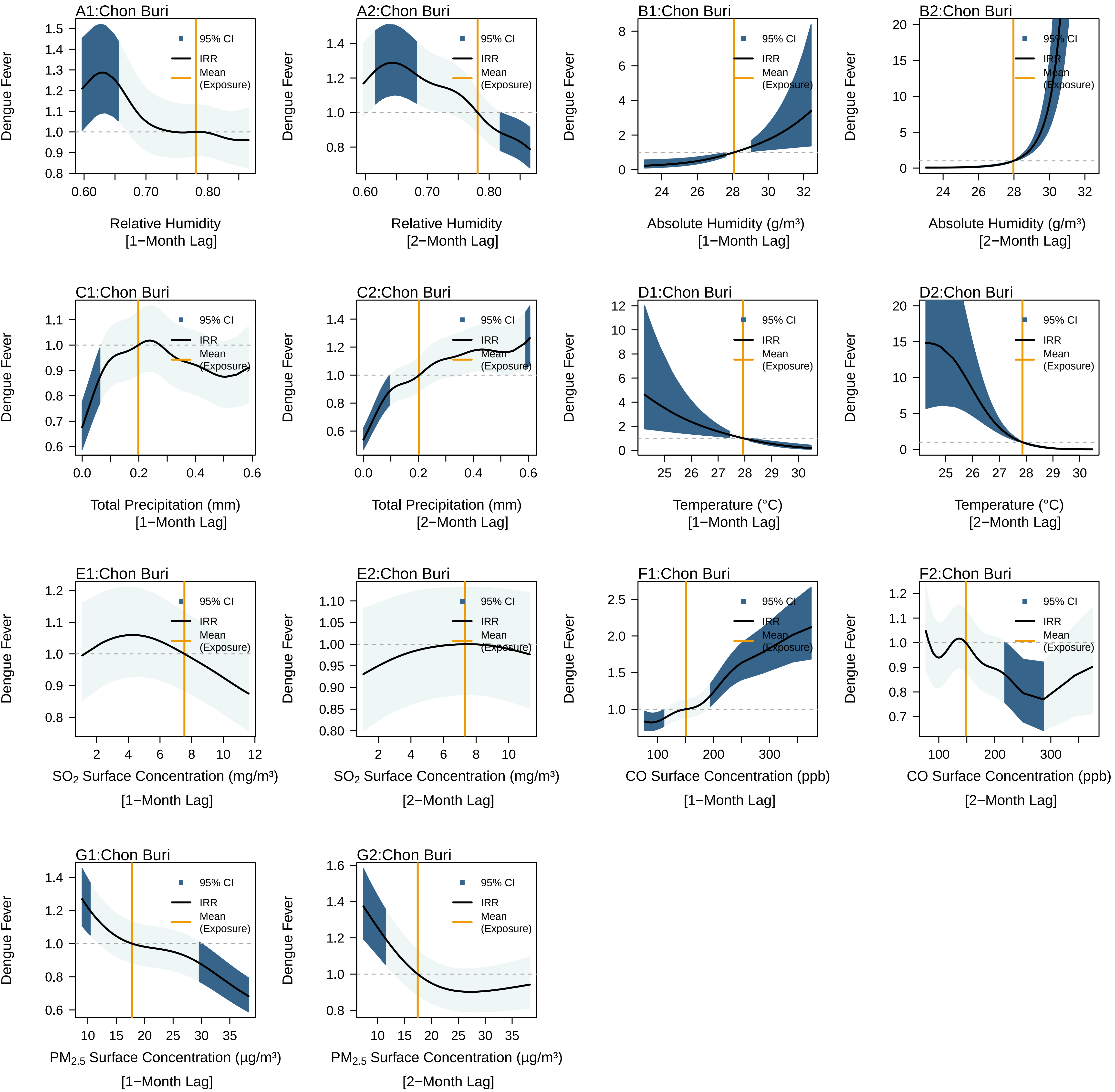
A1:Chon Buri
A2:Chon Buri
B1:Chon Buri
B2:Chon Buri
C1:Chon Buri
C2:Chon Buri
D1:Chon Buri
D2:Chon Buri
E1:Chon Buri
E2:Chon Buri
F1:Chon Buri
F2:Chon Buri
G1:Chon Buri
G2:Chon Buri
Dengue Fever
95% CI
IRR
Mean (Exposure)
Relative Humidity [1−Month Lag]
Relative Humidity [2−Month Lag]
Absolute Humidity (g/m³) [1−Month Lag]
Absolute Humidity (g/m³) [2−Month Lag]
Total Precipitation (mm) [1−Month Lag]
Total Precipitation (mm) [2−Month Lag]
Temperature (°C) [1−Month Lag]
Temperature (°C) [2−Month Lag]
SO2 Surface Concentration (mg/m³) [1−Month Lag]
SO2 Surface Concentration (mg/m³) [2−Month Lag]
CO Surface Concentration (ppb) [1−Month Lag]
CO Surface Concentration (ppb) [2−Month Lag]
PM2.5 Surface Concentration (μg/m³) [1−Month Lag]
PM2.5 Surface Concentration (μg/m³) [2−Month Lag]

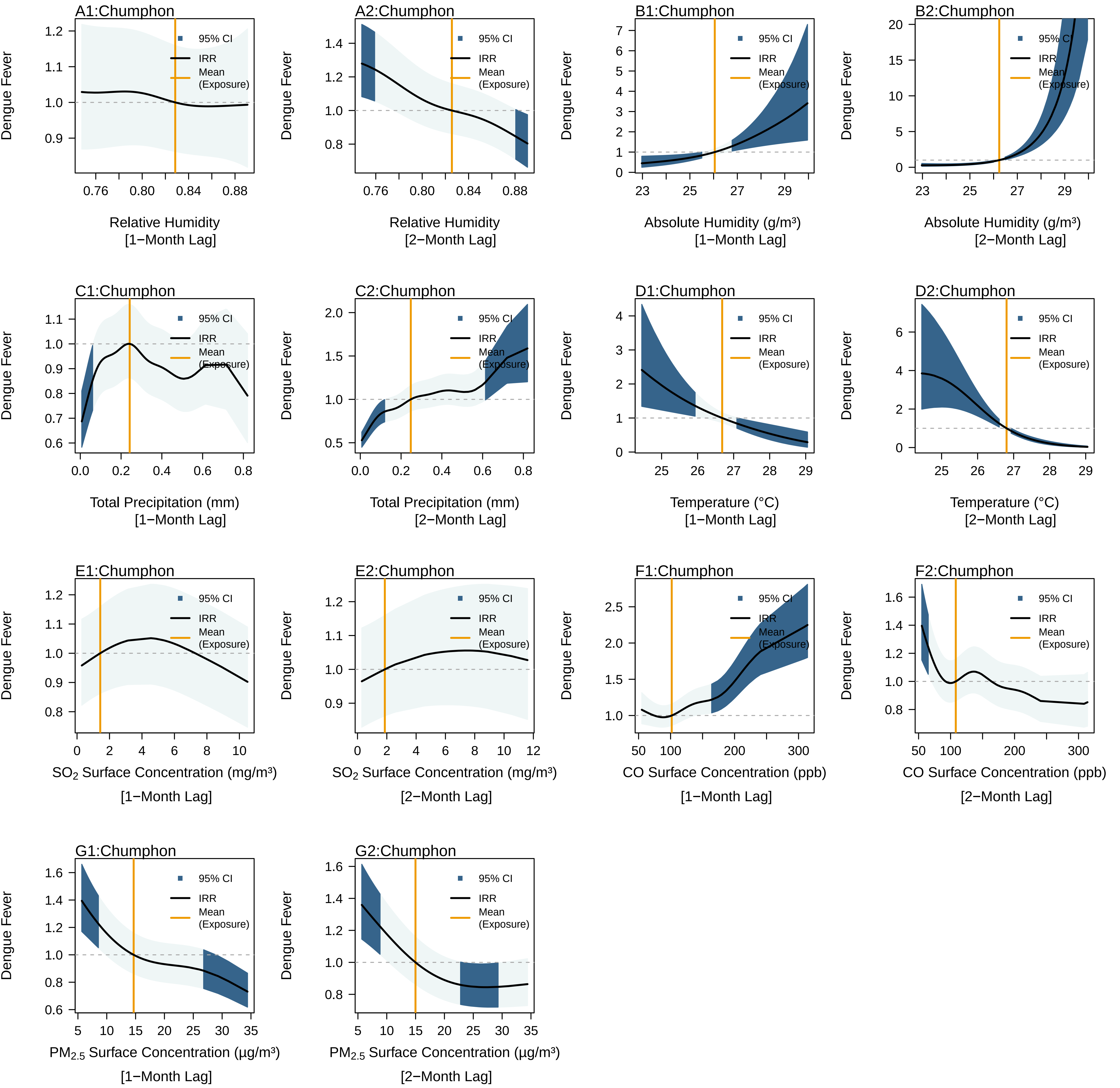
A1:Chumphon
Dengue Fever
95% CI
IRR
Mean
(Exposure)
Relative Humidity
[1−Month Lag]
A2:Chumphon
Relative Humidity
[2−Month Lag]
B1:Chumphon
Absolute Humidity (g/m³)
[1−Month Lag]
B2:Chumphon
Absolute Humidity (g/m³)
[2−Month Lag]
C1:Chumphon
Total Precipitation (mm)
[1−Month Lag]
C2:Chumphon
Total Precipitation (mm)
[2−Month Lag]
D1:Chumphon
Temperature (°C)
[1−Month Lag]
D2:Chumphon
Temperature (°C)
[2−Month Lag]
E1:Chumphon
SO_2 Surface Concentration (mg/m³)
[1−Month Lag]
E2:Chumphon
SO_2 Surface Concentration (mg/m³)
[2−Month Lag]
F1:Chumphon
CO Surface Concentration (ppb)
[1−Month Lag]
F2:Chumphon
CO Surface Concentration (ppb)
[2−Month Lag]
G1:Chumphon
$PM_{2.5}$ Surface Concentration (µg/m³)
[1−Month Lag]
G2:Chumphon
$PM_{2.5}$ Surface Concentration (µg/m³)
[2−Month Lag]

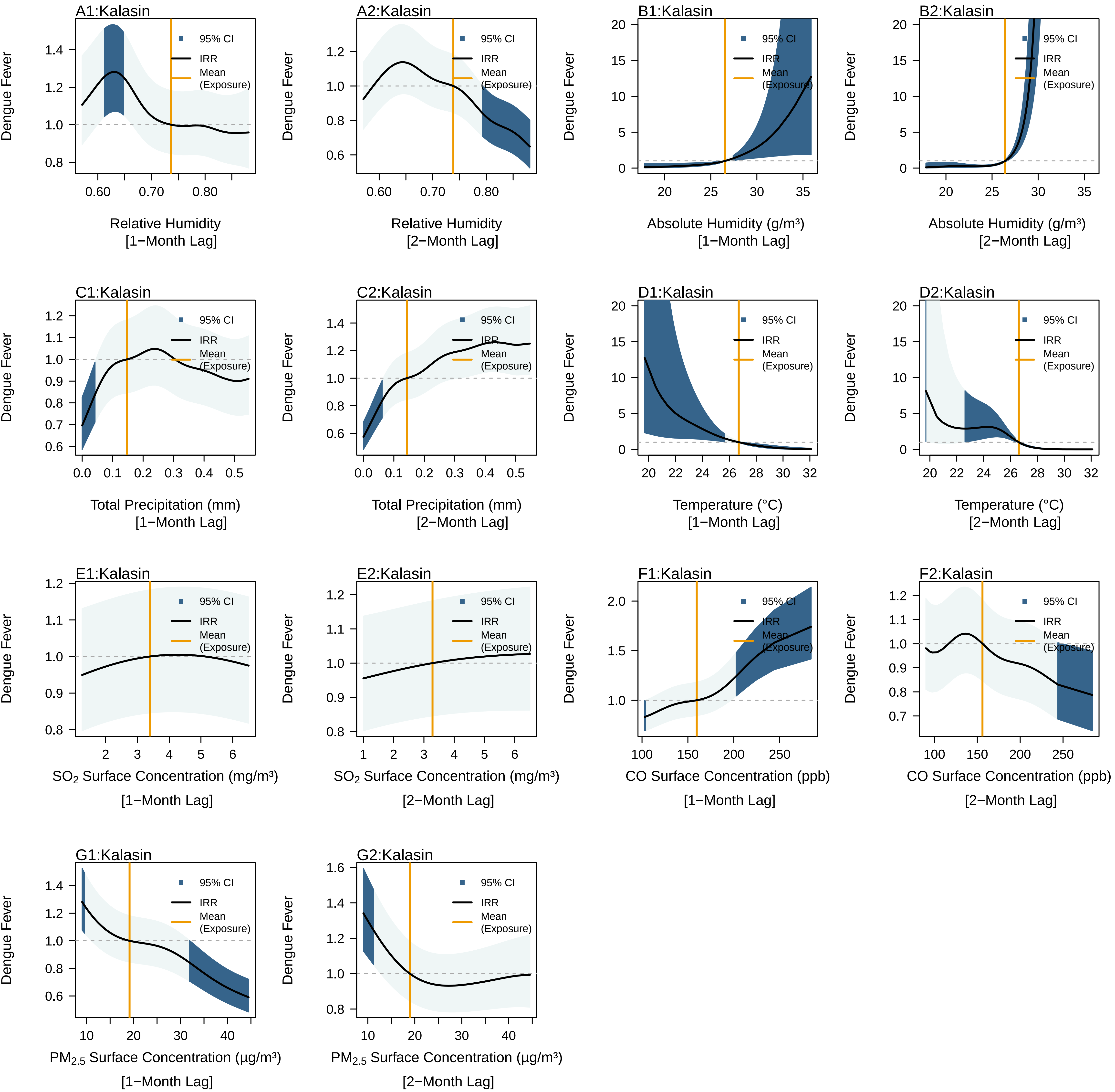
A1:Kalasin
A2:Kalasin
B1:Kalasin
B2:Kalasin
C1:Kalasin
C2:Kalasin
D1:Kalasin
D2:Kalasin
E1:Kalasin
E2:Kalasin
F1:Kalasin
F2:Kalasin
G1:Kalasin
G2:Kalasin
Dengue Fever
95% CI
IRR
Mean
(Exposure)
Relative Humidity
[1−Month Lag]
[2−Month Lag]
Absolute Humidity (g/m³)
Total Precipitation (mm)
Temperature (°C)
SO2 Surface Concentration (mg/m³)
CO Surface Concentration (ppb)
PM2.5 Surface Concentration (µg/m³)

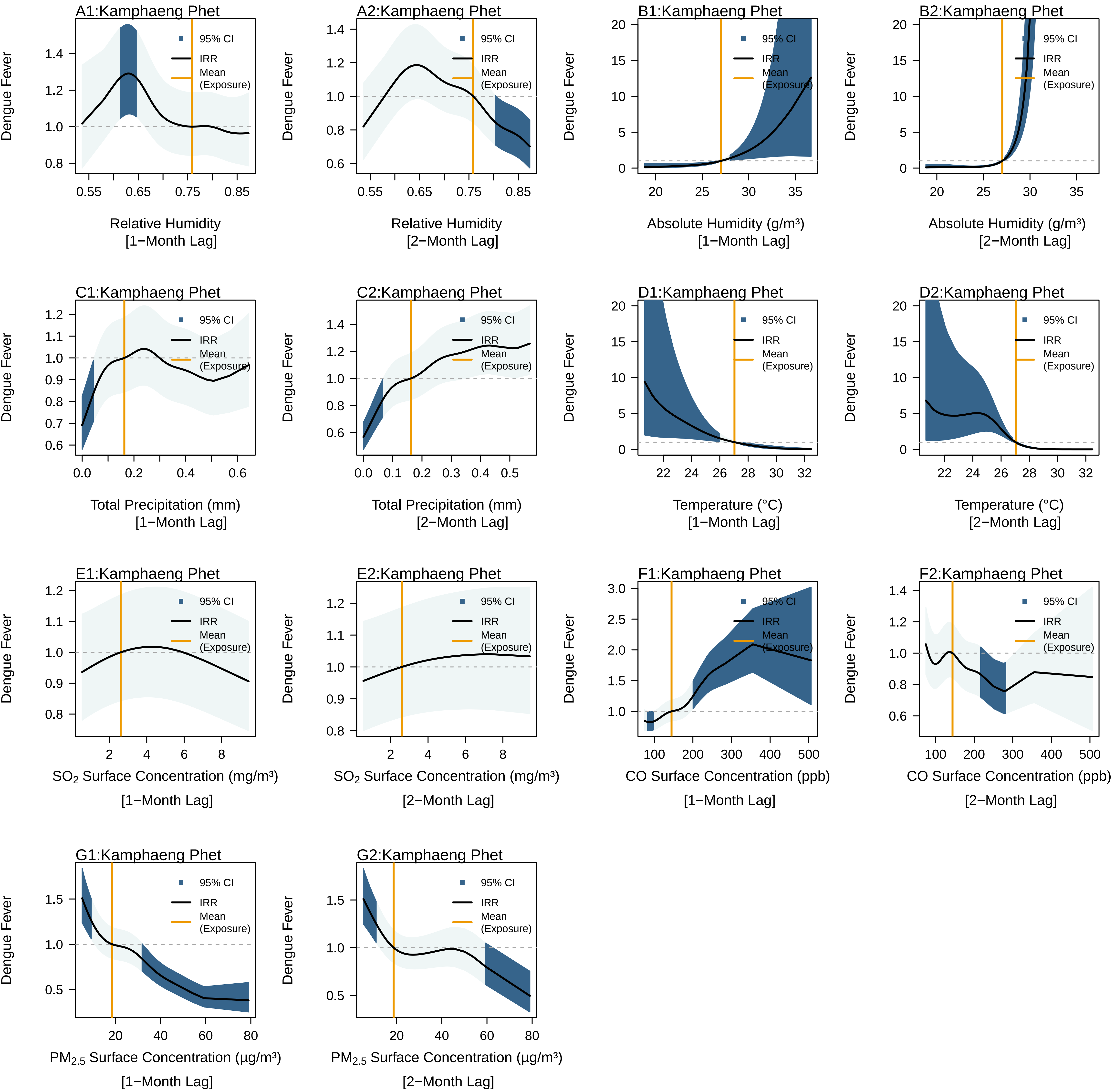
A1:Kamphaeng Phet
A2:Kamphaeng Phet
B1:Kamphaeng Phet
B2:Kamphaeng Phet
95% CI
IRR
Mean (Exposure)
Dengue Fever
Relative Humidity [1−Month Lag]
Relative Humidity [2−Month Lag]
Absolute Humidity (g/m³) [1−Month Lag]
Absolute Humidity (g/m³) [2−Month Lag]
C1:Kamphaeng Phet
C2:Kamphaeng Phet
D1:Kamphaeng Phet
D2:Kamphaeng Phet
Total Precipitation (mm) [1−Month Lag]
Total Precipitation (mm) [2−Month Lag]
Temperature (°C) [1−Month Lag]
Temperature (°C) [2−Month Lag]
E1:Kamphaeng Phet
E2:Kamphaeng Phet
F1:Kamphaeng Phet
F2:Kamphaeng Phet
SO2 Surface Concentration (mg/m³) [1−Month Lag]
SO2 Surface Concentration (mg/m³) [2−Month Lag]
CO Surface Concentration (ppb) [1−Month Lag]
CO Surface Concentration (ppb) [2−Month Lag]
G1:Kamphaeng Phet
G2:Kamphaeng Phet
PM2.5 Surface Concentration (µg/m³) [1−Month Lag]
PM2.5 Surface Concentration (µg/m³) [2−Month Lag]

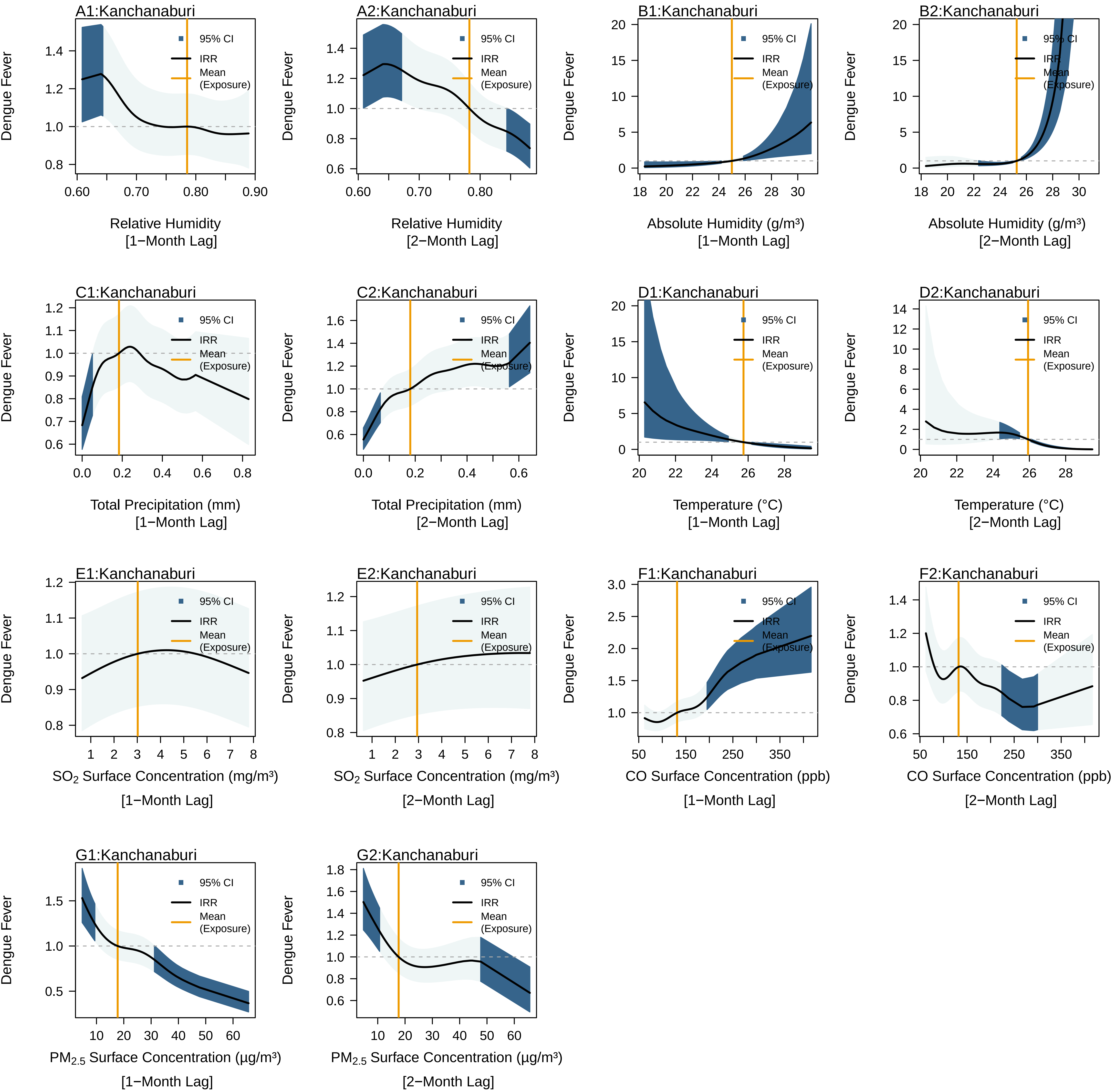

A1:Kanchanaburi
Dengue Fever
95% CI
IRR
Mean (Exposure)
1.4 1.2 1.0 0.8
0.60 0.70 0.80 0.90
Relative Humidity [1−Month Lag]
A2:Kanchanaburi
Dengue Fever
95% CI
IRR
Mean (Exposure)
1.4 1.2 1.0 0.8 0.6
0.60 0.70 0.80
Relative Humidity [2−Month Lag]
B1:Kanchanaburi
Dengue Fever
95% CI
IRR
Mean (Exposure)
20 15 10 5 0
18 20 22 24 26 28 30
Absolute Humidity (g/m³) [1−Month Lag]
B2:Kanchanaburi
Dengue Fever
95% CI
IRR
Mean (Exposure)
20 15 10 5 0
18 20 22 24 26 28 30
Absolute Humidity (g/m³) [2−Month Lag]
C1:Kanchanaburi
Dengue Fever
95% CI
IRR
Mean (Exposure)
1.2 1.1 1.0 0.9 0.8 0.7 0.6
0.0 0.2 0.4 0.6 0.8
Total Precipitation (mm) [1−Month Lag]
C2:Kanchanaburi
Dengue Fever
95% CI
IRR
Mean (Exposure)
1.6 1.4 1.2 1.0 0.8 0.6
0.0 0.2 0.4 0.6
Total Precipitation (mm) [2−Month Lag]
D1:Kanchanaburi
Dengue Fever
95% CI
IRR
Mean (Exposure)
20 15 10 5 0
20 22 24 26 28
Temperature (°C) [1−Month Lag]
D2:Kanchanaburi
Dengue Fever
95% CI
IRR
Mean (Exposure)
14 12 10 8 6 4 2 0
20 22 24 26 28
Temperature (°C) [2−Month Lag]
E1:Kanchanaburi
Dengue Fever
95% CI
IRR
Mean (Exposure)
1.2 1.1 1.0 0.9 0.8
1 2 3 4 5 6 7 8
SO2 Surface Concentration (mg/m³) [1−Month Lag]
E2:Kanchanaburi
Dengue Fever
95% CI
IRR
Mean (Exposure)
1.2 1.1 1.0 0.9 0.8
1 2 3 4 5 6 7 8
SO2 Surface Concentration (mg/m³) [2−Month Lag]
F1:Kanchanaburi
Dengue Fever
95% CI
IRR
Mean (Exposure)
3.0 2.5 2.0 1.5 1.0
50 150 250 350
CO Surface Concentration (ppb) [1−Month Lag]
F2:Kanchanaburi
Dengue Fever
95% CI
IRR
Mean (Exposure)
1.4 1.2 1.0 0.8 0.6
50 150 250 350
CO Surface Concentration (ppb) [2−Month Lag]
G1:Kanchanaburi
Dengue Fever
95% CI
IRR
Mean (Exposure)
1.5 1.0 0.5
10 20 30 40 50 60
PM2.5 Surface Concentration (μg/m³) [1−Month Lag]
G2:Kanchanaburi
Dengue Fever
95% CI
IRR
Mean (Exposure)
1.8 1.6 1.4 1.2 1.0 0.8 0.6
10 20 30 40 50 60
PM2.5 Surface Concentration (μg/m³) [2−Month Lag]

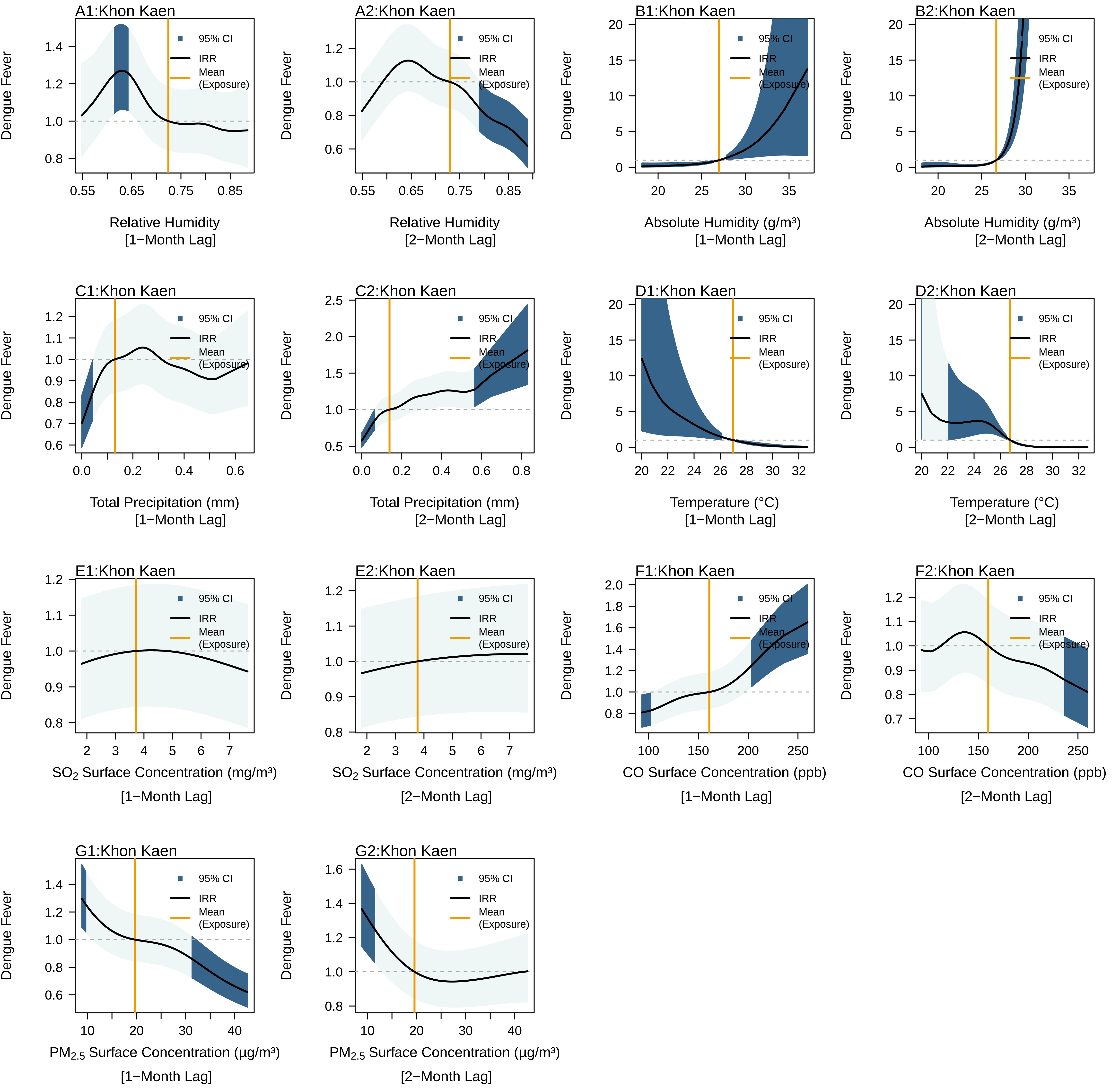

A1:Khon Kaen
A2:Khon Kaen
B1:Khon Kaen
B2:Khon Kaen
C1:Khon Kaen
C2:Khon Kaen
D1:Khon Kaen
D2:Khon Kaen
E1:Khon Kaen
E2:Khon Kaen
F1:Khon Kaen
F2:Khon Kaen
G1:Khon Kaen
G2:Khon Kaen
95% CI
IRR
Mean (Exposure)
Dengue Fever
Relative Humidity [1−Month Lag]
Relative Humidity [2−Month Lag]
Absolute Humidity (g/m³) [1−Month Lag]
Absolute Humidity (g/m³) [2−Month Lag]
Total Precipitation (mm) [1−Month Lag]
Total Precipitation (mm) [2−Month Lag]
Temperature (°C) [1−Month Lag]
Temperature (°C) [2−Month Lag]
SO2 Surface Concentration (mg/m³) [1−Month Lag]
SO2 Surface Concentration (mg/m³) [2−Month Lag]
CO Surface Concentration (ppb) [1−Month Lag]
CO Surface Concentration (ppb) [2−Month Lag]
PM2.5 Surface Concentration (μg/m³) [1−Month Lag]
PM2.5 Surface Concentration (μg/m³) [2−Month Lag]

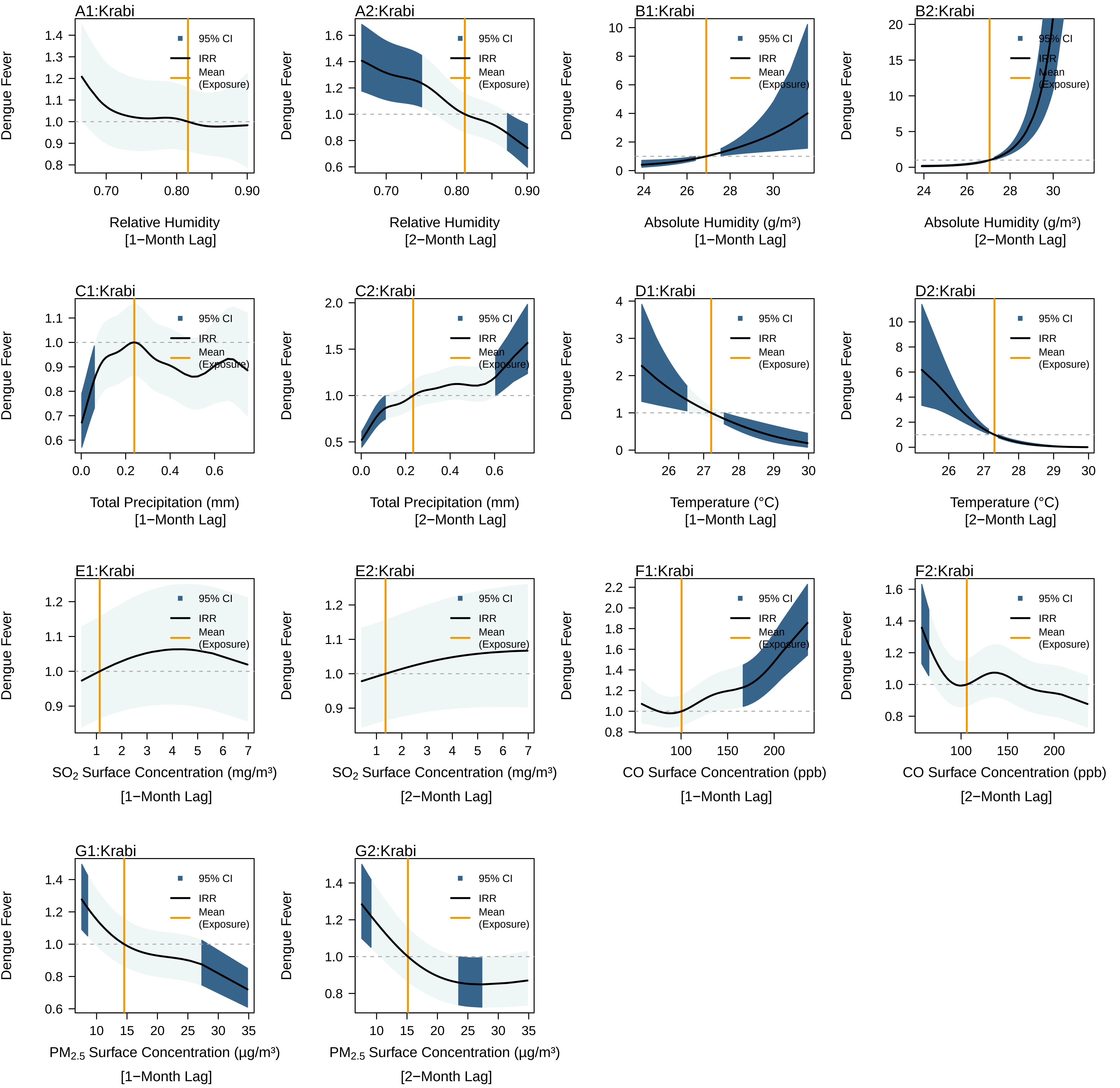
A1:Krabi
A2:Krabi
B1:Krabi
B2:Krabi
Dengue Fever
95% CI
IRR
Mean
(Exposure)
Relative Humidity
[1−Month Lag]
Relative Humidity
[2−Month Lag]
Absolute Humidity (g/m³)
[1−Month Lag]
Absolute Humidity (g/m³)
[2−Month Lag]
C1:Krabi
C2:Krabi
D1:Krabi
D2:Krabi
Total Precipitation (mm)
[1−Month Lag]
Total Precipitation (mm)
[2−Month Lag]
Temperature (°C)
[1−Month Lag]
Temperature (°C)
[2−Month Lag]
E1:Krabi
E2:Krabi
F1:Krabi
F2:Krabi
SO_2 Surface Concentration (mg/m³)
[1−Month Lag]
SO_2 Surface Concentration (mg/m³)
[2−Month Lag]
CO Surface Concentration (ppb)
[1−Month Lag]
CO Surface Concentration (ppb)
[2−Month Lag]
G1:Krabi
G2:Krabi
$PM_{2.5}$ Surface Concentration (µg/m³)
[1−Month Lag]
$PM_{2.5}$ Surface Concentration (µg/m³)
[2−Month Lag]

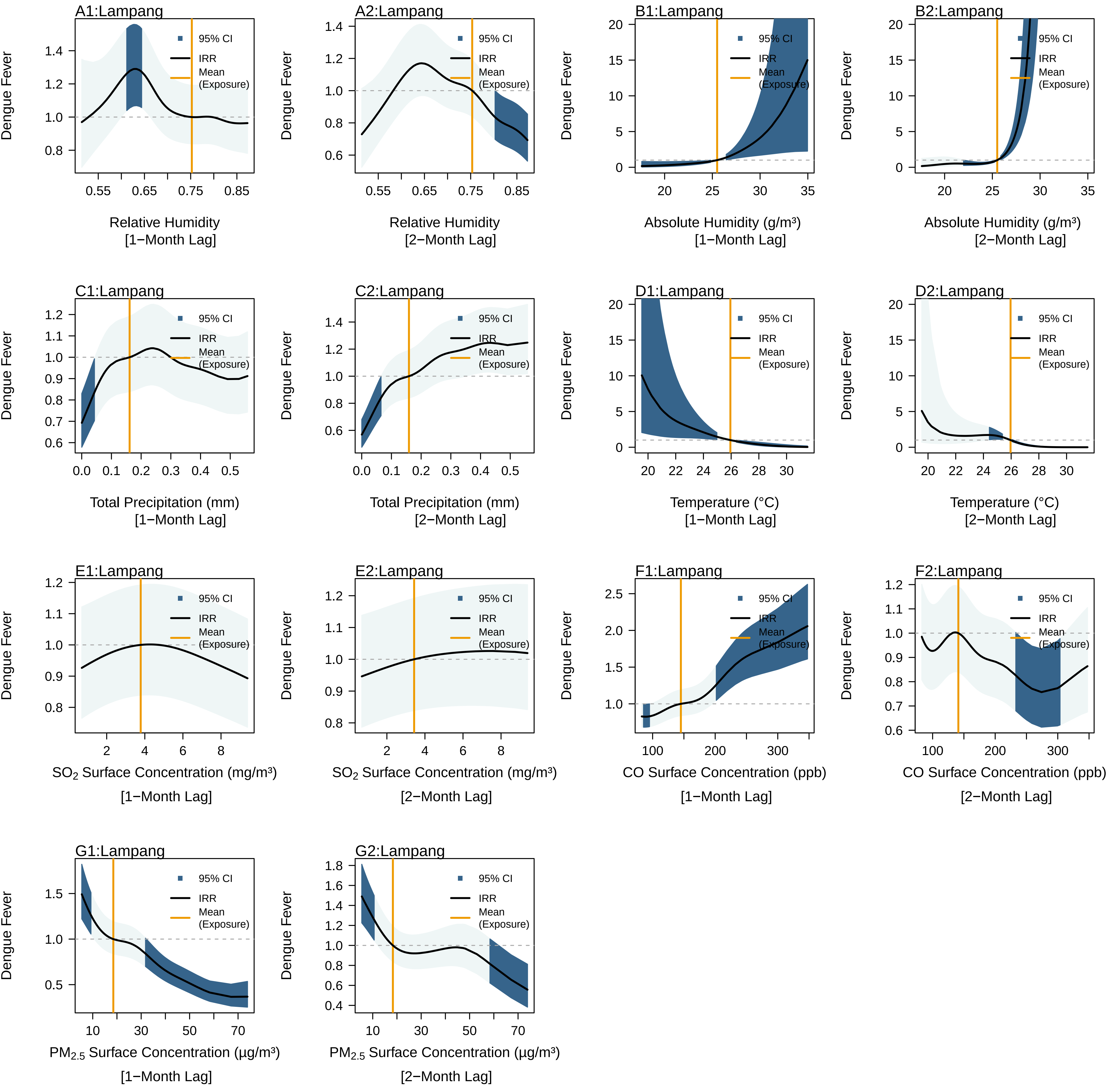
A1:Lampang
Dengue Fever
95% CI
IRR
Mean (Exposure)
Relative Humidity [1−Month Lag]
A2:Lampang
Relative Humidity [2−Month Lag]
B1:Lampang
Absolute Humidity (g/m³) [1−Month Lag]
B2:Lampang
Absolute Humidity (g/m³) [2−Month Lag]
C1:Lampang
Total Precipitation (mm) [1−Month Lag]
C2:Lampang
Total Precipitation (mm) [2−Month Lag]
D1:Lampang
Temperature (°C) [1−Month Lag]
D2:Lampang
Temperature (°C) [2−Month Lag]
E1:Lampang
SO_2 Surface Concentration (mg/m³) [1−Month Lag]
E2:Lampang
SO_2 Surface Concentration (mg/m³) [2−Month Lag]
F1:Lampang
CO Surface Concentration (ppb) [1−Month Lag]
F2:Lampang
CO Surface Concentration (ppb) [2−Month Lag]
G1:Lampang
$PM_{2.5}$ Surface Concentration (µg/m³) [1−Month Lag]
G2:Lampang
$PM_{2.5}$ Surface Concentration (µg/m³) [2−Month Lag]

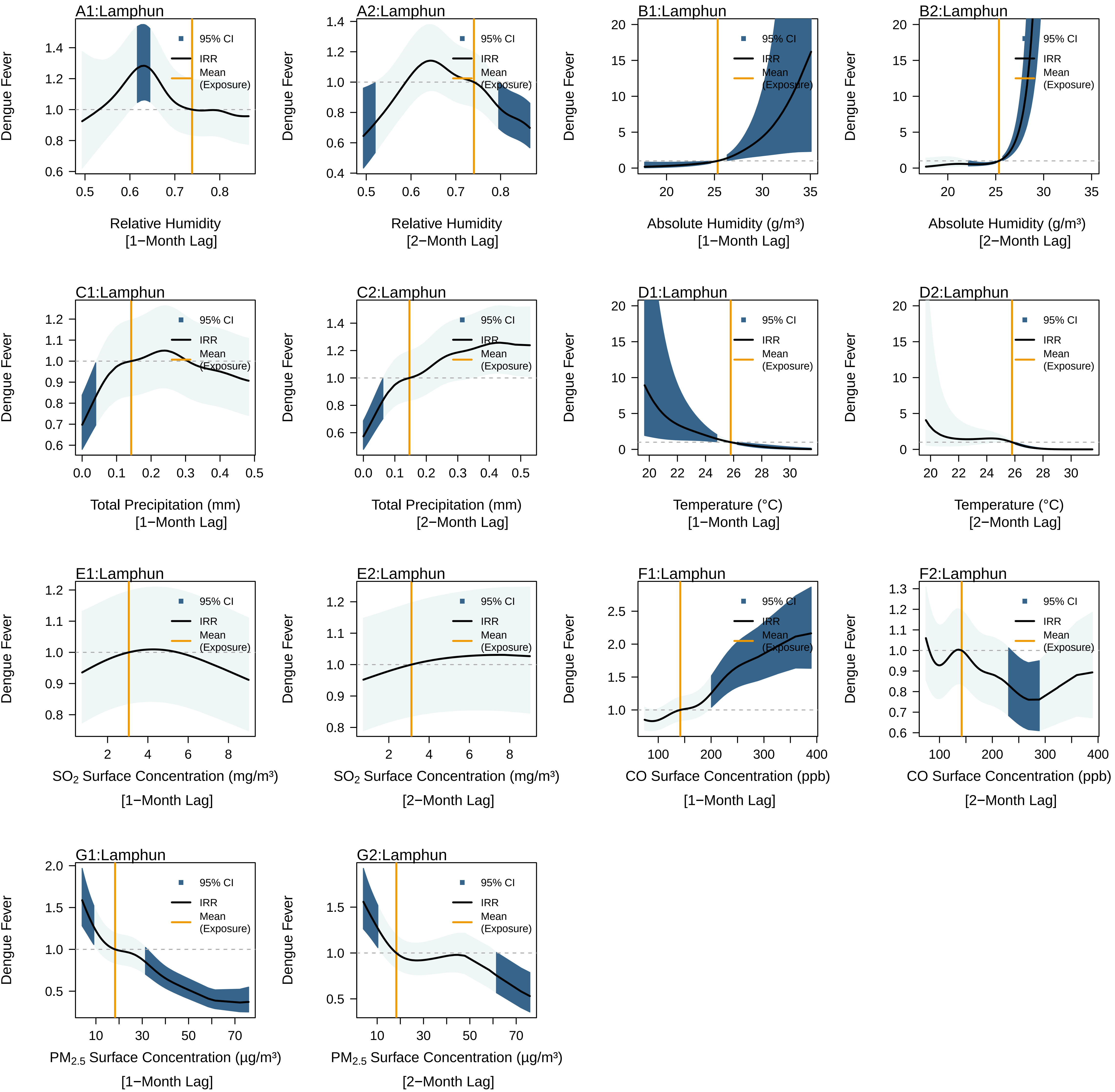
A1:Lamphun
A2:Lamphun
B1:Lamphun
B2:Lamphun
C1:Lamphun
C2:Lamphun
D1:Lamphun
D2:Lamphun
E1:Lamphun
E2:Lamphun
F1:Lamphun
F2:Lamphun
G1:Lamphun
G2:Lamphun
95% CI
IRR
Mean (Exposure)
Dengue Fever
Relative Humidity [1−Month Lag]
Relative Humidity [2−Month Lag]
Absolute Humidity (g/m³) [1−Month Lag]
Absolute Humidity (g/m³) [2−Month Lag]
Total Precipitation (mm) [1−Month Lag]
Total Precipitation (mm) [2−Month Lag]
Temperature (°C) [1−Month Lag]
Temperature (°C) [2−Month Lag]
SO_2 Surface Concentration (mg/m³) [1−Month Lag]
SO_2 Surface Concentration (mg/m³) [2−Month Lag]
CO Surface Concentration (ppb) [1−Month Lag]
CO Surface Concentration (ppb) [2−Month Lag]
$PM_{2.5}$ Surface Concentration (µg/m³) [1−Month Lag]
$PM_{2.5}$ Surface Concentration (µg/m³) [2−Month Lag]

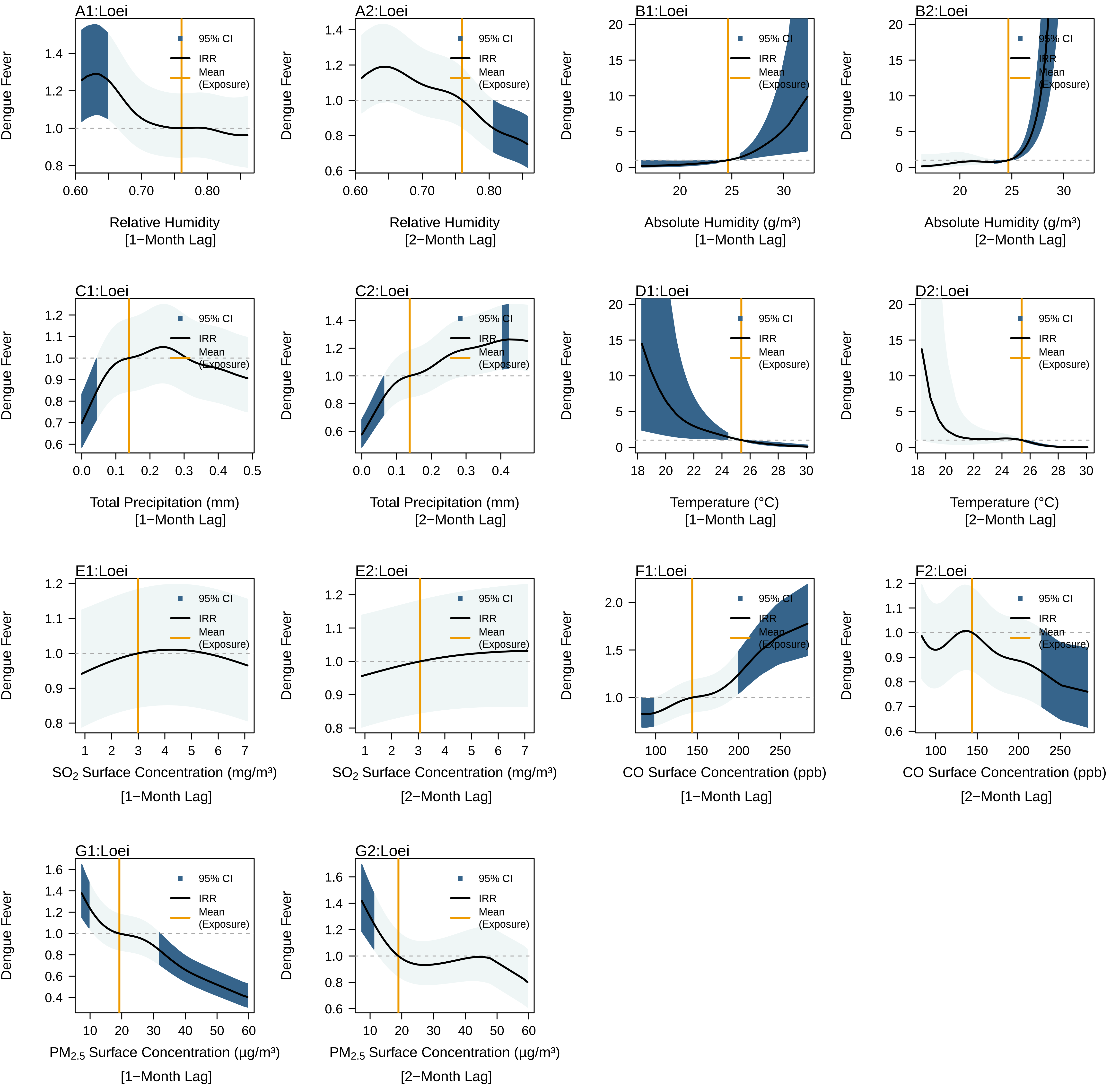
A1:Loei
A2:Loei
B1:Loei
B2:Loei
C1:Loei
C2:Loei
D1:Loei
D2:Loei
E1:Loei
E2:Loei
F1:Loei
F2:Loei
G1:Loei
G2:Loei
95% CI
IRR
Mean (Exposure)
Dengue Fever
Relative Humidity [1−Month Lag]
Relative Humidity [2−Month Lag]
Absolute Humidity (g/m³) [1−Month Lag]
Absolute Humidity (g/m³) [2−Month Lag]
Total Precipitation (mm) [1−Month Lag]
Total Precipitation (mm) [2−Month Lag]
Temperature (°C) [1−Month Lag]
Temperature (°C) [2−Month Lag]
SO2 Surface Concentration (mg/m³) [1−Month Lag]
SO2 Surface Concentration (mg/m³) [2−Month Lag]
CO Surface Concentration (ppb) [1−Month Lag]
CO Surface Concentration (ppb) [2−Month Lag]
PM2.5 Surface Concentration (µg/m³) [1−Month Lag]
PM2.5 Surface Concentration (µg/m³) [2−Month Lag]

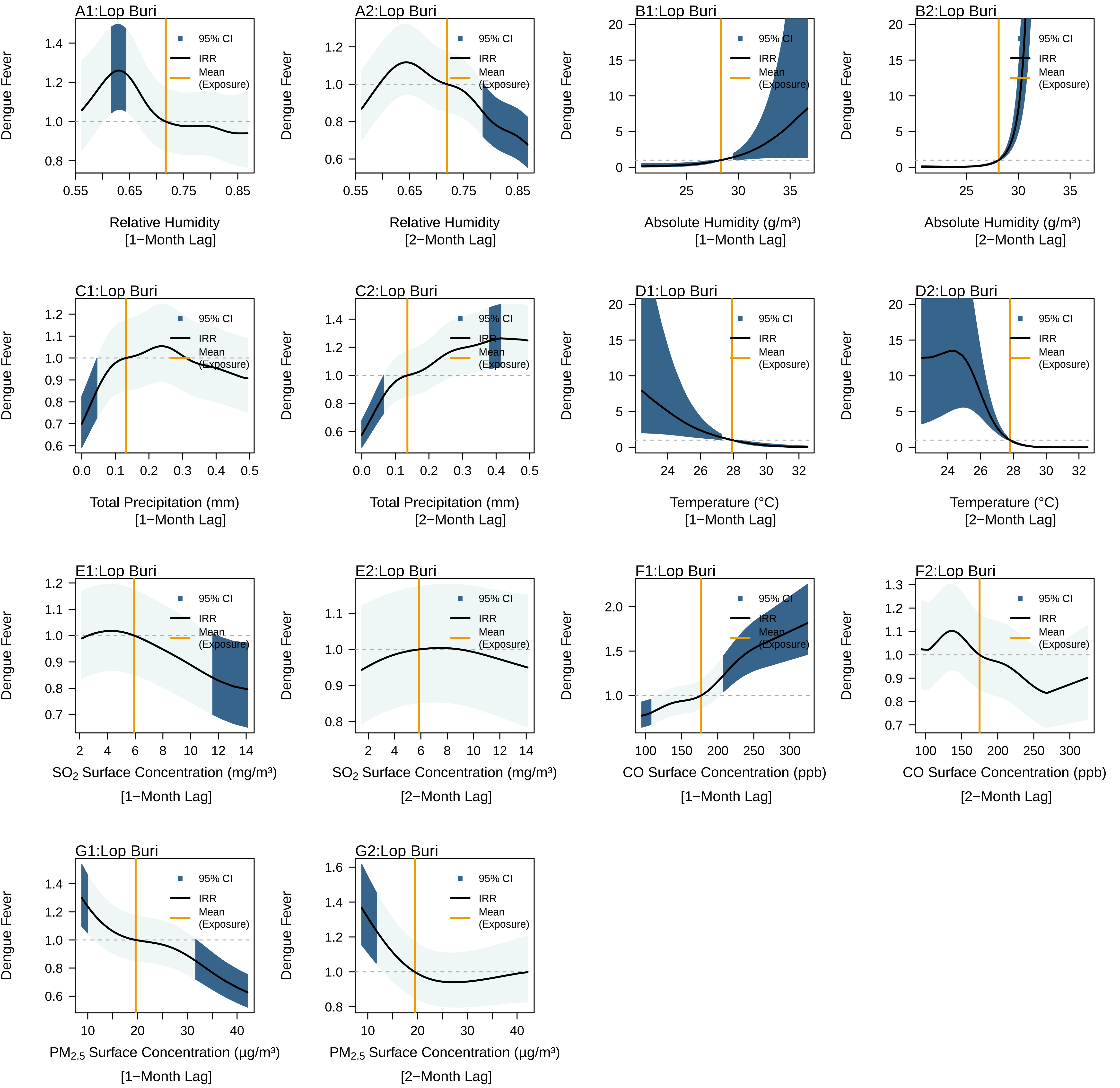

A1:Lop Buri
Dengue Fever
95% CI
IRR
Mean
(Exposure)
Relative Humidity
[1−Month Lag]
A2:Lop Buri
Relative Humidity
[2−Month Lag]
B1:Lop Buri
Absolute Humidity (g/m³)
[1−Month Lag]
B2:Lop Buri
Absolute Humidity (g/m³)
[2−Month Lag]
C1:Lop Buri
Total Precipitation (mm)
[1−Month Lag]
C2:Lop Buri
Total Precipitation (mm)
[2−Month Lag]
D1:Lop Buri
Temperature (°C)
[1−Month Lag]
D2:Lop Buri
Temperature (°C)
[2−Month Lag]
E1:Lop Buri
SO_2 Surface Concentration (mg/m³)
[1−Month Lag]
E2:Lop Buri
SO_2 Surface Concentration (mg/m³)
[2−Month Lag]
F1:Lop Buri
CO Surface Concentration (ppb)
[1−Month Lag]
F2:Lop Buri
CO Surface Concentration (ppb)
[2−Month Lag]
G1:Lop Buri
$PM_{2.5}$ Surface Concentration (μg/m³)
[1−Month Lag]
G2:Lop Buri
$PM_{2.5}$ Surface Concentration (μg/m³)
[2−Month Lag]

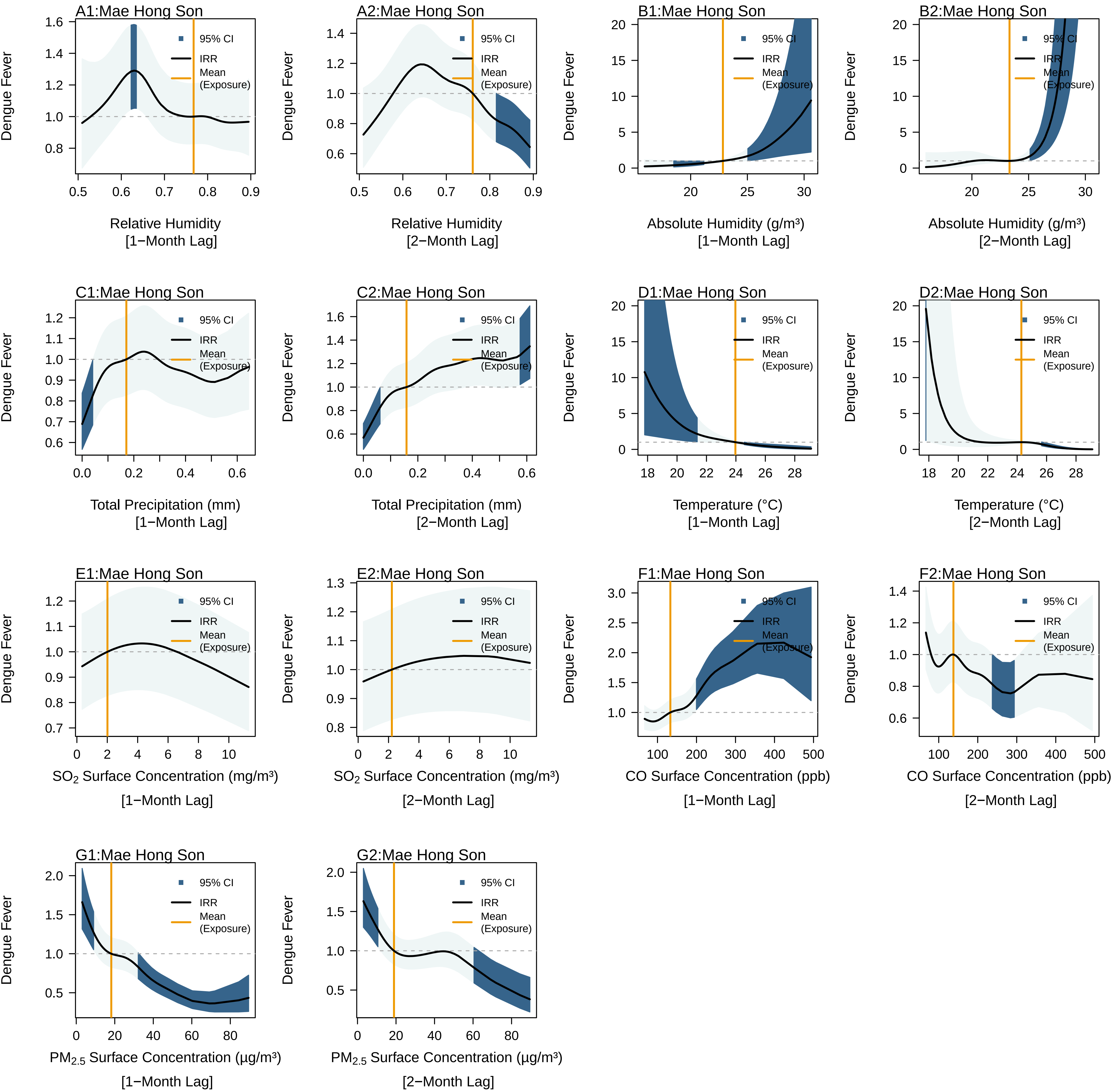
A1:Mae Hong Son
Dengue Fever
Relative Humidity
[1−Month Lag]
A2:Mae Hong Son
Dengue Fever
Relative Humidity
[2−Month Lag]
B1:Mae Hong Son
Dengue Fever
Absolute Humidity (g/m³)
[1−Month Lag]
B2:Mae Hong Son
Dengue Fever
Absolute Humidity (g/m³)
[2−Month Lag]
C1:Mae Hong Son
Dengue Fever
Total Precipitation (mm)
[1−Month Lag]
C2:Mae Hong Son
Dengue Fever
Total Precipitation (mm)
[2−Month Lag]
D1:Mae Hong Son
Dengue Fever
Temperature (°C)
[1−Month Lag]
D2:Mae Hong Son
Dengue Fever
Temperature (°C)
[2−Month Lag]
E1:Mae Hong Son
Dengue Fever
SO_2 Surface Concentration (mg/m³)
[1−Month Lag]
E2:Mae Hong Son
Dengue Fever
SO_2 Surface Concentration (mg/m³)
[2−Month Lag]
F1:Mae Hong Son
Dengue Fever
CO Surface Concentration (ppb)
[1−Month Lag]
F2:Mae Hong Son
Dengue Fever
CO Surface Concentration (ppb)
[2−Month Lag]
G1:Mae Hong Son
Dengue Fever
$PM_{2.5}$ Surface Concentration (µg/m³)
[1−Month Lag]
G2:Mae Hong Son
Dengue Fever
$PM_{2.5}$ Surface Concentration (µg/m³)
[2−Month Lag]
95% CI
IRR
Mean
(Exposure)

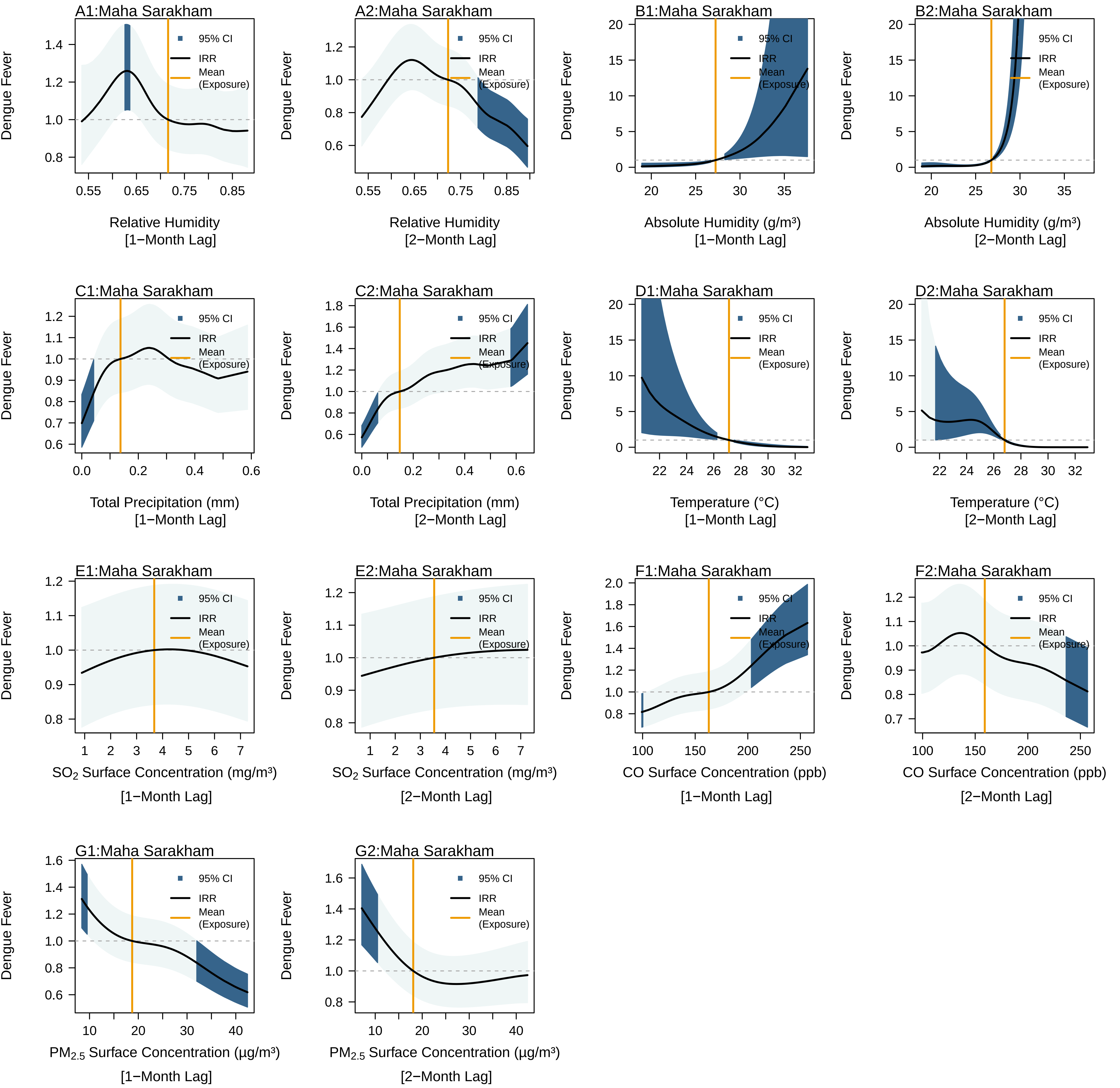
A1:Maha Sarakham
Dengue Fever
95% CI
IRR
Mean (Exposure)
Relative Humidity [1−Month Lag]
A2:Maha Sarakham
Dengue Fever
95% CI
IRR
Mean (Exposure)
Relative Humidity [2−Month Lag]
B1:Maha Sarakham
Dengue Fever
95% CI
IRR
Mean (Exposure)
Absolute Humidity (g/m³) [1−Month Lag]
B2:Maha Sarakham
Dengue Fever
95% CI
IRR
Mean (Exposure)
Absolute Humidity (g/m³) [2−Month Lag]
C1:Maha Sarakham
Dengue Fever
95% CI
IRR
Mean (Exposure)
Total Precipitation (mm) [1−Month Lag]
C2:Maha Sarakham
Dengue Fever
95% CI
IRR
Mean (Exposure)
Total Precipitation (mm) [2−Month Lag]
D1:Maha Sarakham
Dengue Fever
95% CI
IRR
Mean (Exposure)
Temperature (°C) [1−Month Lag]
D2:Maha Sarakham
Dengue Fever
95% CI
IRR
Mean (Exposure)
Temperature (°C) [2−Month Lag]
E1:Maha Sarakham
Dengue Fever
95% CI
IRR
Mean (Exposure)
SO_2 Surface Concentration (mg/m³) [1−Month Lag]
E2:Maha Sarakham
Dengue Fever
95% CI
IRR
Mean (Exposure)
SO_2 Surface Concentration (mg/m³) [2−Month Lag]
F1:Maha Sarakham
Dengue Fever
95% CI
IRR
Mean (Exposure)
CO Surface Concentration (ppb) [1−Month Lag]
F2:Maha Sarakham
Dengue Fever
95% CI
IRR
Mean (Exposure)
CO Surface Concentration (ppb) [2−Month Lag]
G1:Maha Sarakham
Dengue Fever
95% CI
IRR
Mean (Exposure)
$PM_{2.5}$ Surface Concentration (µg/m³) [1−Month Lag]
G2:Maha Sarakham
Dengue Fever
95% CI
IRR
Mean (Exposure)
$PM_{2.5}$ Surface Concentration (µg/m³) [2−Month Lag]

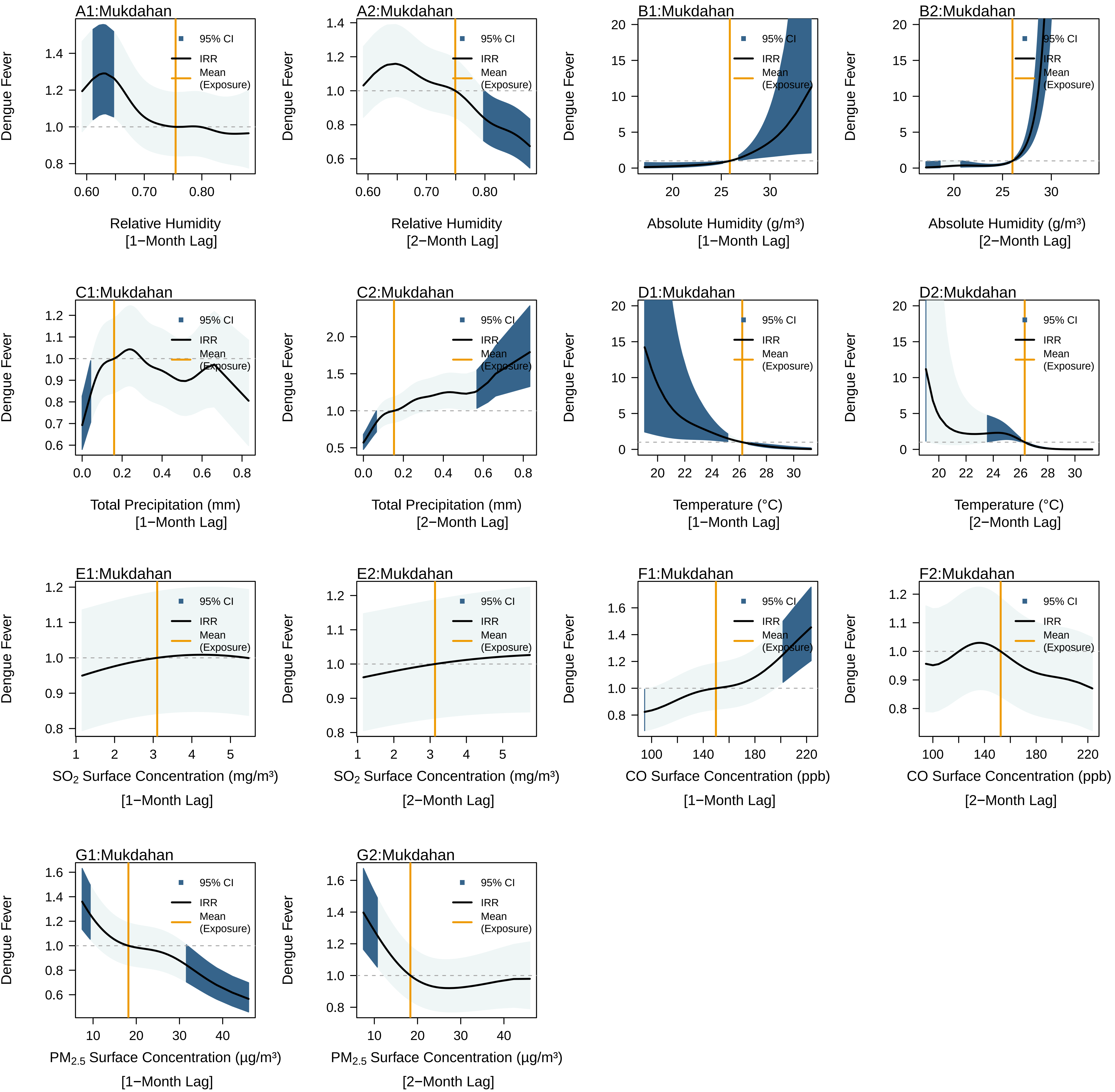
A1:Mukdahan
Dengue Fever
95% CI
IRR
Mean (Exposure)
Relative Humidity [1−Month Lag]
A2:Mukdahan
Relative Humidity [2−Month Lag]
B1:Mukdahan
Absolute Humidity (g/m³) [1−Month Lag]
B2:Mukdahan
Absolute Humidity (g/m³) [2−Month Lag]
C1:Mukdahan
Total Precipitation (mm) [1−Month Lag]
C2:Mukdahan
Total Precipitation (mm) [2−Month Lag]
D1:Mukdahan
Temperature (°C) [1−Month Lag]
D2:Mukdahan
Temperature (°C) [2−Month Lag]
E1:Mukdahan
SO2 Surface Concentration (mg/m³) [1−Month Lag]
E2:Mukdahan
SO2 Surface Concentration (mg/m³) [2−Month Lag]
F1:Mukdahan
CO Surface Concentration (ppb) [1−Month Lag]
F2:Mukdahan
CO Surface Concentration (ppb) [2−Month Lag]
G1:Mukdahan
PM2.5 Surface Concentration (µg/m³) [1−Month Lag]
G2:Mukdahan
PM2.5 Surface Concentration (µg/m³) [2−Month Lag]

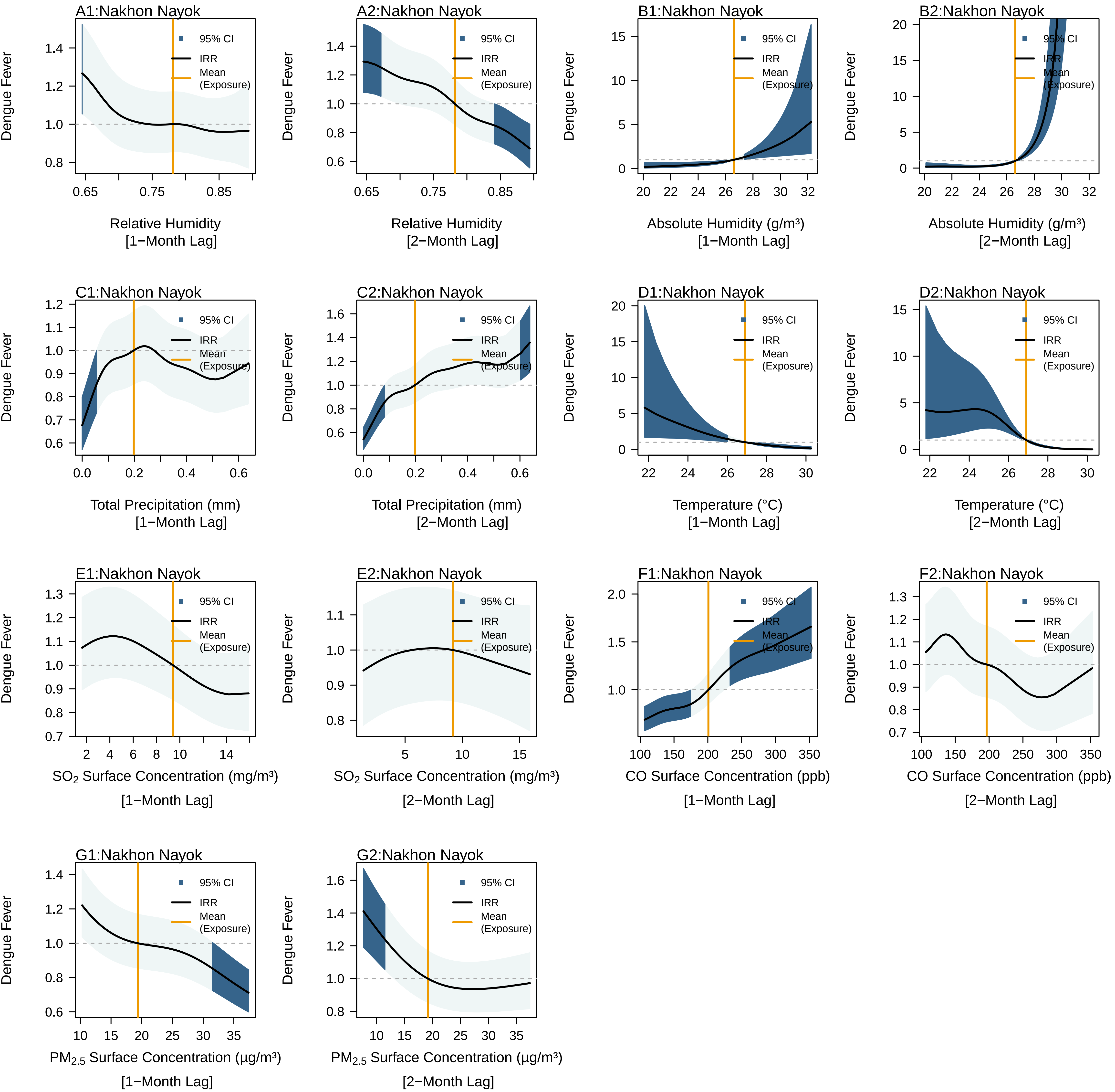
A1:Nakhon Nayok
Dengue Fever
95% CI
IRR
Mean (Exposure)
Relative Humidity [1−Month Lag]
A2:Nakhon Nayok
Relative Humidity [2−Month Lag]
B1:Nakhon Nayok
Absolute Humidity (g/m³) [1−Month Lag]
B2:Nakhon Nayok
Absolute Humidity (g/m³) [2−Month Lag]
C1:Nakhon Nayok
Total Precipitation (mm) [1−Month Lag]
C2:Nakhon Nayok
Total Precipitation (mm) [2−Month Lag]
D1:Nakhon Nayok
Temperature (°C) [1−Month Lag]
D2:Nakhon Nayok
Temperature (°C) [2−Month Lag]
E1:Nakhon Nayok
SO_2 Surface Concentration (mg/m³) [1−Month Lag]
E2:Nakhon Nayok
SO_2 Surface Concentration (mg/m³) [2−Month Lag]
F1:Nakhon Nayok
CO Surface Concentration (ppb) [1−Month Lag]
F2:Nakhon Nayok
CO Surface Concentration (ppb) [2−Month Lag]
G1:Nakhon Nayok
$PM_{2.5}$ Surface Concentration (µg/m³) [1−Month Lag]
G2:Nakhon Nayok
$PM_{2.5}$ Surface Concentration (µg/m³) [2−Month Lag]

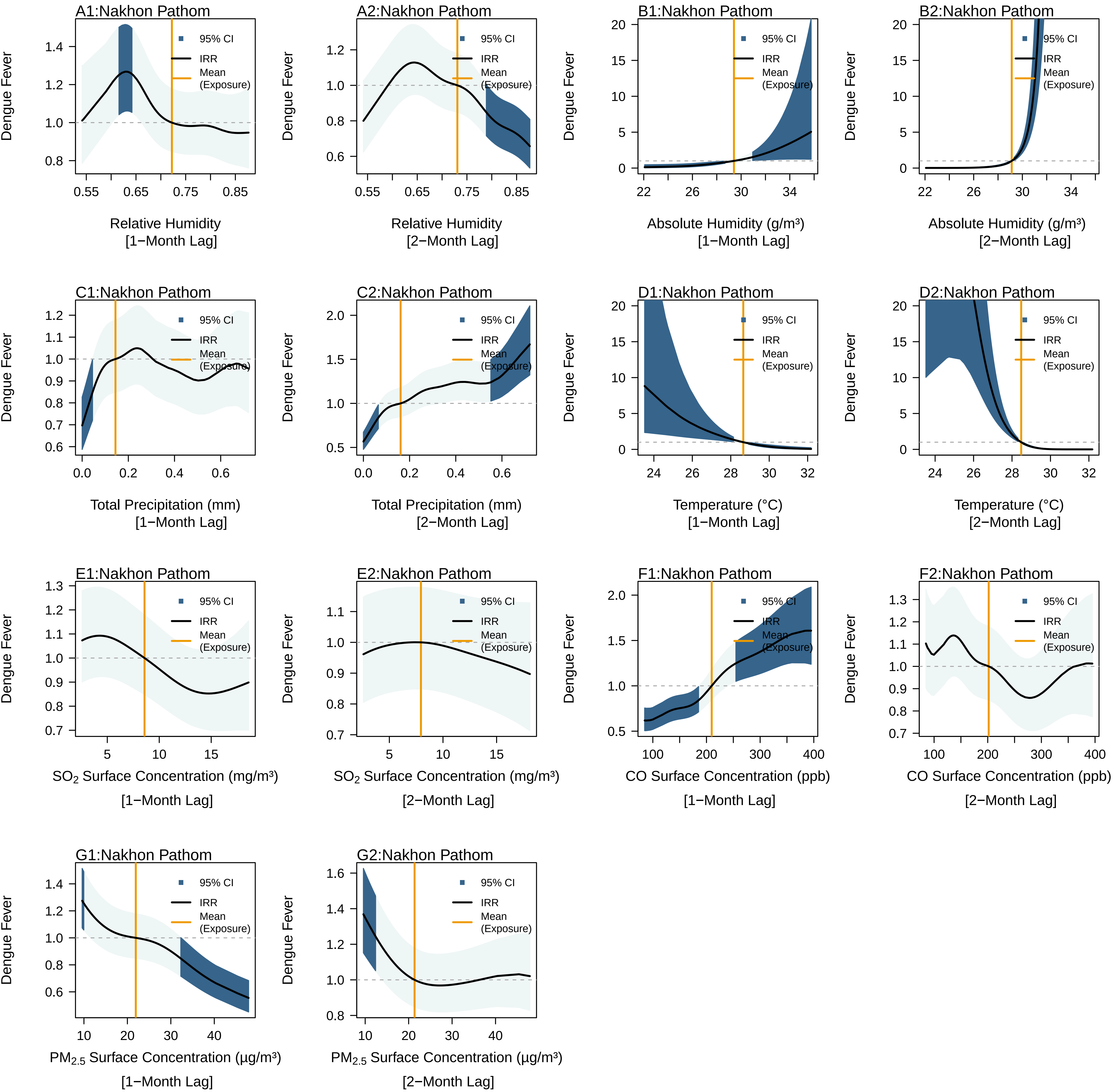
A1:Nakhon Pathom
Dengue Fever
1.4
1.2
1.0
0.8
0.55 0.65 0.75 0.85
95% CI
IRR
Mean
(Exposure)
Relative Humidity
[1−Month Lag]
A2:Nakhon Pathom
Dengue Fever
1.2
1.0
0.8
0.6
0.55 0.65 0.75 0.85
95% CI
IRR
Mean
(Exposure)
Relative Humidity
[2−Month Lag]
B1:Nakhon Pathom
Dengue Fever
20
15
10
5
0
22 26 30 34
95% CI
IRR
Mean
(Exposure)
Absolute Humidity (g/m³)
[1−Month Lag]
B2:Nakhon Pathom
Dengue Fever
20
15
10
5
0
22 26 30 34
95% CI
IRR
Mean
(Exposure)
Absolute Humidity (g/m³)
[2−Month Lag]
C1:Nakhon Pathom
Dengue Fever
1.2
1.1
1.0
0.9
0.8
0.7
0.6
0.0 0.2 0.4 0.6
95% CI
IRR
Mean
(Exposure)
Total Precipitation (mm)
[1−Month Lag]
C2:Nakhon Pathom
Dengue Fever
2.0
1.5
1.0
0.5
0.0 0.2 0.4 0.6
95% CI
IRR
Mean
(Exposure)
Total Precipitation (mm)
[2−Month Lag]
D1:Nakhon Pathom
Dengue Fever
20
15
10
5
0
24 26 28 30 32
95% CI
IRR
Mean
(Exposure)
Temperature (°C)
[1−Month Lag]
D2:Nakhon Pathom
Dengue Fever
20
15
10
5
0
24 26 28 30 32
95% CI
IRR
Mean
(Exposure)
Temperature (°C)
[2−Month Lag]
E1:Nakhon Pathom
Dengue Fever
1.3
1.2
1.1
1.0
0.9
0.8
0.7
5 10 15
95% CI
IRR
Mean
(Exposure)
SO2 Surface Concentration (mg/m³)
[1−Month Lag]
E2:Nakhon Pathom
Dengue Fever
1.1
1.0
0.9
0.8
0.7
5 10 15
95% CI
IRR
Mean
(Exposure)
SO2 Surface Concentration (mg/m³)
[2−Month Lag]
F1:Nakhon Pathom
Dengue Fever
2.0
1.5
1.0
0.5
100 200 300 400
95% CI
IRR
Mean
(Exposure)
CO Surface Concentration (ppb)
[1−Month Lag]
F2:Nakhon Pathom
Dengue Fever
1.3
1.2
1.1
1.0
0.9
0.8
0.7
100 200 300 400
95% CI
IRR
Mean
(Exposure)
CO Surface Concentration (ppb)
[2−Month Lag]
G1:Nakhon Pathom
Dengue Fever
1.4
1.2
1.0
0.8
0.6
10 20 30 40
95% CI
IRR
Mean
(Exposure)
PM2.5 Surface Concentration (µg/m³)
[1−Month Lag]
G2:Nakhon Pathom
Dengue Fever
1.6
1.4
1.2
1.0
0.8
10 20 30 40
95% CI
IRR
Mean
(Exposure)
PM2.5 Surface Concentration (µg/m³)
[2−Month Lag]

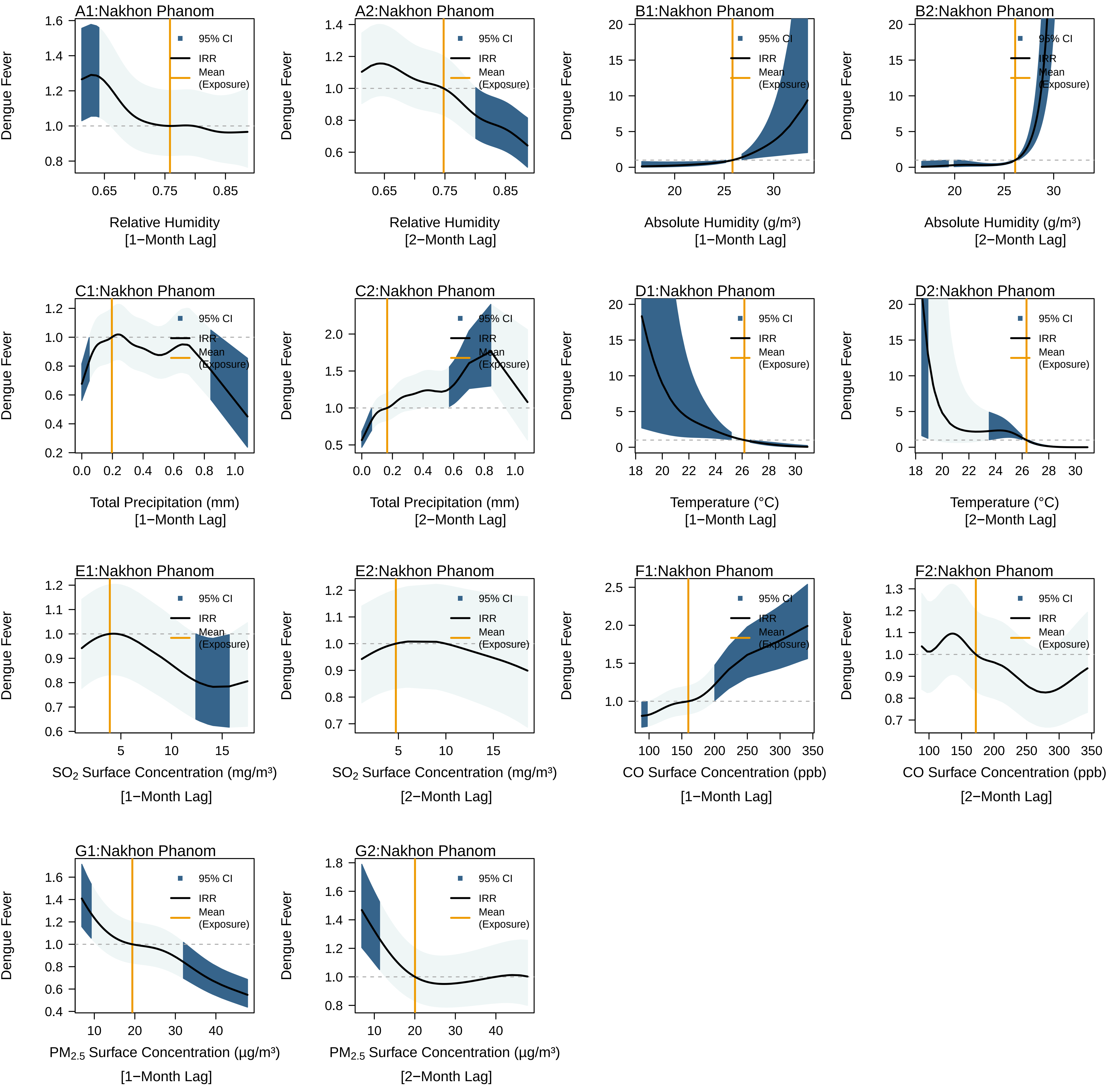

A1:Nakhon Phanom
A2:Nakhon Phanom
B1:Nakhon Phanom
B2:Nakhon Phanom
C1:Nakhon Phanom
C2:Nakhon Phanom
D1:Nakhon Phanom
D2:Nakhon Phanom
E1:Nakhon Phanom
E2:Nakhon Phanom
F1:Nakhon Phanom
F2:Nakhon Phanom
G1:Nakhon Phanom
G2:Nakhon Phanom
Dengue Fever
95% CI
IRR
Mean (Exposure)
Relative Humidity [1−Month Lag]
Relative Humidity [2−Month Lag]
Absolute Humidity (g/m³) [1−Month Lag]
Absolute Humidity (g/m³) [2−Month Lag]
Total Precipitation (mm) [1−Month Lag]
Total Precipitation (mm) [2−Month Lag]
Temperature (°C) [1−Month Lag]
Temperature (°C) [2−Month Lag]
SO_2 Surface Concentration (mg/m³) [1−Month Lag]
SO_2 Surface Concentration (mg/m³) [2−Month Lag]
CO Surface Concentration (ppb) [1−Month Lag]
CO Surface Concentration (ppb) [2−Month Lag]
$PM_{2.5}$ Surface Concentration (µg/m³) [1−Month Lag]
$PM_{2.5}$ Surface Concentration (µg/m³) [2−Month Lag]

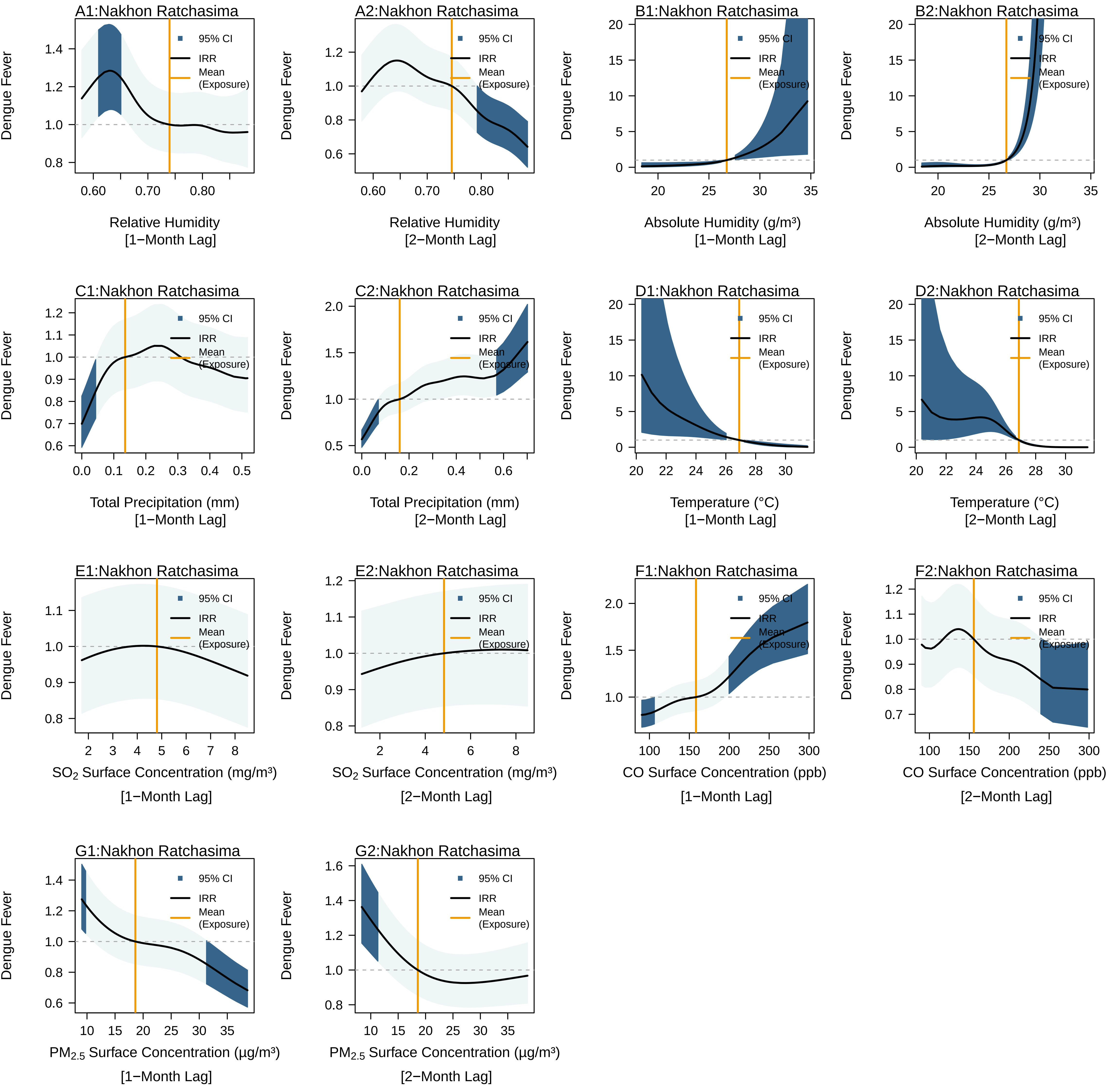

A1:Nakhon Ratchasima
A2:Nakhon Ratchasima
B1:Nakhon Ratchasima
B2:Nakhon Ratchasima
C1:Nakhon Ratchasima
C2:Nakhon Ratchasima
D1:Nakhon Ratchasima
D2:Nakhon Ratchasima
E1:Nakhon Ratchasima
E2:Nakhon Ratchasima
F1:Nakhon Ratchasima
F2:Nakhon Ratchasima
G1:Nakhon Ratchasima
G2:Nakhon Ratchasima
Dengue Fever
95% CI
IRR
Mean (Exposure)
Relative Humidity [1−Month Lag]
Relative Humidity [2−Month Lag]
Absolute Humidity (g/m³) [1−Month Lag]
Absolute Humidity (g/m³) [2−Month Lag]
Total Precipitation (mm) [1−Month Lag]
Total Precipitation (mm) [2−Month Lag]
Temperature (°C) [1−Month Lag]
Temperature (°C) [2−Month Lag]
SO_2 Surface Concentration (mg/m³) [1−Month Lag]
SO_2 Surface Concentration (mg/m³) [2−Month Lag]
CO Surface Concentration (ppb) [1−Month Lag]
CO Surface Concentration (ppb) [2−Month Lag]
$PM_{2.5}$ Surface Concentration (µg/m³) [1−Month Lag]
$PM_{2.5}$ Surface Concentration (µg/m³) [2−Month Lag]

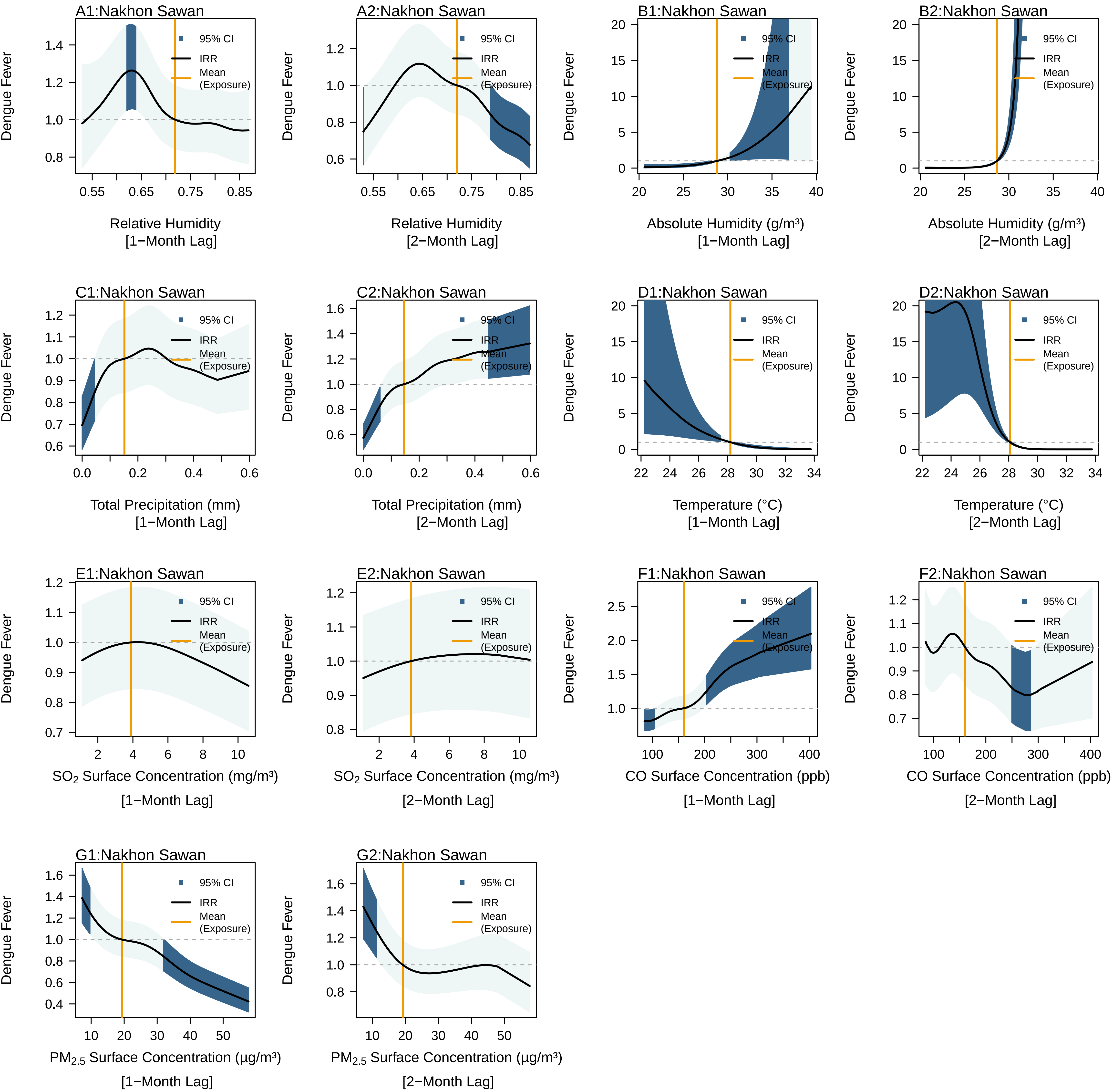
A1:Nakhon Sawan
Dengue Fever
95% CI
IRR
Mean (Exposure)
Relative Humidity [1−Month Lag]
A2:Nakhon Sawan
Relative Humidity [2−Month Lag]
B1:Nakhon Sawan
Absolute Humidity (g/m³) [1−Month Lag]
B2:Nakhon Sawan
Absolute Humidity (g/m³) [2−Month Lag]
C1:Nakhon Sawan
Total Precipitation (mm) [1−Month Lag]
C2:Nakhon Sawan
Total Precipitation (mm) [2−Month Lag]
D1:Nakhon Sawan
Temperature (°C) [1−Month Lag]
D2:Nakhon Sawan
Temperature (°C) [2−Month Lag]
E1:Nakhon Sawan
SO_2 Surface Concentration (mg/m³) [1−Month Lag]
E2:Nakhon Sawan
SO_2 Surface Concentration (mg/m³) [2−Month Lag]
F1:Nakhon Sawan
CO Surface Concentration (ppb) [1−Month Lag]
F2:Nakhon Sawan
CO Surface Concentration (ppb) [2−Month Lag]
G1:Nakhon Sawan
$PM_{2.5}$ Surface Concentration (µg/m³) [1−Month Lag]
G2:Nakhon Sawan
$PM_{2.5}$ Surface Concentration (µg/m³) [2−Month Lag]

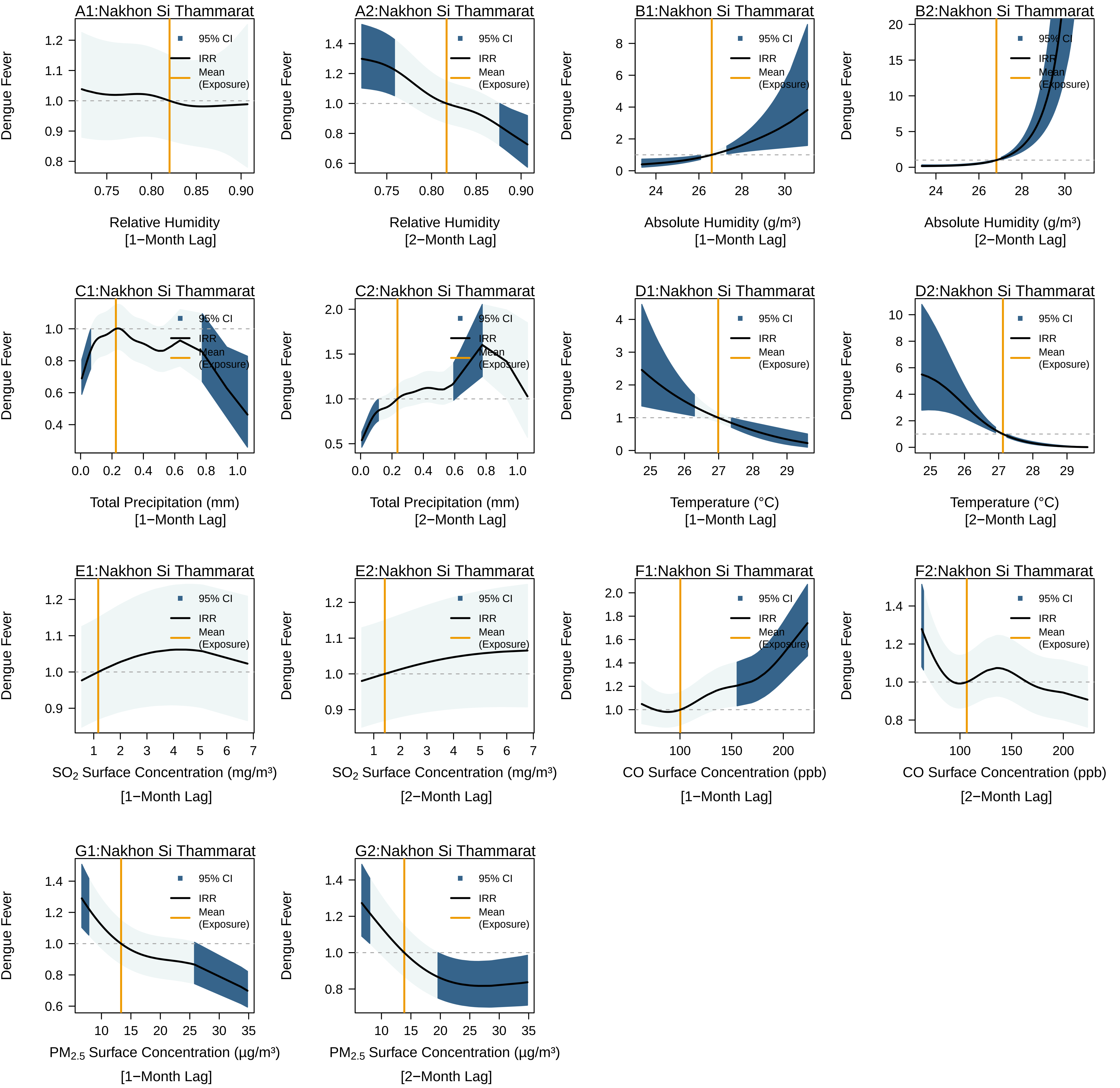

A1:Nakhon Si Thammarat
Relative Humidity [1−Month Lag]
A2:Nakhon Si Thammarat
Relative Humidity [2−Month Lag]
B1:Nakhon Si Thammarat
Absolute Humidity (g/m³) [1−Month Lag]
B2:Nakhon Si Thammarat
Absolute Humidity (g/m³) [2−Month Lag]
C1:Nakhon Si Thammarat
Total Precipitation (mm) [1−Month Lag]
C2:Nakhon Si Thammarat
Total Precipitation (mm) [2−Month Lag]
D1:Nakhon Si Thammarat
Temperature (°C) [1−Month Lag]
D2:Nakhon Si Thammarat
Temperature (°C) [2−Month Lag]
E1:Nakhon Si Thammarat
SO_2 Surface Concentration (mg/m³) [1−Month Lag]
E2:Nakhon Si Thammarat
SO_2 Surface Concentration (mg/m³) [2−Month Lag]
F1:Nakhon Si Thammarat
CO Surface Concentration (ppb) [1−Month Lag]
F2:Nakhon Si Thammarat
CO Surface Concentration (ppb) [2−Month Lag]
G1:Nakhon Si Thammarat
$PM_{2.5}$ Surface Concentration (µg/m³) [1−Month Lag]
G2:Nakhon Si Thammarat
$PM_{2.5}$ Surface Concentration (µg/m³) [2−Month Lag]
Dengue Fever
95% CI
IRR
Mean (Exposure)

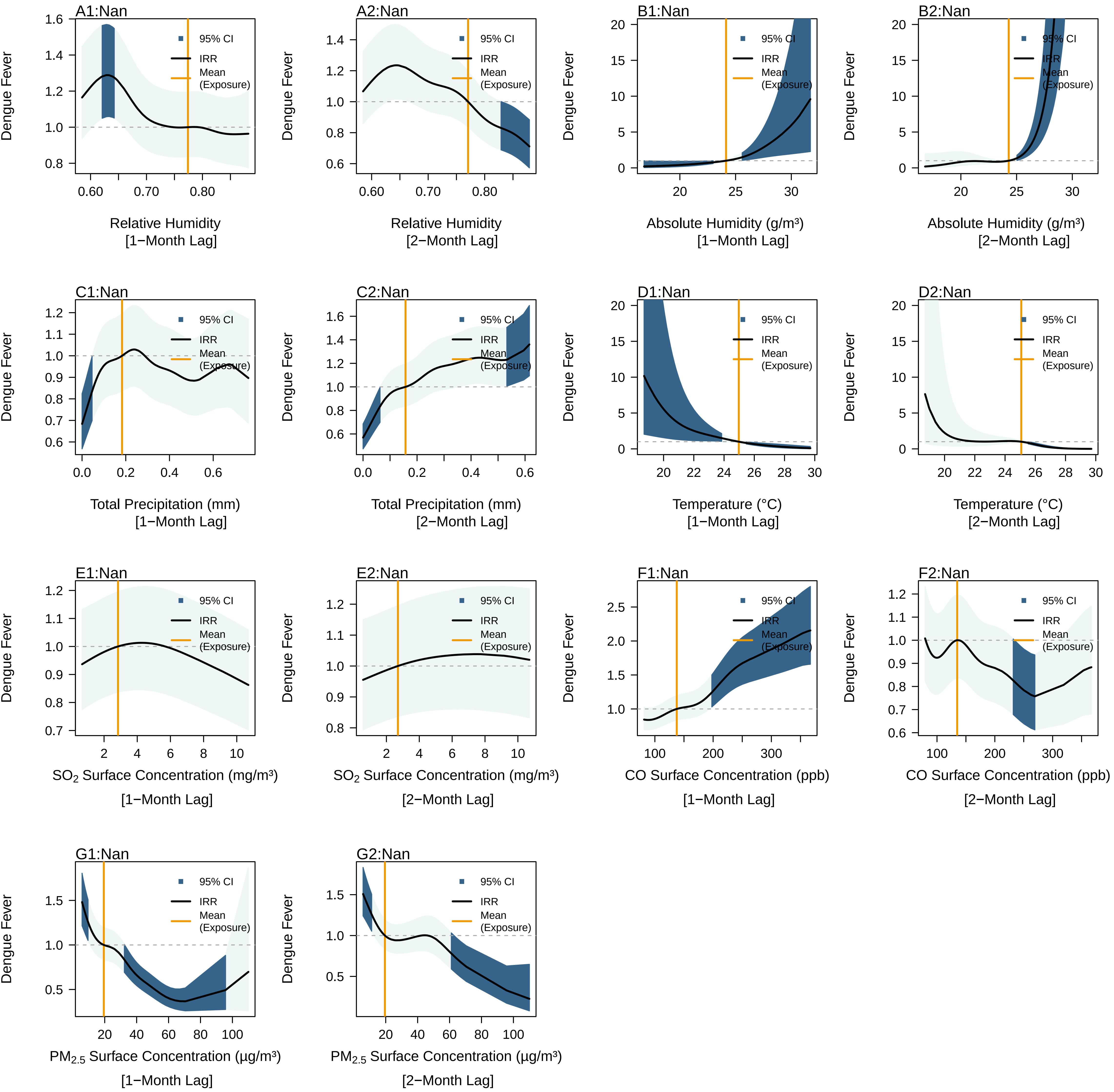

A1:Nan
A2:Nan
B1:Nan
B2:Nan
C1:Nan
C2:Nan
D1:Nan
D2:Nan
E1:Nan
E2:Nan
F1:Nan
F2:Nan
G1:Nan
G2:Nan
Dengue Fever
95% CI
IRR
Mean (Exposure)
Relative Humidity [1−Month Lag]
Relative Humidity [2−Month Lag]
Absolute Humidity (g/m³) [1−Month Lag]
Absolute Humidity (g/m³) [2−Month Lag]
Total Precipitation (mm) [1−Month Lag]
Total Precipitation (mm) [2−Month Lag]
Temperature (°C) [1−Month Lag]
Temperature (°C) [2−Month Lag]
SO_2 Surface Concentration (mg/m³) [1−Month Lag]
SO_2 Surface Concentration (mg/m³) [2−Month Lag]
CO Surface Concentration (ppb) [1−Month Lag]
CO Surface Concentration (ppb) [2−Month Lag]
$PM_{2.5}$ Surface Concentration (µg/m³) [1−Month Lag]
$PM_{2.5}$ Surface Concentration (µg/m³) [2−Month Lag]

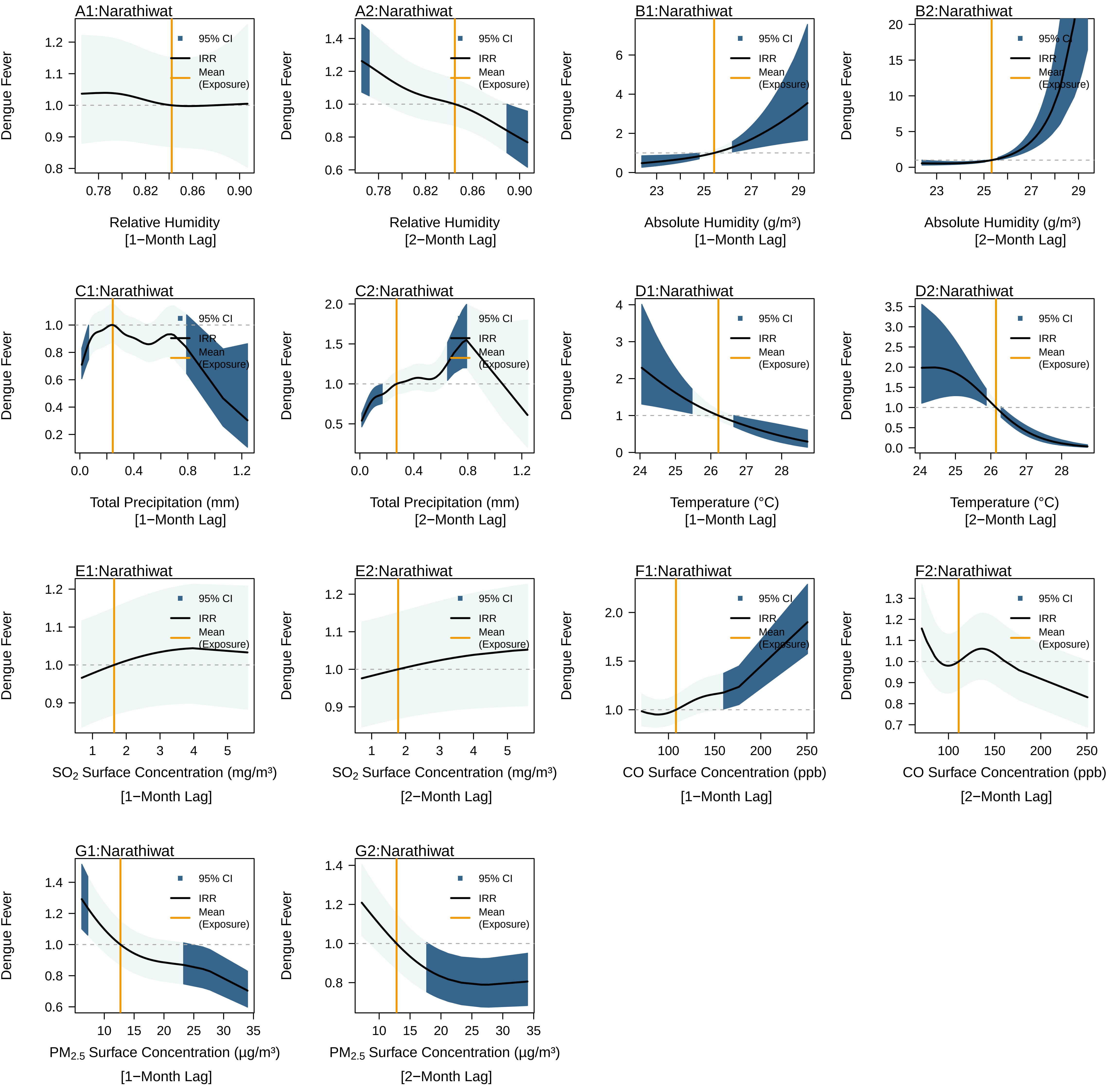

A1:Narathiwat
A2:Narathiwat
B1:Narathiwat
B2:Narathiwat
C1:Narathiwat
C2:Narathiwat
D1:Narathiwat
D2:Narathiwat
E1:Narathiwat
E2:Narathiwat
F1:Narathiwat
F2:Narathiwat
G1:Narathiwat
G2:Narathiwat
Dengue Fever
95% CI
IRR
Mean (Exposure)
Relative Humidity [1−Month Lag]
Relative Humidity [2−Month Lag]
Absolute Humidity (g/m³) [1−Month Lag]
Absolute Humidity (g/m³) [2−Month Lag]
Total Precipitation (mm) [1−Month Lag]
Total Precipitation (mm) [2−Month Lag]
Temperature (°C) [1−Month Lag]
Temperature (°C) [2−Month Lag]
SO2 Surface Concentration (mg/m³) [1−Month Lag]
SO2 Surface Concentration (mg/m³) [2−Month Lag]
CO Surface Concentration (ppb) [1−Month Lag]
CO Surface Concentration (ppb) [2−Month Lag]
PM2.5 Surface Concentration (µg/m³) [1−Month Lag]
PM2.5 Surface Concentration (µg/m³) [2−Month Lag]

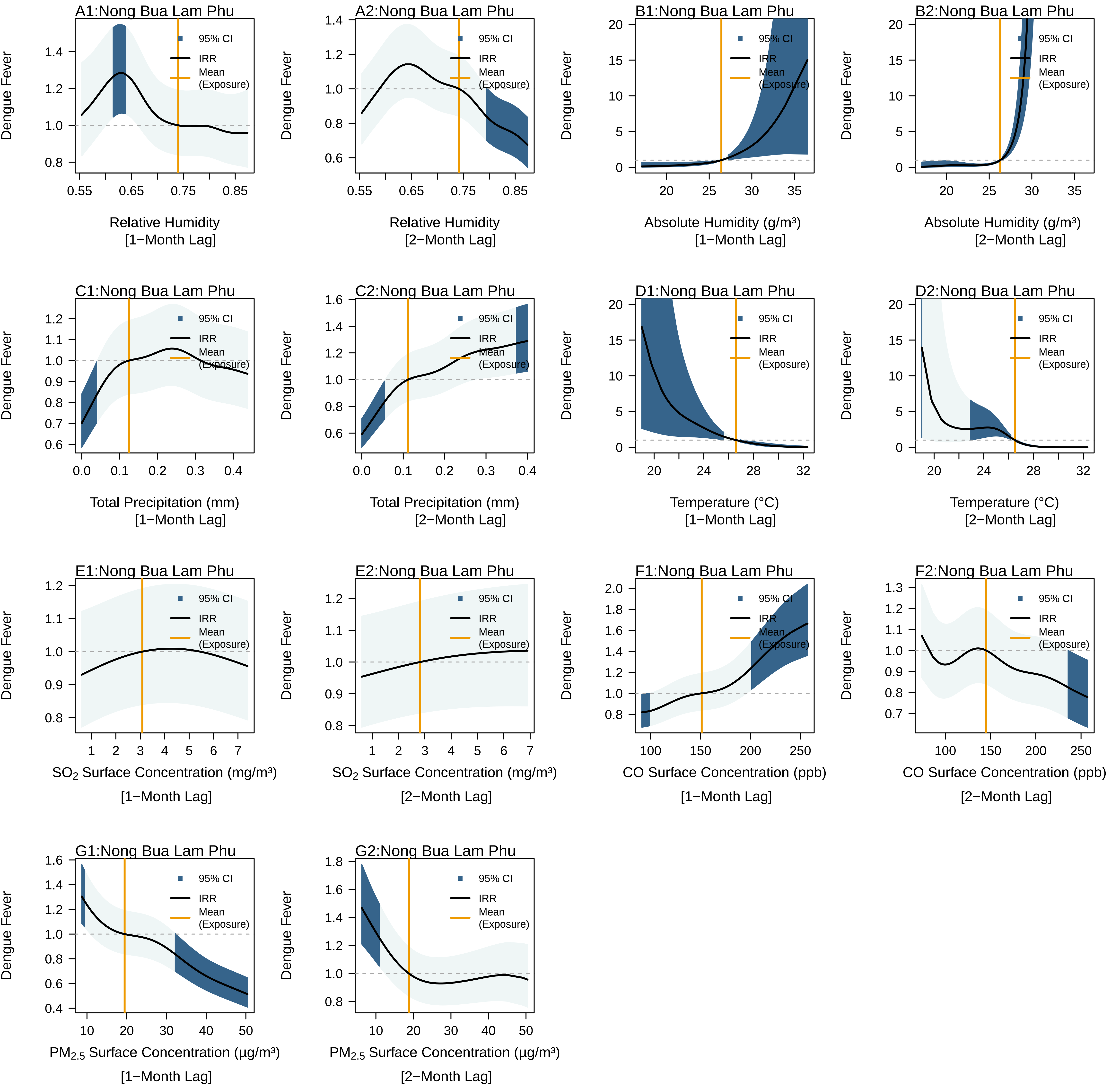
A1:Nong Bua Lam Phu
Dengue Fever
95% CI
IRR
Mean (Exposure)
Relative Humidity [1−Month Lag]
A2:Nong Bua Lam Phu
Relative Humidity [2−Month Lag]
B1:Nong Bua Lam Phu
Absolute Humidity (g/m³) [1−Month Lag]
B2:Nong Bua Lam Phu
Absolute Humidity (g/m³) [2−Month Lag]
C1:Nong Bua Lam Phu
Total Precipitation (mm) [1−Month Lag]
C2:Nong Bua Lam Phu
Total Precipitation (mm) [2−Month Lag]
D1:Nong Bua Lam Phu
Temperature (°C) [1−Month Lag]
D2:Nong Bua Lam Phu
Temperature (°C) [2−Month Lag]
E1:Nong Bua Lam Phu
SO_2 Surface Concentration (mg/m³) [1−Month Lag]
E2:Nong Bua Lam Phu
SO_2 Surface Concentration (mg/m³) [2−Month Lag]
F1:Nong Bua Lam Phu
CO Surface Concentration (ppb) [1−Month Lag]
F2:Nong Bua Lam Phu
CO Surface Concentration (ppb) [2−Month Lag]
G1:Nong Bua Lam Phu
$PM_{2.5}$ Surface Concentration (µg/m³) [1−Month Lag]
G2:Nong Bua Lam Phu
$PM_{2.5}$ Surface Concentration (µg/m³) [2−Month Lag]

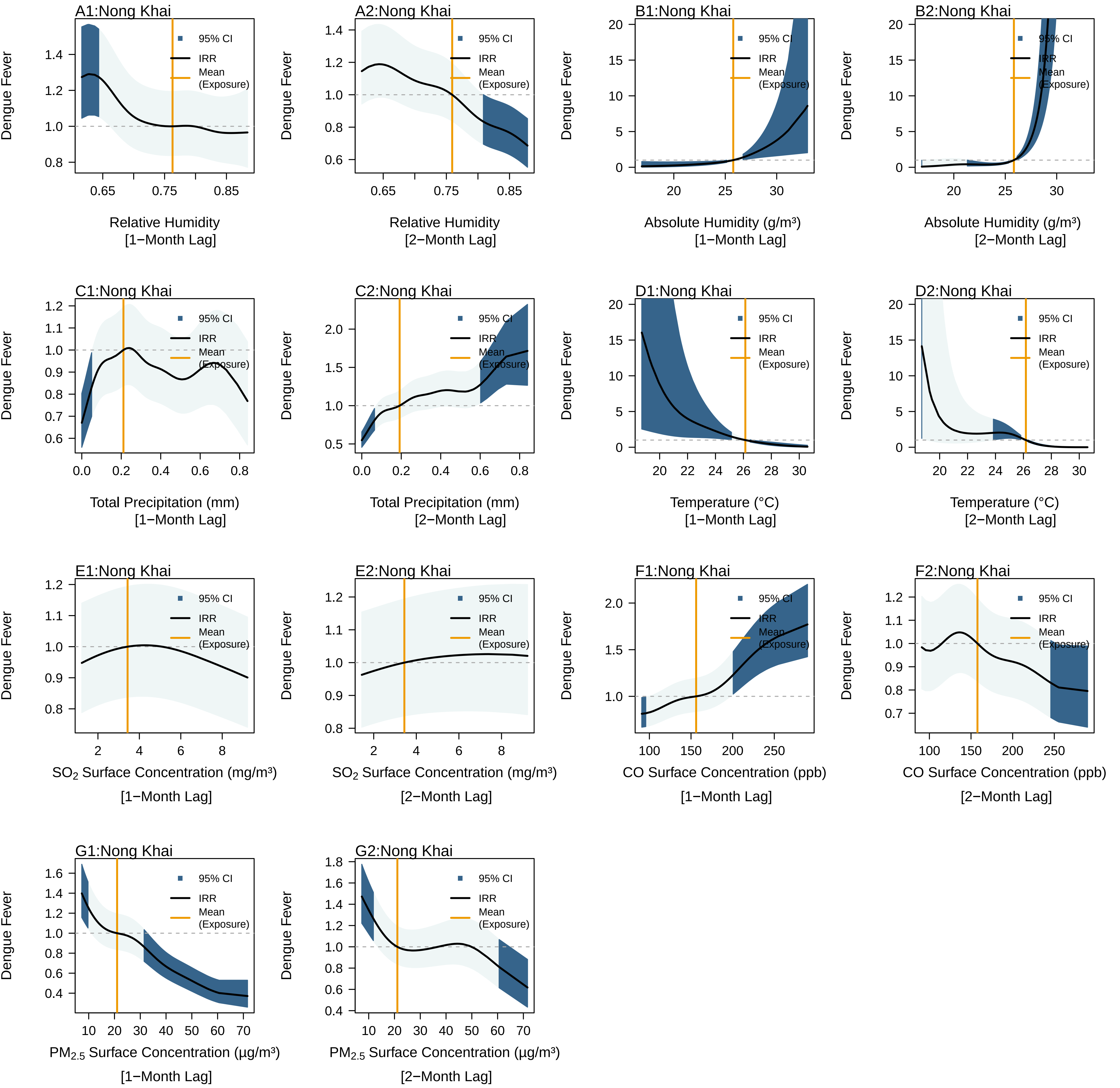

A1:Nong Khai
A2:Nong Khai
B1:Nong Khai
B2:Nong Khai
C1:Nong Khai
C2:Nong Khai
D1:Nong Khai
D2:Nong Khai
E1:Nong Khai
E2:Nong Khai
F1:Nong Khai
F2:Nong Khai
G1:Nong Khai
G2:Nong Khai
Dengue Fever
95% CI
IRR
Mean (Exposure)
Relative Humidity [1−Month Lag]
Relative Humidity [2−Month Lag]
Absolute Humidity (g/m³) [1−Month Lag]
Absolute Humidity (g/m³) [2−Month Lag]
Total Precipitation (mm) [1−Month Lag]
Total Precipitation (mm) [2−Month Lag]
Temperature (°C) [1−Month Lag]
Temperature (°C) [2−Month Lag]
SO2 Surface Concentration (mg/m³) [1−Month Lag]
SO2 Surface Concentration (mg/m³) [2−Month Lag]
CO Surface Concentration (ppb) [1−Month Lag]
CO Surface Concentration (ppb) [2−Month Lag]
PM2.5 Surface Concentration (µg/m³) [1−Month Lag]
PM2.5 Surface Concentration (µg/m³) [2−Month Lag]

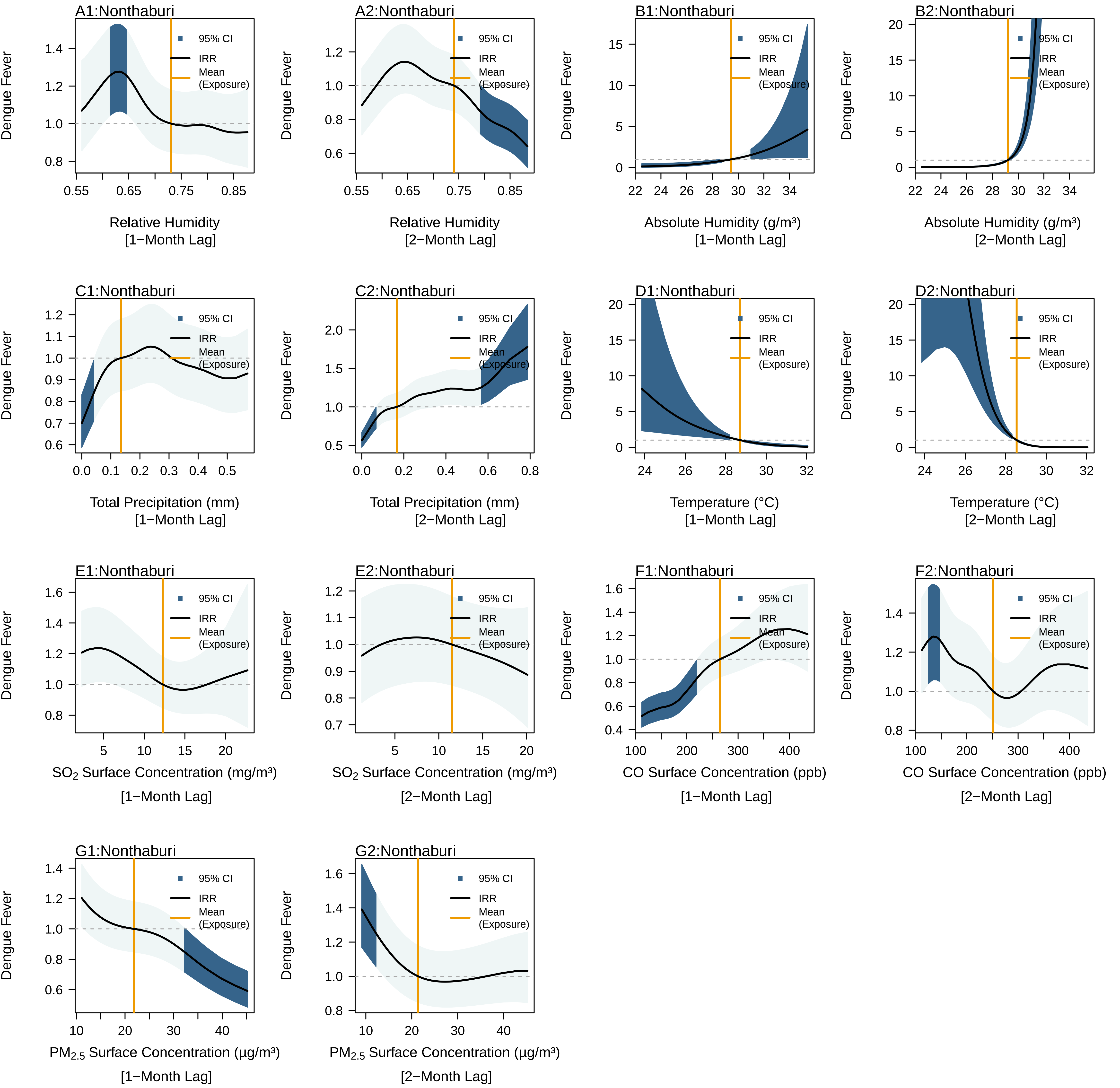

A1:Nonthaburi
A2:Nonthaburi
B1:Nonthaburi
B2:Nonthaburi
C1:Nonthaburi
C2:Nonthaburi
D1:Nonthaburi
D2:Nonthaburi
E1:Nonthaburi
E2:Nonthaburi
F1:Nonthaburi
F2:Nonthaburi
G1:Nonthaburi
G2:Nonthaburi
95% CI
IRR
Mean (Exposure)
Dengue Fever
Relative Humidity [1−Month Lag]
Relative Humidity [2−Month Lag]
Absolute Humidity (g/m³) [1−Month Lag]
Absolute Humidity (g/m³) [2−Month Lag]
Total Precipitation (mm) [1−Month Lag]
Total Precipitation (mm) [2−Month Lag]
Temperature (°C) [1−Month Lag]
Temperature (°C) [2−Month Lag]
SO2 Surface Concentration (mg/m³) [1−Month Lag]
SO2 Surface Concentration (mg/m³) [2−Month Lag]
CO Surface Concentration (ppb) [1−Month Lag]
CO Surface Concentration (ppb) [2−Month Lag]
PM2.5 Surface Concentration (µg/m³) [1−Month Lag]
PM2.5 Surface Concentration (µg/m³) [2−Month Lag]

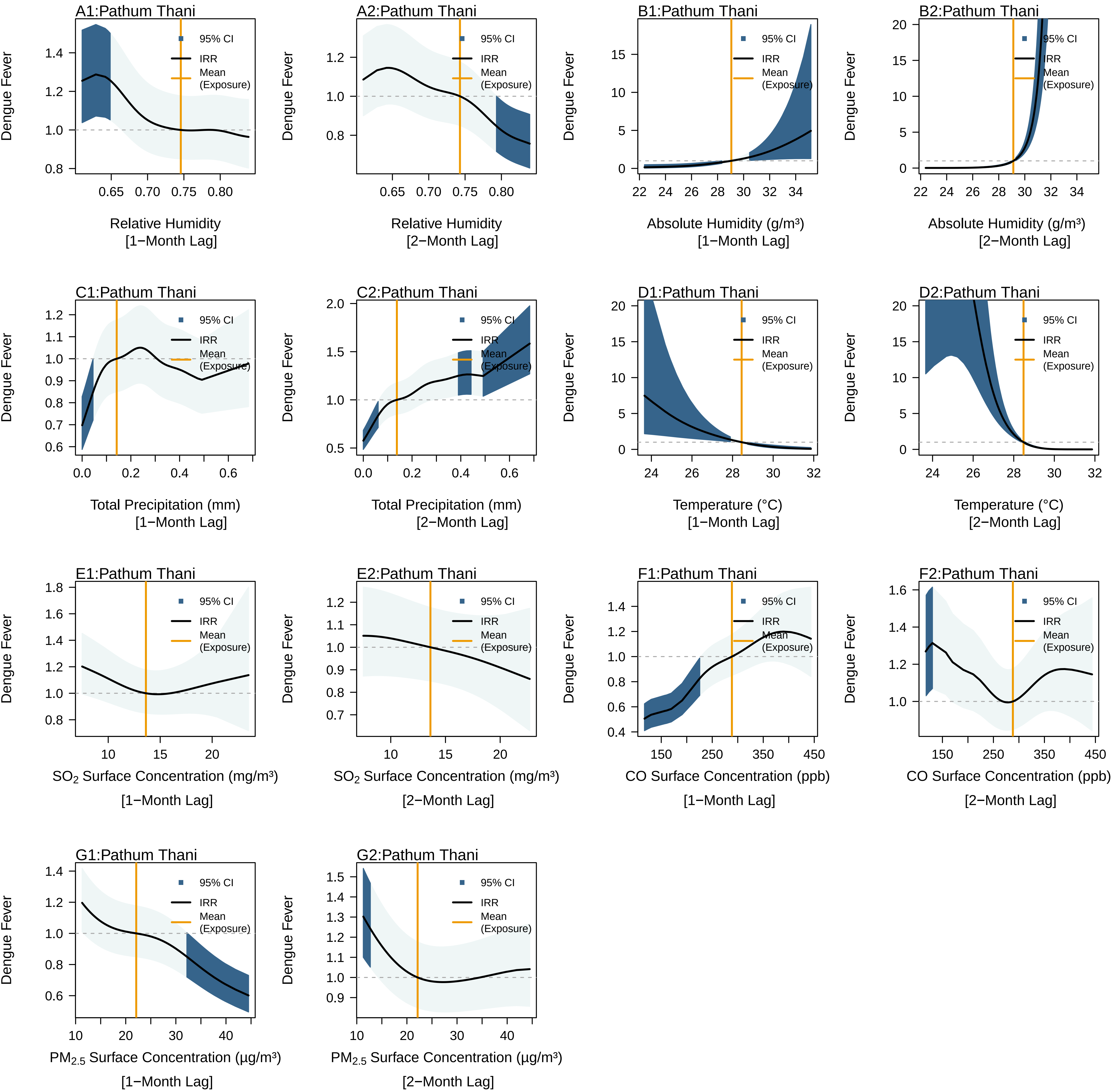
A1:Pathum Thani
A2:Pathum Thani
B1:Pathum Thani
B2:Pathum Thani
C1:Pathum Thani
C2:Pathum Thani
D1:Pathum Thani
D2:Pathum Thani
E1:Pathum Thani
E2:Pathum Thani
F1:Pathum Thani
F2:Pathum Thani
G1:Pathum Thani
G2:Pathum Thani
Dengue Fever
95% CI
IRR
Mean (Exposure)
Relative Humidity [1−Month Lag]
Relative Humidity [2−Month Lag]
Absolute Humidity (g/m³) [1−Month Lag]
Absolute Humidity (g/m³) [2−Month Lag]
Total Precipitation (mm) [1−Month Lag]
Total Precipitation (mm) [2−Month Lag]
Temperature (°C) [1−Month Lag]
Temperature (°C) [2−Month Lag]
SO2 Surface Concentration (mg/m³) [1−Month Lag]
SO2 Surface Concentration (mg/m³) [2−Month Lag]
CO Surface Concentration (ppb) [1−Month Lag]
CO Surface Concentration (ppb) [2−Month Lag]
PM2.5 Surface Concentration (µg/m³) [1−Month Lag]
PM2.5 Surface Concentration (µg/m³) [2−Month Lag]

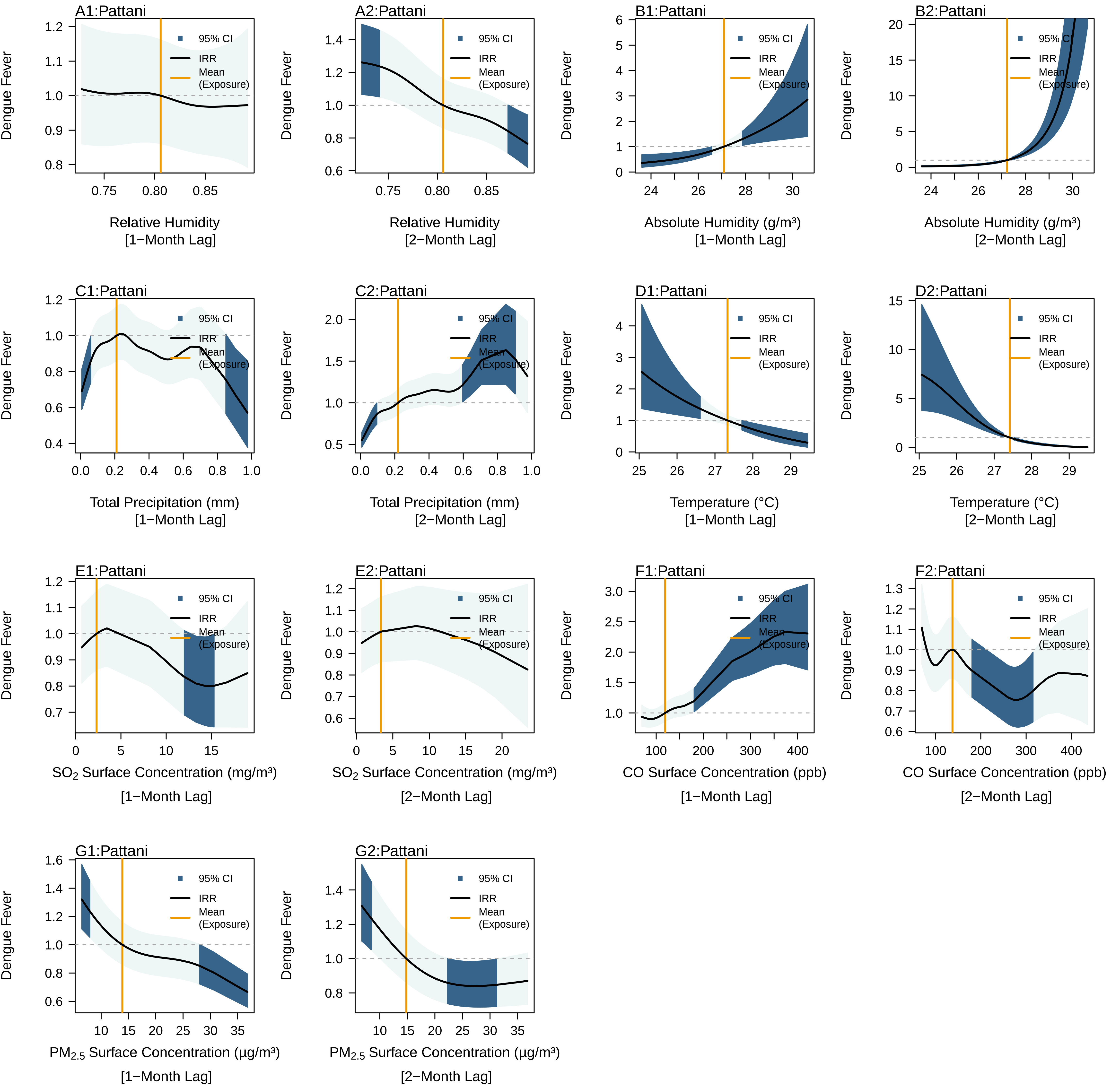
A1:Pattani
95% CI
IRR
Mean (Exposure)
Dengue Fever
Relative Humidity [1−Month Lag]
A2:Pattani
Relative Humidity [2−Month Lag]
B1:Pattani
Absolute Humidity (g/m³) [1−Month Lag]
B2:Pattani
Absolute Humidity (g/m³) [2−Month Lag]
C1:Pattani
Total Precipitation (mm) [1−Month Lag]
C2:Pattani
Total Precipitation (mm) [2−Month Lag]
D1:Pattani
Temperature (°C) [1−Month Lag]
D2:Pattani
Temperature (°C) [2−Month Lag]
E1:Pattani
SO2 Surface Concentration (mg/m³) [1−Month Lag]
E2:Pattani
SO2 Surface Concentration (mg/m³) [2−Month Lag]
F1:Pattani
CO Surface Concentration (ppb) [1−Month Lag]
F2:Pattani
CO Surface Concentration (ppb) [2−Month Lag]
G1:Pattani
PM2.5 Surface Concentration (µg/m³) [1−Month Lag]
G2:Pattani
PM2.5 Surface Concentration (µg/m³) [2−Month Lag]

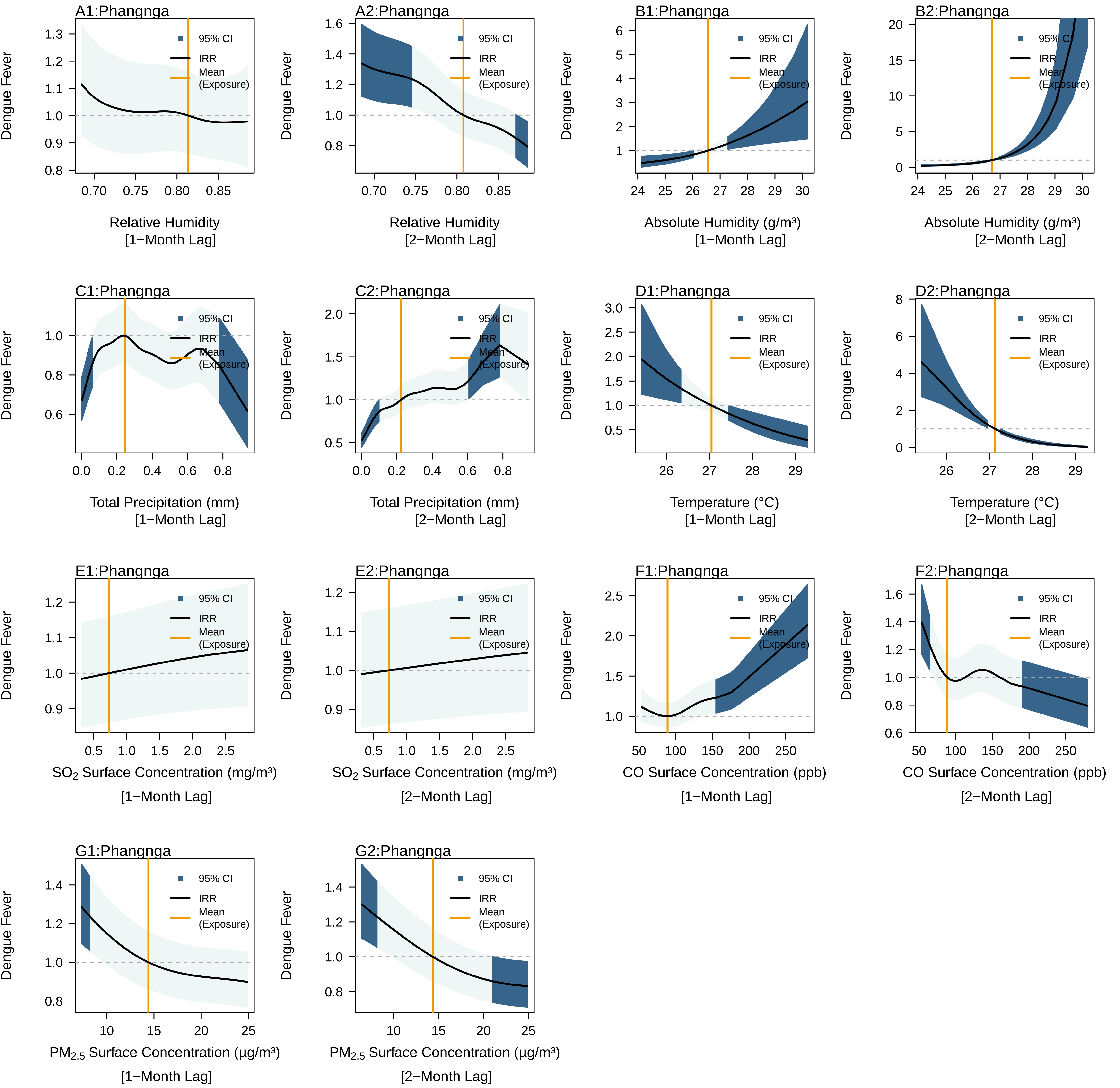

A1:Phangnga
A2:Phangnga
B1:Phangnga
B2:Phangnga
C1:Phangnga
C2:Phangnga
D1:Phangnga
D2:Phangnga
E1:Phangnga
E2:Phangnga
F1:Phangnga
F2:Phangnga
G1:Phangnga
G2:Phangnga
Dengue Fever
95% CI
IRR
Mean
(Exposure)
Relative Humidity
[1−Month Lag]
Relative Humidity
[2−Month Lag]
Absolute Humidity (g/m³)
[1−Month Lag]
Absolute Humidity (g/m³)
[2−Month Lag]
Total Precipitation (mm)
[1−Month Lag]
Total Precipitation (mm)
[2−Month Lag]
Temperature (°C)
[1−Month Lag]
Temperature (°C)
[2−Month Lag]
SO_2 Surface Concentration (mg/m³)
[1−Month Lag]
SO_2 Surface Concentration (mg/m³)
[2−Month Lag]
CO Surface Concentration (ppb)
[1−Month Lag]
CO Surface Concentration (ppb)
[2−Month Lag]
$PM_{2.5}$ Surface Concentration (µg/m³)
[1−Month Lag]
$PM_{2.5}$ Surface Concentration (µg/m³)
[2−Month Lag]

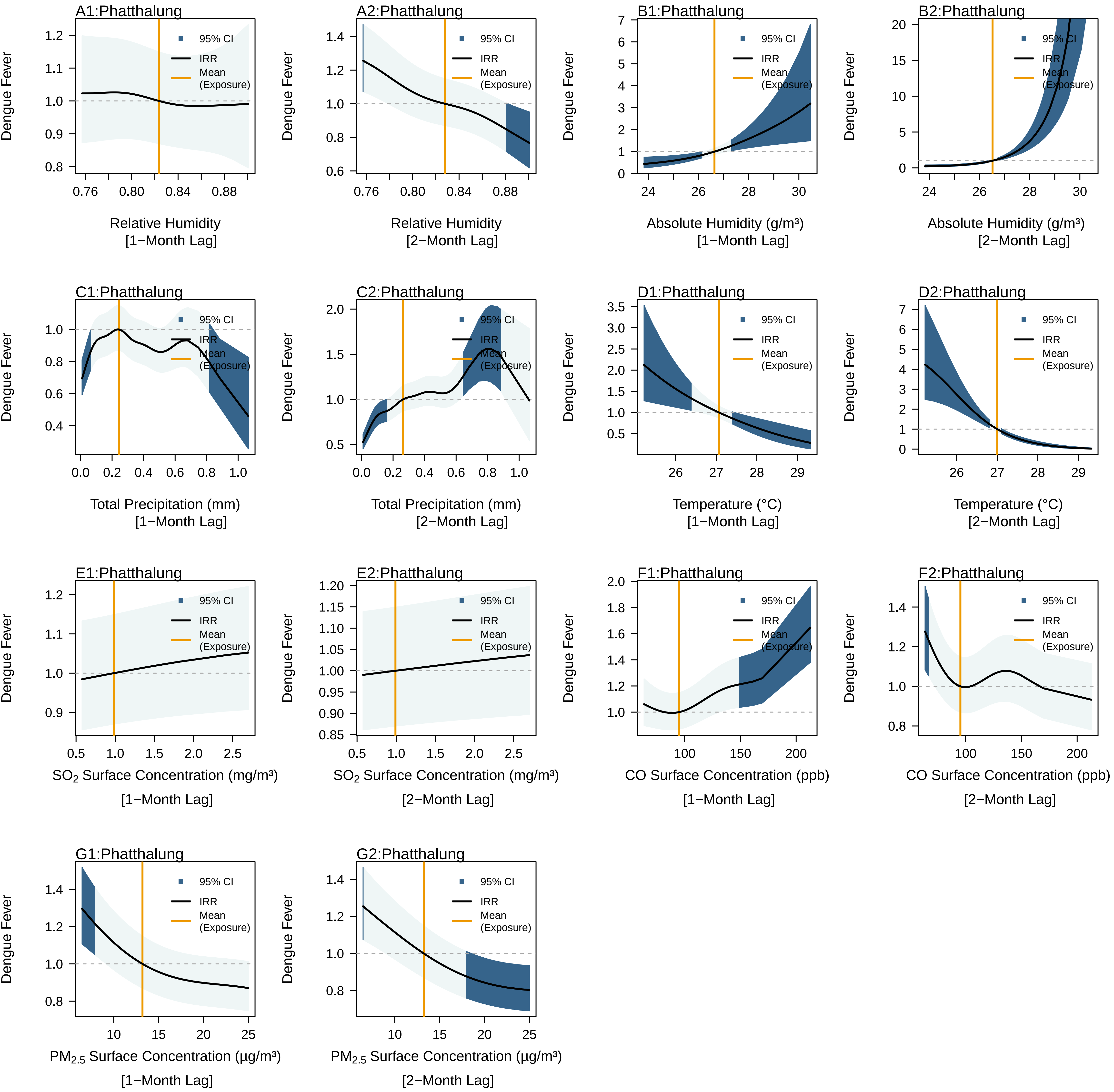
A1:Phatthalung
A2:Phatthalung
B1:Phatthalung
B2:Phatthalung
C1:Phatthalung
C2:Phatthalung
D1:Phatthalung
D2:Phatthalung
E1:Phatthalung
E2:Phatthalung
F1:Phatthalung
F2:Phatthalung
G1:Phatthalung
G2:Phatthalung
95% CI
IRR
Mean (Exposure)
Dengue Fever
Relative Humidity [1−Month Lag]
Relative Humidity [2−Month Lag]
Absolute Humidity (g/m³) [1−Month Lag]
Absolute Humidity (g/m³) [2−Month Lag]
Total Precipitation (mm) [1−Month Lag]
Total Precipitation (mm) [2−Month Lag]
Temperature (°C) [1−Month Lag]
Temperature (°C) [2−Month Lag]
SO_2 Surface Concentration (mg/m³) [1−Month Lag]
SO_2 Surface Concentration (mg/m³) [2−Month Lag]
CO Surface Concentration (ppb) [1−Month Lag]
CO Surface Concentration (ppb) [2−Month Lag]
$PM_{2.5}$ Surface Concentration (µg/m³) [1−Month Lag]
$PM_{2.5}$ Surface Concentration (µg/m³) [2−Month Lag]

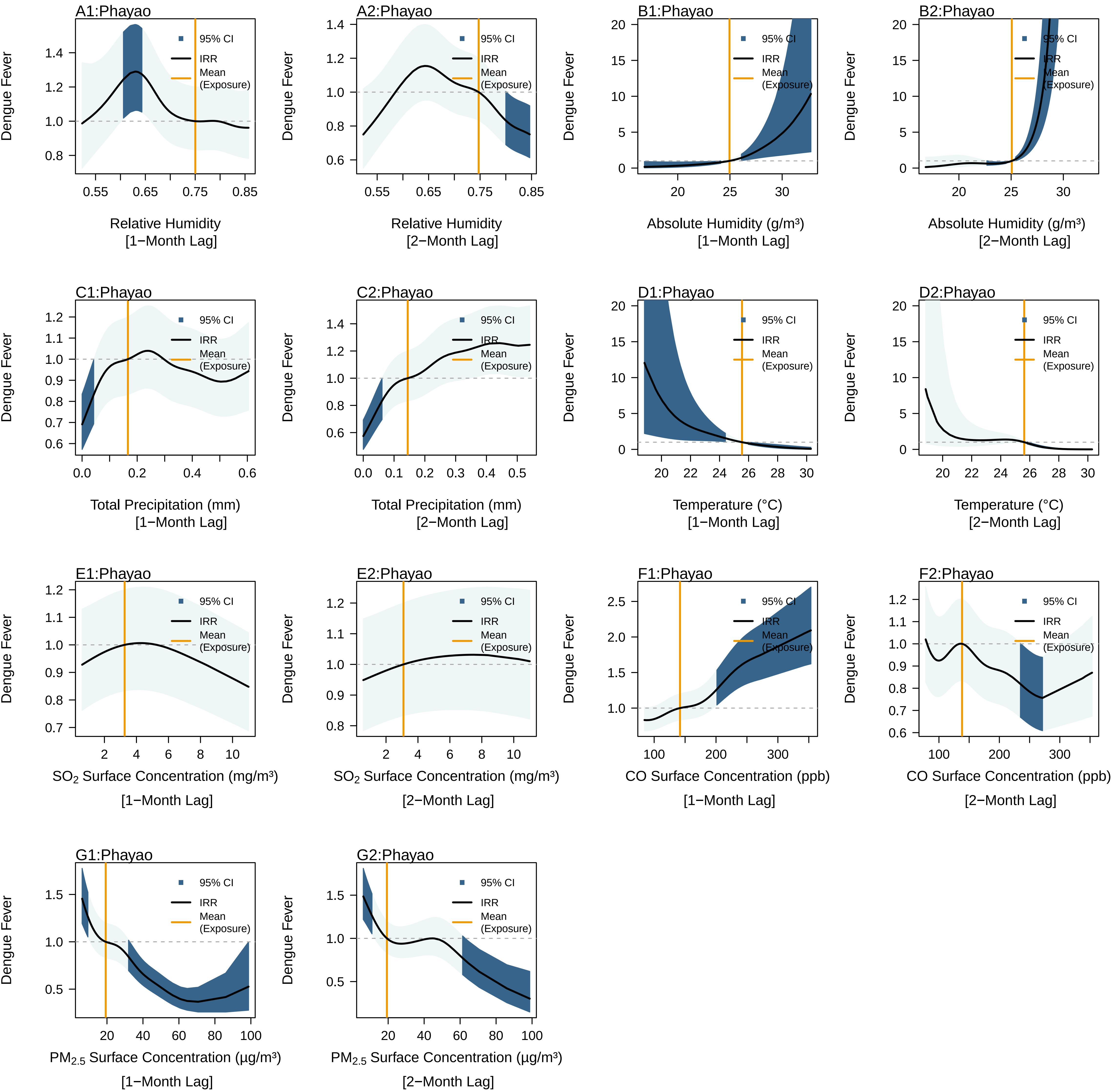
A1:Phayao
Dengue Fever
95% CI
IRR
Mean
(Exposure)
Relative Humidity
[1−Month Lag]
A2:Phayao
Relative Humidity
[2−Month Lag]
B1:Phayao
Absolute Humidity (g/m³)
[1−Month Lag]
B2:Phayao
Absolute Humidity (g/m³)
[2−Month Lag]
C1:Phayao
Total Precipitation (mm)
[1−Month Lag]
C2:Phayao
Total Precipitation (mm)
[2−Month Lag]
D1:Phayao
Temperature (°C)
[1−Month Lag]
D2:Phayao
Temperature (°C)
[2−Month Lag]
E1:Phayao
SO_2 Surface Concentration (mg/m³)
[1−Month Lag]
E2:Phayao
SO_2 Surface Concentration (mg/m³)
[2−Month Lag]
F1:Phayao
CO Surface Concentration (ppb)
[1−Month Lag]
F2:Phayao
CO Surface Concentration (ppb)
[2−Month Lag]
G1:Phayao
$PM_{2.5}$ Surface Concentration (µg/m³)
[1−Month Lag]
G2:Phayao
$PM_{2.5}$ Surface Concentration (µg/m³)
[2−Month Lag]

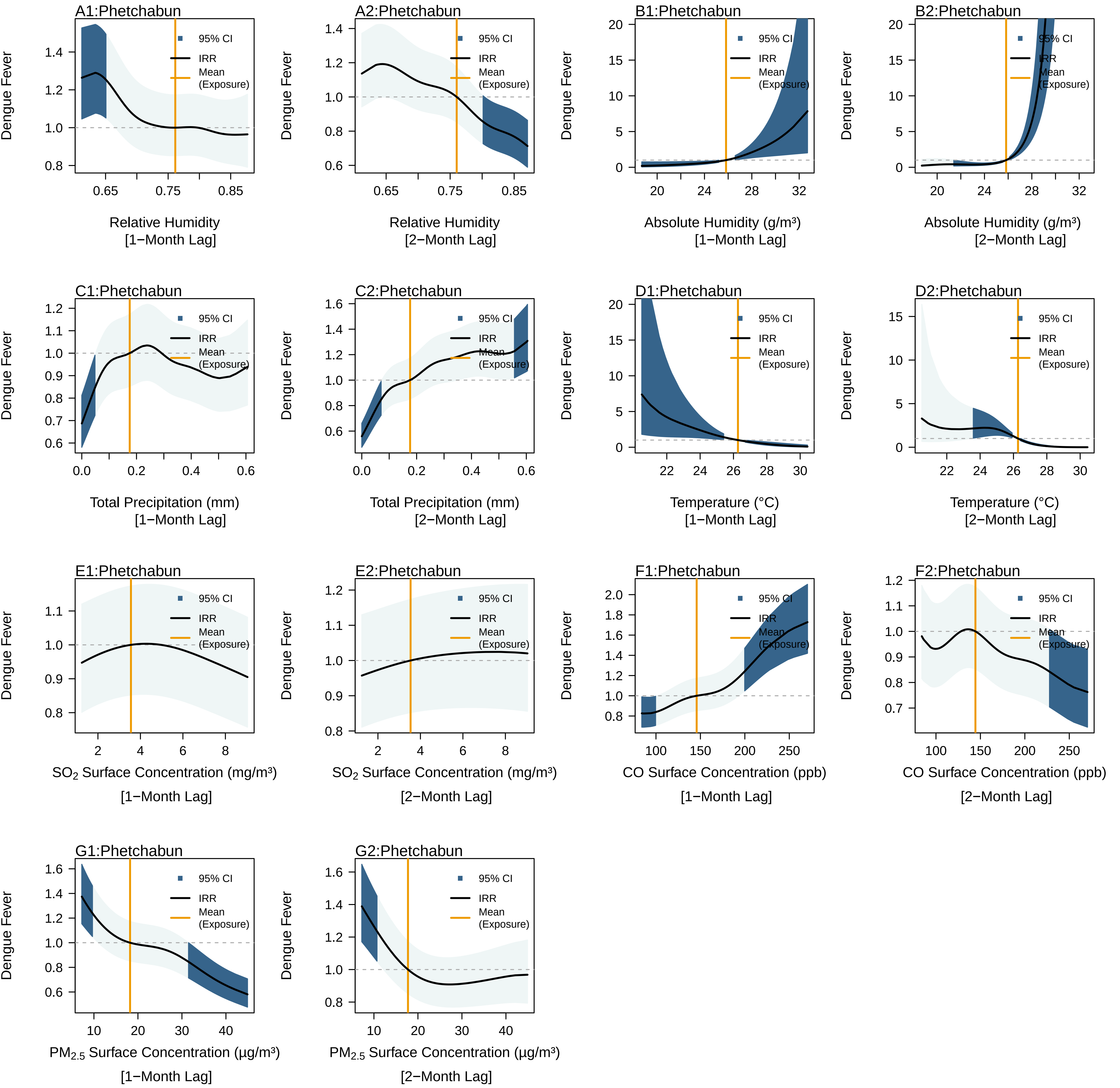

A1:Phetchabun
Dengue Fever
95% CI
IRR
Mean (Exposure)
Relative Humidity [1−Month Lag]
A2:Phetchabun
Dengue Fever
95% CI
IRR
Mean (Exposure)
Relative Humidity [2−Month Lag]
B1:Phetchabun
Dengue Fever
95% CI
IRR
Mean (Exposure)
Absolute Humidity (g/m³) [1−Month Lag]
B2:Phetchabun
Dengue Fever
95% CI
IRR
Mean (Exposure)
Absolute Humidity (g/m³) [2−Month Lag]
C1:Phetchabun
Dengue Fever
95% CI
IRR
Mean (Exposure)
Total Precipitation (mm) [1−Month Lag]
C2:Phetchabun
Dengue Fever
95% CI
IRR
Mean (Exposure)
Total Precipitation (mm) [2−Month Lag]
D1:Phetchabun
Dengue Fever
95% CI
IRR
Mean (Exposure)
Temperature (°C) [1−Month Lag]
D2:Phetchabun
Dengue Fever
95% CI
IRR
Mean (Exposure)
Temperature (°C) [2−Month Lag]
E1:Phetchabun
Dengue Fever
95% CI
IRR
Mean (Exposure)
SO2 Surface Concentration (mg/m³) [1−Month Lag]
E2:Phetchabun
Dengue Fever
95% CI
IRR
Mean (Exposure)
SO2 Surface Concentration (mg/m³) [2−Month Lag]
F1:Phetchabun
Dengue Fever
95% CI
IRR
Mean (Exposure)
CO Surface Concentration (ppb) [1−Month Lag]
F2:Phetchabun
Dengue Fever
95% CI
IRR
Mean (Exposure)
CO Surface Concentration (ppb) [2−Month Lag]
G1:Phetchabun
Dengue Fever
95% CI
IRR
Mean (Exposure)
PM2.5 Surface Concentration (μg/m³) [1−Month Lag]
G2:Phetchabun
Dengue Fever
95% CI
IRR
Mean (Exposure)
PM2.5 Surface Concentration (μg/m³) [2−Month Lag]

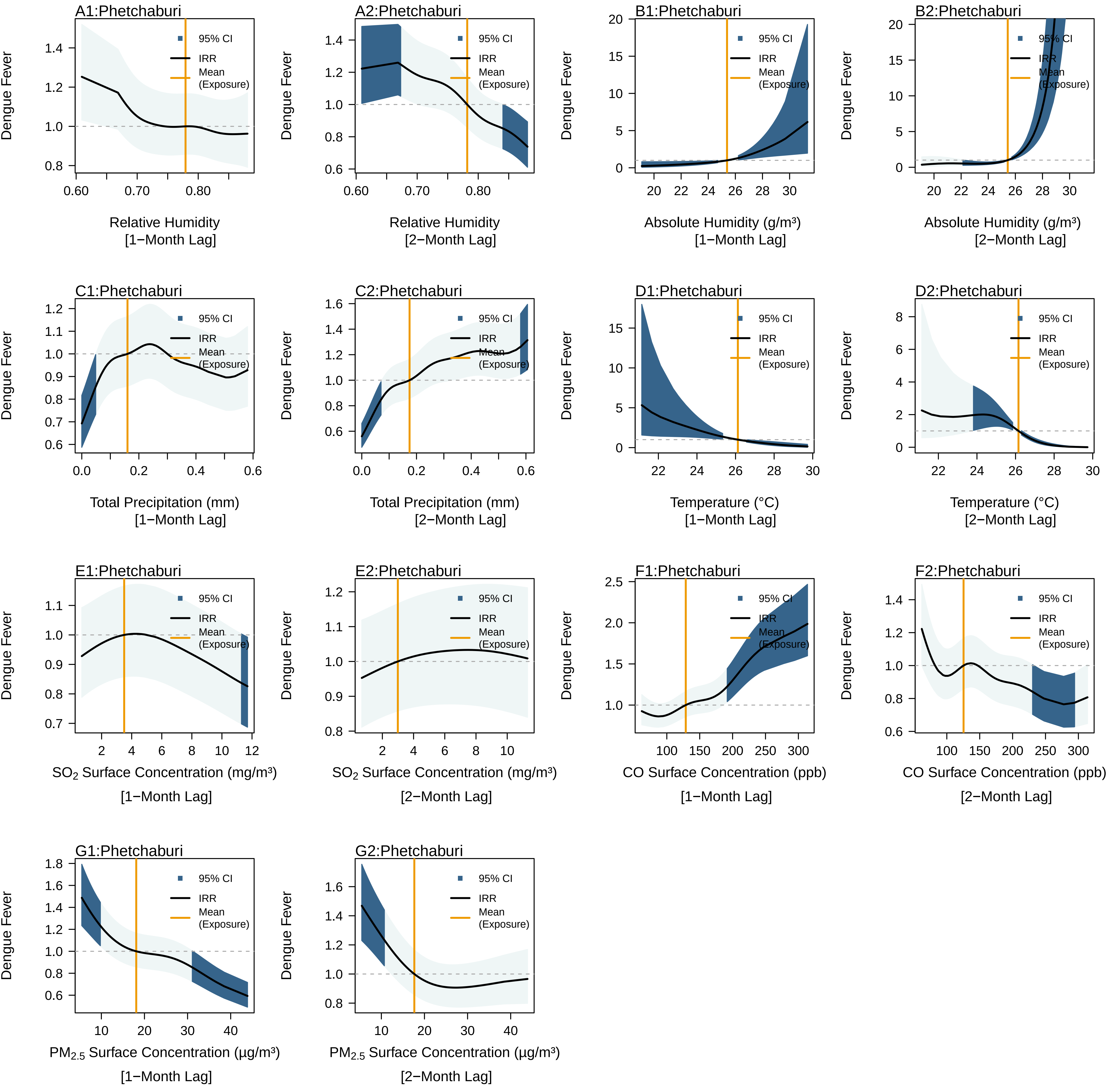
A1:Phetchaburi
A2:Phetchaburi
B1:Phetchaburi
B2:Phetchaburi
C1:Phetchaburi
C2:Phetchaburi
D1:Phetchaburi
D2:Phetchaburi
E1:Phetchaburi
E2:Phetchaburi
F1:Phetchaburi
F2:Phetchaburi
G1:Phetchaburi
G2:Phetchaburi
Dengue Fever
95% CI
IRR
Mean
(Exposure)
Relative Humidity
[1−Month Lag]
Relative Humidity
[2−Month Lag]
Absolute Humidity (g/m³)
[1−Month Lag]
Absolute Humidity (g/m³)
[2−Month Lag]
Total Precipitation (mm)
[1−Month Lag]
Total Precipitation (mm)
[2−Month Lag]
Temperature (°C)
[1−Month Lag]
Temperature (°C)
[2−Month Lag]
SO_2 Surface Concentration (mg/m³)
[1−Month Lag]
SO_2 Surface Concentration (mg/m³)
[2−Month Lag]
CO Surface Concentration (ppb)
[1−Month Lag]
CO Surface Concentration (ppb)
[2−Month Lag]
$PM_{2.5}$ Surface Concentration (µg/m³)
[1−Month Lag]
$PM_{2.5}$ Surface Concentration (µg/m³)
[2−Month Lag]

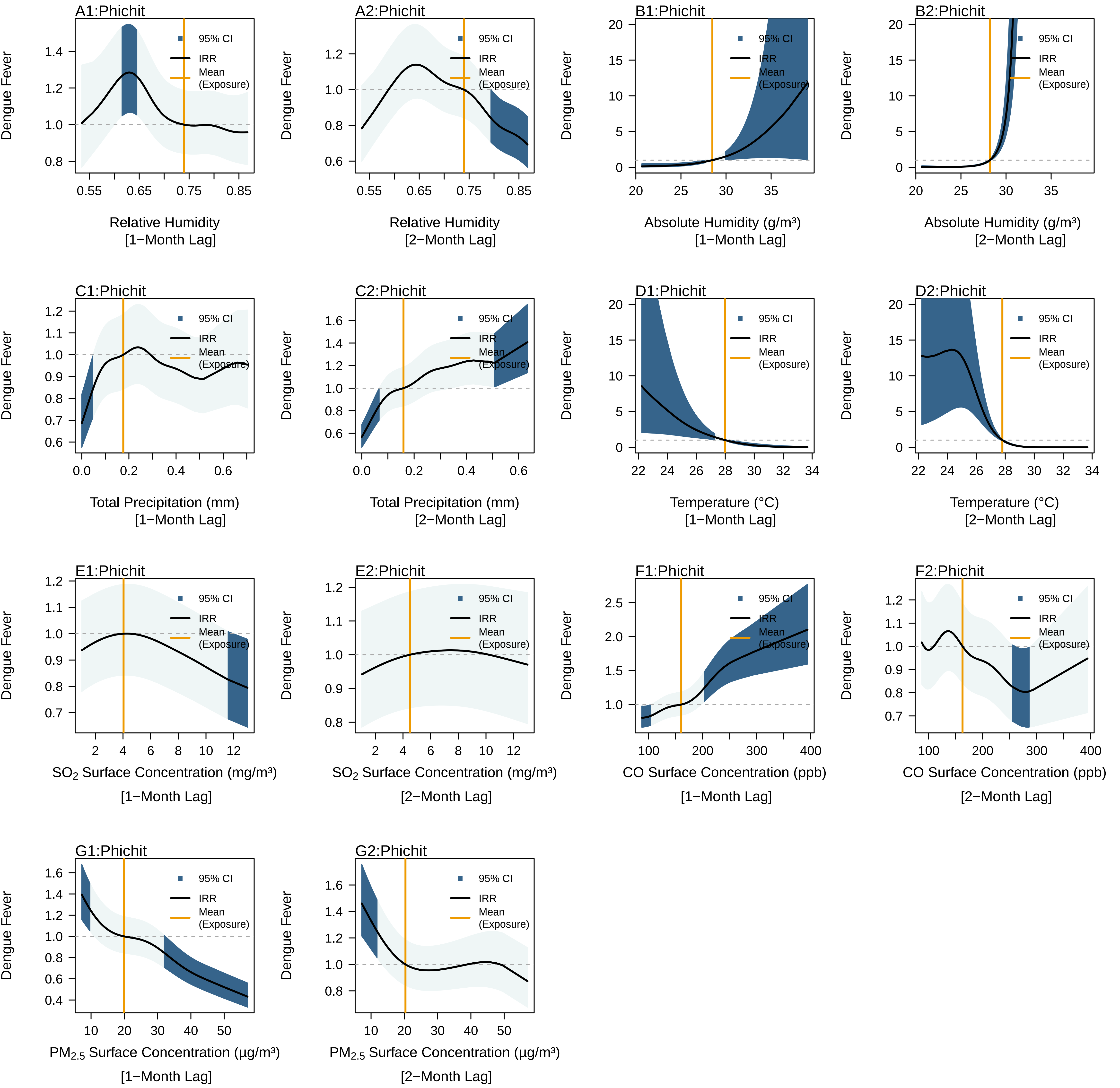
A1:Phichit
A2:Phichit
B1:Phichit
B2:Phichit
C1:Phichit
C2:Phichit
D1:Phichit
D2:Phichit
E1:Phichit
E2:Phichit
F1:Phichit
F2:Phichit
G1:Phichit
G2:Phichit
Dengue Fever
95% CI
IRR
Mean
(Exposure)
Relative Humidity
[1−Month Lag]
[2−Month Lag]
Absolute Humidity (g/m³)
Total Precipitation (mm)
Temperature (°C)
SO_2 Surface Concentration (mg/m³)
CO Surface Concentration (ppb)
$PM_{2.5}$ Surface Concentration (µg/m³)

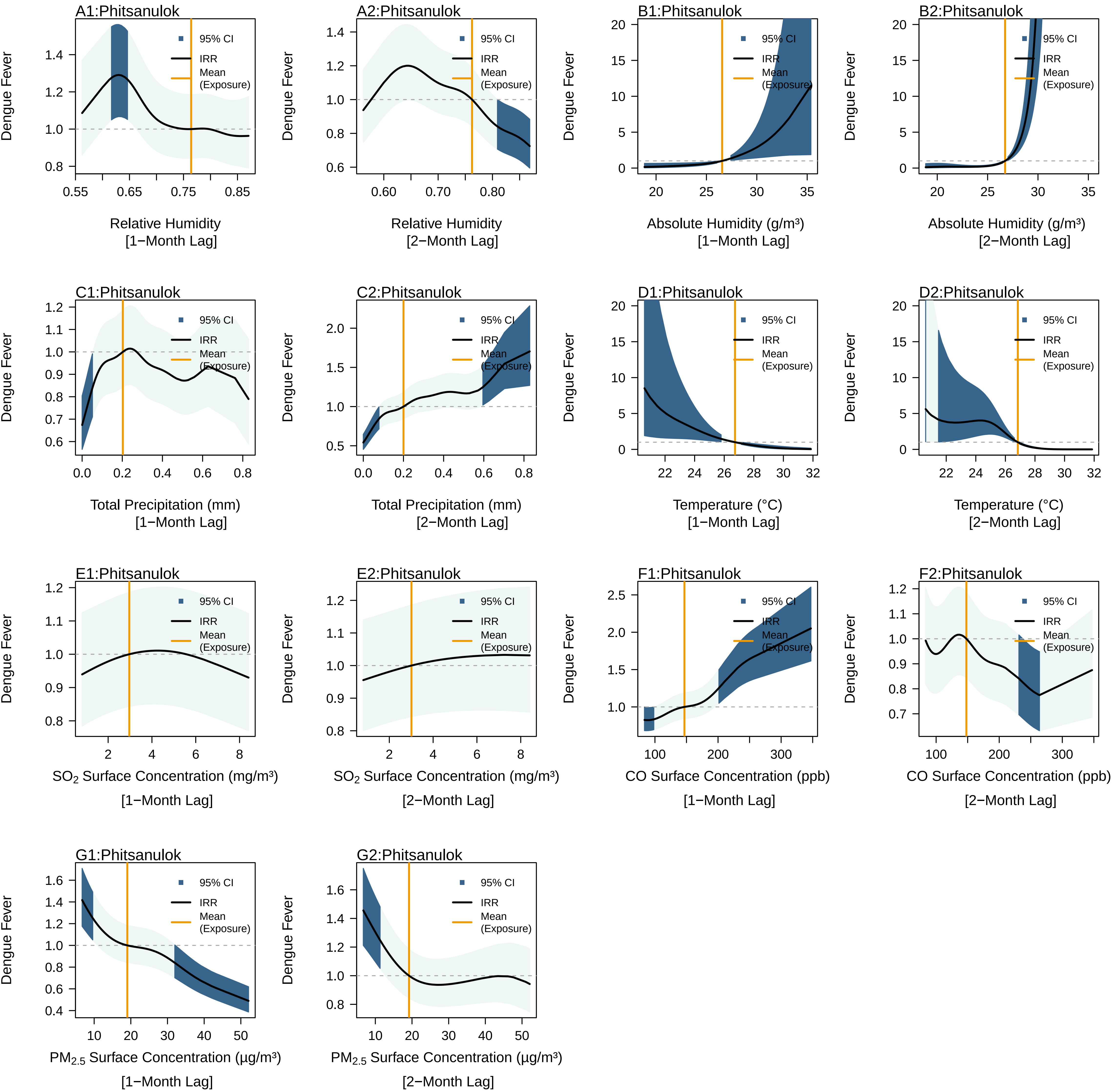
A1:Phitsanulok
A2:Phitsanulok
B1:Phitsanulok
B2:Phitsanulok
C1:Phitsanulok
C2:Phitsanulok
D1:Phitsanulok
D2:Phitsanulok
E1:Phitsanulok
E2:Phitsanulok
F1:Phitsanulok
F2:Phitsanulok
G1:Phitsanulok
G2:Phitsanulok
Dengue Fever
95% CI
IRR
Mean (Exposure)
Relative Humidity [1−Month Lag]
Relative Humidity [2−Month Lag]
Absolute Humidity (g/m³) [1−Month Lag]
Absolute Humidity (g/m³) [2−Month Lag]
Total Precipitation (mm) [1−Month Lag]
Total Precipitation (mm) [2−Month Lag]
Temperature (°C) [1−Month Lag]
Temperature (°C) [2−Month Lag]
SO2 Surface Concentration (mg/m³) [1−Month Lag]
SO2 Surface Concentration (mg/m³) [2−Month Lag]
CO Surface Concentration (ppb) [1−Month Lag]
CO Surface Concentration (ppb) [2−Month Lag]
PM2.5 Surface Concentration (µg/m³) [1−Month Lag]
PM2.5 Surface Concentration (µg/m³) [2−Month Lag]

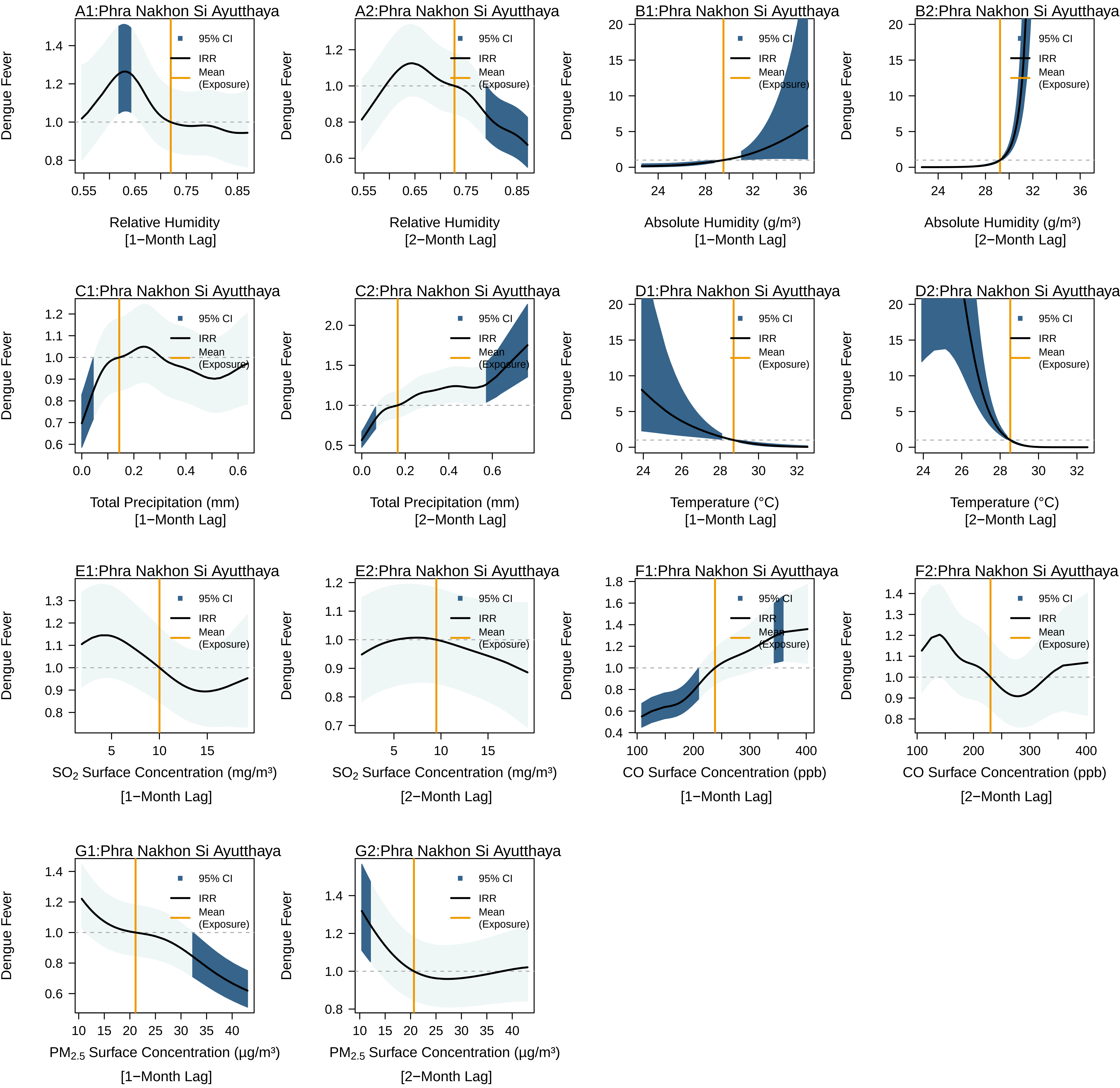
A1:Phra Nakhon Si Ayutthaya
Dengue Fever
95% CI
IRR
Mean (Exposure)
Relative Humidity [1−Month Lag]
A2:Phra Nakhon Si Ayutthaya
Dengue Fever
95% CI
IRR
Mean (Exposure)
Relative Humidity [2−Month Lag]
B1:Phra Nakhon Si Ayutthaya
Dengue Fever
95% CI
IRR
Mean (Exposure)
Absolute Humidity (g/m³) [1−Month Lag]
B2:Phra Nakhon Si Ayutthaya
Dengue Fever
95% CI
IRR
Mean (Exposure)
Absolute Humidity (g/m³) [2−Month Lag]
C1:Phra Nakhon Si Ayutthaya
Dengue Fever
95% CI
IRR
Mean (Exposure)
Total Precipitation (mm) [1−Month Lag]
C2:Phra Nakhon Si Ayutthaya
Dengue Fever
95% CI
IRR
Mean (Exposure)
Total Precipitation (mm) [2−Month Lag]
D1:Phra Nakhon Si Ayutthaya
Dengue Fever
95% CI
IRR
Mean (Exposure)
Temperature (°C) [1−Month Lag]
D2:Phra Nakhon Si Ayutthaya
Dengue Fever
95% CI
IRR
Mean (Exposure)
Temperature (°C) [2−Month Lag]
E1:Phra Nakhon Si Ayutthaya
Dengue Fever
95% CI
IRR
Mean (Exposure)
SO_2 Surface Concentration (mg/m³) [1−Month Lag]
E2:Phra Nakhon Si Ayutthaya
Dengue Fever
95% CI
IRR
Mean (Exposure)
SO_2 Surface Concentration (mg/m³) [2−Month Lag]
F1:Phra Nakhon Si Ayutthaya
Dengue Fever
95% CI
IRR
Mean (Exposure)
CO Surface Concentration (ppb) [1−Month Lag]
F2:Phra Nakhon Si Ayutthaya
Dengue Fever
95% CI
IRR
Mean (Exposure)
CO Surface Concentration (ppb) [2−Month Lag]
G1:Phra Nakhon Si Ayutthaya
Dengue Fever
95% CI
IRR
Mean (Exposure)
$PM_{2.5}$ Surface Concentration (µg/m³) [1−Month Lag]
G2:Phra Nakhon Si Ayutthaya
Dengue Fever
95% CI
IRR
Mean (Exposure)
$PM_{2.5}$ Surface Concentration (µg/m³) [2−Month Lag]

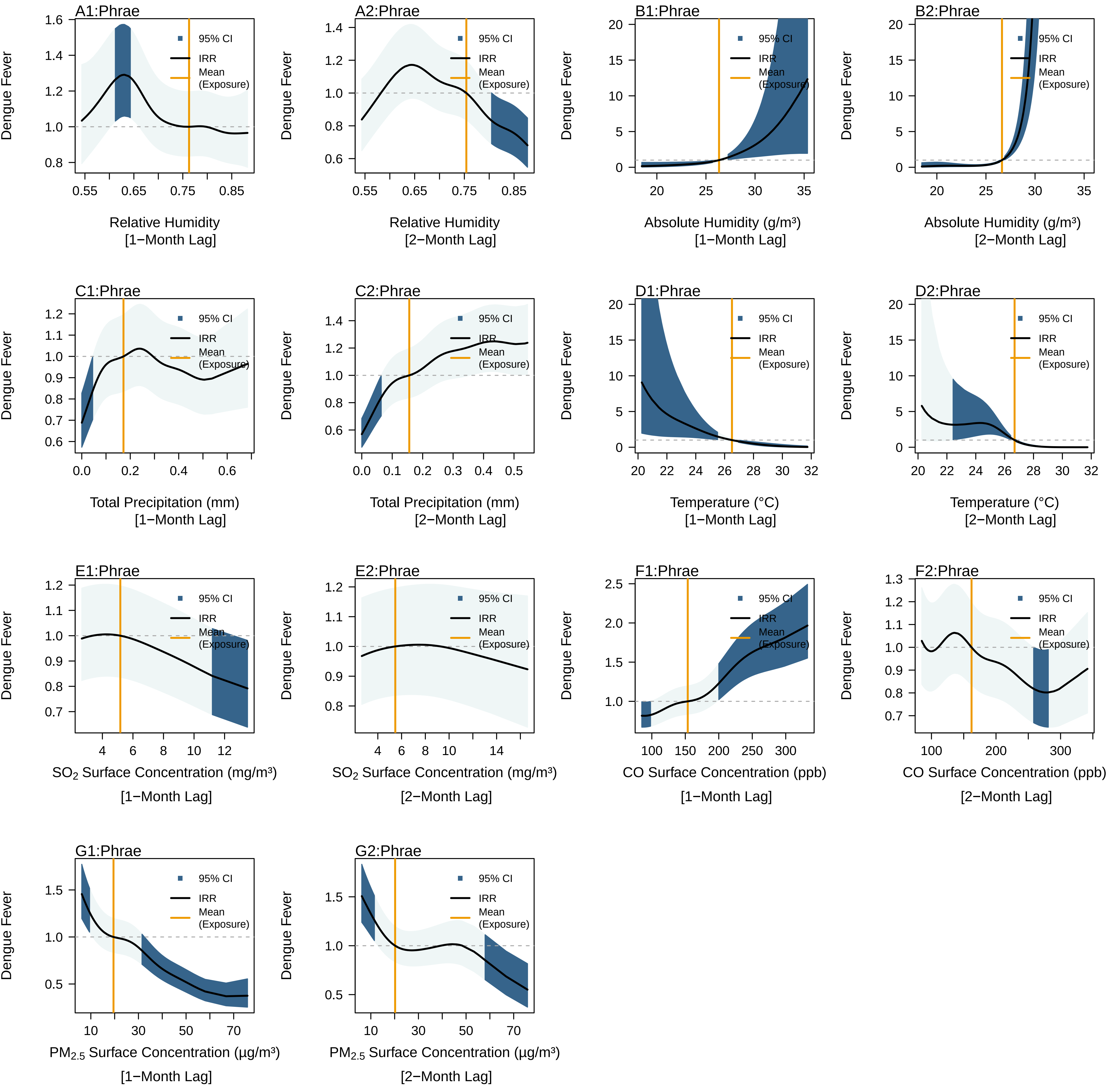
A1:Phrae
Dengue Fever
95% CI
IRR
Mean (Exposure)
Relative Humidity [1−Month Lag]
A2:Phrae
Relative Humidity [2−Month Lag]
B1:Phrae
Absolute Humidity (g/m³) [1−Month Lag]
B2:Phrae
Absolute Humidity (g/m³) [2−Month Lag]
C1:Phrae
Total Precipitation (mm) [1−Month Lag]
C2:Phrae
Total Precipitation (mm) [2−Month Lag]
D1:Phrae
Temperature (°C) [1−Month Lag]
D2:Phrae
Temperature (°C) [2−Month Lag]
E1:Phrae
SO2 Surface Concentration (mg/m³) [1−Month Lag]
E2:Phrae
SO2 Surface Concentration (mg/m³) [2−Month Lag]
F1:Phrae
CO Surface Concentration (ppb) [1−Month Lag]
F2:Phrae
CO Surface Concentration (ppb) [2−Month Lag]
G1:Phrae
PM2.5 Surface Concentration (µg/m³) [1−Month Lag]
G2:Phrae
PM2.5 Surface Concentration (µg/m³) [2−Month Lag]

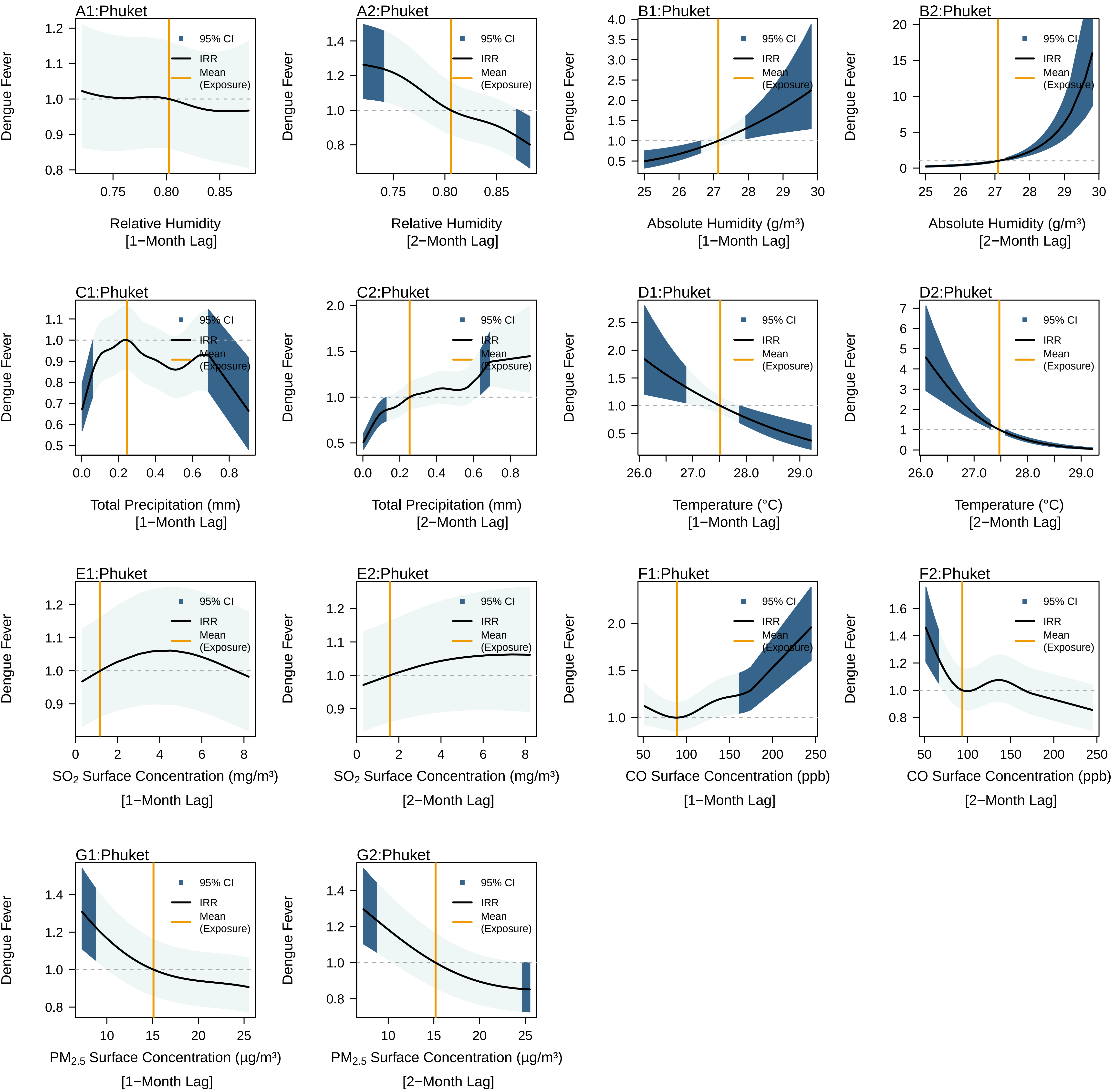
A1:Phuket
Dengue Fever
95% CI
IRR
Mean
(Exposure)
Relative Humidity
[1−Month Lag]
A2:Phuket
Relative Humidity
[2−Month Lag]
B1:Phuket
Absolute Humidity (g/m³)
[1−Month Lag]
B2:Phuket
Absolute Humidity (g/m³)
[2−Month Lag]
C1:Phuket
Total Precipitation (mm)
[1−Month Lag]
C2:Phuket
Total Precipitation (mm)
[2−Month Lag]
D1:Phuket
Temperature (°C)
[1−Month Lag]
D2:Phuket
Temperature (°C)
[2−Month Lag]
E1:Phuket
SO_2 Surface Concentration (mg/m³)
[1−Month Lag]
E2:Phuket
SO_2 Surface Concentration (mg/m³)
[2−Month Lag]
F1:Phuket
CO Surface Concentration (ppb)
[1−Month Lag]
F2:Phuket
CO Surface Concentration (ppb)
[2−Month Lag]
G1:Phuket
$PM_{2.5}$ Surface Concentration (μg/m³)
[1−Month Lag]
G2:Phuket
$PM_{2.5}$ Surface Concentration (μg/m³)
[2−Month Lag]

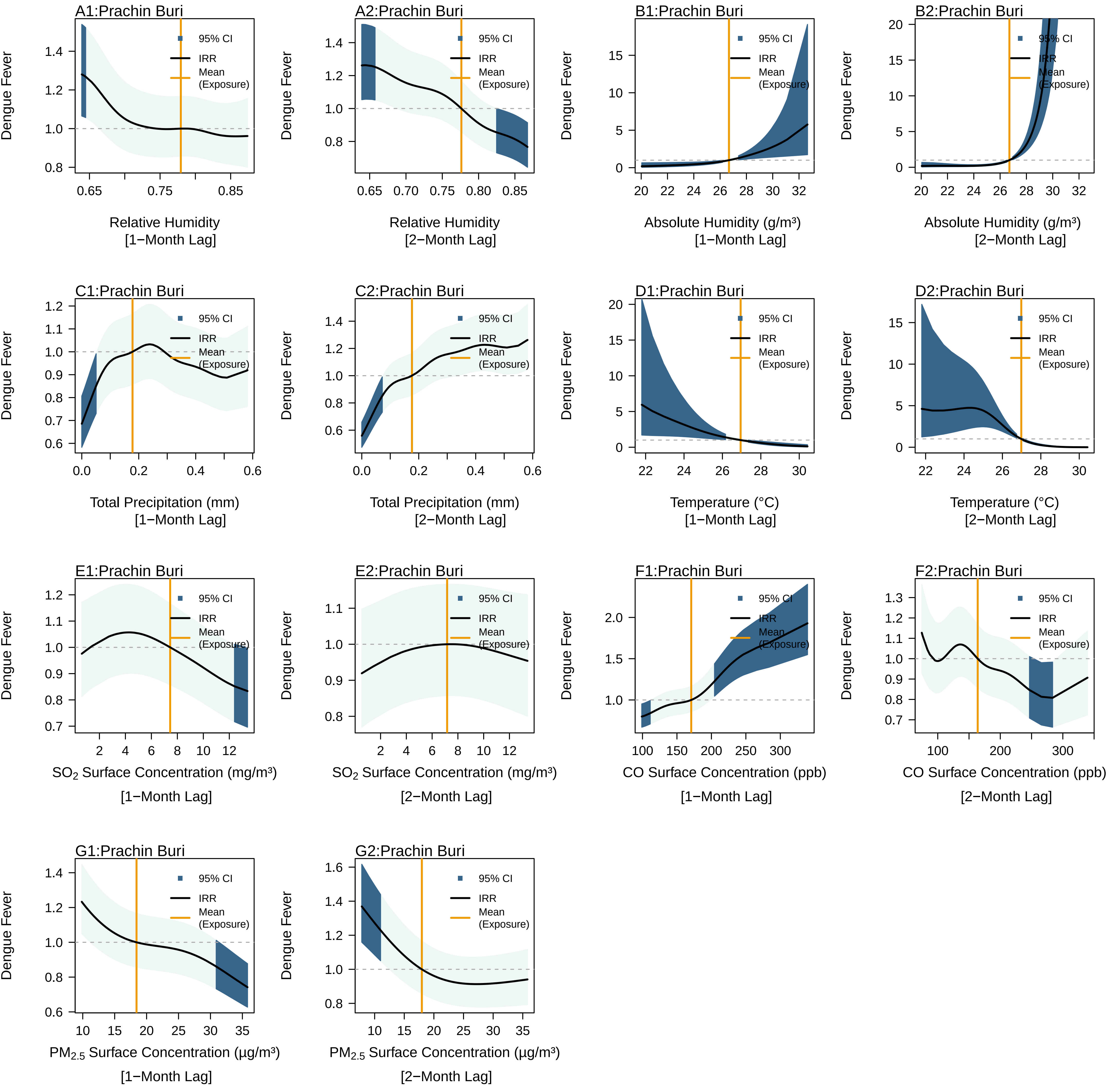
A1:Prachin Buri
A2:Prachin Buri
B1:Prachin Buri
B2:Prachin Buri
C1:Prachin Buri
C2:Prachin Buri
D1:Prachin Buri
D2:Prachin Buri
E1:Prachin Buri
E2:Prachin Buri
F1:Prachin Buri
F2:Prachin Buri
G1:Prachin Buri
G2:Prachin Buri
95% CI
IRR
Mean (Exposure)
Dengue Fever
Relative Humidity [1−Month Lag]
Relative Humidity [2−Month Lag]
Absolute Humidity (g/m³) [1−Month Lag]
Absolute Humidity (g/m³) [2−Month Lag]
Total Precipitation (mm) [1−Month Lag]
Total Precipitation (mm) [2−Month Lag]
Temperature (°C) [1−Month Lag]
Temperature (°C) [2−Month Lag]
SO2 Surface Concentration (mg/m³) [1−Month Lag]
SO2 Surface Concentration (mg/m³) [2−Month Lag]
CO Surface Concentration (ppb) [1−Month Lag]
CO Surface Concentration (ppb) [2−Month Lag]
PM2.5 Surface Concentration (µg/m³) [1−Month Lag]
PM2.5 Surface Concentration (µg/m³) [2−Month Lag]

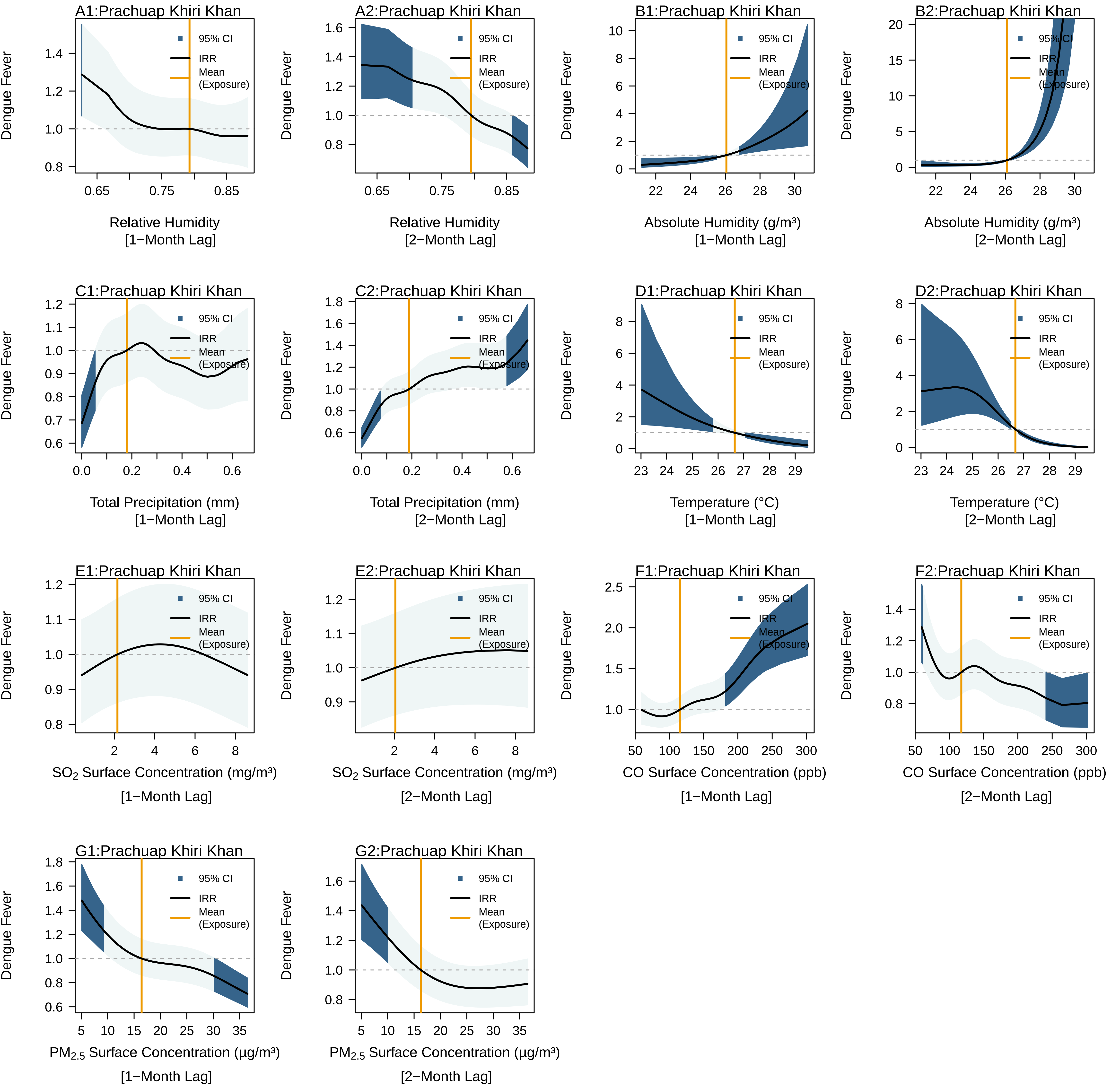
A1:Prachuap Khiri Khan
A2:Prachuap Khiri Khan
B1:Prachuap Khiri Khan
B2:Prachuap Khiri Khan
C1:Prachuap Khiri Khan
C2:Prachuap Khiri Khan
D1:Prachuap Khiri Khan
D2:Prachuap Khiri Khan
E1:Prachuap Khiri Khan
E2:Prachuap Khiri Khan
F1:Prachuap Khiri Khan
F2:Prachuap Khiri Khan
G1:Prachuap Khiri Khan
G2:Prachuap Khiri Khan
95% CI
IRR
Mean (Exposure)
Dengue Fever
Relative Humidity [1−Month Lag]
Relative Humidity [2−Month Lag]
Absolute Humidity (g/m³) [1−Month Lag]
Absolute Humidity (g/m³) [2−Month Lag]
Total Precipitation (mm) [1−Month Lag]
Total Precipitation (mm) [2−Month Lag]
Temperature (°C) [1−Month Lag]
Temperature (°C) [2−Month Lag]
SO_2 Surface Concentration (mg/m³) [1−Month Lag]
SO_2 Surface Concentration (mg/m³) [2−Month Lag]
CO Surface Concentration (ppb) [1−Month Lag]
CO Surface Concentration (ppb) [2−Month Lag]
$PM_{2.5}$ Surface Concentration (µg/m³) [1−Month Lag]
$PM_{2.5}$ Surface Concentration (µg/m³) [2−Month Lag]

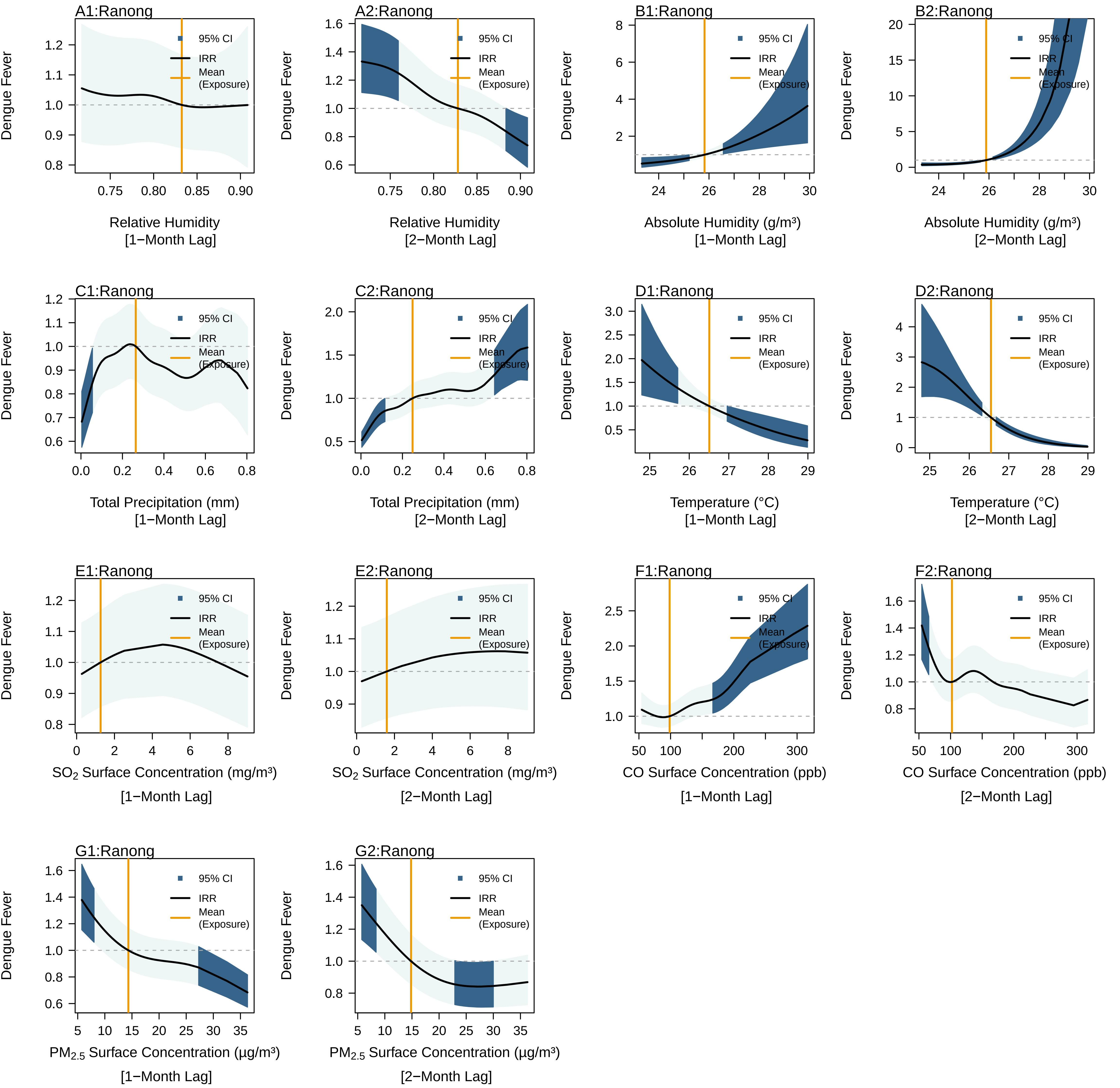
A1:Ranong
A2:Ranong
B1:Ranong
B2:Ranong
95% CI
IRR
Mean
(Exposure)
Dengue Fever
Relative Humidity
[1−Month Lag]
Relative Humidity
[2−Month Lag]
Absolute Humidity (g/m³)
[1−Month Lag]
Absolute Humidity (g/m³)
[2−Month Lag]
C1:Ranong
C2:Ranong
D1:Ranong
D2:Ranong
Total Precipitation (mm)
[1−Month Lag]
Total Precipitation (mm)
[2−Month Lag]
Temperature (°C)
[1−Month Lag]
Temperature (°C)
[2−Month Lag]
E1:Ranong
E2:Ranong
F1:Ranong
F2:Ranong
SO_2 Surface Concentration (mg/m³)
[1−Month Lag]
SO_2 Surface Concentration (mg/m³)
[2−Month Lag]
CO Surface Concentration (ppb)
[1−Month Lag]
CO Surface Concentration (ppb)
[2−Month Lag]
G1:Ranong
G2:Ranong
$PM_{2.5}$ Surface Concentration (µg/m³)
[1−Month Lag]
$PM_{2.5}$ Surface Concentration (µg/m³)
[2−Month Lag]

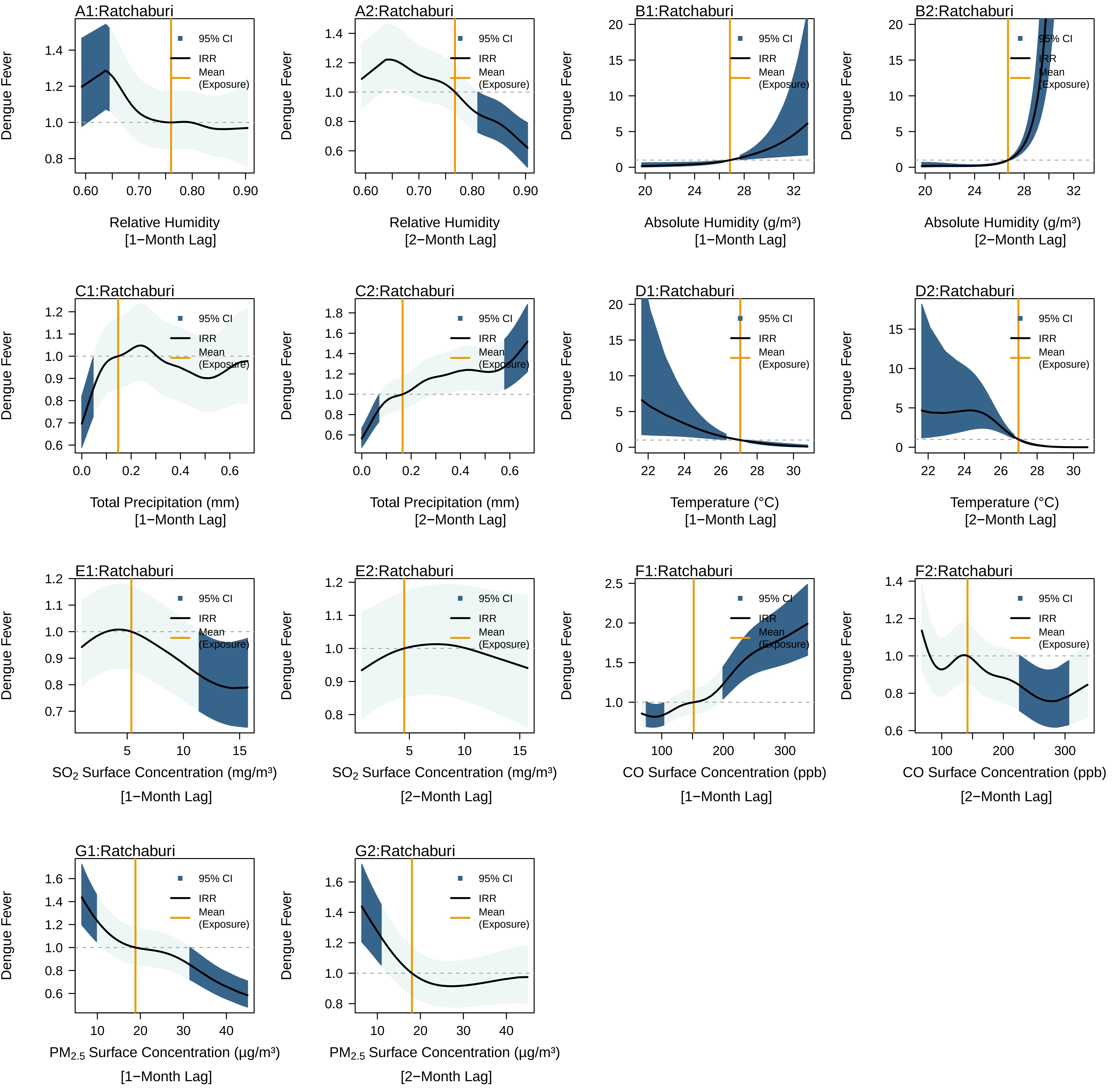
A1:Ratchaburi
Dengue Fever
95% CI
IRR
Mean (Exposure)
Relative Humidity [1−Month Lag]
A2:Ratchaburi
Relative Humidity [2−Month Lag]
B1:Ratchaburi
Absolute Humidity (g/m³) [1−Month Lag]
B2:Ratchaburi
Absolute Humidity (g/m³) [2−Month Lag]
C1:Ratchaburi
Total Precipitation (mm) [1−Month Lag]
C2:Ratchaburi
Total Precipitation (mm) [2−Month Lag]
D1:Ratchaburi
Temperature (°C) [1−Month Lag]
D2:Ratchaburi
Temperature (°C) [2−Month Lag]
E1:Ratchaburi
SO_2 Surface Concentration (mg/m³) [1−Month Lag]
E2:Ratchaburi
SO_2 Surface Concentration (mg/m³) [2−Month Lag]
F1:Ratchaburi
CO Surface Concentration (ppb) [1−Month Lag]
F2:Ratchaburi
CO Surface Concentration (ppb) [2−Month Lag]
G1:Ratchaburi
$PM_{2.5}$ Surface Concentration (µg/m³) [1−Month Lag]
G2:Ratchaburi
$PM_{2.5}$ Surface Concentration (µg/m³) [2−Month Lag]

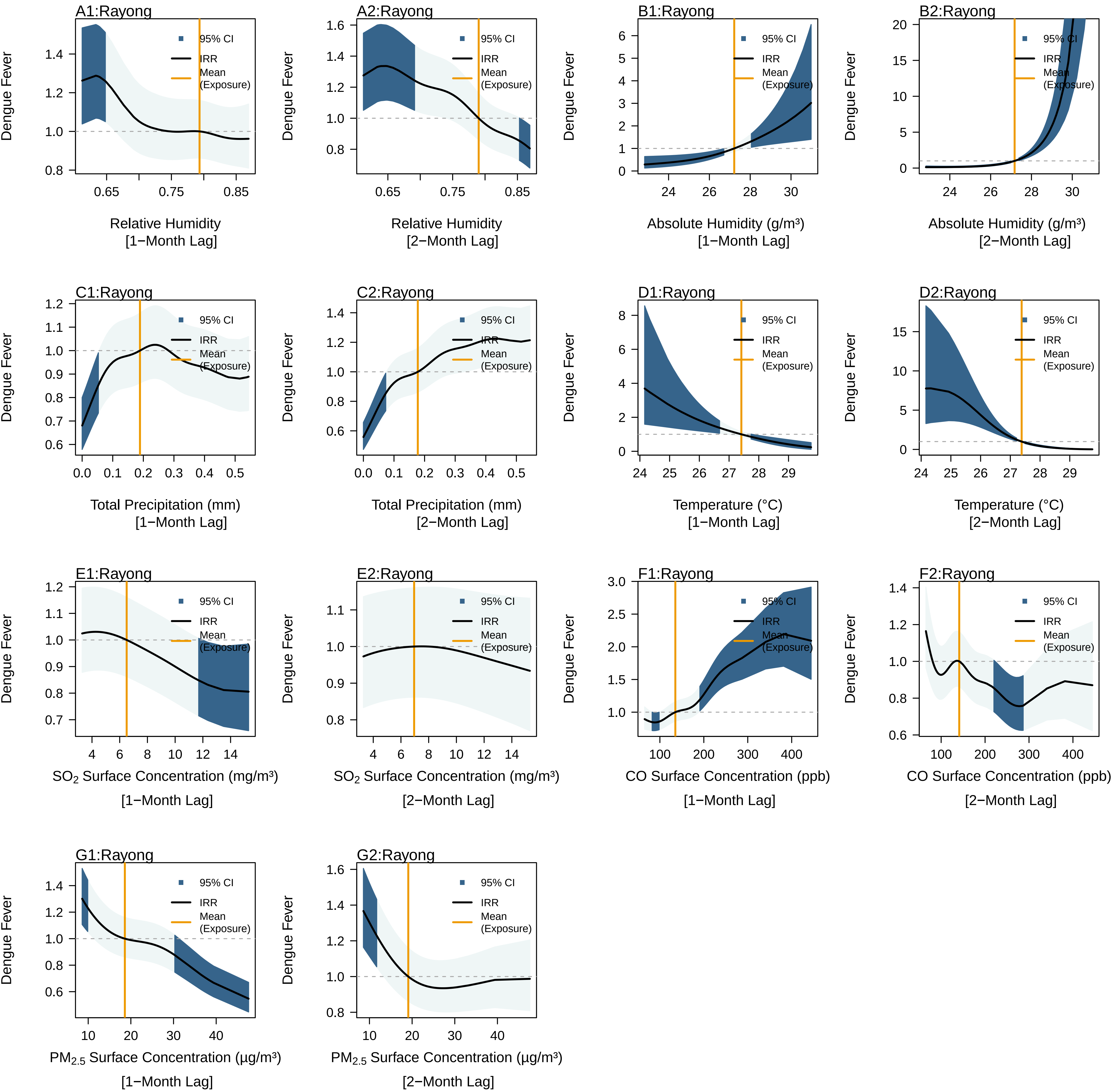
A1:Rayong
A2:Rayong
B1:Rayong
B2:Rayong
C1:Rayong
C2:Rayong
D1:Rayong
D2:Rayong
E1:Rayong
E2:Rayong
F1:Rayong
F2:Rayong
G1:Rayong
G2:Rayong
95% CI
IRR
Mean (Exposure)
Dengue Fever
Relative Humidity [1−Month Lag]
Relative Humidity [2−Month Lag]
Absolute Humidity (g/m³) [1−Month Lag]
Absolute Humidity (g/m³) [2−Month Lag]
Total Precipitation (mm) [1−Month Lag]
Total Precipitation (mm) [2−Month Lag]
Temperature (°C) [1−Month Lag]
Temperature (°C) [2−Month Lag]
SO_2 Surface Concentration (mg/m³) [1−Month Lag]
SO_2 Surface Concentration (mg/m³) [2−Month Lag]
CO Surface Concentration (ppb) [1−Month Lag]
CO Surface Concentration (ppb) [2−Month Lag]
$PM_{2.5}$ Surface Concentration (µg/m³) [1−Month Lag]
$PM_{2.5}$ Surface Concentration (µg/m³) [2−Month Lag]

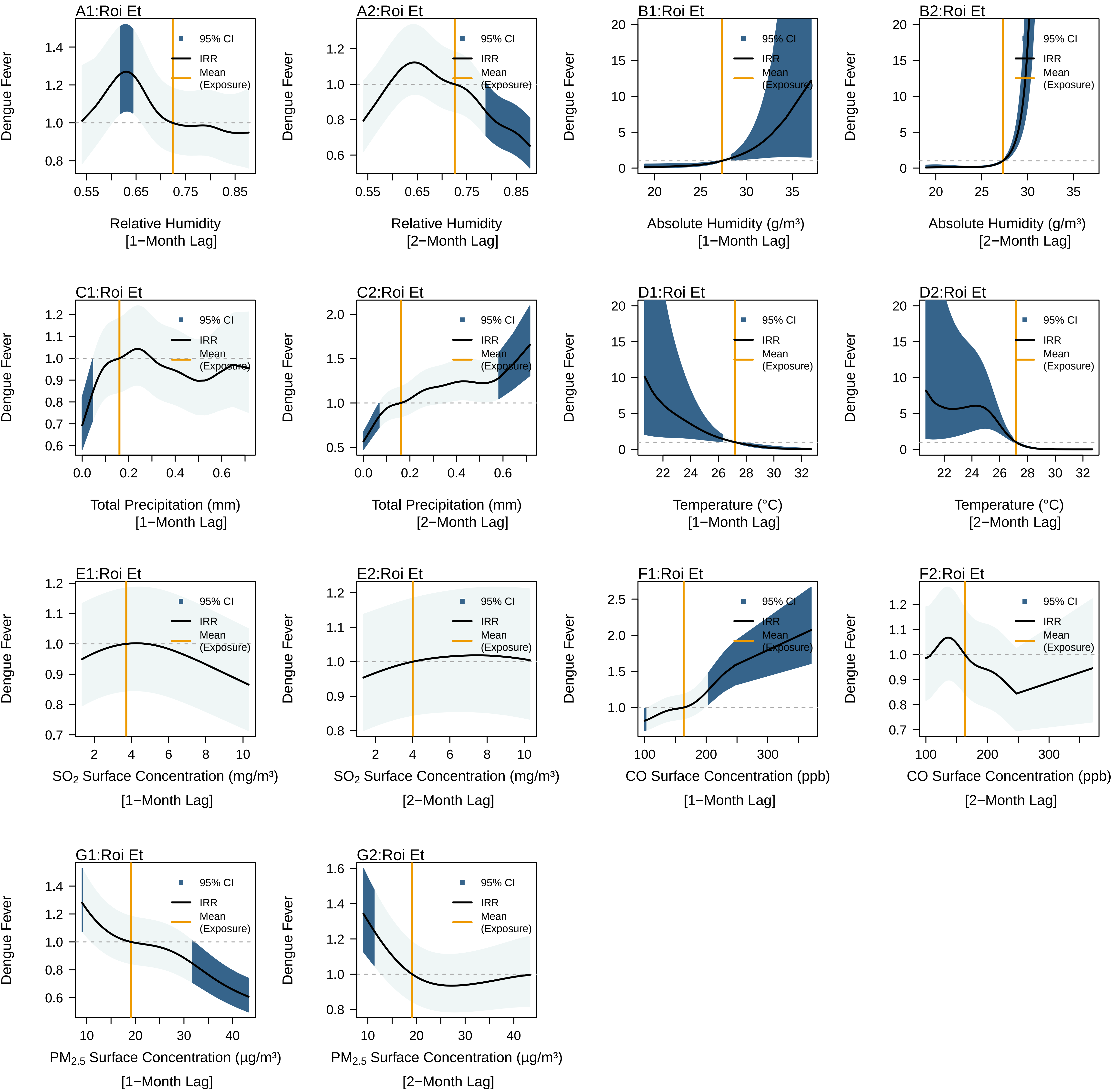
A1:Roi Et
Dengue Fever
95% CI
IRR
Mean
(Exposure)
Relative Humidity
[1−Month Lag]
A2:Roi Et
Dengue Fever
Relative Humidity
[2−Month Lag]
B1:Roi Et
Dengue Fever
Absolute Humidity (g/m³)
[1−Month Lag]
B2:Roi Et
Dengue Fever
Absolute Humidity (g/m³)
[2−Month Lag]
C1:Roi Et
Dengue Fever
Total Precipitation (mm)
[1−Month Lag]
C2:Roi Et
Dengue Fever
Total Precipitation (mm)
[2−Month Lag]
D1:Roi Et
Dengue Fever
Temperature (°C)
[1−Month Lag]
D2:Roi Et
Dengue Fever
Temperature (°C)
[2−Month Lag]
E1:Roi Et
Dengue Fever
SO2 Surface Concentration (mg/m³)
[1−Month Lag]
E2:Roi Et
Dengue Fever
SO2 Surface Concentration (mg/m³)
[2−Month Lag]
F1:Roi Et
Dengue Fever
CO Surface Concentration (ppb)
[1−Month Lag]
F2:Roi Et
Dengue Fever
CO Surface Concentration (ppb)
[2−Month Lag]
G1:Roi Et
Dengue Fever
PM2.5 Surface Concentration (µg/m³)
[1−Month Lag]
G2:Roi Et
Dengue Fever
PM2.5 Surface Concentration (µg/m³)
[2−Month Lag]

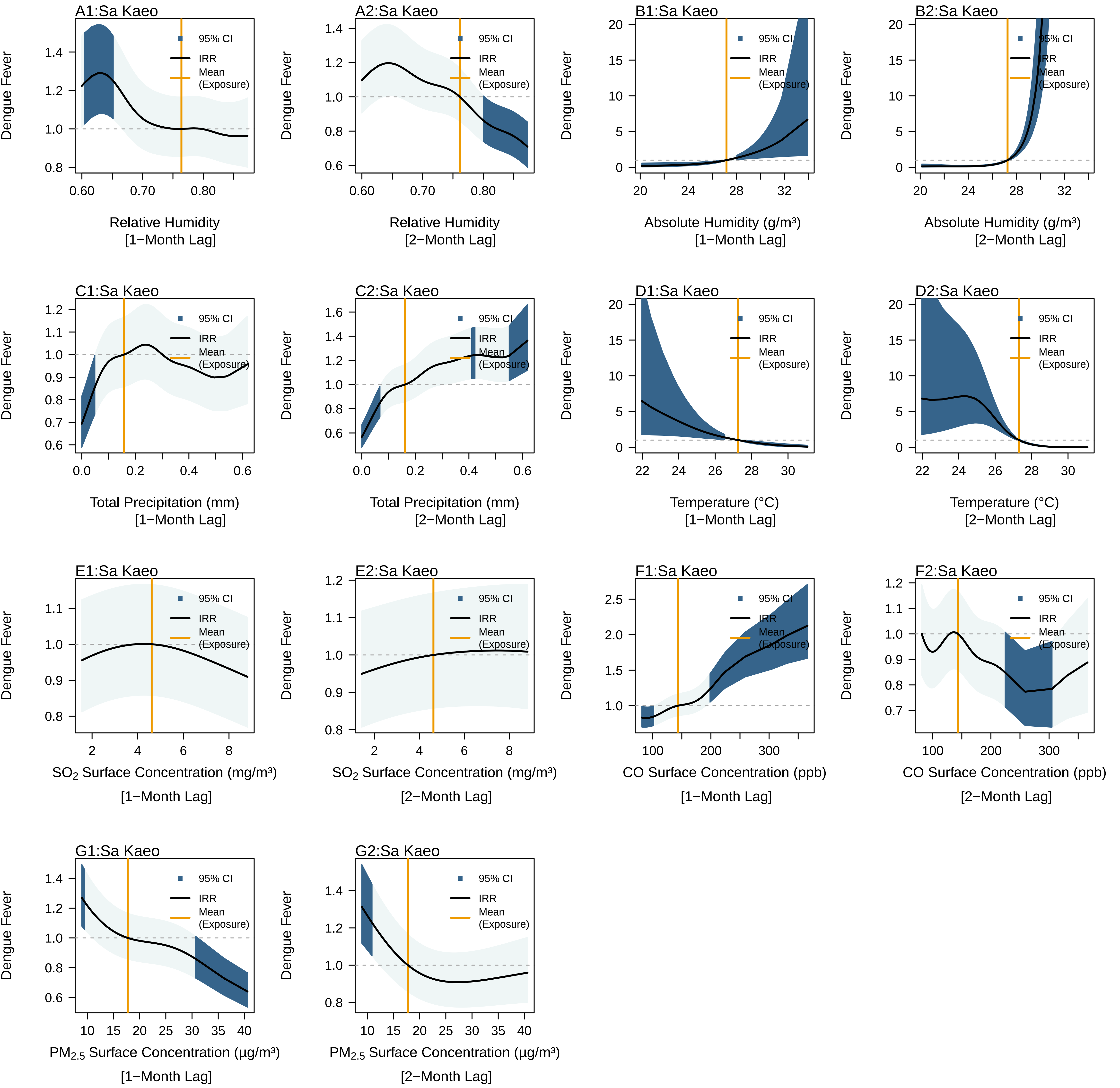
A1:Sa Kaeo
Dengue Fever
95% CI
IRR
Mean
(Exposure)
Relative Humidity
[1−Month Lag]
A2:Sa Kaeo
Relative Humidity
[2−Month Lag]
B1:Sa Kaeo
Absolute Humidity (g/m³)
[1−Month Lag]
B2:Sa Kaeo
Absolute Humidity (g/m³)
[2−Month Lag]
C1:Sa Kaeo
Total Precipitation (mm)
[1−Month Lag]
C2:Sa Kaeo
Total Precipitation (mm)
[2−Month Lag]
D1:Sa Kaeo
Temperature (°C)
[1−Month Lag]
D2:Sa Kaeo
Temperature (°C)
[2−Month Lag]
E1:Sa Kaeo
SO_2 Surface Concentration (mg/m³)
[1−Month Lag]
E2:Sa Kaeo
SO_2 Surface Concentration (mg/m³)
[2−Month Lag]
F1:Sa Kaeo
CO Surface Concentration (ppb)
[1−Month Lag]
F2:Sa Kaeo
CO Surface Concentration (ppb)
[2−Month Lag]
G1:Sa Kaeo
$PM_{2.5}$ Surface Concentration (µg/m³)
[1−Month Lag]
G2:Sa Kaeo
$PM_{2.5}$ Surface Concentration (µg/m³)
[2−Month Lag]

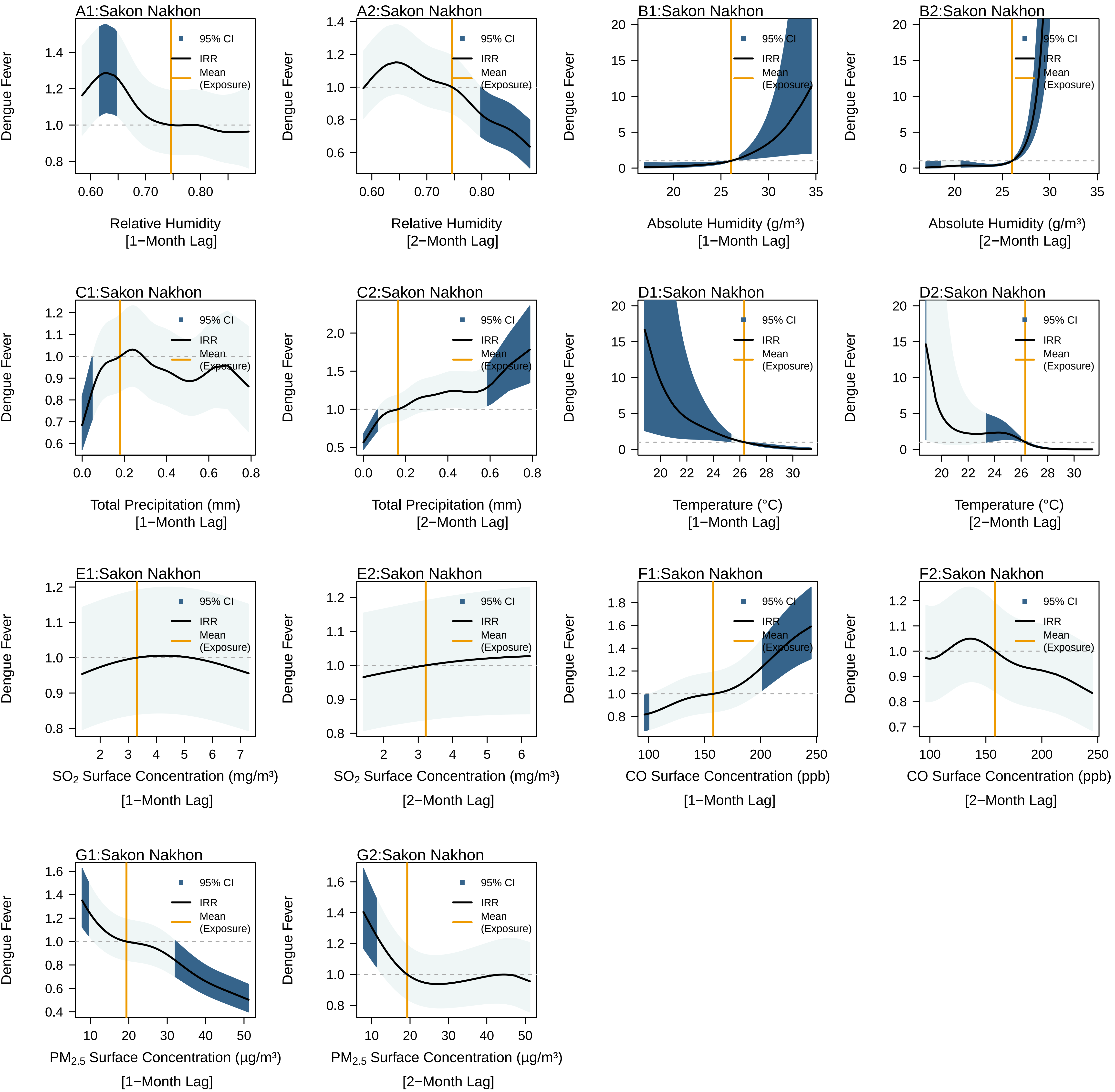
A1:Sakon Nakhon
Dengue Fever
Relative Humidity [1−Month Lag]
A2:Sakon Nakhon
Relative Humidity [2−Month Lag]
B1:Sakon Nakhon
Absolute Humidity (g/m³) [1−Month Lag]
B2:Sakon Nakhon
Absolute Humidity (g/m³) [2−Month Lag]
C1:Sakon Nakhon
Total Precipitation (mm) [1−Month Lag]
C2:Sakon Nakhon
Total Precipitation (mm) [2−Month Lag]
D1:Sakon Nakhon
Temperature (°C) [1−Month Lag]
D2:Sakon Nakhon
Temperature (°C) [2−Month Lag]
E1:Sakon Nakhon
SO_2 Surface Concentration (mg/m³) [1−Month Lag]
E2:Sakon Nakhon
SO_2 Surface Concentration (mg/m³) [2−Month Lag]
F1:Sakon Nakhon
CO Surface Concentration (ppb) [1−Month Lag]
F2:Sakon Nakhon
CO Surface Concentration (ppb) [2−Month Lag]
G1:Sakon Nakhon
$PM_{2.5}$ Surface Concentration (µg/m³) [1−Month Lag]
G2:Sakon Nakhon
$PM_{2.5}$ Surface Concentration (µg/m³) [2−Month Lag]
95% CI
IRR
Mean (Exposure)

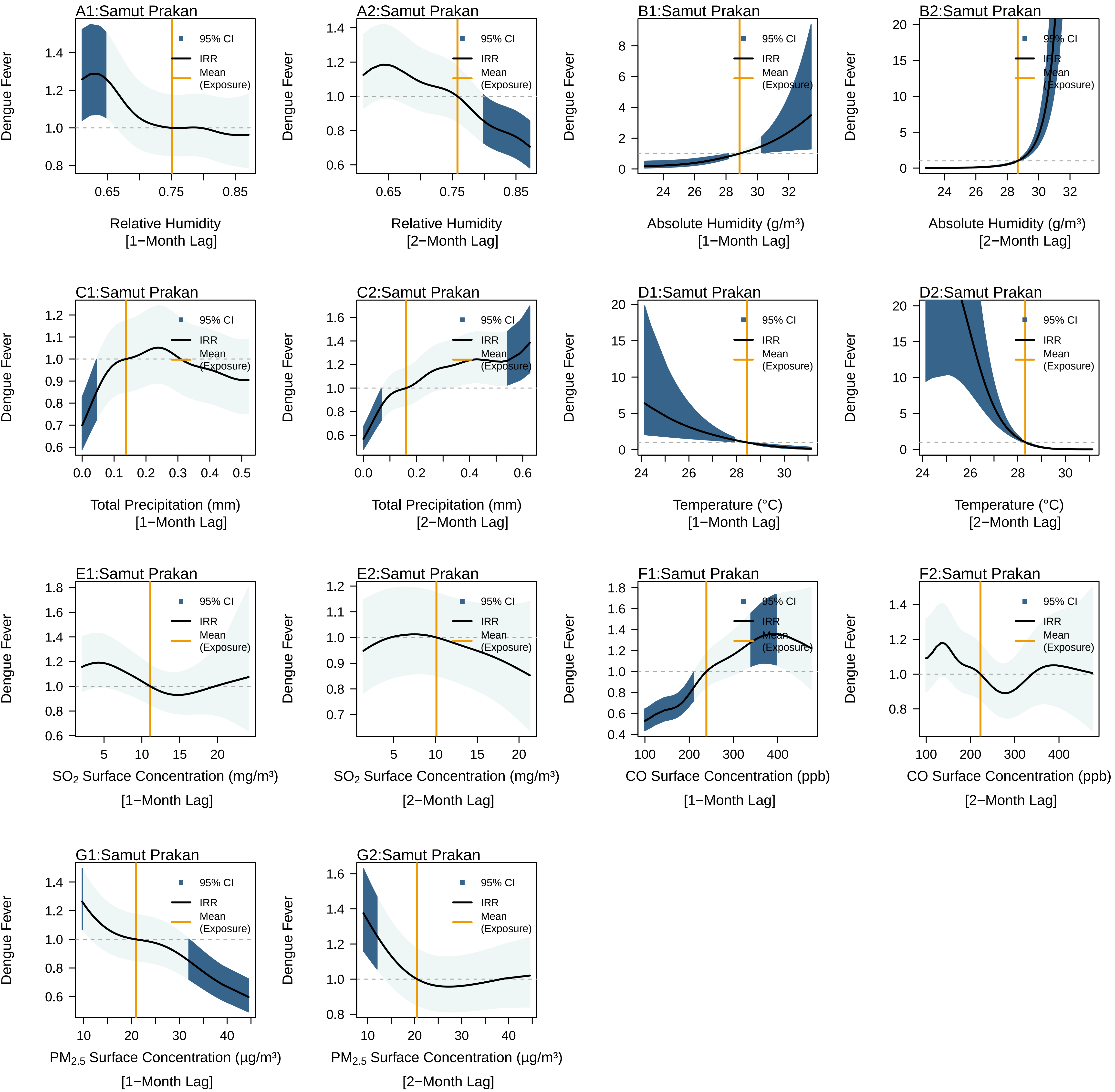
A1:Samut Prakan
Relative Humidity
[1−Month Lag]
A2:Samut Prakan
Relative Humidity
[2−Month Lag]
B1:Samut Prakan
Absolute Humidity (g/m³)
[1−Month Lag]
B2:Samut Prakan
Absolute Humidity (g/m³)
[2−Month Lag]
C1:Samut Prakan
Total Precipitation (mm)
[1−Month Lag]
C2:Samut Prakan
Total Precipitation (mm)
[2−Month Lag]
D1:Samut Prakan
Temperature (°C)
[1−Month Lag]
D2:Samut Prakan
Temperature (°C)
[2−Month Lag]
E1:Samut Prakan
SO_2 Surface Concentration (mg/m³)
[1−Month Lag]
E2:Samut Prakan
SO_2 Surface Concentration (mg/m³)
[2−Month Lag]
F1:Samut Prakan
CO Surface Concentration (ppb)
[1−Month Lag]
F2:Samut Prakan
CO Surface Concentration (ppb)
[2−Month Lag]
G1:Samut Prakan
$PM_{2.5}$ Surface Concentration (µg/m³)
[1−Month Lag]
G2:Samut Prakan
$PM_{2.5}$ Surface Concentration (µg/m³)
[2−Month Lag]
Dengue Fever
95% CI
IRR
Mean
(Exposure)

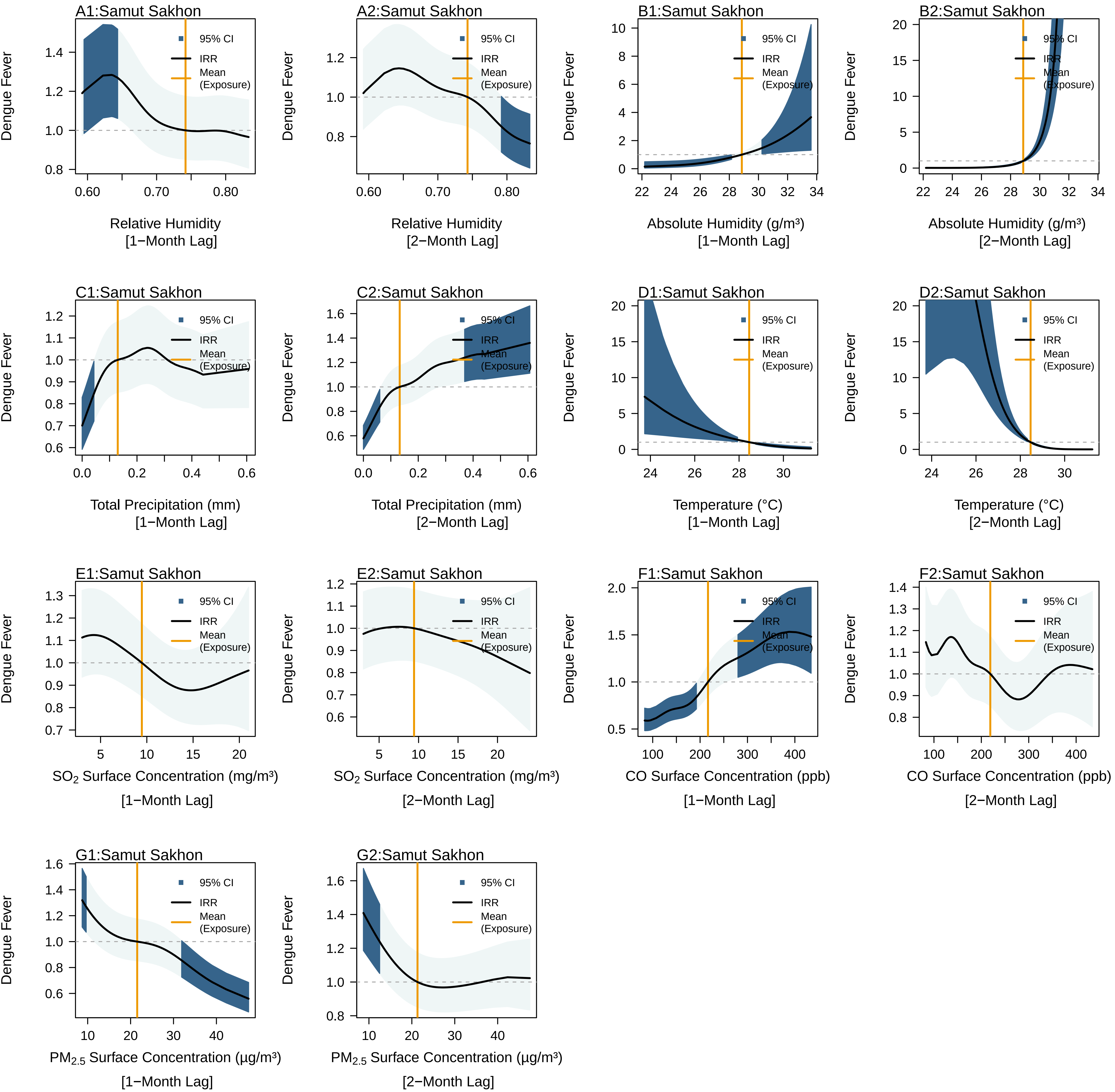
A1:Samut Sakhon
A2:Samut Sakhon
B1:Samut Sakhon
B2:Samut Sakhon
C1:Samut Sakhon
C2:Samut Sakhon
D1:Samut Sakhon
D2:Samut Sakhon
E1:Samut Sakhon
E2:Samut Sakhon
F1:Samut Sakhon
F2:Samut Sakhon
G1:Samut Sakhon
G2:Samut Sakhon
95% CI
IRR
Mean (Exposure)
Dengue Fever
Relative Humidity [1−Month Lag]
Relative Humidity [2−Month Lag]
Absolute Humidity (g/m³) [1−Month Lag]
Absolute Humidity (g/m³) [2−Month Lag]
Total Precipitation (mm) [1−Month Lag]
Total Precipitation (mm) [2−Month Lag]
Temperature (°C) [1−Month Lag]
Temperature (°C) [2−Month Lag]
SO_2 Surface Concentration (mg/m³) [1−Month Lag]
SO_2 Surface Concentration (mg/m³) [2−Month Lag]
CO Surface Concentration (ppb) [1−Month Lag]
CO Surface Concentration (ppb) [2−Month Lag]
$PM_{2.5}$ Surface Concentration (µg/m³) [1−Month Lag]
$PM_{2.5}$ Surface Concentration (µg/m³) [2−Month Lag]

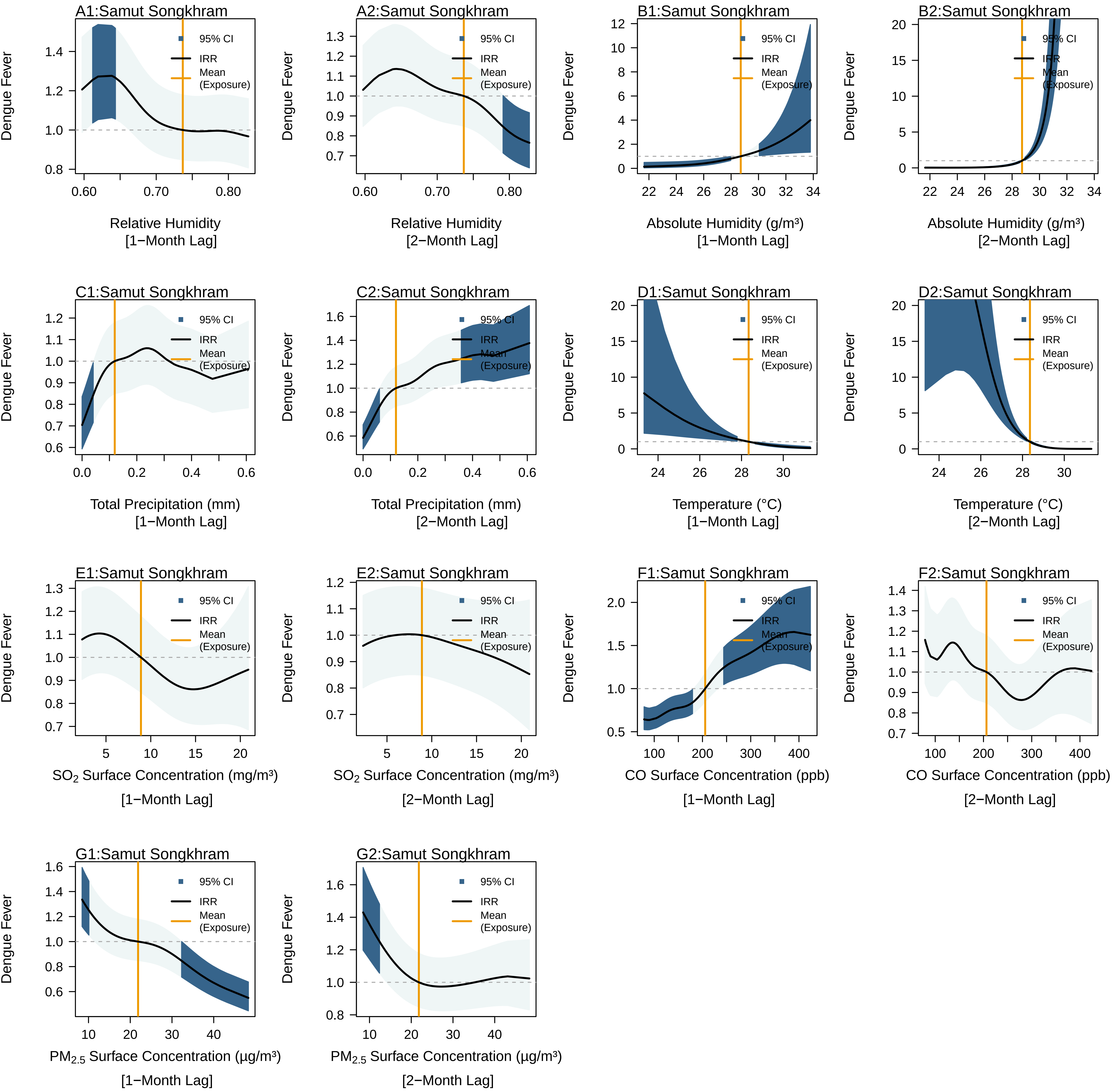
A1:Samut Songkhram
A2:Samut Songkhram
B1:Samut Songkhram
B2:Samut Songkhram
C1:Samut Songkhram
C2:Samut Songkhram
D1:Samut Songkhram
D2:Samut Songkhram
E1:Samut Songkhram
E2:Samut Songkhram
F1:Samut Songkhram
F2:Samut Songkhram
G1:Samut Songkhram
G2:Samut Songkhram
95% CI
IRR
Mean (Exposure)
Dengue Fever
Relative Humidity [1−Month Lag]
Relative Humidity [2−Month Lag]
Absolute Humidity (g/m³) [1−Month Lag]
Absolute Humidity (g/m³) [2−Month Lag]
Total Precipitation (mm) [1−Month Lag]
Total Precipitation (mm) [2−Month Lag]
Temperature (°C) [1−Month Lag]
Temperature (°C) [2−Month Lag]
SO_2 Surface Concentration (mg/m³) [1−Month Lag]
SO_2 Surface Concentration (mg/m³) [2−Month Lag]
CO Surface Concentration (ppb) [1−Month Lag]
CO Surface Concentration (ppb) [2−Month Lag]
$PM_{2.5}$ Surface Concentration (µg/m³) [1−Month Lag]
$PM_{2.5}$ Surface Concentration (µg/m³) [2−Month Lag]

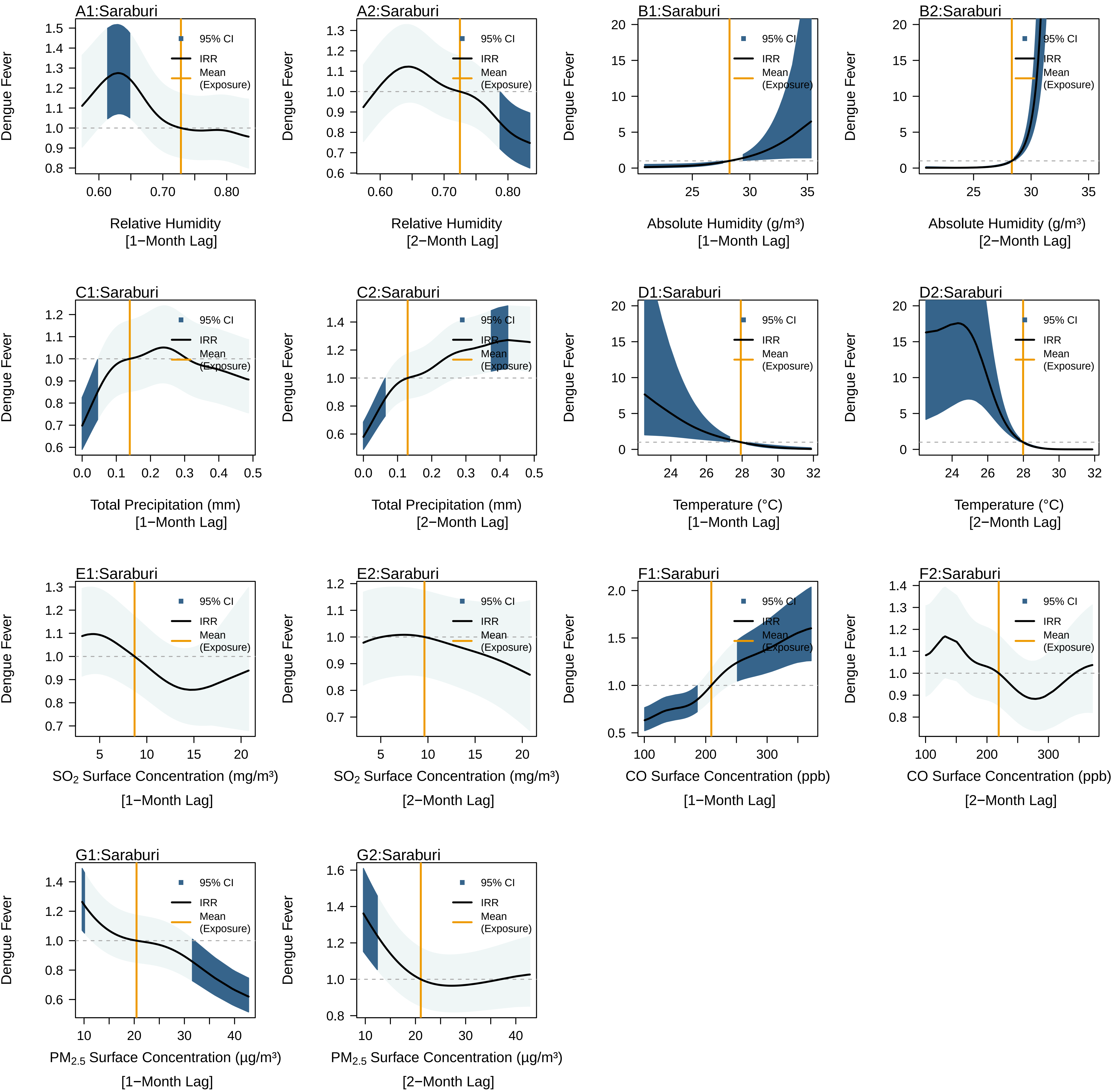
A1:Saraburi
A2:Saraburi
B1:Saraburi
B2:Saraburi
C1:Saraburi
C2:Saraburi
D1:Saraburi
D2:Saraburi
E1:Saraburi
E2:Saraburi
F1:Saraburi
F2:Saraburi
G1:Saraburi
G2:Saraburi
Dengue Fever
95% CI
IRR
Mean (Exposure)
Relative Humidity [1−Month Lag]
Relative Humidity [2−Month Lag]
Absolute Humidity (g/m³) [1−Month Lag]
Absolute Humidity (g/m³) [2−Month Lag]
Total Precipitation (mm) [1−Month Lag]
Total Precipitation (mm) [2−Month Lag]
Temperature (°C) [1−Month Lag]
Temperature (°C) [2−Month Lag]
SO_2 Surface Concentration (mg/m³) [1−Month Lag]
SO_2 Surface Concentration (mg/m³) [2−Month Lag]
CO Surface Concentration (ppb) [1−Month Lag]
CO Surface Concentration (ppb) [2−Month Lag]
$PM_{2.5}$ Surface Concentration (µg/m³) [1−Month Lag]
$PM_{2.5}$ Surface Concentration (µg/m³) [2−Month Lag]

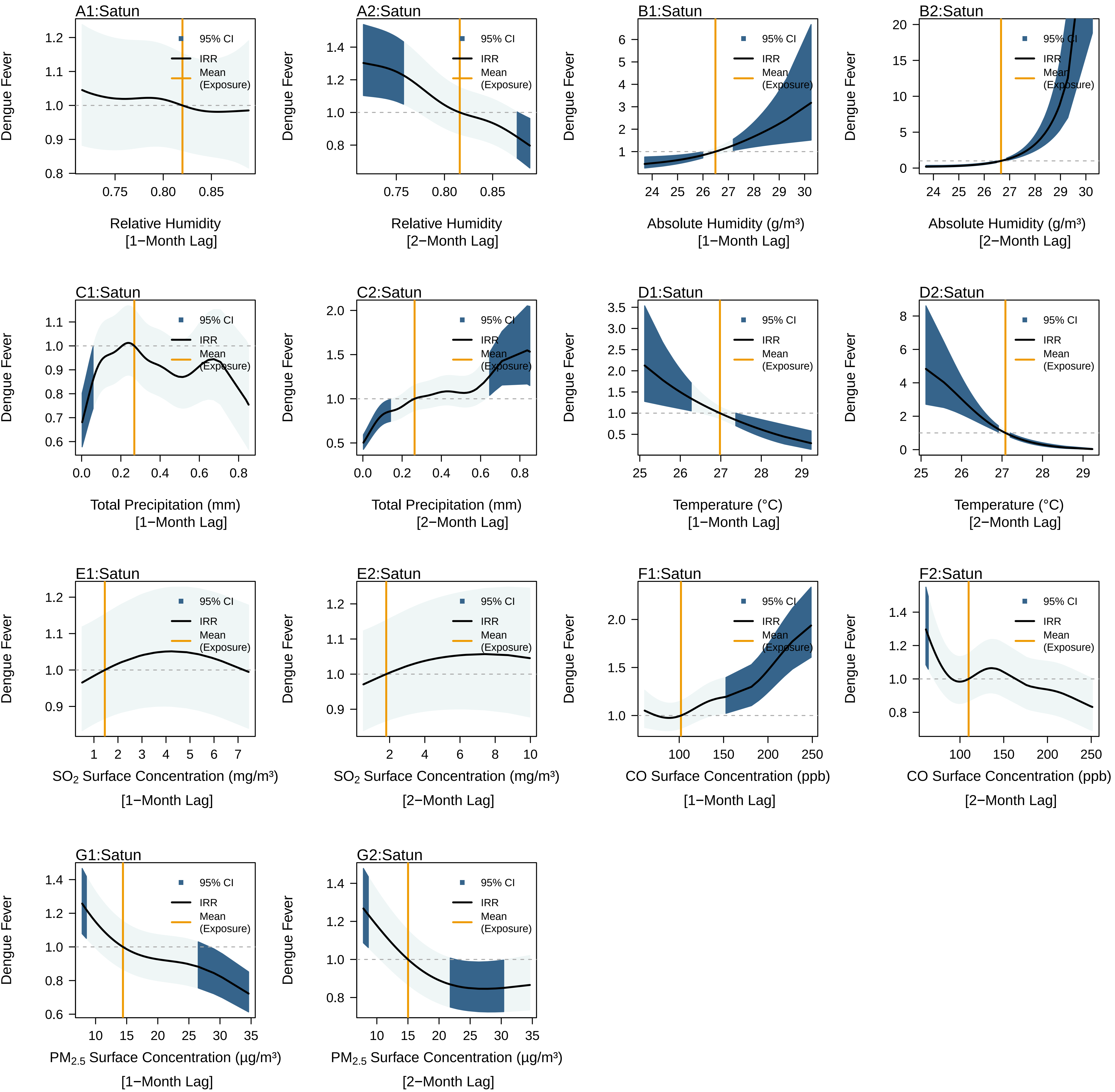
A1:Satun
A2:Satun
B1:Satun
B2:Satun
C1:Satun
C2:Satun
D1:Satun
D2:Satun
E1:Satun
E2:Satun
F1:Satun
F2:Satun
G1:Satun
G2:Satun
95% CI
IRR
Mean
(Exposure)
Dengue Fever
Relative Humidity
[1−Month Lag]
Relative Humidity
[2−Month Lag]
Absolute Humidity (g/m³)
[1−Month Lag]
Absolute Humidity (g/m³)
[2−Month Lag]
Total Precipitation (mm)
[1−Month Lag]
Total Precipitation (mm)
[2−Month Lag]
Temperature (°C)
[1−Month Lag]
Temperature (°C)
[2−Month Lag]
SO_2 Surface Concentration (mg/m³)
[1−Month Lag]
SO_2 Surface Concentration (mg/m³)
[2−Month Lag]
CO Surface Concentration (ppb)
[1−Month Lag]
CO Surface Concentration (ppb)
[2−Month Lag]
$PM_{2.5}$ Surface Concentration (µg/m³)
[1−Month Lag]
$PM_{2.5}$ Surface Concentration (µg/m³)
[2−Month Lag]

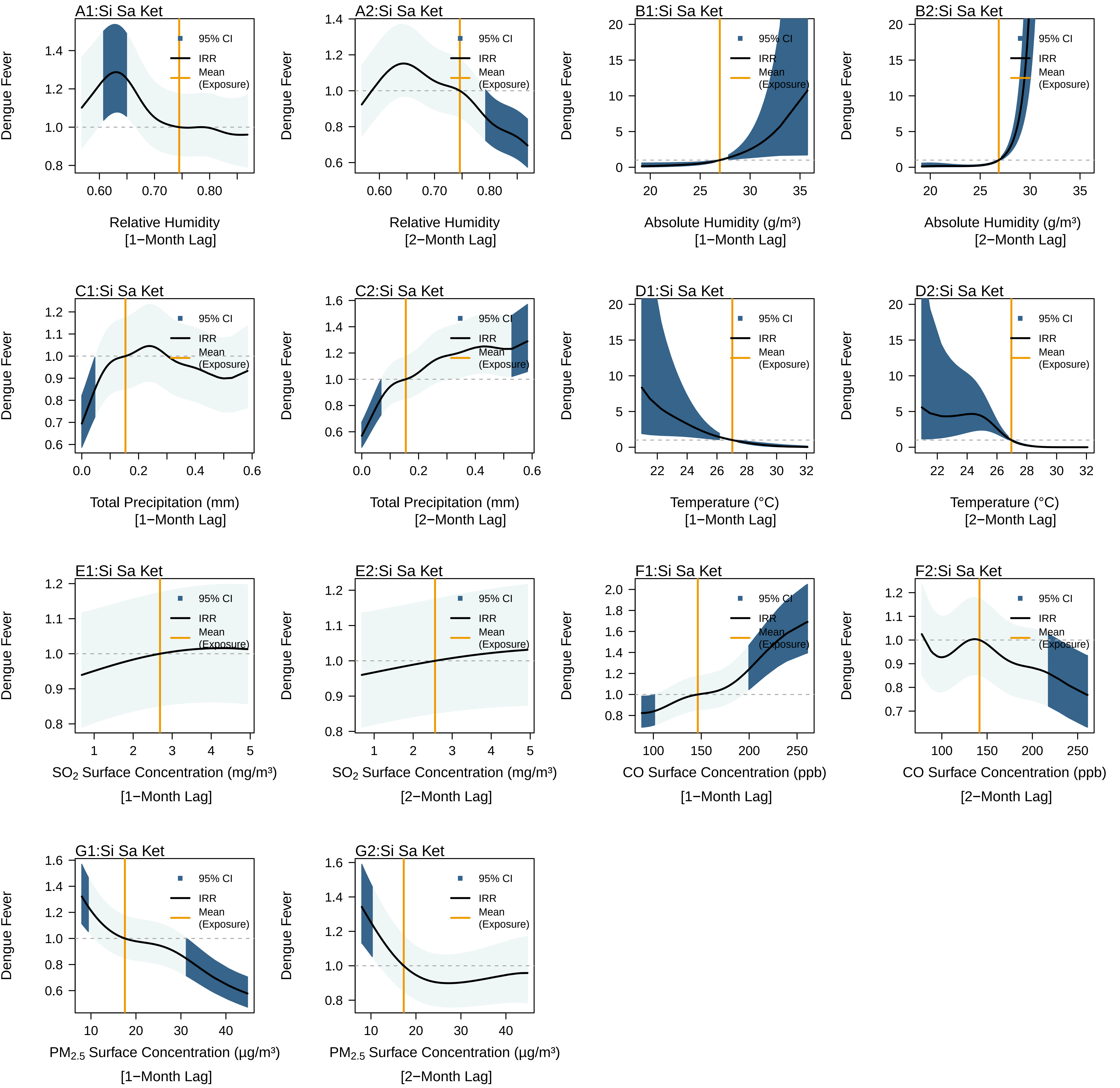
A1:Si Sa Ket
Dengue Fever
95% CI
IRR
Mean
(Exposure)
Relative Humidity
[1−Month Lag]
A2:Si Sa Ket
Dengue Fever
Relative Humidity
[2−Month Lag]
B1:Si Sa Ket
Dengue Fever
Absolute Humidity (g/m³)
[1−Month Lag]
B2:Si Sa Ket
Dengue Fever
Absolute Humidity (g/m³)
[2−Month Lag]
C1:Si Sa Ket
Dengue Fever
Total Precipitation (mm)
[1−Month Lag]
C2:Si Sa Ket
Dengue Fever
Total Precipitation (mm)
[2−Month Lag]
D1:Si Sa Ket
Dengue Fever
Temperature (°C)
[1−Month Lag]
D2:Si Sa Ket
Dengue Fever
Temperature (°C)
[2−Month Lag]
E1:Si Sa Ket
Dengue Fever
SO_2 Surface Concentration (mg/m³)
[1−Month Lag]
E2:Si Sa Ket
Dengue Fever
SO_2 Surface Concentration (mg/m³)
[2−Month Lag]
F1:Si Sa Ket
Dengue Fever
CO Surface Concentration (ppb)
[1−Month Lag]
F2:Si Sa Ket
Dengue Fever
CO Surface Concentration (ppb)
[2−Month Lag]
G1:Si Sa Ket
Dengue Fever
$PM_{2.5}$ Surface Concentration (µg/m³)
[1−Month Lag]
G2:Si Sa Ket
Dengue Fever
$PM_{2.5}$ Surface Concentration (µg/m³)
[2−Month Lag]

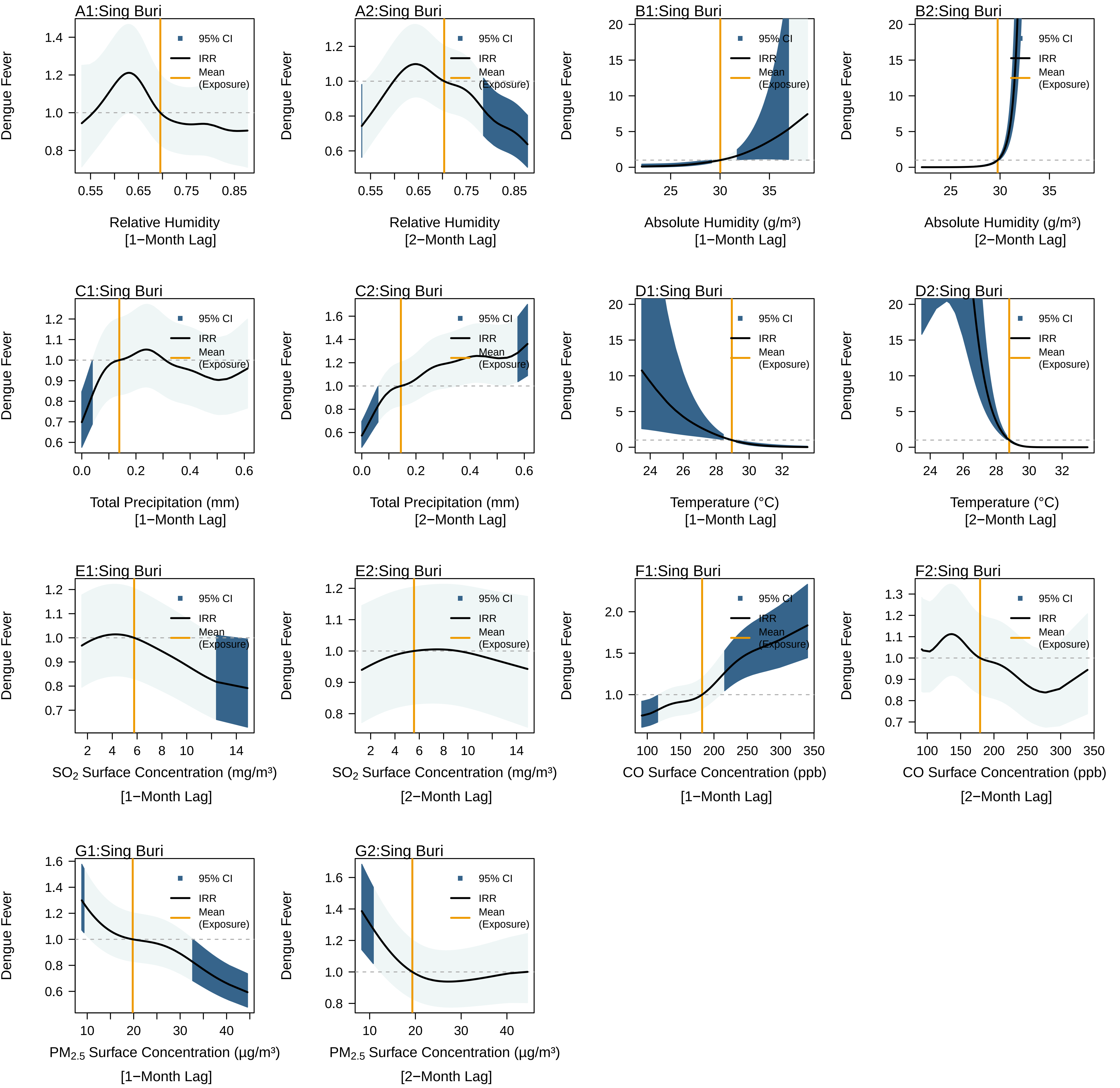

A1:Sing Buri
Dengue Fever
1.4 1.2 1.0 0.8
0.55 0.65 0.75 0.85
95% CI
IRR
Mean
(Exposure)
Relative Humidity
[1−Month Lag]
A2:Sing Buri
Dengue Fever
1.2 1.0 0.8 0.6
0.55 0.65 0.75 0.85
95% CI
IRR
Mean
(Exposure)
Relative Humidity
[2−Month Lag]
B1:Sing Buri
Dengue Fever
20 15 10 5 0
25 30 35
95% CI
IRR
Mean
(Exposure)
Absolute Humidity (g/m³)
[1−Month Lag]
B2:Sing Buri
Dengue Fever
20 15 10 5 0
25 30 35
95% CI
IRR
Mean
(Exposure)
Absolute Humidity (g/m³)
[2−Month Lag]
C1:Sing Buri
Dengue Fever
1.2 1.1 1.0 0.9 0.8 0.7 0.6
0.0 0.2 0.4 0.6
95% CI
IRR
Mean
(Exposure)
Total Precipitation (mm)
[1−Month Lag]
C2:Sing Buri
Dengue Fever
1.6 1.4 1.2 1.0 0.8 0.6
0.0 0.2 0.4 0.6
95% CI
IRR
Mean
(Exposure)
Total Precipitation (mm)
[2−Month Lag]
D1:Sing Buri
Dengue Fever
20 15 10 5 0
24 26 28 30 32
95% CI
IRR
Mean
(Exposure)
Temperature (°C)
[1−Month Lag]
D2:Sing Buri
Dengue Fever
20 15 10 5 0
24 26 28 30 32
95% CI
IRR
Mean
(Exposure)
Temperature (°C)
[2−Month Lag]
E1:Sing Buri
Dengue Fever
1.2 1.1 1.0 0.9 0.8 0.7
2 4 6 8 10 14
95% CI
IRR
Mean
(Exposure)
SO2 Surface Concentration (mg/m³)
[1−Month Lag]
E2:Sing Buri
Dengue Fever
1.2 1.1 1.0 0.9 0.8
2 4 6 8 10 14
95% CI
IRR
Mean
(Exposure)
SO2 Surface Concentration (mg/m³)
[2−Month Lag]
F1:Sing Buri
Dengue Fever
2.0 1.5 1.0
100 150 200 250 300 350
95% CI
IRR
Mean
(Exposure)
CO Surface Concentration (ppb)
[1−Month Lag]
F2:Sing Buri
Dengue Fever
1.3 1.2 1.1 1.0 0.9 0.8 0.7
100 150 200 250 300 350
95% CI
IRR
Mean
(Exposure)
CO Surface Concentration (ppb)
[2−Month Lag]
G1:Sing Buri
Dengue Fever
1.6 1.4 1.2 1.0 0.8 0.6
10 20 30 40
95% CI
IRR
Mean
(Exposure)
PM2.5 Surface Concentration (µg/m³)
[1−Month Lag]
G2:Sing Buri
Dengue Fever
1.6 1.4 1.2 1.0 0.8
10 20 30 40
95% CI
IRR
Mean
(Exposure)
PM2.5 Surface Concentration (µg/m³)
[2−Month Lag]

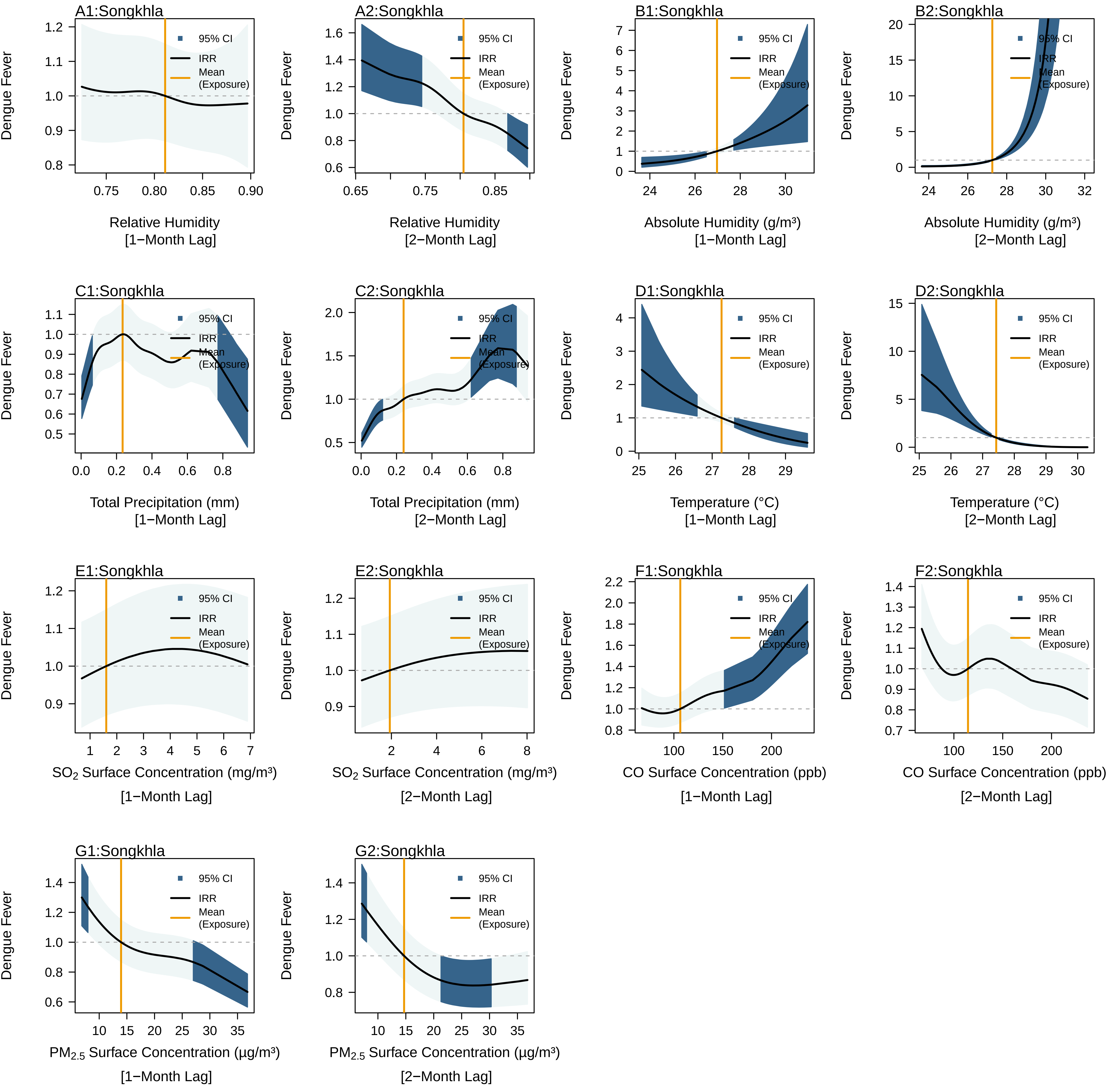
A1:Songkhla
Dengue Fever
95% CI
IRR
Mean (Exposure)
Relative Humidity [1−Month Lag]
A2:Songkhla
Relative Humidity [2−Month Lag]
B1:Songkhla
Absolute Humidity (g/m³) [1−Month Lag]
B2:Songkhla
Absolute Humidity (g/m³) [2−Month Lag]
C1:Songkhla
Total Precipitation (mm) [1−Month Lag]
C2:Songkhla
Total Precipitation (mm) [2−Month Lag]
D1:Songkhla
Temperature (°C) [1−Month Lag]
D2:Songkhla
Temperature (°C) [2−Month Lag]
E1:Songkhla
SO_2 Surface Concentration (mg/m³) [1−Month Lag]
E2:Songkhla
SO_2 Surface Concentration (mg/m³) [2−Month Lag]
F1:Songkhla
CO Surface Concentration (ppb) [1−Month Lag]
F2:Songkhla
CO Surface Concentration (ppb) [2−Month Lag]
G1:Songkhla
$PM_{2.5}$ Surface Concentration (µg/m³) [1−Month Lag]
G2:Songkhla
$PM_{2.5}$ Surface Concentration (µg/m³) [2−Month Lag]

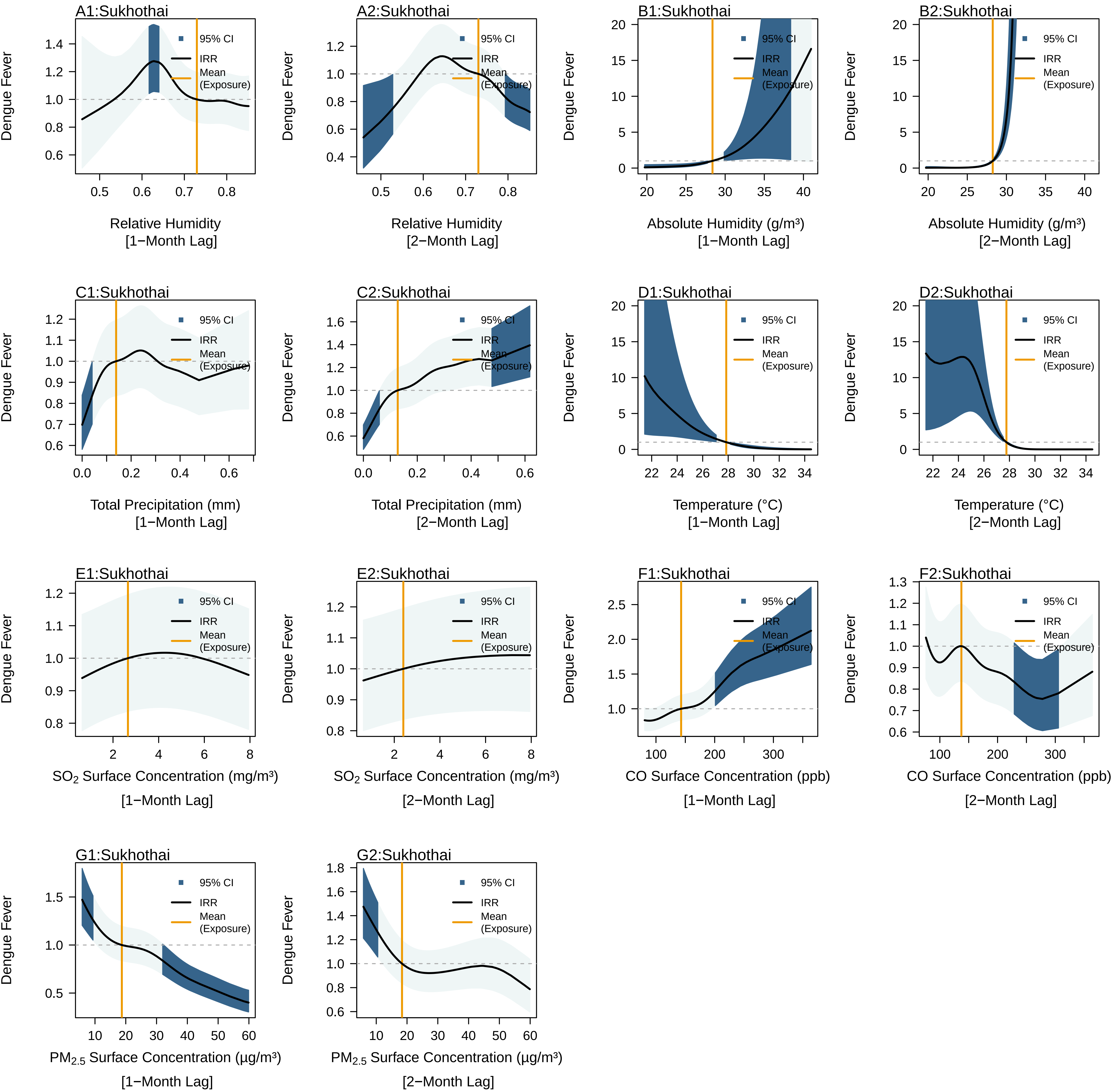

A1:Sukhothai
Dengue Fever
95% CI
IRR
Mean (Exposure)
Relative Humidity [1−Month Lag]
A2:Sukhothai
Dengue Fever
95% CI
IRR
Mean (Exposure)
Relative Humidity [2−Month Lag]
B1:Sukhothai
Dengue Fever
95% CI
IRR
Mean (Exposure)
Absolute Humidity (g/m³) [1−Month Lag]
B2:Sukhothai
Dengue Fever
95% CI
IRR
Mean (Exposure)
Absolute Humidity (g/m³) [2−Month Lag]
C1:Sukhothai
Dengue Fever
95% CI
IRR
Mean (Exposure)
Total Precipitation (mm) [1−Month Lag]
C2:Sukhothai
Dengue Fever
95% CI
IRR
Mean (Exposure)
Total Precipitation (mm) [2−Month Lag]
D1:Sukhothai
Dengue Fever
95% CI
IRR
Mean (Exposure)
Temperature (°C) [1−Month Lag]
D2:Sukhothai
Dengue Fever
95% CI
IRR
Mean (Exposure)
Temperature (°C) [2−Month Lag]
E1:Sukhothai
Dengue Fever
95% CI
IRR
Mean (Exposure)
SO_2 Surface Concentration (mg/m³) [1−Month Lag]
E2:Sukhothai
Dengue Fever
95% CI
IRR
Mean (Exposure)
SO_2 Surface Concentration (mg/m³) [2−Month Lag]
F1:Sukhothai
Dengue Fever
95% CI
IRR
Mean (Exposure)
CO Surface Concentration (ppb) [1−Month Lag]
F2:Sukhothai
Dengue Fever
95% CI
IRR
Mean (Exposure)
CO Surface Concentration (ppb) [2−Month Lag]
G1:Sukhothai
Dengue Fever
95% CI
IRR
Mean (Exposure)
$PM_{2.5}$ Surface Concentration (µg/m³) [1−Month Lag]
G2:Sukhothai
Dengue Fever
95% CI
IRR
Mean (Exposure)
$PM_{2.5}$ Surface Concentration (µg/m³) [2−Month Lag]

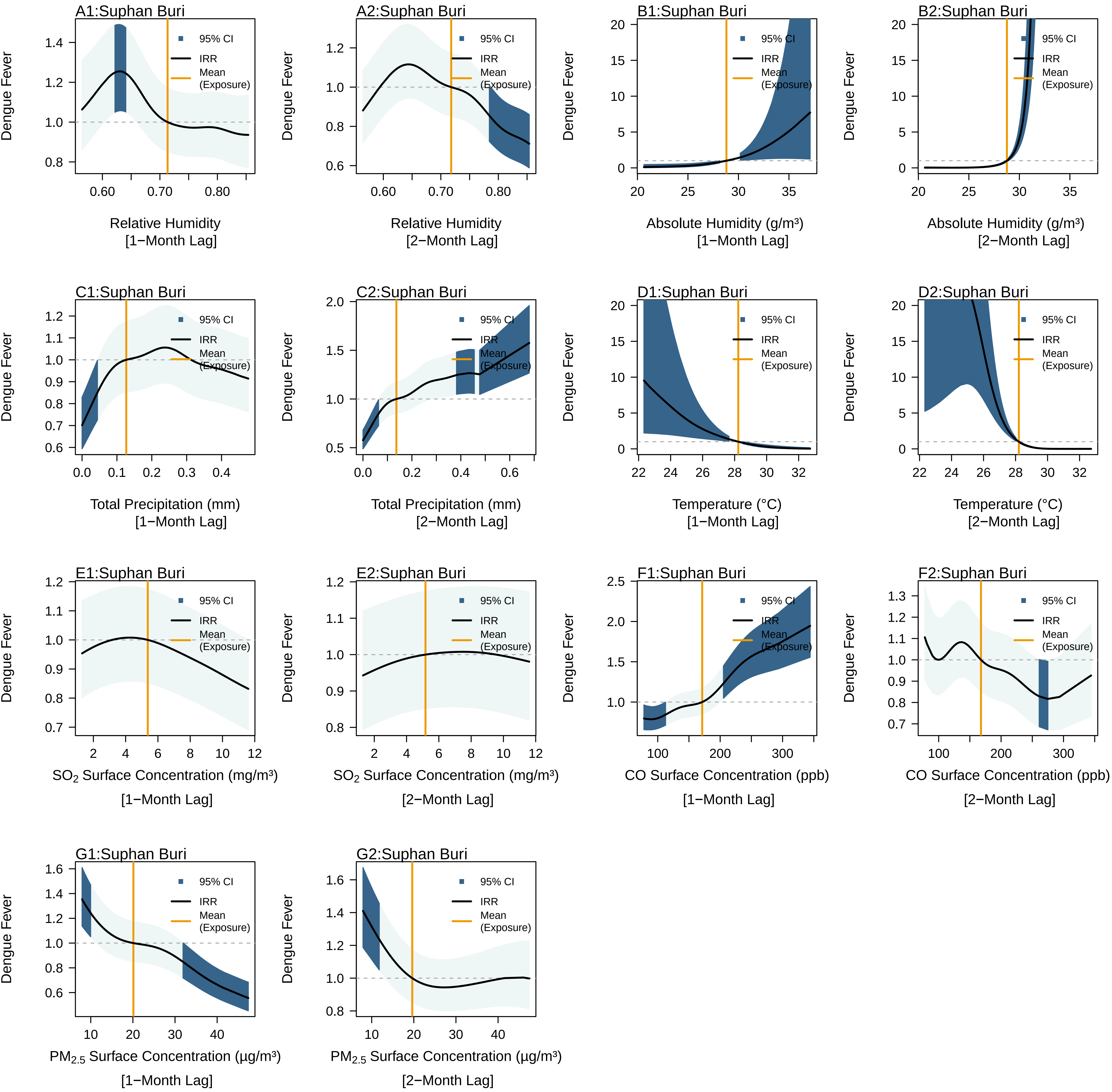
A1:Suphan Buri
Dengue Fever
95% CI
IRR
Mean (Exposure)
Relative Humidity [1−Month Lag]
A2:Suphan Buri
Dengue Fever
95% CI
IRR
Mean (Exposure)
Relative Humidity [2−Month Lag]
B1:Suphan Buri
Dengue Fever
95% CI
IRR
Mean (Exposure)
Absolute Humidity (g/m³) [1−Month Lag]
B2:Suphan Buri
Dengue Fever
95% CI
IRR
Mean (Exposure)
Absolute Humidity (g/m³) [2−Month Lag]
C1:Suphan Buri
Dengue Fever
95% CI
IRR
Mean (Exposure)
Total Precipitation (mm) [1−Month Lag]
C2:Suphan Buri
Dengue Fever
95% CI
IRR
Mean (Exposure)
Total Precipitation (mm) [2−Month Lag]
D1:Suphan Buri
Dengue Fever
95% CI
IRR
Mean (Exposure)
Temperature (°C) [1−Month Lag]
D2:Suphan Buri
Dengue Fever
95% CI
IRR
Mean (Exposure)
Temperature (°C) [2−Month Lag]
E1:Suphan Buri
Dengue Fever
95% CI
IRR
Mean (Exposure)
SO_2 Surface Concentration (mg/m³) [1−Month Lag]
E2:Suphan Buri
Dengue Fever
95% CI
IRR
Mean (Exposure)
SO_2 Surface Concentration (mg/m³) [2−Month Lag]
F1:Suphan Buri
Dengue Fever
95% CI
IRR
Mean (Exposure)
CO Surface Concentration (ppb) [1−Month Lag]
F2:Suphan Buri
Dengue Fever
95% CI
IRR
Mean (Exposure)
CO Surface Concentration (ppb) [2−Month Lag]
G1:Suphan Buri
Dengue Fever
95% CI
IRR
Mean (Exposure)
$PM_{2.5}$ Surface Concentration (µg/m³) [1−Month Lag]
G2:Suphan Buri
Dengue Fever
95% CI
IRR
Mean (Exposure)
$PM_{2.5}$ Surface Concentration (µg/m³) [2−Month Lag]

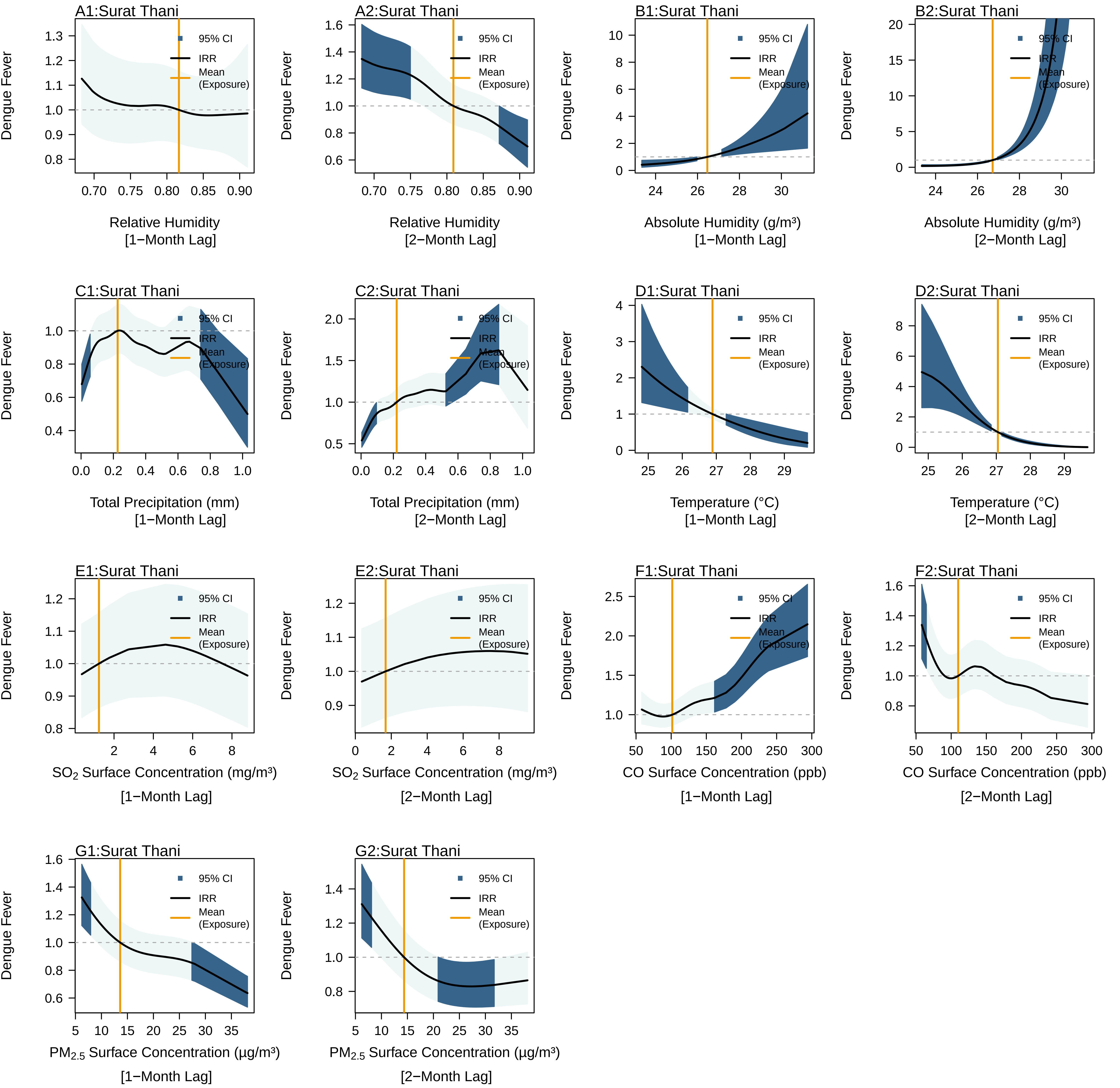

A1:Surat Thani
A2:Surat Thani
B1:Surat Thani
B2:Surat Thani
C1:Surat Thani
C2:Surat Thani
D1:Surat Thani
D2:Surat Thani
E1:Surat Thani
E2:Surat Thani
F1:Surat Thani
F2:Surat Thani
G1:Surat Thani
G2:Surat Thani
Dengue Fever
95% CI
IRR
Mean (Exposure)
Relative Humidity [1−Month Lag]
Relative Humidity [2−Month Lag]
Absolute Humidity (g/m³) [1−Month Lag]
Absolute Humidity (g/m³) [2−Month Lag]
Total Precipitation (mm) [1−Month Lag]
Total Precipitation (mm) [2−Month Lag]
Temperature (°C) [1−Month Lag]
Temperature (°C) [2−Month Lag]
SO2 Surface Concentration (mg/m³) [1−Month Lag]
SO2 Surface Concentration (mg/m³) [2−Month Lag]
CO Surface Concentration (ppb) [1−Month Lag]
CO Surface Concentration (ppb) [2−Month Lag]
PM2.5 Surface Concentration (μg/m³) [1−Month Lag]
PM2.5 Surface Concentration (μg/m³) [2−Month Lag]

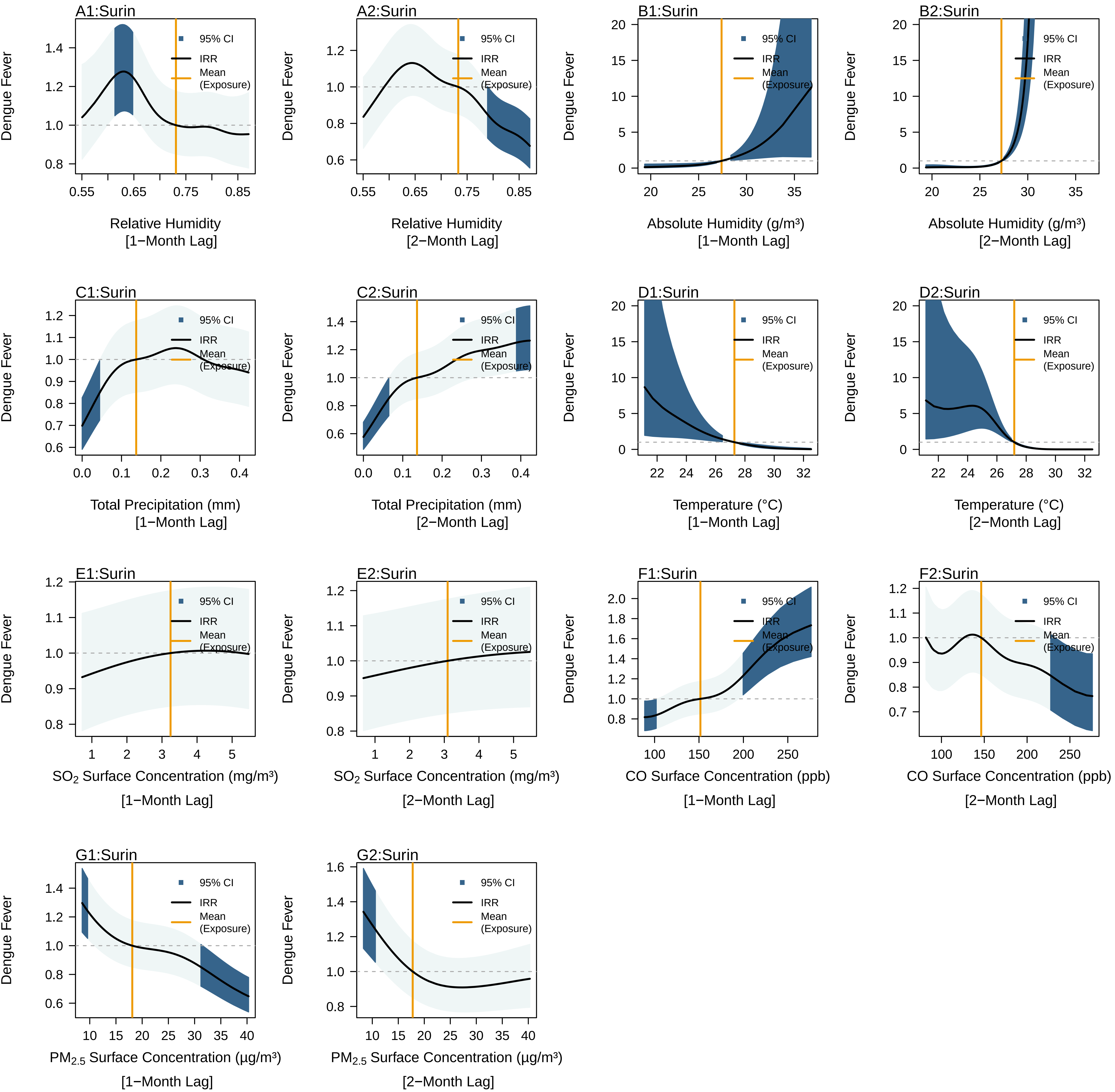
A1:Surin
95% CI
IRR
Mean
(Exposure)
Dengue Fever
Relative Humidity
[1−Month Lag]
A2:Surin
Relative Humidity
[2−Month Lag]
B1:Surin
Absolute Humidity (g/m³)
[1−Month Lag]
B2:Surin
Absolute Humidity (g/m³)
[2−Month Lag]
C1:Surin
Total Precipitation (mm)
[1−Month Lag]
C2:Surin
Total Precipitation (mm)
[2−Month Lag]
D1:Surin
Temperature (°C)
[1−Month Lag]
D2:Surin
Temperature (°C)
[2−Month Lag]
E1:Surin
SO_2 Surface Concentration (mg/m³)
[1−Month Lag]
E2:Surin
SO_2 Surface Concentration (mg/m³)
[2−Month Lag]
F1:Surin
CO Surface Concentration (ppb)
[1−Month Lag]
F2:Surin
CO Surface Concentration (ppb)
[2−Month Lag]
G1:Surin
$PM_{2.5}$ Surface Concentration (µg/m³)
[1−Month Lag]
G2:Surin
$PM_{2.5}$ Surface Concentration (µg/m³)
[2−Month Lag]

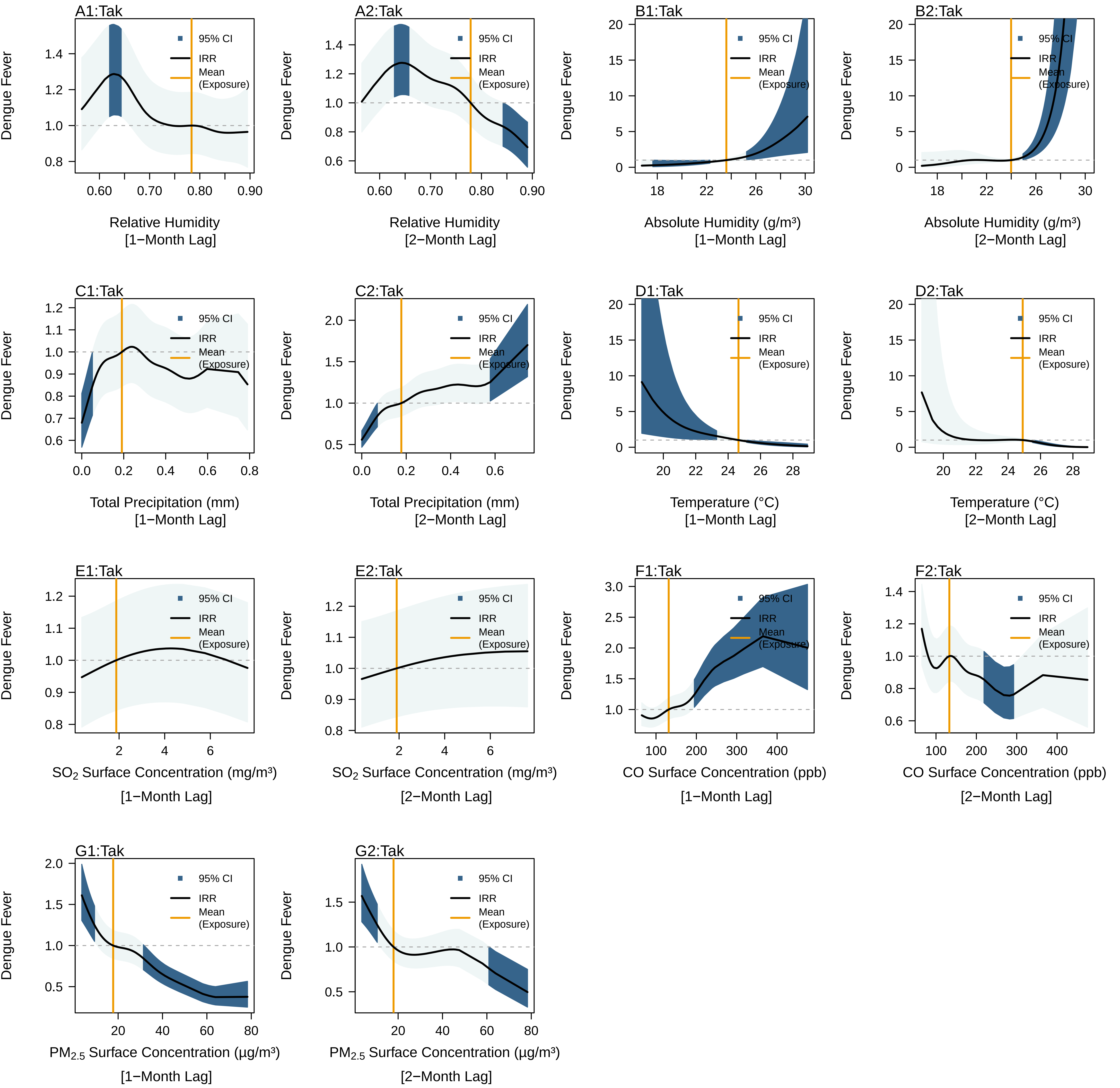
A1:Tak
A2:Tak
B1:Tak
B2:Tak
C1:Tak
C2:Tak
D1:Tak
D2:Tak
E1:Tak
E2:Tak
F1:Tak
F2:Tak
G1:Tak
G2:Tak
95% CI
IRR
Mean
(Exposure)
Dengue Fever
Relative Humidity
[1−Month Lag]
[2−Month Lag]
Absolute Humidity (g/m³)
Total Precipitation (mm)
Temperature (°C)
SO2 Surface Concentration (mg/m³)
CO Surface Concentration (ppb)
PM2.5 Surface Concentration (µg/m³)

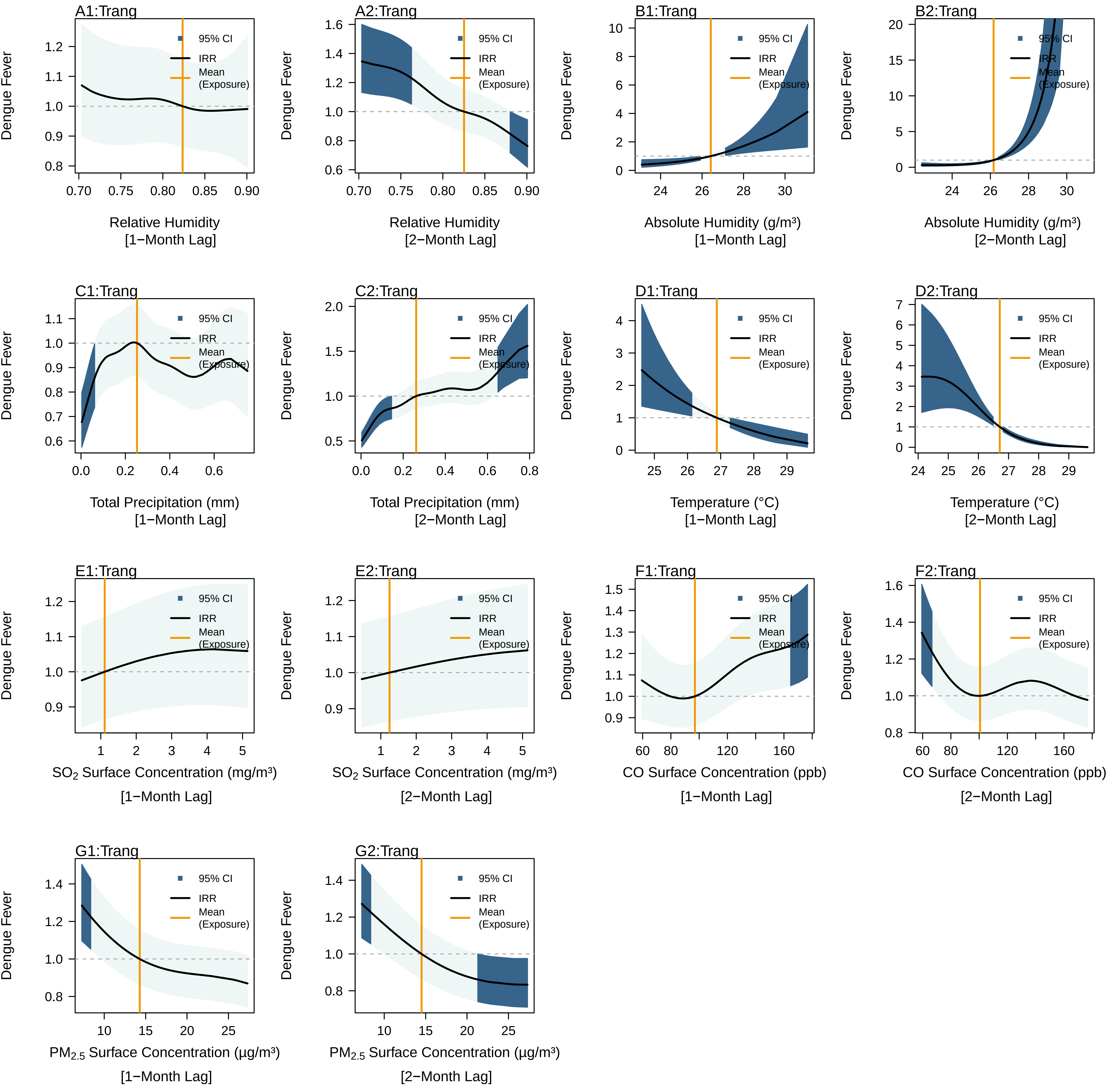
A1:Trang
Dengue Fever
95% CI
IRR
Mean
(Exposure)
Relative Humidity
[1−Month Lag]
A2:Trang
Dengue Fever
Relative Humidity
[2−Month Lag]
B1:Trang
Dengue Fever
Absolute Humidity (g/m³)
[1−Month Lag]
B2:Trang
Dengue Fever
Absolute Humidity (g/m³)
[2−Month Lag]
C1:Trang
Dengue Fever
Total Precipitation (mm)
[1−Month Lag]
C2:Trang
Dengue Fever
Total Precipitation (mm)
[2−Month Lag]
D1:Trang
Dengue Fever
Temperature (°C)
[1−Month Lag]
D2:Trang
Dengue Fever
Temperature (°C)
[2−Month Lag]
E1:Trang
Dengue Fever
SO2 Surface Concentration (mg/m³)
[1−Month Lag]
E2:Trang
Dengue Fever
SO2 Surface Concentration (mg/m³)
[2−Month Lag]
F1:Trang
Dengue Fever
CO Surface Concentration (ppb)
[1−Month Lag]
F2:Trang
Dengue Fever
CO Surface Concentration (ppb)
[2−Month Lag]
G1:Trang
Dengue Fever
PM2.5 Surface Concentration (µg/m³)
[1−Month Lag]
G2:Trang
Dengue Fever
PM2.5 Surface Concentration (µg/m³)
[2−Month Lag]

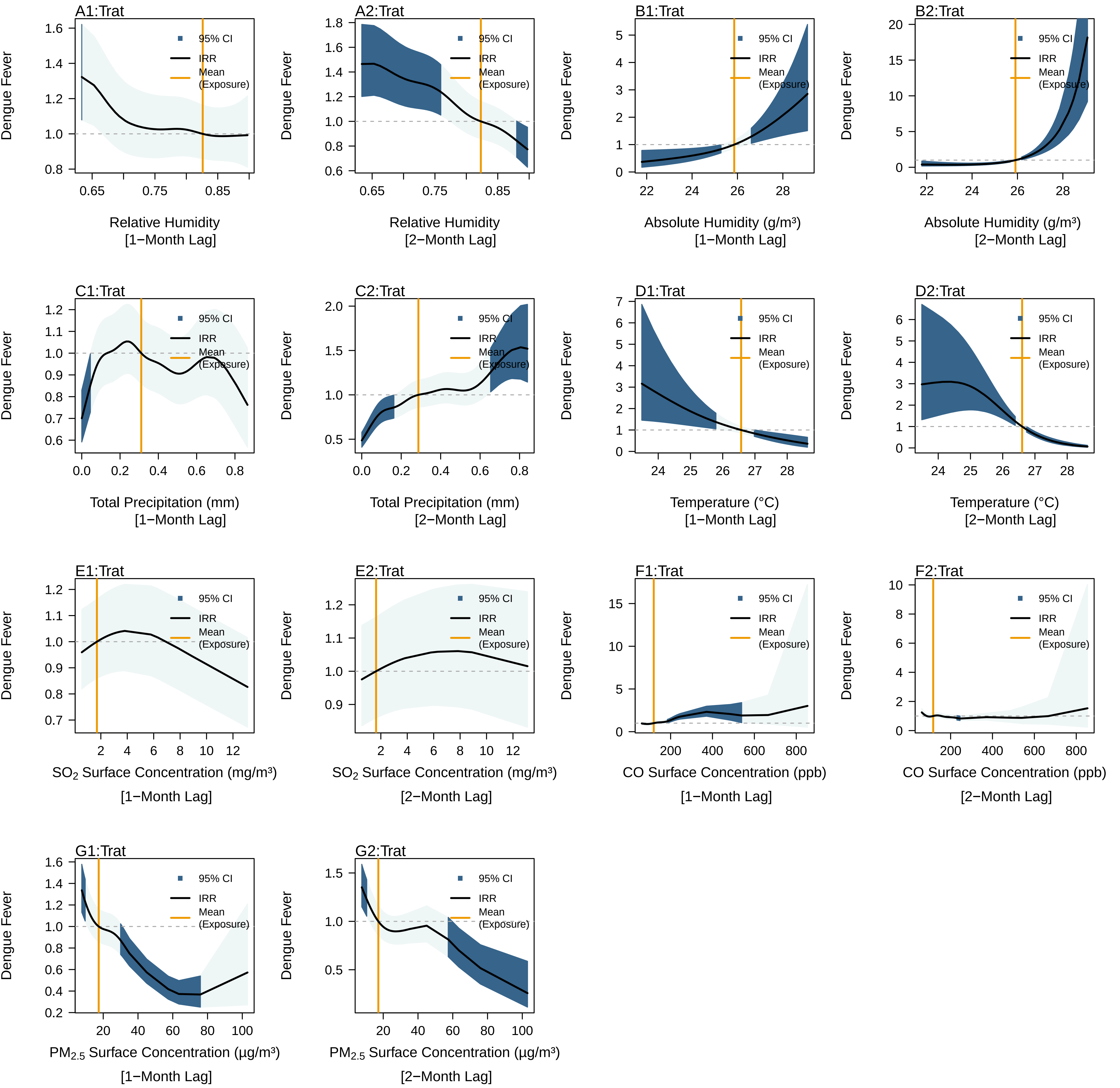
A1:Trat
Dengue Fever
95% CI
IRR
Mean (Exposure)
Relative Humidity [1−Month Lag]
A2:Trat
Dengue Fever
95% CI
IRR
Mean (Exposure)
Relative Humidity [2−Month Lag]
B1:Trat
Dengue Fever
95% CI
IRR
Mean (Exposure)
Absolute Humidity (g/m³) [1−Month Lag]
B2:Trat
Dengue Fever
95% CI
IRR
Mean (Exposure)
Absolute Humidity (g/m³) [2−Month Lag]
C1:Trat
Dengue Fever
95% CI
IRR
Mean (Exposure)
Total Precipitation (mm) [1−Month Lag]
C2:Trat
Dengue Fever
95% CI
IRR
Mean (Exposure)
Total Precipitation (mm) [2−Month Lag]
D1:Trat
Dengue Fever
95% CI
IRR
Mean (Exposure)
Temperature (°C) [1−Month Lag]
D2:Trat
Dengue Fever
95% CI
IRR
Mean (Exposure)
Temperature (°C) [2−Month Lag]
E1:Trat
Dengue Fever
95% CI
IRR
Mean (Exposure)
SO2 Surface Concentration (mg/m³) [1−Month Lag]
E2:Trat
Dengue Fever
95% CI
IRR
Mean (Exposure)
SO2 Surface Concentration (mg/m³) [2−Month Lag]
F1:Trat
Dengue Fever
95% CI
IRR
Mean (Exposure)
CO Surface Concentration (ppb) [1−Month Lag]
F2:Trat
Dengue Fever
95% CI
IRR
Mean (Exposure)
CO Surface Concentration (ppb) [2−Month Lag]
G1:Trat
Dengue Fever
95% CI
IRR
Mean (Exposure)
PM2.5 Surface Concentration (µg/m³) [1−Month Lag]
G2:Trat
Dengue Fever
95% CI
IRR
Mean (Exposure)
PM2.5 Surface Concentration (µg/m³) [2−Month Lag]

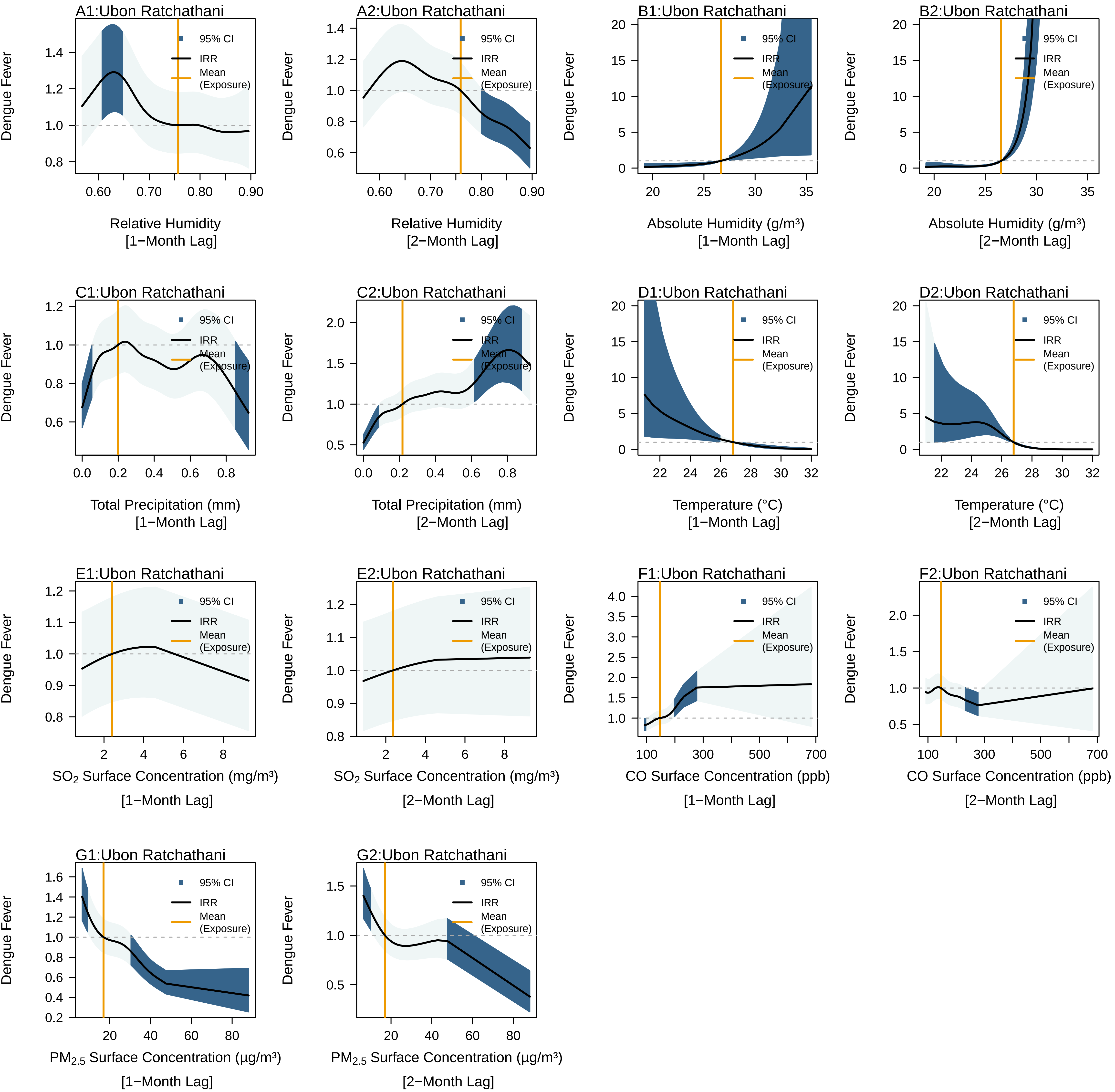
A1:Ubon Ratchathani
Dengue Fever
95% CI
IRR
Mean (Exposure)
0.8 1.0 1.2 1.4
0.60 0.70 0.80 0.90
Relative Humidity [1−Month Lag]
A2:Ubon Ratchathani
0.6 0.8 1.0 1.2 1.4
0.60 0.70 0.80 0.90
Relative Humidity [2−Month Lag]
B1:Ubon Ratchathani
0 5 10 15 20
20 25 30 35
Absolute Humidity (g/m³) [1−Month Lag]
B2:Ubon Ratchathani
Absolute Humidity (g/m³) [2−Month Lag]
C1:Ubon Ratchathani
0.6 0.8 1.0 1.2
0.0 0.2 0.4 0.6 0.8
Total Precipitation (mm) [1−Month Lag]
C2:Ubon Ratchathani
0.5 1.0 1.5 2.0
Total Precipitation (mm) [2−Month Lag]
D1:Ubon Ratchathani
22 24 26 28 30 32
Temperature (°C) [1−Month Lag]
D2:Ubon Ratchathani
Temperature (°C) [2−Month Lag]
E1:Ubon Ratchathani
0.8 0.9 1.0 1.1 1.2
2 4 6 8
SO2 Surface Concentration (mg/m³) [1−Month Lag]
E2:Ubon Ratchathani
SO2 Surface Concentration (mg/m³) [2−Month Lag]
F1:Ubon Ratchathani
1.0 1.5 2.0 2.5 3.0 3.5 4.0
100 300 500 700
CO Surface Concentration (ppb) [1−Month Lag]
F2:Ubon Ratchathani
0.5 1.0 1.5 2.0
CO Surface Concentration (ppb) [2−Month Lag]
G1:Ubon Ratchathani
0.2 0.4 0.6 0.8 1.0 1.2 1.4 1.6
20 40 60 80
PM2.5 Surface Concentration (µg/m³) [1−Month Lag]
G2:Ubon Ratchathani
0.5 1.0 1.5
PM2.5 Surface Concentration (µg/m³) [2−Month Lag]

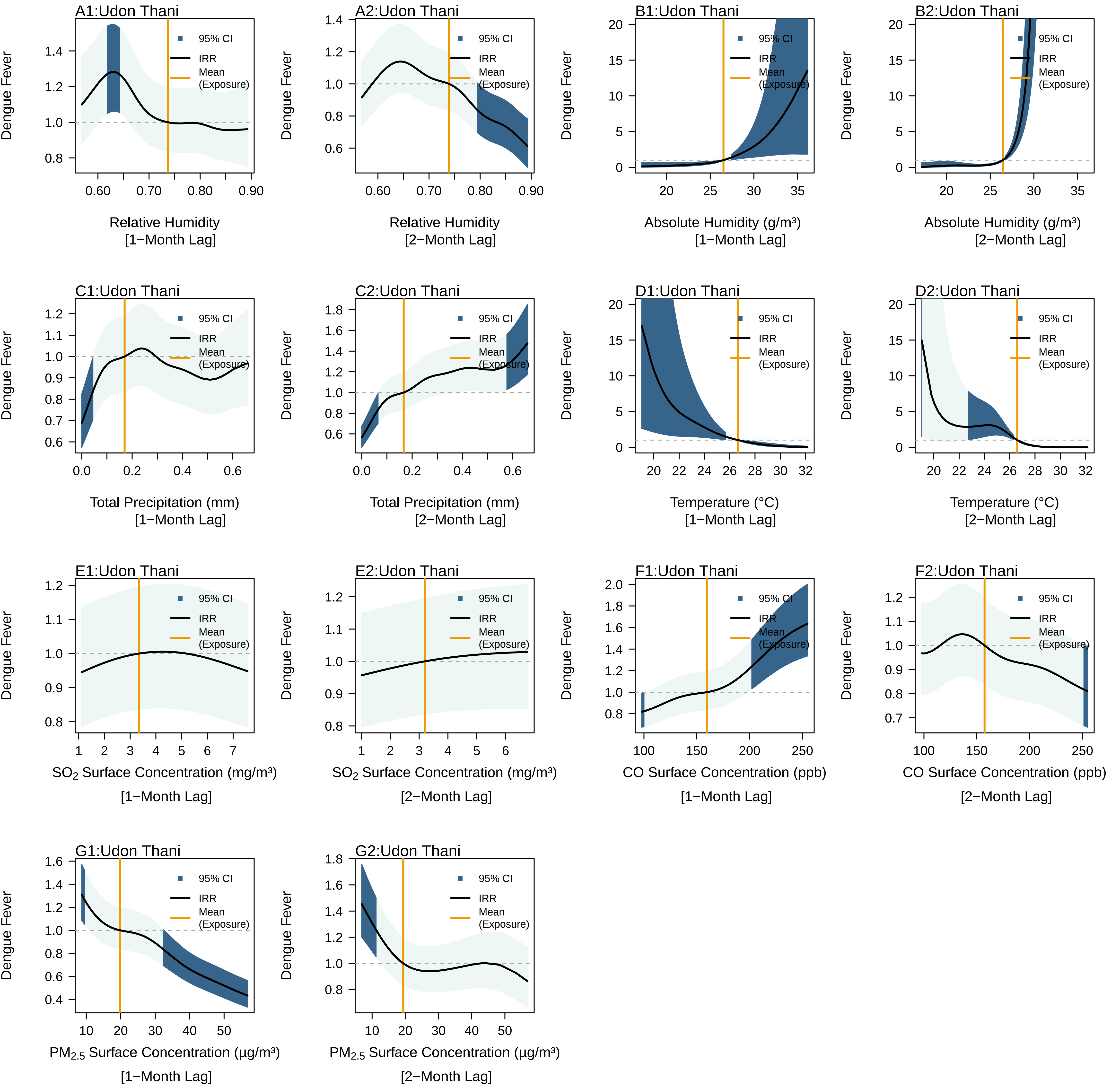

A1:Udon Thani
Dengue Fever
95% CI
IRR
Mean (Exposure)
Relative Humidity [1−Month Lag]
A2:Udon Thani
Dengue Fever
95% CI
IRR
Mean (Exposure)
Relative Humidity [2−Month Lag]
B1:Udon Thani
Dengue Fever
95% CI
IRR
Mean (Exposure)
Absolute Humidity (g/m³) [1−Month Lag]
B2:Udon Thani
Dengue Fever
95% CI
IRR
Mean (Exposure)
Absolute Humidity (g/m³) [2−Month Lag]
C1:Udon Thani
Dengue Fever
95% CI
IRR
Mean (Exposure)
Total Precipitation (mm) [1−Month Lag]
C2:Udon Thani
Dengue Fever
95% CI
IRR
Mean (Exposure)
Total Precipitation (mm) [2−Month Lag]
D1:Udon Thani
Dengue Fever
95% CI
IRR
Mean (Exposure)
Temperature (°C) [1−Month Lag]
D2:Udon Thani
Dengue Fever
95% CI
IRR
Mean (Exposure)
Temperature (°C) [2−Month Lag]
E1:Udon Thani
Dengue Fever
95% CI
IRR
Mean (Exposure)
SO2 Surface Concentration (mg/m³) [1−Month Lag]
E2:Udon Thani
Dengue Fever
95% CI
IRR
Mean (Exposure)
SO2 Surface Concentration (mg/m³) [2−Month Lag]
F1:Udon Thani
Dengue Fever
95% CI
IRR
Mean (Exposure)
CO Surface Concentration (ppb) [1−Month Lag]
F2:Udon Thani
Dengue Fever
95% CI
IRR
Mean (Exposure)
CO Surface Concentration (ppb) [2−Month Lag]
G1:Udon Thani
Dengue Fever
95% CI
IRR
Mean (Exposure)
PM2.5 Surface Concentration (μg/m³) [1−Month Lag]
G2:Udon Thani
Dengue Fever
95% CI
IRR
Mean (Exposure)
PM2.5 Surface Concentration (μg/m³) [2−Month Lag]

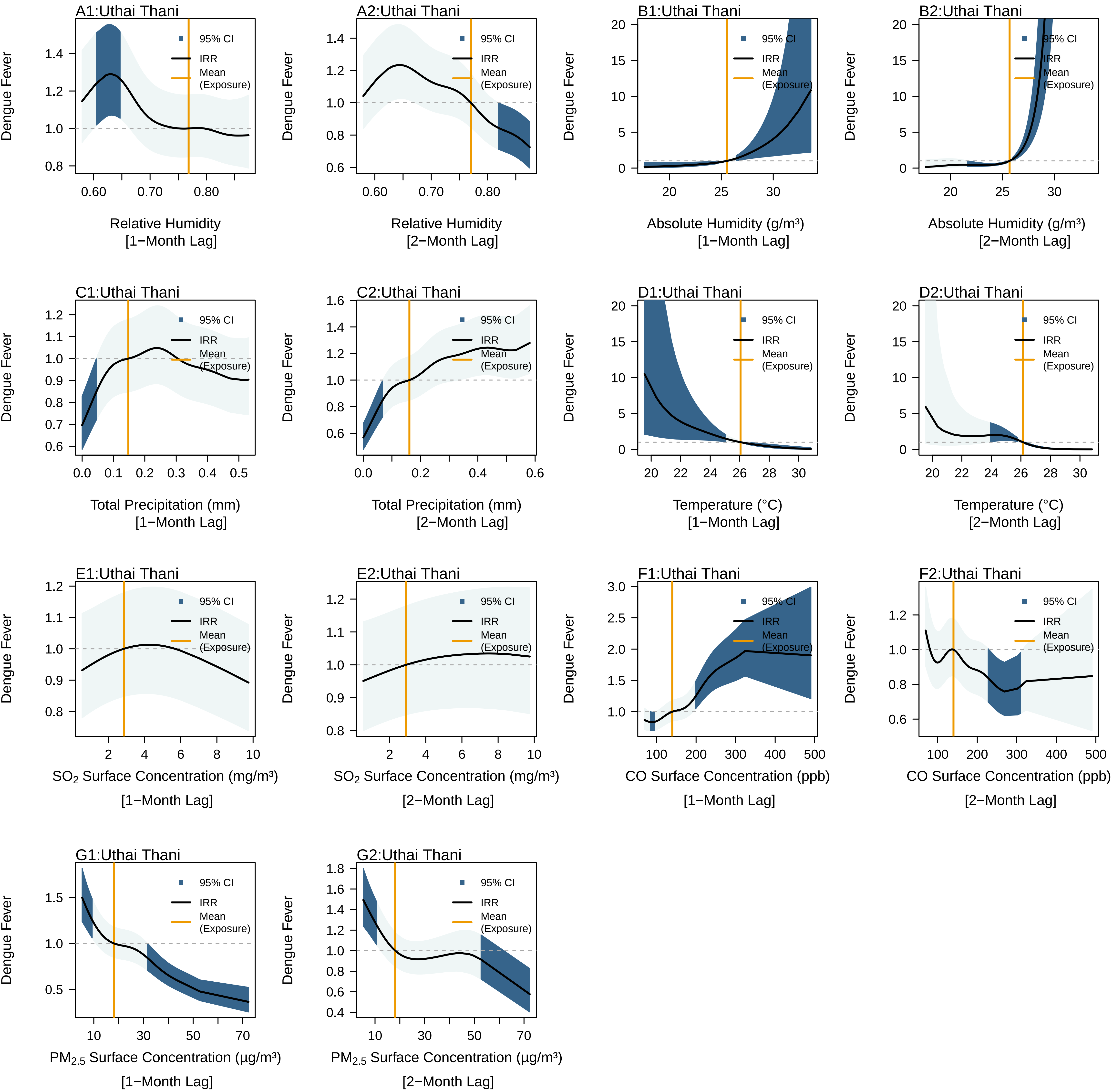

A1:Uthai Thani
Dengue Fever
95% CI
IRR
Mean (Exposure)
Relative Humidity [1−Month Lag]
A2:Uthai Thani
Relative Humidity [2−Month Lag]
B1:Uthai Thani
Absolute Humidity (g/m³) [1−Month Lag]
B2:Uthai Thani
Absolute Humidity (g/m³) [2−Month Lag]
C1:Uthai Thani
Total Precipitation (mm) [1−Month Lag]
C2:Uthai Thani
Total Precipitation (mm) [2−Month Lag]
D1:Uthai Thani
Temperature (°C) [1−Month Lag]
D2:Uthai Thani
Temperature (°C) [2−Month Lag]
E1:Uthai Thani
SO_2 Surface Concentration (mg/m³) [1−Month Lag]
E2:Uthai Thani
SO_2 Surface Concentration (mg/m³) [2−Month Lag]
F1:Uthai Thani
CO Surface Concentration (ppb) [1−Month Lag]
F2:Uthai Thani
CO Surface Concentration (ppb) [2−Month Lag]
G1:Uthai Thani
$PM_{2.5}$ Surface Concentration (µg/m³) [1−Month Lag]
G2:Uthai Thani
$PM_{2.5}$ Surface Concentration (µg/m³) [2−Month Lag]

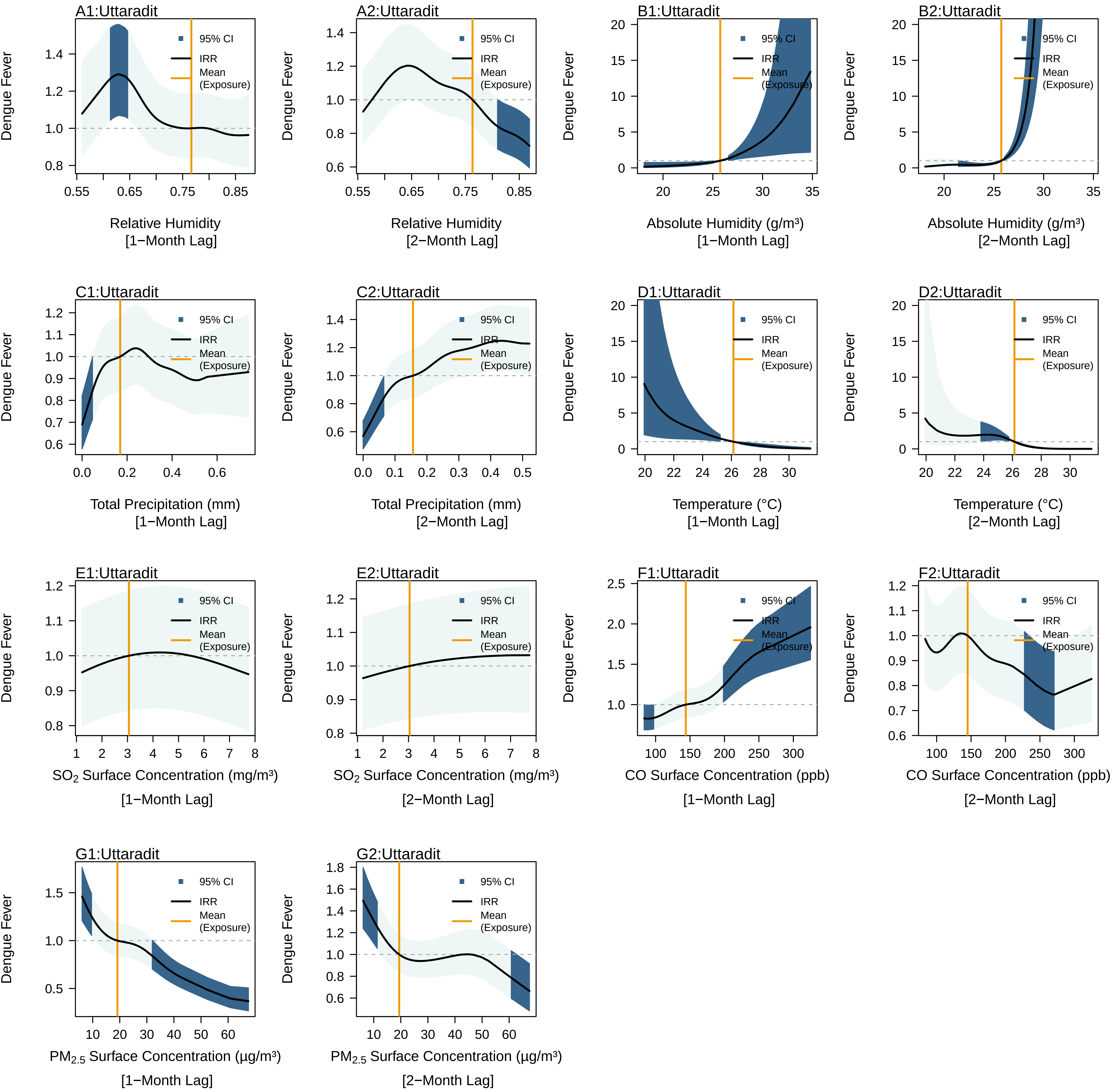
A1:Uttaradit
Dengue Fever
95% CI
IRR
Mean (Exposure)
Relative Humidity [1−Month Lag]
A2:Uttaradit
Dengue Fever
95% CI
IRR
Mean (Exposure)
Relative Humidity [2−Month Lag]
B1:Uttaradit
Dengue Fever
95% CI
IRR
Mean (Exposure)
Absolute Humidity (g/m³) [1−Month Lag]
B2:Uttaradit
Dengue Fever
95% CI
IRR
Mean (Exposure)
Absolute Humidity (g/m³) [2−Month Lag]
C1:Uttaradit
Dengue Fever
95% CI
IRR
Mean (Exposure)
Total Precipitation (mm) [1−Month Lag]
C2:Uttaradit
Dengue Fever
95% CI
IRR
Mean (Exposure)
Total Precipitation (mm) [2−Month Lag]
D1:Uttaradit
Dengue Fever
95% CI
IRR
Mean (Exposure)
Temperature (°C) [1−Month Lag]
D2:Uttaradit
Dengue Fever
95% CI
IRR
Mean (Exposure)
Temperature (°C) [2−Month Lag]
E1:Uttaradit
Dengue Fever
95% CI
IRR
Mean (Exposure)
SO_2 Surface Concentration (mg/m³) [1−Month Lag]
E2:Uttaradit
Dengue Fever
95% CI
IRR
Mean (Exposure)
SO_2 Surface Concentration (mg/m³) [2−Month Lag]
F1:Uttaradit
Dengue Fever
95% CI
IRR
Mean (Exposure)
CO Surface Concentration (ppb) [1−Month Lag]
F2:Uttaradit
Dengue Fever
95% CI
IRR
Mean (Exposure)
CO Surface Concentration (ppb) [2−Month Lag]
G1:Uttaradit
Dengue Fever
95% CI
IRR
Mean (Exposure)
$PM_{2.5}$ Surface Concentration (µg/m³) [1−Month Lag]
G2:Uttaradit
Dengue Fever
95% CI
IRR
Mean (Exposure)
$PM_{2.5}$ Surface Concentration (µg/m³) [2−Month Lag]

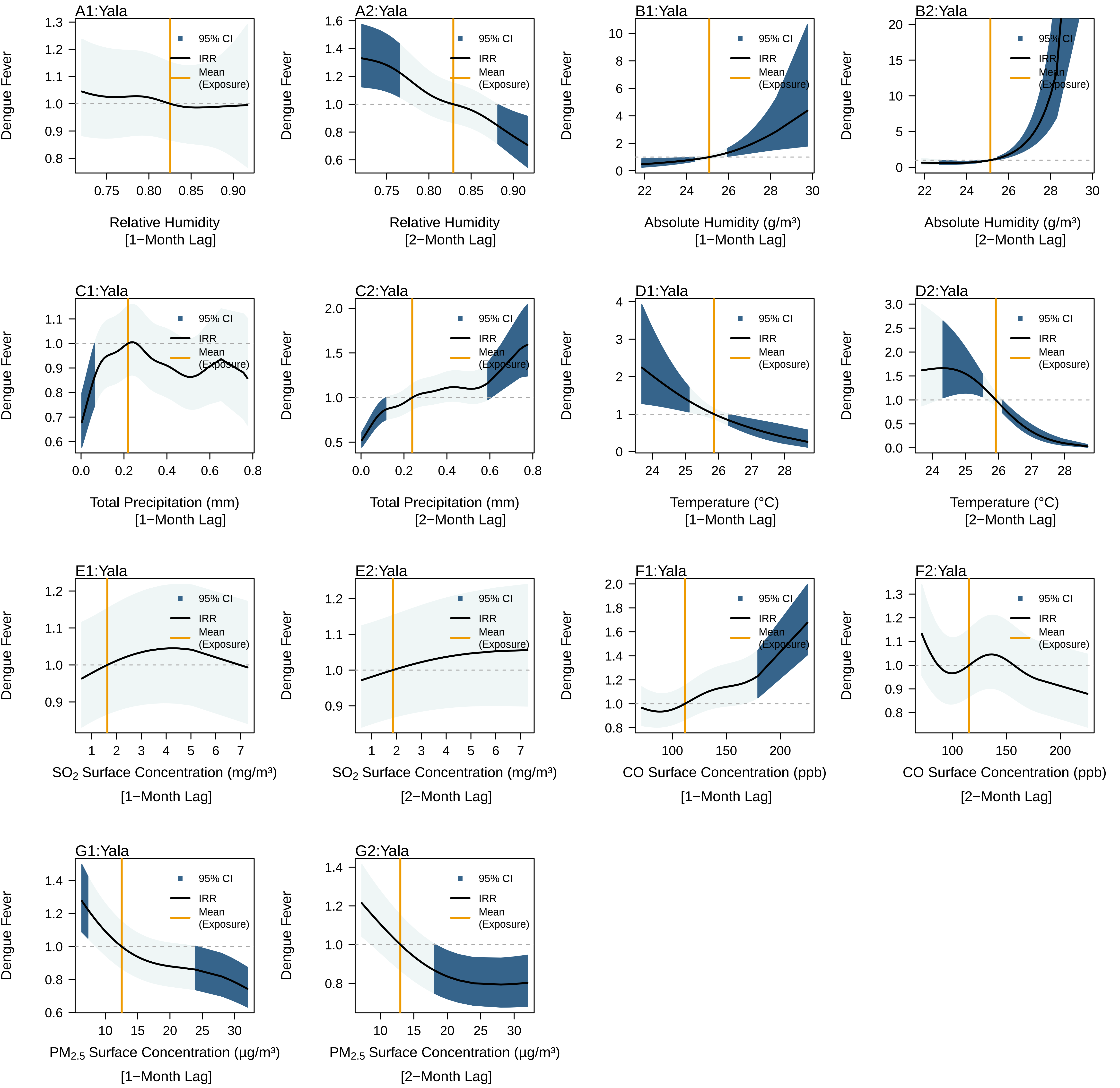

A1:Yala
A2:Yala
B1:Yala
B2:Yala
C1:Yala
C2:Yala
D1:Yala
D2:Yala
E1:Yala
E2:Yala
F1:Yala
F2:Yala
G1:Yala
G2:Yala
Dengue Fever
95% CI
IRR
Mean (Exposure)
Relative Humidity [1−Month Lag]
Relative Humidity [2−Month Lag]
Absolute Humidity (g/m³) [1−Month Lag]
Absolute Humidity (g/m³) [2−Month Lag]
Total Precipitation (mm) [1−Month Lag]
Total Precipitation (mm) [2−Month Lag]
Temperature (°C) [1−Month Lag]
Temperature (°C) [2−Month Lag]
SO2 Surface Concentration (mg/m³) [1−Month Lag]
SO2 Surface Concentration (mg/m³) [2−Month Lag]
CO Surface Concentration (ppb) [1−Month Lag]
CO Surface Concentration (ppb) [2−Month Lag]
PM2.5 Surface Concentration (µg/m³) [1−Month Lag]
PM2.5 Surface Concentration (µg/m³) [2−Month Lag]

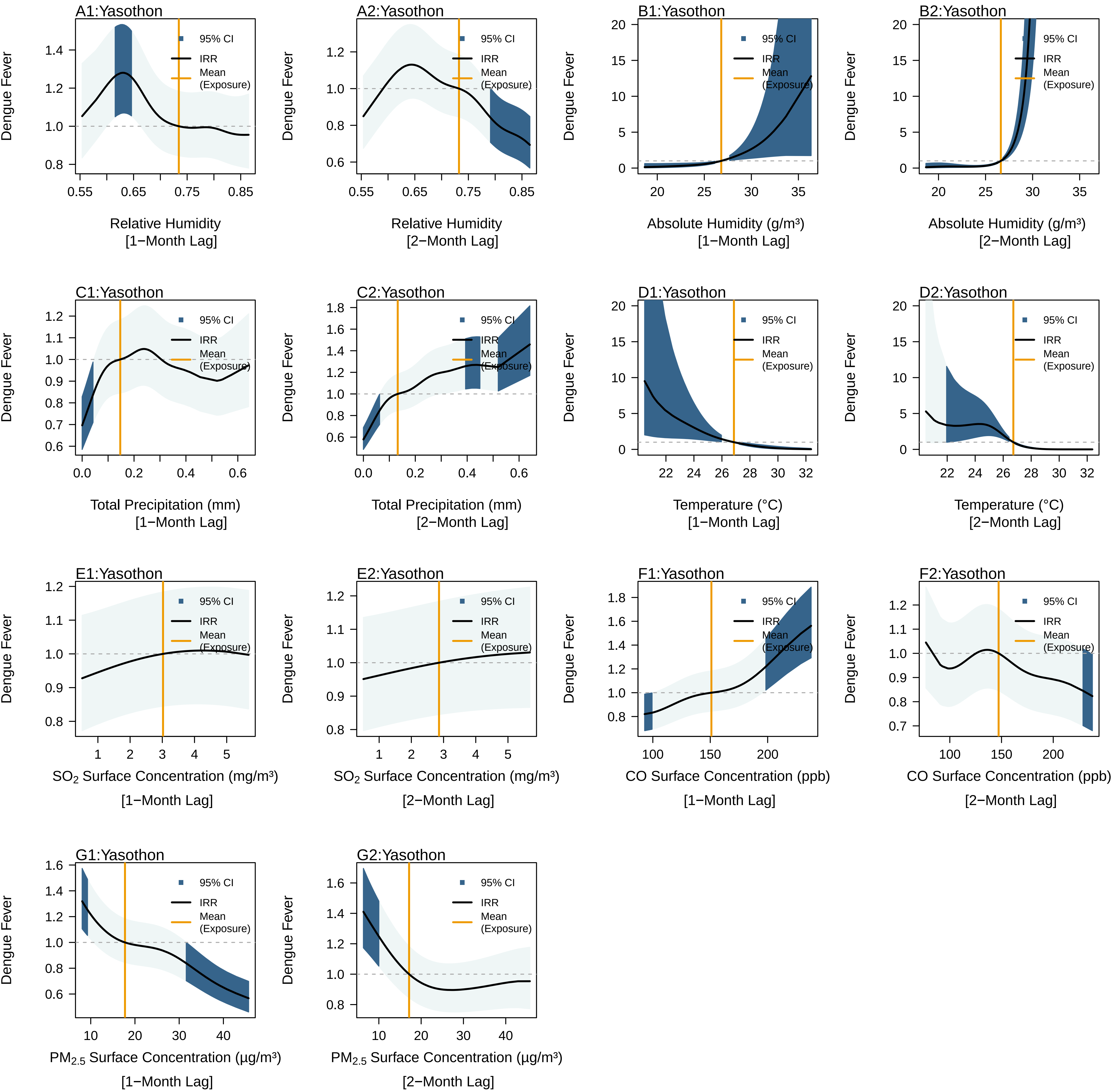
A1:Yasothon
Dengue Fever
95% CI
IRR
Mean (Exposure)
Relative Humidity [1−Month Lag]
A2:Yasothon
Dengue Fever
95% CI
IRR
Mean (Exposure)
Relative Humidity [2−Month Lag]
B1:Yasothon
Dengue Fever
95% CI
IRR
Mean (Exposure)
Absolute Humidity (g/m³) [1−Month Lag]
B2:Yasothon
Dengue Fever
95% CI
IRR
Mean (Exposure)
Absolute Humidity (g/m³) [2−Month Lag]
C1:Yasothon
Dengue Fever
95% CI
IRR
Mean (Exposure)
Total Precipitation (mm) [1−Month Lag]
C2:Yasothon
Dengue Fever
95% CI
IRR
Mean (Exposure)
Total Precipitation (mm) [2−Month Lag]
D1:Yasothon
Dengue Fever
95% CI
IRR
Mean (Exposure)
Temperature (°C) [1−Month Lag]
D2:Yasothon
Dengue Fever
95% CI
IRR
Mean (Exposure)
Temperature (°C) [2−Month Lag]
E1:Yasothon
Dengue Fever
95% CI
IRR
Mean (Exposure)
SO2 Surface Concentration (mg/m³) [1−Month Lag]
E2:Yasothon
Dengue Fever
95% CI
IRR
Mean (Exposure)
SO2 Surface Concentration (mg/m³) [2−Month Lag]
F1:Yasothon
Dengue Fever
95% CI
IRR
Mean (Exposure)
CO Surface Concentration (ppb) [1−Month Lag]
F2:Yasothon
Dengue Fever
95% CI
IRR
Mean (Exposure)
CO Surface Concentration (ppb) [2−Month Lag]
G1:Yasothon
Dengue Fever
95% CI
IRR
Mean (Exposure)
PM2.5 Surface Concentration (μg/m³) [1−Month Lag]
G2:Yasothon
Dengue Fever
95% CI
IRR
Mean (Exposure)
PM2.5 Surface Concentration (μg/m³) [2−Month Lag]
